# Safety of Stimulants Across Patient Populations: A Meta-Analysis

**DOI:** 10.1001/jamanetworkopen.2025.9492

**Published:** 2025-05-09

**Authors:** Henrique Nunes Pereira Oliva, Tiago Paiva Prudente, Talia F. Mayerson, Marcella M. Mignosa, Isabela Oliveira Oliva, Marc N. Potenza, Oluwole O. Jegede, Gustavo A. Angarita

**Affiliations:** 1 Department of Psychiatry, Yale University School of Medicine, New Haven, Connecticut; 2 Clinical Neuroscience Research Unit, Connecticut Mental Health Center, New Haven; 3 Connecticut Mental Health Center, New Haven; 4 School of Medicine, Universidade Federal de Goiás, Goiânia, Goiás, Brazil; 5 School of Medicine, Centro Universitario FIPMoc, Montes Claros, Minas Gerais, Brazil; 6 Child Study Center, Yale University School of Medicine, New Haven, Connecticut; 7 Department of Neuroscience, Yale University, New Haven, Connecticut; 8 Connecticut Council on Problem Gambling, Wethersfield; 9 Wu Tsai Institute, Yale University, New Haven, Connecticut

## Abstract

**Importance:**

The use of stimulant medications has expanded substantially beyond the traditional treatment of attention-deficit/hyperactivity disorder (ADHD) to encompass a variety of other clinical conditions. Understanding the safety of these medications is important as their use increases across diverse patient populations.

**Objective:**

To assess the safety of stimulant medications as reported in randomized clinical trials (RCTs) investigating methylphenidate, lisdexamfetamine, and other amphetamines.

**Data Sources:**

A comprehensive literature search was conducted from July 1, 2024, through February 28, 2025, using CINAHL, Embase, PubMed or MEDLINE, ScienceDirect, and Web of Science for studies published since 2000. Keywords included *safety, adverse event, side effect, amphetamine, dextroamphetamine, stimulant, lisdexamfetamine,* and *methylphenidate*.

**Study Selection:**

RCTs published between January 1, 2000, and December 13, 2024, were included. These trials investigated the safety of stimulants in various clinical conditions, including ADHD, depression, binge eating disorder, schizophrenia, Alzheimer disease, and stimulant use disorders as well as in healthy individuals. Trials not focused on safety or adverse events (AEs) of stimulants, nonoriginal research, nonhuman research, trials with concomitant prescriptions other than stimulants, and trials without a placebo group were excluded.

**Data Extraction and Synthesis:**

Data extraction followed the Preferred Reporting Items for Systematic Reviews and Meta-Analyses (PRISMA) reporting guideline. Independent reviewers extracted study data, and a random-effects model was used to pool results. Heterogeneity was assessed using the *I*^2^ statistic.

**Main Outcomes and Measures:**

The primary outcome was the risk ratio (RR) of developing any AE in participants taking stimulants vs placebo.

**Results:**

A total of 93 RCTs were included after exclusions. The methodological quality assessment of the included trials showed overall low or unclear risk of bias. Trials with a duration of up to 52 weeks showed that stimulant medications were associated with an increased risk of overall AEs compared with placebo (RR, 1.34; 90% CI, 1.27-1.41), with high heterogeneity (*I*^2^ = 67%). Statistical significance of this finding was maintained when subgroups (ie, methylphenidate, lisdexamfetamine, and other amphetamines) were separately analyzed.

**Conclusions and Relevance:**

This meta-analysis found an increased risk of overall AEs associated with stimulants compared with placebo. Future research could provide more standardized and consistent assessments of this outcome and may improve understanding about misuse risk.

## Key Points

**Question:**

What are the adverse events (AEs) and safety profiles of stimulants across different clinical conditions?

**Findings:**

In this meta-analysis of 93 randomized clinical trials, stimulants such as methylphenidate, lisdexamfetamine, and other amphetamines were associated with an increased risk of overall AEs compared with placebo.

**Meaning:**

The finding suggests that longer longitudinal studies are warranted to improve understanding about stimulant misuse risk and inform treatment strategies for attention-deficit/hyperactivity disorder and other psychiatric disorders.

## Teaser

This meta-analysis evaluates risk of adverse events associated with stimulant medications among patients with a variety of conditions.

## Introduction

Between 2006 and 2016, stimulant prescriptions in the US increased by 250%, and according to the Centers for Diseases Control and Prevention, these prescriptions are steadily growing in number.^1,2^ While most stimulants have historically been prescribed for children and young adults, these drugs are increasingly prescribed for other populations, including older adults.^1^ Although stimulants have been prescribed primarily for treating attention-deficit/hyperactivity disorder (ADHD), research and clinical interest over the past 2 decades suggest other possible uses, such as therapy for depressive eating, sleep (eg, narcolepsy), and stimulant use disorders (eg, cocaine use disorder).^3,4,5,6^ Other therapeutic applications, together with the loosening of legislation and insurance policies surrounding telemedicine during the COVID-19 pandemic, likely underlies increased prescribing of stimulants in the US.^2^

The increasing number of stimulant prescriptions and the expanding range of therapeutic applications highlight the need to better understand the safety of stimulants, particularly in relation to drug diversion, misuse,^7,8^ and adult ADHD diagnoses.^1^ The long-term safety of these drugs given the stimulant treatment-emergent adverse events (TEAEs) associated with cardiovascular health, sleep, appetite, or growth is of particular interest. Prolonged exposure to stimulants lasting 5 to 14 years may increase the risk of cardiovascular disease.^9^ In response to these concerns, several literature reviews examined the safety of stimulants in treating conditions, such as ADHD, depressive disorders, and sleep disorders.^4,5,10^ However, many of these reviews relied on studies with varying methodologies and often focused on a single drug formulation or type.^3,4,5,6,7,10,11,12,13^

While previous reviews offer valuable insights,^14,15^ none in the past decade has exclusively analyzed the safety of stimulants using data from placebo-controlled randomized clinical trials (RCTs). Studies from the past 2 decades provide updated data, reflecting current clinical practices and including long-acting formulations, novel delivery systems, and flexible-dose designs.^16^ Given the emerging recognition of the nocebo effects of stimulants, assessing RCTs is important.^17^ Furthermore, previous reviews may have missed newer medication formulations, flexible-dose designs, and broader clinical applications of stimulants beyond ADHD.^18^ The present meta-analysis aimed to fill this gap by assessing the safety of stimulant medications prescribed for various diagnoses, including ADHD, depression, binge-eating disorder (BED), schizophrenia, Alzheimer disease, and stimulant use disorders, as reported in RCTs investigating methylphenidate, lisdexamfetamine, and other amphetamines.

## Methods

### Search Strategy

Literature search was conducted from July 1, 2024, through February 28, 2025, using CINAHL, Embase, PubMed or MEDLINE, ScienceDirect, and Web of Science for studies published since 2000. The search strategy for PubMed included the following: ((“safety”[Title/Abstract] OR “adverse event*”[Title/Abstract] OR “side effect*”[Title/Abstract]) AND (“amphetamine”[Title/Abstract] OR “dextroamphetamine”[Title/Abstract] OR “stimulant*”[Title/Abstract] OR “lisdexamfetamine”[Title/Abstract] OR “methylphenidate”[Title/Abstract])) AND ((clinicaltrial[Filter]) AND (fft[Filter])) (eTable 1 in Supplement 1). The protocol was registered on PROSPERO (CRD42024542765). We followed the Preferred Reporting Items for Systematic Reviews and Meta-Analyses (PRISMA) reporting guideline.

### Identification and Selection of Studies

RCTs published between January 1, 2000, and December 13, 2024, that reported on the safety or adverse events (AEs) of amphetamine, dextroamphetamine, lisdexamfetamine, methylphenidate, or other stimulants were included. Identified articles were imported to an online tool (Rayyan)^19^ for duplicate removal and screening. Study selection and data extraction were performed independently by 2 reviewers (H.N.P.O. and T.P.P.). In cases of disagreement, a third reviewer was consulted (G.A.A.). Exclusion criteria included the following: RCTs not focused on safety or AEs of the stimulants, nonoriginal research (eg, letters, commentaries, and meeting abstracts), nonhuman research, trials with concomitant prescriptions other than stimulants, and trials without a placebo group.

Three authors (H.N.P.O., I.O.O., and T.P.P.) independently assessed the methodological quality of the included studies using the Cochrane Risk-of-Bias tool for RCTs, version 2 (RoB 2; Cochrane Collaboration).^20^ Publication bias was appraised using the rank correlation test,^21^ the fail-safe N method,^22^ and the regression test for funnel plot asymmetry^23^ in jamovi^24^ (Jamovi Project).

### Statistical Analysis

The primary outcome was the risk ratio (RR) of developing any AE in participants taking stimulants vs placebo. The secondary outcomes included RRs for specific AEs (decreased appetite, insomnia, headaches, dry mouth, nausea, irritability, and anxiety), cardiovascular changes measured by mean differences (MDs) in vital signs (heart rate, systolic blood pressure [SBP], and diastolic blood pressure [DBP]), and reported emergence of psychosis or drug misuse. We also looked for reports of growth delays. We used RevMan 5 software (Cochrane Collaboration)^25^ to create forest plots and funnel plots. We extracted data (ie, number of participants who had AEs in each group and the total number of participants in each group) to generate analyses with the measure of association (RR). For continuous data, forest plots were generated using the MD between the intervention group (stimulant group) and the control group (placebo group). Subgroup analyses were performed, separating the data according to the type of stimulant under investigation: methylphenidate, lisdexamfetamine, and other amphetamines.

A sensitivity analysis was performed for all forest plots to detect if individual RCTs deviated from overall results. To evaluate heterogeneity across studies, both the Cochran *Q* test and the *I*^2^ index were used, in which heterogeneity was considered not important (*I*^2^ = 0%-40%), moderate (*I*^2^ = 40%-60%), substantial (*I*^2^ = 60%-90%), or considerable (*I*^2^ = 90%-100%). The results with heterogeneity above 50% were pooled using random-effects models, while fixed-effects models were used otherwise. Additionally, using R version 4.4.2 (R Project for Statistical Computing),^26^ we conducted a bayesian analysis to explore potential causal interpretations.^27^

Two-sided *P* < .10 indicated statistical significance. Data analysis was performed with RevMan 5 (Cochrane Collaboration).

## Results

### Overview of Included and Analyzed Trials

**Figure 1.**
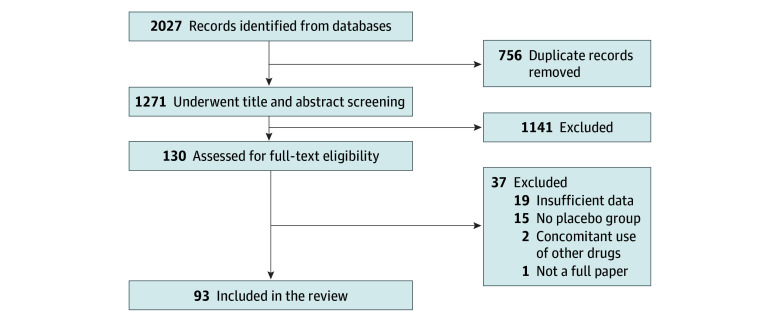
PRISMA Flow Diagram of Studies

**Figure 2.**
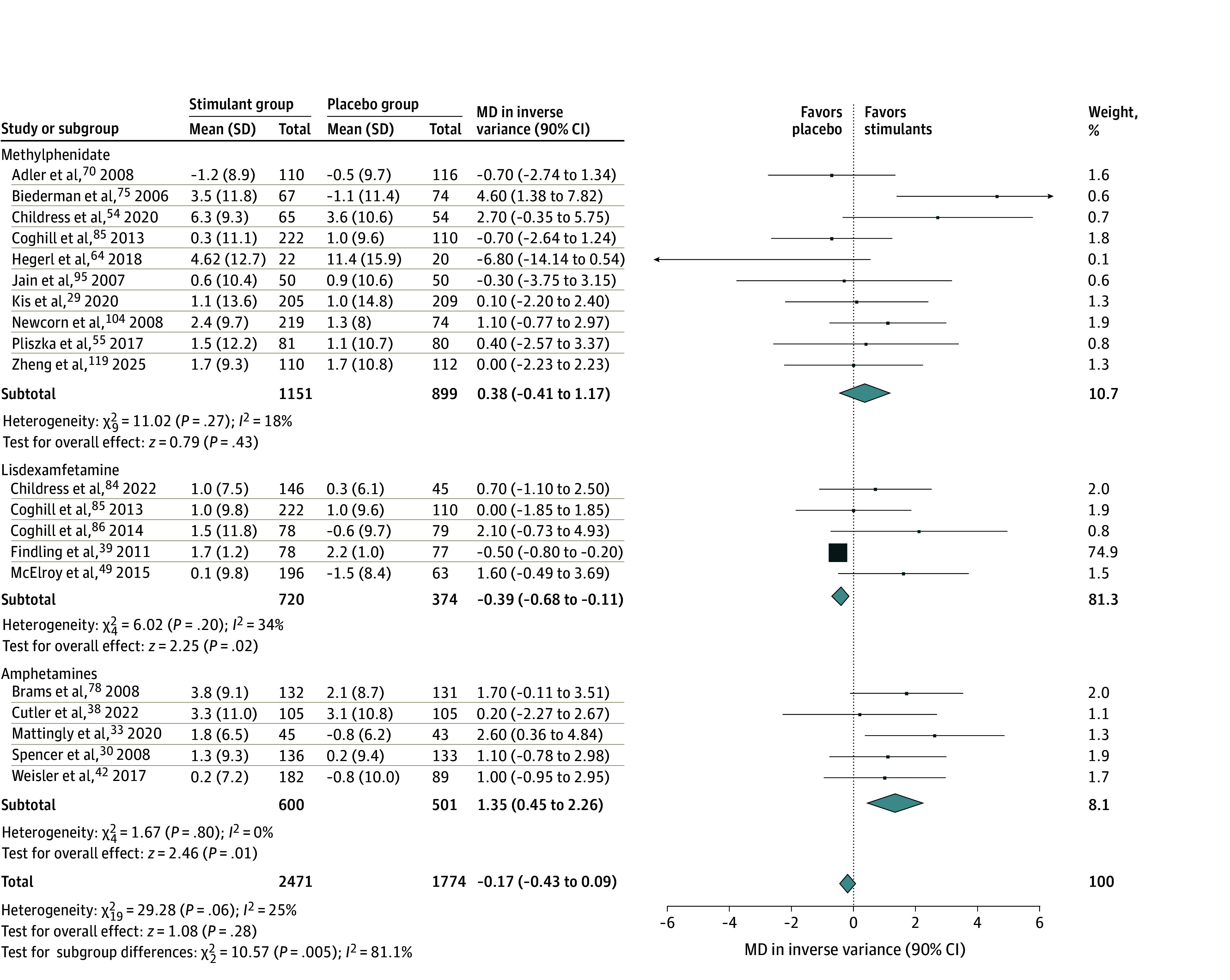
Forest Plot Comparing the Mean Changes in Systolic Blood Pressure in Patients Receiving Stimulants vs Placebo Diamonds represent the pooled mean difference (MD) estimates for systolic blood pressure for each stimulant subgroup and the overall analysis. Squares represent individual study estimates. Error bars represent 90% CIs.

**Figure 3.**
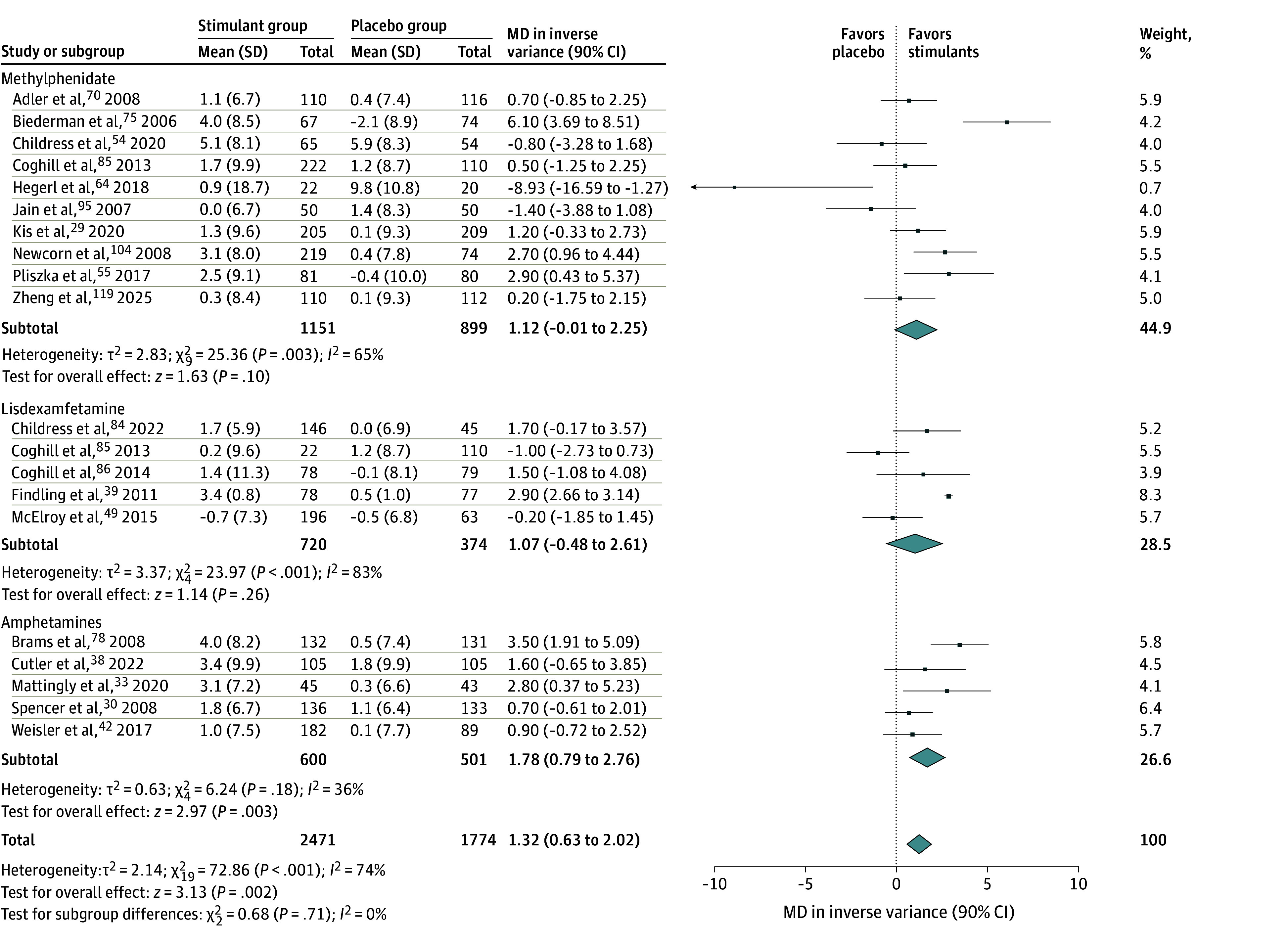
Forest Plot Comparing the Mean Changes in Diastolic Blood Pressure in Patients Receiving Stimulants vs Placebo Diamonds represent the pooled mean difference (MD) estimates for diastolic blood pressure for each stimulant subgroup and the overall analysis. Squares represent individual study estimates. Error bars represent 90% CIs.

**Figure 4.**
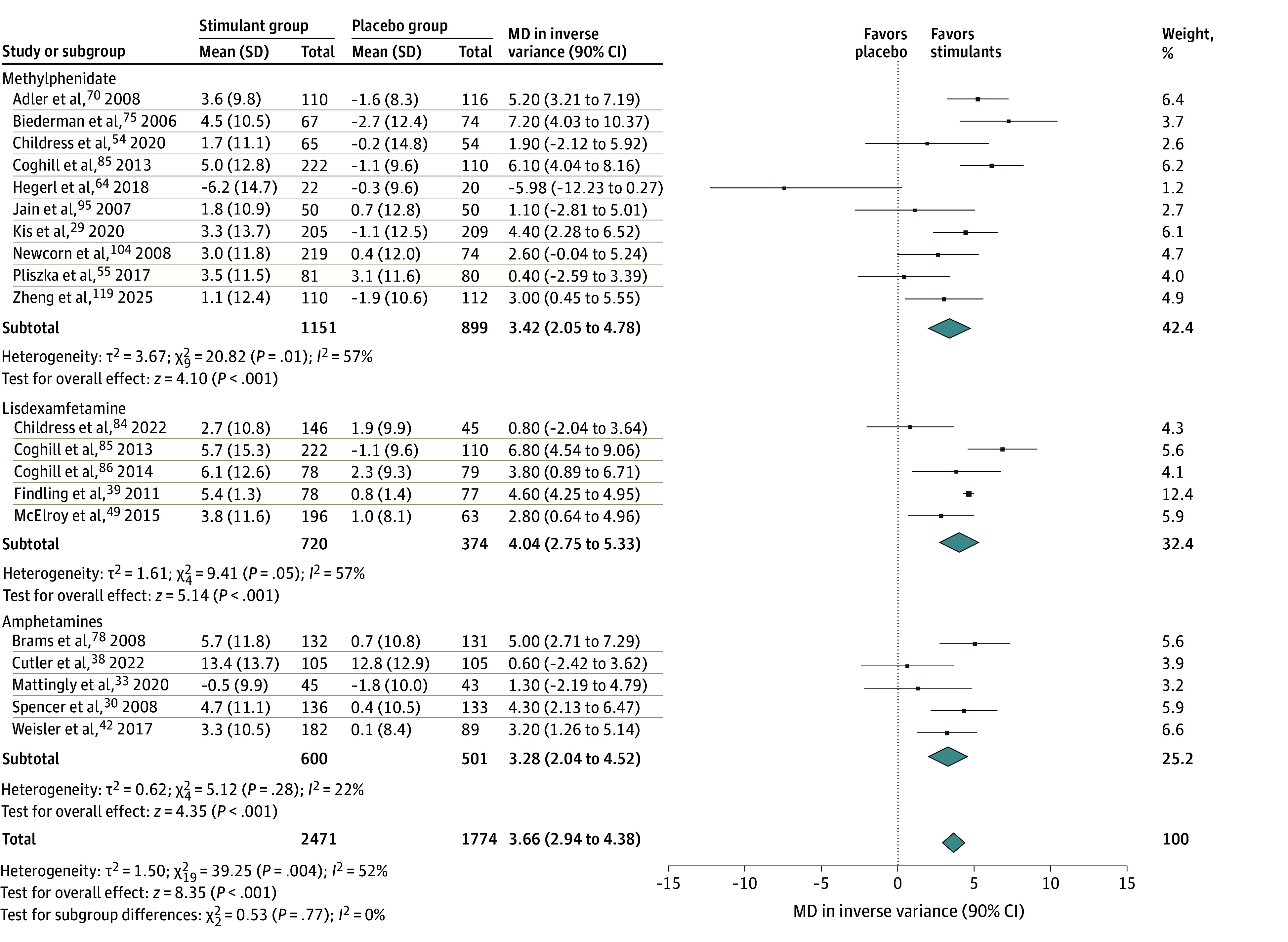
Forest Plot Comparing the Mean Changes in Heart Rate in Patients Receiving Stimulants vs Placebo Diamonds represent the pooled mean difference (MD) estimates for heart rate for each stimulant subgroup and the overall analysis. Squares represent individual study estimates. Error bars represent 90% CIs.

The initial search retrieved 2027 RCTs, of which 756 were duplicates. After screening the titles and abstracts of 1271 articles, 1141 were excluded. Of the remaining 130 articles read in their entirety, 93 RCTs^28,29,30,31,32,33,34,35,36,37,38,39,40,41,42,43,44,45,46,47,48,49,50,51,52,53,54,55,56,57,58,59,60,61,62,63,64,65,66,67,68,69,70,71,72,73,74,75,76,77,78,79,80,81,82,83,84,85,86,87,88,89,90,91,92,93,94,95,96,97,98,99,100,101,102,103,104,105,106,107,108,109,110,111,112,113,114,115,116,117,118,119^ were included, of which 2 were retrieved from the reference lists of previously published articles (Figure 1; eTable 2 in Supplement 1). Trial characteristics are detailed in the Table and eTable 3 in Supplement 1. The overall population included 11 034 males (67.4%) and 5348 females (32.6%). The methodological quality assessment of the included RCTs showed overall low or unclear risk of bias (eFigure 9 in Supplement 1). We meta-analyzed the most commonly mentioned AEs (Figure 2, Figure 3, and Figure 4; eFigures 1-8 in Supplement 1).

**Table.** Stimulant Safety Randomized Clinical Trial Summary

Source	Stimulant	Formulation (if specific)	Sample size, No.	Male sex, No. (%)	Associated condition	Participants with AEs, No. (%)^a^
Adler et al,^69^ 2008	Lisdexamfetamine	NA	Control: 62 Intervention: 358	228 (54.2)	ADHD	Lisdexamfetamine: 282 (78.7) Placebo: 36 (58.0)
Adler et al,^120^ 2009	Methylphenidate	OROS	Control: 62 Intervention: 358	228 (54.2)	ADHD	34 (8.3)
Adler et al,^70^ 2009	Methylphenidate	OROS	Control: 116 Intervention: 110	127 (56.2)	ADHD	Methylphenidate: 93 (84.5) Placebo: 74 (63.8)
Adler et al,^72^ 2013	Lisdexamfetamine	NA	Control: 80 Intervention: 79	NR	ADHD + EFI	Lisdexamfetamine: 62 (78.5) Placebo: 47 (58.8)
Ahmann et al,^73^ 2001	Adderall (amphetamine)	NA	Control: 154^b^ Intervention: 154^b^	113 (73.0)	ADHD	NR
Armstrong et al,^74^ 2012	Methylphenidate	OROS	Control: 167^b^ Intervention: 167^b^	115 (69.0)	ADHD	NR
Biederman et al,^75^ 2006	Methylphenidate	OROS	Control: 74 Intervention: 67	73 (51.7)	NR	NR
Biederman et al,^35^ 2007	Lisdexamfetamine and mixed amphetamine salts	Lisdexamfetamine: oral; mixed amphetamine salts: XR	Control: 52 Intervention: 52	33 (63.5)	ADHD	Lisdexamfetamine: 8 (16.0) Mixed amphetamine salts: 9 (18.0) Placebo: 8 (16.0)
Biederman et al,^76^ 2007	Lisdexamfetamine	NA	Control: 72 Intervention: 218	201 (69.0)	ADHD	Lisdexamfetamine: 162 (74.3) Placebo: 34 (47.2)
Biederman et al,^53^ 2003	Methylphenidate	LA	Control: 71 Intervention: 65	104 (76.5)	ADHD	Methylphenidate: 16 (24.6) Placebo: 17 (23.9)
Bouffard et al,^77^ 2003	Methylphenidate	NR	Control 30 Intervention: 30	24 (80.0)	ADHD	NR
Brams et al,^78^ 2008	Dexmethylphenidate	ER	Control: 86^b^ Intervention: 86^b^	53 (61.6)	ADHD	Methylphenidate: 15 (17.4) Placebo: 19 (22.1)
Brams et al,^79^ 2011	Lisdexamfetamine	NA	Control: 142^b^ Intervention: 142^b^	88 (62.0)	ADHD	NR
Brams et al,^36^ 2018	SHP465 (triple-bead mixed amphetamine salts)	NA	Control: 131 Intervention: 132	163 (62.0)	ADHD	SHP465: 70 (53.0) Placebo: 34 (26.0)
Bron et al,^80^ 2014	Methylphenidate	OROS	Control: 22^b^ Intervention: 22^b^	17 (77.3)	ADHD	Methylphenidate: 17 (77.0) Placebo: 10 (46.0)
Brown et al,^81^ 2010	Lisdexamfetamine	NA	Control: 127^b^ Intervention: 127^b^	88 (62.0)	ADHD	NR
Buitelaar et al,^45^ 2012	Methylphenidate	OROS	Control: 22 Intervention: 23	18 (40.0)	ADHD	Methylphenidate: 7 (30.5) Placebo: 8 (36.4)
Casas et al,^62^ 2013	Methylphenidate	OROS	Control: 97 Intervention: 182	146 (52.0)	ADHD	54 mg Methylphenidate: 77 (86.5) 72 mg Methylphenidate: 84 (91.3) Placebo: 76 (78.4)
Childress et al,^82^ 2009	Dexmethylphenidate	ER	Control: 65 Intervention: 188	163 (64.4)	ADHD	Methylphenidate: 116 (63.7) Placebo: 36 (57.1)
Childress et al,^37^ 2015	Evekeo (racemic amphetamine sulfate)	NA	Control: 97 Intervention: 97	59 (60.8)	ADHD	Evekeo: 10 (10.3) Placebo: 6 (6.2)
Childress et al,^32^ 2018	Amphetamine	ER	Control: 48 Intervention: 51	68 (68.7)	ADHD	Amphetamine: 9 (17.3) Placebo: 6 (12.5)
Childress et al,^54^ 2020	Methylphenidate	DR and ER	Control: 54 Intervention: 65	80 (67.5)	ADHD	Methylphenidate: 24 (36.9) Placebo: 22 (40.7)
Childress et al,^56^ 2020	PRC-063 (ER-methylphenidate)	ER	Control: 73 Intervention: 75	96 (65.4)	ADHD	Methylphenidate: 18 (24.0) Placebo: 7 (9.6)
Childress et al,^83^ 2020	Aptensio XR (methylphenidate)	XR	Control: 50 Intervention: 39	68 (75.6)	ADHD	Methylphenidate: 10 (25.6) Placebo: 6 (12)
Childress et al,^50^ 2022	PRC-063 (ER-methylphenidate)	DR and ER	Control: 118 Intervention: 121	108 (45.3)	ADHD	Methylphenidate: 25 (20.7) Placebo: 18 (15.3)
Childress et al,^84^ 2022	Lisdexamfetamine	NA	Control: 45 Intervention: 146	129 (67.1)	ADHD	Lisdexamfetamine: 68 (46.6) Placebo: 19 (42.2)
Chronis-Tuscano et al,^58^ 2008	Methylphenidate	OROS	Control: 23 Intervention: 23	13 (57.0)	ADHD	NR
Coghill et al,^85^ 2013	Lisdexamfetamine and methylphenidate	Lisdexamfetamine: NA Methylphenidate: OROS	Control: 110 Intervention: 222 (111, lisdexamfetamine; 111, methylphenidate)	268 (80.7)	ADHD	Lisdexamfetamine: 80 (72.1) Methylphenidate: 72 (64.9) Placebo: 63 (57.3)
Coghill et al,^86^ 2014	Lisdexamfetamine	NA	Control: 79 Intervention: 78	123 (78.3)	ADHD	Lisdexamfetamine: 31 (39.7) Placebo: 20 (25.3)
Cutler et al,^40^ 2022	Amphetamine	ER	Control: 65 Intervention: 62	76 (59.8)	ADHD	Amphetamine: 54 (87.0) Placebo: 35 (54.0)
Cutler et al,^38^ 2022	Dextroamphetamine transdermal system	MTS	Control: 105^b^ Intervention: 105^b^	76 (69.1)	ADHD	Dextroamphetamine transdermal system: 44 (41.9) Placebo: 43 (41)
Dupaul et al,^44^ 2012	Lisdexamfetamine and methylphenidate	NA	Control: 24^b^ Intervention: 24^b^	29 (62.5)	ADHD	NR
Ermer et al,^68^ 2020	Lisdexamfetamine	NA	Control: 6 Intervention: 26	24 (70.8)	Healthy	Lisdexamfetamine: 20 (76.9) Placebo: 1 (16.6)
Faraone et al,^41^ 2021	AR19 (amphetamine sulfate)	Manipulation-resistant formulation	Control: 107 Intervention: 214	174 (54.4)	ADHD	NR
Findling et al,^87^ 2008	Methylphenidate	MTS and OROS	Control: 85 Intervention: 189 (98, MTS; 91, OROS)	181 (66.3)	ADHD	MTS: 74 (75.5) OROS: 63 (69.2) Placebo: 49 (57.6)
Findling et al,^39^ 2011	Lisdexamfetamine	Oral	Control: 77 Intervention: 233	217 (70.3)	ADHD	Lisdexamfetamine: 160 (68.7) Placebo: 45 (58.4)
Froehlich et al,^88^ 2020	Methylphenidate	OROS	Control: 171^b^ Intervention: 171^b^	121 (71.0)	ADHD	NR
Galloway et al,^67^ 2011	Dextroamphetamine transdermal system	SR	Control: 30 Intervention: 30	34 (56.6)	SUD (methamphetamine dependence)	NR
Ginsberg and Lindefors,^89^ 2012	Methylphenidate	OROS	Control: 15 Intervention: 15	30 (100)	ADHD	NR
Ginsberg et al,^90^ 2014	Methylphenidate	LA	Control: 82 Intervention: 216	160 (53.7)	ADHD	Methylphenidate: 175 (81.0) Placebo: 65 (79.3)
Goodman et al,^91^ 2017	Methylphenidate	OROS	Control: 175 Intervention: 174	184 (52.7)	ADHD	Methylphenidate: 126 (72.4) Placebo: 87 (49.7)
Greenhill et al,^92^ 2002	Methylphenidate	MR	Control: 161 Intervention: 155	257 (81.8)	ADHD	Methylphenidate: 80 (51.6) Placebo: 61 (37.9)
Hegerl et al,^64^ 2018	Methylphenidate	IR	Control: 20 Intervention: 22	22 (52.3)	Acute mania	NR
Huang et al,^93^ 2021	Methylphenidate	ORADUR	Control: 101^b^ Intervention: 110^b^	73 (73.0)	ADHD	Methylphenidate: 79 (71.8) Placebo: 10 (9.9)
Huss et al,^94^ 2014	Methylphenidate	LA	Control: 180 Intervention: 542	395 (54.5)	ADHD	Methylphenidate: 401 (74.0) Placebo: 108 (60.0)
Jain et al,^95^ 2007	Methylphenidate	MLR	Control: 50^b^ Intervention: 50^b^	30 (62.5)	ADHD	Methylphenidate: 42 (84.0) Placebo: 29 (58.0)
Jasinski and Krishnan,^66^ 2009	Lisdexamfetamine	Oral	Control: 36^b^ Intervention: 36^b^	32 (84.0)	History of SUD (stimulant use)	Lisdexamfetamine: 15 (41.0) Placebo: 6 (17.0)
Kis et al,^29^ 2020	Methylphenidate	NR	Control: 209 Intervention: 205	2017 (50.1)	ADHD	Methylphenidate: 197 (96.1) Placebo: 184 (88.0)
Konstenius et al,^96^ 2014	Methylphenidate	OROS	Control: 27 Intervention: 27	54 (100)	ADHD and SUD (amphetamine)	NR
Kooij et al,^97^ 2004	Methylphenidate	NR	Control: 45^b^ Intervention: 45^b^	24 (53.3)	ADHD	Methylphenidate: 37 (82.0) Placebo: 31 (69.0)
Lee et al,^98^ 2011	Methylphenidate	NR	Control: 157^b^ Intervention: 157^b^	NR	ADHD	NR
Ling et al,^99^ 2014	Methylphenidate	SR	Control: 55 Intervention: 55	90 (81.8)	SUD (amphetamine)	Methylphenidate: 28 AEs Placebo: 40 AEs
Lopez et al,^100^ 2008	Lisdexamfetamine	Oral	Control: 72 Intervention: 218	NR	ADHD	30 mg Lisdexamfetamine: 51 (72.0) 50 mg Lisdexamfetamine: 50 (68.0) 70 mg Lisdexamfetamine: 61 (84.0) Placebo: 34 (47.0)
Martin et al,^63^ 2014	Lisdexamfetamine	Oral	Control: 7 Intervention: 24	25 (80.6)	Schizophrenia	Lisdexamfetamine: 18 (75.0) Placebo: 2 (28.6)
Martin et al,^101^ 2014	Lisdexamfetamine and mixed amphetamine salts	Lisdexamfetamine: oral; mixed amphetamine salts: IR	Control: 18^b^ Intervention: 18^b^	11 (61.1)	ADHD	Lisdexamfetamine: 12 (66.7) Mixed amphetamine salts-IR: 9 (52.9) Placebo: 8 (47.1)
Mattingly et al,^33^ 2020	SHP465 (triple-bead mixed amphetamine salts)	ER	Control: 43 Intervention: 45	56 (63.6)	ADHD	Amphetamine: 11 (24.4) Placebo: 7 (16.3)
McElroy et al,^49^ 2015	Lisdexamfetamine	ER	Control: 63 Intervention: 196	48 (18.5)	BED	Lisdexamfetamine: 166 (84.7) Placebo: 37 (58.7)
Medori et al,^102^ 2008	Methylphenidate	OROS	Control: 96 Intervention: 305	218 (54.4)	ADHD	Methylphenidate: 182 (59.7) Placebo: 41 (42.7)
McCracken et al,^31^ 2003	SLI381 (Adderall XR, amphetamine)	XR	Control: 51^b^ Intervention: 51^b^	44 (86.3)	ADHD	NR
Mooney et al,^103^ 2015	Lisdexamfetamine	Oral	Control: 21 Intervention: 22	35 (75.7)	SUD (cocaine)	NR
Muniz et al,^57^ 2008	Methylphenidate	ER	Control: 84^b^ Control: 84^b^	55 (65.5)	ADHD	Methylphenidate: 40 (12.0) Placebo: 3 (3.6)
Newcorn et al,^104^ 2008	Methylphenidate	OROS	Control: 74 Intervention: 219	211 (71.7)	ADHD	Methylphenidate: 146 (67.0) Placebo: 40 (54.0)
Nuijten et al,^65^ 2016	Dexamfetamine	SR	Control: 35 Intervention: 38	66 (90.4)	SUD (cocaine)	d-Amphetamine: 28 (74.0) Placebo: 16 (46.0)
Patkar et al,^48^ 2006	Methylphenidate	OROS	Control: 30 Intervention: 30	22 (37.0)	MDD (treatment resistant)	Methylphenidate: 19 (63.0) Placebo: 17 (57.0)
Pearson et al,^105^ 2013	Methylphenidate	LA and IR	Control: 24^b^ Intervention: 24^b^	19 (79.1)	ADHD and autism	NR
Pelham et al,^106^ 2001	Methylphenidate	LA and IR	Control: 68^b^ Intervention: 68^b^	60 (89.0)	ADHD	NR
Pelham Jr et al,^107^ 2005	Methylphenidate	MTS	Control: 36^b^ Intervention: 36^b^	33 (91.6)	ADHD	NR
Pliszka et al,^55^ 2017	Methylphenidate	DR and ER	Control: 80 Intervention: 81	113 (70.2)	ADHD	Methylphenidate: 56 (69.1) Placebo: 39 (48.8)
Quinn et al,^108^ 2004	Methylphenidate	Dexmethylphenidate and racemic methylphenidate hydrochloride	Control: 31^b^ Intervention: 31^b^	31 (100)	ADHD	d-Amphetamine: 19 (61.0) d,l-Amphetamine: 12 (39.0) Placebo: 19 (61.0)
Ramtvedt et al,^109^ 2014	Dextroamphetamine and methylphenidate	IR	Control: 34^b^ Intervention: 34^b^	27 (79.4)	ADHD	NR
Retz et al,^110^ 2012	Methylphenidate	ER	Control: 78 Intervention: 84	76 (46.9)	ADHD	Methylphenidate: 55 (65.4) Placebo: 32 (41.0)
Rosenberg et al,^46^ 2013	Methylphenidate	NR	Control: 29 Intervention: 29	23 (38.0)	Dementia (Alzheimer disease)	NR
Rösler et al,^111^ 2009	Methylphenidate	ER	Control: 66 Intervention: 183	124 (50.0)	ADHD	Methylphenidate: 135 (74.0) Placebo: 37 (57.0)
Schulz et al,^112^ 2010	Methylphenidate	LA and XR	Control: 146^b^ Intervention: 146^b^	119 (81.0)	ADHD	Methylphenidate: 44 (30.0) Placebo: 38 (26.0)
Shram et al,^28^ 2022	Serdexmethylphenidate	US	Control: 45^b^ Intervention: 45^b^	33 (73.3)	Not currently dependent; stimulant-experienced participants	120 mg Serdexmethylphenidate: 18 (38.3) 240 mg Serdexmethylphenidate: 22 (45.8) Placebo: NR
Silva et al,^113^ 2005	Methylphenidate	OROS and ER	Control: 53^b^ Intervention: 53^b^	34 (62.9)	ADHD	ER-Methylphenidate: 5 (9.4) OROS-methylphenidate: 11 (20.0) Placebo: 2 (4.7)
Spencer et al,^34^ 2006	Mixed amphetamine salts	XR	Control: 52 Intervention: 226	182 (65.5)	ADHD	NR
Spencer et al,^114^ 2007	Dexmethylphenidate	ER	Control: 53 Intervention: 165	127 (58.2)	ADHD	Methylphenidate: 145 (87.9) Placebo: 36 (67.9)
Spencer et al,^30^ 2008	SPD465 (triple-bead mixed amphetamine salts)	ER	Control: 135 Intervention: 137	136 (50.0)	ADHD	Mixed amphetamine salts: 122 (89.1) Placebo: 86 (63.7)
Stein et al,^115^ 2003	Methylphenidate	OROS	Control: 47^b^ Intervention: 47^b^	33 (70.2)	ADHD	NR
Sugaya et al,^51^ 2022	Methylphenidate	IR	Control: 51 Intervention: 51	85 (84.0)	ADHD	Methylphenidate: 4 (8.0) Placebo: 4 (8.0)
Takahashi et al,^47^ 2014	Methylphenidate	OROS	Control: 141 Intervention: 143	139 (49.7)	ADHD	Methylphenidate: 117 (81.8) Placebo: 76 (53.9)
Weisler et al,^42^ 2017	SHP465 (triple-bead mixed amphetamine salts)	XR	Control: 89 Intervention: 182	150 (55.3)	ADHD	SHP465: 182 (57.1) Placebo: 19 (21.3)
Weiss et al,^116^ 2021	Methylphenidate	ER	Control: 78 Intervention: 297	177 (47.8)	ADHD	Methylphenidate: 158 (52.3) Placebo: 25 (32.1)
Weiss et al,^60^ 2021	Methylphenidate	ER	Control: 74 Intervention: 293	239 (67.2)	ADHD	PRC-062: 154 (52.6) Placebo: 24 (32.4)
Wigal et al,^117^ 2004	Methylphenidate	Dexmethylphenidate and racemic methylphenidate hydrochloride	Control: 42 Intervention: 90	116 (87.8)	ADHD	NR
Wigal et al,^118^ 2006	Methylphenidate	NR	Control: 160^b^ Intervention: 160^b^	NR	ADHD	NR
Wigal et al,^43^ 2010	Lisdexamfetamine	NA	Control: 117^b^ Intervention: 115^b^	102 (62.0)	ADHD	Lisdexamfetamine: 32 (27.8) Placebo: 42 (35.9)
Wilens et al,^121^ 2005	Mixed amphetamine salts	XR	Control: 233 Intervention: 54	186 (65)	ADHD	NR
Wilens et al,^61^ 2006	Methylphenidate	OROS	Control: 90 Intervention: 87	142 (80.2)	ADHD	Methylphenidate: 15 (17.2) Placebo: 14 (15.5)
Winhusen et al,^59^ 2011	Methylphenidate	OROS	Control: 152 Intervention: 151	239 (78.9)	ADHD and SUD	Methylphenidate: 111 (73.5) Placebo: 98 (64.5)
Wolraich et al,^52^ 2001	Methylphenidate	IR and OROS	Control: 90 Intervention: 192 (97, IR; 95, OROS)	233 (82.6)	ADHD	OROS-methylphenidate: 40 (42.1) IR-methylphenidate: 45 (46.3) Placebo: 31 (34.4)
Zheng et al,^119^ 2025	Methylphenidate	MR	Control: 112 Intervention: 110	188 (85.0)	ADHD	Methylphenidate: 74 (67.3) Placebo: 55 (49.1)

Abbreviations: ADHD, attention-deficit/hyperactivity disorder; AE, adverse event; BED, binge-eating disorder; DR, delayed release; EFI, executive function impairment; ER or XR, extended release; IR, immediate release; LA, long acting; MDD, major depressive disorder; MLR, multilayer release; MR, modified release; MTS, methylphenidate transdermal system; NA, not applicable; NR, not reported; OROS, osmotic-release oral system; SR, sustained release; SUD, substance use disorder.

^a^ Adverse events were treatment-emergent AEs, if specified. In cases where percent was reported per dose, values were reported for the highest dose.

^b^ Crossover trial (all participants received placebo and intervention at different moments). A total of 16382 participants were included in the systematic review overall.

Most included studies investigated both the effectiveness and AEs of stimulants (69 trials [74.2%]^30,31,32,33,34,35,36,37,38,39,40,41,42,43,44,45,46,47,48,49,50,51,52,53,54,55,56,59,60,61,62,63,64,67,69,70,72,73,76,79,82,83,84,85,87,90,91,92,93,94,95,96,97,98,99,102,103,110,111,113,114,115,116,117,119,120,121,122^) in male patients (67.4% [n = 11 034] of the overall population) with ADHD alone (77 trials [82.7%]^29,30,31,32,33,34,35,36,37,38,39,40,41,42,43,44,45,47,50,51,52,53,54,55,56,57,58,60,61,62,69,70,73,74,76,77,78,79,80,81,82,83,84,85,86,87,88,89,90,91,92,93,94,95,97,98,100,101,102,104,106,107,108,109,110,111,112,113,114,115,116,117,118,119,120,121,122^). Additionally, fixed dosing regimens of stimulants (ie, all participants receiving the same predetermined doses within each study) were used in 86 RCTs (92.4%).^29,30,31,32,33,34,35,36,37,38,39,40,41,42,44,46,49,50,51,52,53,54,55,56,57,58,60,61,62,63,64,65,66,67,68,69,70,72,73,74,76,77,78,79,80,81,82,84,85,86,87,88,89,90,91,92,93,94,95,96,97,98,99,100,101,102,103,104,105,106,107,108,109,110,111,112,113,114,115,116,117,118,119,120,121,122^ Among the stimulants, methylphenidate was used in 60 trials (64.5%)^28,29,44,45,46,47,48,50,51,52,53,54,55,56,57,58,59,60,61,62,64,70,74,77,78,80,82,83,85,87,88,89,90,91,92,93,94,95,96,97,98,99,102,104,105,106,107,108,109,110,111,112,113,114,115,116,117,118,119,122^, of which 20 (23.6%)^45,47,48,52,58,59,61,62,70,74,80,85,87,88,89,91,96,102,104,113,115,121,122^ had the osmotic-release oral system (OROS) formulation. Regarding study duration, 21 RCTs (22.5%)^33,34,36,39,42,48,52,60,69,73,76,77,88,93,100,105,115,116,117,120^ had a 4-week duration, 13 (13.9%)^35,43,46,79,80,81,84,91,97,104,109,113,122^ had a 6-week duration, and other trials spanned variable periods^28^ up to 52 weeks.^29^ Therefore, the results were limited to AEs occurring within approximately 1 year after stimulant use. None of the included trials reported growth assessments or growth delays as noted AEs.

### Results of Meta-Analysis

The meta-analysis showed that stimulants were associated with increased risk of developing overall AEs compared with placebo (RR, 1.34; 90% CI, 1.27-1.41), with high heterogeneity (*I*^2^ = 67%). The significance was maintained when subgroups (ie, methylphenidate, lisdexamfetamine, and other amphetamines) were separately analyzed (eFigure 1 in Supplement 1). Visual inspection of the funnel plot did not show asymmetry, which indicated that publication bias was unlikely and was corroborated by the fail-safe N (5566; *P* < .001) and the rank correlation (0.142; *P* = .10) tests (eFigure 9 in Supplement 1). In addition, the RR for all specific AEs analyzed were higher in the stimulant groups than in the placebo groups (eFigures 2-8 in Supplement 1), as follows: decreased appetite (RR, 3.24; 90% CI, 2.75-3.82), headache (RR, 1.23; 90% CI, 1.14-1.32), insomnia (RR, 2.10; 90% CI, 1.91-2.32), dry mouth (RR, 3.34; 90% CI, 2.64-4.24), nausea (RR, 2.01; 90% CI, 1.69-2.38), irritability (RR, 1.15; 90% CI, 1.06-1.26), and anxiety (RR, 1.23; 90% CI, 1.08-1.41). Regarding vital signs, the SBP was not significantly affected by stimulants (MD, −0.17; 90% CI, −0.43 to 0.09), while the DBP (MD, 1.32; 90% CI, 0.63-2.02) and heart rate (MD, 3.66; 90% CI, 2.94-4.38) were higher in the stimulant groups (Figure 2, Figure 3, and Figure 4). However, none of these differences were deemed clinically significant.

Sensitivity analyses for all forest plots did not detect individual trials affecting the overall results. All forest plots of specific AEs, including those with diverse subgroups (ie, stimulant dose, duration of use, and participant age), are provided in eFigures 2 to 8 and 14 to 144 in Supplement 1 as well as in the summary of commonly mentioned AEs (eFigure 145 in Supplement 1). The bayesian analysis supported a causal association between stimulant use and increased likelihood of overall AEs as well as alterations in vital signs (eTables 4-7 and eFigures 10-13 in Supplement 1).

### Stimulant Safety in ADHD

#### Amphetamines 

Amphetamine was studied in various formulations, including extended-release oral suspension (EROS), extended release (XR or ER), mixed amphetamine salts, manipulation-resistant tablets, racemic amphetamine sulfate, dextroamphetamine transdermal system, and lisdexamfetamine. Studies showed that most participants were male, ranging from 50.0%^30^ to 86.3%^31^ of participants.

Among children and adolescents with ADHD using amphetamine, no serious TEAEs leading to discontinuation were reported. Serious TEAEs are defined by the US Food and Drug Administration (FDA) as those involving death, life-threatening conditions, or hospitalization or causing permanent damage.^123,124^ In RCTs of amphetamine EROS, most TEAEs were mild or moderate and aligned with known safety profiles, such as decreased appetite, insomnia, and headaches.^32^ Other amphetamine salt formulations, such as mixed amphetamine salts,^33,34,35,36^ racemic amphetamine sulfate,^37^ and dextroamphetamine transdermal system,^38^ were similarly well tolerated, with most TEAEs being mild to moderate. For lisdexamfetamine, 7 RCTs^35,39,76,84,85,86,100^ in children and adolescents showed consistent safety profiles, with the most common TEAEs being upper respiratory infections, decreased appetite, headache, weight loss, irritability, insomnia, and dry mouth.^39^

In adults with ADHD, amphetamine-XR trials revealed no changes in the expected safety profile. Common TEAEs included insomnia, dry mouth, and irritability, with no serious AEs.^40,41^ Studies of mixed amphetamine salts-XR reported similar findings, with mild to moderate TEAEs, such as decreased appetite, dry mouth, insomnia, and anxiety. However, 6 participants experienced severe AEs, such as migraines, hallucinations, and muscle spasms.^42^ Lisdexamfetamine trials in adults also showed that TEAEs, such as increased pulse, mostly occurred within the first week and subsequently decreased.^43,44^

#### Methylphenidate 

Fifty-five RCTs^29,44,45,47,50,51,52,53,54,55,56,57,58,59,60,61,62,70,74,77,78,80,82,83,85,87,88,89,90,91,92,93,94,95,96,97,98,102,104,105,106,107,108,109,110,111,112,113,114,115,116,117,118,119,122^ evaluated the safety and effectiveness of methylphenidate in patients with ADHD. Most participants were male, although 6 trials included more females.^45,46,47,48,49,50^

Thirty trials^51,52,53,54,55,56,57,58,74,78,82,83,85,87,88,92,93,98,104,105,106,107,108,109,112,113,115,117,118,119^ assessed the safety of methylphenidate in children (aged 3-18 years) with ADHD, with sample sizes ranging from 23 to 332 participants and doses from 5 to 100 mg daily.^51,52,53,54,55,56,57,58,59^ Most of the 30 trials reported mild or moderate TEAEs, such as decreased appetite, headache, insomnia, and abdominal pain. Methylphenidate-XR formulations were associated with weight loss compared with other formulations. Some RCTs also noted increases in blood pressure and pulse rate.^51,54,55,56,57^

Three trials^59,60,61^ focusing on adolescents showed that methylphenidate was generally safe. One trial^96^ of ADHD and substance use disorders (SUDs) reported no significant differences in AEs between methylphenidate and placebo groups.

In adults, 27 trials^28,29,44,45,47,48,50,58,62,64,70,77,80,89,90,91,94,95,96,97,99,102,110,111,114,116,122^ of methylphenidate, including XR and OROS formulations, found the stimulant to be safe, with no reports of serious cardiovascular events.^62^ Modest increases in blood pressure and pulse were observed in some trials, but there were no severe TEAEs.^62^

### Stimulant Safety in Other Mental Disorders

Four RCTs^48,49,63,64^ explored stimulant use in mental disorders other than ADHD. These disorders included BED, schizophrenia, acute mania, and treatment-resistant depression.

An 11-week RCT^49^ of lisdexamfetamine in BED showed that 84.7% of participants (166 of 196) experienced TEAEs compared with 58.7% of participants (37 of 63) in the placebo group. Serious AEs were reported in 1.5% of participants (3 of 196) receiving lisdexamfetamine, and 3.1% of participants (6 of 196) discontinued treatment due to TEAEs.^49^ Among adults with stable schizophrenia on antipsychotic treatment, lisdexamfetamine was well-tolerated, with no serious AEs reported. While pulse increases were dose dependent, blood pressure remained stable.^63^ A short-term trial evaluating methylphenidate for acute mania found that the drug was well-tolerated, with a 4.5% (1 of 22 participants) dropout rate due to AEs and no AEs occurring at a frequency higher than 10%.^48,64^ A trial of OROS-methylphenidate in treatment-resistant depression reported similar AE rates between intervention and control groups (19 of 30 [63.0%] vs 17 of 30 [57.0%]) without significant cardiovascular changes.^48^ Methylphenidate has also been tested in adults with Alzheimer disease. A 6-week trial found methylphenidate to be well-tolerated, with no serious TEAEs other than 1 case of abdominal pain. Participant dropout due to AEs was 10.0% (6 of 60 participants).^46^

In terms of emergence of psychosis, 3 RCTs reported occurrences in the intervention groups: 1 in an adult with ADHD taking OROS-methylphenidate (dose not reported),^47^ 1 in a patient with cocaine dependence receiving heroin-assisted treatment and taking dexamfetamine-XR 60 mg/d,^65^ and 2 in patients with previously stable schizophrenia taking lisdexamfetamine (150 and 200 mg/d).^63^

### Stimulant Safety in SUD

Seven trials^59,65,66,67,96,99,103^ on stimulant use in participants with SUDs were reviewed. One trial evaluated lisdexamfetamine’s abuse potential in stimulant use disorder, finding no significant differences from placebo at lower doses (50 mg and 100 mg) on the Drug Rating Questionnaire-Subject Liking Scale, although differences were noted at 150 mg.^66^ Another RCT of dextroamphetamine transdermal system in individuals with methamphetamine dependence found no significant differences in AEs between stimulant and placebo groups, with no reductions in methamphetamine use observed.^67^ Neither study suggested a risk of misuse or psychotic symptoms.

### Stimulant Safety in Healthy Adults

A phase 1 trial involving Japanese and White participants tested single and multiple doses of lisdexamfetamine.^68^ The trial found no significant racial or ethnic differences in safety outcomes between groups. AEs were consistent with amphetamines’ established safety profile.^68^

## Discussion

In this meta-analysis, stimulants showed higher likelihood of AEs compared with placebo. AEs included decreased appetite, headache, insomnia, anxiety, dry mouth, nausea, and irritability. Additionally, the pooled data on cardiovascular AEs showed that DBP and heart rate were statistically higher in the stimulant groups, although without apparent clinical relevance. No difference was detected on SBP. These findings were from studies with low risk of bias and no evidence of publication bias. Although the overall association between stimulants and AEs has been previously documented,^10,125^ the present study advances this understanding by pooling together and meta-analyzing the most common AEs and discussing clinical conditions beyond ADHD (ie, depression, BED, schizophrenia, Alzheimer disease, and stimulant use disorders).^69,70,122^ Understanding these factors may help patients and clinicians anticipate and manage AEs, increase adherence, and avoid discontinuation.

Among the various categories of AEs associated with stimulants, cardiovascular events raised particular concerns for patients and their families.^126^ However, neither our work nor a prior network meta-analysis by Cortese et al^125^ showed evidence substantiating these concerns. In fact, cardiovascular results from both studies, including measures of SBP, DBP, and heart rate, were not clinically significant. We examined a narrower range of medications but considered a broader spectrum of clinical conditions than Cortese et al.^125^

Regarding fatal or serious cardiovascular events, such as myocardial infarction or stroke, the pooled analysis did not identify significant occurrences. Although the pooled analysis did not reveal associations between fatal events and stimulants, this information only comes from controlled interventional settings, including follow-up duration of up to 52 weeks. In contrast, a recently published pharmacovigilance study^127^ providing insights from larger populations in less controlled settings included reports from over 3 million patients over 8 years. It showed that methylphenidate could be associated with hypertension and myocardial infarction, and amphetamine could be associated with ischemic heart disease.^127^ However, the article did not clarify whether the AEs occurred only in patients who had received prescriptions from health care professionals, nor did it provide information on the doses administered. Moreover, as the data came from the FDA Adverse Event Reporting System, causality could not be demonstrated, and the results might not be directly related to stimulants.

Another category of interest was AEs associated with mood and behavioral disturbances. The pooled data showed a direct association between stimulants and irritability, anxiety, and insomnia. Additionally, some of the included studies reported other AEs that individually were more common in stimulant groups that could not be meta-analyzed, such as aggressiveness,^128^ hypervigilance,^28^ and restlessness.^63^ In a publication involving patients with clinically stable schizophrenia, worsening of psychosis was observed in individuals taking lisdexamfetamine (150 and 200 mg/d).^63^ Two other publications also reported psychotic events: one involving an adult with ADHD taking OROS-methylphenidate (although no further details were provided),^47^ and the other involving a patient with cocaine dependence receiving heroin-assisted treatment and taking dexamfetamine (60 mg/d),^65^ suggesting potentially higher risk in people with preexisting psychiatric conditions and higher potency drugs. The recent pharmacovigilance publication also identified potential associations between amphetamine and psychosis (and aggression) but found no associations between methylphenidate and psychosis or aggression.^127^

Regarding misuse, none of the included RCTs directly identified addiction behaviors associated with the investigated medications, consonant with previous studies.^129,130^ In this sense, a recent FDA review concluded that stimulant misuse mostly occurs through drug diversion rather than self-misuse.^123^ However, 1 study using a 3-question survey found that patients taking 150 mg of lisdexamfetamine reported higher feel-drug-effect ratings than those taking a placebo or lower doses of 50 or 100 mg.^66^ This finding suggests that misuse potential may increase with higher doses, underscoring the need for careful consideration of doses, especially in treating conditions that may require higher doses.^131,132^ To mitigate the risk of overprescribing stimulants and ensure safe use, Prescription Drug Monitoring Programs, state-based systems that track prescriptions for controlled substances, may be consulted.^133,134^

Another AE of potential concern includes growth delays in developing children.^128,135^ None of the studies we reviewed reported on this AE. While long-term implications for growth and adulthood are unclear, evidence suggests growth delays may be reversible.^128,135^ In addition, a meta-analysis of observational studies with over 4500 children taking methylphenidate showed that, although growth delays are possible, they may have small effect sizes and minimal clinical significance.^71^

### Limitations

A limitation of this meta-analysis is the high heterogeneity of the included studies, partly attributed to the inclusion of trials with different conditions, as we aimed to explore stimulants’ broader clinical applications. Furthermore, many trials lacked detailed age-specific analyses, particularly for older adults, a population within which stimulant prescriptions are increasing.^1^ Future research should focus on age-stratified analyses, representation of different races and ethnicities, and sex differences. As adult ADHD diagnoses increase, studies with longer follow-up periods assessing stimulant-related AEs are needed. Alongside ongoing clinical research investigating the potential benefits of amphetamines for treating cocaine use disorder, there is a pressing need for larger, more comprehensive RCTs to thoroughly assess the safety and effectiveness of these medications in such contexts.

## Conclusions

This meta-analysis found an increased risk of AEs with stimulant use compared with placebo, without clinically significant cardiovascular outcomes. This pattern holds across sensitivity analyses and subgroups, with low risk of bias across the included studies. While stimulant abuse potential and emergence of psychosis, particularly in individuals with SUD, remain understudied, future research could provide more standardized and consistent assessments of this outcome. These efforts are important and may improve understanding about misuse risk and inform more personalized treatment strategies for ADHD and other psychiatric disorders.

## Notes

Supplement 1. **eTable 1.** Search Strategy (February 2025) **eTable 2.** Studies Excluded in Phase **eTable 3.** Included Studies and Summary of Data **eFigure 1.** Forest Plot Comparing the Mean Changes in Vital Signs, and Risk of Overall Adverse Events, With 95% Confidence Interval **eFigure 2.** Forest Plot Showing the Risk Ratio of Decreased Appetite Between Control and Experimental Groups **eFigure 3.** Forest Plot Showing the Risk Ratio of Headache Between Control and Experimental Groups **eFigure 4.** Forest Plot Showing the Risk Ratio of Insomnia Between Control and Experimental Groups **eFigure 5.** Forest Plot Showing the Risk Ratio of Dry Mouth Between Control and Experimental Groups **eFigure 6.** Forest Plot Showing the Risk Ratio of Nausea Between Control and Experimental Groups **eFigure 7.** Forest Plot Showing the Risk Ratio of Irritability Between Control and Experimental Groups **eFigure 8.** Forest Plot Showing the Risk Ratio of Anxiety Between Control and Experimental Groups **eFigure 9.** Risk of Bias Assessment **eTable 4.** Bayesian Analysis for Overall AEs **eFigure 10.** Posterior Distribution for Overall AEs **eTable 5.** Bayesian Analysis Results for Systolic Blood Pressure **eFigure 11.** Posterior Distribution for Systolic Blood Pressure **eTable 6.** Bayesian Analysis Results for Diastolic Blood Pressure **eFigure 12.** Posterior Distribution for Diastolic Blood Pressure **eTable 7.** Bayesian Analysis Results for Heart Rate **eFigure 13.** Posterior Distribution for Heart Rate **eFigure 14.** Forest Plot Showing the Risk Ratio of All Adverse Events Between Control and Experimental Groups Using Methylphenidate Subdivided by Age **eFigure 15.** Forest Plot Showing the Risk Ratio of Anxiety Between Control and Experimental Groups Using Methylphenidate Subdivided by Age **eFigure 16.** Forest Plot Showing the Risk Ratio of Decreased Appetite Between Control and Experimental Groups Using Methylphenidate Subdivided by Age **eFigure 17.** Forest Plot Showing the Risk Ratio of Dry Mouth Between Control and Experimental Groups Using Methylphenidate Subdivided by Age **eFigure 18.** Forest Plot Showing the Risk Ratio of Headache Between Control and Experimental Groups Using Methylphenidate Subdivided by Age **eFigure 19.** Forest Plot Showing the Risk Ratio of Insomnia Between Control and Experimental Groups Using Methylphenidate Subdivided by Age **eFigure 20.** Forest Plot Showing the Risk Ratio of Irritability Between Control and Experimental Groups Using Methylphenidate Subdivided by Age **eFigure 21.** Forest Plot Showing the Risk Ratio of Nausea Between Control and Experimental Groups Using Methylphenidate Subdivided by Age **eFigure 22.** Forest Plot Showing the Mean Differences in Heart Rate Between Control and Experimental Groups Using Methylphenidate Subdivided by Age **eFigure 23.** Forest Plot Showing the Mean Differences in Diastolic Blood Pressure Between Control and Experimental Groups Using Methylphenidate Subdivided by Age **eFigure 24.** Forest Plot Showing the Mean Differences in Systolic Blood Pressure Between Control and Experimental Groups Using Methylphenidate Subdivided by Age **eFigure 25.** Forest Plot Showing the Risk Ratio of All Adverse Events Between Control and Experimental Groups Using Lisdexamfetamine Subdivided by Age **eFigure 26.** Forest Plot Showing the Risk Ratio of Anxiety Between Control and Experimental Groups Using Lisdexamfetamine Subdivided by Age **eFigure 27.** Forest Plot Showing the Risk Ratio of Decreased Appetite Between Control and Experimental Groups Using Lisdexamfetamine Subdivided by Age **eFigure 28.** Forest Plot Showing the Risk Ratio of Headache Between Control and Experimental Groups Using Lisdexamfetamine Subdivided by Age **eFigure 29.** Forest Plot Showing the Risk Ratio of Insomnia Between Control and Experimental Groups Using Lisdexamfetamine Subdivided by Age **eFigure 30.** Forest Plot Showing the Risk Ratio of Nausea Between Control and Experimental Groups Using Lisdexamfetamine Subdivided by Age **eFigure 31.** Forest Plot Showing the Mean Differences in Heart Rate Between Control and Experimental Groups Using Lisdexamfetamine Subdivided by Age **eFigure 32.** Forest Plot Showing the Mean Differences in Diastolic Blood Pressure Between Control and Experimental Groups Using Lisdexamfetamine Subdivided by Age **eFigure 33.** Forest Plot Showing the Mean Differences in Systolic Blood Pressure Between Control and Experimental Groups Using Lisdexamfetamine Subdivided by Age **eFigure 34.** Forest Plot Showing the Risk Ratio of All Adverse Events Between Control and Experimental Groups Using Amphetamines Subdivided by Age **eFigure 35.** Forest Plot Showing the Risk Ratio of Anxiety Between Control and Experimental Groups Using Amphetamines Subdivided by Age **eFigure 36.** Forest Plot Showing the Risk Ratio of Decreased Appetite Between Control and Experimental Groups Using Amphetamines Subdivided by Age **eFigure 37.** Forest Plot Showing the Risk Ratio of Dry Mouth Between Control and Experimental Groups Using Amphetamines Subdivided by Age **eFigure 38.** Forest Plot Showing the Risk Ratio of Headache Between Control and Experimental Groups Using Amphetamines Subdivided by Age **eFigure 39.** Forest Plot Showing the Risk Ratio of Insomnia Between Control and Experimental Groups Using Amphetamines Subdivided by Age **eFigure 40.** Forest Plot Showing the Risk Ratio of Irritability Between Control and Experimental Groups Using Amphetamines Subdivided by Age **eFigure 41.** Forest Plot Showing the Risk Ratio of Nausea Between Control and Experimental Groups Using Amphetamines Subdivided by Age **eFigure 42.** Forest Plot Showing the Mean Differences in Heart Rate Between Control and Experimental Groups Using Amphetamines Subdivided by Age **eFigure 43.** Forest Plot Showing the Mean Differences in Diastolic Blood Pressure Between Control and Experimental Groups Using Amphetamines Subdivided by Age **eFigure 44.** Forest Plot Showing the Mean Differences in Systolic Blood Pressure Between Control and Experimental Groups Using Amphetamines Subdivided by Age **eFigure 45.** Forest Plot Showing the Risk Ratio of All Adverse Events Between Control and Experimental Groups in Children Using Methylphenidate Subdivided by Stimulant Dosage **eFigure 46.** Forest Plot Showing the Risk Ratio of Anxiety Between Control and Experimental Groups in Children Using Methylphenidate Subdivided by Stimulant Dosage **eFigure 47.** Forest Plot Showing the Risk Ratio of Decreased Appetite Between Control and Experimental Groups in Children Using Methylphenidate Subdivided by Stimulant Dosage **eFigure 48.** Forest Plot Showing the Risk Ratio of Headache Between Control and Experimental Groups in Children Using Methylphenidate Subdivided by Stimulant Dosage **eFigure 49.** Forest Plot Showing the Risk Ratio of Insomnia Between Control and Experimental Groups in Children Using Methylphenidate Subdivided by Stimulant Dosage **eFigure 50.** Forest Plot Showing the Risk Ratio of Irritability Between Control and Experimental Groups in Children Using Methylphenidate Subdivided by Stimulant Dosage **eFigure 51.** Forest Plot Showing the Mean Differences in Heart Rate Between Control and Experimental Groups Using Methylphenidate in Children Subdivided by Stimulant Dosage **eFigure 52.** Forest Plot Showing the Mean Differences in Diastolic Blood Pressure Between Control and Experimental Groups Using Methylphenidate in Children Subdivided by Stimulant Dosage **eFigure 53.** Forest Plot Showing the Mean Differences in Systolic Blood Pressure Between Control and Experimental Groups Using Methylphenidate in Children Subdivided by Stimulant Dosage **eFigure 54.** Forest Plot Showing the Risk Ratio of All Adverse Events Between Control and Experimental Groups in Adults Using Methylphenidate Subdivided by Stimulant Dosage **eFigure 55.** Forest Plot Showing the Risk Ratio of Anxiety Between Control and Experimental Groups in Adults Using Methylphenidate Subdivided by Stimulant Dosage **eFigure 56.** Forest Plot Showing the Risk Ratio of Decreased Appetite Between Control and Experimental Groups in Adults Using Methylphenidate Subdivided by Stimulant Dosage **eFigure 57.** Forest Plot Showing the Risk Ratio of Dry Mouth Between Control and Experimental Groups in Adults Using Methylphenidate Subdivided by Stimulant Dosage **eFigure 58.** Forest Plot Showing the Risk Ratio of Headache Between Control and Experimental Groups in Adults Using Methylphenidate Subdivided by Stimulant Dosage **eFigure 59.** Forest Plot Showing the Risk Ratio of Insomnia Between Control and Experimental Groups in Adults Using Methylphenidate Subdivided by Stimulant Dosage **eFigure 60.** Forest Plot Showing the Risk Ratio of Irritability Between Control and Experimental Groups in Adults Using Methylphenidate Subdivided by Stimulant Dosage **eFigure 61.** Forest Plot Showing the Risk Ratio of Nausea Between Control and Experimental Groups in Adults Using Methylphenidate Subdivided by Stimulant Dosage **eFigure 62.** Forest Plot Showing the Mean Differences in Heart Rate Between Control and Experimental Groups Using Methylphenidate in Adults Subdivided by Stimulant Dosage **eFigure 63.** Forest Plot Showing the Mean Differences in Diastolic Blood Pressure Between Control and Experimental Groups Using Methylphenidate in Adults Subdivided by Stimulant Dosage **eFigure 64.** Forest Plot Showing the Mean Differences in Systolic Blood Pressure Between Control and Experimental Groups Using Methylphenidate in Adults Subdivided by Stimulant Dosage **eFigure 65.** Forest Plot Showing the Risk Ratio of All Adverse Events Between Control and Experimental Groups in Children Using Lisdexamfetamine Subdivided by Stimulant Dosage **eFigure 66.** Forest Plot Showing the Risk Ratio of Decreased Appetite Between Control and Experimental Groups in Children Using Lisdexamfetamine Subdivided by Stimulant Dosage **eFigure 67.** Forest Plot Showing the Risk Ratio of Headache Between Control and Experimental Groups in Children Using Lisdexamfetamine Subdivided by Stimulant Dosage **eFigure 68.** Forest Plot Showing the Mean Differences in Heart Rate Between Control and Experimental Groups Using Lisdexamfetamine in Children Subdivided by Stimulant Dosage **eFigure 69.** Forest Plot Showing the Mean Differences in Diastolic Blood Pressure Between Control and Experimental Groups Using Lisdexamfetamine in Children Subdivided by Stimulant Dosage **eFigure 70.** Forest Plot Showing the Mean Differences in Systolic Blood Pressure Between Control and Experimental Groups Using Lisdexamfetamine in Children Subdivided by Stimulant Dosage **eFigure 71.** Forest Plot Showing the Risk Ratio of All Adverse Events Between Control and Experimental Groups in Adults Using Lisdexamfetamine Subdivided by Stimulant Dosage **eFigure 72.** Forest Plot Showing the Risk Ratio of Anxiety Between Control and Experimental Groups in Adults Using Lisdexamfetamine Subdivided by Stimulant Dosage **eFigure 73.** Forest Plot Showing the Risk Ratio of Decreased Appetite Between Control and Experimental Groups in Adults Using Lisdexamfetamine Subdivided by Stimulant Dosage **eFigure 74.** Forest Plot Showing the Risk Ratio of Dry Mouth Between Control and Experimental Groups in Adults Using Lisdexamfetamine Subdivided by Stimulant Dosage **eFigure 75.** Forest Plot Showing the Risk Ratio of Headache Between Control and Experimental Groups in Adults Using Lisdexamfetamine Subdivided by Stimulant Dosage **eFigure 76.** Forest Plot Showing the Risk Ratio of Insomnia Between Control and Experimental Groups in Adults Using Lisdexamfetamine Subdivided by Stimulant Dosage **eFigure 77.** Forest Plot Showing the Mean Differences in Heart Rate Between Control and Experimental Groups Using Lisdexamfetamine in Adults Subdivided by Stimulant Dosage **eFigure 78.** Forest Plot Showing the Mean Differences in Diastolic Blood Pressure Between Control and Experimental Groups Using Lisdexamfetamine in Adults Subdivided by Stimulant Dosage **eFigure 79.** Forest Plot Showing the Mean Differences in Systolic Blood Pressure Between Control and Experimental Groups Using Lisdexamfetamine in Adults Subdivided by Stimulant Dosage **eFigure 80.** Forest Plot Showing the Risk Ratio of All Adverse Events Between Control and Experimental Groups in Children Using Dexmethylphenidate Subdivided by Stimulant Dosage **eFigure 81.** Forest Plot Showing the Risk Ratio of Anxiety Between Control and Experimental Groups in Children Using Dexmethylphenidate Subdivided by Stimulant Dosage **eFigure 82.** Forest Plot Showing the Risk Ratio of Decreased Appetite Between Control and Experimental Groups in Children Using Dexmethylphenidate Subdivided by Stimulant Dosage **eFigure 83.** Forest Plot Showing the Risk Ratio of Headache Between Control and Experimental Groups in Children Using Dexmethylphenidate Subdivided by Stimulant Dosage **eFigure 84.** Forest Plot Showing the Mean Differences in Heart Rate Between Control and Experimental Groups Using Dexmethylphenidate in Children Subdivided by Stimulant Dosage **eFigure 85.** Forest Plot Showing the Mean Differences in Diastolic Blood Pressure Between Control and Experimental Groups Using Dexmethylphenidate in Children Subdivided by Stimulant Dosage **eFigure 86.** Forest Plot Showing the Mean Differences in Systolic Blood Pressure Between Control and Experimental Groups Using Dexmethylphenidate in Children Subdivided by Stimulant Dosage **eFigure 87.** Forest Plot Showing the Risk Ratio of All Adverse Events Between Control and Experimental Groups in Adults Using Dexmethylphenidate Subdivided by Stimulant Dosage **eFigure 88.** Forest Plot Showing the Risk Ratio of Anxiety Between Control and Experimental Groups in Adults Using Dexmethylphenidate Subdivided by Stimulant Dosage **eFigure 89.** Forest Plot Showing the Risk Ratio of Decreased Appetite Between Control and Experimental Groups in Adults Using Dexmethylphenidate Subdivided by Stimulant Dosage **eFigure 90.** Forest Plot Showing the Risk Ratio of Dry Mouth Between Control and Experimental Groups in Adults Using Dexmethylphenidate Subdivided by Stimulant Dosage **eFigure 91.** Forest Plot Showing the Risk Ratio of Headache Between Control and Experimental Groups in Adults Using Dexmethylphenidate Subdivided by Stimulant Dosage **eFigure 92.** Forest Plot Showing the Risk Ratio of Insomnia Between Control and Experimental Groups in Adults Using Dexmethylphenidate Subdivided by Stimulant Dosage **eFigure 93.** Forest Plot Showing the Mean Differences in Heart Rate Between Control and Experimental Groups Using Dexmethylphenidate in Adults Subdivided by Stimulant Dosage **eFigure 94.** Forest Plot Showing the Mean Differences in Diastolic Blood Pressure Between Control and Experimental Groups Using Dexmethylphenidate in Adults Subdivided by Stimulant Dosage **eFigure 95.** Forest Plot Showing the Mean Differences in Systolic Blood Pressure Between Control and Experimental Groups Using Dexmethylphenidate in Adults Subdivided by Stimulant Dosage **eFigure 96.** Forest Plot Showing the Risk Ratio of All Adverse Events Between Control and Experimental Groups in Children Using Amphetamine Subdivided by Stimulant Dosage **eFigure 97.** Forest Plot Showing the Risk Ratio of Anxiety Between Control and Experimental Groups in Children Using Amphetamine Subdivided by Stimulant Dosage **eFigure 98.** Forest Plot Showing the Risk Ratio of Decreased Appetite Between Control and Experimental Groups in Children Using Amphetamine Subdivided by Stimulant Dosage **eFigure 99.** Forest Plot Showing the Risk Ratio of Headache Between Control and Experimental Groups in Children Using Amphetamine Subdivided by Stimulant Dosage **eFigure 100.** Forest Plot Showing the Risk Ratio of All Adverse Events Between Control and Experimental Groups in Participants Using Medium Dose Methylphenidate Subdivided by Duration of Use **eFigure 101.** Forest Plot Showing the Risk Ratio of Anxiety Between Control and Experimental Groups in Participants Using Medium Dose Methylphenidate Subdivided by Duration of Use **eFigure 102.** Forest Plot Showing the Risk Ratio of Decreased Appetite Between Control and Experimental Groups in Participants Using Medium Dose Methylphenidate Subdivided by Duration of Use **eFigure 103.** Forest Plot Showing the Risk Ratio of Dry Mouth Between Control and Experimental Groups in Participants Using Medium Dose Methylphenidate Subdivided by Duration of Use **eFigure 104.** Forest Plot Showing the Risk Ratio of Headache Between Control and Experimental Groups in Participants Using Medium Dose Methylphenidate Subdivided by Duration of Use **eFigure 105.** Forest Plot Showing the Risk Ratio of Insomnia Between Control and Experimental Groups in Participants Using Medium Dose Methylphenidate Subdivided by Duration of Use **eFigure 106.** Forest Plot Showing the Risk Ratio of Irritability Between Control and Experimental Groups in Participants Using Medium Dose Methylphenidate Subdivided by Duration of Use **eFigure 107.** Forest Plot Showing the Risk Ratio of Nausea Between Control and Experimental Groups in Participants Using Medium Dose Methylphenidate Subdivided by Duration of Use **eFigure 108.** Forest Plot Showing the Mean Differences in Heart Rate Between Control and Experimental Groups in Participants Using Medium Dose Methylphenidate Subdivided by Duration of Use **eFigure 109.** Forest Plot Showing the Mean Differences in Diastolic Blood Pressure Between Control and Experimental Groups in Participants Using Medium Dose Methylphenidate Subdivided by Duration of Use **eFigure 110.** Forest Plot Showing the Mean Differences in Systolic Blood Pressure Between Control and Experimental Groups in Participants Using Medium Dose Methylphenidate Subdivided by Duration of Use **eFigure 111.** Forest Plot Showing the Risk Ratio of All Adverse Events Between Control and Experimental Groups in Participants Using High Dose Methylphenidate Subdivided by Duration of Use **eFigure 112.** Forest Plot Showing the Risk Ratio of Anxiety Between Control and Experimental Groups in Participants Using High Dose Methylphenidate Subdivided by Duration of Use **eFigure 113.** Forest Plot Showing the Risk Ratio of Decreased Appetite Between Control and Experimental Groups in Participants Using High Dose Methylphenidate Subdivided by Duration of Use **eFigure 114.** Forest Plot Showing the Risk Ratio of Dry Mouth Between Control and Experimental Groups in Participants Using High Dose Methylphenidate Subdivided by Duration of Use **eFigure 115.** Forest Plot Showing the Risk Ratio of Headache Between Control and Experimental Groups in Participants Using High Dose Methylphenidate Subdivided by Duration of Use **eFigure 116.** Forest Plot Showing the Risk Ratio of Insomnia Between Control and Experimental Groups in Participants Using High Dose Methylphenidate Subdivided by Duration of Use **eFigure 117.** Forest Plot Showing the Risk Ratio of Irritability Between Control and Experimental Groups in Participants Using High Dose Methylphenidate Subdivided by Duration of Use **eFigure 118.** Forest Plot Showing the Risk Ratio of Nausea Between Control and Experimental Groups in Participants Using High Dose Methylphenidate Subdivided by Duration of Use **eFigure 119.** Forest Plot Showing the Mean Differences in Heart Rate Between Control and Experimental Groups in Participants Using High Dose Methylphenidate Subdivided by Duration of Use **eFigure 120.** Forest Plot Showing the Mean Differences in Diastolic Blood Pressure Between Control and Experimental Groups in Participants Using High Dose Methylphenidate Subdivided by Duration of Use **eFigure 121.** Forest Plot Showing the Mean Differences in Systolic Blood Pressure Between Control and Experimental Groups in Participants Using High Dose Methylphenidate Subdivided by Duration of Use **eFigure 122.** Forest Plot Showing the Risk Ratio of All Adverse Events Between Control and Experimental Groups in Participants Using Very High Dose Methylphenidate Subdivided by Duration of Use **eFigure 123.** Forest Plot Showing the Risk Ratio of Anxiety Between Control and Experimental Groups in Participants Using Very High Dose Methylphenidate Subdivided by Duration of Use **eFigure 124.** Forest Plot Showing the Risk Ratio of Decreased Appetite Between Control and Experimental Groups in Participants Using Very High Dose Methylphenidate Subdivided by Duration of Use **eFigure 125.** Forest Plot Showing the Risk Ratio of Dry Mouth Between Control and Experimental Groups in Participants Using Very High Dose Methylphenidate Subdivided by Duration of Use **eFigure 126.** Forest Plot Showing the Risk Ratio of Headache Between Control and Experimental Groups in Participants Using Very High Dose Methylphenidate Subdivided by Duration of Use **eFigure 127.** Forest Plot Showing the Risk Ratio of Insomnia Between Control and Experimental Groups in Participants Using Very High Dose Methylphenidate Subdivided by Duration of Use **eFigure 128.** Forest Plot Showing the Risk Ratio of Irritability Between Control and Experimental Groups in Participants Using Very High Dose Methylphenidate Subdivided by Duration of Use **eFigure 129.** Forest Plot Showing the Risk Ratio of Nausea Between Control and Experimental Groups in Participants Using Very High Dose Methylphenidate Subdivided by Duration of Use **eFigure 130.** Forest Plot Showing the Mean Differences in Heart Rate Between Control and Experimental Groups in Participants Using Very High Dose Methylphenidate Subdivided by Duration of Use **eFigure 131.** Forest Plot Showing the Mean Differences in Diastolic Blood Pressure Between Control and Experimental Groups in Participants Using Very High Dose Methylphenidate Subdivided by Duration of Use **eFigure 132.** Forest Plot Showing the Mean Differences in Systolic Blood Pressure Between Control and Experimental Groups in Participants Using Very High Dose Methylphenidate Subdivided by Duration of Use **eFigure 133.** Forest Plot Showing the Risk Ratio of All Adverse Events Between Control and Experimental Groups in Participants Using Methylphenidate in Different Forms Subdivided by Duration of Use **eFigure 134.** Forest Plot Showing the Risk Ratio of Anxiety Between Control and Experimental Groups in Participants Using Methylphenidate in Different Forms Subdivided by Duration of Use **eFigure 135.** Forest Plot Showing the Risk Ratio of Decreased Appetite Between Control and Experimental Groups in Participants Using Methylphenidate in Different Forms Subdivided by Duration of Use **eFigure 136.** Forest Plot Showing the Risk Ratio of Dry Mouth Between Control and Experimental Groups in Participants Using Methylphenidate in Different Forms Subdivided by Duration of Use **eFigure 137.** Forest Plot Showing the Risk Ratio of Headache Between Control and Experimental Groups in Participants Using Methylphenidate in Different Forms Subdivided by Duration of Use **eFigure 138.** Forest Plot Showing the Risk Ratio of Insomnia Between Control and Experimental Groups in Participants Using Methylphenidate in Different Forms Subdivided by Duration of Use **eFigure 139.** Forest Plot Showing the Risk Ratio of Irritability Between Control and Experimental Groups in Participants Using Methylphenidate in Different Forms Subdivided by Duration of Use **eFigure 140.** Forest Plot Showing the Risk Ratio of Nausea Between Control and Experimental Groups in Participants Using Methylphenidate in Different Forms Subdivided by Duration of Use **eFigure 141.** Forest Plot Showing the Mean Differences in Heart Rate Between Control and Experimental Groups in Participants Using Methylphenidate in Different Forms Subdivided by Duration of Use **eFigure 142.** Forest Plot Showing the Mean Differences in Diastolic Blood Pressure Between Control and Experimental Groups in Participants Using Methylphenidate in Different Forms Subdivided by Duration of Use **eFigure 143.** Forest Plot Showing the Mean Differences in Systolic Blood Pressure Between Control and Experimental Groups in Participants Using Methylphenidate in Different Forms Subdivided by Duration of Use **eFigure 144.** Forest Plot Showing the Risk Ratio of All Adverse Events Between Control and Experimental Groups in Participants Using Methylphenidate Subdivided by Gender **eFigure 145.** Word Cloud
eReferences

Supplement 2.
Data Sharing Statement
