# Supplementary material for: Safety of Stimulants Across Patient Populations: A Meta-Analysis
Source: JAMA Netw Open. 2025 May 9;8(5):e259492. doi: 10.1001/jamanetworkopen.2025.9492 (PMC12065045; doi:10.1001/jamanetworkopen.2025.9492)
Supplement: Supplement 1. — eTable 1. Search Strategy (February 2025) eTable 2. Studies Excluded in Phase eTable 3. Included Studies and Summary of Data eFigure 1. Forest Plot Comparing the Mean Changes in Vital Signs, and Risk of Overall Adverse Events, With 95% Confidence Interval eFigure 2. Forest Plot Showing the Risk Ratio of Decreased Appetite Between Control and Experimental Groups eFigure 3. Forest Plot Showing the Risk Ratio of Headache Between Control and Experimental Groups eFigure 4. Forest Plot Showing the Risk Ratio of Insomnia Between Control and Experimental Groups eFigure 5. Forest Plot Showing the Risk Ratio of Dry Mouth Between Control and Experimental Groups eFigure 6. Forest Plot Showing the Risk Ratio of Nausea Between Control and Experimental Groups eFigure 7. Forest Plot Showing the Risk Ratio of Irritability Between Control and Experimental Groups eFigure 8. Forest Plot Showing the Risk Ratio of Anxiety Between Control and Experimental Groups eFigure 9. Risk of Bias Assessment eTable 4. Bayesian Analysis for Overall AEs eFigure 10. Posterior Distribution for Overall AEs eTable 5. Bayesian Analysis Results for Systolic Blood Pressure eFigure 11. Posterior Distribution for Systolic Blood Pressure eTable 6. Bayesian Analysis Results for Diastolic Blood Pressure eFigure 12. Posterior Distribution for Diastolic Blood Pressure eTable 7. Bayesian Analysis Results for Heart Rate eFigure 13. Posterior Distribution for Heart Rate eFigure 14. Forest Plot Showing the Risk Ratio of All Adverse Events Between Control and Experimental Groups Using Methylphenidate Subdivided by Age eFigure 15. Forest Plot Showing the Risk Ratio of Anxiety Between Control and Experimental Groups Using Methylphenidate Subdivided by Age eFigure 16. Forest Plot Showing the Risk Ratio of Decreased Appetite Between Control and Experimental Groups Using Methylphenidate Subdivided by Age eFigure 17. Forest Plot Showing the Risk Ratio of Dry Mouth Between Control and Experimental Groups Using Methylphenidate S [file jamanetwopen-e259492-s001.pdf]

Supplemental Online Content

Oliva HNP, Prudente TP, Mayerson TF, et al. Safety of stimulants across patient populations: a meta-analysis. *JAMA Netw Open*. 2025;8(5):e259492. doi:10.1001/jamanetworkopen.2025.9492

eTable 1. Search Strategy (February 2025)

eTable 2. Studies Excluded in Phase

eTable 3. Included Studies and Summary of Data

eFigure 1. Forest Plot Comparing the Mean Changes in Vital Signs, and Risk of Overall Adverse Events, With 95% Confidence Interval

eFigure 2. Forest Plot Showing the Risk Ratio of Decreased Appetite Between Control and Experimental Groups

eFigure 3. Forest Plot Showing the Risk Ratio of Headache Between Control and Experimental Groups

eFigure 4. Forest Plot Showing the Risk Ratio of Insomnia Between Control and Experimental Groups

eFigure 5. Forest Plot Showing the Risk Ratio of Dry Mouth Between Control and Experimental Groups

eFigure 6. Forest Plot Showing the Risk Ratio of Nausea Between Control and Experimental Groups

eFigure 7. Forest Plot Showing the Risk Ratio of Irritability Between Control and Experimental Groups

eFigure 8. Forest Plot Showing the Risk Ratio of Anxiety Between Control and Experimental Groups

eFigure 9. Risk of Bias Assessment

eTable 4. Bayesian Analysis for Overall AEs

eFigure 10. Posterior Distribution for Overall AEs

eTable 5. Bayesian Analysis Results for Systolic Blood Pressure

eFigure 11. Posterior Distribution for Systolic Blood Pressure

eTable 6. Bayesian Analysis Results for Diastolic Blood Pressure

eFigure 12. Posterior Distribution for Diastolic Blood Pressure

eTable 7. Bayesian Analysis Results for Heart Rate

eFigure 13. Posterior Distribution for Heart Rate

eFigure 14. Forest Plot Showing the Risk Ratio of All Adverse Events Between Control and Experimental Groups Using Methylphenidate Subdivided by Age

© 2025 Oliva HNP et al. *JAMA Network Open*

eFigure 15. Forest Plot Showing the Risk Ratio of Anxiety Between Control and Experimental Groups Using Methylphenidate Subdivided by Age

eFigure 16. Forest Plot Showing the Risk Ratio of Decreased Appetite Between Control and Experimental Groups Using Methylphenidate Subdivided by Age

eFigure 17. Forest Plot Showing the Risk Ratio of Dry Mouth Between Control and Experimental Groups Using Methylphenidate Subdivided by Age

eFigure 18. Forest Plot Showing the Risk Ratio of Headache Between Control and Experimental Groups Using Methylphenidate Subdivided by Age

eFigure 19. Forest Plot Showing the Risk Ratio of Insomnia Between Control and Experimental Groups Using Methylphenidate Subdivided by Age

eFigure 20. Forest Plot Showing the Risk Ratio of Irritability Between Control and Experimental Groups Using Methylphenidate Subdivided by Age

eFigure 21. Forest Plot Showing the Risk Ratio of Nausea Between Control and Experimental Groups Using Methylphenidate Subdivided by Age

eFigure 22. Forest Plot Showing the Mean Differences in Heart Rate Between Control and Experimental Groups Using Methylphenidate Subdivided by Age

eFigure 23. Forest Plot Showing the Mean Differences in Diastolic Blood Pressure Between Control and Experimental Groups Using Methylphenidate Subdivided by Age

eFigure 24. Forest Plot Showing the Mean Differences in Systolic Blood Pressure Between Control and Experimental Groups Using Methylphenidate Subdivided by Age

eFigure 25. Forest Plot Showing the Risk Ratio of All Adverse Events Between Control and Experimental Groups Using Lisdexamfetamine Subdivided by Age

eFigure 26. Forest Plot Showing the Risk Ratio of Anxiety Between Control and Experimental Groups Using Lisdexamfetamine Subdivided by Age

eFigure 27. Forest Plot Showing the Risk Ratio of Decreased Appetite Between Control and Experimental Groups Using Lisdexamfetamine Subdivided by Age

eFigure 28. Forest Plot Showing the Risk Ratio of Headache Between Control and Experimental Groups Using Lisdexamfetamine Subdivided by Age

eFigure 29. Forest Plot Showing the Risk Ratio of Insomnia Between Control and Experimental Groups Using Lisdexamfetamine Subdivided by Age

eFigure 30. Forest Plot Showing the Risk Ratio of Nausea Between Control and Experimental Groups Using Lisdexamfetamine Subdivided by Age

eFigure 31. Forest Plot Showing the Mean Differences in Heart Rate Between Control and Experimental Groups Using Lisdexamfetamine Subdivided by Age

eFigure 32. Forest Plot Showing the Mean Differences in Diastolic Blood Pressure Between Control and Experimental Groups Using Lisdexamfetamine Subdivided by Age

© 2025 Oliva HNP et al. *JAMA Network Open*

eFigure 33. Forest Plot Showing the Mean Differences in Systolic Blood Pressure Between Control and Experimental Groups Using Lisdexamfetamine Subdivided by Age

eFigure 34. Forest Plot Showing the Risk Ratio of All Adverse Events Between Control and Experimental Groups Using Amphetamines Subdivided by Age

eFigure 35. Forest Plot Showing the Risk Ratio of Anxiety Between Control and Experimental Groups Using Amphetamines Subdivided by Age

eFigure 36. Forest Plot Showing the Risk Ratio of Decreased Appetite Between Control and Experimental Groups Using Amphetamines Subdivided by Age

eFigure 37. Forest Plot Showing the Risk Ratio of Dry Mouth Between Control and Experimental Groups Using Amphetamines Subdivided by Age

eFigure 38. Forest Plot Showing the Risk Ratio of Headache Between Control and Experimental Groups Using Amphetamines Subdivided by Age

eFigure 39. Forest Plot Showing the Risk Ratio of Insomnia Between Control and Experimental Groups Using Amphetamines Subdivided by Age

eFigure 40. Forest Plot Showing the Risk Ratio of Irritability Between Control and Experimental Groups Using Amphetamines Subdivided by Age

eFigure 41. Forest Plot Showing the Risk Ratio of Nausea Between Control and Experimental Groups Using Amphetamines Subdivided by Age

eFigure 42. Forest Plot Showing the Mean Differences in Heart Rate Between Control and Experimental Groups Using Amphetamines Subdivided by Age

eFigure 43. Forest Plot Showing the Mean Differences in Diastolic Blood Pressure Between Control and Experimental Groups Using Amphetamines Subdivided by Age

eFigure 44. Forest Plot Showing the Mean Differences in Systolic Blood Pressure Between Control and Experimental Groups Using Amphetamines Subdivided by Age

eFigure 45. Forest Plot Showing the Risk Ratio of All Adverse Events Between Control and Experimental Groups in Children Using Methylphenidate Subdivided by Stimulant Dosage

eFigure 46. Forest Plot Showing the Risk Ratio of Anxiety Between Control and Experimental Groups in Children Using Methylphenidate Subdivided by Stimulant Dosage

eFigure 47. Forest Plot Showing the Risk Ratio of Decreased Appetite Between Control and Experimental Groups in Children Using Methylphenidate Subdivided by Stimulant Dosage

eFigure 48. Forest Plot Showing the Risk Ratio of Headache Between Control and Experimental Groups in Children Using Methylphenidate Subdivided by Stimulant Dosage

© 2025 Oliva HNP et al. *JAMA Network Open*

eFigure 49. Forest Plot Showing the Risk Ratio of Insomnia Between Control and Experimental Groups in Children Using Methylphenidate Subdivided by Stimulant Dosage

eFigure 50. Forest Plot Showing the Risk Ratio of Irritability Between Control and Experimental Groups in Children Using Methylphenidate Subdivided by Stimulant Dosage

eFigure 51. Forest Plot Showing the Mean Differences in Heart Rate Between Control and Experimental Groups Using Methylphenidate in Children Subdivided by Stimulant Dosage

eFigure 52. Forest Plot Showing the Mean Differences in Diastolic Blood Pressure Between Control and Experimental Groups Using Methylphenidate in Children Subdivided by Stimulant Dosage

eFigure 53. Forest Plot Showing the Mean Differences in Systolic Blood Pressure Between Control and Experimental Groups Using Methylphenidate in Children Subdivided by Stimulant Dosage

eFigure 54. Forest Plot Showing the Risk Ratio of All Adverse Events Between Control and Experimental Groups in Adults Using Methylphenidate Subdivided by Stimulant Dosage

eFigure 55. Forest Plot Showing the Risk Ratio of Anxiety Between Control and Experimental Groups in Adults Using Methylphenidate Subdivided by Stimulant Dosage

eFigure 56. Forest Plot Showing the Risk Ratio of Decreased Appetite Between Control and Experimental Groups in Adults Using Methylphenidate Subdivided by Stimulant Dosage

eFigure 57. Forest Plot Showing the Risk Ratio of Dry Mouth Between Control and Experimental Groups in Adults Using Methylphenidate Subdivided by Stimulant Dosage

eFigure 58. Forest Plot Showing the Risk Ratio of Headache Between Control and Experimental Groups in Adults Using Methylphenidate Subdivided by Stimulant Dosage

eFigure 59. Forest Plot Showing the Risk Ratio of Insomnia Between Control and Experimental Groups in Adults Using Methylphenidate Subdivided by Stimulant Dosage

eFigure 60. Forest Plot Showing the Risk Ratio of Irritability Between Control and Experimental Groups in Adults Using Methylphenidate Subdivided by Stimulant Dosage

eFigure 61. Forest Plot Showing the Risk Ratio of Nausea Between Control and Experimental Groups in Adults Using Methylphenidate Subdivided by Stimulant Dosage

© 2025 Oliva HNP et al. *JAMA Network Open*

eFigure 62. Forest Plot Showing the Mean Differences in Heart Rate Between Control and Experimental Groups Using Methylphenidate in Adults Subdivided by Stimulant Dosage

eFigure 63. Forest Plot Showing the Mean Differences in Diastolic Blood Pressure Between Control and Experimental Groups Using Methylphenidate in Adults Subdivided by Stimulant Dosage

eFigure 64. Forest Plot Showing the Mean Differences in Systolic Blood Pressure Between Control and Experimental Groups Using Methylphenidate in Adults Subdivided by Stimulant Dosage

eFigure 65. Forest Plot Showing the Risk Ratio of All Adverse Events Between Control and Experimental Groups in Children Using Lisdexamfetamine Subdivided by Stimulant Dosage

eFigure 66. Forest Plot Showing the Risk Ratio of Decreased Appetite Between Control and Experimental Groups in Children Using Lisdexamfetamine Subdivided by Stimulant Dosage

eFigure 67. Forest Plot Showing the Risk Ratio of Headache Between Control and Experimental Groups in Children Using Lisdexamfetamine Subdivided by Stimulant Dosage

eFigure 68. Forest Plot Showing the Mean Differences in Heart Rate Between Control and Experimental Groups Using Lisdexamfetamine in Children Subdivided by Stimulant Dosage

eFigure 69. Forest Plot Showing the Mean Differences in Diastolic Blood Pressure Between Control and Experimental Groups Using Lisdexamfetamine in Children Subdivided by Stimulant Dosage

eFigure 70. Forest Plot Showing the Mean Differences in Systolic Blood Pressure Between Control and Experimental Groups Using Lisdexamfetamine in Children Subdivided by Stimulant Dosage

eFigure 71. Forest Plot Showing the Risk Ratio of All Adverse Events Between Control and Experimental Groups in Adults Using Lisdexamfetamine Subdivided by Stimulant Dosage

eFigure 72. Forest Plot Showing the Risk Ratio of Anxiety Between Control and Experimental Groups in Adults Using Lisdexamfetamine Subdivided by Stimulant Dosage

eFigure 73. Forest Plot Showing the Risk Ratio of Decreased Appetite Between Control and Experimental Groups in Adults Using Lisdexamfetamine Subdivided by Stimulant Dosage

eFigure 74. Forest Plot Showing the Risk Ratio of Dry Mouth Between Control and Experimental Groups in Adults Using Lisdexamfetamine Subdivided by Stimulant Dosage

© 2025 Oliva HNP et al. *JAMA Network Open*

eFigure 75. Forest Plot Showing the Risk Ratio of Headache Between Control and Experimental Groups in Adults Using Lisdexamfetamine Subdivided by Stimulant Dosage

eFigure 76. Forest Plot Showing the Risk Ratio of Insomnia Between Control and Experimental Groups in Adults Using Lisdexamfetamine Subdivided by Stimulant Dosage

eFigure 77. Forest Plot Showing the Mean Differences in Heart Rate Between Control and Experimental Groups Using Lisdexamfetamine in Adults Subdivided by Stimulant Dosage

eFigure 78. Forest Plot Showing the Mean Differences in Diastolic Blood Pressure Between Control and Experimental Groups Using Lisdexamfetamine in Adults Subdivided by Stimulant Dosage

eFigure 79. Forest Plot Showing the Mean Differences in Systolic Blood Pressure Between Control and Experimental Groups Using Lisdexamfetamine in Adults Subdivided by Stimulant Dosage

eFigure 80. Forest Plot Showing the Risk Ratio of All Adverse Events Between Control and Experimental Groups in Children Using Dexmethylphenidate Subdivided by Stimulant Dosage

eFigure 81. Forest Plot Showing the Risk Ratio of Anxiety Between Control and Experimental Groups in Children Using Dexmethylphenidate Subdivided by Stimulant Dosage

eFigure 82. Forest Plot Showing the Risk Ratio of Decreased Appetite Between Control and Experimental Groups in Children Using Dexmethylphenidate Subdivided by Stimulant Dosage

eFigure 83. Forest Plot Showing the Risk Ratio of Headache Between Control and Experimental Groups in Children Using Dexmethylphenidate Subdivided by Stimulant Dosage

eFigure 84. Forest Plot Showing the Mean Differences in Heart Rate Between Control and Experimental Groups Using Dexmethylphenidate in Children Subdivided by Stimulant Dosage

eFigure 85. Forest Plot Showing the Mean Differences in Diastolic Blood Pressure Between Control and Experimental Groups Using Dexmethylphenidate in Children Subdivided by Stimulant Dosage

eFigure 86. Forest Plot Showing the Mean Differences in Systolic Blood Pressure Between Control and Experimental Groups Using Dexmethylphenidate in Children Subdivided by Stimulant Dosage

eFigure 87. Forest Plot Showing the Risk Ratio of All Adverse Events Between Control and Experimental Groups in Adults Using Dexmethylphenidate Subdivided by Stimulant Dosage

© 2025 Oliva HNP et al. *JAMA Network Open*

eFigure 88. Forest Plot Showing the Risk Ratio of Anxiety Between Control and Experimental Groups in Adults Using Dexmethylphenidate Subdivided by Stimulant Dosage

eFigure 89. Forest Plot Showing the Risk Ratio of Decreased Appetite Between Control and Experimental Groups in Adults Using Dexmethylphenidate Subdivided by Stimulant Dosage

eFigure 90. Forest Plot Showing the Risk Ratio of Dry Mouth Between Control and Experimental Groups in Adults Using Dexmethylphenidate Subdivided by Stimulant Dosage

eFigure 91. Forest Plot Showing the Risk Ratio of Headache Between Control and Experimental Groups in Adults Using Dexmethylphenidate Subdivided by Stimulant Dosage

eFigure 92. Forest Plot Showing the Risk Ratio of Insomnia Between Control and Experimental Groups in Adults Using Dexmethylphenidate Subdivided by Stimulant Dosage

eFigure 93. Forest Plot Showing the Mean Differences in Heart Rate Between Control and Experimental Groups Using Dexmethylphenidate in Adults Subdivided by Stimulant Dosage

eFigure 94. Forest Plot Showing the Mean Differences in Diastolic Blood Pressure Between Control and Experimental Groups Using Dexmethylphenidate in Adults Subdivided by Stimulant Dosage

eFigure 95. Forest Plot Showing the Mean Differences in Systolic Blood Pressure Between Control and Experimental Groups Using Dexmethylphenidate in Adults Subdivided by Stimulant Dosage

eFigure 96. Forest Plot Showing the Risk Ratio of All Adverse Events Between Control and Experimental Groups in Children Using Amphetamine Subdivided by Stimulant Dosage

eFigure 97. Forest Plot Showing the Risk Ratio of Anxiety Between Control and Experimental Groups in Children Using Amphetamine Subdivided by Stimulant Dosage

eFigure 98. Forest Plot Showing the Risk Ratio of Decreased Appetite Between Control and Experimental Groups in Children Using Amphetamine Subdivided by Stimulant Dosage

eFigure 99. Forest Plot Showing the Risk Ratio of Headache Between Control and Experimental Groups in Children Using Amphetamine Subdivided by Stimulant Dosage

eFigure 100. Forest Plot Showing the Risk Ratio of All Adverse Events Between Control and Experimental Groups in Participants Using Medium Dose Methylphenidate Subdivided by Duration of Use

© 2025 Oliva HNP et al. *JAMA Network Open*

eFigure 101. Forest Plot Showing the Risk Ratio of Anxiety Between Control and Experimental Groups in Participants Using Medium Dose Methylphenidate Subdivided by Duration of Use

eFigure 102. Forest Plot Showing the Risk Ratio of Decreased Appetite Between Control and Experimental Groups in Participants Using Medium Dose Methylphenidate Subdivided by Duration of Use

eFigure 103. Forest Plot Showing the Risk Ratio of Dry Mouth Between Control and Experimental Groups in Participants Using Medium Dose Methylphenidate Subdivided by Duration of Use

eFigure 104. Forest Plot Showing the Risk Ratio of Headache Between Control and Experimental Groups in Participants Using Medium Dose Methylphenidate Subdivided by Duration of Use

eFigure 105. Forest Plot Showing the Risk Ratio of Insomnia Between Control and Experimental Groups in Participants Using Medium Dose Methylphenidate Subdivided by Duration of Use

eFigure 106. Forest Plot Showing the Risk Ratio of Irritability Between Control and Experimental Groups in Participants Using Medium Dose Methylphenidate Subdivided by Duration of Use

eFigure 107. Forest Plot Showing the Risk Ratio of Nausea Between Control and Experimental Groups in Participants Using Medium Dose Methylphenidate Subdivided by Duration of Use

eFigure 108. Forest Plot Showing the Mean Differences in Heart Rate Between Control and Experimental Groups in Participants Using Medium Dose Methylphenidate Subdivided by Duration of Use

eFigure 109. Forest Plot Showing the Mean Differences in Diastolic Blood Pressure Between Control and Experimental Groups in Participants Using Medium Dose Methylphenidate Subdivided by Duration of Use

eFigure 110. Forest Plot Showing the Mean Differences in Systolic Blood Pressure Between Control and Experimental Groups in Participants Using Medium Dose Methylphenidate Subdivided by Duration of Use

eFigure 111. Forest Plot Showing the Risk Ratio of All Adverse Events Between Control and Experimental Groups in Participants Using High Dose Methylphenidate Subdivided by Duration of Use

eFigure 112. Forest Plot Showing the Risk Ratio of Anxiety Between Control and Experimental Groups in Participants Using High Dose Methylphenidate Subdivided by Duration of Use

eFigure 113. Forest Plot Showing the Risk Ratio of Decreased Appetite Between Control and Experimental Groups in Participants Using High Dose Methylphenidate Subdivided by Duration of Use

© 2025 Oliva HNP et al. *JAMA Network Open*

eFigure 114. Forest Plot Showing the Risk Ratio of Dry Mouth Between Control and Experimental Groups in Participants Using High Dose Methylphenidate Subdivided by Duration of Use

eFigure 115. Forest Plot Showing the Risk Ratio of Headache Between Control and Experimental Groups in Participants Using High Dose Methylphenidate Subdivided by Duration of Use

eFigure 116. Forest Plot Showing the Risk Ratio of Insomnia Between Control and Experimental Groups in Participants Using High Dose Methylphenidate Subdivided by Duration of Use

eFigure 117. Forest Plot Showing the Risk Ratio of Irritability Between Control and Experimental Groups in Participants Using High Dose Methylphenidate Subdivided by Duration of Use

eFigure 118. Forest Plot Showing the Risk Ratio of Nausea Between Control and Experimental Groups in Participants Using High Dose Methylphenidate Subdivided by Duration of Use

eFigure 119. Forest Plot Showing the Mean Differences in Heart Rate Between Control and Experimental Groups in Participants Using High Dose Methylphenidate Subdivided by Duration of Use

eFigure 120. Forest Plot Showing the Mean Differences in Diastolic Blood Pressure Between Control and Experimental Groups in Participants Using High Dose Methylphenidate Subdivided by Duration of Use

eFigure 121. Forest Plot Showing the Mean Differences in Systolic Blood Pressure Between Control and Experimental Groups in Participants Using High Dose Methylphenidate Subdivided by Duration of Use

eFigure 122. Forest Plot Showing the Risk Ratio of All Adverse Events Between Control and Experimental Groups in Participants Using Very High Dose Methylphenidate Subdivided by Duration of Use

eFigure 123. Forest Plot Showing the Risk Ratio of Anxiety Between Control and Experimental Groups in Participants Using Very High Dose Methylphenidate Subdivided by Duration of Use

eFigure 124. Forest Plot Showing the Risk Ratio of Decreased Appetite Between Control and Experimental Groups in Participants Using Very High Dose Methylphenidate Subdivided by Duration of Use

eFigure 125. Forest Plot Showing the Risk Ratio of Dry Mouth Between Control and Experimental Groups in Participants Using Very High Dose Methylphenidate Subdivided by Duration of Use

eFigure 126. Forest Plot Showing the Risk Ratio of Headache Between Control and Experimental Groups in Participants Using Very High Dose Methylphenidate Subdivided by Duration of Use

© 2025 Oliva HNP et al. *JAMA Network Open*

eFigure 127. Forest Plot Showing the Risk Ratio of Insomnia Between Control and Experimental Groups in Participants Using Very High Dose Methylphenidate Subdivided by Duration of Use

eFigure 128. Forest Plot Showing the Risk Ratio of Irritability Between Control and Experimental Groups in Participants Using Very High Dose Methylphenidate Subdivided by Duration of Use

eFigure 129. Forest Plot Showing the Risk Ratio of Nausea Between Control and Experimental Groups in Participants Using Very High Dose Methylphenidate Subdivided by Duration of Use

eFigure 130. Forest Plot Showing the Mean Differences in Heart Rate Between Control and Experimental Groups in Participants Using Very High Dose Methylphenidate Subdivided by Duration of Use

eFigure 131. Forest Plot Showing the Mean Differences in Diastolic Blood Pressure Between Control and Experimental Groups in Participants Using Very High Dose Methylphenidate Subdivided by Duration of Use

eFigure 132. Forest Plot Showing the Mean Differences in Systolic Blood Pressure Between Control and Experimental Groups in Participants Using Very High Dose Methylphenidate Subdivided by Duration of Use

eFigure 133. Forest Plot Showing the Risk Ratio of All Adverse Events Between Control and Experimental Groups in Participants Using Methylphenidate in Different Forms Subdivided by Duration of Use

eFigure 134. Forest Plot Showing the Risk Ratio of Anxiety Between Control and Experimental Groups in Participants Using Methylphenidate in Different Forms Subdivided by Duration of Use

eFigure 135. Forest Plot Showing the Risk Ratio of Decreased Appetite Between Control and Experimental Groups in Participants Using Methylphenidate in Different Forms Subdivided by Duration of Use

eFigure 136. Forest Plot Showing the Risk Ratio of Dry Mouth Between Control and Experimental Groups in Participants Using Methylphenidate in Different Forms Subdivided by Duration of Use

eFigure 137. Forest Plot Showing the Risk Ratio of Headache Between Control and Experimental Groups in Participants Using Methylphenidate in Different Forms Subdivided by Duration of Use

eFigure 138. Forest Plot Showing the Risk Ratio of Insomnia Between Control and Experimental Groups in Participants Using Methylphenidate in Different Forms Subdivided by Duration of Use

eFigure 139. Forest Plot Showing the Risk Ratio of Irritability Between Control and Experimental Groups in Participants Using Methylphenidate in Different Forms Subdivided by Duration of Use

© 2025 Oliva HNP et al. *JAMA Network Open*

eFigure 140. Forest Plot Showing the Risk Ratio of Nausea Between Control and Experimental Groups in Participants Using Methylphenidate in Different Forms Subdivided by Duration of Use

eFigure 141. Forest Plot Showing the Mean Differences in Heart Rate Between Control and Experimental Groups in Participants Using Methylphenidate in Different Forms Subdivided by Duration of Use

eFigure 142. Forest Plot Showing the Mean Differences in Diastolic Blood Pressure Between Control and Experimental Groups in Participants Using Methylphenidate in Different Forms Subdivided by Duration of Use

eFigure 143. Forest Plot Showing the Mean Differences in Systolic Blood Pressure Between Control and Experimental Groups in Participants Using Methylphenidate in Different Forms Subdivided by Duration of Use

eFigure 144. Forest Plot Showing the Risk Ratio of All Adverse Events Between Control and Experimental Groups in Participants Using Methylphenidate Subdivided by Gender

eFigure 145. Word Cloud

eReferences

This supplemental material has been provided by the authors to give readers additional information about their work.

eTable 1. Search strategy (February 2025)

Database	Results
**PubMed**<br>(("safety"[Title/Abstract] OR "adverse event*"[Title/Abstract] OR "side effect*"[Title/Abstract]) AND ("amphetamine"[Title/Abstract] OR "dextroamphetamine"[Title/Abstract] OR "stimulant*"[Title/Abstract] OR "lisdexamfetamine"[Title/Abstract] OR "methylphenidate"[Title/Abstract])) AND ((clinicaltrial[Filter]) AND (fft[Filter]))	**597**
**Web of Science**<br>TI=("safety" OR "adverse event*" OR "side effect*") AND TS=("amphetamine" OR "dextroamphetamine" OR "stimulant*" OR "lisdexamfetamine" OR "methylphenidate")	**372**
**CINAHL/EBSCO**<br>TI ((("safety" OR "adverse event*" OR "side effect*") AND ("amphetamine" OR "dextroamphetamine" OR "stimulant*" OR "lisdexamfetamine" OR "methylphenidate")))	**148**
**EMBASE**<br>('safety':ti,ab OR 'adverse event*':ti,ab OR 'side effect*':ti,ab) AND ('amphetamine':ti,ab OR 'dextroamphetamine':ti,ab OR 'stimulant*':ti,ab OR lisdexamfetamine:ti,ab OR 'methylphenidate':ti,ab) AND [clinical trial]/lim AND [embase]/lim NOT ([embase]/lim AND [medline]/lim)	**305**
**ScienceDirect**<br>Title, abstract, keywords: ("safety" OR "adverse events" OR "side effects") AND ("amphetamine" OR "dextroamphetamine" OR "stimulant" OR "lisdexamfetamine" OR "methylphenidate")	**603**
**Total**	**2,025**

eTable 2. Studies excluded in phase 2.

**Study (year)**	**Link**	**Reason for exclusion**
Adler et al, 2017	https://pubmed.ncbi.nlm.nih.gov/28412886/	No placebo
Barragán et al, 2014	https://pubmed.ncbi.nlm.nih.gov/24464327/	No placebo
Biederman et al, 2005	https://pubmed.ncbi.nlm.nih.gov/16344837/	No placebo
Brown et al, 2011	https://pubmed.ncbi.nlm.nih.gov/21973229/	Insufficient data
Carlson et al, 2007	https://pubmed.ncbi.nlm.nih.gov/17897473/	Concomitant use of other drug(s)
Childress et al, 2017B	https://www.ncbi.nlm.nih.gov/pmc/articles/PMC5326982/	Insufficient data
Childress et al, 2019	https://www.ncbi.nlm.nih.gov/pmc/articles/PMC6362322/	Insufficient data
Childress et al, 2022B	https://pubmed.ncbi.nlm.nih.gov/33892111/	No placebo
Faraone et al, 2023	https://www.ncbi.nlm.nih.gov/pmc/articles/PMC9963474/	Insufficient data
Findling et al, 2009	https://pubmed.ncbi.nlm.nih.gov/19808143/	No placebo
Findling et al, 2013	https://pubmed.ncbi.nlm.nih.gov/23410138/	No placebo
Ginsberg et al, 2011	https://pubmed.ncbi.nlm.nih.gov/21438796/#:~:text=However%2C%20long%2Dterm%20LDX%20treatment,with%20long%2Dacting%20stimulant%20use.	No placebo
Greenhill et al, 2006	https://pubmed.ncbi.nlm.nih.gov/17023867/	Insufficient data
Jasinski et al, 2009B	https://pubmed.ncbi.nlm.nih.gov/18635707/	Insufficient data
Lavretsky et al, 2015	https://www.ncbi.nlm.nih.gov/pmc/articles/PMC4451432/	No placebo
Longo et al, 2010	https://pubmed.ncbi.nlm.nih.gov/19839966/	Insufficient data
Lopez et al, 2003	https://pubmed.ncbi.nlm.nih.gov/12895137/	Insufficient data
Manos et al, 2009	https://pubmed.ncbi.nlm.nih.gov/19849639/	Insufficient data
McGough et al, 2005	https://pubmed.ncbi.nlm.nih.gov/15908835/	No placebo
Mikami et al, 2009	https://pubmed.ncbi.nlm.nih.gov/19418208/#:~:text=No%20interactions%20were%20found%20between,and%20female%20adolescents%20with%20ADHD.	Insufficient data
Murff et al, 2008	https://onlinelibrary.wiley.com/doi/abs/10.1111/j.1530-0277.2008.00689_19.x	Abstract
Murray et al, 2011	https://pubmed.ncbi.nlm.nih.gov/21436147/	Insufficient data
Palumbo et al, 2004	https://pubmed.ncbi.nlm.nih.gov/15319016/	Insufficient data
Parasrampuria et al, 2007	https://pubmed.ncbi.nlm.nih.gov/17962423/	Insufficient data
Riggs et al, 2011	https://www.ncbi.nlm.nih.gov/pmc/articles/PMC3164797/	Insufficient data
Robb et al, 2017	https://pubmed.ncbi.nlm.nih.gov/24874348/#:~:text=Conclusion%3A%20MEROS%20was%20efficacious%20in,other%20extended%2Drelease%20methylphenidate%20pharmacotherapies.	No placebo
Rush et al, 2009	https://pubmed.ncbi.nlm.nih.gov/18926645/	Insufficient data
Rush et al, 2010	https://pubmed.ncbi.nlm.nih.gov/20520288/	Insufficient data
Schein et al, 2024	https://pmc.ncbi.nlm.nih.gov/articles/PMC11363209/	No placebo
Szobot et al, 2008	https://pubmed.ncbi.nlm.nih.gov/18327433/#:~:text=There%20was%20no%20significant%20effect,of%20adolescents%20with%20comorbid%20SUD.	Insufficient data
Trivedi et al, 2013	https://pubmed.ncbi.nlm.nih.gov/24021497/	Concomitant use of other drug(s)
Weisler et al, 2009	https://pubmed.ncbi.nlm.nih.gov/20095369/	No placebo
Wigal et al, 2012	https://pubmed.ncbi.nlm.nih.gov/22372513/	No placebo
Wigal et al, 2017	https://pubmed.ncbi.nlm.nih.gov/28557548/	No placebo
Wilens et al, 2008	https://pubmed.ncbi.nlm.nih.gov/18434918/	Insufficient data
Winhusen et al, 2006	https://pubmed.ncbi.nlm.nih.gov/16916538/	Insufficient data
Zhu et al, 2017	https://e-century.us/files/ijcem/10/6/ijcem0054944.pdf	No placebo

Table legend: Insufficient data: Studies that did not provide enough data to calculate the rate of overall or specific adverse events in comparison with control groups.

eTable 3. Included Studies and Summary of Data

Stimulant Safety Randomized Control Trial (RCT) Summary. Characteristic summaries of all RCTs reviewed in the body of the review are noted here, with notes on stimulant type, drug formulation, daily dosage, trial duration, sample size (N), cohort (i.e., condition population within which the drug was tested), common adverse effects (i.e., treatment emergent adverse events occuring in >10% of the sample, if specified; in cases where % is reported per dosage, values were reported for highest dose), and drop-out rate (i.e., percentage of participants who discontinued treatment after being randomized to the study drug).

Source	Country	Stimulant	Associated condition	Population age	Duration (treatment)	Study design	Explored SUD	Rate of Subjects with AEs, No. (%)
**Adler et al,[1] 2008**	USA	Lisdexamfetamine	ADHD	18-55	4 weeks	RCT	NR	Lisdexamfetamine: 282 (78.7)<br>Placebo: 36 (58)
**Adler et al,[2] 2009**	USA	Methylphenidate	ADHD	18-65	4 weeks	RCT	NR	34 (8.3)
**Adler et al,[3] 2009**	USA	Methylphenidate	ADHD	18-65	7 weeks	RCT	NR	Methylphenidate: 93 (84.5)<br>Placebo: 74 (63.8)
**Adler et al,[4] 2013**	USA	Lisdexamfetamine	ADHD + EFI	18-55	10 weeks	RCT	NR	Lisdexamfetamine: 62 (78.5)<br>Placebo: 47 (58.8)
**Ahmann et al,[5] 2001**	USA	Adderall	ADHD	5-16	4 weeks	COT	NR	NR
**Armstrong et al,[6] 2011**	USA	Methylphenidate	ADHD	9-12	2 weeks	COT	NR	NR
**Biederman et al,[7] 2006**	USA	Methylphenidate	NR	19-60	6 weeks	RCT	NR	NR
**Biederman et al,[8] 2007**	USA	Lisdexamfetamine and mixed amphetamine salts	ADHD	6-12	6 weeks	RCT	NR	Lisdexamfetamine: 8 (16)<br>Mixed amphetamine salts: 9 (18)<br>Placebo: 8 (16)
**Biederman et al,[9] 2007**	USA	Lisdexamfetamine	ADHD	6-12	4 weeks	RCT	NR	Lisdexamfetamine: 162 (74.3)<br>Placebo: 34 (47.2)
**Biederman et al,[10] 2003**	USA and Canada	Methylphenidate	ADHD	6-14	2 weeks	RCT	NR	Methylphenidate: 16 (24.6)<br>Placebo: 17 (23.9)
**Bouffard et al,[11] 2003**	Canada	Methylphenidate	ADHD	17-51	4 weeks	RCT	NR	NR

Source	Country	Stimulant	Associated condition	Population age	Duration (treatment)	Study design	Explored SUD	Rate of Subjects with AEs, No. (%)
**Brams et al,[12] 2008**	USA	Dexmethylphenidate	ADHD	6-12	7 days	COT	NR	Methylphenidate: 15 (17.4) Placebo: 19 (22.1)
**Brams et al,[13] 2011**	USA	Lisdexamfetamine	ADHD	18-55	6 weeks	RCT	NR	NR
**Brams et al,[14] 2018**	USA	SHP465 (Triple-Bead mixed amphetamine salts)	ADHD	6-17	4 weeks	RCT	NR	SHP465: 70 (53) Placebo: 34 (26)
**Bron et al,[15] 2014**	the Netherlands	Methylphenidate	ADHD	18-55	6 weeks	COT	NR	Methylphenidate: 17 (77) Placebo: 10 (46)
**Brown et al,[16] 2010**	USA	Lisdexamfetamine	ADHD	18-55	6 weeks	COT	NR	NR
**Buitelaar et al,[17] 2012**	European countries	Methylphenidate	ADHD	20-62	7 weeks	RCT	NR	Methylphenidate: 7 (30.45) Placebo: 8 (36.4)
**Casas et al,[18] 2013**	European countries	Methylphenidate	ADHD	18-65	13 weeks	RCT	NR	54 mg Methylphenidate: 77 (86.5) 72 mg Methylphenidate: 84 (91.3) Placebo: 76 (78.4)
**Childress et al,[19] 2009**	USA	Dexmethylphenidate	ADHD	6-12	5 weeks	RCT	NR	Methylphenidate: 116 (63.7) Placebo: 36 (57.1)
**Childress et al,[20] 2015**	USA	Evekeo (Racemic Amphetamine Sulfate)	ADHD	6-12	2 weeks	RCT	NR	Evekeo: 10 (10.3) Placebo: 6 (6.2)
**Childress et al,[21] 2017**	USA	Amphetamine	ADHD	6-12	1 week	RCT	NR	Amphetamine: 9 (17.3) Placebo: 6 (12.5)
**Childress et al,[22] 2020**	USA	HLD200 (Delayed-Release and Extended-Release Methylphenidate)	ADHD	6-12	1 week	RCT	NR	Methylphenidate: 24 (36.9) Placebo: 22 (40.7)
**Childress et al,[23] 2020**	USA	PRC-063 (ER-Methylphenidate)	ADHD	6-12	1 week	RCT	NR	Methylphenidate: 18 (24) Placebo: 7 (9.6)
**Childress et al,[24] 2020**	USA	Aptensio XR (Methylphenidate)	ADHD	4-5	2 weeks	RCT	NR	Methylphenidate: 10 (25.6) Placebo: 6 (12)
**Childress et al,[25] 2022**	USA	PRC-063 (ER-Methylphenidate)	ADHD	18-60	1 week	RCT	NR	Methylphenidate: 25 (20.7) Placebo: 18 (15.3)
**Childress et al,[26] 2022**	USA	Lisdexamfetamine	ADHD	4-5	6 weeks	RCT	NR	Lisdexamfetamine: 68 (46.6) Placebo: 19 (42.2)

Source	Country	Stimulant	Associated condition	Population age	Duration (treatment)	Study design	Explored SUD	Rate of Subjects with AEs, No. (%)
**Chronis-Tuscano et al,[27] 2008**	USA	Methylphenidate	ADHD	Mothers: 39.78 ± 5.53; Children: 6-12	7 weeks	RCT	NR	NR
**Coghill et al,[28] 2013**	European countries	Lisdexamfetamine and Methylphenidate	ADHD	6-17	7 weeks	RCT	NR	Lisdexamfetamine: 80 (72.1)<br>Methylphenidate: 72 (64.9)<br>Placebo: 63 (57.3)
**Coghill et al,[29] 2014**	USA and European countries	Lisdexamfetamine	ADHD	6-17	33 weeks	RCT	NR	Lisdexamfetamine: 31 (39.7) Placebo: 20 (25.3)
**Cutler et al,[30] 2022**	USA	Amphetamine	ADHD	18-60	5 weeks	RCT	NR	Amphetamine: 54 (87)<br>Placebo: 35 (54)
**Cutler et al,[31] 2022**	USA	d-ATS (Dextroamphetamine)	ADHD	6-17	2 weeks	COT	NR	d-ATS: 44 (41.9) Placebo: 43 (41)
**Dupaul et al,[32] 2012**	USA	Lisdexamfetamine and Methylphenidate	ADHD	18-23	5 weeks	COT	NR	NR
**Ermer et al,[33] 2019**	USA	Lisdexamfetamine	Healthy	18-55	18 days	RCT	NR	Lisdexamfetamine: 20 (76.9)<br>Placebo: 1 (16.6)
**Faraone et al,[34] 2021**	USA	AR19 (Amphetamine Sulfate)	ADHD	18-55	5 weeks	RCT	NR	NR
**Findling et al,[35] 2008**	USA	Methylphenidate	ADHD	6-12	7 weeks	RCT	NR	MTS: 74 (75.5)<br>OROS: 63 (69.2)<br>Placebo: 49 (57.6)
**Findling et al,[36] 2011**	USA	Lisdexamfetamine	ADHD	13-17	4 weeks	RCT	NR	Lisdexamfetamine: 160 (68.7) Placebo: 45 (58.4)
**Froehlich et al,[37] 2020**	USA	Methylphenidate	ADHD	7-11	4 weeks	COT	NR	NR
**Galloway et al,[38] 2011**	USA	d-ATS (DextroAMPH)	SUD (Metamphetamine dependence)	18-50	8 weeks	RCT	NR	NR
**Ginsberg et al,[39] 2012**	Sweden	Methylphenidate	ADHD	21-61	5 weeks	RCT	Yes	NR
**Ginsberg et al,[40] 2014**	European countries	Methylphenidate	ADHD	18-60	40 weeks	RCT	NR	Methylphenidate: 175 (81)<br>Placebo: 65 (79.3)

Source	Country	Stimulant	Associated condition	Population age	Duration (treatment)	Study design	Explored SUD	Rate of Subjects with AEs, No. (%)
**Goodmanet al,[41] 2017**	USA	Methylphenidate	ADHD	18-65	6 weeks	RCT	NR	Methylphenidate: 126 (72.4)<br>Placebo: 87 (49.7)
**Greenhill et al,[42] 2002**	USA	Methylphenidate	ADHD	6-16	3 weeks	RCT		Methylphenidate: 80 (51.6)<br>Placebo: 61 (37.9)
**Hegerl et al,[43] 2018**	European countries	Methylphenidate	Acute mania	46.21 (mean)	2.5 days	RCT	NR	NR
**Huang et al,[44] 2021**	Taiwan	Methylphenidate	ADHD	6-16	4 weeks	COT	NR	Methylphenidate: 79 (71.8)<br>Placebo: 10 (9.9)
**Huss et al,[45] 2014**	European countries	Methylphenidate	ADHD	18-60	9 weeks	RCT	NR	Methylphenidate: 401 (74)<br>Placebo: 108 (60)
**Jain et al,[46] 2007**	Canada	Methylphenidate	ADHD	18-60	5-11 weeks	RCT	NR	Methylphenidate: 42 (84)<br>Placebo: 29 (58)
**Jasinski et al,[47] 2009**	USA	Lisdexamfetamine	History of SUD (stimulant use)	18-55	6 days (1 day for each treatment/dose)	COT	Yes	Lisdexamfetamine: 15 (41)<br>Placebo: 6 (17)
**Kis et al,[48] 2020**	Germany	Methylphenidate	ADHD	35 (mean)	52 weeks	RCT	NR	Methylphenidate: 197 (96.1)<br>Placebo: 184 (88)
**Konstenius et al,[49] 2014**	Sweden	Methylphenidate	ADHD + SUD (amphetamine)	18-65	24 weeks	RCT	NR	NR
**Kooij et al,[50] 2004**	the Netherlands.	Methylphenidate	ADHD	20-56	6 weeks	COT	NR	Methylphenidate: 37 (82)<br>Placebo: 31 (69)
**Lee et al,[51] 2011**	Canada	Methylphenidate	ADHD	6-12	2 weeks	COT	NR	NR
**Ling et al,[52] 2014**	USA	Methylphenidate	SUD (amphetamine)	18-59	10 weeks	RCT	NR	Methylphenidate: 28 events<br>Placebo: 40 events
**Lopez et al,[53] 2008**	USA	Lisdexamfetamine	ADHD	6-12	4 weeks	RCT	NR	30 mg Lisdexamfetamine: 51 (72)<br>50 mg Lisdexamfetamine: 50 (68)

Source	Country	Stimulant	Associated condition	Population age	Duration (treatment)	Study design	Explored SUD	Rate of Subjects with AEs, No. (%)
								70 mg Lisdexamfetamine: 61 (84)<br>Placebo: 34 (47)
**Martin et al,[54] 2014**	USA	Lisdexamfetamine	Schizophrenia	18-65	30 days	RCT	NR	Lisdexamfetamine: 18 (75)<br>Placebo: 2 (28.6)
**Martin et al,[55] 2014**	USA	Lisdexamfetamine and mixed amphetamine salts	ADHD	18-55	21 days	COT	NR	Lisdexamfetamine: 12 (66.7)<br>Mixed amphetamine salts-IR: 9 (52.9)<br>Placebo: 8 (47.1)
**Mattingly et al,[56] 2020**	USA	SHP465 (Triple-Bead mixed amphetamine salts)	ADHD	6-12	4 weeks	RCT	NR	Amphetamine: 11 (24.4)<br>Placebo: 7 (16.3)
**McElroy et al,[57] 2015**	USA	Lisdexamfetamine	BED	18–55	11 weeks	RCT	NR	Lisdexamfetamine: 166 (84.7)<br>Placebo: 37 (58.7)
**Medori et al,[58] 2008**	Germany	Methylphenidate	ADHD	18-63	5 weeks	RCT	NR	Methylphenidate: 182 (59.7)<br>Placebo: 41 (42.7)
**McCracken et al,[59] 2003**	USA	SLI381 (Adderall XR)	ADHD	6-12	5 weeks	COT	NR	NR
**Mooney et al,[60] 2015**	USA	Lisdexamfetamine	SUD (cocaine)	18-65	14 weeks	RCT	NR	NR
**Muniz et al,[61] 2008**	USA	Methylphenidate	ADHD	6-12	1 day	COT	NR	Methylphenidate: 40 (12)<br>Placebo: 3 (3.6)
**Newcorn et al,[62] 2008**	USA	Methylphenidate	ADHD	6-16	6 weeks	RCT	NR	Methylphenidate: 146 (67)<br>Placebo: 40 (54)
**Nuijten et al,[63] 2016**	the Netherlands	Dextroamphetamine	SUD (cocaine)	>25	12 weeks	RCT	Yes	d-Amphetamine: 28 (74)<br>Placebo: 16 (46)

Source	Country	Stimulant	Associated condition	Population age	Duration (treatment)	Study design	Explored SUD	Rate of Subjects with AEs, No. (%)
**Patkar et al,[64] 2006**	USA	Methylphenidate	MDD (treatment-resistant)	18-65	4 weeks	RCT	NR	Methylphenidate: 19 (63)<br>Placebo: 17 (57)
**Pearson et al,[65] 2013**	USA	Methylphenidate	ADHD + Autism	8.8 (mean)	4 weeks	COT	NR	NR
**Pelham Jr et al,[66] 2001**	USA	Methylphenidate	ADHD	6-12	7 days	COT	NR	NR
**Pelham Jr et al,[67] 2005**	USA	Methylphenidate	ADHD	6-13	8 days	COT	NR	NR
**Pliszka et al,[68] 2017**	USA	Methylphenidate	ADHD	6-12	3 weeks	RCT	NR	Methylphenidate: 56 (69.1)<br>Placebo: 39 (48.8)
**Quinn et al,[69] 2004**	Canada	Methylphenidate	ADHD	9-12	7 days	COT	NR	d-Amphetamine: 19 (61)<br>d,l-Amphetamine: 12 (39)<br>Placebo: 19 (61)
**Ramtvedt et al,[70] 2014**	Norway	DextroAMPH and Methylphenidate	ADHD	9-14	6 weeks	COT	NR	NR
**Retz et al,[71] 2012**	Germany	Methylphenidate	ADHD	>18	8 weeks	RCT	NR	Methylphenidate: 55 (65.4)<br>Placebo: 32 (41)
**Rosenberg et al,[72] 2014**	USA and Canada	Methylphenidate	Dementia (Alzheimer's disease)	76 (mean)	6 weeks	RCT	NR	NR
**Rösler et al,[73] 2009**	Germany	Methylphenidate	ADHD	>18	24 weeks	RCT	NR	Methylphenidate: 135 (74)<br>Placebo: 37 (57)
**Schulz et al,[74] 2010**	Germany	Methylphenidate	ADHD	6-14	3 weeks	COT	NR	Methylphenidate: 44 (30)<br>Placebo: 38 (26)

Source	Country	Stimulant	Associated condition	Population age	Duration (treatment)	Study design	Explored SUD	Rate of Subjects with AEs, No. (%)
**Shram et al,[75] 2022**	USA	Serdexmethylphenidate	Not currently dependent, stimulant experienced subjects	18–50	Single dose	COT	Yes	120 mg: 18 (38.3)<br>240 mg: 22 (45.8)<br>Placebo: NR
**Silva et al,[76] 2005**	USA	Methylphenidate	ADHD	6-12	6 weeks	COT	NR	ER-Methylphenidate: 5 (9.4)<br>OROS-Methylphenidate: 11 (20)<br>Placebo: 2 (4.7)
**Spencer et al,[77] 2006**	USA	Mixed amphetamine salts	ADHD	13-17	4 weeks	RCT	NR	NR
**Spencer et al,[78] 2007**	USA	Dexmethylphenidate	ADHD	18-60	5 weeks	RCT	NR	Methylphenidate: 145 (87.9)<br>Placebo: 36 (67.9)
**Spencer et al,[79] 2008**	USA	SPD465 (Triple-Bead mixed amphetamine salts)	ADHD	18-55	7 weeks	RCT	NR	Mixed amphetamine salts: 122 (89.1)<br>Placebo: 86 (63.7)
**Stein et al,[80] 2003**	USA	Methylphenidate	ADHD	5-16	4 weeks	COT	NR	NR
**Sugaya et al,[81] 2022**	Brazil	Methylphenidate	ADHD	3-5	8 weeks	RCT	NR	Methylphenidate: 4 (8)<br>Placebo: 4 (8)
**Takahashi et al,[82] 2014**	Japan	Methylphenidate	ADHD	18-64	8 weeks	RCT	Yes	Methylphenidate: 117 (81.8)<br>Placebo: 76 (53.9)
**Weisler et al,[83] 2017**	USA	SHP465 (Triple-Bead mixed amphetamine salts)	ADHD	18–55	4 weeks	RCT	NR	SHP465: 182 (57.1)<br>Placebo: 19 (21.3)
**Weiss et al,[84] 2020**	USA	Methylphenidate	ADHD	>18	4 weeks	RCT	NR	Methylphenidate: 158 (52.3)<br>Placebo: 25 (32.1)
**Weiss et al,[85] 2021**	USA	Methylphenidate	ADHD	12-17	4 weeks	RCT	NR	PRC-062: 154 (52.6)<br>Placebo: 24 (32.4)
**Wigal et al,[86] 2004**	USA	Methylphenidate	ADHD	6-17	4 weeks	RCT	NR	NR
**Wigal et al,[87] 2006**	USA	Methylphenidate	ADHD	3-5	5 weeks	COT	NR	NR

Source	Country	Stimulant	Associated condition	Population age	Duration (treatment)	Study design	Explored SUD	Rate of Subjects with AEs, No. (%)
**Wigal et al,[88] 2010**	USA	Lisdexamfetamine	ADHD	18–55	6 weeks	COT	NR	Lisdexamfetamine: 32 (27.8)<br>Placebo: 42 (35.9)
**Wilens et al,[89] 2005**	USA	Mixed amphetamine salts	ADHD	13-17	4 weeks	RCT	NR	NR
**Wilens et al,[90] 2006**	USA	Methylphenidate	ADHD	13-18	2 weeks	RCT	NR	Methylphenidate: 15 (17.2)<br>Placebo: 14 (15.5)
**Winhusen et al,[91] 2011**	USA	Methylphenidate	ADHD + SUD	13-18	16 weeks	RCT	Yes	Methylphenidate: 111 (73.5)<br>Placebo: 98 (64.5)
**Wolraich et al,[92] 2001**	USA	Methylphenidate	ADHD	6-12	4 weeks	RCT	NR	OROS Methylphenidate: 40 (42.1)<br>IR Methylphenidate: 45 (46.3)<br>Placebo: 31 (34.4)
**Zheng et al,[93] 2024**	USA	Methylphenidate	ADHD	6-16	9 weeks	RCT	NR	Methylphenidate: 74 (67.3)<br>Placebo: 55 (49.1)

Table legend: **Stimulant Type:** d-ATS, dextroamphetamine transdermal system; MTS, Methylphenidate transdermal system. **Drug Formulation:** EROS, extended-release oral suspension; ER or XR, extended-release; IR, immediate-release; LA, long-acting; MR, modified release; OROS, osmotic-release oral system; SR, sustained release. **Cohort:** ADHD; attention-deficit/hyperactivity; BED, binge eating disorder; EFI, executive function impairment; MDD, Major Depressive Disorder; NA, not applicable; NR, not reported; SUD, non-prescribed substance use disorder. COT: Crossover trial. RCT: Randomized controlled trial. **SUD**: substance use disorder.

eFigure 1. Forest plot comparing the mean changes in vital signs, and risk of overall adverse events, with 95% confidence interval (A-D), and overall adverse events with 90% confidence interval (E).

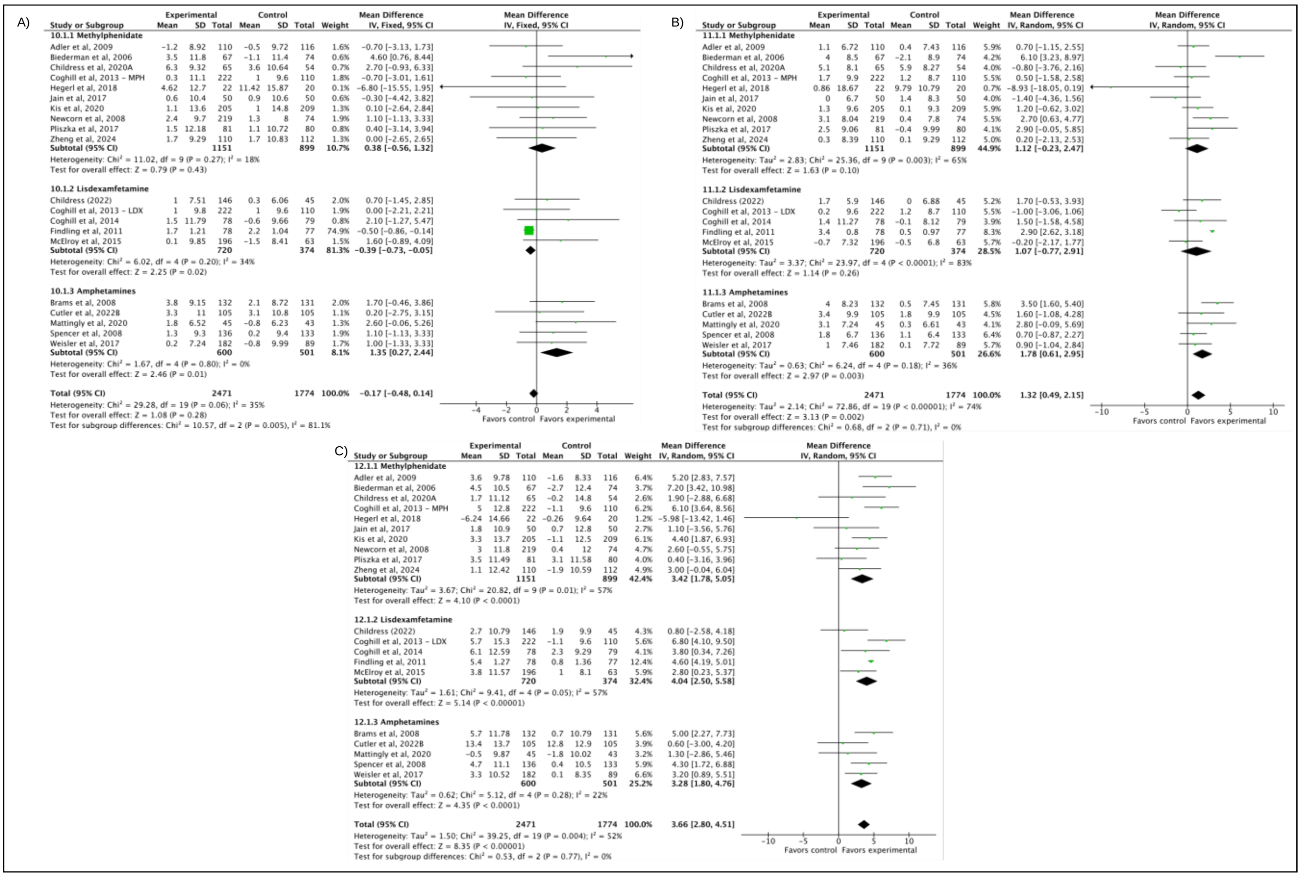

eFigure 1A-C. Forest plot comparing the mean changes in vital signs in patients receiving stimulants vs placebo. A) Systolic blood pressure, B) Diastolic blood pressure, C) Heart rate. Figure legend: 95% CI, 95% confidence interval; IV, inverse-variance; LDX, lisdexamfetamine; MPH, methylphenidate; SD, standard deviation.

D)

Study or Subgroup	Experimental Events	Experimental Total	Control Events	Control Total	Weight	Risk Ratio M-H, Random, 95% CI
**1.1.1 Methylphenidate**						
Adler et al, 2009	93	110	74	116	2.8%	1.33 [1.13, 1.55]
Brams et al, 2008	15	86	19	86	0.8%	0.79 [0.43, 1.45]
Bron et al, 2014	17	22	10	22	1.0%	1.70 [1.02, 2.83]
Buitelaar et al, 2012	7	23	8	22	0.5%	0.84 [0.37, 1.92]
Casas et al, 2013	161	181	76	97	3.0%	1.14 [1.01, 1.28]
Childress (2022)	25	121	18	118	0.9%	1.35 [0.78, 2.35]
Childress et al, 2009	116	182	36	63	2.2%	1.12 [0.88, 1.42]
Childress et al, 2020A	24	65	22	54	1.2%	0.91 [0.58, 1.42]
Childress et al, 2020B	18	75	7	73	0.5%	2.50 [1.11, 5.63]
Childress et al, 2020C	10	39	6	50	0.4%	2.14 [0.85, 5.37]
Coghill et al, 2013 - MPH	72	111	63	110	2.4%	1.13 [0.92, 1.40]
Findling et al, 2008 - MPH	74	98	49	85	2.4%	1.31 [1.06, 1.62]
Findling et al, 2008 - MTS	63	91	49	85	2.3%	1.20 [0.96, 1.51]
Ginsberg et al, 2014	175	216	65	82	2.9%	1.02 [0.90, 1.16]
Goodman et al, 2017	126	174	87	175	2.7%	1.46 [1.22, 1.74]
Greenhill et al, 2002	80	155	61	161	2.2%	1.36 [1.06, 1.75]
Huang et al, 2021	79	110	10	101	0.8%	7.25 [3.98, 13.21]
Huss et al, 2014	401	542	108	180	2.9%	1.23 [1.08, 1.40]
Jain et al, 2017	42	50	29	50	2.1%	1.45 [1.11, 1.89]
Kis et al, 2020	197	205	184	209	3.3%	1.09 [1.03, 1.16]
Kooji et al, 2004	37	45	31	45	2.2%	1.19 [0.94, 1.52]
Medori et al, 2008	182	305	41	96	2.2%	1.40 [1.09, 1.79]
Muniz et al, 2008	40	332	0	83	0.0%	20.43 [1.27, 328.88]
Newcorn et al, 2008	146	219	40	74	2.3%	1.23 [0.98, 1.55]
Patkar et al, 2006	19	30	17	30	1.3%	1.12 [0.74, 1.69]
Pliszka et al, 2017	56	81	39	80	2.1%	1.42 [1.09, 1.85]
Retz et al, 2012	55	84	32	78	1.8%	1.60 [1.17, 2.17]
Rösler et al, 2009	135	183	37	66	2.3%	1.32 [1.05, 1.66]
Schulz et al, 2010	44	146	38	146	1.5%	1.16 [0.80, 1.67]
Silva et al, 2005 - ER	5	53	2	53	0.1%	2.50 [0.51, 12.32]
Silva et al, 2005 - OROS	11	53	2	53	0.2%	5.50 [1.28, 23.63]
Spencer et al, 2007	145	165	36	53	2.5%	1.29 [1.07, 1.57]
Sugaya et al, 2022	4	51	4	51	0.2%	1.00 [0.26, 3.78]
Takahashi et al, 2014	117	143	76	141	2.7%	1.52 [1.28, 1.80]
Weiss et al, 2020	158	297	25	78	1.7%	1.66 [1.18, 2.33]
Weiss et al, 2021	154	293	24	74	1.6%	1.62 [1.15, 2.29]
Wilens et al, 2006	15	87	14	90	0.7%	1.11 [0.57, 2.16]
Winhusen et al, 2011	111	151	98	152	2.8%	1.14 [0.98, 1.33]
Wolraich et al, 2001 - IR	40	95	31	90	1.5%	1.22 [0.84, 1.77]
Wolraich et al, 2001 - OROS	45	97	31	90	1.6%	1.35 [0.94, 1.92]
Zheng et al, 2024	74	110	55	112	2.3%	1.37 [1.09, 1.72]
**Subtotal (95% CI)**		**5676**		**3674**	**71.3%**	**1.31 [1.22, 1.40]**
Total events	3388		1654			
Heterogeneity: $Tau^2 = 0.03$; $Chi^2 = 131.63$, df = 40 (P < 0.00001); $I^2 = 70\%$						
Test for overall effect: Z = 7.28 (P < 0.00001)						
**1.1.2 Lisdexamfetamine dimesylate**						
Adler et al, 2013	62	79	47	80	2.4%	1.34 [1.08, 1.66]
Childress (2022)	68	146	19	45	1.5%	1.10 [0.75, 1.62]
Coghill et al, 2013 - LDX	80	111	63	110	2.5%	1.26 [1.03, 1.53]
Coghill et al, 2014	31	78	20	79	1.2%	1.57 [0.98, 2.50]
Ermer et al, 2019	20	26	1	6	0.1%	4.62 [0.76, 27.96]
Findling et al, 2011	160	232	45	77	2.5%	1.18 [0.96, 1.45]
Jasinski et al, 2009	15	37	6	36	0.5%	2.43 [1.06, 5.57]
Martin et al, 2014A	18	24	2	7	0.3%	2.63 [0.80, 8.66]
Martin et al, 2014B - LDX	12	18	8	17	0.8%	1.42 [0.78, 2.58]
McElroy et al, 2015	166	196	37	63	2.4%	1.44 [1.16, 1.79]
Wigal et al, 2010 - LDX	32	115	42	117	1.5%	0.78 [0.53, 1.13]
**Subtotal (95% CI)**		**1062**		**637**	**15.5%**	**1.28 [1.12, 1.46]**
Total events	664		290			
Heterogeneity: $Tau^2 = 0.02$; $Chi^2 = 15.67$, df = 10 (P = 0.11); $I^2 = 36\%$						
Test for overall effect: Z = 3.67 (P = 0.0002)						
**1.1.3 Amphetamines**						
Brams et al, 2018	70	131	34	131	1.7%	2.06 [1.48, 2.87]
Childress et al, 2015	10	97	6	97	0.4%	1.67 [0.63, 4.41]
Childress et al, 2017	9	51	6	48	0.4%	1.41 [0.54, 3.67]
Cutler et al, 2022A	54	62	35	65	2.2%	1.62 [1.27, 2.07]
Cutler et al, 2022B	44	105	43	105	1.8%	1.02 [0.74, 1.41]
Martin et al, 2014B - AMPH	9	17	8	17	0.7%	1.13 [0.57, 2.21]
Mattingly et al, 2020	11	45	7	43	0.5%	1.50 [0.64, 3.51]
Nujiten et al, 2016	28	38	16	35	1.4%	1.61 [1.07, 2.42]
Spencer et al, 2008	122	137	86	135	2.9%	1.40 [1.22, 1.61]
Weisler et al, 2017	104	182	19	89	1.3%	2.68 [1.76, 4.07]
**Subtotal (95% CI)**		**865**		**765**	**13.2%**	**1.56 [1.30, 1.88]**
Total events	461		260			
Heterogeneity: $Tau^2 = 0.04$; $Chi^2 = 19.77$, df = 9 (P = 0.02); $I^2 = 54\%$						
Test for overall effect: Z = 4.79 (P < 0.00001)						
**Total (95% CI)**		**7603**		**5076**	**100.0%**	**1.34 [1.26, 1.42]**
Total events	4513		2204			
Heterogeneity: $Tau^2 = 0.03$; $Chi^2 = 185.38$, df = 61 (P < 0.00001); $I^2 = 67\%$						
Test for overall effect: Z = 9.15 (P < 0.00001)						
Test for subgroup differences: $Chi^2 = 3.53$, df = 2 (P = 0.17), $I^2 = 43.3\%$						

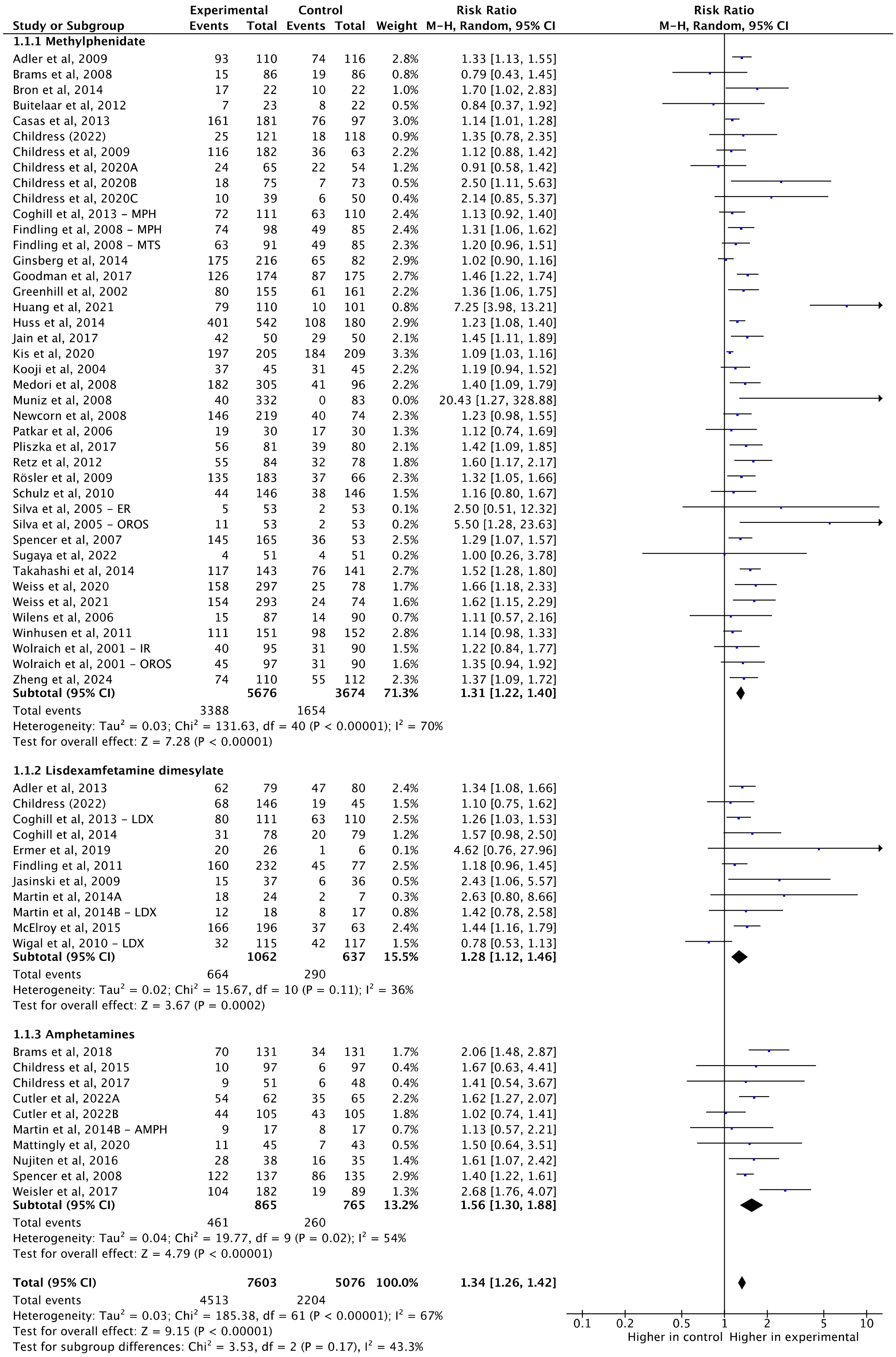

eFigure 1D. Forest plot comparing the risk of overall adverse events in patients receiving stimulants vs placebo. Figure legend: 95% CI, 95% confidence interval; AMPH, amphetamine; IR, immediate-release; LDX, lisdexamfetamine; M-H, Mantel-Haenszel; MPH, methylphenidate; MTS, transdermal system; OROS, osmotic-controlled release oral delivery system.

E)

Study or Subgroup	Experimental Events	Experimental Total	Control Events	Control Total	Weight	Risk Ratio M-H, Random, 90% CI
**1.1.1 Methylphenidate**						
Adler et al, 2009	93	110	74	116	2.8%	1.33 [1.16, 1.51]
Brams et al, 2008	15	86	19	86	0.8%	0.79 [0.47, 1.31]
Bron et al, 2014	17	22	10	22	1.0%	1.70 [1.11, 2.61]
Buitelaar et al, 2012	7	23	8	22	0.5%	0.84 [0.42, 1.68]
Casas et al, 2013	161	181	76	97	3.0%	1.14 [1.03, 1.25]
Childress (2022)	25	121	18	118	0.9%	1.35 [0.85, 2.15]
Childress et al, 2009	116	182	36	63	2.2%	1.12 [0.91, 1.36]
Childress et al, 2020A	24	65	22	54	1.2%	0.91 [0.62, 1.32]
Childress et al, 2020B	18	75	7	73	0.5%	2.50 [1.27, 4.94]
Childress et al, 2020C	10	39	6	50	0.4%	2.14 [0.99, 4.63]
Coghill et al, 2013 - MPH	72	111	63	110	2.4%	1.13 [0.95, 1.35]
Findling et al, 2008 - MPH	74	98	49	85	2.4%	1.31 [1.09, 1.57]
Findling et al, 2008 - MTS	63	91	49	85	2.3%	1.20 [0.99, 1.45]
Ginsberg et al, 2014	175	216	65	82	2.9%	1.02 [0.92, 1.14]
Goodman et al, 2017	126	174	87	175	2.7%	1.46 [1.26, 1.69]
Greenhill et al, 2002	80	155	61	161	2.2%	1.36 [1.10, 1.68]
Huang et al, 2021	79	110	10	101	0.8%	7.25 [4.38, 12.00]
Huss et al, 2014	401	542	108	180	2.9%	1.23 [1.11, 1.37]
Jain et al, 2017	42	50	29	50	2.1%	1.45 [1.16, 1.81]
Kis et al, 2020	197	205	184	209	3.3%	1.09 [1.04, 1.15]
Kooji et al, 2004	37	45	31	45	2.2%	1.19 [0.98, 1.46]
Medori et al, 2008	182	305	41	96	2.2%	1.40 [1.13, 1.72]
Muniz et al, 2008	40	332	0	83	0.0%	20.43 [1.98, 210.39]
Newcorn et al, 2008	146	219	40	74	2.3%	1.23 [1.02, 1.50]
Patkar et al, 2006	19	30	17	30	1.3%	1.12 [0.79, 1.58]
Pliszka et al, 2017	56	81	39	80	2.1%	1.42 [1.13, 1.78]
Retz et al, 2012	55	84	32	78	1.8%	1.60 [1.23, 2.07]
Rösler et al, 2009	135	183	37	66	2.3%	1.32 [1.08, 1.60]
Schulz et al, 2010	44	146	38	146	1.5%	1.16 [0.85, 1.58]
Silva et al, 2005 - ER	5	53	2	53	0.1%	2.50 [0.66, 9.53]
Silva et al, 2005 - OROS	11	53	2	53	0.2%	5.50 [1.62, 18.69]
Spencer et al, 2007	145	165	36	53	2.5%	1.29 [1.10, 1.52]
Sugaya et al, 2022	4	51	4	51	0.2%	1.00 [0.33, 3.05]
Takahashi et al, 2014	117	143	76	141	2.7%	1.52 [1.31, 1.75]
Weiss et al, 2020	158	297	25	78	1.7%	1.66 [1.25, 2.21]
Weiss et al, 2021	154	293	24	74	1.6%	1.62 [1.21, 2.17]
Wilens et al, 2006	15	87	14	90	0.7%	1.11 [0.63, 1.94]
Winhusen et al, 2011	111	151	98	152	2.8%	1.14 [1.00, 1.30]
Wolraich et al, 2001 - IR	40	95	31	90	1.5%	1.22 [0.90, 1.67]
Wolraich et al, 2001 - OROS	45	97	31	90	1.6%	1.35 [1.00, 1.82]
Zheng et al, 2024	74	110	55	112	2.3%	1.37 [1.13, 1.66]
**Subtotal (90% CI)**		**5676**		**3674**	**71.3%**	**1.31 [1.23, 1.39]**
Total events	3388		1654			
Heterogeneity: Tau² = 0.03; Chi² = 131.63, df = 40 (P < 0.00001); I² = 70%						
Test for overall effect: Z = 7.28 (P < 0.00001)						
**1.1.2 Lisdexamfetamine dimesylate**						
Adler et al, 2013	62	79	47	80	2.4%	1.34 [1.11, 1.60]
Childress (2022)	68	146	19	45	1.5%	1.10 [0.80, 1.52]
Coghill et al, 2013 - LDX	80	111	63	110	2.5%	1.26 [1.07, 1.49]
Coghill et al, 2014	31	78	20	79	1.2%	1.57 [1.06, 2.32]
Ermer et al, 2019	20	26	1	6	0.1%	4.62 [1.02, 20.93]
Findling et al, 2011	160	232	45	77	2.5%	1.18 [0.99, 1.40]
Jasinski et al, 2009	15	37	6	36	0.5%	2.43 [1.21, 4.87]
Martin et al, 2014A	18	24	2	7	0.3%	2.63 [0.96, 7.15]
Martin et al, 2014B - LDX	12	18	8	17	0.8%	1.42 [0.86, 2.35]
McElroy et al, 2015	166	196	37	63	2.4%	1.44 [1.20, 1.73]
Wigal et al, 2010 - LDX	32	115	42	117	1.5%	0.78 [0.56, 1.07]
**Subtotal (90% CI)**		**1062**		**637**	**15.5%**	**1.28 [1.15, 1.43]**
Total events	664		290			
Heterogeneity: Tau² = 0.02; Chi² = 15.67, df = 10 (P = 0.11); I² = 36%						
Test for overall effect: Z = 3.67 (P = 0.0002)						
**1.1.3 Amphetamines**						
Brams et al, 2018	70	131	34	131	1.7%	2.06 [1.56, 2.72]
Childress et al, 2015	10	97	6	97	0.4%	1.67 [0.74, 3.77]
Childress et al, 2017	9	51	6	48	0.4%	1.41 [0.63, 3.15]
Cutler et al, 2022A	54	62	35	65	2.2%	1.62 [1.32, 1.99]
Cutler et al, 2022B	44	105	43	105	1.8%	1.02 [0.78, 1.34]
Martin et al, 2014B - AMPH	9	17	8	17	0.7%	1.13 [0.64, 1.98]
Mattingly et al, 2020	11	45	7	43	0.5%	1.50 [0.74, 3.07]
Nujiten et al, 2016	28	38	16	35	1.4%	1.61 [1.14, 2.27]
Spencer et al, 2008	122	137	86	135	2.9%	1.40 [1.24, 1.57]
Weisler et al, 2017	104	182	19	89	1.3%	2.68 [1.88, 3.80]
**Subtotal (90% CI)**		**865**		**765**	**13.2%**	**1.56 [1.34, 1.82]**
Total events	461		260			
Heterogeneity: Tau² = 0.04; Chi² = 19.77, df = 9 (P = 0.02); I² = 54%						
Test for overall effect: Z = 4.79 (P < 0.00001)						
**Total (90% CI)**		**7603**		**5076**	**100.0%**	**1.34 [1.27, 1.41]**
Total events	4513		2204			
Heterogeneity: Tau² = 0.03; Chi² = 185.38, df = 61 (P < 0.00001); I² = 67%						
Test for overall effect: Z = 9.15 (P < 0.00001)						
Test for subgroup differences: Chi² = 3.53, df = 2 (P = 0.17), I² = 43.3%						

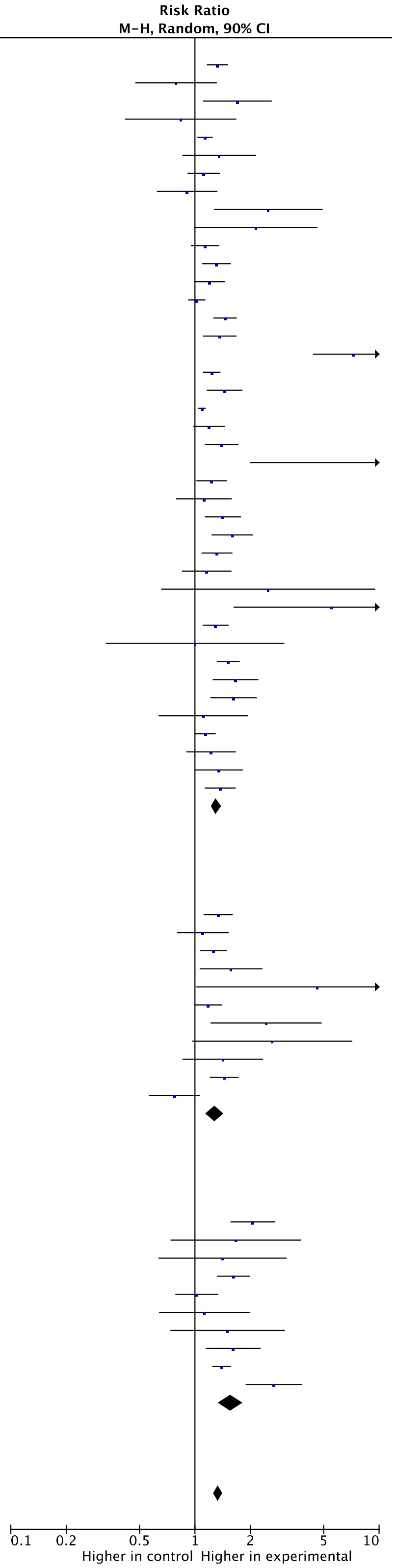

eFigure 1E. Forest plot comparing the risk of overall adverse events in patients receiving stimulants vs placebo. Figure legend: 90% CI, 90% confidence interval; AMPH, amphetamine; IR, immediate-release; LDX, lisdexamfetamine; M-H, Mantel-Haenszel; MPH, methylphenidate; MTS, transdermal system; OROS, osmotic-controlled release oral delivery system.

eFigure 2-8: Forest plots dividing groups according to different stimulants.

eFigure 2a. Forest Plot Showing the Risk Ratio of **Decreased Appetite** Between Control and Experimental Groups (90% confidence interval).

Study or Subgroup	Experimental Events	Experimental Total	Control Events	Control Total	Weight	Risk Ratio M-H, Random, 95% CI
**3.1.1 Methylphenidate**						
Adler et al, 2009	28	110	7	116	2.3%	4.22 [1.92, 9.26]
Biederman et al, 2006	23	67	2	74	1.3%	12.70 [3.11, 51.85]
Casas et al, 2013	43	181	5	97	2.1%	4.61 [1.89, 11.25]
Childress et al, 2009	22	182	3	63	1.6%	2.54 [0.79, 8.19]
Chronis-Tuscano et al, 2008	2	20	1	23	0.6%	2.30 [0.23, 23.51]
Coghill et al, 2013 - MPH	17	111	3	110	1.5%	5.62 [1.69, 18.62]
Findling et al, 2008 - MPH	25	98	4	85	1.8%	5.42 [1.97, 14.95]
Findling et al, 2008 - MTS	17	91	4	85	1.8%	3.97 [1.39, 11.32]
Froehlich et al, 2020	106	171	45	171	3.4%	2.36 [1.79, 3.11]
Ginsberg et al, 2014	15	216	11	82	2.4%	0.52 [0.25, 1.08]
Goodman et al, 2017	25	174	7	175	2.2%	3.59 [1.60, 8.09]
Greenhill et al, 2002	15	155	4	161	1.7%	3.90 [1.32, 11.48]
Huang et al, 2021	53	110	1	101	0.8%	48.66 [6.86, 345.42]
Huss et al, 2014	136	542	8	180	2.5%	5.65 [2.82, 11.29]
Jain et al, 2017	11	50	3	50	1.5%	3.67 [1.09, 12.36]
Kis et al, 2020	46	205	8	209	2.4%	5.86 [2.84, 12.11]
Kooji et al, 2004	10	45	2	45	1.2%	5.00 [1.16, 21.55]
Lee et al, 2011	77	157	31	157	3.2%	2.48 [1.74, 3.54]
Medori et al, 2008	77	305	7	96	2.4%	3.46 [1.65, 7.25]
Muniz et al, 2008	3	332	0	83	0.4%	1.77 [0.09, 33.86]
Newcorn et al, 2008	37	219	2	74	1.3%	6.25 [1.54, 25.31]
Patkar et al, 2006	3	30	1	30	0.6%	3.00 [0.33, 27.23]
Pearson et al, 2013	9	24	1	24	0.8%	9.00 [1.23, 65.64]
Pelham Jr et al, 2005	22	36	2	36	1.3%	11.00 [2.79, 43.37]
Pliszka et al, 2017	15	81	3	80	1.5%	4.94 [1.49, 16.40]
Ramtvedt et al, 2014 - MPH	25	34	17	34	3.2%	1.47 [0.99, 2.18]
Retz et al, 2012	40	84	8	78	2.5%	4.64 [2.32, 9.29]
Rosenberg et al, 2014	8	29	7	29	2.1%	1.14 [0.48, 2.74]
Rösler et al, 2009	70	183	9	66	2.6%	2.81 [1.49, 5.29]
Schulz et al, 2010	3	146	2	146	0.9%	1.50 [0.25, 8.85]
Spencer et al, 2007	30	165	6	53	2.2%	1.61 [0.71, 3.65]
Stein et al, 2003	35	47	16	47	3.1%	2.19 [1.42, 3.37]
Sugaya et al, 2022	13	51	3	51	1.5%	4.33 [1.31, 14.30]
Weiss et al, 2020	33	297	2	78	1.3%	4.33 [1.06, 17.67]
Wigal et al, 2006 - MPH	23	162	14	160	2.6%	1.62 [0.87, 3.04]
Zheng et al, 2024	33	110	2	112	1.3%	16.80 [4.13, 68.32]
**Subtotal (95% CI)**		**5020**		**3261**	**65.8%**	**3.32 [2.61, 4.23]**
Total events	1150		251			
Heterogeneity: Tau^2 = 0.27; Chi^2 = 98.34, df = 35 (P < 0.00001); I^2 = 64%						
Test for overall effect: Z = 9.77 (P < 0.00001)						
**3.1.2 Lisdexamfetamine**						
Childress (2022)	20	146	4	45	1.8%	1.54 [0.56, 4.27]
Coghill et al, 2013 - LDX	28	111	3	110	1.6%	9.25 [2.90, 29.54]
Findling et al, 2011	79	232	2	77	1.3%	13.11 [3.30, 52.08]
Martin et al, 2014B - LDX	3	18	1	17	0.7%	2.83 [0.33, 24.66]
McElroy et al, 2015	42	196	4	63	1.9%	3.38 [1.26, 9.04]
Mooney et al, 2015	12	22	7	21	2.4%	1.64 [0.80, 3.35]
Wigal et al, 2010 - LDX	4	115	2	117	1.0%	2.03 [0.38, 10.89]
**Subtotal (95% CI)**		**840**		**450**	**10.7%**	**3.33 [1.63, 6.80]**
Total events	188		23			
Heterogeneity: Tau^2 = 0.53; Chi^2 = 15.35, df = 6 (P = 0.02); I^2 = 61%						
Test for overall effect: Z = 3.30 (P = 0.0010)						
**3.1.3 Amphetamines**						
Brams et al, 2018	40	132	9	131	2.5%	4.41 [2.23, 8.72]
Cutler et al, 2022A	30	62	14	65	2.9%	2.25 [1.32, 3.82]
Cutler et al, 2022B	13	105	2	105	1.2%	6.50 [1.50, 28.10]
Faraone et al, 2021	25	214	5	107	2.0%	2.50 [0.98, 6.35]
Martin et al, 2014B - AMPH	4	17	1	17	0.7%	4.00 [0.50, 32.20]
Mattingly et al, 2020	1	45	2	43	0.6%	0.48 [0.04, 5.08]
McCracken et al, 2003	22	48	11	49	2.7%	2.04 [1.12, 3.74]
McCracken et al, 2003 - XR	27	49	11	49	2.7%	2.45 [1.38, 4.38]
Nujiten et al, 2016	6	38	2	35	1.1%	2.76 [0.60, 12.80]
Ramtvedt et al, 2014 - AMPH	24	34	17	34	3.1%	1.41 [0.95, 2.11]
Spencer et al, 2006	83	226	1	52	0.8%	19.10 [2.72, 134.04]
Spencer et al, 2008	27	137	2	135	1.2%	13.30 [3.23, 54.84]
Weisler et al, 2017	45	182	4	89	1.9%	5.50 [2.04, 14.82]
**Subtotal (95% CI)**		**1289**		**911**	**23.5%**	**3.06 [2.01, 4.65]**
Total events	347		81			
Heterogeneity: Tau^2 = 0.31; Chi^2 = 33.83, df = 12 (P = 0.0007); I^2 = 65%						
Test for overall effect: Z = 5.24 (P < 0.00001)						
**Total (95% CI)**		**7149**		**4622**	**100.0%**	**3.24 [2.67, 3.94]**
Total events	1685		355			
Heterogeneity: Tau^2 = 0.28; Chi^2 = 148.58, df = 55 (P < 0.00001); I^2 = 63%						
Test for overall effect: Z = 11.78 (P < 0.00001)						
Test for subgroup differences: Chi^2 = 0.11, df = 2 (P = 0.94), I^2 = 0%						

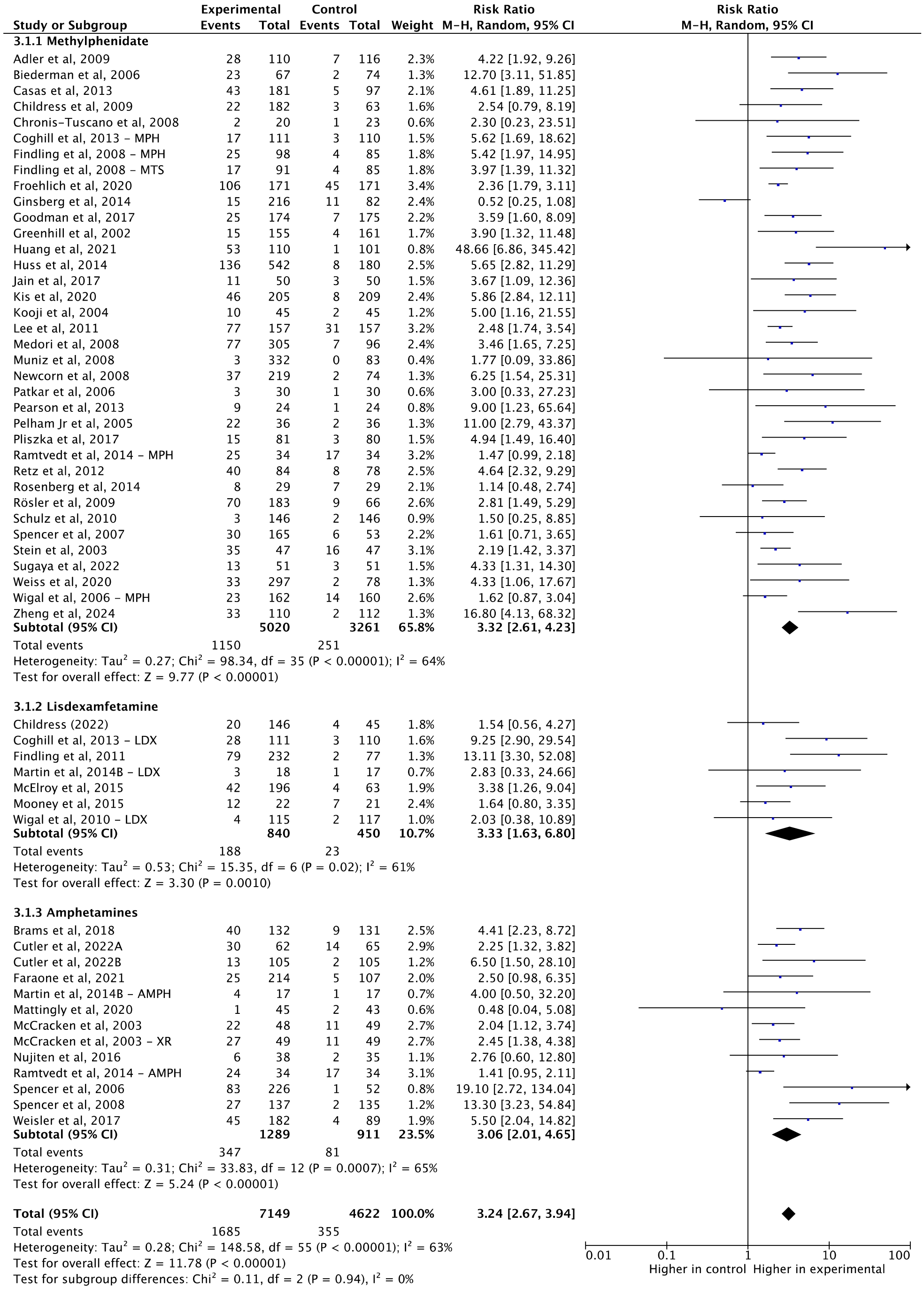

Figure legend: 95%CI, 95% confidence interval; AMPH, amphetamine; LDX, lisdexanfetamine; M-H, Mantel–Haenszel; MPH, methylphenidate; MTS, transdermal system; XR, extended-release.

eFigure 2b. Forest Plot Showing the Risk Ratio of **Decreased Appetite** Between Control and Experimental Groups (90% confidence interval).

Study or Subgroup	Experimental Events	Experimental Total	Control Events	Control Total	Weight	Risk Ratio M-H, Random, 90% CI
**3.1.1 Methylphenidate**						
Adler et al, 2009	28	110	7	116	2.3%	4.22 [2.18, 8.16]
Biederman et al, 2006	23	67	2	74	1.3%	12.70 [3.90, 41.35]
Casas et al, 2013	43	181	5	97	2.1%	4.61 [2.18, 9.75]
Childress et al, 2009	22	182	3	63	1.6%	2.54 [0.95, 6.79]
Chronis-Tuscano et al, 2008	2	20	1	23	0.6%	2.30 [0.33, 16.18]
Coghill et al, 2013 - MPH	17	111	3	110	1.5%	5.62 [2.05, 15.36]
Findling et al, 2008 - MPH	25	98	4	85	1.8%	5.42 [2.31, 12.70]
Findling et al, 2008 - MTS	17	91	4	85	1.8%	3.97 [1.65, 9.57]
Froehlich et al, 2020	106	171	45	171	3.4%	2.36 [1.87, 2.97]
Ginsberg et al, 2014	15	216	11	82	2.4%	0.52 [0.28, 0.96]
Goodman et al, 2017	25	174	7	175	2.2%	3.59 [1.82, 7.10]
Greenhill et al, 2002	15	155	4	161	1.7%	3.90 [1.57, 9.65]
Huang et al, 2021	53	110	1	101	0.8%	48.66 [9.40, 252.06]
Huss et al, 2014	136	542	8	180	2.5%	5.65 [3.16, 10.10]
Jain et al, 2017	11	50	3	50	1.5%	3.67 [1.32, 10.16]
Kis et al, 2020	46	205	8	209	2.4%	5.86 [3.19, 10.78]
Kooji et al, 2004	10	45	2	45	1.2%	5.00 [1.47, 17.04]
Lee et al, 2011	77	157	31	157	3.2%	2.48 [1.85, 3.34]
Medori et al, 2008	77	305	7	96	2.4%	3.46 [1.86, 6.44]
Muniz et al, 2008	3	332	0	83	0.4%	1.77 [0.15, 21.06]
Newcorn et al, 2008	37	219	2	74	1.3%	6.25 [1.93, 20.21]
Patkar et al, 2006	3	30	1	30	0.6%	3.00 [0.47, 19.10]
Pearson et al, 2013	9	24	1	24	0.8%	9.00 [1.70, 47.69]
Pelham Jr et al, 2005	22	36	2	36	1.3%	11.00 [3.48, 34.78]
Pliszka et al, 2017	15	81	3	80	1.5%	4.94 [1.80, 13.52]
Ramtvedt et al, 2014 - MPH	25	34	17	34	3.2%	1.47 [1.06, 2.04]
Retz et al, 2012	40	84	8	78	2.5%	4.64 [2.59, 8.31]
Rosenberg et al, 2014	8	29	7	29	2.1%	1.14 [0.55, 2.38]
Rösler et al, 2009	70	183	9	66	2.6%	2.81 [1.65, 4.78]
Schulz et al, 2010	3	146	2	146	0.9%	1.50 [0.34, 6.65]
Spencer et al, 2007	30	165	6	53	2.2%	1.61 [0.81, 3.20]
Stein et al, 2003	35	47	16	47	3.1%	2.19 [1.52, 3.14]
Sugaya et al, 2022	13	51	3	51	1.5%	4.33 [1.59, 11.80]
Weiss et al, 2020	33	297	2	78	1.3%	4.33 [1.33, 14.09]
Wigal et al, 2006 - MPH	23	162	14	160	2.6%	1.62 [0.96, 2.75]
Zheng et al, 2024	33	110	2	112	1.3%	16.80 [5.18, 54.53]
**Subtotal (90% CI)**		**5020**		**3261**	**65.8%**	**3.32 [2.71, 4.07]**
Total events	1150		251			
Heterogeneity: $Tau^2 = 0.27$; $Chi^2 = 98.34$, df = 35 ($P < 0.00001$); $I^2 = 64\%$						
Test for overall effect: Z = 9.77 ($P < 0.00001$)						
**3.1.2 Lisdexamfetamine**						
Childress (2022)	20	146	4	45	1.8%	1.54 [0.65, 3.63]
Coghill et al, 2013 - LDX	28	111	3	110	1.6%	9.25 [3.49, 24.51]
Findling et al, 2011	79	232	2	77	1.3%	13.11 [4.12, 41.72]
Martin et al, 2014B - LDX	3	18	1	17	0.7%	2.83 [0.46, 17.42]
McElroy et al, 2015	42	196	4	63	1.9%	3.38 [1.48, 7.72]
Mooney et al, 2015	12	22	7	21	2.4%	1.64 [0.90, 2.98]
Wigal et al, 2010 - LDX	4	115	2	117	1.0%	2.03 [0.50, 8.32]
**Subtotal (90% CI)**		**840**		**450**	**10.7%**	**3.33 [1.83, 6.06]**
Total events	188		23			
Heterogeneity: $Tau^2 = 0.53$; $Chi^2 = 15.35$, df = 6 ($P = 0.02$); $I^2 = 61\%$						
Test for overall effect: Z = 3.30 ($P = 0.0010$)						
**3.1.3 Amphetamines**						
Brams et al, 2018	40	132	9	131	2.5%	4.41 [2.49, 7.81]
Cutler et al, 2022A	30	62	14	65	2.9%	2.25 [1.44, 3.51]
Cutler et al, 2022B	13	105	2	105	1.2%	6.50 [1.90, 22.21]
Faraone et al, 2021	25	214	5	107	2.0%	2.50 [1.14, 5.46]
Martin et al, 2014B - AMPH	4	17	1	17	0.7%	4.00 [0.69, 23.03]
Mattingly et al, 2020	1	45	2	43	0.6%	0.48 [0.07, 3.47]
McCracken et al, 2003	22	48	11	49	2.7%	2.04 [1.23, 3.39]
McCracken et al, 2003 - XR	27	49	11	49	2.7%	2.45 [1.51, 3.99]
Nujiten et al, 2016	6	38	2	35	1.1%	2.76 [0.76, 10.00]
Ramtvedt et al, 2014 - AMPH	24	34	17	34	3.1%	1.41 [1.01, 1.98]
Spencer et al, 2006	83	226	1	52	0.8%	19.10 [3.72, 97.99]
Spencer et al, 2008	27	137	2	135	1.2%	13.30 [4.05, 43.67]
Weisler et al, 2017	45	182	4	89	1.9%	5.50 [2.40, 12.63]
**Subtotal (90% CI)**		**1289**		**911**	**23.5%**	**3.06 [2.15, 4.35]**
Total events	347		81			
Heterogeneity: $Tau^2 = 0.31$; $Chi^2 = 33.83$, df = 12 ($P = 0.0007$); $I^2 = 65\%$						
Test for overall effect: Z = 5.24 ($P < 0.00001$)						
**Total (90% CI)**		**7149**		**4622**	**100.0%**	**3.24 [2.75, 3.82]**
Total events	1685		355			
Heterogeneity: $Tau^2 = 0.28$; $Chi^2 = 148.58$, df = 55 ($P < 0.00001$); $I^2 = 63\%$						
Test for overall effect: Z = 11.78 ($P < 0.00001$)						
Test for subgroup differences: $Chi^2 = 0.11$, df = 2 ($P = 0.94$), $I^2 = 0\%$						

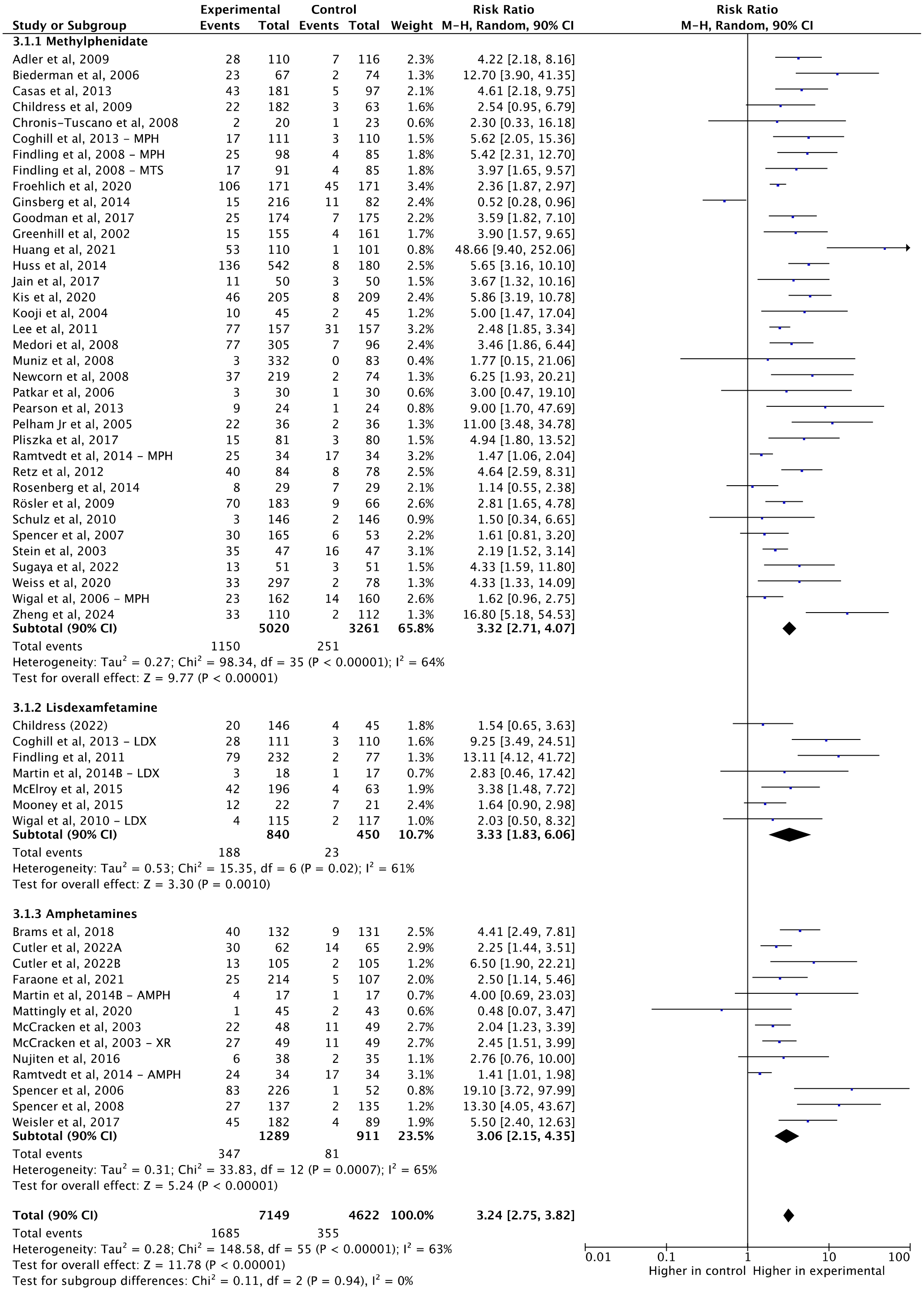

Figure legend: 90%CI, 90% confidence interval; AMPH, amphetamine; LDX, lisdexanfetamine; M-H, Mantel–Haenszel; MPH, methylphenidate; MTS, transdermal system; XR, extended-release.

eFigure 3a. Forest Plot Showing the Risk Ratio of **Headache** Between Control and Experimental Groups (95% confidence interval).

Study or Subgroup	Experimental Events	Experimental Total	Control Events	Control Total	Weight	Risk Ratio M-H, Fixed, 95% CI
**4.1.1 Methylphenidate**						
Adler et al, 2009	28	110	16	116	2.3%	1.85 [1.06, 3.22]
Biederman et al, 2006	21	67	22	74	3.1%	1.05 [0.64, 1.74]
Brams et al, 2018	3	86	2	86	0.3%	1.50 [0.26, 8.75]
Casas et al, 2013	52	181	27	97	5.1%	1.03 [0.70, 1.53]
Childress et al, 2009	22	182	6	63	1.3%	1.27 [0.54, 2.99]
Childress et al, 2020A	1	65	1	54	0.2%	0.83 [0.05, 12.97]
Coghill et al, 2013 - MPH	22	111	22	110	3.2%	0.99 [0.58, 1.68]
Ginsberg et al, 2014	41	216	21	82	4.5%	0.74 [0.47, 1.17]
Goodman et al, 2017	33	174	20	175	2.9%	1.66 [0.99, 2.78]
Greenhill et al, 2002	23	155	17	161	2.4%	1.41 [0.78, 2.53]
Huang et al, 2021	9	110	1	101	0.2%	8.26 [1.07, 64.08]
Huss et al, 2014	111	542	30	180	6.6%	1.23 [0.85, 1.77]
Jain et al, 2017	13	50	12	50	1.8%	1.08 [0.55, 2.14]
Kis et al, 2020	65	205	55	209	8.0%	1.20 [0.89, 1.63]
Konstenius et al, 2014	6	27	2	27	0.3%	3.00 [0.66, 13.56]
Kooji et al, 2004	7	45	2	45	0.3%	3.50 [0.77, 15.94]
Lee et al, 2011	48	157	30	157	4.4%	1.60 [1.07, 2.38]
Medori et al, 2008	64	305	17	96	3.8%	1.18 [0.73, 1.92]
Newcorn et al, 2008	25	219	7	74	1.5%	1.21 [0.54, 2.67]
Patkar et al, 2006	3	30	2	30	0.3%	1.50 [0.27, 8.34]
Pearson et al, 2013	4	24	2	24	0.3%	2.00 [0.40, 9.91]
Pelham Jr et al, 2001 - IR	11	36	16	36	2.3%	0.69 [0.37, 1.27]
Pelham Jr et al, 2001 - LA	8	36	16	36	2.3%	0.50 [0.25, 1.02]
Pelham Jr et al, 2005	9	36	3	36	0.4%	3.00 [0.88, 10.18]
Pliszka et al, 2017	6	81	3	80	0.4%	1.98 [0.51, 7.63]
Quinn et al, 2004	2	31	2	31	0.3%	1.00 [0.15, 6.66]
Ramtvedt et al, 2014 - MPH	9	34	8	34	1.2%	1.13 [0.49, 2.57]
Retz et al, 2012	25	84	13	78	2.0%	1.79 [0.98, 3.24]
Rosenberg et al, 2014	5	29	4	29	0.6%	1.25 [0.37, 4.19]
Schulz et al, 2010	7	146	5	146	0.7%	1.40 [0.45, 4.31]
Shram et al, 2022	5	48	2	46	0.3%	2.40 [0.49, 11.74]
Spencer et al, 2007	52	165	10	53	2.2%	1.67 [0.91, 3.05]
Stein et al, 2003	17	47	16	47	2.3%	1.06 [0.61, 1.84]
Sugaya et al, 2022	3	51	3	51	0.4%	1.00 [0.21, 4.72]
Weiss et al, 2020	52	297	9	78	2.1%	1.52 [0.78, 2.94]
Weiss et al, 2021	44	293	7	74	1.6%	1.59 [0.75, 3.38]
Wigal et al, 2004	17	90	4	42	0.8%	1.98 [0.71, 5.53]
Wilens et al, 2006	3	87	6	90	0.9%	0.52 [0.13, 2.00]
**Subtotal (95% CI)**		**4652**		**2998**	**73.7%**	**1.28 [1.15, 1.42]**
Total events	876		441			
Heterogeneity: $Chi^2 = 38.17$, df = 37 (P = 0.42); $I^2 = 3\%$						
Test for overall effect: Z = 4.53 (P < 0.00001)						
**4.1.2 Lisdexamfetamine**						
Coghill et al, 2013 - LDX	16	111	22	110	3.2%	0.72 [0.40, 1.30]
Coghill et al, 2014	6	78	5	79	0.7%	1.22 [0.39, 3.82]
Findling et al, 2011	34	232	10	77	2.2%	1.13 [0.59, 2.18]
Jasinski et al, 2009	6	37	1	36	0.1%	5.84 [0.74, 46.11]
Martin et al, 2014B - LDX	1	18	3	17	0.5%	0.31 [0.04, 2.74]
McElroy et al, 2015	23	196	6	63	1.3%	1.23 [0.53, 2.89]
Mooney et al, 2015	10	22	2	21	0.3%	4.77 [1.18, 19.27]
Wigal et al, 2010 - LDX	2	115	3	117	0.4%	0.68 [0.12, 3.98]
**Subtotal (95% CI)**		**809**		**520**	**8.8%**	**1.14 [0.82, 1.58]**
Total events	98		52			
Heterogeneity: $Chi^2 = 10.52$, df = 7 (P = 0.16); $I^2 = 33\%$						
Test for overall effect: Z = 0.79 (P = 0.43)						
**4.1.3 Amphetamines**						
Brams et al, 2018	16	132	14	131	2.1%	1.13 [0.58, 2.23]
Childress et al, 2017	1	51	1	48	0.2%	0.94 [0.06, 14.63]
Cutler et al, 2022B	6	105	4	105	0.6%	1.50 [0.44, 5.16]
Faraone et al, 2021	26	214	13	107	2.5%	1.00 [0.54, 1.87]
Martin et al, 2014B - AMPH	1	17	3	17	0.4%	0.33 [0.04, 2.89]
Mattingly et al, 2020	2	45	3	43	0.4%	0.64 [0.11, 3.63]
McCracken et al, 2003	12	48	12	49	1.7%	1.02 [0.51, 2.04]
McCracken et al, 2003 - XR	12	49	12	49	1.8%	1.00 [0.50, 2.00]
Nujiten et al, 2016	1	38	1	35	0.2%	0.92 [0.06, 14.17]
Ramtvedt et al, 2014 - AMPH	8	34	8	34	1.2%	1.00 [0.42, 2.36]
Spencer et al, 2006	38	226	12	54	2.8%	0.76 [0.42, 1.35]
Spencer et al, 2008	25	137	19	135	2.8%	1.30 [0.75, 2.24]
Weisler et al, 2017	17	182	4	89	0.8%	2.08 [0.72, 5.99]
**Subtotal (95% CI)**		**1278**		**896**	**17.5%**	**1.06 [0.85, 1.34]**
Total events	165		106			
Heterogeneity: $Chi^2 = 5.27$, df = 12 (P = 0.95); $I^2 = 0\%$						
Test for overall effect: Z = 0.53 (P = 0.60)						
**Total (95% CI)**		**6739**		**4414**	**100.0%**	**1.23 [1.12, 1.34]**
Total events	1139		599			
Heterogeneity: $Chi^2 = 55.93$, df = 58 (P = 0.55); $I^2 = 0\%$						
Test for overall effect: Z = 4.36 (P < 0.0001)						
Test for subgroup differences: $Chi^2 = 2.20$, df = 2 (P = 0.33), $I^2 = 8.9\%$						

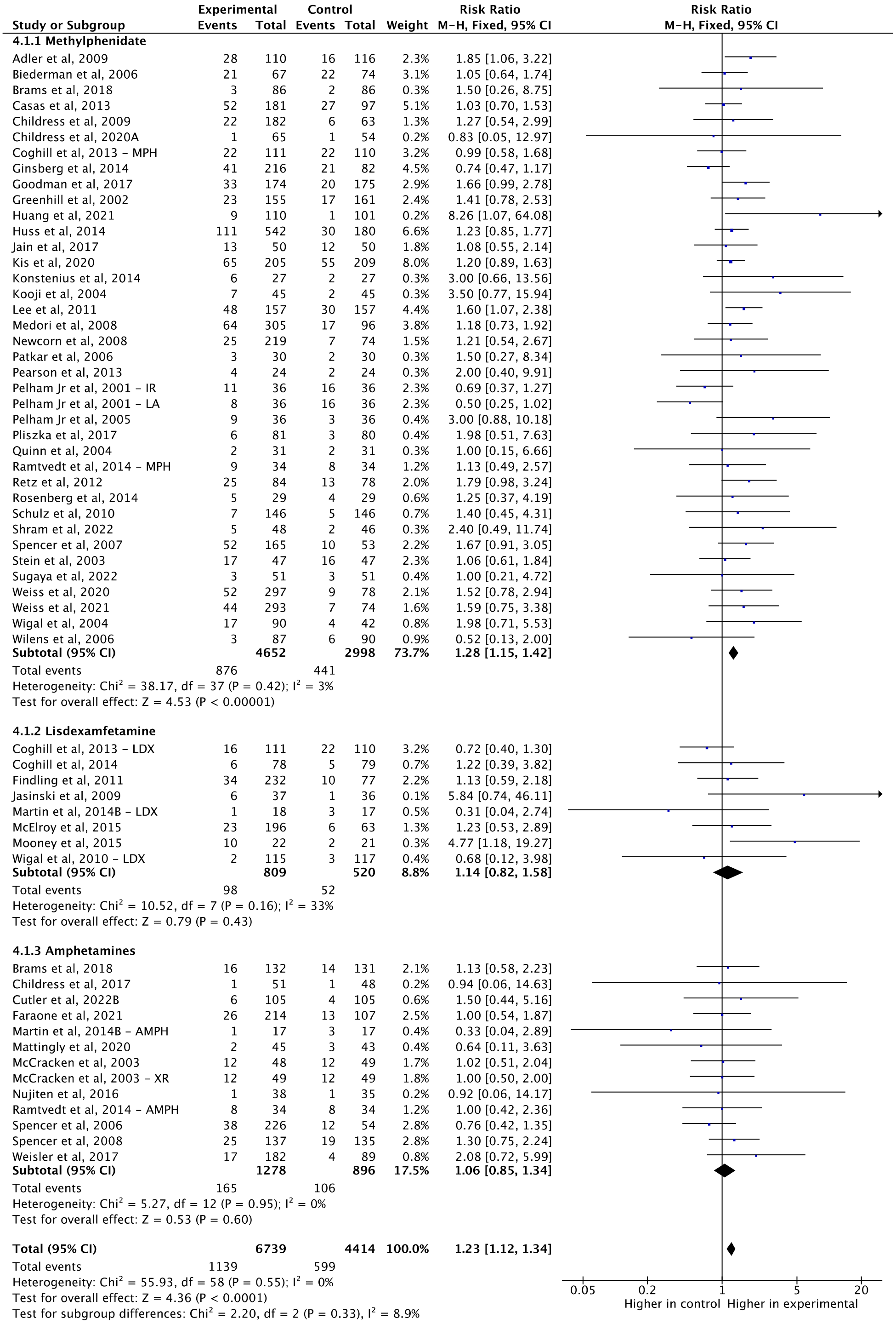

Figure legend: 95%CI, 95% confidence interval; AMPH, amphetamine; LA, long-acting; IR, immediate-release; LDX, lisdexanfetamine; M-H, Mantel–Haenszel; MPH, methylphenidate; XR, extended-release.

eFigure 3b. Forest Plot Showing the Risk Ratio of **Headache** Between Control and Experimental Groups (90% confidence interval).

Study or Subgroup	Experimental Events	Experimental Total	Control Events	Control Total	Weight	Risk Ratio M-H, Fixed, 90% CI
**4.1.1 Methylphenidate**						
Adler et al, 2009	28	110	16	116	2.3%	1.85 [1.16, 2.94]
Biederman et al, 2006	21	67	22	74	3.1%	1.05 [0.69, 1.60]
Brams et al, 2018	3	86	2	86	0.3%	1.50 [0.34, 6.59]
Casas et al, 2013	52	181	27	97	5.1%	1.03 [0.74, 1.44]
Childress et al, 2009	22	182	6	63	1.3%	1.27 [0.62, 2.60]
Childress et al, 2020A	1	65	1	54	0.2%	0.83 [0.08, 8.34]
Coghill et al, 2013 - MPH	22	111	22	110	3.2%	0.99 [0.64, 1.54]
Ginsberg et al, 2014	41	216	21	82	4.5%	0.74 [0.50, 1.09]
Goodman et al, 2017	33	174	20	175	2.9%	1.66 [1.08, 2.56]
Greenhill et al, 2002	23	155	17	161	2.4%	1.41 [0.86, 2.30]
Huang et al, 2021	9	110	1	101	0.2%	8.26 [1.48, 46.10]
Huss et al, 2014	111	542	30	180	6.6%	1.23 [0.90, 1.67]
Jain et al, 2017	13	50	12	50	1.8%	1.08 [0.61, 1.92]
Kis et al, 2020	65	205	55	209	8.0%	1.20 [0.93, 1.55]
Konstenius et al, 2014	6	27	2	27	0.3%	3.00 [0.85, 10.64]
Kooji et al, 2004	7	45	2	45	0.3%	3.50 [0.98, 12.49]
Lee et al, 2011	48	157	30	157	4.4%	1.60 [1.14, 2.24]
Medori et al, 2008	64	305	17	96	3.8%	1.18 [0.79, 1.78]
Newcorn et al, 2008	25	219	7	74	1.5%	1.21 [0.62, 2.35]
Patkar et al, 2006	3	30	2	30	0.3%	1.50 [0.36, 6.33]
Pearson et al, 2013	4	24	2	24	0.3%	2.00 [0.52, 7.66]
Pelham Jr et al, 2001 - IR	11	36	16	36	2.3%	0.69 [0.41, 1.15]
Pelham Jr et al, 2001 - LA	8	36	16	36	2.3%	0.50 [0.28, 0.91]
Pelham Jr et al, 2005	9	36	3	36	0.4%	3.00 [1.08, 8.37]
Pliszka et al, 2017	6	81	3	80	0.4%	1.98 [0.64, 6.14]
Quinn et al, 2004	2	31	2	31	0.3%	1.00 [0.20, 4.91]
Ramtvedt et al, 2014 - MPH	9	34	8	34	1.2%	1.13 [0.56, 2.25]
Retz et al, 2012	25	84	13	78	2.0%	1.79 [1.08, 2.94]
Rosenberg et al, 2014	5	29	4	29	0.6%	1.25 [0.45, 3.45]
Schulz et al, 2010	7	146	5	146	0.7%	1.40 [0.54, 3.60]
Shram et al, 2022	5	48	2	46	0.3%	2.40 [0.63, 9.09]
Spencer et al, 2007	52	165	10	53	2.2%	1.67 [1.01, 2.77]
Stein et al, 2003	17	47	16	47	2.3%	1.06 [0.67, 1.69]
Sugaya et al, 2022	3	51	3	51	0.4%	1.00 [0.27, 3.68]
Weiss et al, 2020	52	297	9	78	2.1%	1.52 [0.87, 2.65]
Weiss et al, 2021	44	293	7	74	1.6%	1.59 [0.84, 2.99]
Wigal et al, 2004	17	90	4	42	0.8%	1.98 [0.84, 4.69]
Wilens et al, 2006	3	87	6	90	0.9%	0.52 [0.17, 1.61]
**Subtotal (90% CI)**		**4652**		**2998**	**73.7%**	**1.28 [1.17, 1.39]**
Total events	876		441			
Heterogeneity: $Chi^2 = 38.17$, df = 37 (P = 0.42); $I^2 = 3\%$						
Test for overall effect: Z = 4.53 (P < 0.00001)						
**4.1.2 Lisdexamfetamine**						
Coghill et al, 2013 - LDX	16	111	22	110	3.2%	0.72 [0.44, 1.18]
Coghill et al, 2014	6	78	5	79	0.7%	1.22 [0.46, 3.18]
Findling et al, 2011	34	232	10	77	2.2%	1.13 [0.65, 1.96]
Jasinski et al, 2009	6	37	1	36	0.1%	5.84 [1.03, 33.07]
Martin et al, 2014B - LDX	1	18	3	17	0.5%	0.31 [0.05, 1.94]
McElroy et al, 2015	23	196	6	63	1.3%	1.23 [0.60, 2.52]
Mooney et al, 2015	10	22	2	21	0.3%	4.77 [1.48, 15.39]
Wigal et al, 2010 - LDX	2	115	3	117	0.4%	0.68 [0.15, 3.00]
**Subtotal (90% CI)**		**809**		**520**	**8.8%**	**1.14 [0.87, 1.50]**
Total events	98		52			
Heterogeneity: $Chi^2 = 10.52$, df = 7 (P = 0.16); $I^2 = 33\%$						
Test for overall effect: Z = 0.79 (P = 0.43)						
**4.1.3 Amphetamines**						
Brams et al, 2018	16	132	14	131	2.1%	1.13 [0.64, 2.00]
Childress et al, 2017	1	51	1	48	0.2%	0.94 [0.09, 9.41]
Cutler et al, 2022B	6	105	4	105	0.6%	1.50 [0.53, 4.23]
Faraone et al, 2021	26	214	13	107	2.5%	1.00 [0.59, 1.69]
Martin et al, 2014B - AMPH	1	17	3	17	0.4%	0.33 [0.05, 2.04]
Mattingly et al, 2020	2	45	3	43	0.4%	0.64 [0.15, 2.74]
McCracken et al, 2003	12	48	12	49	1.7%	1.02 [0.57, 1.83]
McCracken et al, 2003 - XR	12	49	12	49	1.8%	1.00 [0.56, 1.79]
Nujiten et al, 2016	1	38	1	35	0.2%	0.92 [0.09, 9.13]
Ramtvedt et al, 2014 - AMPH	8	34	8	34	1.2%	1.00 [0.49, 2.05]
Spencer et al, 2006	38	226	12	54	2.8%	0.76 [0.47, 1.23]
Spencer et al, 2008	25	137	19	135	2.8%	1.30 [0.82, 2.05]
Weisler et al, 2017	17	182	4	89	0.8%	2.08 [0.85, 5.06]
**Subtotal (90% CI)**		**1278**		**896**	**17.5%**	**1.06 [0.88, 1.29]**
Total events	165		106			
Heterogeneity: $Chi^2 = 5.27$, df = 12 (P = 0.95); $I^2 = 0\%$						
Test for overall effect: Z = 0.53 (P = 0.60)						
**Total (90% CI)**		**6739**		**4414**	**100.0%**	**1.23 [1.14, 1.32]**
Total events	1139		599			
Heterogeneity: $Chi^2 = 55.93$, df = 58 (P = 0.55); $I^2 = 0\%$						
Test for overall effect: Z = 4.36 (P < 0.0001)						
Test for subgroup differences: $Chi^2 = 2.20$, df = 2 (P = 0.33), $I^2 = 8.9\%$						

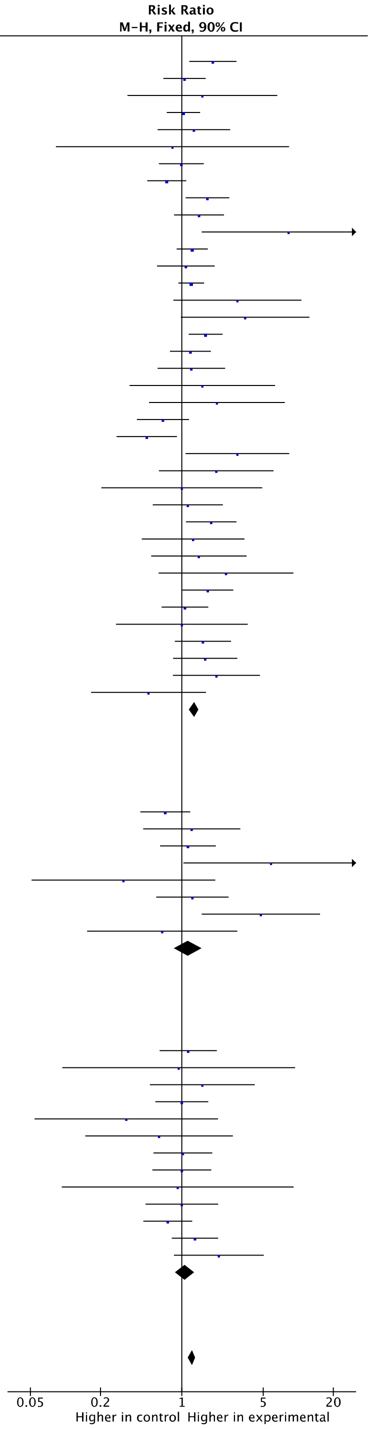

Figure legend: 90%CI, 90% confidence interval; AMPH, amphetamine; LA, long-acting; IR, immediate-release; LDX, lisdexanfetamine; M-H, Mantel–Haenszel; MPH, methylphenidate; XR, extended-release.

eFigure 4a. Forest Plot Showing the Risk Ratio of **Insomnia** Between Control and Experimental Groups (95% confidence interval).

Study or Subgroup	Experimental Events	Experimental Total	Control Events	Control Total	Weight	Risk Ratio M-H, Fixed, 95% CI
**5.1.1 Methylphenidate**						
Adler et al, 2009	10	110	6	116	1.7%	1.76 [0.66, 4.67]
Biederman et al, 2006	12	67	4	74	1.1%	3.31 [1.12, 9.78]
Casas et al, 2013	28	181	11	97	4.1%	1.36 [0.71, 2.62]
Childress et al, 2020A	5	65	5	54	1.6%	0.83 [0.25, 2.72]
Findling et al, 2008 - MPH	13	98	4	85	1.2%	2.82 [0.96, 8.32]
Findling et al, 2008 - MTS	7	91	4	85	1.2%	1.63 [0.50, 5.39]
Froehlich et al, 2020	94	171	37	171	10.6%	2.54 [1.85, 3.48]
Greenhill et al, 2002	11	155	4	161	1.1%	2.86 [0.93, 8.78]
Jain et al, 2017	11	50	4	50	1.1%	2.75 [0.94, 8.06]
Kis et al, 2020	16	205	12	209	3.4%	1.36 [0.66, 2.80]
Konstenius et al, 2014	6	27	2	27	0.6%	3.00 [0.66, 13.56]
Kooji et al, 2004	15	45	10	45	2.9%	1.50 [0.76, 2.98]
Lee et al, 2011	74	157	39	157	11.1%	1.90 [1.38, 2.61]
Medori et al, 2008	41	305	7	96	3.0%	1.84 [0.86, 3.97]
Newcorn et al, 2008	17	219	1	74	0.4%	5.74 [0.78, 42.42]
Patkar et al, 2006	3	30	3	30	0.9%	1.00 [0.22, 4.56]
Pearson et al, 2013	12	24	5	24	1.4%	2.40 [1.00, 5.77]
Pelham Jr et al, 2005	17	36	3	36	0.9%	5.67 [1.82, 17.67]
Pliszka et al, 2017	27	81	9	80	2.6%	2.96 [1.49, 5.90]
Ramtvedt et al, 2014 - MPH	18	34	14	34	4.0%	1.29 [0.77, 2.14]
Rösler et al, 2009	46	183	12	66	5.0%	1.38 [0.78, 2.44]
Spencer et al, 2007	27	165	6	53	2.6%	1.45 [0.63, 3.31]
Stein et al, 2003	30	47	21	47	6.0%	1.43 [0.97, 2.10]
Weiss et al, 2020	18	297	1	78	0.5%	4.73 [0.64, 34.87]
Weiss et al, 2021	19	293	1	74	0.5%	4.80 [0.65, 35.27]
Wigal et al, 2006 - MPH	22	162	13	160	3.7%	1.67 [0.87, 3.20]
Zheng et al, 2024	3	110	2	112	0.6%	1.53 [0.26, 8.96]
**Subtotal (95% CI)**		**3408**		**2295**	**73.7%**	**1.96 [1.72, 2.24]**
Total events	602		240			
Heterogeneity: $Chi^2 = 25.84$, df = 26 (P = 0.47); $I^2 = 0\%$						
Test for overall effect: Z = 9.84 (P < 0.00001)						
**5.1.2 Lisdexamfetamine**						
Findling et al, 2011	26	232	3	77	1.3%	2.88 [0.90, 9.24]
McElroy et al, 2015	26	196	5	63	2.2%	1.67 [0.67, 4.17]
Mooney et al, 2015	7	22	2	21	0.6%	3.34 [0.78, 14.29]
Wigal et al, 2010 - LDX	3	115	2	117	0.6%	1.53 [0.26, 8.96]
**Subtotal (95% CI)**		**565**		**278**	**4.6%**	**2.20 [1.21, 4.03]**
Total events	62		12			
Heterogeneity: $Chi^2 = 1.03$, df = 3 (P = 0.79); $I^2 = 0\%$						
Test for overall effect: Z = 2.57 (P = 0.01)						
**5.1.3 Amphetamines**						
Brams et al, 2018	22	132	3	131	0.9%	7.28 [2.23, 23.73]
Childress et al, 2017	1	51	1	48	0.3%	0.94 [0.06, 14.63]
Cutler et al, 2022A	14	62	8	65	2.2%	1.83 [0.83, 4.07]
Cutler et al, 2022B	6	105	5	105	1.4%	1.20 [0.38, 3.81]
Faraone et al, 2021	19	214	4	107	1.5%	2.38 [0.83, 6.81]
McCracken et al, 2003	17	48	10	49	2.8%	1.74 [0.89, 3.40]
McCracken et al, 2003 - XR	14	49	10	49	2.9%	1.40 [0.69, 2.84]
Nujiten et al, 2016	13	38	3	35	0.9%	3.99 [1.24, 12.84]
Ramtvedt et al, 2014 - AMPH	30	34	14	34	4.0%	2.14 [1.41, 3.26]
Spencer et al, 2006	28	226	2	52	0.9%	3.22 [0.79, 13.10]
Spencer et al, 2008	40	137	12	135	3.5%	3.28 [1.80, 5.98]
Weisler et al, 2017	22	182	1	89	0.4%	10.76 [1.47, 78.54]
**Subtotal (95% CI)**		**1278**		**899**	**21.7%**	**2.56 [2.00, 3.26]**
Total events	226		73			
Heterogeneity: $Chi^2 = 13.94$, df = 11 (P = 0.24); $I^2 = 21\%$						
Test for overall effect: Z = 7.55 (P < 0.00001)						
**Total (95% CI)**		**5251**		**3472**	**100.0%**	**2.10 [1.87, 2.36]**
Total events	890		325			
Heterogeneity: $Chi^2 = 42.80$, df = 42 (P = 0.44); $I^2 = 2\%$						
Test for overall effect: Z = 12.60 (P < 0.00001)						
Test for subgroup differences: $Chi^2 = 3.53$, df = 2 (P = 0.17), $I^2 = 43.3\%$						

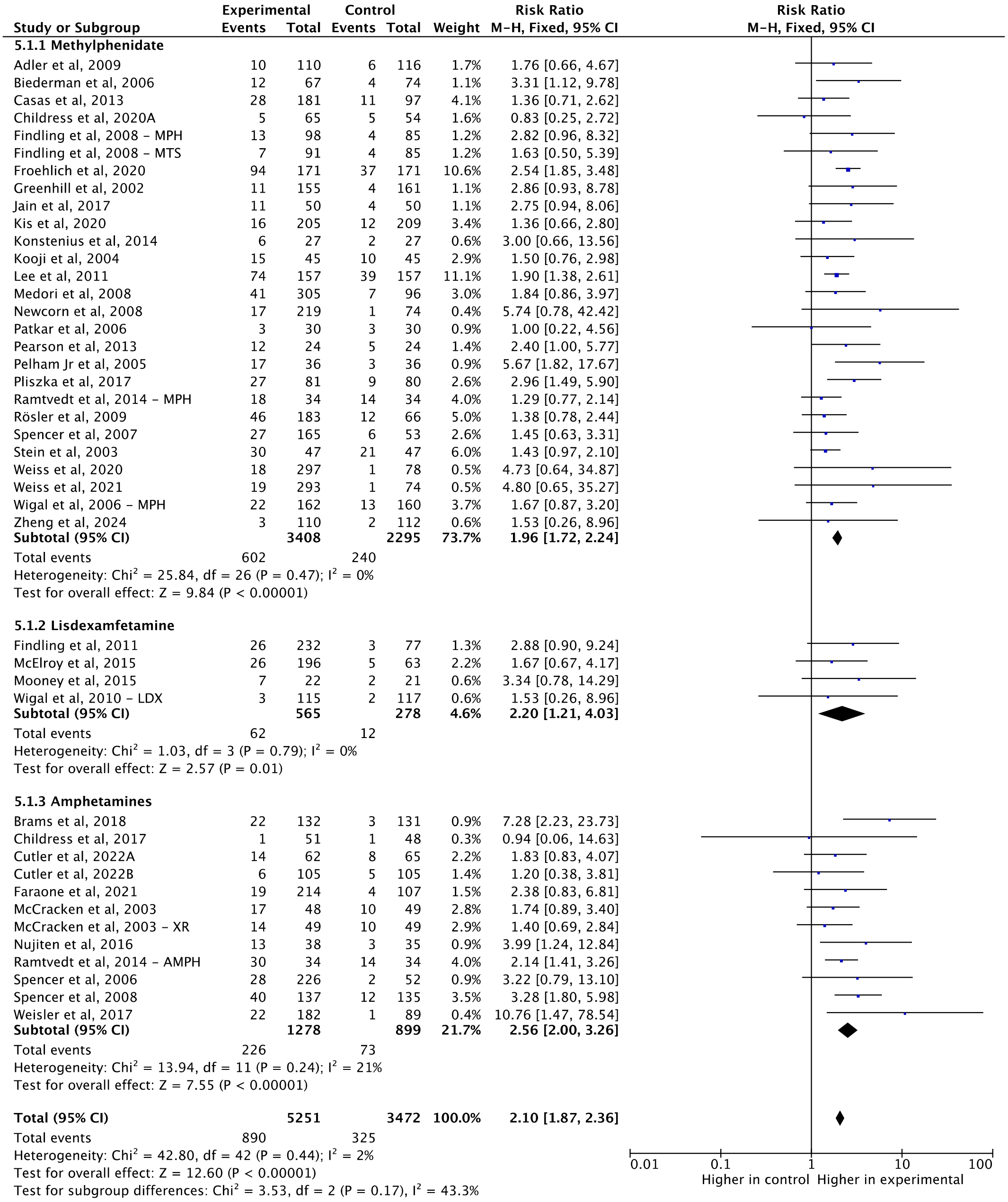

Figure legend: 95%CI, 95% confidence interval; AMPH, amphetamine; LDX, lisdexanfetamine; M-H, Mantel–Haenszel; MPH, methylphenidate; MTS, transdermal system; XR, extended-release.

eFigure 4b. Forest Plot Showing the Risk Ratio of **Insomnia** Between Control and Experimental Groups (90% confidence interval).

Study or Subgroup	Experimental Events	Experimental Total	Control Events	Control Total	Weight	Risk Ratio M-H, Fixed, 90% CI
**5.1.1 Methylphenidate**						
Adler et al, 2009	10	110	6	116	1.7%	1.76 [0.77, 3.99]
Biederman et al, 2006	12	67	4	74	1.1%	3.31 [1.34, 8.22]
Casas et al, 2013	28	181	11	97	4.1%	1.36 [0.79, 2.36]
Childress et al, 2020A	5	65	5	54	1.6%	0.83 [0.31, 2.25]
Findling et al, 2008 - MPH	13	98	4	85	1.2%	2.82 [1.14, 6.99]
Findling et al, 2008 - MTS	7	91	4	85	1.2%	1.63 [0.60, 4.45]
Froehlich et al, 2020	94	171	37	171	10.6%	2.54 [1.95, 3.31]
Greenhill et al, 2002	11	155	4	161	1.1%	2.86 [1.11, 7.33]
Jain et al, 2017	11	50	4	50	1.1%	2.75 [1.12, 6.78]
Kis et al, 2020	16	205	12	209	3.4%	1.36 [0.74, 2.49]
Konstenius et al, 2014	6	27	2	27	0.6%	3.00 [0.85, 10.64]
Kooji et al, 2004	15	45	10	45	2.9%	1.50 [0.84, 2.67]
Lee et al, 2011	74	157	39	157	11.1%	1.90 [1.45, 2.48]
Medori et al, 2008	41	305	7	96	3.0%	1.84 [0.97, 3.51]
Newcorn et al, 2008	17	219	1	74	0.4%	5.74 [1.07, 30.76]
Patkar et al, 2006	3	30	3	30	0.9%	1.00 [0.28, 3.58]
Pearson et al, 2013	12	24	5	24	1.4%	2.40 [1.15, 5.01]
Pelham Jr et al, 2005	17	36	3	36	0.9%	5.67 [2.18, 14.72]
Pliszka et al, 2017	27	81	9	80	2.6%	2.96 [1.66, 5.28]
Ramtvedt et al, 2014 - MPH	18	34	14	34	4.0%	1.29 [0.84, 1.98]
Rösler et al, 2009	46	183	12	66	5.0%	1.38 [0.86, 2.23]
Spencer et al, 2007	27	165	6	53	2.6%	1.45 [0.72, 2.90]
Stein et al, 2003	30	47	21	47	6.0%	1.43 [1.03, 1.97]
Weiss et al, 2020	18	297	1	78	0.5%	4.73 [0.88, 25.29]
Weiss et al, 2021	19	293	1	74	0.5%	4.80 [0.90, 25.59]
Wigal et al, 2006 - MPH	22	162	13	160	3.7%	1.67 [0.97, 2.88]
Zheng et al, 2024	3	110	2	112	0.6%	1.53 [0.35, 6.74]
**Subtotal (90% CI)**		**3408**		**2295**	**73.7%**	**1.96 [1.75, 2.20]**
Total events	602		240			
Heterogeneity: Chi^2 = 25.84, df = 26 (P = 0.47); I^2 = 0%						
Test for overall effect: Z = 9.84 (P < 0.00001)						
**5.1.2 Lisdexamfetamine**						
Findling et al, 2011	26	232	3	77	1.3%	2.88 [1.08, 7.66]
McElroy et al, 2015	26	196	5	63	2.2%	1.67 [0.78, 3.60]
Mooney et al, 2015	7	22	2	21	0.6%	3.34 [0.99, 11.31]
Wigal et al, 2010 - LDX	3	115	2	117	0.6%	1.53 [0.35, 6.74]
**Subtotal (90% CI)**		**565**		**278**	**4.6%**	**2.20 [1.33, 3.65]**
Total events	62		12			
Heterogeneity: Chi^2 = 1.03, df = 3 (P = 0.79); I^2 = 0%						
Test for overall effect: Z = 2.57 (P = 0.01)						
**5.1.3 Amphetamines**						
Brams et al, 2018	22	132	3	131	0.9%	7.28 [2.70, 19.62]
Childress et al, 2017	1	51	1	48	0.3%	0.94 [0.09, 9.41]
Cutler et al, 2022A	14	62	8	65	2.2%	1.83 [0.94, 3.58]
Cutler et al, 2022B	6	105	5	105	1.4%	1.20 [0.45, 3.16]
Faraone et al, 2021	19	214	4	107	1.5%	2.38 [0.98, 5.75]
McCracken et al, 2003	17	48	10	49	2.8%	1.74 [0.99, 3.05]
McCracken et al, 2003 - XR	14	49	10	49	2.9%	1.40 [0.77, 2.54]
Nujiten et al, 2016	13	38	3	35	0.9%	3.99 [1.50, 10.64]
Ramtvedt et al, 2014 - AMPH	30	34	14	34	4.0%	2.14 [1.51, 3.05]
Spencer et al, 2006	28	226	2	52	0.9%	3.22 [0.99, 10.45]
Spencer et al, 2008	40	137	12	135	3.5%	3.28 [1.99, 5.43]
Weisler et al, 2017	22	182	1	89	0.4%	10.76 [2.03, 57.05]
**Subtotal (90% CI)**		**1278**		**899**	**21.7%**	**2.56 [2.08, 3.14]**
Total events	226		73			
Heterogeneity: Chi^2 = 13.94, df = 11 (P = 0.24); I^2 = 21%						
Test for overall effect: Z = 7.55 (P < 0.00001)						
**Total (90% CI)**		**5251**		**3472**	**100.0%**	**2.10 [1.91, 2.32]**
Total events	890		325			
Heterogeneity: Chi^2 = 42.80, df = 42 (P = 0.44); I^2 = 2%						
Test for overall effect: Z = 12.60 (P < 0.00001)						
Test for subgroup differences: Chi^2 = 3.53, df = 2 (P = 0.17), I^2 = 43.3%						

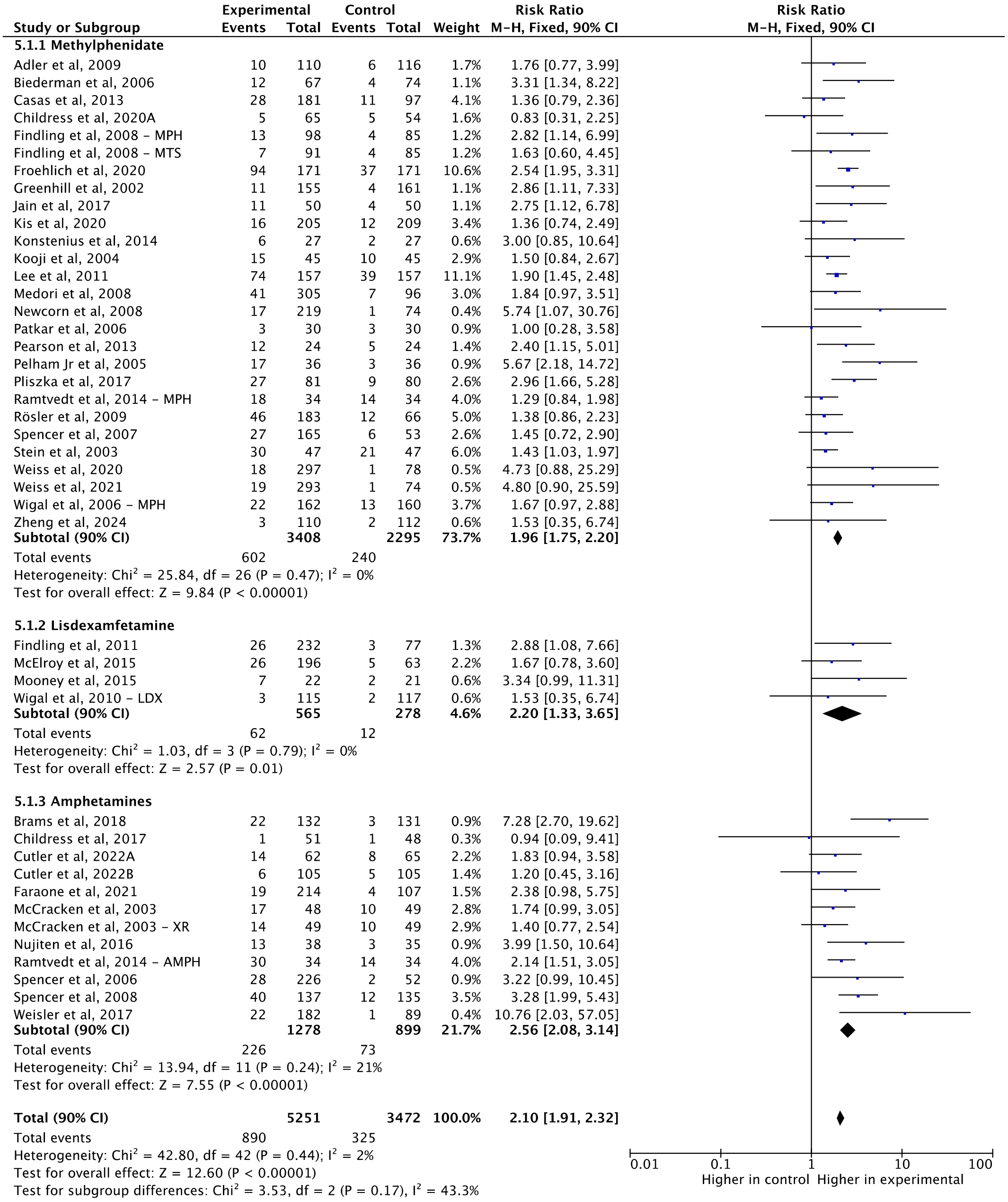

Figure legend: 90%CI, 90% confidence interval; AMPH, amphetamine; LDX, lisdexanfetamine; M-H, Mantel–Haenszel; MPH, methylphenidate; MTS, transdermal system; XR, extended-release.

eFigure 5a. Forest Plot Showing the Risk Ratio of **Dry Mouth** Between Control and Experimental Groups (95% confidence interval).

Study or Subgroup	Experimental Events	Experimental Total	Control Events	Control Total	Weight	Risk Ratio M-H, Random, 95% CI
**8.1.1 Methylphenidate**						
Adler et al, 2009	22	110	6	116	5.6%	3.87 [1.63, 9.18]
Biederman et al, 2006	23	67	5	74	5.3%	5.08 [2.05, 12.61]
Casas et al, 2013	32	181	3	97	4.0%	5.72 [1.80, 18.19]
Ginsberg et al, 2014	15	216	9	82	6.1%	0.63 [0.29, 1.39]
Goodman et al, 2017	27	174	3	175	3.9%	9.05 [2.80, 29.29]
Huss et al, 2014	110	542	4	180	4.8%	9.13 [3.42, 24.41]
Jain et al, 2017	6	50	1	50	1.6%	6.00 [0.75, 48.05]
Kis et al, 2020	30	205	10	209	6.9%	3.06 [1.54, 6.09]
Konstenius et al, 2014	2	27	1	27	1.3%	2.00 [0.19, 20.77]
Kooji et al, 2004	11	45	3	45	3.7%	3.67 [1.10, 12.27]
Medori et al, 2008	36	305	2	96	3.0%	5.67 [1.39, 23.10]
Retz et al, 2012	32	84	11	78	7.5%	2.70 [1.47, 4.98]
Rösler et al, 2009	55	183	10	66	7.5%	1.98 [1.08, 3.66]
Spencer et al, 2007	26	165	2	53	3.0%	4.18 [1.03, 17.01]
Weiss et al, 2020	27	297	3	78	3.9%	2.36 [0.74, 7.59]
**Subtotal (95% CI)**		**2651**		**1426**	**68.1%**	**3.37 [2.29, 4.98]**
Total events	454		73			
Heterogeneity: $Tau^2 = 0.29$; $Chi^2 = 31.33$, df = 14 (P = 0.005); $I^2 = 55\%$						
Test for overall effect: Z = 6.12 (P < 0.00001)						
**8.1.2 Lisdexamfetamine**						
Findling et al, 2011	10	232	1	77	1.7%	3.32 [0.43, 25.51]
Martin et al, 2014B - LDX	6	18	3	17	3.7%	1.89 [0.56, 6.38]
McElroy et al, 2015	71	196	5	63	5.6%	4.56 [1.93, 10.80]
Wigal et al, 2010 - LDX	4	115	1	117	1.5%	4.07 [0.46, 35.86]
**Subtotal (95% CI)**		**561**		**274**	**12.4%**	**3.44 [1.82, 6.50]**
Total events	91		10			
Heterogeneity: $Tau^2 = 0.00$; $Chi^2 = 1.44$, df = 3 (P = 0.70); $I^2 = 0\%$						
Test for overall effect: Z = 3.81 (P = 0.0001)						
**8.1.3 Amphetamines**						
Cutler et al, 2022A	12	62	1	65	1.7%	12.58 [1.69, 93.90]
Faraone et al, 2021	18	214	4	107	4.4%	2.25 [0.78, 6.48]
Martin et al, 2014B - AMPH	4	17	3	17	3.2%	1.33 [0.35, 5.08]
Spencer et al, 2008	31	137	7	135	6.1%	4.36 [1.99, 9.57]
Weisler et al, 2017	33	182	3	89	4.0%	5.38 [1.70, 17.06]
**Subtotal (95% CI)**		**612**		**413**	**19.5%**	**3.53 [1.96, 6.36]**
Total events	98		18			
Heterogeneity: $Tau^2 = 0.11$; $Chi^2 = 5.24$, df = 4 (P = 0.26); $I^2 = 24\%$						
Test for overall effect: Z = 4.19 (P < 0.0001)						
**Total (95% CI)**		**3824**		**2113**	**100.0%**	**3.34 [2.52, 4.44]**
Total events	643		101			
Heterogeneity: $Tau^2 = 0.18$; $Chi^2 = 38.40$, df = 23 (P = 0.02); $I^2 = 40\%$						
Test for overall effect: Z = 8.33 (P < 0.00001)						
Test for subgroup differences: $Chi^2 = 0.02$, df = 2 (P = 0.99), $I^2 = 0\%$						

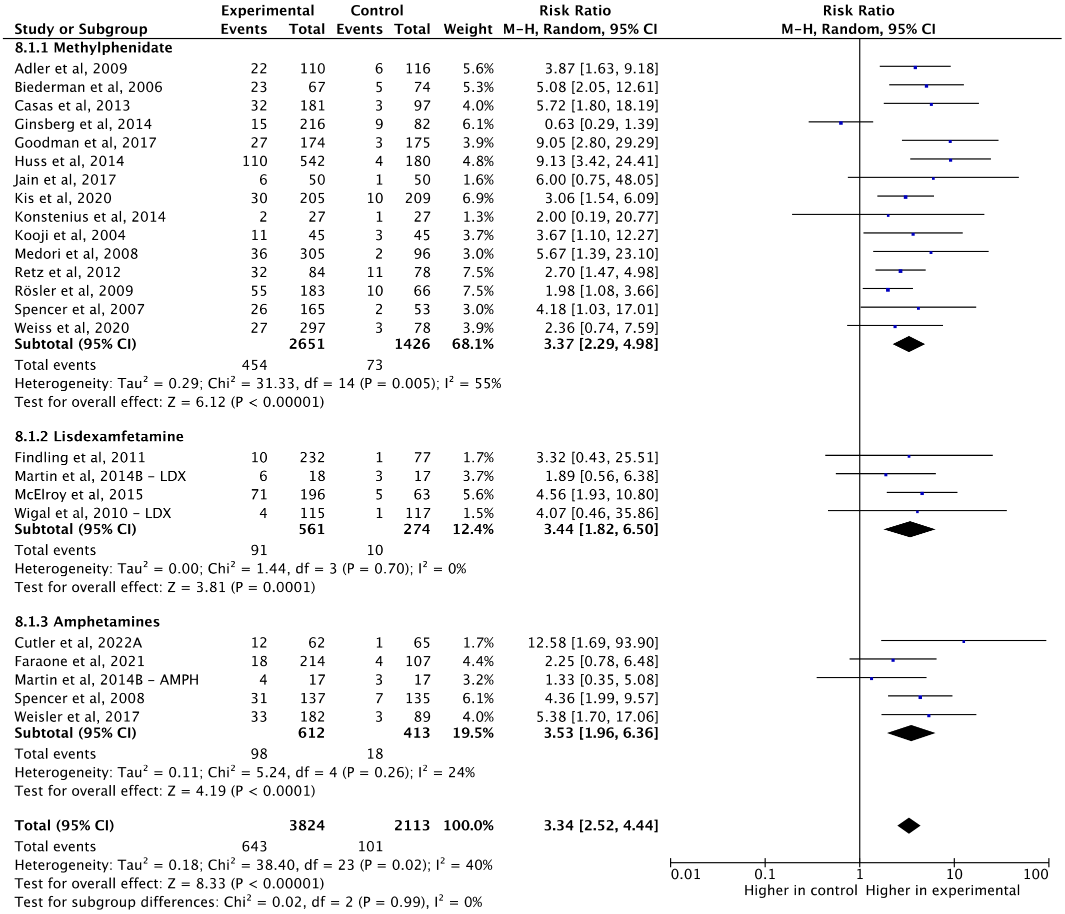

Figure legend: 95%CI, 95% confidence interval; AMPH, amphetamine; LDX, lisdexanfetamine; M-H, Mantel–Haenszel.

eFigure 5b. Forest Plot Showing the Risk Ratio of **Dry Mouth** Between Control and Experimental Groups (90% confidence interval).

Study or Subgroup	Experimental Events	Experimental Total	Control Events	Control Total	Weight	Risk Ratio M-H, Random, 90% CI
**8.1.1 Methylphenidate**						
Adler et al, 2009	22	110	6	116	5.6%	3.87 [1.87, 7.99]
Biederman et al, 2006	23	67	5	74	5.3%	5.08 [2.37, 10.89]
Casas et al, 2013	32	181	3	97	4.0%	5.72 [2.16, 15.10]
Ginsberg et al, 2014	15	216	9	82	6.1%	0.63 [0.33, 1.22]
Goodman et al, 2017	27	174	3	175	3.9%	9.05 [3.38, 24.25]
Huss et al, 2014	110	542	4	180	4.8%	9.13 [4.00, 20.84]
Jain et al, 2017	6	50	1	50	1.6%	6.00 [1.05, 34.39]
Kis et al, 2020	30	205	10	209	6.9%	3.06 [1.72, 5.45]
Konstenius et al, 2014	2	27	1	27	1.3%	2.00 [0.28, 14.26]
Kooji et al, 2004	11	45	3	45	3.7%	3.67 [1.33, 10.10]
Medori et al, 2008	36	305	2	96	3.0%	5.67 [1.74, 18.43]
Retz et al, 2012	32	84	11	78	7.5%	2.70 [1.62, 4.51]
Rösler et al, 2009	55	183	10	66	7.5%	1.98 [1.19, 3.32]
Spencer et al, 2007	26	165	2	53	3.0%	4.18 [1.28, 13.57]
Weiss et al, 2020	27	297	3	78	3.9%	2.36 [0.89, 6.29]
**Subtotal (90% CI)**		**2651**		**1426**	**68.1%**	**3.37 [2.43, 4.67]**
Total events	454		73			
Heterogeneity: $Tau^2 = 0.29$; $Chi^2 = 31.33$, df = 14 (P = 0.005); $I^2 = 55\%$						
Test for overall effect: Z = 6.12 (P < 0.00001)						
**8.1.2 Lisdexamfetamine**						
Findling et al, 2011	10	232	1	77	1.7%	3.32 [0.60, 18.38]
Martin et al, 2014B - LDX	6	18	3	17	3.7%	1.89 [0.68, 5.25]
McElroy et al, 2015	71	196	5	63	5.6%	4.56 [2.22, 9.40]
Wigal et al, 2010 - LDX	4	115	1	117	1.5%	4.07 [0.66, 25.27]
**Subtotal (90% CI)**		**561**		**274**	**12.4%**	**3.44 [2.02, 5.87]**
Total events	91		10			
Heterogeneity: $Tau^2 = 0.00$; $Chi^2 = 1.44$, df = 3 (P = 0.70); $I^2 = 0\%$						
Test for overall effect: Z = 3.81 (P = 0.0001)						
**8.1.3 Amphetamines**						
Cutler et al, 2022A	12	62	1	65	1.7%	12.58 [2.33, 67.97]
Faraone et al, 2021	18	214	4	107	4.4%	2.25 [0.93, 5.47]
Martin et al, 2014B - AMPH	4	17	3	17	3.2%	1.33 [0.43, 4.10]
Spencer et al, 2008	31	137	7	135	6.1%	4.36 [2.26, 8.43]
Weisler et al, 2017	33	182	3	89	4.0%	5.38 [2.04, 14.17]
**Subtotal (90% CI)**		**612**		**413**	**19.5%**	**3.53 [2.15, 5.78]**
Total events	98		18			
Heterogeneity: $Tau^2 = 0.11$; $Chi^2 = 5.24$, df = 4 (P = 0.26); $I^2 = 24\%$						
Test for overall effect: Z = 4.19 (P < 0.0001)						
**Total (90% CI)**		**3824**		**2113**	**100.0%**	**3.34 [2.64, 4.24]**
Total events	643		101			
Heterogeneity: $Tau^2 = 0.18$; $Chi^2 = 38.40$, df = 23 (P = 0.02); $I^2 = 40\%$						
Test for overall effect: Z = 8.33 (P < 0.00001)						
Test for subgroup differences: $Chi^2 = 0.02$, df = 2 (P = 0.99), $I^2 = 0\%$						

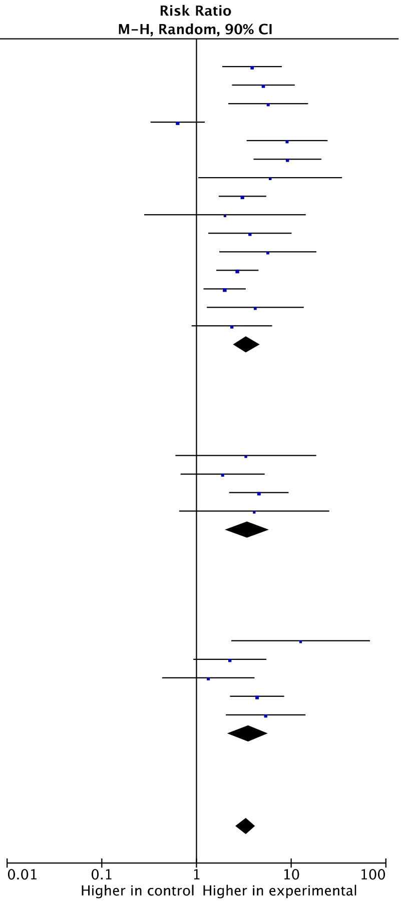

Figure legend: 90%CI, 90% confidence interval; AMPH, amphetamine; LDX, lisdexanfetamine; M-H, Mantel–Haenszel.

eFigure 6a. Forest Plot Showing the Risk Ratio of **Nausea** Between Control and Experimental Groups (95% confidence interval).

Study or Subgroup	Experimental Events	Experimental Total	Control Events	Control Total	Weight	Risk Ratio M-H, Fixed, 95% CI
**7.1.1 Methylphenidate**						
Adler et al, 2009	14	110	3	116	2.2%	4.92 [1.45, 16.66]
Casas et al, 2013	31	181	8	97	7.7%	2.08 [0.99, 4.34]
Coghill et al, 2013 - MPH	8	111	3	110	2.2%	2.64 [0.72, 9.70]
Findling et al, 2008 - MPH	12	198	4	85	4.1%	1.29 [0.43, 3.88]
Findling et al, 2008 - MTS	7	91	4	85	3.1%	1.63 [0.50, 5.39]
Huss et al, 2014	58	542	9	180	10.0%	2.14 [1.08, 4.23]
Jain et al, 2017	8	50	4	50	3.0%	2.00 [0.64, 6.22]
Kis et al, 2020	25	205	20	209	14.6%	1.27 [0.73, 2.22]
Medori et al, 2008	39	305	4	96	4.5%	3.07 [1.13, 8.37]
Newcorn et al, 2008	13	219	6	74	6.6%	0.73 [0.29, 1.86]
Patkar et al, 2006	2	30	1	30	0.7%	2.00 [0.19, 20.90]
Pearson et al, 2013	1	24	1	24	0.7%	1.00 [0.07, 15.08]
Ramtvedt et al, 2014 - MPH	10	34	6	34	4.4%	1.67 [0.68, 4.07]
Retz et al, 2012	14	84	3	78	2.3%	4.33 [1.29, 14.51]
Rosenberg et al, 2014	3	29	7	29	5.2%	0.43 [0.12, 1.50]
Rösler et al, 2009	16	183	2	66	2.2%	2.89 [0.68, 12.21]
Weiss et al, 2020	18	297	2	78	2.3%	2.36 [0.56, 9.97]
Weiss et al, 2021	17	293	3	74	3.5%	1.43 [0.43, 4.75]
Wilens et al, 2006	1	87	2	90	1.5%	0.52 [0.05, 5.60]
Zheng et al, 2024	12	110	1	112	0.7%	12.22 [1.62, 92.38]
**Subtotal (95% CI)**		**3183**		**1717**	**81.6%**	**1.91 [1.52, 2.40]**
Total events	309		93			
Heterogeneity: Chi² = 22.81, df = 19 (P = 0.25); I² = 17%						
Test for overall effect: Z = 5.52 (P < 0.00001)						
**7.1.2 Lisdexamfetamine**						
Coghill et al, 2013 - LDX	12	111	3	110	2.2%	3.96 [1.15, 13.66]
Findling et al, 2011	9	232	2	77	2.2%	1.49 [0.33, 6.76]
McElroy et al, 2015	15	196	0	63	0.6%	10.07 [0.61, 165.95]
Mooney et al, 2015	5	22	1	21	0.8%	4.77 [0.61, 37.52]
**Subtotal (95% CI)**		**561**		**271**	**5.8%**	**3.71 [1.63, 8.44]**
Total events	41		6			
Heterogeneity: Chi² = 1.95, df = 3 (P = 0.58); I² = 0%						
Test for overall effect: Z = 3.13 (P = 0.002)						
**7.1.3 Amphetamines**						
Brams et al, 2018	9	132	4	131	3.0%	2.23 [0.71, 7.07]
Cutler et al, 2022B	3	105	1	105	0.7%	3.00 [0.32, 28.38]
Ramtvedt et al, 2014 - AMPH	10	34	6	34	4.4%	1.67 [0.68, 4.07]
Spencer et al, 2008	10	137	6	135	4.5%	1.64 [0.61, 4.39]
**Subtotal (95% CI)**		**408**		**405**	**12.6%**	**1.87 [1.07, 3.26]**
Total events	32		17			
Heterogeneity: Chi² = 0.39, df = 3 (P = 0.94); I² = 0%						
Test for overall effect: Z = 2.20 (P = 0.03)						
**Total (95% CI)**		**4152**		**2393**	**100.0%**	**2.01 [1.64, 2.46]**
Total events	382		116			
Heterogeneity: Chi² = 27.28, df = 27 (P = 0.45); I² = 1%						
Test for overall effect: Z = 6.66 (P < 0.00001)						
Test for subgroup differences: Chi² = 2.38, df = 2 (P = 0.30), I² = 16.0%						

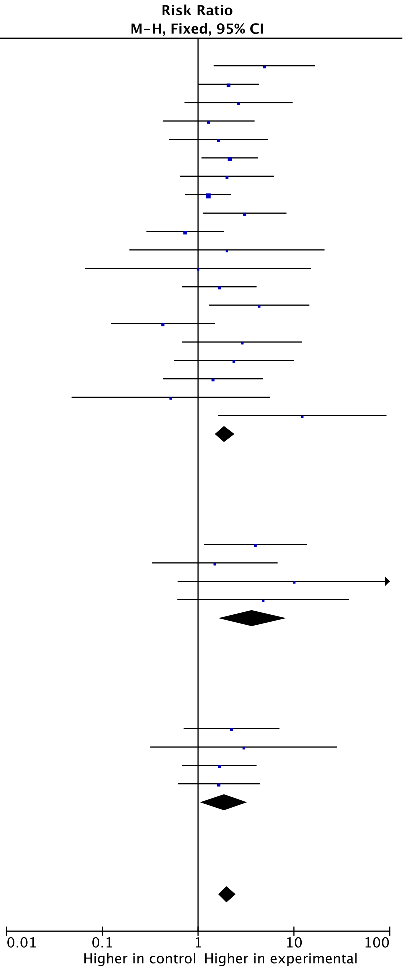

Figure legend: 95%CI, 95% confidence interval; AMPH, amphetamine; LDX, lisdexanfetamine; M-H, Mantel–Haenszel; MPH, methylphenidate; MTS, transdermal system.

eFigure 6b. Forest Plot Showing the Risk Ratio of **Nausea** Between Control and Experimental Groups (90% confidence interval).

	Experimental		Control			Risk Ratio
Study or Subgroup	Events	Total	Events	Total	Weight	M-H, Fixed, 90% CI
**7.1.1 Methylphenidate**						
Adler et al, 2009	14	110	3	116	2.2%	4.92 [1.77, 13.69]
Casas et al, 2013	31	181	8	97	7.7%	2.08 [1.12, 3.85]
Coghill et al, 2013 - MPH	8	111	3	110	2.2%	2.64 [0.89, 7.87]
Findling et al, 2008 - MPH	12	198	4	85	4.1%	1.29 [0.51, 3.25]
Findling et al, 2008 - MTS	7	91	4	85	3.1%	1.63 [0.60, 4.45]
Huss et al, 2014	58	542	9	180	10.0%	2.14 [1.21, 3.79]
Jain et al, 2017	8	50	4	50	3.0%	2.00 [0.77, 5.18]
Kis et al, 2020	25	205	20	209	14.6%	1.27 [0.80, 2.03]
Medori et al, 2008	39	305	4	96	4.5%	3.07 [1.32, 7.12]
Newcorn et al, 2008	13	219	6	74	6.6%	0.73 [0.34, 1.60]
Patkar et al, 2006	2	30	1	30	0.7%	2.00 [0.28, 14.33]
Pearson et al, 2013	1	24	1	24	0.7%	1.00 [0.10, 9.75]
Ramtvedt et al, 2014 - MPH	10	34	6	34	4.4%	1.67 [0.79, 3.53]
Retz et al, 2012	14	84	3	78	2.3%	4.33 [1.57, 11.95]
Rosenberg et al, 2014	3	29	7	29	5.2%	0.43 [0.15, 1.22]
Rösler et al, 2009	16	183	2	66	2.2%	2.89 [0.86, 9.68]
Weiss et al, 2020	18	297	2	78	2.3%	2.36 [0.71, 7.91]
Weiss et al, 2021	17	293	3	74	3.5%	1.43 [0.52, 3.92]
Wilens et al, 2006	1	87	2	90	1.5%	0.52 [0.07, 3.82]
Zheng et al, 2024	12	110	1	112	0.7%	12.22 [2.24, 66.73]
**Subtotal (90% CI)**		**3183**		**1717**	**81.6%**	**1.91 [1.57, 2.31]**
Total events	309		93			
Heterogeneity: Chi² = 22.81, df = 19 (P = 0.25); I² = 17%						
Test for overall effect: Z = 5.52 (P < 0.00001)						
**7.1.2 Lisdexamfetamine**						
Coghill et al, 2013 - LDX	12	111	3	110	2.2%	3.96 [1.40, 11.20]
Findling et al, 2011	9	232	2	77	2.2%	1.49 [0.42, 5.31]
McElroy et al, 2015	15	196	0	63	0.6%	10.07 [0.96, 105.76]
Mooney et al, 2015	5	22	1	21	0.8%	4.77 [0.85, 26.94]
**Subtotal (90% CI)**		**561**		**271**	**5.8%**	**3.71 [1.86, 7.39]**
Total events	41		6			
Heterogeneity: Chi² = 1.95, df = 3 (P = 0.58); I² = 0%						
Test for overall effect: Z = 3.13 (P = 0.002)						
**7.1.3 Amphetamines**						
Brams et al, 2018	9	132	4	131	3.0%	2.23 [0.85, 5.88]
Cutler et al, 2022B	3	105	1	105	0.7%	3.00 [0.46, 19.77]
Ramtvedt et al, 2014 - AMPH	10	34	6	34	4.4%	1.67 [0.79, 3.53]
Spencer et al, 2008	10	137	6	135	4.5%	1.64 [0.72, 3.75]
**Subtotal (90% CI)**		**408**		**405**	**12.6%**	**1.87 [1.17, 2.98]**
Total events	32		17			
Heterogeneity: Chi² = 0.39, df = 3 (P = 0.94); I² = 0%						
Test for overall effect: Z = 2.20 (P = 0.03)						
**Total (90% CI)**		**4152**		**2393**	**100.0%**	**2.01 [1.69, 2.38]**
Total events	382		116			
Heterogeneity: Chi² = 27.28, df = 27 (P = 0.45); I² = 1%						
Test for overall effect: Z = 6.66 (P < 0.00001)						
Test for subgroup differences: Chi² = 2.38, df = 2 (P = 0.30), I² = 16.0%						

Risk Ratio
M-H, Fixed, 90% CI

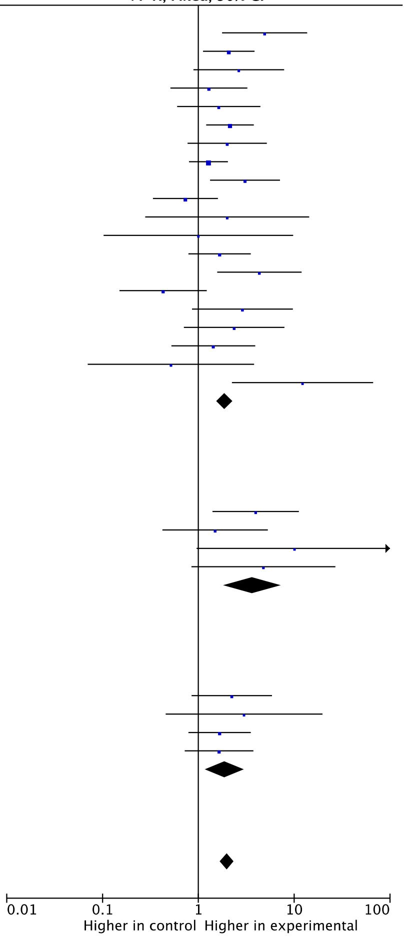

Figure legend: 90%CI, 90% confidence interval; AMPH, amphetamine; LDX, lisdexanfetamine; M-H, Mantel–Haenszel; MPH, methylphenidate; MTS, transdermal system.

eFigure 7a. Forest Plot Showing the Risk Ratio of **Irritability** Between Control and Experimental Groups (95% confidence interval).

Study or Subgroup	Experimental Events	Experimental Total	Control Events	Control Total	Weight	Risk Ratio M-H, Fixed, 95% CI
**9.1.1 Methylphenidate**						
Adler et al, 2009	7	110	2	116	0.5%	3.69 [0.78, 17.38]
Chronis-Tuscano et al, 2008	3	15	1	23	0.2%	4.60 [0.53, 40.19]
Froehlich et al, 2020	77	171	67	171	17.7%	1.15 [0.90, 1.48]
Jain et al, 2017	10	50	2	50	0.5%	5.00 [1.15, 21.67]
Kis et al, 2020	14	205	11	209	2.9%	1.30 [0.60, 2.79]
Lee et al, 2011	84	157	81	157	21.4%	1.04 [0.84, 1.28]
Medori et al, 2008	17	305	1	96	0.4%	5.35 [0.72, 39.68]
Pearson et al, 2013	8	24	11	24	2.9%	0.73 [0.36, 1.48]
Pelham Jr et al, 2005	10	36	1	36	0.3%	10.00 [1.35, 74.12]
Ramtvedt et al, 2014 - MPH	22	34	27	34	7.1%	0.81 [0.60, 1.10]
Stein et al, 2003	33	47	27	47	7.1%	1.22 [0.90, 1.66]
Sugaya et al, 2022	22	51	19	51	5.0%	1.16 [0.72, 1.86]
Weiss et al, 2020	16	297	4	78	1.7%	1.05 [0.36, 3.05]
Weiss et al, 2021	24	293	7	74	3.0%	0.87 [0.39, 1.93]
**Subtotal (95% CI)**		**1795**		**1166**	**70.8%**	**1.18 [1.04, 1.33]**
Total events	347		261			
Heterogeneity: $Chi^2 = 23.60$, df = 13 (P = 0.04); $I^2 = 45\%$						
Test for overall effect: Z = 2.61 (P = 0.009)						
**9.1.2 Lisdexamfetamine**						
Findling et al, 2011	16	232	3	77	1.2%	1.77 [0.53, 5.91]
McElroy et al, 2015	11	196	4	63	1.6%	0.88 [0.29, 2.68]
**Subtotal (95% CI)**		**428**		**140**	**2.8%**	**1.26 [0.56, 2.83]**
Total events	27		7			
Heterogeneity: $Chi^2 = 0.70$, df = 1 (P = 0.40); $I^2 = 0\%$						
Test for overall effect: Z = 0.56 (P = 0.57)						
**9.1.3 Amphetamines**						
Brams et al, 2018	9	132	2	131	0.5%	4.47 [0.98, 20.28]
Cutler et al, 2022A	11	62	5	65	1.3%	2.31 [0.85, 6.26]
Cutler et al, 2022B	2	105	1	105	0.3%	2.00 [0.18, 21.72]
McCracken et al, 2003	22	48	29	49	7.6%	0.77 [0.53, 1.14]
McCracken et al, 2003 - XR	21	49	29	49	7.7%	0.72 [0.49, 1.08]
Nujiten et al, 2016	6	38	2	35	0.6%	2.76 [0.60, 12.80]
Ramtvedt et al, 2014 - AMPH	23	34	27	34	7.1%	0.85 [0.64, 1.14]
Spencer et al, 2008	13	137	5	135	1.3%	2.56 [0.94, 6.99]
**Subtotal (95% CI)**		**605**		**603**	**26.4%**	**1.07 [0.88, 1.32]**
Total events	107		100			
Heterogeneity: $Chi^2 = 19.27$, df = 7 (P = 0.007); $I^2 = 64\%$						
Test for overall effect: Z = 0.69 (P = 0.49)						
**Total (95% CI)**		**2828**		**1909**	**100.0%**	**1.15 [1.04, 1.28]**
Total events	481		368			
Heterogeneity: $Chi^2 = 45.81$, df = 23 (P = 0.003); $I^2 = 50\%$						
Test for overall effect: Z = 2.66 (P = 0.008)						
Test for subgroup differences: $Chi^2 = 0.63$, df = 2 (P = 0.73), $I^2 = 0\%$						

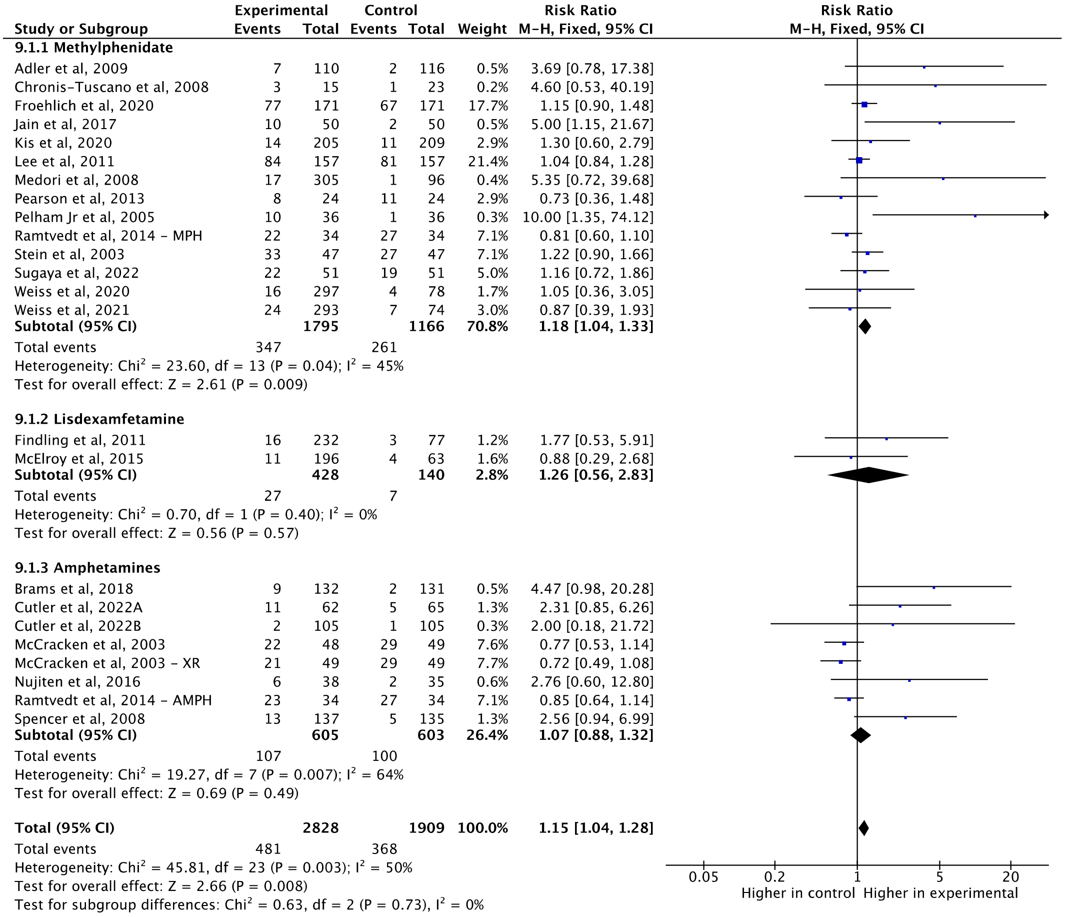

Figure legend: 95%CI, 95% confidence interval; AMPH, amphetamine; M-H, Mantel–Haenszel; MPH, methylphenidate; MTS, transdermal system; XR, extended-release.

eFigure 7b. Forest Plot Showing the Risk Ratio of **Irritability** Between Control and Experimental Groups (90% confidence interval).

Study or Subgroup	Experimental Events	Experimental Total	Control Events	Control Total	Weight	Risk Ratio M-H, Fixed, 90% CI
**9.1.1 Methylphenidate**						
Adler et al, 2009	7	110	2	116	0.5%	3.69 [1.01, 13.55]
Chronis-Tuscano et al, 2008	3	15	1	23	0.2%	4.60 [0.75, 28.37]
Froehlich et al, 2020	77	171	67	171	17.7%	1.15 [0.93, 1.42]
Jain et al, 2017	10	50	2	50	0.5%	5.00 [1.46, 17.12]
Kis et al, 2020	14	205	11	209	2.9%	1.30 [0.68, 2.47]
Lee et al, 2011	84	157	81	157	21.4%	1.04 [0.87, 1.24]
Medori et al, 2008	17	305	1	96	0.4%	5.35 [1.00, 28.75]
Pearson et al, 2013	8	24	11	24	2.9%	0.73 [0.40, 1.32]
Pelham Jr et al, 2005	10	36	1	36	0.3%	10.00 [1.86, 53.71]
Ramtvedt et al, 2014 - MPH	22	34	27	34	7.1%	0.81 [0.63, 1.05]
Stein et al, 2003	33	47	27	47	7.1%	1.22 [0.94, 1.58]
Sugaya et al, 2022	22	51	19	51	5.0%	1.16 [0.78, 1.73]
Weiss et al, 2020	16	297	4	78	1.7%	1.05 [0.43, 2.57]
Weiss et al, 2021	24	293	7	74	3.0%	0.87 [0.44, 1.70]
**Subtotal (90% CI)**		**1795**		**1166**	**70.8%**	**1.18 [1.06, 1.31]**
Total events	347		261			
Heterogeneity: Chi² = 23.60, df = 13 (P = 0.04); I² = 45%						
Test for overall effect: Z = 2.61 (P = 0.009)						
**9.1.2 Lisdexamfetamine**						
Findling et al, 2011	16	232	3	77	1.2%	1.77 [0.64, 4.87]
McElroy et al, 2015	11	196	4	63	1.6%	0.88 [0.35, 2.24]
**Subtotal (90% CI)**		**428**		**140**	**2.8%**	**1.26 [0.64, 2.49]**
Total events	27		7			
Heterogeneity: Chi² = 0.70, df = 1 (P = 0.40); I² = 0%						
Test for overall effect: Z = 0.56 (P = 0.57)						
**9.1.3 Amphetamines**						
Brams et al, 2018	9	132	2	131	0.5%	4.47 [1.25, 15.90]
Cutler et al, 2022A	11	62	5	65	1.3%	2.31 [1.00, 5.33]
Cutler et al, 2022B	2	105	1	105	0.3%	2.00 [0.27, 14.80]
McCracken et al, 2003	22	48	29	49	7.6%	0.77 [0.56, 1.07]
McCracken et al, 2003 - XR	21	49	29	49	7.7%	0.72 [0.52, 1.01]
Nujiten et al, 2016	6	38	2	35	0.6%	2.76 [0.76, 10.00]
Ramtvedt et al, 2014 - AMPH	23	34	27	34	7.1%	0.85 [0.67, 1.09]
Spencer et al, 2008	13	137	5	135	1.3%	2.56 [1.10, 5.95]
**Subtotal (90% CI)**		**605**		**603**	**26.4%**	**1.07 [0.91, 1.27]**
Total events	107		100			
Heterogeneity: Chi² = 19.27, df = 7 (P = 0.007); I² = 64%						
Test for overall effect: Z = 0.69 (P = 0.49)						
**Total (90% CI)**		**2828**		**1909**	**100.0%**	**1.15 [1.06, 1.26]**
Total events	481		368			
Heterogeneity: Chi² = 45.81, df = 23 (P = 0.003); I² = 50%						
Test for overall effect: Z = 2.66 (P = 0.008)						
Test for subgroup differences: Chi² = 0.63, df = 2 (P = 0.73), I² = 0%						

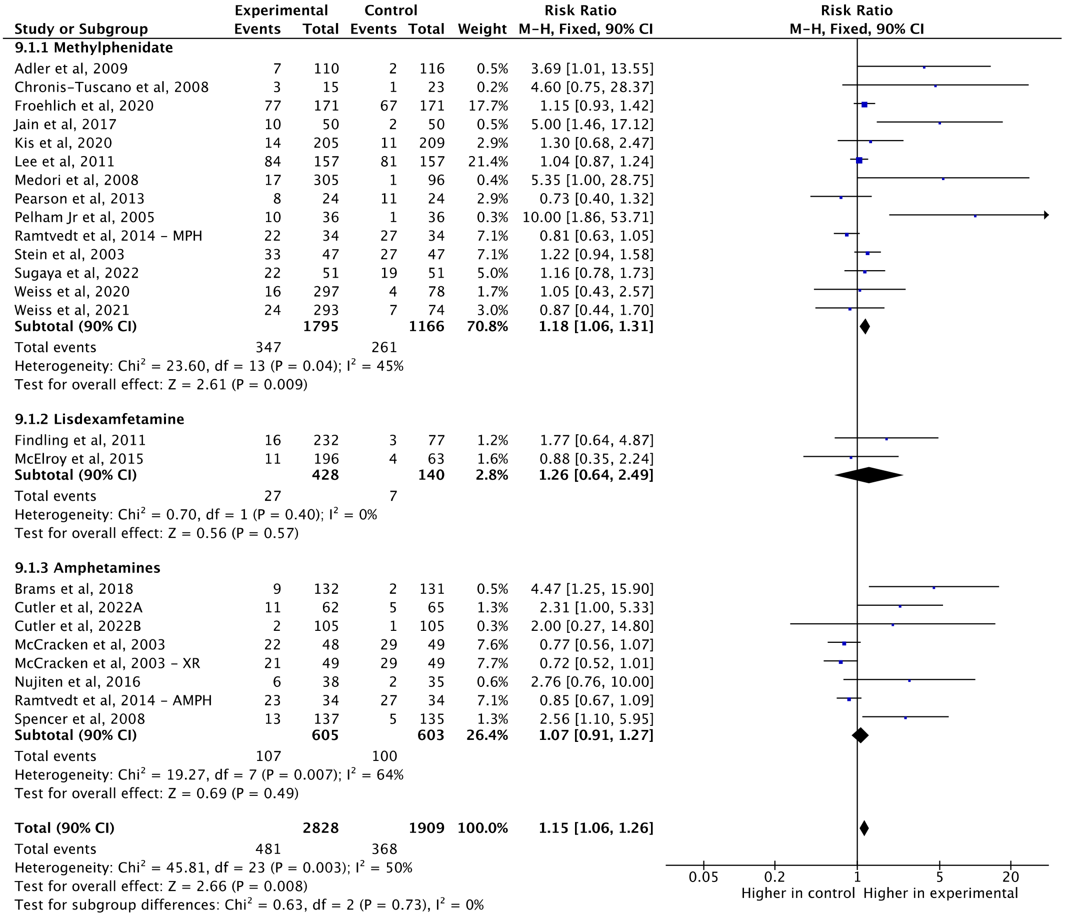

Figure legend: 90%CI, 90% confidence interval; AMPH, amphetamine; M-H, Mantel–Haenszel; MPH, methylphenidate; MTS, transdermal system; XR, extended-release.

eFigure 8a. Forest Plot Showing the Risk Ratio of **Anxiety** Between Control and Experimental Groups (95% confidence interval).

Study or Subgroup	Experimental Events	Experimental Total	Control Events	Control Total	Weight	Risk Ratio M-H, Fixed, 95% CI
**6.1.1 Methylphenidate**						
Adler et al, 2009	18	110	4	116	2.0%	4.75 [1.66, 13.58]
Froehlich et al, 2020	32	171	26	171	13.2%	1.23 [0.77, 1.97]
Konstenius et al, 2014	1	27	4	27	2.0%	0.25 [0.03, 2.09]
Lee et al, 2011	53	157	58	157	29.6%	0.91 [0.68, 1.23]
Medori et al, 2008	16	305	1	96	0.8%	5.04 [0.68, 37.48]
Patkar et al, 2006	2	30	1	30	0.5%	2.00 [0.19, 20.90]
Pearson et al, 2013	6	24	4	24	2.0%	1.50 [0.48, 4.65]
Ramtvedt et al, 2014 - MPH	10	34	7	34	3.6%	1.43 [0.62, 3.31]
Rosenberg et al, 2014	17	29	10	29	5.1%	1.70 [0.94, 3.06]
Stein et al, 2003	22	47	24	47	12.2%	0.92 [0.61, 1.39]
Sugaya et al, 2022	18	51	16	51	8.2%	1.13 [0.65, 1.95]
**Subtotal (95% CI)**		**985**		**782**	**79.2%**	**1.20 [1.01, 1.44]**
Total events	195		155			
Heterogeneity: Chi² = 17.39, df = 10 (P = 0.07); I² = 42%						
Test for overall effect: Z = 2.03 (P = 0.04)						
**6.1.2 Lisdexamfetamine**						
McElroy et al, 2015	9	196	5	63	3.9%	0.58 [0.20, 1.66]
Mooney et al, 2015	7	22	1	21	0.5%	6.68 [0.90, 49.78]
**Subtotal (95% CI)**		**218**		**84**	**4.4%**	**1.31 [0.57, 2.97]**
Total events	16		6			
Heterogeneity: Chi² = 4.82, df = 1 (P = 0.03); I² = 79%						
Test for overall effect: Z = 0.64 (P = 0.53)						
**6.1.3 Amphetamines**						
McCracken et al, 2003	11	48	10	49	5.0%	1.12 [0.53, 2.40]
McCracken et al, 2003 - XR	9	49	10	49	5.1%	0.90 [0.40, 2.02]
Ramtvedt et al, 2014 - AMPH	8	34	7	34	3.6%	1.14 [0.47, 2.80]
Spencer et al, 2008	9	137	4	135	2.1%	2.22 [0.70, 7.03]
Weisler et al, 2017	10	182	1	89	0.7%	4.89 [0.64, 37.61]
**Subtotal (95% CI)**		**450**		**356**	**16.4%**	**1.35 [0.89, 2.06]**
Total events	47		32			
Heterogeneity: Chi² = 3.57, df = 4 (P = 0.47); I² = 0%						
Test for overall effect: Z = 1.40 (P = 0.16)						
**Total (95% CI)**		**1653**		**1222**	**100.0%**	**1.23 [1.05, 1.45]**
Total events	258		193			
Heterogeneity: Chi² = 25.93, df = 17 (P = 0.08); I² = 34%						
Test for overall effect: Z = 2.53 (P = 0.01)						
Test for subgroup differences: Chi² = 0.26, df = 2 (P = 0.88), I² = 0%						

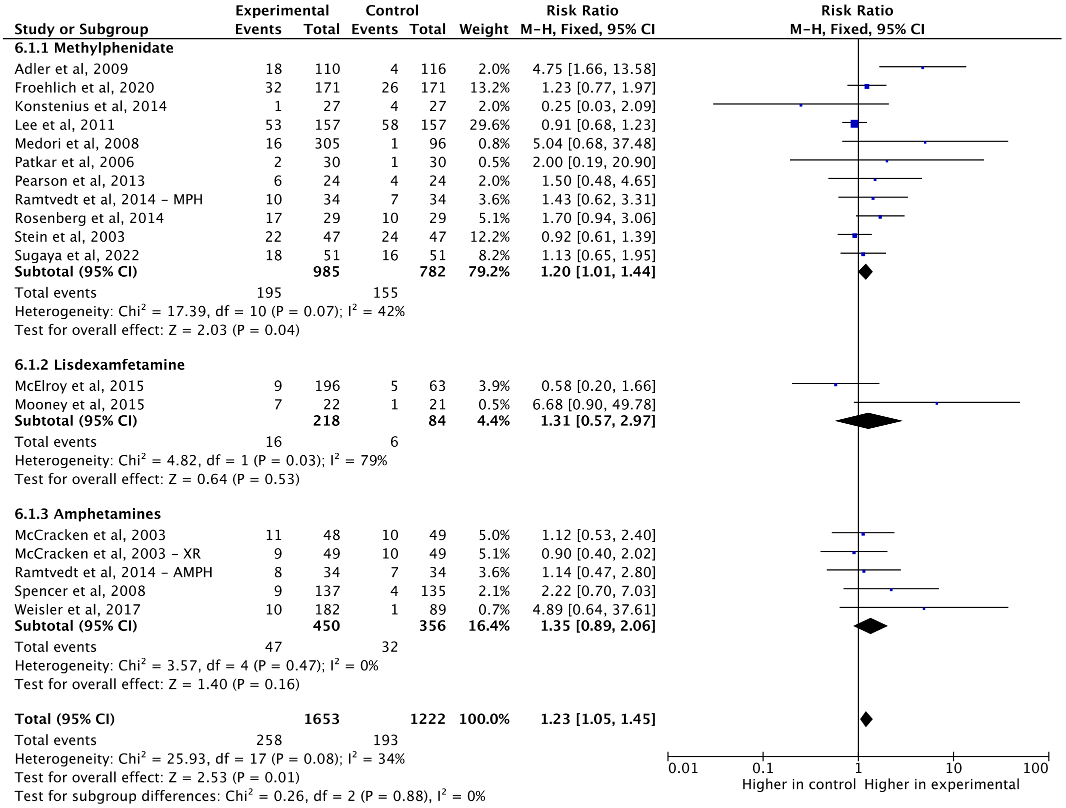

Figure legend: 95%CI, 95% confidence interval; AMPH, amphetamine; M-H, Mantel–Haenszel; MPH, methylphenidate; XR, extended-release.

eFigure 8b. Forest Plot Showing the Risk Ratio of **Anxiety** Between Control and Experimental Groups (90% confidence interval).

Study or Subgroup	Experimental Events	Experimental Total	Control Events	Control Total	Weight	Risk Ratio M-H, Fixed, 90% CI
**6.1.1 Methylphenidate**						
Adler et al, 2009	18	110	4	116	2.0%	4.75 [1.96, 11.47]
Froehlich et al, 2020	32	171	26	171	13.2%	1.23 [0.83, 1.83]
Konstenius et al, 2014	1	27	4	27	2.0%	0.25 [0.04, 1.49]
Lee et al, 2011	53	157	58	157	29.6%	0.91 [0.71, 1.18]
Medori et al, 2008	16	305	1	96	0.8%	5.04 [0.93, 27.14]
Patkar et al, 2006	2	30	1	30	0.5%	2.00 [0.28, 14.33]
Pearson et al, 2013	6	24	4	24	2.0%	1.50 [0.58, 3.88]
Ramtvedt et al, 2014 - MPH	10	34	7	34	3.6%	1.43 [0.71, 2.89]
Rosenberg et al, 2014	17	29	10	29	5.1%	1.70 [1.04, 2.78]
Stein et al, 2003	22	47	24	47	12.2%	0.92 [0.65, 1.30]
Sugaya et al, 2022	18	51	16	51	8.2%	1.13 [0.71, 1.79]
**Subtotal (90% CI)**		**985**		**782**	**79.2%**	**1.20 [1.04, 1.40]**
Total events	195		155			
Heterogeneity: Chi^2 = 17.39, df = 10 (P = 0.07); I^2 = 42%						
Test for overall effect: Z = 2.03 (P = 0.04)						
**6.1.2 Lisdexamfetamine**						
McElroy et al, 2015	9	196	5	63	3.9%	0.58 [0.24, 1.40]
Mooney et al, 2015	7	22	1	21	0.5%	6.68 [1.24, 36.04]
**Subtotal (90% CI)**		**218**		**84**	**4.4%**	**1.31 [0.65, 2.60]**
Total events	16		6			
Heterogeneity: Chi^2 = 4.82, df = 1 (P = 0.03); I^2 = 79%						
Test for overall effect: Z = 0.64 (P = 0.53)						
**6.1.3 Amphetamines**						
McCracken et al, 2003	11	48	10	49	5.0%	1.12 [0.59, 2.12]
McCracken et al, 2003 - XR	9	49	10	49	5.1%	0.90 [0.46, 1.77]
Ramtvedt et al, 2014 - AMPH	8	34	7	34	3.6%	1.14 [0.54, 2.42]
Spencer et al, 2008	9	137	4	135	2.1%	2.22 [0.84, 5.84]
Weisler et al, 2017	10	182	1	89	0.7%	4.89 [0.88, 27.09]
**Subtotal (90% CI)**		**450**		**356**	**16.4%**	**1.35 [0.95, 1.93]**
Total events	47		32			
Heterogeneity: Chi^2 = 3.57, df = 4 (P = 0.47); I^2 = 0%						
Test for overall effect: Z = 1.40 (P = 0.16)						
**Total (90% CI)**		**1653**		**1222**	**100.0%**	**1.23 [1.08, 1.41]**
Total events	258		193			
Heterogeneity: Chi^2 = 25.93, df = 17 (P = 0.08); I^2 = 34%						
Test for overall effect: Z = 2.53 (P = 0.01)						
Test for subgroup differences: Chi^2 = 0.26, df = 2 (P = 0.88), I^2 = 0%						

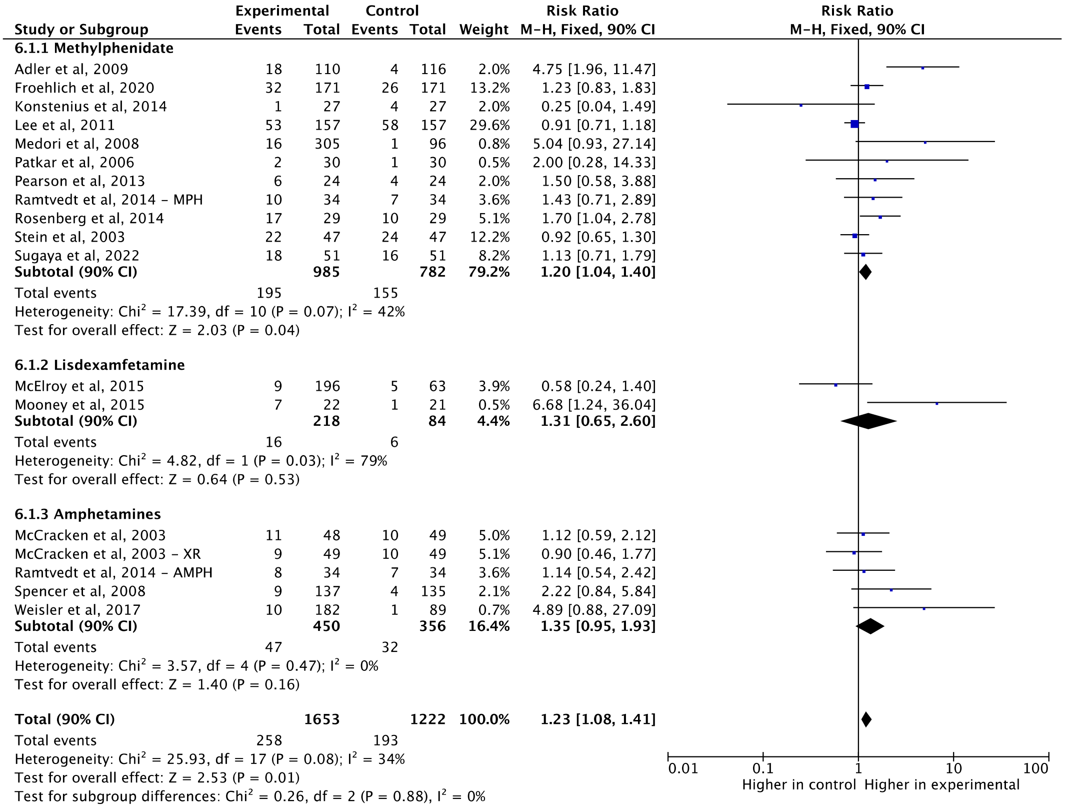

Figure legend: 90%CI, 90% confidence interval; AMPH, amphetamine; M-H, Mantel–Haenszel; MPH, methylphenidate; XR, extended-release.

eFigure 9. Risk of Bias Assessment

Risk of bias was assessed and is illustrated (eFigure 9a). Roughly half of the studies were considered to have an overall low risk of bias and the other half considered to have an unclear risk of bias. the main reason for the unclear results were in the selection bias domain, as the randomization process was not clearly described in some of the included studies. Performance bias was mostly low, with the only exceptions being the few open-label studies without blinding. Attrition bias was also mostly low, with studies clearly stating dropout rates and achieving their targeted number of participants. Detection bias was low, as the measurement of outcomes was reported and consistent between groups in most studies. Reporting bias was also low, as most studies reported on the planned outcomes with no significant selective outcome reporting.

a)

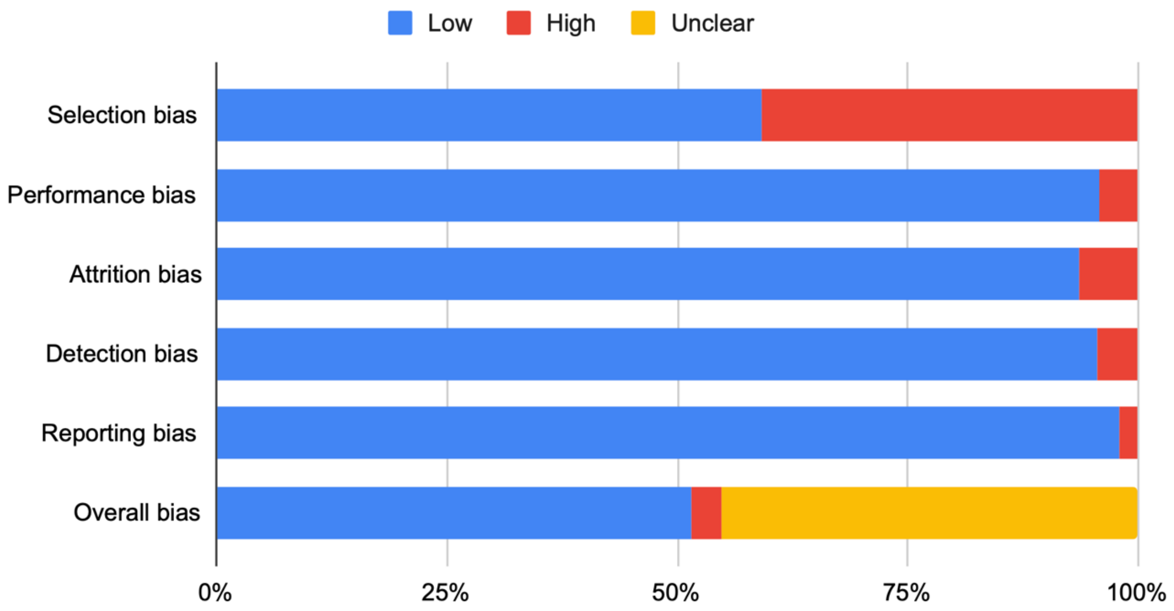

eFigure 9a. Methodological quality assessment of the included studies.

9b)

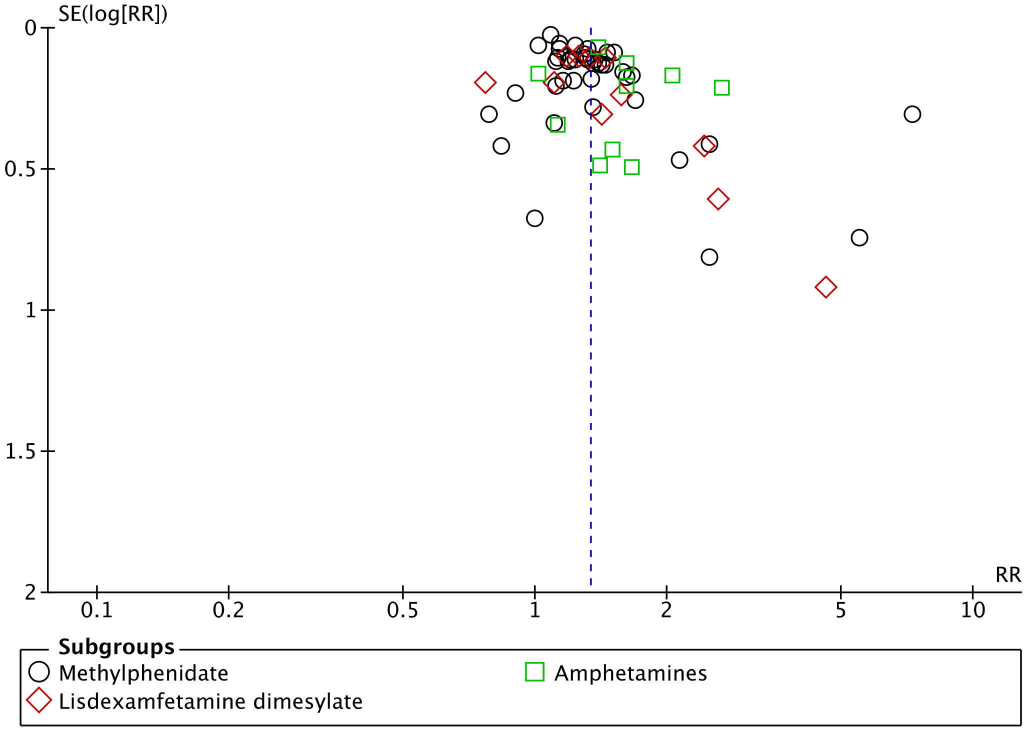

eFigure 9b. Funnel plot and statistic tests results for publication bias considering the overall adverse events.

Fail-Safe N Analysis (File Drawer Analysis)

Fail-safe N	p
5566.000	<.001

Note. Fail-safe N Calculation Using the Rosenthal Approach

Rank Correlation Test for Funnel Plot Asymmetry

Kendall's Tau	p
0.142	0.102

Regression Test for Funnel Plot Asymmetry

Z	p
4.040	<.001

9c)

the Bayesian model[94] used provides a multilevel analysis of the log-transformed Risk Ratios (logRRs) across number of observations from studies meta-analyzed for overall adverse events (AEs), with a random intercept for each study[95,96]. the analysis estimates the overall effect of the stimulants on the risk of AEs, accounting for variability across studies (eTable 4). Below we list the key findings from the model:

Intercept Estimate: the mean estimate for the treatment effect (logRR) is 0.38, with a 95% credible interval (CrI) ranging from 0.27 to 0.49. This suggests that, on average, the stimulants have a positive effect on the risk of adverse events, meaning that the risk is increased by 38% compared to the control group (in the original scale, this would be approximately exp(0.38) ≈ 1.46, or a 46% increase in risk). Since the entire CrI is above zero, we can infer that the stimulants are associated with an increase in risk.

Random Effects (Study-Level Variability): the standard deviation of the random intercepts for each study is 0.27 (95% CrI: 0.04 to 0.43). This indicates there is considerable variability in the treatment effects across different studies, suggesting that some studies report larger effects while others report smaller effects. This variability is accounted for by the random intercept structure, allowing for study-specific deviations from the overall mean effect.

Sigma (Residual Error): the standard deviation of the residuals (sigma) is 0.30 (95% CrI: 0.17 to 0.45), indicating the degree of unexplained variability in the logRR data after accounting for study-level differences. This suggests that while the stimulants are generally associated with an increase in risk, there is still considerable residual variability in the data that is not fully explained by the model.

Convergence Diagnostics: the model's Rhat values are all 1.00, which indicates that the chains have converged well, meaning that the model has successfully mixed and that the estimates are reliable. Rhat is a convergence diagnostic that compares the variance within each chain to the variance between chains. Additionally, the effective sample sizes (Bulk_ESS and Tail_ESS) are all sufficiently high, suggesting that the posterior estimates are stable and well-supported by the data.

eTable 4. Bayesian analysis for overall AEs

Parameter	Estimate	Standard Error (Est.Error)	95% CrI Lower (l-95% CrI)	95% CrI Upper (u-95% CrI)	Rhat	Bulk_ESS	Tail_ESS
**Intercept**	0.38	0.05	0.27	0.49	1.00	3843	4159
**Standard Deviation (study-level)**	0.27	0.10	0.04	0.43	1.00	552	1772
**Residual Standard Deviation (sigma)**	0.30	0.08	0.17	0.45	1.01	545	1168

Table legend: **Estimate**: the posterior mean of the parameter, representing the best estimate of the parameter's value after accounting for the data and prior information. **Standard Error** (Est.Error): the standard deviation of the posterior distribution of the parameter, representing the uncertainty or variability in the estimate. **95% CrI Lower** (l-95% CrI): the lower bound of the 95% credible interval for the parameter, indicating the range within which the true value of the parameter lies with 95% probability. **95% CrI Upper** (u-95% CrI): the upper bound of the 95% credible interval for the parameter, indicating the range within which the true value of the parameter lies with 95% probability. **Rhat**: the potential scale reduction factor, which is a measure of convergence. An Rhat value close to 1.00 indicates that the chains have converged and that the parameter estimates are reliable. **Bulk_ESS**: the effective sample size for the bulk of the posterior distribution, which indicates how many independent samples the posterior resembles. Larger values indicate better mixing and more reliable estimates. **Tail_ESS**: the effective sample size for the tail of the posterior distribution, representing the reliability of extreme values in the distribution. Larger values suggest more reliable estimates of the tails.

the results from this Bayesian meta-analysis suggest that stimulants are associated with a statistically significant increase in the risk of adverse events. the positive treatment effect (logRR = 0.38) means that, on average, stimulant treatment is linked to a 46% higher risk of experiencing adverse events compared to controls. Importantly, the 95% CrI for this effect does not cross zero, providing strong evidence that the stimulants do indeed increase the risk. Furthermore, the study-level variability captured by the random intercepts indicates that the treatment effects differ across studies, suggesting that some studies report stronger effects than others. This variability may be due to differences in study populations, methodologies, or other unaccounted factors. Overall, the model provides a robust estimate of the treatment effect and quantifies the uncertainty around that estimate. Given the credible intervals, this result can be considered as solid evidence of a positive association between stimulant use and adverse events. However, the residual error suggests there might still be additional factors influencing the risk, which were not captured in the model.

These findings underscore the importance of considering both the overall treatment effect and the study-level variability when interpreting meta-analytic results, especially when planning future research or clinical decisions based on this evidence. the posterior

distribution of the treatment effect (logRR) can be visualized through a plot (eFigure 10), which helps illustrate the uncertainty around the treatment effect estimate. This distribution allows to see the range of possible treatment effects, providing a clear picture of the precision of our estimate. Based on the model output, we can expect this distribution to be concentrated around 0.38, with a tail extending toward smaller and larger values of the effect.

eFigure 10. Posterior distribution for overall AEs

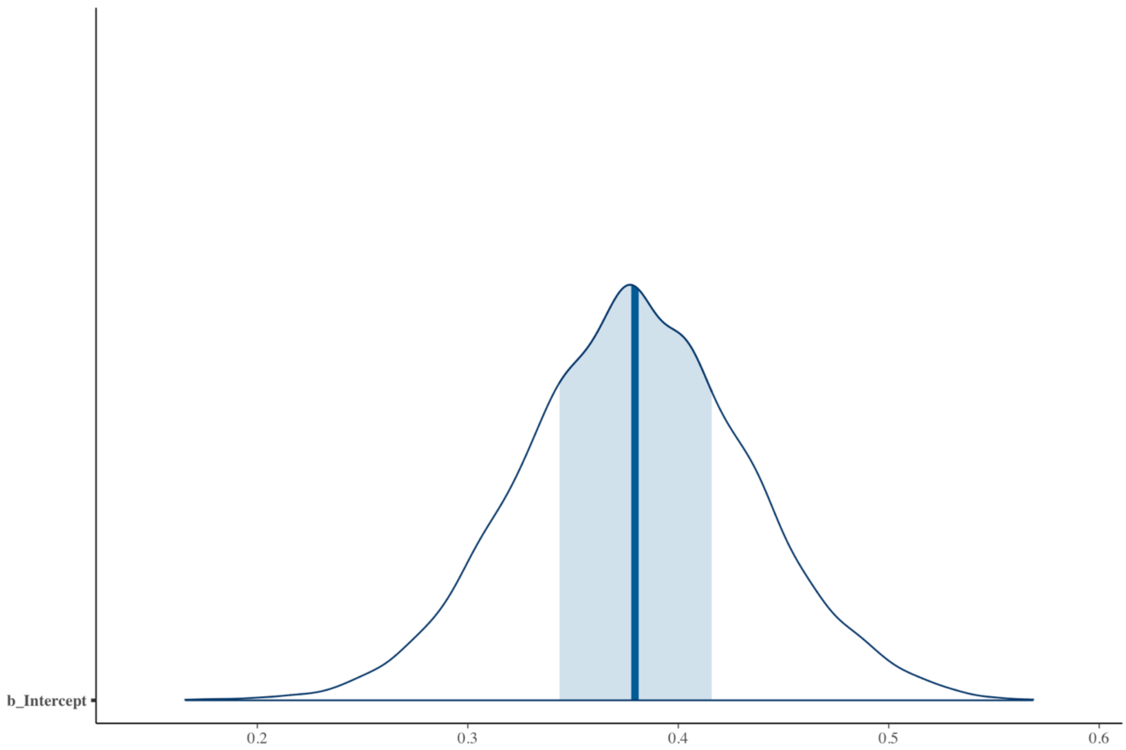

Figure legend: Posterior distribution of the treatment effect (logRR) for overall adverse events (AEs).

Example R code using Bayesian framework

```
## Install required packages
# install.packages(c("meta", "brms", "ggplot2", bayesplot"))
## Load the libraries
library(meta)      # For meta-analysis
library(brms)      # For Bayesian meta-analysis
library(ggplot2)   # For visualization
library(bayesplot) # For visualizing Bayesian results

# Load your data from the CSV file
data <- read.csv("Safety_of_Stimulants.csv") #

# Check the first few rows of the data
head(data)

## Ensure the data is structured properly for meta-analysis
# in our case, we created a 'meta-analysis' object for the Mantel-Haenszel method
# Perform the meta-analysis using Mantel-Haenszel method for Risk Ratio (RR)
meta_analysis <- metabin(
  event.e = event.e,          # Number of events in the exposed group
  n.e = n.e,                  # Number of participants in the exposed group
  event.c = event.c,          # Number of events in the control group
  n.c = n.c,                  # Number of participants in the control group
  data = data,                # Your dataset
  sm = "RR",                  # Effect size: Risk Ratio (RR)
  method = "MH"               # Mantel-Haenszel method
)

# Print the meta-analysis results
print(meta_analysis)

# Generate the forest plot
forest(meta_analysis,
       main = "Forest Plot for Risk Ratio",
       xlab = "Risk Ratio (95% CI)",
       refline = 1,             # Reference line at RR = 1 (null effect)
       fontsize = 14,           # Font size
       digits = 2)              # Display Risk Ratios with 2 decimal places

## Creating a data frame with the necessary variables
# We used study-specific effect sizes and variances
# Calculate the Risk Ratio (RR) and its log-transformed version for the Bayesian
analysis
data$RR <- data$event.e / data$n.e / (data$event.c / data$n.c) # Calculate Risk Ratio for
each study
data$logRR <- log(data$RR)  # Log-transformed Risk Ratio (used in the Bayesian
model)
# Create a new column for standard error of the log(RR)
# Standard error of logRR can be calculated as:
```

```
# SE(logRR) = sqrt(1/event.e + 1/event.c - 1/n.e - 1/n.c)
data$logRR_se <- sqrt(1/data$event.e + 1/data$event.c - 1/data$n.e - 1/data$n.c)
# Ensure the 'study' variable is a factor
data$study <- factor(data$study)
# Check the levels of the study variable
levels(data$study)
# Remove rows with Inf values in logRR
data_clean <- data[!is.infinite(data$logRR), ]
# Check the summary again
summary(data_clean$logRR)

## Fit a Bayesian random effects model using brms
bayesian_model <- brm(
  formula = logRR ~ 1 + (1|study),  # Model with random intercepts
  data = data_clean,                # Cleaned data
  family = gaussian(),              # Gaussian distribution for the log(RR)
  prior = c(
    prior(normal(0, 5), class = "Intercept")  # Prior for the intercept
  ),
  chains = 4,                       # Number of chains
  iter = 4000,                      # Increased number of iterations
  warmup = 2000,                    # Increased warm-up iterations
  control = list(adapt_delta = 0.95)  # Control to reduce divergent transitions
)
# Print the summary of the Bayesian model
summary(bayesian_model)
# Visualize the posterior distribution of the treatment effect (logRR)
posterior <- as.array(bayesian_model)
bayesplot::mcmc_areas(posterior, pars = "b_Intercept") +
  ggtitle("Posterior Distribution of Treatment Effect (logRR)")
summary(data$logRR)
```

Bayesian Framework for Meta-Analysis Using brms

in this analysis, we used Bayesian random effects modeling to estimate the treatment effect (in this case, the log of the Risk Ratio, or logRR) across multiple studies.[96,97] This approach provides a way to incorporate prior knowledge and quantify uncertainty in model parameters, rather than relying on frequentist point estimates.

1. Hierarchical (Random Effects) Model

the Bayesian approach can be thought of as an extension of the classical random effects model. in a random effects meta-analysis, the true effect of the treatment is assumed to vary across studies. the model accounts for both within-study and between-study variance.

the general hierarchical model for the log-transformed Risk Ratio (logRR) in the Bayesian framework can be expressed as:

$$y_i \sim N\left(\mu_i, \sigma^2_{study}\right)$$

Where:

- y_i represents the log-transformed effect size (logRR) for the i-th study.

  μ_i is the study-specific mean effect, modeled as the overall treatment effect μ plus a random effect u_i:

  $$\mu_i = \mu + u_i$$

  where $u_i \sim N\left(0, \sigma^2_{study}\right)$ represents the random effect for the i-th study, and σ^2_{study} is the between-study variance.

- σ^2_{study} represents the variance of the random effects (study-level deviations).

the model specification used in the brm() function, logRR ~ 1 + (1 | study), corresponds to the following:

$$y_i = \mu + u_i + \epsilon_i$$

Where:

- y_i is the global intercept, representing the overall treatment effect across studies (i.e., the average logRR).
- u_i is the random intercept for the i-th study, capturing the variability in the treatment effect across studies.
- $\epsilon_i \sim N(0, \sigma^2_{obs})$ represents the residual error for each study, modeling within-study variation.

2. Prior Distributions

in Bayesian inference, we must specify prior distributions for the model parameters. in the R code, a normal prior is used for the intercept:

$$\mu \sim N(0{,}5)$$

This means that we assume a prior belief that the average treatment effect is normally distributed with a mean of 0 (no effect) and a standard deviation of 5. This prior distribution reflects the uncertainty about the average treatment effect before observing the data. For the random effects variance σ^2_{study}, a default prior (usually half-Cauchy or inverse gamma) is used, but it can be specified it explicitly if needed.

3. Posterior Distribution

Once the model is specified and the data are observed, the goal of Bayesian analysis is to estimate the posterior distribution of the parameters. the posterior distribution combines the prior beliefs and the likelihood of the data:

$$p(\mu, \sigma^2_{study} \mid y) \propto p(y \mid \mu, \sigma^2_{study})\, p(\mu)\, p(\sigma^2_{study})$$

Where:

- $p(y \mid \mu, \sigma^2_{study})$ is the likelihood of observing the data given the parameters.
- $p(\mu)$ and $p\left(\sigma^2_{study}\right)$ are the prior distributions for the parameters.

the posterior is computed using Markov Chain Monte Carlo (MCMC) methods, which provide samples from the posterior distribution. These samples can be used to estimate the parameter values, quantify uncertainty, and make inferences about the treatment effect.

4. Posterior Summaries and Visualization

Once the posterior distribution is obtained, we typically summarize it using various statistics:

- Posterior mean: the average of the samples, representing the best estimate of the parameter.
- Posterior credible intervals (CrIs): intervals within which the parameter lies with a specified probability (e.g., 95% credible interval).

For the parameter of interest, μ (the average logRR), the posterior distribution is summarized as:

$$\mu \sim N\left(\hat{\mu}, \hat{\sigma}^2_{\mu}\right)$$

Where $\hat{\mu}$ is the posterior mean and $\hat{\sigma}^2_{\mu}$ is the posterior variance.

We can visualize the posterior distribution using plots, such as the MCMC trace plot or density plot, to assess convergence and uncertainty.

5. Model Output

the output of the brm() function includes:

- Posterior estimates of the fixed effects (overall treatment effect, μ) and the random effects variance (σ^2_{study}).
- Diagnostics: checking for convergence (e.g., Gelman-Rubin diagnostic), effective sample size, and autocorrelation.
- Posterior predictive checks: evaluating the fit of the model to the data.

Mathematical Model Summary:

1. Model Formula:

$$y_i = \mu + u_i + \epsilon_i$$

   - y_i is the log-transformed treatment effect for study ii.
   - μ is the global mean treatment effect.
   - u_i is the random effect (study-specific deviation).
   - ϵ_i is the residual error.

2. Random Effects:

$$u_i \sim N\left(0, \sigma^2_{study}\right)$$

3. Prior Distribution for Intercept:

$$\mu \sim N(0, 5)$$

4. Likelihood:

$$p(y_i \mid \mu, \sigma^2_{study}) = N(y_i \mid \mu, \sigma^2_{study} + \sigma^2_{obs})$$

5. Posterior:

$$p(\mu, \sigma^2_{study} \mid y) \propto p(y \mid \mu, \sigma^2_{study})\, p(\mu)\, p(\sigma^2_{study})$$

Systolic Blood Pressure (SBP)

We fit a Bayesian random effects model to investigate the relationship between stimulant medication use and changes in systolic blood pressure (SBP) across studies (eTable 5 and eFigure 11). the model accounts for study-specific variations in the outcome.

the intercept estimate is 0.46 (95% CrI: -0.48 to 1.39), suggesting a modest positive effect of stimulant medication on systolic blood pressure across studies. This implies a small increase in systolic blood pressure as a result of stimulant use, but with substantial uncertainty about the exact size of the effect.
the study-level variability (sd = 1.64, 95% CrI: 0.11 to 2.97) indicates notable differences in the magnitude of SBP responses across individual studies, reflecting the heterogeneity in treatment effects across studies. This highlights the diversity of outcomes in different trial settings.
the residual standard deviation (σ = 1.37, 95% CrI: 0.29 to 2.79) reflects unexplained variability in SBP changes even after accounting for study-specific effects, suggesting that additional factors not captured in the model may influence systolic blood pressure changes.

Model diagnostics:
R-hat values are close to 1 for most parameters (1.00 for the intercept, 1.01 for study variability, 1.01 for residual variability), indicating that the model chains have converged well and that the parameter estimates are reliable.
Effective sample sizes are sufficiently large (Bulk ESS and Tail ESS), particularly for the intercept and study variability, ensuring reliable posterior estimates for these parameters. However, the residual variability has a lower effective sample size (Bulk ESS = 749, Tail ESS = 3308), which may warrant some caution in interpreting the residual standard deviation.
Since the data come from controlled trials, we can make a causal inference that stimulant medications are associated with changes in systolic blood pressure. the randomized design of the studies helps control for confounding factors, allowing us to attribute the observed changes in SBP to the stimulant treatment. While the model diagnostics suggest good convergence and reliable estimates, the study-level variability and residual variability point to the need for further exploration. Additional iterations, including more studies, or pooling individual participant data may help improve the robustness and precision of these findings.

eTable 5. Bayesian analysis results for systolic blood pressure

Parameter	Estimate	Standard Error (Est.Error)	95% CrI Lower (l-95% CrI)	95% CrI Upper (u-95% CrI)	Rhat	Bulk_ESS	Tail_ESS
Intercept	0.46	0.47	-0.48	1.39	1.00	3014	1698
Standard Deviation (study-level)	1.64	0.77	0.11	2.97	1.01	749	3308
Residual Standard Deviation (sigma)	1.37	0.72	0.29	2.79	1.01	440	542

Table Legend: Estimate: the posterior mean of the parameter, representing the best estimate of the parameter's value after accounting for the data and prior information. **Standard Error (Est.Error)**: the standard deviation of the posterior distribution of the parameter, representing the uncertainty or variability in the estimate. **95% CrI Lower (l-95% CrI)**: the lower bound of the 95% credible interval for the parameter, indicating the range within which the true value of the parameter lies with 95% probability. **95% CrI Upper (u-95% CrI)**: the upper bound of the 95% credible interval for the parameter, indicating the range within which the true value of the parameter lies with 95% probability. **Rhat**: the potential scale reduction factor, which is a measure of convergence. An Rhat value close to 1.00 indicates that the chains have converged and that the parameter estimates are reliable. **Bulk_ESS**: the effective sample size for the bulk of the posterior distribution, which indicates how many independent samples the posterior resembles. Larger values indicate better mixing and more reliable estimates. **Tail_ESS**: the effective sample size for the tail of the posterior distribution, representing the reliability of extreme values in the distribution. Larger values suggest more reliable estimates of the tails.

eFigure 11. Posterior distribution for systolic blood pressure

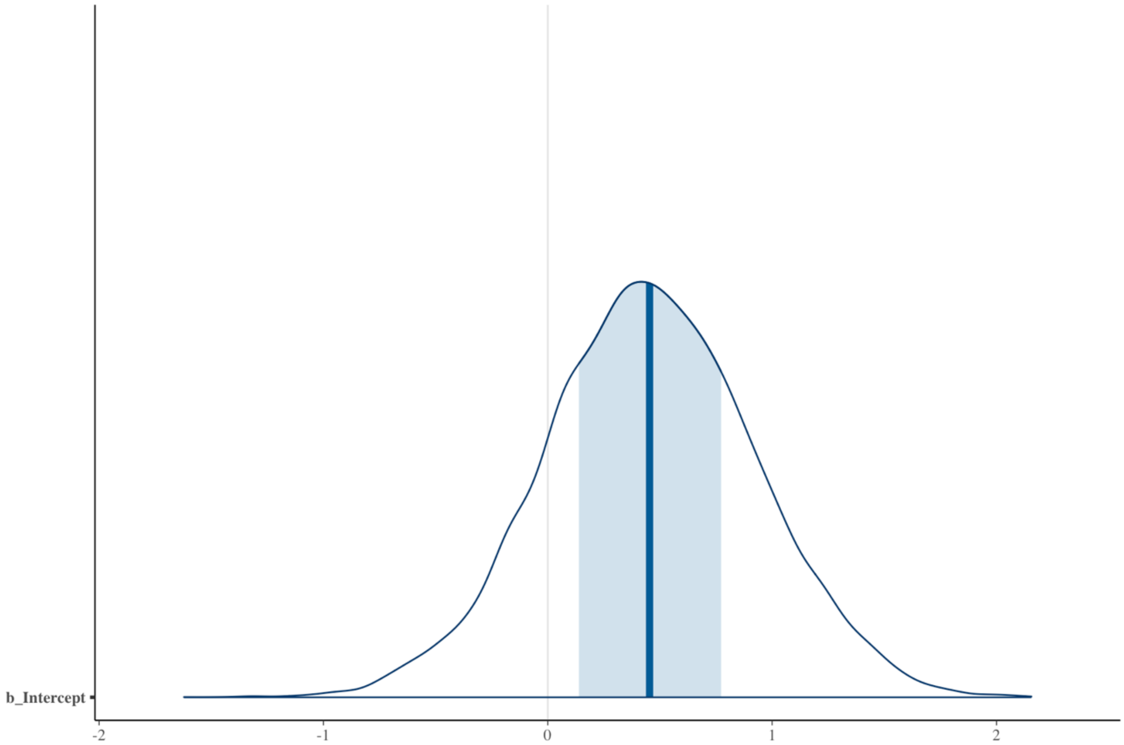

Figure legend: Posterior distribution of the treatment effect (mean difference) for systolic blood pressure.

Diastolic Blood Pressure (DBP)

We fit a Bayesian random effects model to investigate the relationship between stimulant medication use and changes in diastolic blood pressure (denoted as md, mean difference) across studies. This model accounts for study-specific variations in the outcome (eTable 7 and eFigure 13).
the intercept estimate is 1.86 (95% CrI: 0.64 to 3.01), suggesting a positive effect of stimulant medication on diastolic blood pressure across studies. This indicates a baseline increase in DBP associated with stimulant use.
the study-level variability (sd = 2.46, 95% CrI: 0.32 to 4.10) shows considerable differences in the magnitude of DBP responses across individual studies, highlighting the heterogeneity in effects. This variability suggests that while the overall effect is positive, the response to stimulant medications varies significantly between studies.
the residual standard deviation (σ = 1.56, 95% CrI: 0.31 to 3.66) reflects unexplained variability in DBP changes even after accounting for study-specific effects. This suggests that while the model accounts for study-level differences, some degree of variability remains that cannot be attributed to specific studies.

Model Diagnostics:
R-hat values are close to 1 for most parameters (1.00 for the intercept, 1.01 for study variability, 1.01 for residual variability), indicating good convergence of the model chains. These values suggest that the model has adequately converged and the parameter estimates are reliable.
Effective sample sizes (Bulk ESS and Tail ESS) are sufficiently large, particularly for the intercept and study variability, indicating reliable posterior estimates for these parameters. For example, the Bulk ESS for the intercept is 940, and the Tail ESS is 1242, indicating high confidence in the estimates. However, some caution is warranted when interpreting the residual variability due to a slightly lower effective sample size (Bulk ESS = 199, Tail ESS = 148), which suggests that the estimate of residual variability may be less precise.
Since the data come from controlled trials, we can make a causal inference that stimulant medications are associated with increased diastolic blood pressure. the randomized design helps control for confounding factors, allowing us to attribute changes in DBP to the stimulant treatment. While the model diagnostics suggest adequate convergence and reliable estimates, the study-level variability and residual variability point to the need for further exploration. Additional iterations, including more studies, or pooling individual participant data may help improve the robustness and precision of these findings.

eTable 6. Bayesian analysis results for diastolic blood pressure

**Parameter**	**Estimate**	**Standard Error (Est.Error)**	**95% CrI Lower (l-95% CrI)**	**95% CrI Upper (u-95% CrI)**	**Rhat**	**Bulk_ESS**	**Tail_ESS**
**Intercept**	1.86	0.61	0.64	3.01	1.00	940	1242
**Standard Deviation (study-level)**	2.46	0.92	0.32	4.10	1.01	557	2454
**Residual Standard Deviation (σ)**	1.56	0.97	0.31	3.66	1.01	199	148

Table Legend: **Estimate:** the posterior mean of the parameter, representing the best estimate of the parameter's value after accounting for the data and prior information. **Standard Error (Est.Error):** the standard deviation of the posterior distribution of the parameter, representing the uncertainty or variability in the estimate. **95% CrI Lower (l-95% CrI):** the lower bound of the 95% credible interval for the parameter, indicating the range within which the true value of the parameter lies with 95% probability. **95% CrI Upper (u-95% CrI):** the upper bound of the 95% credible interval for the parameter, indicating the range within which the true value of the parameter lies with 95% probability. **Rhat:** the potential scale reduction factor, which is a measure of convergence. An Rhat value close to 1.00 indicates that the chains have converged and that the parameter estimates are reliable. **Bulk_ESS:** the effective sample size for the bulk of the posterior distribution, which indicates how many independent samples the posterior resembles. Larger values indicate better mixing and more reliable estimates. **Tail_ESS:** the effective sample size for the tail of the posterior distribution, representing the reliability of extreme values in the distribution. Larger values suggest more reliable estimates of the tails.

eFigure 12. Posterior distribution for diastolic blood pressure

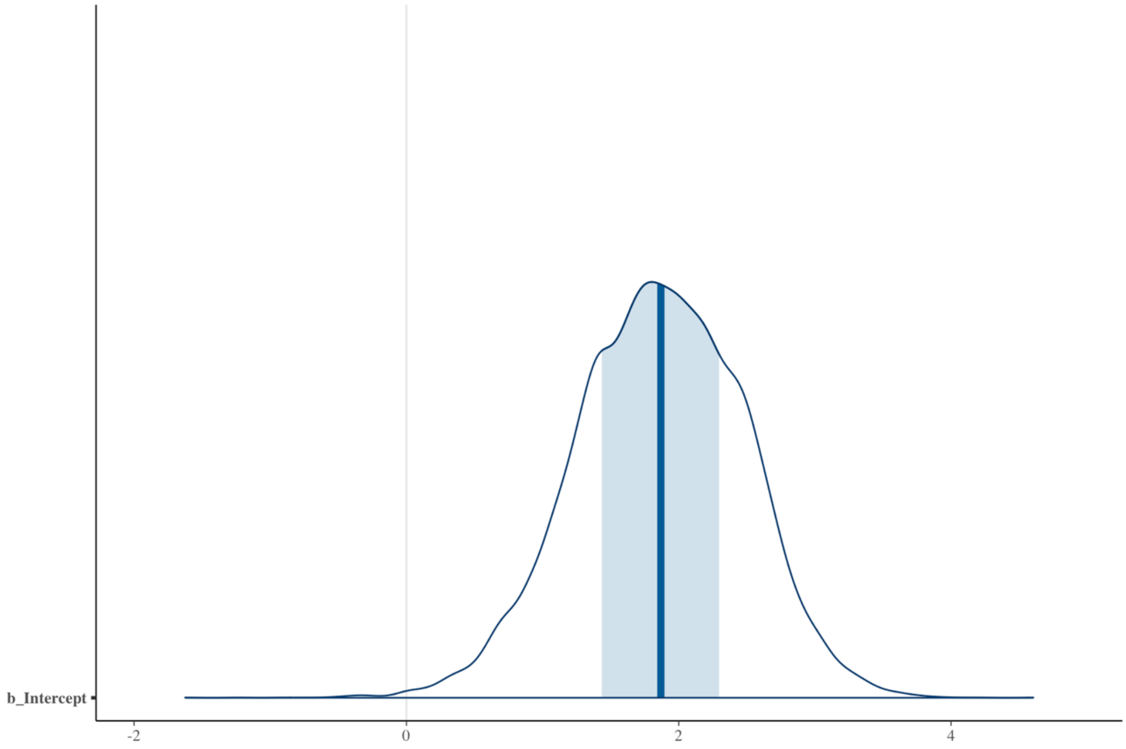

Figure legend: Posterior distribution of the treatment effect (mean difference) for diastolic blood pressure.

Heart Rate (HR)

We fit a Bayesian random effects model to investigate the relationship between stimulant medication use and changes in heart rate (denoted as md, mean difference) across studies (*eTable 7* and *eFigure 13*). the model accounts for study-specific variations in the outcome.

the **intercept estimate** is 1.83 (95% CrI: 0.52 to 3.01), suggesting a positive effect of stimulant medication on heart rate across studies. This indicates a baseline increase in heart rate as a result of stimulant use.

the **study-level variability** (sd = 2.50, 95% CrI: 0.30 to 4.19) shows considerable differences in the magnitude of heart rate responses across individual studies, highlighting the heterogeneity in effects.

the **residual standard deviation** (σ = 1.56, 95% CrI: 0.33 to 3.71) reflects unexplained variability in heart rate changes even after accounting for study-specific effects.

Model diagnostics:

R-hat values are close to 1 for most parameters (1.00 for the intercept, 1.01 for study variability, 1.02 for residual variability), suggesting good convergence of the model chains.

Effective sample sizes are sufficiently large (Bulk ESS and Tail ESS), particularly for the intercept and study variability, indicating reliable posterior estimates for these parameters. However, some caution is warranted when interpreting the residual variability due to a somewhat lower ESS (Bulk ESS = 336, Tail ESS = 565).

Since the data come from controlled trials, we can make a causal inference that stimulant medications are associated with increased heart rate. the randomized design helps to control for confounding factors, allowing us to attribute changes in heart rate to the stimulant treatment. While the model diagnostics suggest adequate convergence and reliable estimates, the study-level variability and residual variability point to the need for further exploration. Additional iterations, including more studies, or pooling individual participant data may help improve the robustness and precision of these findings.

eTable 7. Bayesian analysis results for heart rate

Parameter	Estimate	Standard Error (Est.Error)	95% CrI Lower (l-95% CrI)	95% CrI Upper (u-95% CrI)	Rhat	Bulk_ESS	Tail_ESS
**Intercept**	1.83	0.63	0.52	3.01	1.00	2653	2156
**Standard Deviation (study-level)**	2.50	0.95	0.30	4.19	1.01	785	2749
**Residual Standard Deviation (sigma)**	1.56	0.98	0.33	3.71	1.02	336	565

Table legend: **Estimate**: the posterior mean of the parameter, representing the best estimate of the parameter's value after accounting for the data and prior information. **Standard Error** (Est.Error): the standard deviation of the posterior distribution of the parameter, representing the uncertainty or variability in the estimate. **95% CrI Lower** (l-95% CrI): the lower bound of the 95% credible interval for the parameter, indicating the range within which the true value of the parameter lies with 95% probability. **95% CrI Upper** (u-95% CrI): the upper bound of the 95% credible interval for the parameter, indicating the range within which the true value of the parameter lies with 95% probability. **Rhat**: the potential scale reduction factor, which is a measure of convergence. An Rhat value close to 1.00 indicates that the chains have converged and that the parameter estimates are reliable. **Bulk_ESS**: the effective sample size for the bulk of the posterior distribution, which indicates how many independent samples the posterior resembles. Larger values indicate better mixing and more reliable estimates. **Tail_ESS**: the effective sample size for the tail of the posterior distribution, representing the reliability of extreme values in the distribution. Larger values suggest more reliable estimates of the tails.

eFigure 13. Posterior distribution for heart rate

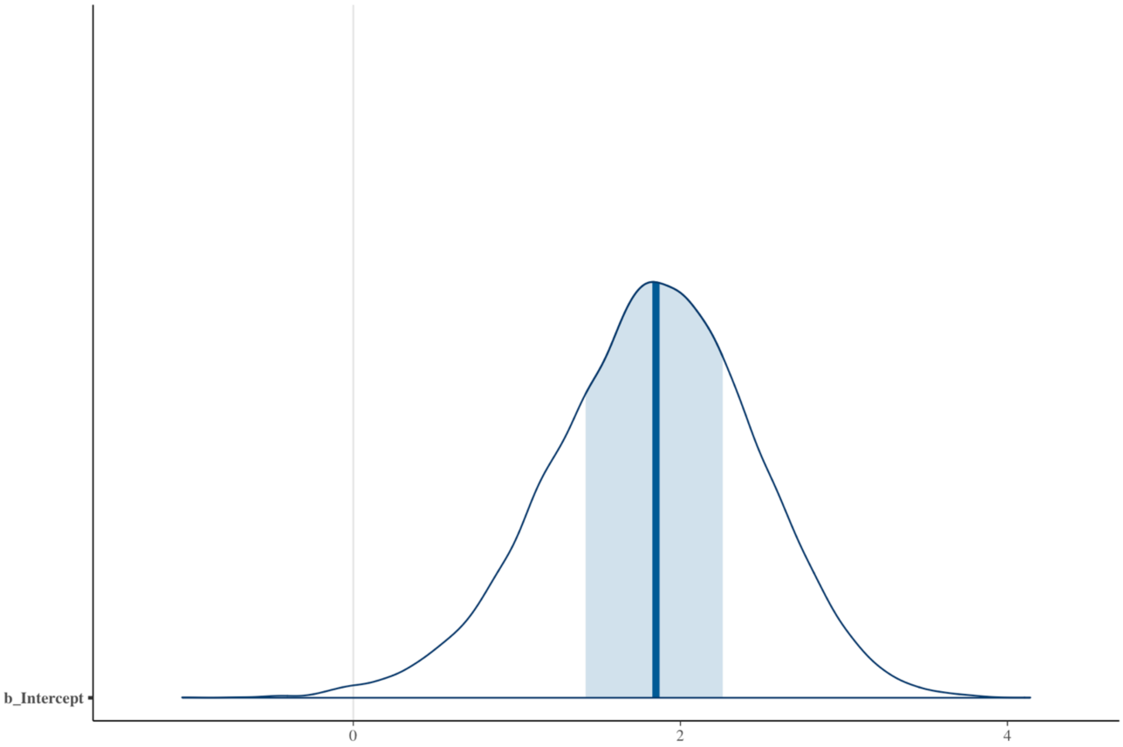

Figure legend: Posterior distribution of the treatment effect (mean difference) for heart rate.

Methods for subgroup division in the forest plots

To explore potential sources of heterogeneity in the meta-analysis, we conducted subgroup analyses based on age, duration of drug use, and drug dose. These subgroup classifications were applied consistently across all included studies to enable meaningful comparisons and to assess potential moderators of treatment effects.

Age Subgroups

Participants were categorized into two age groups: children/adolescents, defined as individuals up to 18 years old, and adults/older adults, defined as individuals over 18 years old. This classification was based on widely accepted age groupings in clinical and epidemiological research, as well as the most used divisions found in the included studies.

Duration of Drug Use Subgroups

the duration of drug use was classified into three categories: short-term use, defined as four or fewer weeks of treatment; medium-term use, defined as five to 12 weeks of treatment; and long-term use, defined as 13 or more weeks of treatment.[98]

Drug Dose Subgroups

Drug doses were categorized as low, medium, or high according to the drug labels provided by regulatory agencies and manufacturers. When necessary, dose ranges were standardized across studies to facilitate comparison.

eFigures 14-44. Forest plots dividing groups according to participant age.

eFigure 14. Forest plot showing the risk ratio of **all adverse events** between control and experimental groups using **methylphenidate** subdivided by age.

Study or Subgroup	Experimental Events	Experimental Total	Control Events	Control Total	Weight	Risk Ratio M-H, Random, 95% CI
**13.10.1 Children/Adolescents**						
Brams et al, 2008	15	86	19	86	1.1%	0.79 [0.43, 1.45]
Childress et al, 2009	116	182	36	63	3.1%	1.12 [0.88, 1.42]
Childress et al, 2020A	24	65	22	54	1.7%	0.91 [0.58, 1.42]
Childress et al, 2020B	18	75	7	73	0.7%	2.50 [1.11, 5.63]
Childress et al, 2020C	10	39	6	50	0.5%	2.14 [0.85, 5.37]
Coghill et al, 2013 - MPH	72	111	63	110	3.4%	1.13 [0.92, 1.40]
Findling et al, 2008 - MPH	74	98	49	85	3.4%	1.31 [1.06, 1.62]
Findling et al, 2008 - MTS	63	91	49	85	3.3%	1.20 [0.96, 1.51]
Greenhill et al, 2002	80	155	61	161	3.1%	1.36 [1.06, 1.75]
Huang et al, 2021	79	110	10	101	1.1%	7.25 [3.98, 13.21]
Muniz et al, 2008	40	332	0	83	0.1%	20.43 [1.27, 328.88]
Newcorn et al, 2008	146	219	40	74	3.2%	1.23 [0.98, 1.55]
Pliszka et al, 2017	56	81	39	80	2.9%	1.42 [1.09, 1.85]
Schulz et al, 2010	44	146	38	146	2.1%	1.16 [0.80, 1.67]
Silva et al, 2005 - ER	5	53	2	53	0.2%	2.50 [0.51, 12.32]
Silva et al, 2005 - OROS	11	53	2	53	0.2%	5.50 [1.28, 23.63]
Sugaya et al, 2022	4	51	4	51	0.3%	1.00 [0.26, 3.78]
Weiss et al, 2021	154	293	24	74	2.3%	1.62 [1.15, 2.29]
Wilens et al, 2006	15	87	14	90	0.9%	1.11 [0.57, 2.16]
Winhusen et al, 2011	111	151	98	152	4.0%	1.14 [0.98, 1.33]
Wolraich et al, 2001 - IR	40	95	31	90	2.1%	1.22 [0.84, 1.77]
Wolraich et al, 2001 - OROS	45	97	31	90	2.2%	1.35 [0.94, 1.92]
Zheng et al, 2024	74	110	55	112	3.2%	1.37 [1.09, 1.72]
**Subtotal (95% CI)**		**2780**		**2016**	**45.2%**	**1.34 [1.18, 1.53]**
Total events	1296		700			
Heterogeneity: $Tau^2 = 0.05; Chi^2 = 63.03, df = 22 (P < 0.00001); I^2 = 65\%$						
Test for overall effect: Z = 4.56 (P < 0.00001)						
**13.10.2 Adults/Older adults**						
Adler et al, 2009	93	110	74	116	3.9%	1.33 [1.13, 1.55]
Bron et al, 2014	17	22	10	22	1.4%	1.70 [1.02, 2.83]
Buitelaar et al, 2012	7	23	8	22	0.7%	0.84 [0.37, 1.92]
Casas et al, 2013	161	181	76	97	4.3%	1.14 [1.01, 1.28]
Childress et al, 2022	25	121	18	118	1.3%	1.35 [0.78, 2.35]
Ginsberg et al, 2014	175	216	65	82	4.2%	1.02 [0.90, 1.16]
Goodman et al, 2017	126	174	87	175	3.8%	1.46 [1.22, 1.74]
Huss et al, 2014	401	542	108	180	4.2%	1.23 [1.08, 1.40]
Jain et al, 2017	42	50	29	50	2.9%	1.45 [1.11, 1.89]
Kis et al, 2020	197	205	184	209	4.7%	1.09 [1.03, 1.16]
Kooji et al, 2004	37	45	31	45	3.2%	1.19 [0.94, 1.52]
Medori et al, 2008	182	305	41	96	3.1%	1.40 [1.09, 1.79]
Patkar et al, 2006	19	30	17	30	1.9%	1.12 [0.74, 1.69]
Retz et al, 2012	55	84	32	78	2.6%	1.60 [1.17, 2.17]
Rösler et al, 2009	135	183	37	66	3.2%	1.32 [1.05, 1.66]
Spencer et al, 2007	145	165	36	53	3.6%	1.29 [1.07, 1.57]
Takahashi et al, 2014	117	143	76	141	3.8%	1.52 [1.28, 1.80]
Weiss et al, 2020	158	297	25	78	2.3%	1.66 [1.18, 2.33]
**Subtotal (95% CI)**		**2896**		**1658**	**54.8%**	**1.29 [1.18, 1.40]**
Total events	2092		954			
Heterogeneity: $Tau^2 = 0.02; Chi^2 = 58.85, df = 17 (P < 0.00001); I^2 = 71\%$						
Test for overall effect: Z = 5.83 (P < 0.00001)						
**Total (95% CI)**		**5676**		**3674**	**100.0%**	**1.31 [1.22, 1.40]**
Total events	3388		1654			
Heterogeneity: $Tau^2 = 0.03; Chi^2 = 131.63, df = 40 (P < 0.00001); I^2 = 70\%$						
Test for overall effect: Z = 7.28 (P < 0.00001)						
Test for subgroup differences: $Chi^2 = 0.32, df = 1 (P = 0.57), I^2 = 0\%$						

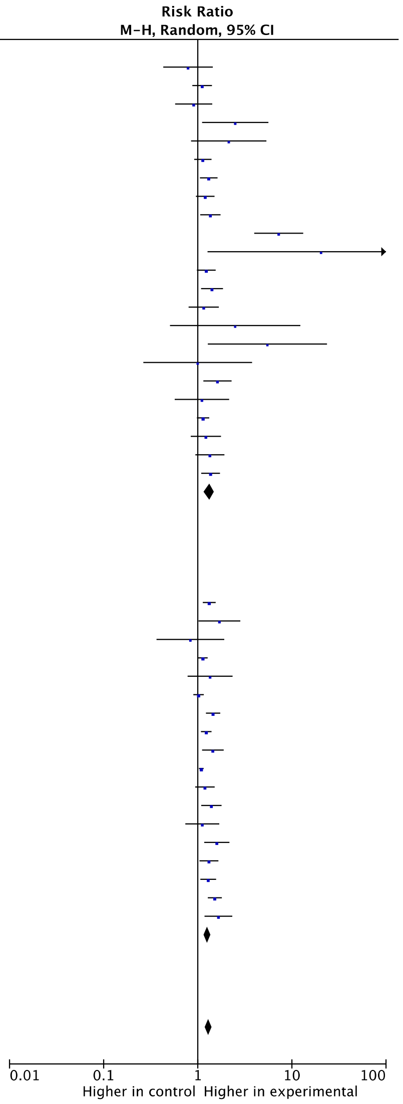

Figure legend: 95%CI, 95% confidence interval; IR, immediate release; M-H, Mantel–Haenszel; MPH, methylphenidate; MTS, transdermal system; OROS, osmotic release oral system; XR, ER extended-release.

eFigure 15. Forest plot showing the risk ratio of **anxiety** between control and experimental groups using **methylphenidate** subdivided by age.

Study or Subgroup	Experimental Events	Experimental Total	Control Events	Control Total	Weight	Risk Ratio M-H, Fixed, 95% CI
**13.6.1 Children/Adolescents**						
Froehlich et al, 2020	32	171	26	171	16.7%	1.23 [0.77, 1.97]
Lee et al, 2011	53	157	58	157	37.3%	0.91 [0.68, 1.23]
Pearson et al, 2013	6	24	4	24	2.6%	1.50 [0.48, 4.65]
Ramtvedt et al, 2014 - MPH	10	34	7	34	4.5%	1.43 [0.62, 3.31]
Stein et al, 2003	22	47	24	47	15.4%	0.92 [0.61, 1.39]
Sugaya et al, 2022	18	51	16	51	10.3%	1.13 [0.65, 1.95]
**Subtotal (95% CI)**		**484**		**484**	**86.9%**	**1.04 [0.86, 1.27]**
Total events	141		135			
Heterogeneity: $Chi^2 = 2.61$, df = 5 (P = 0.76); $I^2 = 0\%$						
Test for overall effect: Z = 0.44 (P = 0.66)						
**13.6.2 Adults/Older adults**						
Adler et al, 2009	18	110	4	116	2.5%	4.75 [1.66, 13.58]
Konstenius et al, 2014	1	27	4	27	2.6%	0.25 [0.03, 2.09]
Medori et al, 2008	16	305	1	96	1.0%	5.04 [0.68, 37.48]
Patkar et al, 2006	2	30	1	30	0.6%	2.00 [0.19, 20.90]
Rosenberg et al, 2014	17	29	10	29	6.4%	1.70 [0.94, 3.06]
**Subtotal (95% CI)**		**501**		**298**	**13.1%**	**2.26 [1.40, 3.64]**
Total events	54		20			
Heterogeneity: $Chi^2 = 7.56$, df = 4 (P = 0.11); $I^2 = 47\%$						
Test for overall effect: Z = 3.35 (P = 0.0008)						
**Total (95% CI)**		**985**		**782**	**100.0%**	**1.20 [1.01, 1.44]**
Total events	195		155			
Heterogeneity: $Chi^2 = 17.39$, df = 10 (P = 0.07); $I^2 = 42\%$						
Test for overall effect: Z = 2.03 (P = 0.04)						
Test for subgroup differences: $Chi^2 = 8.63$, df = 1 (P = 0.003), $I^2 = 88.4\%$						

Risk Ratio
M-H, Fixed, 95% CI

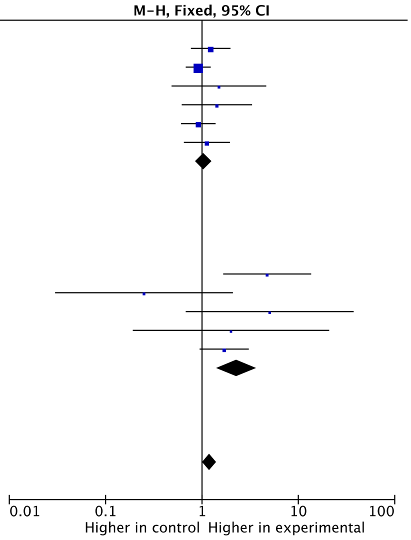

Figure legend: 95%CI, 95% confidence interval; M-H, Mantel–Haenszel; MPH, methylphenidate.

eFigure 16. Forest plot showing the risk ratio of **decreased appetite** between control and experimental groups using **methylphenidate** subdivided by age.

Study or Subgroup	Experimental Events	Experimental Total	Control Events	Control Total	Weight	Risk Ratio M-H, Random, 95% CI
**13.1.1 Children/Adolescents**						
Biederman et al, 2006	23	67	2	74	1.7%	12.70 [3.11, 51.85]
Chronis-Tuscano et al, 2008	2	20	1	23	0.8%	2.30 [0.23, 23.51]
Coghill et al, 2013 - MPH	17	111	3	110	2.1%	5.62 [1.69, 18.62]
Findling et al, 2008 - MPH	25	98	4	85	2.6%	5.42 [1.97, 14.95]
Findling et al, 2008 - MTS	17	91	4	85	2.5%	3.97 [1.39, 11.32]
Froehlich et al, 2020	106	171	45	171	4.8%	2.36 [1.79, 3.11]
Greenhill et al, 2002	15	155	4	161	2.4%	3.90 [1.32, 11.48]
Huang et al, 2021	53	110	1	101	1.1%	48.66 [6.86, 345.42]
Lee et al, 2011	77	157	31	157	4.6%	2.48 [1.74, 3.54]
Muniz et al, 2008	3	332	0	83	0.5%	1.77 [0.09, 33.86]
Newcorn et al, 2008	37	219	2	74	1.8%	6.25 [1.54, 25.31]
Pearson et al, 2013	9	24	1	24	1.1%	9.00 [1.23, 65.64]
Pelham Jr et al, 2005	22	36	2	36	1.8%	11.00 [2.79, 43.37]
Pliszka et al, 2017	15	81	3	80	2.1%	4.94 [1.49, 16.40]
Ramtvedt et al, 2014 - MPH	25	34	17	34	4.5%	1.47 [0.99, 2.18]
Schulz et al, 2010	3	146	2	146	1.3%	1.50 [0.25, 8.85]
Stein et al, 2003	35	47	16	47	4.3%	2.19 [1.42, 3.37]
Stein et al, 2003	35	47	16	47	4.3%	2.19 [1.42, 3.37]
Sugaya et al, 2022	13	51	3	51	2.1%	4.33 [1.31, 14.30]
Weiss et al, 2020	33	297	2	78	1.8%	4.33 [1.06, 17.67]
Wigal et al, 2006 - MPH	23	162	14	160	3.7%	1.62 [0.87, 3.04]
Zheng et al, 2024	33	110	2	112	1.8%	16.80 [4.13, 68.32]
**Subtotal (95% CI)**		**2566**		**1939**	**53.6%**	**3.45 [2.55, 4.67]**
Total events	621		175			
Heterogeneity: Tau² = 0.23; Chi² = 59.14, df = 21 (P < 0.0001); I² = 64%						
Test for overall effect: Z = 8.06 (P < 0.00001)						
**13.1.2 Adults/Older adults**						
Adler et al, 2009	28	110	7	116	3.2%	4.22 [1.92, 9.26]
Biederman et al, 2006	23	67	2	74	1.7%	12.70 [3.11, 51.85]
Casas et al, 2013	43	181	5	97	2.9%	4.61 [1.89, 11.25]
Childress et al, 2009	22	182	3	63	2.2%	2.54 [0.79, 8.19]
Ginsberg et al, 2014	15	216	11	82	3.4%	0.52 [0.25, 1.08]
Goodman et al, 2017	25	174	7	175	3.1%	3.59 [1.60, 8.09]
Huss et al, 2014	136	542	8	180	3.5%	5.65 [2.82, 11.29]
Jain et al, 2017	11	50	3	50	2.1%	3.67 [1.09, 12.36]
Kis et al, 2020	46	205	8	209	3.4%	5.86 [2.84, 12.11]
Kooji et al, 2004	10	45	2	45	1.7%	5.00 [1.16, 21.55]
Medori et al, 2008	77	305	7	96	3.3%	3.46 [1.65, 7.25]
Patkar et al, 2006	3	30	1	30	0.9%	3.00 [0.33, 27.23]
Retz et al, 2012	40	84	8	78	3.5%	4.64 [2.32, 9.29]
Rosenberg et al, 2014	8	29	7	29	2.9%	1.14 [0.48, 2.74]
Rösler et al, 2009	70	183	9	66	3.7%	2.81 [1.49, 5.29]
Spencer et al, 2007	30	165	6	53	3.1%	1.61 [0.71, 3.65]
Weiss et al, 2020	33	297	2	78	1.8%	4.33 [1.06, 17.67]
**Subtotal (95% CI)**		**2865**		**1521**	**46.4%**	**3.19 [2.20, 4.61]**
Total events	620		96			
Heterogeneity: Tau² = 0.36; Chi² = 44.78, df = 16 (P = 0.0002); I² = 64%						
Test for overall effect: Z = 6.16 (P < 0.00001)						
**Total (95% CI)**		**5431**		**3460**	**100.0%**	**3.35 [2.66, 4.21]**
Total events	1241		271			
Heterogeneity: Tau² = 0.27; Chi² = 105.91, df = 38 (P < 0.00001); I² = 64%						
Test for overall effect: Z = 10.34 (P < 0.00001)						
Test for subgroup differences: Chi² = 0.11, df = 1 (P = 0.74), I² = 0%						

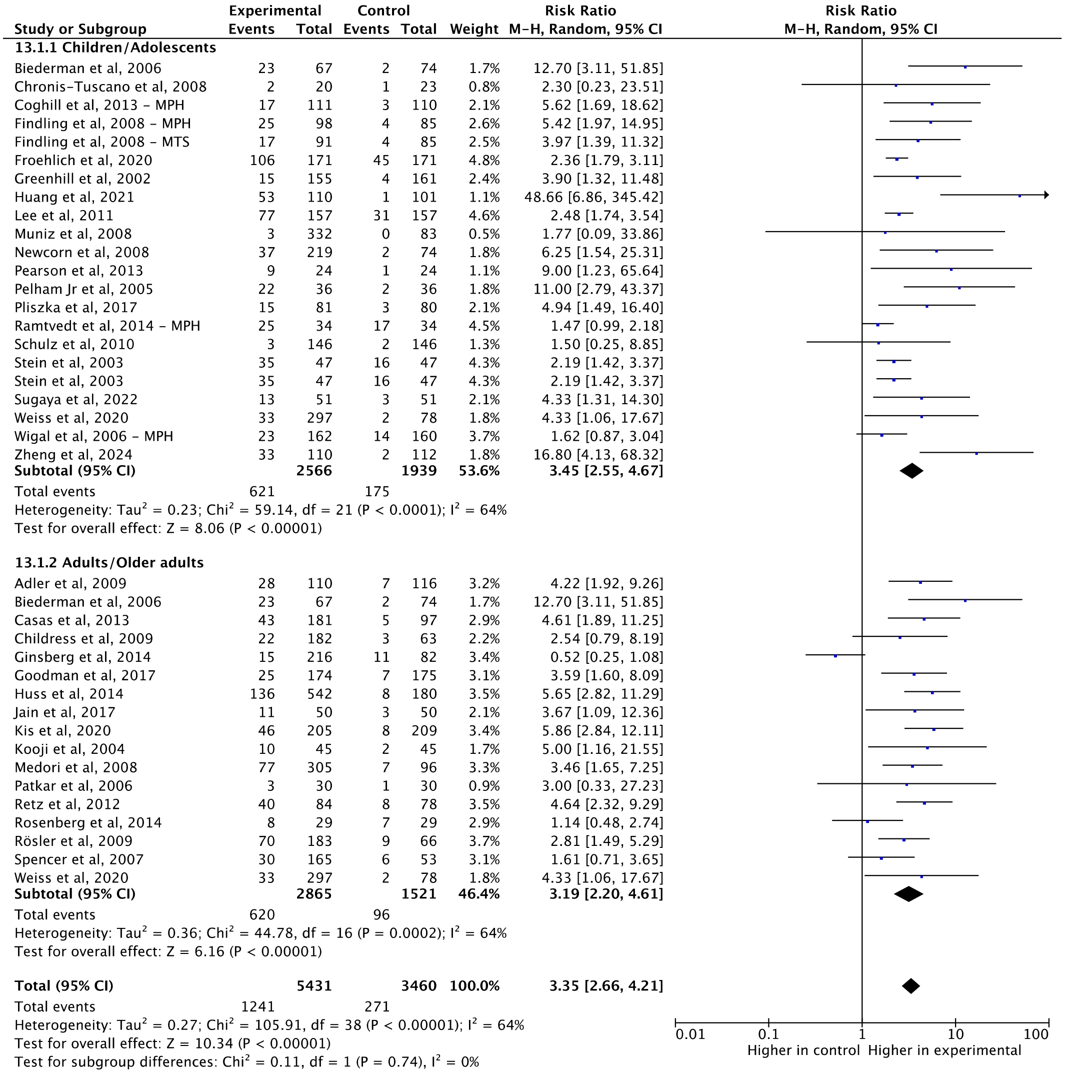

Figure legend: 95%CI, 95% confidence interval; M-H, Mantel–Haenszel; MPH, methylphenidate; MTS, transdermal system.

eFigure 17. Forest plot showing the risk ratio of **dry mouth** between control and experimental groups using **methylphenidate** subdivided by age.

Study or Subgroup	Experimental Events	Experimental Total	Control Events	Control Total	Weight	Risk Ratio M-H, Random, 95% CI
**13.5.1 Children/Adolescents**						
**Subtotal (95% CI)**		**0**		**0**		**Not estimable**
Total events	0		0			
Heterogeneity: Not applicable						
Test for overall effect: Not applicable						
**13.5.2 Adults/Older adults**						
Adler et al, 2009	22	110	6	116	8.1%	3.87 [1.63, 9.18]
Biederman et al, 2006	23	67	5	74	7.7%	5.08 [2.05, 12.61]
Casas et al, 2013	32	181	3	97	6.1%	5.72 [1.80, 18.19]
Ginsberg et al, 2014	15	216	9	82	8.6%	0.63 [0.29, 1.39]
Goodman et al, 2017	27	174	3	175	6.0%	9.05 [2.80, 29.29]
Huss et al, 2014	110	542	4	180	7.2%	9.13 [3.42, 24.41]
Jain et al, 2017	6	50	1	50	2.8%	6.00 [0.75, 48.05]
Kis et al, 2020	30	205	10	209	9.4%	3.06 [1.54, 6.09]
Konstenius et al, 2014	2	27	1	27	2.3%	2.00 [0.19, 20.77]
Kooji et al, 2004	11	45	3	45	5.8%	3.67 [1.10, 12.27]
Medori et al, 2008	36	305	2	96	4.9%	5.67 [1.39, 23.10]
Retz et al, 2012	32	84	11	78	10.0%	2.70 [1.47, 4.98]
Rösler et al, 2009	55	183	10	66	10.0%	1.98 [1.08, 3.66]
Spencer et al, 2007	26	165	2	53	4.9%	4.18 [1.03, 17.01]
Weiss et al, 2020	27	297	3	78	6.1%	2.36 [0.74, 7.59]
**Subtotal (95% CI)**		**2651**		**1426**	**100.0%**	**3.37 [2.29, 4.98]**
Total events	454		73			
Heterogeneity: $Tau^2 = 0.29$; $Chi^2 = 31.33$, df = 14 (P = 0.005); $I^2 = 55\%$						
Test for overall effect: Z = 6.12 (P < 0.00001)						
**Total (95% CI)**		**2651**		**1426**	**100.0%**	**3.37 [2.29, 4.98]**
Total events	454		73			
Heterogeneity: $Tau^2 = 0.29$; $Chi^2 = 31.33$, df = 14 (P = 0.005); $I^2 = 55\%$						
Test for overall effect: Z = 6.12 (P < 0.00001)						
Test for subgroup differences: Not applicable						

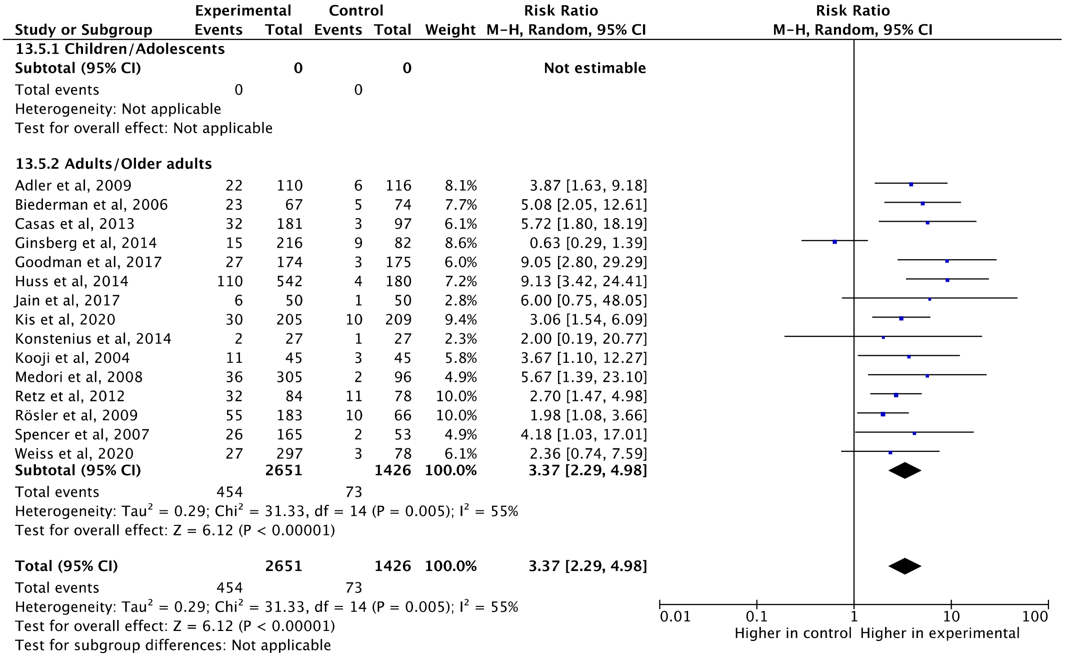

Figure legend: 95%CI, 95% confidence interval; M-H, Mantel–Haenszel. No study encompassing children/adolescents was included in the meta-analysis.

eFigure 18. Forest plot showing the risk ratio of **headache** between control and experimental groups using **methylphenidate** subdivided by age.

Study or Subgroup	Experimental Events	Experimental Total	Control Events	Control Total	Weight	Risk Ratio M-H, Fixed, 95% CI
**13.2.1 Children/Adolescents**						
Biederman et al, 2006	21	67	22	74	4.0%	1.05 [0.64, 1.74]
Brams et al, 2018	3	86	2	86	0.4%	1.50 [0.26, 8.75]
Childress et al, 2009	22	182	6	63	1.7%	1.27 [0.54, 2.99]
Childress et al, 2020A	1	65	1	54	0.2%	0.83 [0.05, 12.97]
Coghill et al, 2013 - MPH	22	111	22	110	4.3%	0.99 [0.58, 1.68]
Greenhill et al, 2002	23	155	17	161	3.2%	1.41 [0.78, 2.53]
Huang et al, 2021	9	110	1	101	0.2%	8.26 [1.07, 64.08]
Lee et al, 2011	48	157	30	157	5.8%	1.60 [1.07, 2.38]
Newcorn et al, 2008	25	219	7	74	2.0%	1.21 [0.54, 2.67]
Pearson et al, 2013	4	24	2	24	0.4%	2.00 [0.40, 9.91]
Pelham Jr et al, 2001 - IR	11	36	16	36	3.1%	0.69 [0.37, 1.27]
Pelham Jr et al, 2001 - LA	8	36	16	36	3.1%	0.50 [0.25, 1.02]
Pelham Jr et al, 2005	9	36	3	36	0.6%	3.00 [0.88, 10.18]
Pliszka et al, 2017	6	81	3	80	0.6%	1.98 [0.51, 7.63]
Ramtvedt et al, 2014 - MPH	9	34	8	34	1.5%	1.13 [0.49, 2.57]
Stein et al, 2003	17	47	16	47	3.1%	1.06 [0.61, 1.84]
Sugaya et al, 2022	3	51	3	51	0.6%	1.00 [0.21, 4.72]
Weiss et al, 2021	44	293	7	74	2.2%	1.59 [0.75, 3.38]
Wigal et al, 2004	17	90	4	42	1.1%	1.98 [0.71, 5.53]
Wilens et al, 2006	3	87	6	90	1.1%	0.52 [0.13, 2.00]
**Subtotal (95% CI)**		**1967**		**1430**	**39.2%**	**1.24 [1.04, 1.46]**
Total events	305		192			
Heterogeneity: $Chi^2 = 22.08$, df = 19 (P = 0.28); $I^2 = 14\%$						
Test for overall effect: Z = 2.47 (P = 0.01)						
**13.2.2 Adults/Older adults**						
Adler et al, 2009	28	110	16	116	3.0%	1.85 [1.06, 3.22]
Biederman et al, 2006	21	67	22	74	4.0%	1.05 [0.64, 1.74]
Casas et al, 2013	52	181	27	97	6.8%	1.03 [0.70, 1.53]
Ginsberg et al, 2014	41	216	21	82	5.9%	0.74 [0.47, 1.17]
Goodman et al, 2017	33	174	20	175	3.9%	1.66 [0.99, 2.78]
Huss et al, 2014	111	542	30	180	8.7%	1.23 [0.85, 1.77]
Jain et al, 2017	13	50	12	50	2.3%	1.08 [0.55, 2.14]
Kis et al, 2020	65	205	55	209	10.5%	1.20 [0.89, 1.63]
Konstenius et al, 2014	6	27	2	27	0.4%	3.00 [0.66, 13.56]
Kooji et al, 2004	7	45	2	45	0.4%	3.50 [0.77, 15.94]
Medori et al, 2008	64	305	17	96	5.0%	1.18 [0.73, 1.92]
Patkar et al, 2006	3	30	2	30	0.4%	1.50 [0.27, 8.34]
Retz et al, 2012	25	84	13	78	2.6%	1.79 [0.98, 3.24]
Rosenberg et al, 2014	5	29	4	29	0.8%	1.25 [0.37, 4.19]
Shram et al, 2022	5	48	2	46	0.4%	2.40 [0.49, 11.74]
Spencer et al, 2007	52	165	10	53	2.9%	1.67 [0.91, 3.05]
Weiss et al, 2020	52	297	9	78	2.8%	1.52 [0.78, 2.94]
**Subtotal (95% CI)**		**2575**		**1465**	**60.8%**	**1.29 [1.13, 1.47]**
Total events	583		264			
Heterogeneity: $Chi^2 = 16.11$, df = 16 (P = 0.45); $I^2 = 1\%$						
Test for overall effect: Z = 3.74 (P = 0.0002)						
**Total (95% CI)**		**4542**		**2895**	**100.0%**	**1.27 [1.14, 1.40]**
Total events	888		456			
Heterogeneity: $Chi^2 = 38.46$, df = 36 (P = 0.36); $I^2 = 6\%$						
Test for overall effect: Z = 4.46 (P < 0.00001)						
Test for subgroup differences: $Chi^2 = 0.14$, df = 1 (P = 0.71), $I^2 = 0\%$						

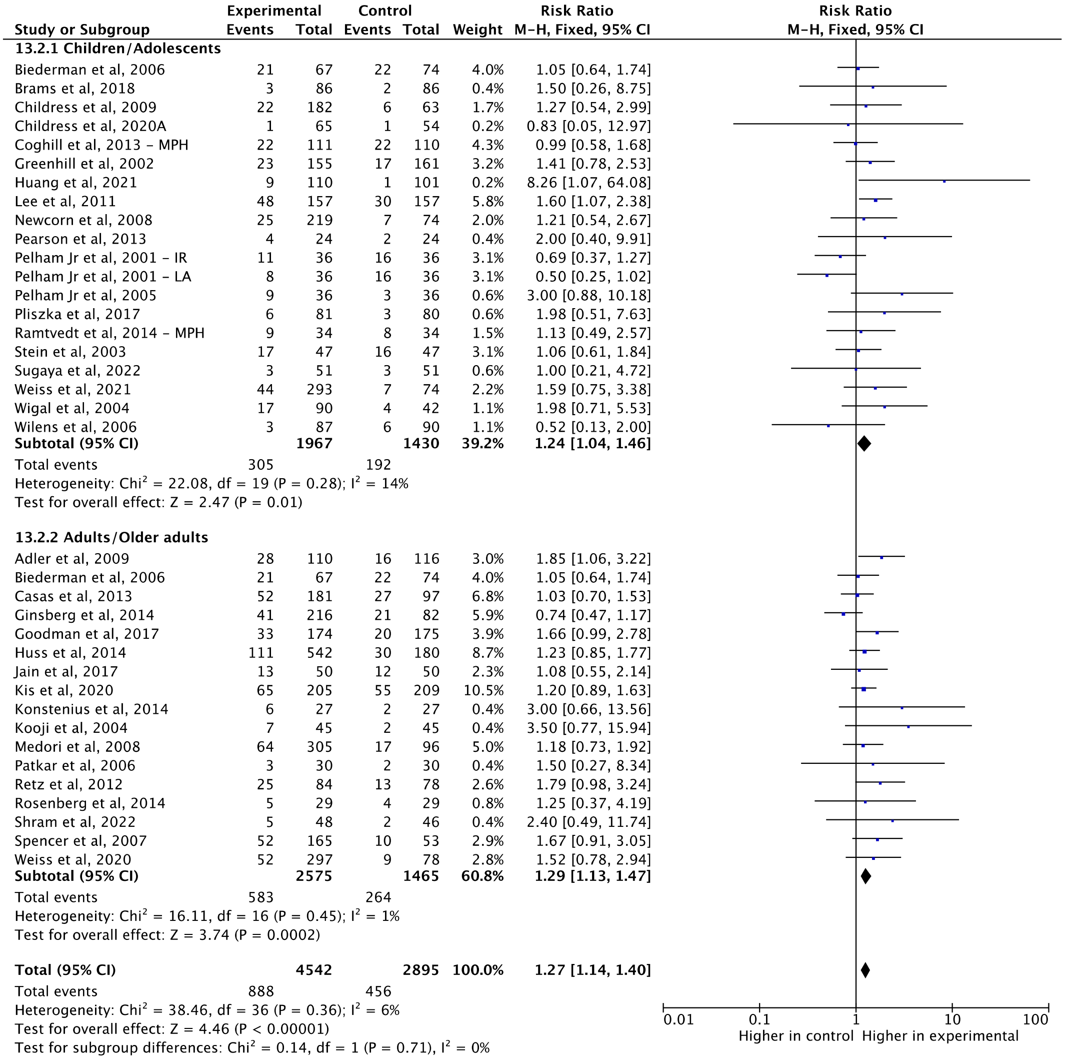

Figure legend: 95%CI, 95% confidence interval; IR, immediate release; M-H, Mantel–Haenszel; LA, long-acting; MPH, methylphenidate.

eFigure 19. Forest plot showing the risk ratio of **insomnia** between control and experimental groups using **methylphenidate** subdivided by age.

Study or Subgroup	Experimental Events	Experimental Total	Control Events	Control Total	Weight	Risk Ratio M-H, Fixed, 95% CI
**13.3.1 Children/Adolescents**						
Childress et al, 2020A	5	65	5	54	2.1%	0.83 [0.25, 2.72]
Findling et al, 2008 - MPH	13	98	4	85	1.7%	2.82 [0.96, 8.32]
Findling et al, 2008 - MTS	7	91	4	85	1.6%	1.63 [0.50, 5.39]
Froehlich et al, 2020	94	171	37	171	14.4%	2.54 [1.85, 3.48]
Greenhill et al, 2002	11	155	4	161	1.5%	2.86 [0.93, 8.78]
Lee et al, 2011	74	157	39	157	15.1%	1.90 [1.38, 2.61]
Newcorn et al, 2008	17	219	1	74	0.6%	5.74 [0.78, 42.42]
Pearson et al, 2013	12	24	5	24	1.9%	2.40 [1.00, 5.77]
Pelham Jr et al, 2005	17	36	3	36	1.2%	5.67 [1.82, 17.67]
Pliszka et al, 2017	27	81	9	80	3.5%	2.96 [1.49, 5.90]
Ramtvedt et al, 2014 - MPH	18	34	14	34	5.4%	1.29 [0.77, 2.14]
Stein et al, 2003	30	47	21	47	8.1%	1.43 [0.97, 2.10]
Weiss et al, 2021	19	293	1	74	0.6%	4.80 [0.65, 35.27]
Wigal et al, 2006 - MPH	22	162	13	160	5.1%	1.67 [0.87, 3.20]
Zheng et al, 2024	3	110	2	112	0.8%	1.53 [0.26, 8.96]
**Subtotal (95% CI)**		**1743**		**1354**	**63.6%**	**2.12 [1.81, 2.49]**
Total events	369		162			
Heterogeneity: $Chi^2 = 18.66$, df = 14 (P = 0.18); $I^2 = 25\%$						
Test for overall effect: Z = 9.21 (P < 0.00001)						
**13.3.2 Adults/Older adults**						
Adler et al, 2009	10	110	6	116	2.3%	1.76 [0.66, 4.67]
Biederman et al, 2006	12	67	4	74	1.5%	3.31 [1.12, 9.78]
Casas et al, 2013	28	181	11	97	5.6%	1.36 [0.71, 2.62]
Jain et al, 2017	11	50	4	50	1.6%	2.75 [0.94, 8.06]
Kis et al, 2020	16	205	12	209	4.6%	1.36 [0.66, 2.80]
Konstenius et al, 2014	6	27	2	27	0.8%	3.00 [0.66, 13.56]
Kooji et al, 2004	15	45	10	45	3.9%	1.50 [0.76, 2.98]
Medori et al, 2008	41	305	7	96	4.1%	1.84 [0.86, 3.97]
Patkar et al, 2006	3	30	3	30	1.2%	1.00 [0.22, 4.56]
Rösler et al, 2009	46	183	12	66	6.8%	1.38 [0.78, 2.44]
Spencer et al, 2007	27	165	6	53	3.5%	1.45 [0.63, 3.31]
Weiss et al, 2020	18	297	1	78	0.6%	4.73 [0.64, 34.87]
**Subtotal (95% CI)**		**1665**		**941**	**36.4%**	**1.69 [1.32, 2.16]**
Total events	233		78			
Heterogeneity: $Chi^2 = 5.85$, df = 11 (P = 0.88); $I^2 = 0\%$						
Test for overall effect: Z = 4.17 (P < 0.0001)						
**Total (95% CI)**		**3408**		**2295**	**100.0%**	**1.96 [1.72, 2.24]**
Total events	602		240			
Heterogeneity: $Chi^2 = 25.84$, df = 26 (P = 0.47); $I^2 = 0\%$						
Test for overall effect: Z = 9.84 (P < 0.00001)						
Test for subgroup differences: $Chi^2 = 2.33$, df = 1 (P = 0.13), $I^2 = 57.2\%$						

Risk Ratio
M-H, Fixed, 95% CI

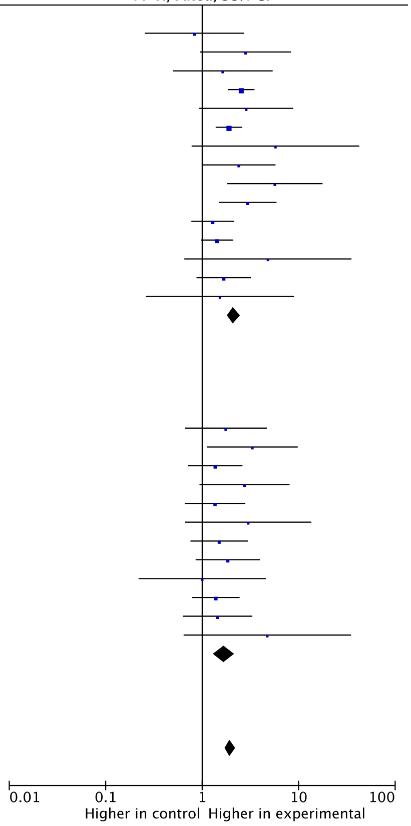

Figure legend: 95%CI, 95% confidence interval; M-H, Mantel–Haenszel; MPH, methylphenidate; MTS, transdermal system.

eFigure 20. Forest plot showing the risk ratio of **irritability** between control and experimental groups using **methylphenidate** subdivided by age.

Study or Subgroup	Experimental Events	Experimental Total	Control Events	Control Total	Weight	Risk Ratio M-H, Fixed, 95% CI
**13.4.1 Children/Adolescents**						
Chronis-Tuscano et al, 2008	3	15	1	23	0.3%	4.60 [0.53, 40.19]
Froehlich et al, 2020	77	171	67	171	25.0%	1.15 [0.90, 1.48]
Lee et al, 2011	84	157	81	157	30.3%	1.04 [0.84, 1.28]
Pearson et al, 2013	8	24	11	24	4.1%	0.73 [0.36, 1.48]
Pelham Jr et al, 2005	10	36	1	36	0.4%	10.00 [1.35, 74.12]
Ramtvedt et al, 2014 - MPH	22	34	27	34	10.1%	0.81 [0.60, 1.10]
Stein et al, 2003	33	47	27	47	10.1%	1.22 [0.90, 1.66]
Sugaya et al, 2022	22	51	19	51	7.1%	1.16 [0.72, 1.86]
Weiss et al, 2021	24	293	7	74	4.2%	0.87 [0.39, 1.93]
**Subtotal (95% CI)**		**828**		**617**	**91.5%**	**1.10 [0.97, 1.25]**
Total events	283		241			
Heterogeneity: Chi^2 = 12.68, df = 8 (P = 0.12); I^2 = 37%						
Test for overall effect: Z = 1.49 (P = 0.14)						
**13.4.2 Adults/Older adults**						
Adler et al, 2009	7	110	2	116	0.7%	3.69 [0.78, 17.38]
Jain et al, 2017	10	50	2	50	0.7%	5.00 [1.15, 21.67]
Kis et al, 2020	14	205	11	209	4.1%	1.30 [0.60, 2.79]
Medori et al, 2008	17	305	1	96	0.6%	5.35 [0.72, 39.68]
Weiss et al, 2020	16	297	4	78	2.4%	1.05 [0.36, 3.05]
**Subtotal (95% CI)**		**967**		**549**	**8.5%**	**2.03 [1.23, 3.36]**
Total events	64		20			
Heterogeneity: Chi^2 = 5.70, df = 4 (P = 0.22); I^2 = 30%						
Test for overall effect: Z = 2.76 (P = 0.006)						
**Total (95% CI)**		**1795**		**1166**	**100.0%**	**1.18 [1.04, 1.33]**
Total events	347		261			
Heterogeneity: Chi^2 = 23.60, df = 13 (P = 0.04); I^2 = 45%						
Test for overall effect: Z = 2.61 (P = 0.009)						
Test for subgroup differences: Chi^2 = 5.40, df = 1 (P = 0.02), I^2 = 81.5%						

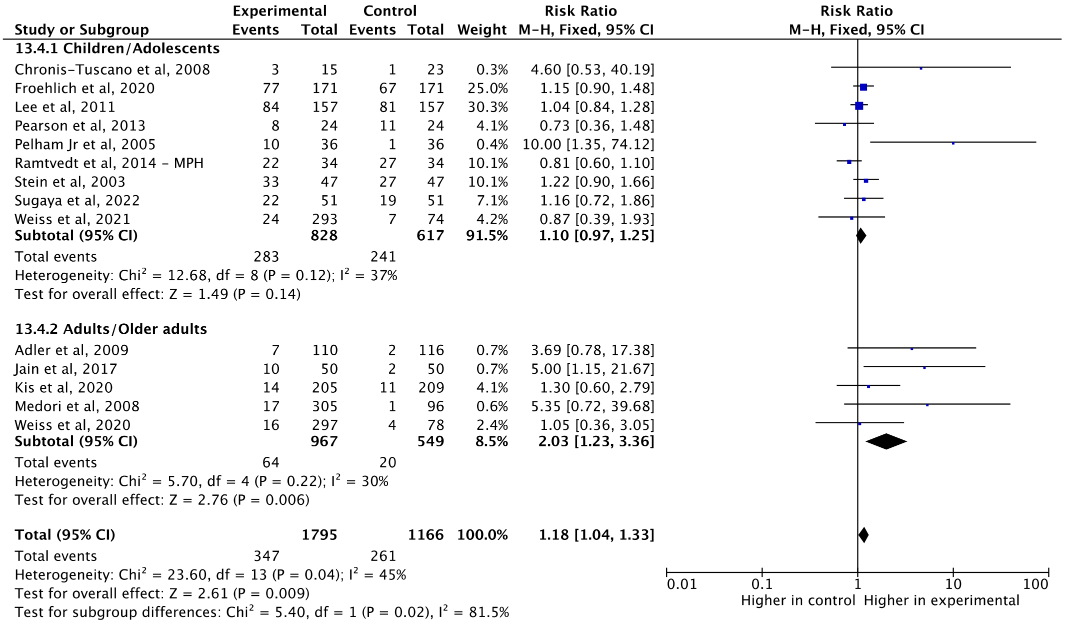

Figure legend: 95%CI, 95% confidence interval; M-H, Mantel–Haenszel; MPH, methylphenidate.

eFigure 21. Forest plot showing the risk ratio of **nausea** between control and experimental groups using **methylphenidate** subdivided by age.

Study or Subgroup	Experimental Events	Experimental Total	Control Events	Control Total	Weight	Risk Ratio M-H, Fixed, 95% CI
**13.11.1 Children/Adolescents**						
Coghill et al, 2013 - MPH	8	111	3	110	2.7%	2.64 [0.72, 9.70]
Findling et al, 2008 - MPH	12	198	4	85	5.1%	1.29 [0.43, 3.88]
Findling et al, 2008 - MTS	7	91	4	85	3.7%	1.63 [0.50, 5.39]
Newcorn et al, 2008	13	219	6	74	8.1%	0.73 [0.29, 1.86]
Pearson et al, 2013	1	24	1	24	0.9%	1.00 [0.07, 15.08]
Ramtvedt et al, 2014 - MPH	10	34	6	34	5.4%	1.67 [0.68, 4.07]
Weiss et al, 2021	17	293	3	74	4.3%	1.43 [0.43, 4.75]
Wilens et al, 2006	1	87	2	90	1.8%	0.52 [0.05, 5.60]
Zheng et al, 2024	12	110	1	112	0.9%	12.22 [1.62, 92.38]
**Subtotal (95% CI)**		**1167**		**688**	**33.0%**	**1.63 [1.10, 2.43]**
Total events	81		30			
Heterogeneity: Chi² = 8.42, df = 8 (P = 0.39); I² = 5%						
Test for overall effect: Z = 2.41 (P = 0.02)						
**13.11.2 Adults/Older adults**						
Adler et al, 2009	14	110	3	116	2.6%	4.92 [1.45, 16.66]
Casas et al, 2013	31	181	8	97	9.4%	2.08 [0.99, 4.34]
Huss et al, 2014	58	542	9	180	12.2%	2.14 [1.08, 4.23]
Jain et al, 2017	8	50	4	50	3.6%	2.00 [0.64, 6.22]
Kis et al, 2020	25	205	20	209	17.9%	1.27 [0.73, 2.22]
Medori et al, 2008	39	305	4	96	5.5%	3.07 [1.13, 8.37]
Patkar et al, 2006	2	30	1	30	0.9%	2.00 [0.19, 20.90]
Retz et al, 2012	14	84	3	78	2.8%	4.33 [1.29, 14.51]
Rosenberg et al, 2014	3	29	7	29	6.3%	0.43 [0.12, 1.50]
Rösler et al, 2009	16	183	2	66	2.7%	2.89 [0.68, 12.21]
Weiss et al, 2020	18	297	2	78	2.9%	2.36 [0.56, 9.97]
**Subtotal (95% CI)**		**2016**		**1029**	**67.0%**	**2.05 [1.54, 2.71]**
Total events	228		63			
Heterogeneity: Chi² = 13.17, df = 10 (P = 0.21); I² = 24%						
Test for overall effect: Z = 5.00 (P < 0.00001)						
**Total (95% CI)**		**3183**		**1717**	**100.0%**	**1.91 [1.52, 2.40]**
Total events	309		93			
Heterogeneity: Chi² = 22.81, df = 19 (P = 0.25); I² = 17%						
Test for overall effect: Z = 5.52 (P < 0.00001)						
Test for subgroup differences: Chi² = 0.83, df = 1 (P = 0.36), I² = 0%						

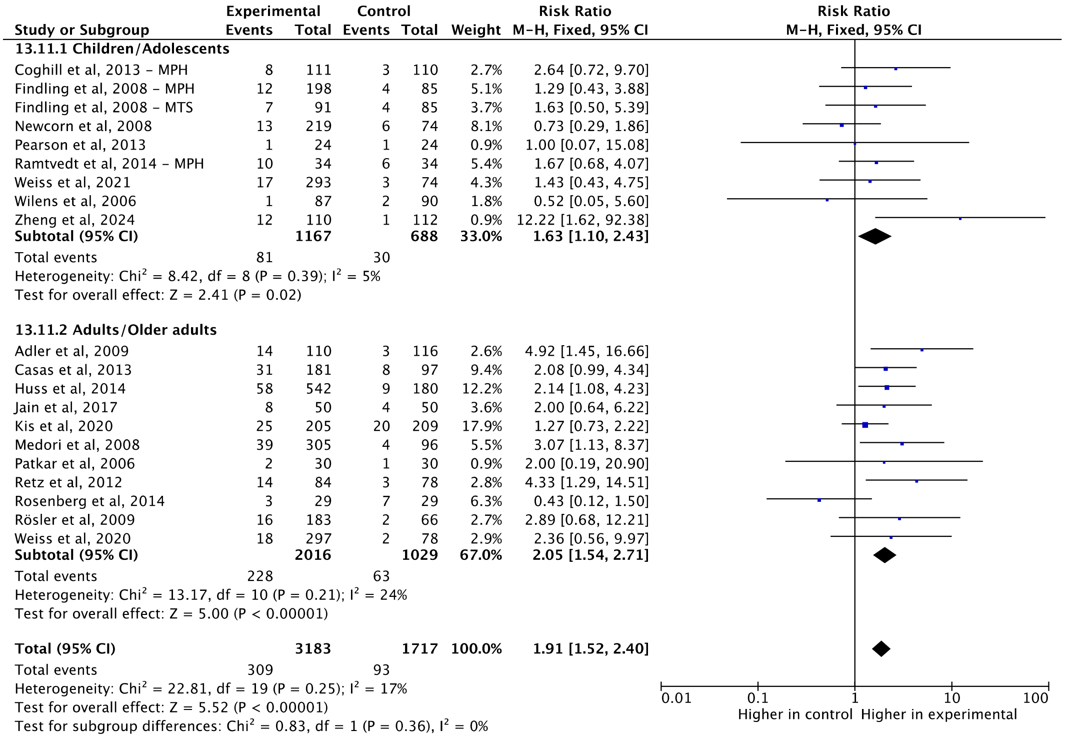

Figure legend: 95%CI, 95% confidence interval; M-H, Mantel–Haenszel; MPH, methylphenidate; MTS, transdermal system.

eFigure 22. Forest plot showing the mean differences in **heart rate** between control and experimental groups using **methylphenidate** subdivided by age.

Study or Subgroup	Experimental Mean	Experimental SD	Experimental Total	Control Mean	Control SD	Control Total	Weight	Mean Difference IV, Random, 95% CI
**13.9.1 Children/Adolescents**								
Childress et al, 2020A	1.7	11.12	65	−0.2	14.8	54	7.2%	1.90 [−2.88, 6.68]
Coghill et al, 2013 – MPH	5	12.8	222	−1.1	9.6	110	13.2%	6.10 [3.64, 8.56]
Newcorn et al, 2008	3	11.8	219	0.4	12	74	11.1%	2.60 [−0.55, 5.75]
Pliszka et al, 2017	3.5	11.49	81	3.1	11.58	80	9.9%	0.40 [−3.16, 3.96]
Zheng et al, 2024	1.1	12.42	110	−1.9	10.59	112	11.4%	3.00 [−0.04, 6.04]
**Subtotal (95% CI)**			**697**			**430**	**52.9%**	**3.10 [1.02, 5.17]**
Heterogeneity: $Tau^2 = 2.76$; $Chi^2 = 8.04$, df = 4 (P = 0.09); $I^2 = 50\%$								
Test for overall effect: Z = 2.92 (P = 0.003)								
**13.9.2 Adults/Older adults**								
Adler et al, 2009	3.6	9.78	110	−1.6	8.33	116	13.5%	5.20 [2.83, 7.57]
Biederman et al, 2006	4.5	10.5	67	−2.7	12.4	74	9.4%	7.20 [3.42, 10.98]
Hegerl et al, 2018	−6.24	14.66	22	−0.26	9.64	20	3.8%	−5.98 [−13.42, 1.46]
Jain et al, 2017	1.8	10.9	50	0.7	12.8	50	7.4%	1.10 [−3.56, 5.76]
Kis et al, 2020	3.3	13.7	205	−1.1	12.5	209	13.0%	4.40 [1.87, 6.93]
**Subtotal (95% CI)**			**454**			**469**	**47.1%**	**3.59 [0.80, 6.39]**
Heterogeneity: $Tau^2 = 6.22$; $Chi^2 = 11.94$, df = 4 (P = 0.02); $I^2 = 67\%$								
Test for overall effect: Z = 2.52 (P = 0.01)								
**Total (95% CI)**			**1151**			**899**	**100.0%**	**3.42 [1.78, 5.05]**
Heterogeneity: $Tau^2 = 3.67$; $Chi^2 = 20.82$, df = 9 (P = 0.01); $I^2 = 57\%$								
Test for overall effect: Z = 4.10 (P < 0.0001)								
Test for subgroup differences: $Chi^2 = 0.08$, df = 1 (P = 0.78), $I^2 = 0\%$								

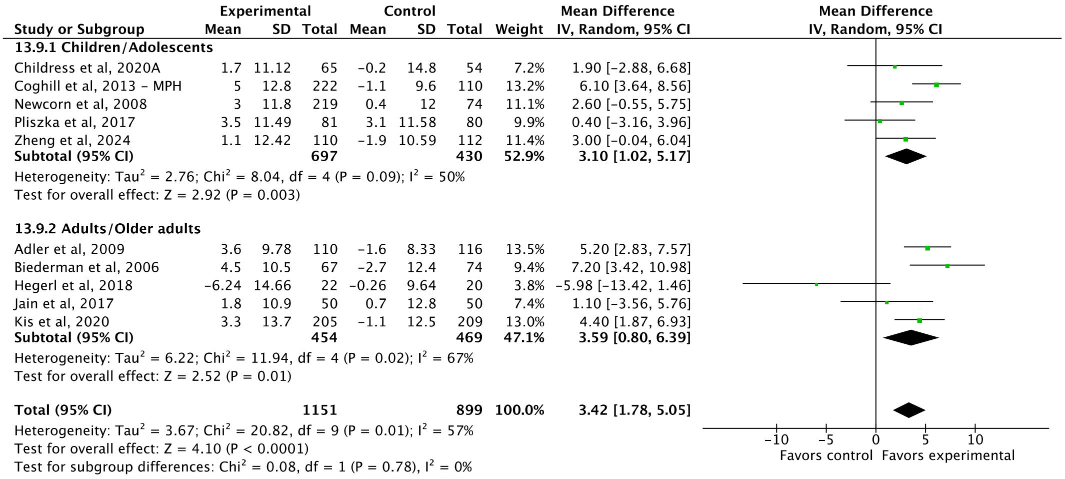

Figure legend: 95%CI, 95% confidence interval; IV, inverse variance; MPH, methylphenidate; SD, standard deviation.

eFigure 23. Forest plot showing the mean differences in **diastolic blood pressure** between control and experimental groups using **methylphenidate** subdivided by age.

	Experimental			Control				Mean Difference
**Study or Subgroup**	**Mean**	**SD**	**Total**	**Mean**	**SD**	**Total**	**Weight**	**IV, Random, 95% CI**
**13.8.1 Children/Adolescents**								
Childress et al, 2020A	5.1	8.1	65	5.9	8.27	54	9.3%	−0.80 [−3.76, 2.16]
Coghill et al, 2013 – MPH	1.7	9.9	222	1.2	8.7	110	12.0%	0.50 [−1.58, 2.58]
Newcorn et al, 2008	3.1	8.04	219	0.4	7.8	74	12.0%	2.70 [0.63, 4.77]
Pliszka et al, 2017	2.5	9.06	81	−0.4	9.99	80	9.3%	2.90 [−0.05, 5.85]
Zheng et al, 2024	0.3	8.39	110	0.1	9.29	112	11.2%	0.20 [−2.13, 2.53]
**Subtotal (95% CI)**			**697**			**430**	**53.7%**	**1.14 [−0.21, 2.49]**
Heterogeneity: $Tau^2 = 0.83$; $Chi^2 = 6.19$, df = 4 (P = 0.19); $I^2 = 35\%$								
Test for overall effect: Z = 1.66 (P = 0.10)								
**13.8.2 Adults/Older adults**								
Adler et al, 2009	1.1	6.72	110	0.4	7.43	116	12.7%	0.70 [−1.15, 2.55]
Biederman et al, 2006	4	8.5	67	−2.1	8.9	74	9.5%	6.10 [3.23, 8.97]
Hegerl et al, 2018	0.86	18.67	22	9.79	10.79	20	1.9%	−8.93 [−18.05, 0.19]
Jain et al, 2017	0	6.7	50	1.4	8.3	50	9.3%	−1.40 [−4.36, 1.56]
Kis et al, 2020	1.3	9.6	205	0.1	9.3	209	12.8%	1.20 [−0.62, 3.02]
**Subtotal (95% CI)**			**454**			**469**	**46.3%**	**0.90 [−1.79, 3.59]**
Heterogeneity: $Tau^2 = 6.58$; $Chi^2 = 19.16$, df = 4 (P = 0.0007); $I^2 = 79\%$								
Test for overall effect: Z = 0.66 (P = 0.51)								
**Total (95% CI)**			**1151**			**899**	**100.0%**	**1.12 [−0.23, 2.47]**
Heterogeneity: $Tau^2 = 2.83$; $Chi^2 = 25.36$, df = 9 (P = 0.003); $I^2 = 65\%$								
Test for overall effect: Z = 1.63 (P = 0.10)								
Test for subgroup differences: $Chi^2 = 0.02$, df = 1 (P = 0.88), $I^2 = 0\%$								

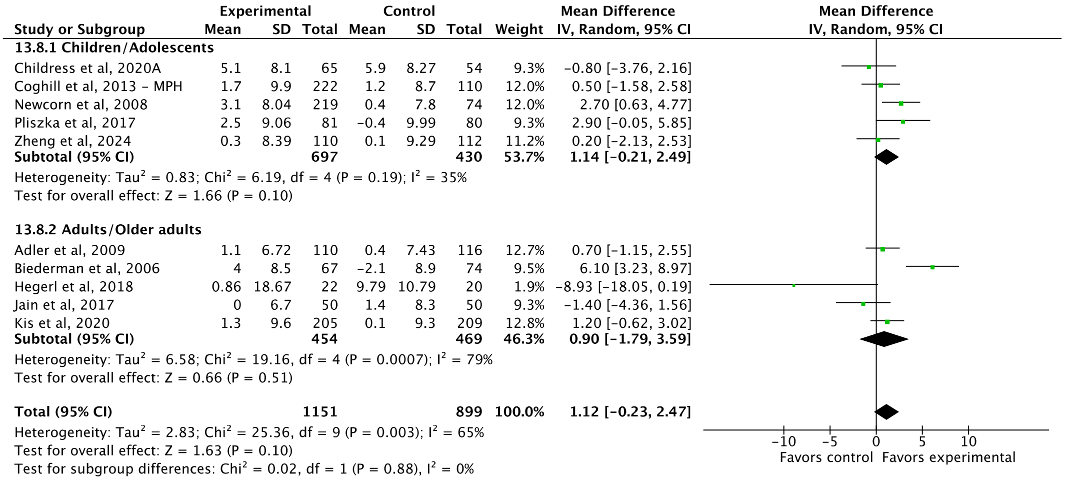

Figure legend: 95%CI, 95% confidence interval; IV, inverse variance; MPH, methylphenidate; SD, standard deviation.

eFigure 24. Forest plot showing the mean differences in **systolic blood pressure** between control and experimental groups using **methylphenidate** subdivided by age.

	Experimental			Control				Mean Difference
**Study or Subgroup**	**Mean**	**SD**	**Total**	**Mean**	**SD**	**Total**	**Weight**	**IV, Fixed, 95% CI**
**13.7.1 Children/Adolescents**								
Childress et al, 2020A	6.3	9.32	65	3.6	10.64	54	6.7%	2.70 [−0.93, 6.33]
Coghill et al, 2013 – MPH	0.3	11.1	222	1	9.6	110	16.6%	−0.70 [−3.01, 1.61]
Newcorn et al, 2008	2.4	9.7	219	1.3	8	74	17.8%	1.10 [−1.13, 3.33]
Pliszka et al, 2017	1.5	12.18	81	1.1	10.72	80	7.1%	0.40 [−3.14, 3.94]
Zheng et al, 2024	1.7	9.29	110	1.7	10.83	112	12.6%	0.00 [−2.65, 2.65]
**Subtotal (95% CI)**			**697**			**430**	**60.8%**	**0.48 [−0.73, 1.68]**
Heterogeneity: $Chi^2 = 2.86$, df = 4 (P = 0.58); $I^2 = 0\%$								
Test for overall effect: Z = 0.77 (P = 0.44)								
**13.7.2 Adults/Older adults**								
Adler et al, 2009	−1.2	8.92	110	−0.5	9.72	116	15.0%	−0.70 [−3.13, 1.73]
Biederman et al, 2006	3.5	11.8	67	−1.1	11.4	74	6.0%	4.60 [0.76, 8.44]
Hegerl et al, 2018	4.62	12.7	22	11.42	15.87	20	1.2%	−6.80 [−15.55, 1.95]
Jain et al, 2017	0.6	10.4	50	0.9	10.6	50	5.2%	−0.30 [−4.42, 3.82]
Kis et al, 2020	1.1	13.6	205	1	14.8	209	11.8%	0.10 [−2.64, 2.84]
**Subtotal (95% CI)**			**454**			**469**	**39.2%**	**0.23 [−1.28, 1.73]**
Heterogeneity: $Chi^2 = 8.10$, df = 4 (P = 0.09); $I^2 = 51\%$								
Test for overall effect: Z = 0.30 (P = 0.77)								
**Total (95% CI)**			**1151**			**899**	**100.0%**	**0.38 [−0.56, 1.32]**
Heterogeneity: $Chi^2 = 11.02$, df = 9 (P = 0.27); $I^2 = 18\%$								
Test for overall effect: Z = 0.79 (P = 0.43)								
Test for subgroup differences: $Chi^2 = 0.06$, df = 1 (P = 0.80), $I^2 = 0\%$								

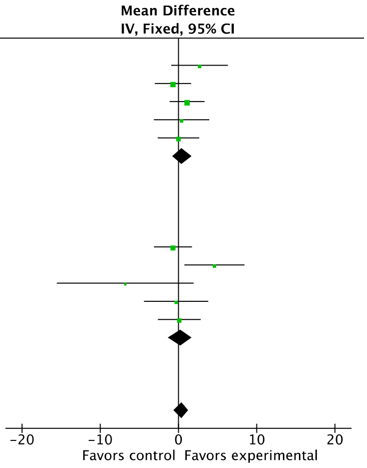

Figure legend: 95%CI, 95% confidence interval; IV, inverse variance; MPH, methylphenidate; SD, standard deviation.

eFigure 25. Forest plot showing the risk ratio of **all adverse events** between control and experimental groups using **lisdexamfetamine** subdivided by age.

Study or Subgroup	Experimental Events	Experimental Total	Control Events	Control Total	Weight	Risk Ratio M-H, Fixed, 95% CI
**14.10.1 Children/Adolescents**						
Childress et al, 2022	68	146	19	45	8.5%	1.10 [0.75, 1.62]
Coghill et al, 2013 - LDX	80	111	63	110	18.4%	1.26 [1.03, 1.53]
Coghill et al, 2014	31	78	20	79	5.8%	1.57 [0.98, 2.50]
Findling et al, 2011	160	232	45	77	19.7%	1.18 [0.96, 1.45]
**Subtotal (95% CI)**		**567**		**311**	**52.4%**	**1.24 [1.08, 1.41]**
Total events	339		147			
Heterogeneity: Chi² = 1.57, df = 3 (P = 0.67); I² = 0%						
Test for overall effect: Z = 3.16 (P = 0.002)						
**14.10.2 Adults/Older adults**						
Adler et al, 2013	62	79	47	80	13.6%	1.34 [1.08, 1.66]
Ermer et al, 2019	20	26	1	6	0.5%	4.62 [0.76, 27.96]
Jasinski et al, 2009	15	37	6	36	1.8%	2.43 [1.06, 5.57]
Martin et al, 2014A	18	24	2	7	0.9%	2.63 [0.80, 8.66]
Martin et al, 2014B - LDX	12	18	8	17	2.4%	1.42 [0.78, 2.58]
McElroy et al, 2015	166	196	37	63	16.3%	1.44 [1.16, 1.79]
Wigal et al, 2010 - LDX	32	115	42	117	12.1%	0.78 [0.53, 1.13]
**Subtotal (95% CI)**		**495**		**326**	**47.6%**	**1.33 [1.16, 1.53]**
Total events	325		143			
Heterogeneity: Chi² = 13.41, df = 6 (P = 0.04); I² = 55%						
Test for overall effect: Z = 3.96 (P < 0.0001)						
**Total (95% CI)**		**1062**		**637**	**100.0%**	**1.28 [1.16, 1.41]**
Total events	664		290			
Heterogeneity: Chi² = 15.67, df = 10 (P = 0.11); I² = 36%						
Test for overall effect: Z = 5.04 (P < 0.00001)						
Test for subgroup differences: Chi² = 0.54, df = 1 (P = 0.46), I² = 0%						

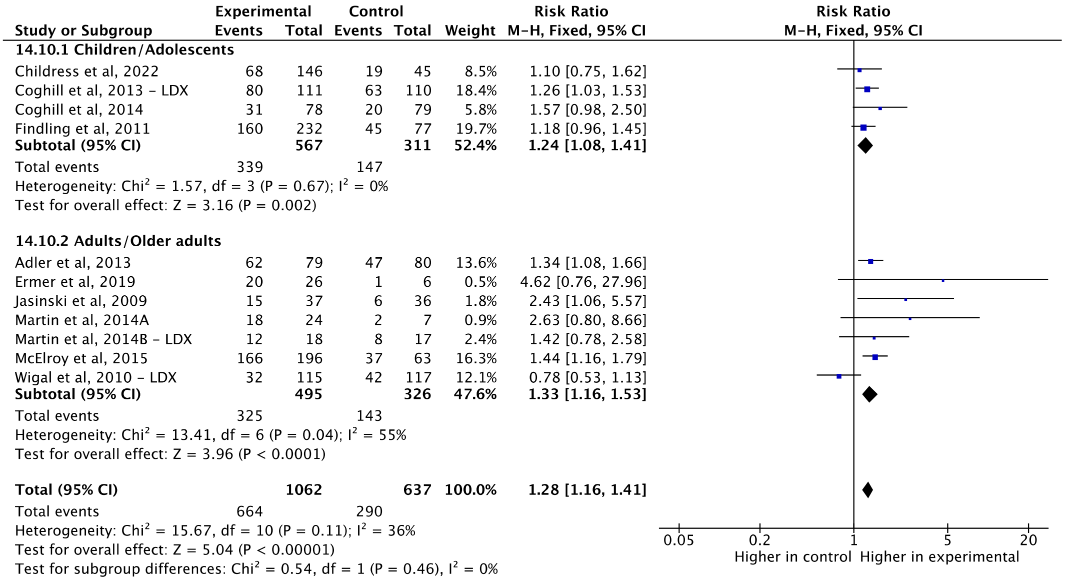

Figure legend: 95%CI, 95% confidence interval; LDX, lisdexamfetamine; M-H, Mantel–Haenszel.

eFigure 26. Forest plot showing the risk ratio of **anxiety** between control and experimental groups using **lisdexamfetamine** subdivided by age.

Study or Subgroup	Experimental Events	Experimental Total	Control Events	Control Total	Weight	Risk Ratio M-H, Random, 95% CI
**14.6.1 Children/Adolescents**						
**Subtotal (95% CI)**		**0**		**0**		**Not estimable**
Total events	0		0			
Heterogeneity: Not applicable						
Test for overall effect: Not applicable						
**14.6.2 Adults/Older adults**						
McElroy et al, 2015	9	196	5	63	55.9%	0.58 [0.20, 1.66]
Mooney et al, 2015	7	22	1	21	44.1%	6.68 [0.90, 49.78]
**Subtotal (95% CI)**		**218**		**84**	**100.0%**	**1.70 [0.14, 20.21]**
Total events	16		6			
Heterogeneity: $Tau^2 = 2.56$; $Chi^2 = 4.82$, df = 1 (P = 0.03); $I^2 = 79\%$						
Test for overall effect: Z = 0.42 (P = 0.67)						
**Total (95% CI)**		**218**		**84**	**100.0%**	**1.70 [0.14, 20.21]**
Total events	16		6			
Heterogeneity: $Tau^2 = 2.56$; $Chi^2 = 4.82$, df = 1 (P = 0.03); $I^2 = 79\%$						
Test for overall effect: Z = 0.42 (P = 0.67)						
Test for subgroup differences: Not applicable						

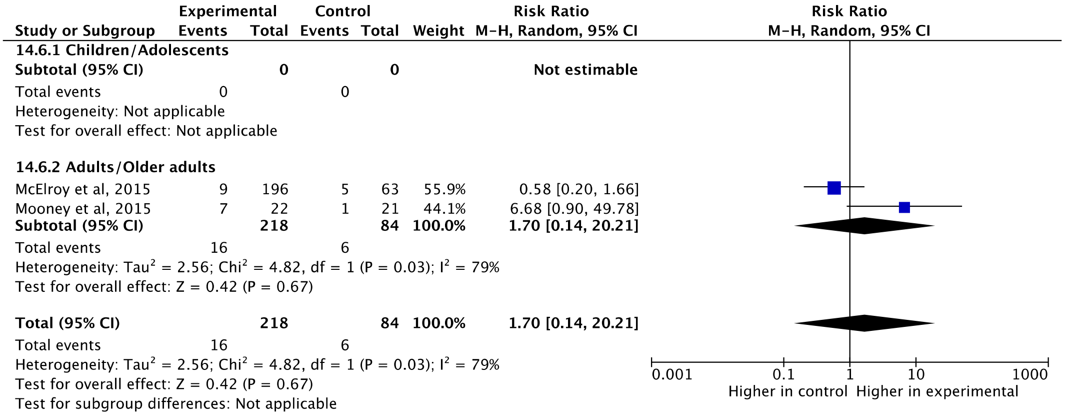

Figure legend: 95%CI, 95% confidence interval; LDX, lisdexamfetamine; M-H, Mantel–Haenszel. No study encompassing children/adolescents was included in the meta-analysis.

eFigure 27. Forest plot showing the risk ratio of **decreased appetite** between control and experimental groups using **lisdexamfetamine** subdivided by age.

	Experimental		Control			Risk Ratio
**Study or Subgroup**	**Events**	**Total**	**Events**	**Total**	**Weight**	**M-H, Random, 95% CI**
**14.1.1 Children/Adolescents**						
Childress et al, 2022	20	146	4	45	16.6%	1.54 [0.56, 4.27]
Coghill et al, 2013 - LDX	28	111	3	110	15.1%	9.25 [2.90, 29.54]
Findling et al, 2011	79	232	2	77	13.0%	13.11 [3.30, 52.08]
Wigal et al, 2010 - LDX	4	115	2	117	10.5%	2.03 [0.38, 10.89]
**Subtotal (95% CI)**		**604**		**349**	**55.3%**	**4.41 [1.38, 14.09]**
Total events	131		11			
Heterogeneity: Tau² = 0.96; Chi² = 9.85, df = 3 (P = 0.02); I² = 70%						
Test for overall effect: Z = 2.51 (P = 0.01)						
**14.1.2 Adults/Older adults**						
Martin et al, 2014B - LDX	3	18	1	17	7.6%	2.83 [0.33, 24.66]
McElroy et al, 2015	42	196	4	63	17.0%	3.38 [1.26, 9.04]
Mooney et al, 2015	12	22	7	21	20.1%	1.64 [0.80, 3.35]
**Subtotal (95% CI)**		**236**		**101**	**44.7%**	**2.14 [1.23, 3.75]**
Total events	57		12			
Heterogeneity: Tau² = 0.00; Chi² = 1.66, df = 2 (P = 0.44); I² = 0%						
Test for overall effect: Z = 2.67 (P = 0.008)						
**Total (95% CI)**		**840**		**450**	**100.0%**	**3.33 [1.63, 6.80]**
Total events	188		23			
Heterogeneity: Tau² = 0.53; Chi² = 15.35, df = 6 (P = 0.02); I² = 61%						
Test for overall effect: Z = 3.30 (P = 0.0010)						
Test for subgroup differences: Chi² = 1.21, df = 1 (P = 0.27), I² = 17.3%						

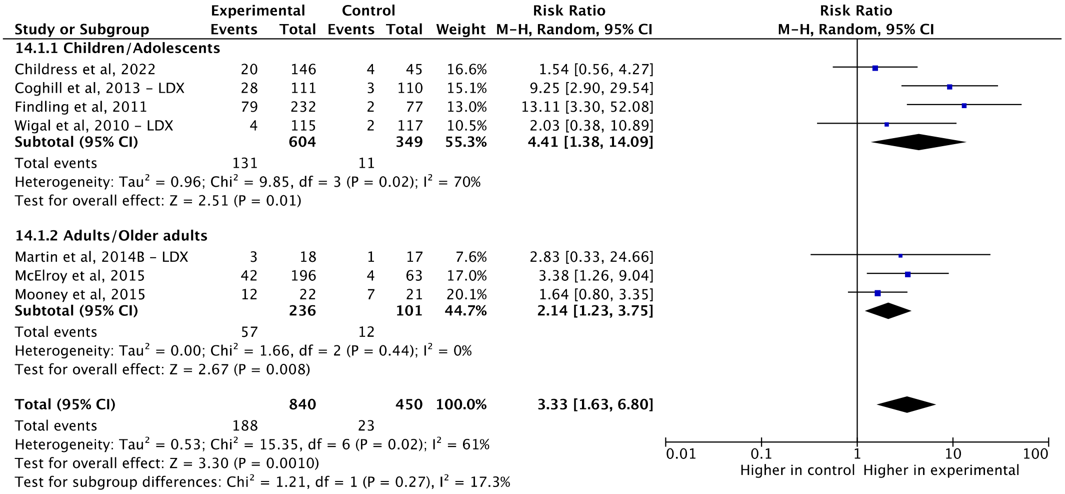

Figure legend: 95%CI, 95% confidence interval; LDX, lisdexamfetamine; M-H, Mantel–Haenszel.

eFigure 28. Forest plot showing the risk ratio of **headache** between control and experimental groups using **lisdexamfetamine** subdivided by age.

Study or Subgroup	Experimental Events	Experimental Total	Control Events	Control Total	Weight	Risk Ratio M-H, Fixed, 95% CI
**14.2.1 Children/Adolescents**						
Coghill et al, 2013 - LDX	16	111	22	110	36.7%	0.72 [0.40, 1.30]
Coghill et al, 2014	6	78	5	79	8.2%	1.22 [0.39, 3.82]
Findling et al, 2011	34	232	10	77	24.9%	1.13 [0.59, 2.18]
**Subtotal (95% CI)**		**421**		**266**	**69.8%**	**0.92 [0.62, 1.39]**
Total events	56		37			
Heterogeneity: $Chi^2 = 1.26$, df = 2 (P = 0.53); $I^2 = 0\%$						
Test for overall effect: Z = 0.38 (P = 0.71)						
**14.2.2 Adults/Older adults**						
Jasinski et al, 2009	6	37	1	36	1.7%	5.84 [0.74, 46.11]
Martin et al, 2014B - LDX	1	18	3	17	5.1%	0.31 [0.04, 2.74]
McElroy et al, 2015	23	196	6	63	15.1%	1.23 [0.53, 2.89]
Mooney et al, 2015	10	22	2	21	3.4%	4.77 [1.18, 19.27]
Wigal et al, 2010 - LDX	2	115	3	117	4.9%	0.68 [0.12, 3.98]
**Subtotal (95% CI)**		**388**		**254**	**30.2%**	**1.64 [0.94, 2.88]**
Total events	42		15			
Heterogeneity: $Chi^2 = 7.32$, df = 4 (P = 0.12); $I^2 = 45\%$						
Test for overall effect: Z = 1.73 (P = 0.08)						
**Total (95% CI)**		**809**		**520**	**100.0%**	**1.14 [0.82, 1.58]**
Total events	98		52			
Heterogeneity: $Chi^2 = 10.52$, df = 7 (P = 0.16); $I^2 = 33\%$						
Test for overall effect: Z = 0.79 (P = 0.43)						
Test for subgroup differences: $Chi^2 = 2.63$, df = 1 (P = 0.10), $I^2 = 62.0\%$						

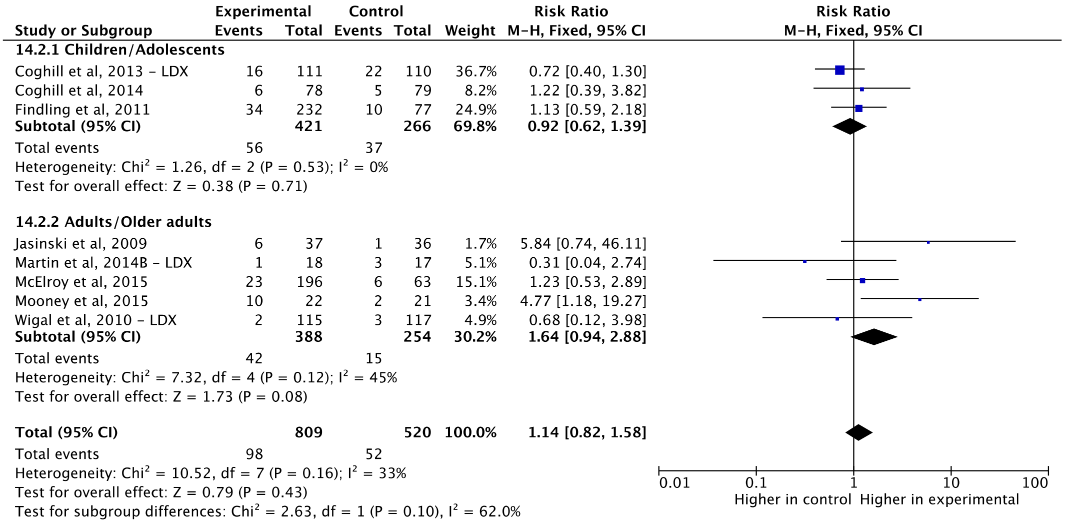

Figure legend: 95%CI, 95% confidence interval; LDX, lisdexamfetamine; M-H, Mantel–Haenszel.

eFigure 29. Forest plot showing the risk ratio of **insomnia** between control and experimental groups using **lisdexamfetamine** subdivided by age.

Study or Subgroup	Experimental Events	Experimental Total	Control Events	Control Total	Weight	Risk Ratio M-H, Fixed, 95% CI
**14.3.1 Children/Adolescents**						
Findling et al, 2011	26	232	3	77	28.0%	2.88 [0.90, 9.24]
**Subtotal (95% CI)**		**232**		**77**	**28.0%**	**2.88 [0.90, 9.24]**
Total events	26		3			
Heterogeneity: Not applicable						
Test for overall effect: Z = 1.77 (P = 0.08)						
**14.3.2 Adults/Older adults**						
McElroy et al, 2015	26	196	5	63	47.0%	1.67 [0.67, 4.17]
Mooney et al, 2015	7	22	2	21	12.7%	3.34 [0.78, 14.29]
Wigal et al, 2010 - LDX	3	115	2	117	12.3%	1.53 [0.26, 8.96]
**Subtotal (95% CI)**		**333**		**201**	**72.0%**	**1.94 [0.96, 3.91]**
Total events	36		9			
Heterogeneity: $Chi^2 = 0.71$, df = 2 (P = 0.70); $I^2 = 0\%$						
Test for overall effect: Z = 1.85 (P = 0.06)						
**Total (95% CI)**		**565**		**278**	**100.0%**	**2.20 [1.21, 4.03]**
Total events	62		12			
Heterogeneity: $Chi^2 = 1.03$, df = 3 (P = 0.79); $I^2 = 0\%$						
Test for overall effect: Z = 2.57 (P = 0.01)						
Test for subgroup differences: $Chi^2 = 0.32$, df = 1 (P = 0.57), $I^2 = 0\%$						

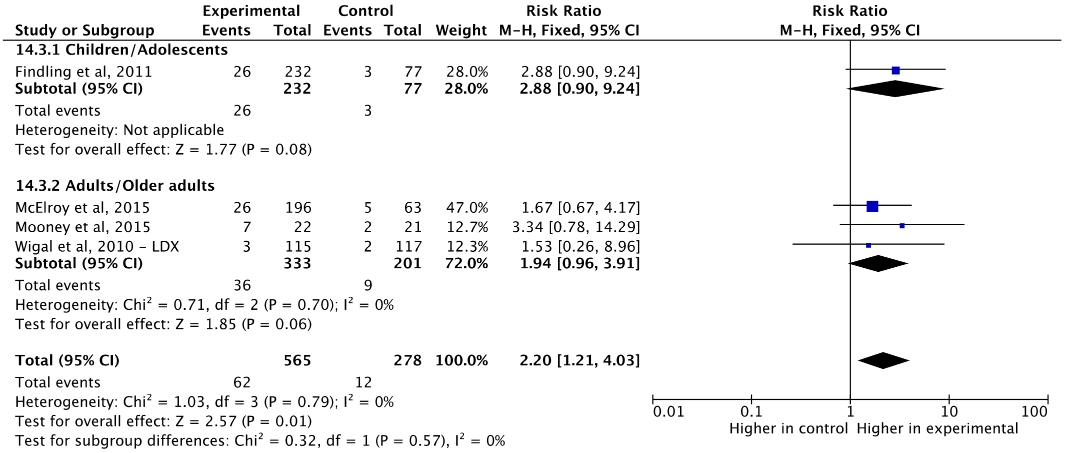

Figure legend: 95%CI, 95% confidence interval; LDX, lisdexamfetamine; M-H, Mantel–Haenszel.

eFigure 30. Forest plot showing the risk ratio of **nausea** between control and experimental groups using **lisdexamfetamine** subdivided by age.

Study or Subgroup	Experimental Events	Experimental Total	Control Events	Control Total	Weight	Risk Ratio M-H, Fixed, 95% CI
**14.11.1 Children/Adolescents**						
Coghill et al, 2013 - LDX	12	111	3	110	38.7%	3.96 [1.15, 13.66]
Findling et al, 2011	9	232	2	77	38.5%	1.49 [0.33, 6.76]
**Subtotal (95% CI)**		**343**		**187**	**77.2%**	**2.73 [1.08, 6.93]**
Total events	21		5			
Heterogeneity: Chi² = 0.96, df = 1 (P = 0.33); I² = 0%						
Test for overall effect: Z = 2.11 (P = 0.03)						
**14.11.2 Adults/Older adults**						
McElroy et al, 2015	15	196	0	63	9.7%	10.07 [0.61, 165.95]
Mooney et al, 2015	5	22	1	21	13.1%	4.77 [0.61, 37.52]
**Subtotal (95% CI)**		**218**		**84**	**22.8%**	**7.02 [1.25, 39.53]**
Total events	20		1			
Heterogeneity: Chi² = 0.20, df = 1 (P = 0.66); I² = 0%						
Test for overall effect: Z = 2.21 (P = 0.03)						
**Total (95% CI)**		**561**		**271**	**100.0%**	**3.71 [1.63, 8.44]**
Total events	41		6			
Heterogeneity: Chi² = 1.95, df = 3 (P = 0.58); I² = 0%						
Test for overall effect: Z = 3.13 (P = 0.002)						
Test for subgroup differences: Chi² = 0.89, df = 1 (P = 0.35), I² = 0%						

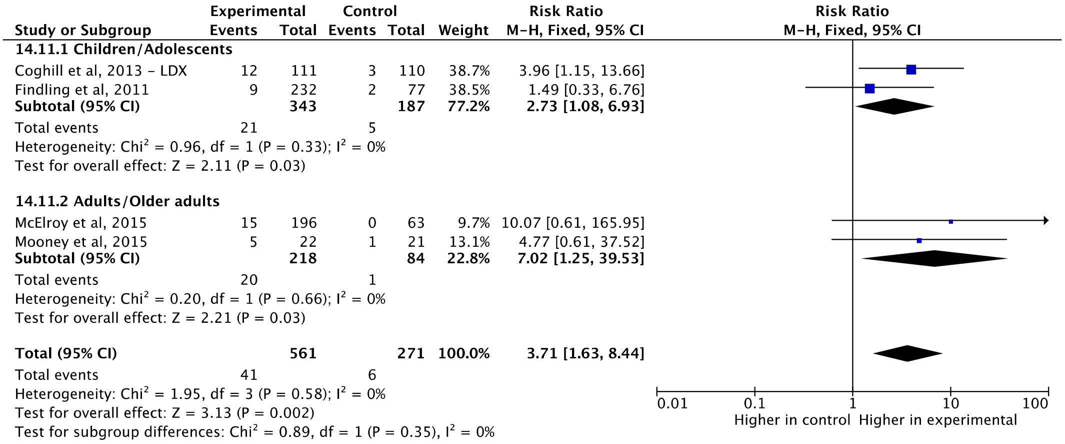

Figure legend: 95%CI, 95% confidence interval; LDX, lisdexamfetamine; M-H, Mantel–Haenszel.

eFigure 31. Forest plot showing the mean differences in **heart rate** between control and experimental groups using **lisdexamfetamine** subdivided by age.

Study or Subgroup	Experimental Mean	Experimental SD	Experimental Total	Control Mean	Control SD	Control Total	Weight	Mean Difference IV, Random, 95% CI
**14.9.1 Children/Adolescents**								
Childress et al, 2022	2.7	10.79	146	1.9	9.9	45	13.5%	0.80 [−2.58, 4.18]
Coghill et al, 2013 - LDX	5.7	15.3	222	−1.1	9.6	110	17.6%	6.80 [4.10, 9.50]
Coghill et al, 2014	6.1	12.59	78	2.3	9.29	79	13.0%	3.80 [0.34, 7.26]
Findling et al, 2011	5.4	1.27	78	0.8	1.36	77	37.3%	4.60 [4.19, 5.01]
**Subtotal (95% CI)**			**524**			**311**	**81.5%**	**4.29 [2.45, 6.13]**
Heterogeneity: $Tau^2 = 2.01$; $Chi^2 = 7.61$, df = 3 (P = 0.05); $I^2 = 61\%$								
Test for overall effect: Z = 4.57 (P < 0.00001)								
**14.9.2 Adults/Older adults**								
McElroy et al, 2015	3.8	11.57	196	1	8.1	63	18.5%	2.80 [0.23, 5.37]
**Subtotal (95% CI)**			**196**			**63**	**18.5%**	**2.80 [0.23, 5.37]**
Heterogeneity: Not applicable								
Test for overall effect: Z = 2.13 (P = 0.03)								
**Total (95% CI)**			**720**			**374**	**100.0%**	**4.04 [2.50, 5.58]**
Heterogeneity: $Tau^2 = 1.61$; $Chi^2 = 9.41$, df = 4 (P = 0.05); $I^2 = 57\%$								
Test for overall effect: Z = 5.14 (P < 0.00001)								
Test for subgroup differences: $Chi^2 = 0.85$, df = 1 (P = 0.36), $I^2 = 0\%$								

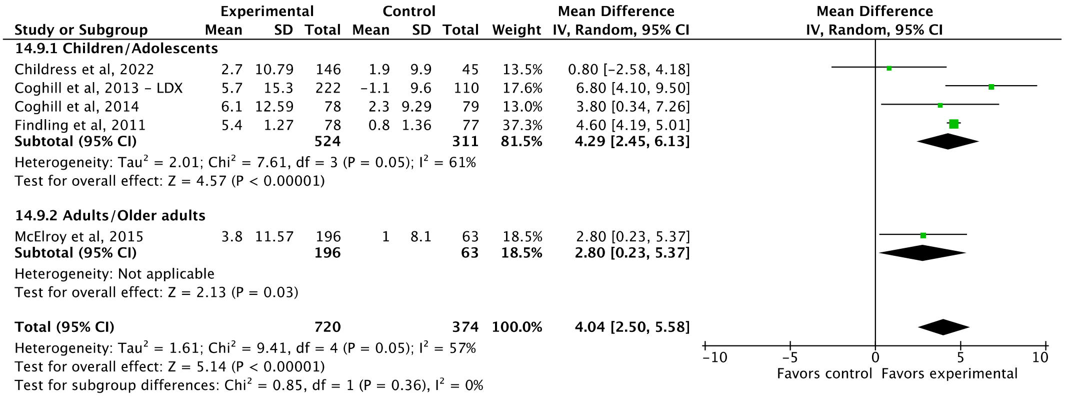

Figure legend: 95%CI, 95% confidence interval; IV, inverse variance; LDX, lisdexamfetamine; SD, standard deviation.

eFigure 32. Forest plot showing the mean differences in **diastolic blood pressure** between control and experimental groups using **lisdexamfetamine** subdivided by age.

Study or Subgroup	Experimental Mean	Experimental SD	Experimental Total	Control Mean	Control SD	Control Total	Weight	Mean Difference IV, Random, 95% CI
**14.8.1 Children/Adolescents**								
Childress et al, 2022	1.7	5.9	146	0	6.88	45	18.9%	1.70 [−0.53, 3.93]
Coghill et al, 2013 - LDX	0.2	9.6	222	1.2	8.7	110	19.7%	−1.00 [−3.06, 1.06]
Coghill et al, 2014	1.4	11.27	78	−0.1	8.12	79	15.1%	1.50 [−1.58, 4.58]
Findling et al, 2011	3.4	0.8	78	0.5	0.97	77	26.0%	2.90 [2.62, 3.18]
**Subtotal (95% CI)**			**524**			**311**	**79.8%**	**1.40 [−0.57, 3.37]**
Heterogeneity: $Tau^2 = 3.01$; $Chi^2 = 15.20$, df = 3 (P = 0.002); $I^2 = 80\%$								
Test for overall effect: Z = 1.39 (P = 0.16)								
**14.8.2 Adults/Older adults**								
McElroy et al, 2015	−0.7	7.32	196	−0.5	6.8	63	20.2%	−0.20 [−2.17, 1.77]
**Subtotal (95% CI)**			**196**			**63**	**20.2%**	**−0.20 [−2.17, 1.77]**
Heterogeneity: Not applicable								
Test for overall effect: Z = 0.20 (P = 0.84)								
**Total (95% CI)**			**720**			**374**	**100.0%**	**1.07 [−0.77, 2.91]**
Heterogeneity: $Tau^2 = 3.37$; $Chi^2 = 23.97$, df = 4 (P < 0.0001); $I^2 = 83\%$								
Test for overall effect: Z = 1.14 (P = 0.26)								
Test for subgroup differences: $Chi^2 = 1.27$, df = 1 (P = 0.26), $I^2 = 21.1\%$								

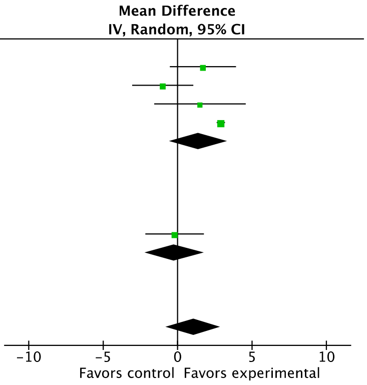

Figure legend: 95%CI, 95% confidence interval; IV, inverse variance; LDX, lisdexamfetamine; SD, standard deviation.

eFigure 33. Forest plot showing the mean differences in **systolic blood pressure** between control and experimental groups using **lisdexamfetamine** subdivided by age.

Study or Subgroup	Experimental Mean	Experimental SD	Experimental Total	Control Mean	Control SD	Control Total	Weight	Mean Difference IV, Fixed, 95% CI
**14.7.1 Children/Adolescents**								
Childress et al, 2022	1	7.51	146	0.3	6.06	45	2.5%	0.70 [−1.45, 2.85]
Coghill et al, 2013 - LDX	1	9.8	222	1	9.6	110	2.4%	0.00 [−2.21, 2.21]
Coghill et al, 2014	1.5	11.79	78	−0.6	9.66	79	1.0%	2.10 [−1.27, 5.47]
Findling et al, 2011	1.7	1.21	78	2.2	1.04	77	92.2%	−0.50 [−0.86, −0.14]
**Subtotal (95% CI)**			**524**			**311**	**98.1%**	**−0.43 [−0.77, −0.09]**
Heterogeneity: Chi² = 3.52, df = 3 (P = 0.32); I² = 15%								
Test for overall effect: Z = 2.45 (P = 0.01)								
**14.7.2 Adults/Older adults**								
McElroy et al, 2015	0.1	9.85	196	−1.5	8.41	63	1.9%	1.60 [−0.89, 4.09]
**Subtotal (95% CI)**			**196**			**63**	**1.9%**	**1.60 [−0.89, 4.09]**
Heterogeneity: Not applicable								
Test for overall effect: Z = 1.26 (P = 0.21)								
**Total (95% CI)**			**720**			**374**	**100.0%**	**−0.39 [−0.73, −0.05]**
Heterogeneity: Chi² = 6.02, df = 4 (P = 0.20); I² = 34%								
Test for overall effect: Z = 2.25 (P = 0.02)								
Test for subgroup differences: Chi² = 2.50, df = 1 (P = 0.11), I² = 60.0%								

Mean Difference
IV, Fixed, 95% CI

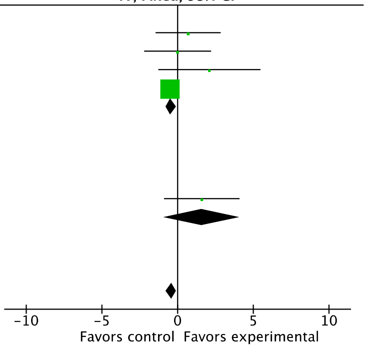

Figure legend: 95%CI, 95% confidence interval; IV, inverse variance; LDX, lisdexamfetamine; SD, standard deviation.

eFigure 34. Forest plot showing the risk ratio of **all adverse events** between control and experimental groups using **amphetamines** subdivided by age.

Study or Subgroup	Experimental Events	Experimental Total	Control Events	Control Total	Weight	Risk Ratio M-H, Random, 95% CI
**15.10.1 Children/Adolescents**						
Brams et al, 2018	70	131	34	131	13.1%	2.06 [1.48, 2.87]
Childress et al, 2015	10	97	6	97	3.1%	1.67 [0.63, 4.41]
Childress et al, 2017	9	51	6	48	3.2%	1.41 [0.54, 3.67]
Cutler et al, 2022B	44	105	43	105	13.4%	1.02 [0.74, 1.41]
Mattingly et al, 2020	11	45	7	43	3.9%	1.50 [0.64, 3.51]
**Subtotal (95% CI)**		**429**		**424**	**36.7%**	**1.47 [1.01, 2.16]**
Total events	144		96			
Heterogeneity: $Tau^2 = 0.09$; $Chi^2 = 8.98$, df = 4 (P = 0.06); $I^2 = 55\%$						
Test for overall effect: Z = 2.00 (P = 0.05)						
**15.10.2 Adults/Older adults**						
Cutler et al, 2022A	54	62	35	65	16.3%	1.62 [1.27, 2.07]
Martin et al, 2014B - AMPH	9	17	8	17	5.6%	1.13 [0.57, 2.21]
Nujiten et al, 2016	28	38	16	35	10.7%	1.61 [1.07, 2.42]
Spencer et al, 2008	122	137	86	135	20.2%	1.40 [1.22, 1.61]
Weisler et al, 2017	104	182	19	89	10.5%	2.68 [1.76, 4.07]
**Subtotal (95% CI)**		**436**		**341**	**63.3%**	**1.63 [1.28, 2.07]**
Total events	317		164			
Heterogeneity: $Tau^2 = 0.04$; $Chi^2 = 11.50$, df = 4 (P = 0.02); $I^2 = 65\%$						
Test for overall effect: Z = 3.98 (P < 0.0001)						
**Total (95% CI)**		**865**		**765**	**100.0%**	**1.56 [1.30, 1.88]**
Total events	461		260			
Heterogeneity: $Tau^2 = 0.04$; $Chi^2 = 19.77$, df = 9 (P = 0.02); $I^2 = 54\%$						
Test for overall effect: Z = 4.79 (P < 0.00001)						
Test for subgroup differences: $Chi^2 = 0.18$, df = 1 (P = 0.67), $I^2 = 0\%$						

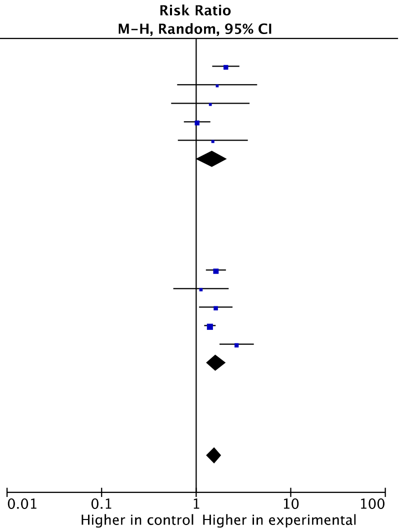

Figure legend: 95%CI, 95% confidence interval; AMPH, amphetamines; M-H, Mantel–Haenszel.

eFigure 35. Forest plot showing the risk ratio of **anxiety** between control and experimental groups using **amphetamines** subdivided by age.

Study or Subgroup	Experimental Events	Experimental Total	Control Events	Control Total	Weight	Risk Ratio M-H, Fixed, 95% CI
**15.6.1 Children/Adolescents**						
McCracken et al, 2003	11	48	10	49	30.7%	1.12 [0.53, 2.40]
McCracken et al, 2003 - XR	9	49	10	49	31.0%	0.90 [0.40, 2.02]
Ramtvedt et al, 2014 - AMPH	8	34	7	34	21.7%	1.14 [0.47, 2.80]
**Subtotal (95% CI)**		**131**		**132**	**83.4%**	**1.05 [0.65, 1.67]**
Total events	28		27			
Heterogeneity: Chi² = 0.20, df = 2 (P = 0.90); I² = 0%						
Test for overall effect: Z = 0.18 (P = 0.85)						
**15.6.2 Adults/Older adults**						
Spencer et al, 2008	9	137	4	135	12.5%	2.22 [0.70, 7.03]
Weisler et al, 2017	10	182	1	89	4.2%	4.89 [0.64, 37.61]
**Subtotal (95% CI)**		**319**		**224**	**16.6%**	**2.89 [1.05, 7.91]**
Total events	19		5			
Heterogeneity: Chi² = 0.46, df = 1 (P = 0.50); I² = 0%						
Test for overall effect: Z = 2.06 (P = 0.04)						
**Total (95% CI)**		**450**		**356**	**100.0%**	**1.35 [0.89, 2.06]**
Total events	47		32			
Heterogeneity: Chi² = 3.57, df = 4 (P = 0.47); I² = 0%						
Test for overall effect: Z = 1.40 (P = 0.16)						
Test for subgroup differences: Chi² = 3.20, df = 1 (P = 0.07), I² = 68.8%						

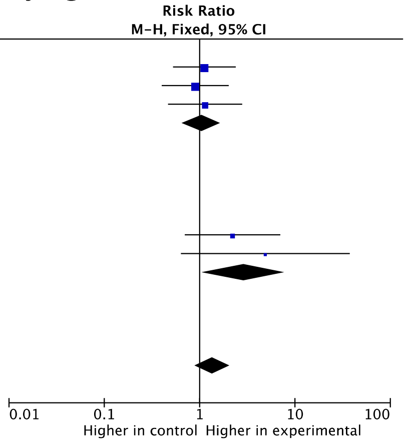

Figure legend: 95%CI, 95% confidence interval; AMPH, amphetamines; M-H, Mantel–Haenszel; XR, extended release.

eFigure 36. Forest plot showing the risk ratio of **decreased appetite** between control and experimental groups using **amphetamines** subdivided by age.

Study or Subgroup	Experimental Events	Experimental Total	Control Events	Control Total	Weight	Risk Ratio M-H, Fixed, 95% CI
**15.1.1 Children/Adolescents**						
Brams et al, 2018	40	132	9	131	8.5%	4.41 [2.23, 8.72]
Cutler et al, 2022B	13	105	2	105	1.9%	6.50 [1.50, 28.10]
Mattingly et al, 2020	1	45	2	43	1.9%	0.48 [0.04, 5.08]
McCracken et al, 2003	22	48	11	49	10.2%	2.04 [1.12, 3.74]
McCracken et al, 2003	22	48	11	49	10.2%	2.04 [1.12, 3.74]
McCracken et al, 2003 - XR	27	49	11	49	10.3%	2.45 [1.38, 4.38]
McCracken et al, 2003 - XR	27	49	11	49	10.3%	2.45 [1.38, 4.38]
Ramtvedt et al, 2014 - AMPH	24	34	17	34	16.0%	1.41 [0.95, 2.11]
Spencer et al, 2006	83	226	1	52	1.5%	19.10 [2.72, 134.04]
**Subtotal (95% CI)**		**736**		**561**	**71.0%**	**2.75 [2.17, 3.48]**
Total events	259		75			
Heterogeneity: $Chi^2 = 21.87$, df = 8 (P = 0.005); $I^2 = 63\%$						
Test for overall effect: Z = 8.42 (P < 0.00001)						
**15.1.2 Adults/Older adults**						
Cutler et al, 2022A	30	62	14	65	12.9%	2.25 [1.32, 3.82]
Faraone et al, 2021	25	214	5	107	6.3%	2.50 [0.98, 6.35]
Martin et al, 2014B - AMPH	4	17	1	17	0.9%	4.00 [0.50, 32.20]
Nujiten et al, 2016	6	38	2	35	2.0%	2.76 [0.60, 12.80]
Spencer et al, 2008	27	137	2	135	1.9%	13.30 [3.23, 54.84]
Weisler et al, 2017	45	182	4	89	5.1%	5.50 [2.04, 14.82]
**Subtotal (95% CI)**		**650**		**448**	**29.0%**	**3.68 [2.50, 5.43]**
Total events	137		28			
Heterogeneity: $Chi^2 = 7.93$, df = 5 (P = 0.16); $I^2 = 37\%$						
Test for overall effect: Z = 6.60 (P < 0.00001)						
**Total (95% CI)**		**1386**		**1009**	**100.0%**	**3.02 [2.47, 3.69]**
Total events	396		103			
Heterogeneity: $Chi^2 = 33.13$, df = 14 (P = 0.003); $I^2 = 58\%$						
Test for overall effect: Z = 10.72 (P < 0.00001)						
Test for subgroup differences: $Chi^2 = 1.61$, df = 1 (P = 0.20), $I^2 = 38.0\%$						

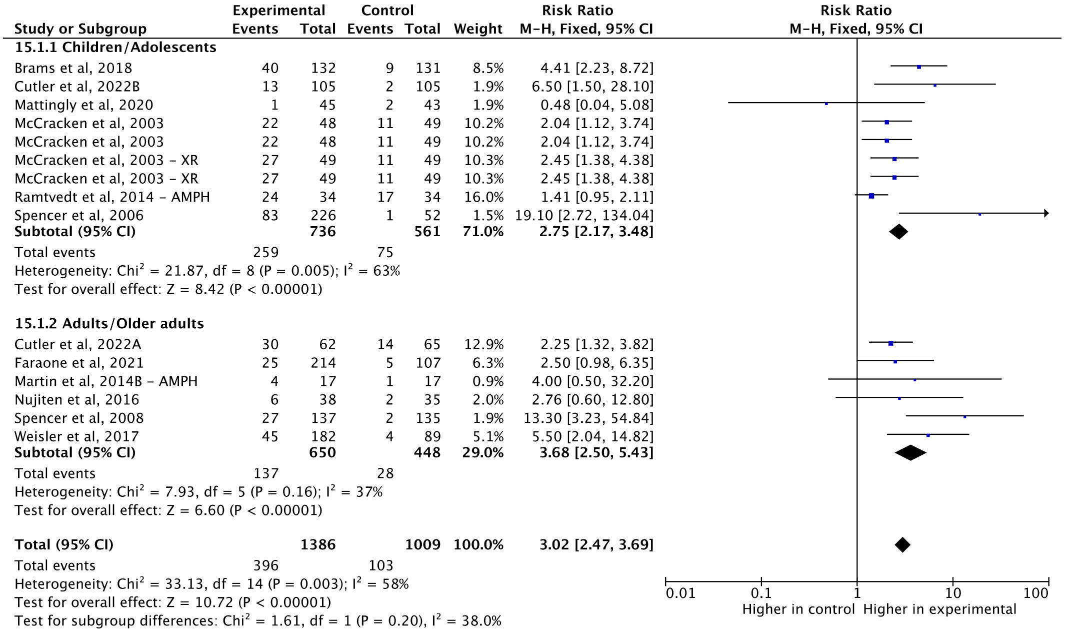

Figure legend: 95%CI, 95% confidence interval; AMPH, amphetamines; M-H, Mantel–Haenszel; XR, extended release.

eFigure 37. Forest plot showing the risk ratio of **dry mouth** between control and experimental groups using **amphetamines** subdivided by age.

Study or Subgroup	Experimental Events	Experimental Total	Control Events	Control Total	Weight	Risk Ratio M-H, Fixed, 95% CI
**15.5.1 Children/Adolescents**						
**Subtotal (95% CI)**		**0**		**0**		**Not estimable**
Total events	0		0			
Heterogeneity: Not applicable						
Test for overall effect: Not applicable						
**15.5.2 Adults/Older adults**						
Cutler et al, 2022A	12	62	1	65	4.8%	12.58 [1.69, 93.90]
Faraone et al, 2021	18	214	4	107	26.2%	2.25 [0.78, 6.48]
Martin et al, 2014B - AMPH	4	17	3	17	14.7%	1.33 [0.35, 5.08]
Spencer et al, 2008	31	137	7	135	34.6%	4.36 [1.99, 9.57]
Weisler et al, 2017	33	182	3	89	19.8%	5.38 [1.70, 17.06]
**Subtotal (95% CI)**		**612**		**413**	**100.0%**	**3.96 [2.43, 6.45]**
Total events	98		18			
Heterogeneity: $Chi^2 = 5.24$, df = 4 (P = 0.26); $I^2 = 24\%$						
Test for overall effect: Z = 5.53 (P < 0.00001)						
**Total (95% CI)**		**612**		**413**	**100.0%**	**3.96 [2.43, 6.45]**
Total events	98		18			
Heterogeneity: $Chi^2 = 5.24$, df = 4 (P = 0.26); $I^2 = 24\%$						
Test for overall effect: Z = 5.53 (P < 0.00001)						
Test for subgroup differences: Not applicable						

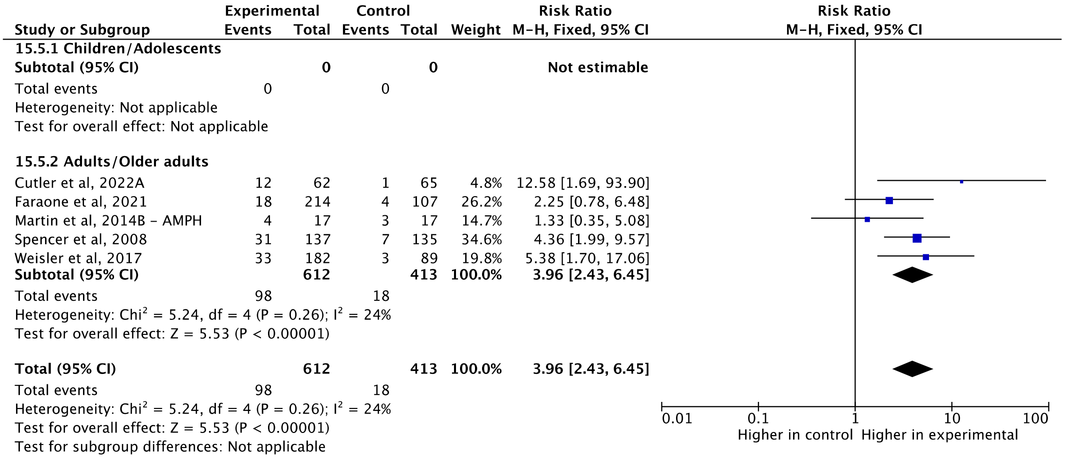

Figure legend: 95%CI, 95% confidence interval; AMPH, amphetamines; M-H, Mantel–Haenszel. No study encompassing children/adolescents was included in the meta-analysis.

eFigure 38. Forest plot showing the risk ratio of **headache** between control and experimental groups using **amphetamines** subdivided by age.

	Experimental		Control			Risk Ratio
**Study or Subgroup**	**Events**	**Total**	**Events**	**Total**	**Weight**	**M-H, Fixed, 95% CI**
**15.2.1 Children/Adolescents**						
Brams et al, 2018	16	132	14	131	9.8%	1.13 [0.58, 2.23]
Childress et al, 2017	1	51	1	48	0.7%	0.94 [0.06, 14.63]
Mattingly et al, 2020	2	45	3	43	2.1%	0.64 [0.11, 3.63]
McCracken et al, 2003	12	48	12	49	8.3%	1.02 [0.51, 2.04]
McCracken et al, 2003	12	48	12	49	8.3%	1.02 [0.51, 2.04]
McCracken et al, 2003 - XR	12	49	12	49	8.4%	1.00 [0.50, 2.00]
McCracken et al, 2003 - XR	12	49	12	49	8.4%	1.00 [0.50, 2.00]
Ramtvedt et al, 2014 - AMPH	8	34	8	34	5.6%	1.00 [0.42, 2.36]
Spencer et al, 2006	38	226	12	54	13.5%	0.76 [0.42, 1.35]
**Subtotal (95% CI)**		**682**		**506**	**65.2%**	**0.96 [0.75, 1.24]**
Total events	113		86			
Heterogeneity: Chi² = 1.20, df = 8 (P = 1.00); I² = 0%						
Test for overall effect: Z = 0.29 (P = 0.77)						
**15.2.2 Adults/Older adults**						
Cutler et al, 2022B	6	105	4	105	2.8%	1.50 [0.44, 5.16]
Faraone et al, 2021	26	214	13	107	12.1%	1.00 [0.54, 1.87]
Martin et al, 2014B - AMPH	1	17	3	17	2.1%	0.33 [0.04, 2.89]
Nujiten et al, 2016	1	38	1	35	0.7%	0.92 [0.06, 14.17]
Spencer et al, 2008	25	137	19	135	13.4%	1.30 [0.75, 2.24]
Weisler et al, 2017	17	182	4	89	3.8%	2.08 [0.72, 5.99]
**Subtotal (95% CI)**		**693**		**488**	**34.8%**	**1.23 [0.86, 1.75]**
Total events	76		44			
Heterogeneity: Chi² = 2.94, df = 5 (P = 0.71); I² = 0%						
Test for overall effect: Z = 1.14 (P = 0.26)						
**Total (95% CI)**		**1375**		**994**	**100.0%**	**1.06 [0.86, 1.30]**
Total events	189		130			
Heterogeneity: Chi² = 5.29, df = 14 (P = 0.98); I² = 0%						
Test for overall effect: Z = 0.51 (P = 0.61)						
Test for subgroup differences: Chi² = 1.20, df = 1 (P = 0.27), I² = 16.4%						

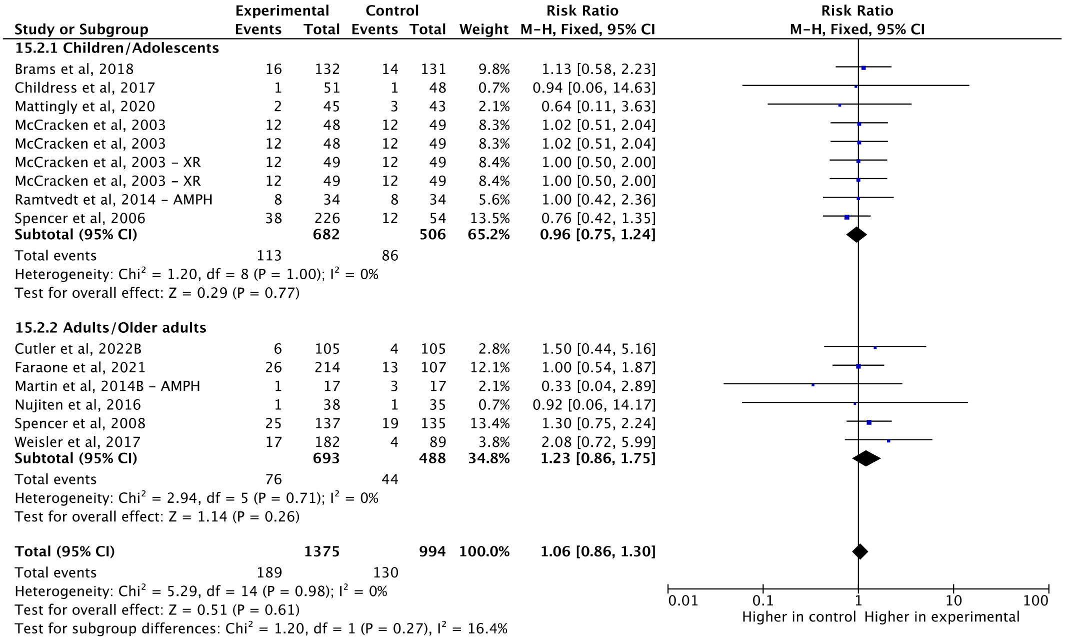

Figure legend: 95%CI, 95% confidence interval; AMPH, amphetamines; M-H, Mantel–Haenszel; XR, extended release.

eFigure 39. Forest plot showing the risk ratio of **insomnia** between control and experimental groups using **amphetamines** subdivided by age.

Study or Subgroup	Experimental Events	Experimental Total	Control Events	Control Total	Weight	Risk Ratio M-H, Fixed, 95% CI
**15.3.1 Children/Adolescents**						
Brams et al, 2018	22	132	3	131	4.0%	7.28 [2.23, 23.73]
Childress et al, 2017	1	51	1	48	1.4%	0.94 [0.06, 14.63]
McCracken et al, 2003	17	48	10	49	13.0%	1.74 [0.89, 3.40]
McCracken et al, 2003 - XR	14	49	10	49	13.2%	1.40 [0.69, 2.84]
Ramtvedt et al, 2014 - AMPH	30	34	14	34	18.4%	2.14 [1.41, 3.26]
Spencer et al, 2006	28	226	2	52	4.3%	3.22 [0.79, 13.10]
**Subtotal (95% CI)**		**540**		**363**	**54.3%**	**2.30 [1.67, 3.15]**
Total events	112		40			
Heterogeneity: $Chi^2 = 6.93$, df = 5 (P = 0.23); $I^2 = 28\%$						
Test for overall effect: Z = 5.16 (P < 0.00001)						
**15.3.2 Adults/Older adults**						
Cutler et al, 2022A	14	62	8	65	10.3%	1.83 [0.83, 4.07]
Cutler et al, 2022B	6	105	5	105	6.6%	1.20 [0.38, 3.81]
Faraone et al, 2021	19	214	4	107	7.0%	2.38 [0.83, 6.81]
Nujiten et al, 2016	13	38	3	35	4.1%	3.99 [1.24, 12.84]
Spencer et al, 2008	40	137	12	135	15.9%	3.28 [1.80, 5.98]
Weisler et al, 2017	22	182	1	89	1.8%	10.76 [1.47, 78.54]
**Subtotal (95% CI)**		**738**		**536**	**45.7%**	**2.87 [1.97, 4.18]**
Total events	114		33			
Heterogeneity: $Chi^2 = 5.72$, df = 5 (P = 0.33); $I^2 = 13\%$						
Test for overall effect: Z = 5.50 (P < 0.00001)						
**Total (95% CI)**		**1278**		**899**	**100.0%**	**2.56 [2.00, 3.26]**
Total events	226		73			
Heterogeneity: $Chi^2 = 13.94$, df = 11 (P = 0.24); $I^2 = 21\%$						
Test for overall effect: Z = 7.55 (P < 0.00001)						
Test for subgroup differences: $Chi^2 = 0.80$, df = 1 (P = 0.37), $I^2 = 0\%$						

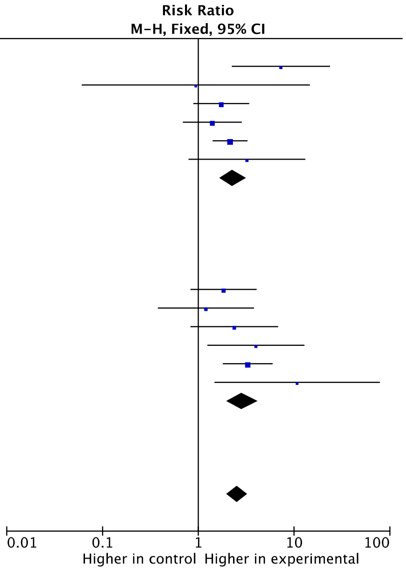

Figure legend: 95%CI, 95% confidence interval; AMPH, amphetamines; M-H, Mantel–Haenszel; XR, extended release.

eFigure 40. Forest plot showing the risk ratio of **irritability** between control and experimental groups using **amphetamines** subdivided by age.

Study or Subgroup	Experimental Events	Experimental Total	Control Events	Control Total	Weight	Risk Ratio M-H, Random, 95% CI
**15.4.1 Children/Adolescents**						
Brams et al, 2018	9	132	2	131	5.7%	4.47 [0.98, 20.28]
Cutler et al, 2022B	2	105	1	105	2.6%	2.00 [0.18, 21.72]
McCracken et al, 2003	22	48	29	49	21.4%	0.77 [0.53, 1.14]
McCracken et al, 2003 - XR	21	49	29	49	21.1%	0.72 [0.49, 1.08]
**Subtotal (95% CI)**		**334**		**334**	**50.8%**	**0.93 [0.56, 1.55]**
Total events	54		61			
Heterogeneity: $Tau^2 = 0.12$; $Chi^2 = 6.37$, df = 3 (P = 0.09); $I^2 = 53\%$						
Test for overall effect: Z = 0.29 (P = 0.77)						
**15.4.2 Adults/Older adults**						
Cutler et al, 2022A	11	62	5	65	10.2%	2.31 [0.85, 6.26]
Nujiten et al, 2016	6	38	2	35	5.5%	2.76 [0.60, 12.80]
Ramtvedt et al, 2014 - AMPH	23	34	27	34	23.4%	0.85 [0.64, 1.14]
Spencer et al, 2008	13	137	5	135	10.1%	2.56 [0.94, 6.99]
**Subtotal (95% CI)**		**271**		**269**	**49.2%**	**1.75 [0.70, 4.38]**
Total events	53		39			
Heterogeneity: $Tau^2 = 0.63$; $Chi^2 = 13.44$, df = 3 (P = 0.004); $I^2 = 78\%$						
Test for overall effect: Z = 1.20 (P = 0.23)						
**Total (95% CI)**		**605**		**603**	**100.0%**	**1.20 [0.80, 1.79]**
Total events	107		100			
Heterogeneity: $Tau^2 = 0.16$; $Chi^2 = 19.27$, df = 7 (P = 0.007); $I^2 = 64\%$						
Test for overall effect: Z = 0.86 (P = 0.39)						
Test for subgroup differences: $Chi^2 = 1.41$, df = 1 (P = 0.24), $I^2 = 29.0\%$						

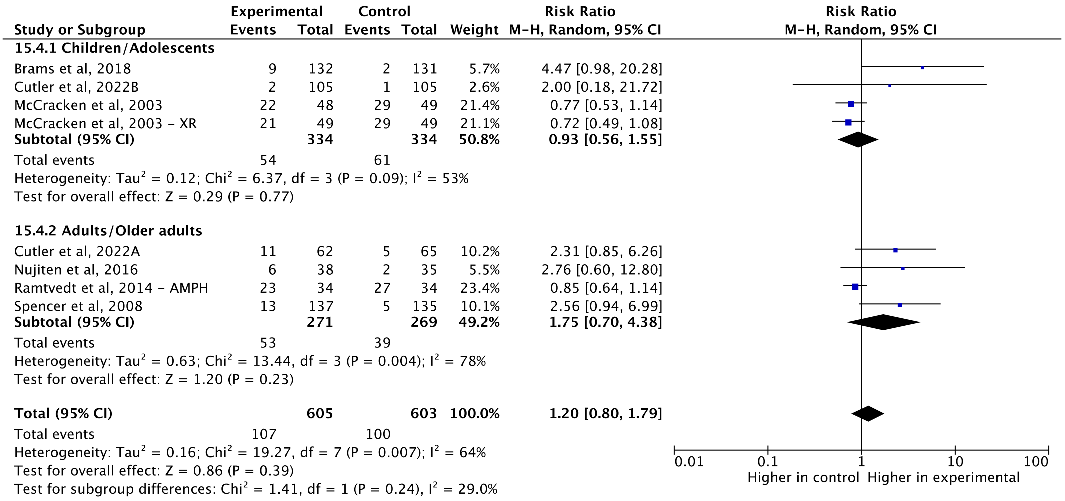

Figure legend: 95%CI, 95% confidence interval; AMPH, amphetamines; M-H, Mantel–Haenszel.

eFigure 41. Forest plot showing the risk ratio of **nausea** between control and experimental groups using **amphetamines** subdivided by age.

Study or Subgroup	Experimental Events	Experimental Total	Control Events	Control Total	Weight	Risk Ratio M-H, Fixed, 95% CI
**15.11.1 Children/Adolescents**						
Brams et al, 2018	9	132	4	131	23.5%	2.23 [0.71, 7.07]
Cutler et al, 2022B	3	105	1	105	5.9%	3.00 [0.32, 28.38]
Ramtvedt et al, 2014 - AMPH	10	34	6	34	35.2%	1.67 [0.68, 4.07]
**Subtotal (95% CI)**		**271**		**270**	**64.6%**	**1.99 [1.01, 3.92]**
Total events	22		11			
Heterogeneity: $Chi^2 = 0.32$, df = 2 (P = 0.85); $I^2 = 0\%$						
Test for overall effect: Z = 2.00 (P = 0.05)						
**15.11.2 Adults/Older adults**						
Spencer et al, 2008	10	137	6	135	35.4%	1.64 [0.61, 4.39]
**Subtotal (95% CI)**		**137**		**135**	**35.4%**	**1.64 [0.61, 4.39]**
Total events	10		6			
Heterogeneity: Not applicable						
Test for overall effect: Z = 0.99 (P = 0.32)						
**Total (95% CI)**		**408**		**405**	**100.0%**	**1.87 [1.07, 3.26]**
Total events	32		17			
Heterogeneity: $Chi^2 = 0.39$, df = 3 (P = 0.94); $I^2 = 0\%$						
Test for overall effect: Z = 2.20 (P = 0.03)						
Test for subgroup differences: $Chi^2 = 0.10$, df = 1 (P = 0.75), $I^2 = 0\%$						

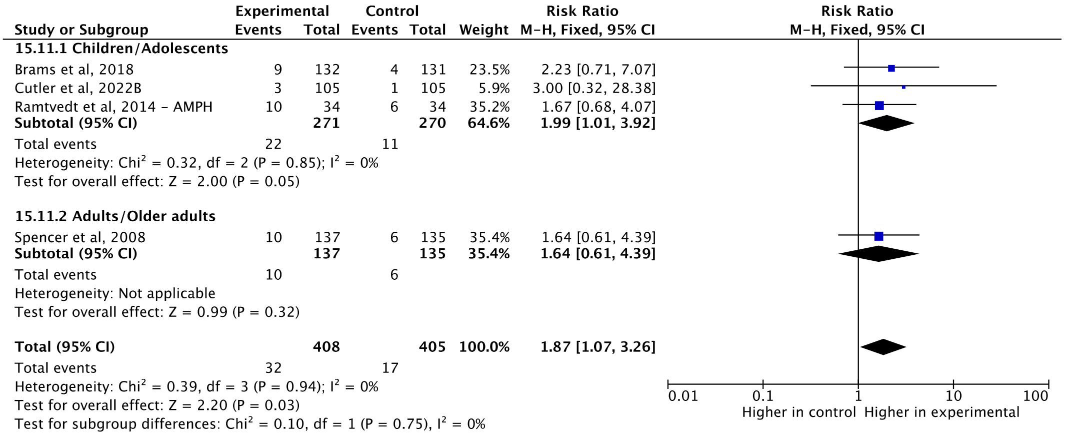

Figure legend: 95%CI, 95% confidence interval; AMPH, amphetamines; M-H, Mantel–Haenszel.

eFigure 42. Forest plot showing the mean differences in **heart rate** between control and experimental groups using **amphetamines** subdivided by age.

Study or Subgroup	Experimental Mean	Experimental SD	Experimental Total	Control Mean	Control SD	Control Total	Weight	Mean Difference IV, Fixed, 95% CI
**15.9.1 Children/Adolescents**								
Brams et al, 2008	5.7	11.78	132	0.7	10.79	131	22.1%	5.00 [2.27, 7.73]
Cutler et al, 2022B	13.4	13.7	105	12.8	12.9	105	12.7%	0.60 [−3.00, 4.20]
Mattingly et al, 2020	−0.5	9.87	45	−1.8	10.02	43	9.5%	1.30 [−2.86, 5.46]
**Subtotal (95% CI)**			**282**			**279**	**44.4%**	**2.94 [1.02, 4.87]**
Heterogeneity: $Chi^2 = 4.41$, df = 2 (P = 0.11); $I^2 = 55\%$								
Test for overall effect: Z = 2.99 (P = 0.003)								
**15.9.2 Adults/Older adults**								
Spencer et al, 2008	4.7	11.1	136	0.4	10.5	133	24.7%	4.30 [1.72, 6.88]
Weisler et al, 2017	3.3	10.52	182	0.1	8.35	89	30.9%	3.20 [0.89, 5.51]
**Subtotal (95% CI)**			**318**			**222**	**55.6%**	**3.69 [1.97, 5.41]**
Heterogeneity: $Chi^2 = 0.39$, df = 1 (P = 0.53); $I^2 = 0\%$								
Test for overall effect: Z = 4.20 (P < 0.0001)								
**Total (95% CI)**			**600**			**501**	**100.0%**	**3.36 [2.07, 4.64]**
Heterogeneity: $Chi^2 = 5.12$, df = 4 (P = 0.28); $I^2 = 22\%$								
Test for overall effect: Z = 5.13 (P < 0.00001)								
Test for subgroup differences: $Chi^2 = 0.32$, df = 1 (P = 0.57), $I^2 = 0\%$								

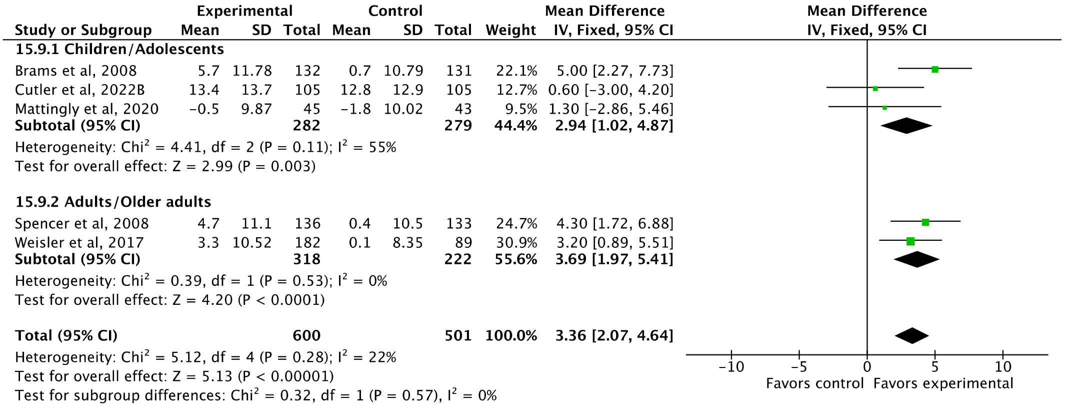

Figure legend: 95%CI, 95% confidence interval; IV, inverse variance; SD, standard deviation.

eFigure 43. Forest plot showing the mean differences in **diastolic blood pressure** between control and experimental groups using **amphetamines** subdivided by age.

	Experimental			Control				Mean Difference	Mean Difference
**Study or Subgroup**	**Mean**	**SD**	**Total**	**Mean**	**SD**	**Total**	**Weight**	**IV, Fixed, 95% CI**	**IV, Fixed, 95% CI**
**15.8.1 Children/Adolescents**									
Brams et al, 2008	4	8.23	132	0.5	7.45	131	22.9%	3.50 [1.60, 5.40]	
Cutler et al, 2022B	3.4	9.9	105	1.8	9.9	105	11.5%	1.60 [−1.08, 4.28]	
Mattingly et al, 2020	3.1	7.24	45	0.3	6.61	43	9.9%	2.80 [−0.09, 5.69]	
**Subtotal (95% CI)**			**282**			**279**	**44.3%**	**2.85 [1.49, 4.22]**	
Heterogeneity: Chi^2 = 1.29, df = 2 (P = 0.52); I^2 = 0%									
Test for overall effect: Z = 4.09 (P < 0.0001)									
**15.8.2 Adults/Older adults**									
Spencer et al, 2008	1.8	6.7	136	1.1	6.4	133	33.7%	0.70 [−0.87, 2.27]	
Weisler et al, 2017	1	7.46	182	0.1	7.72	89	22.0%	0.90 [−1.04, 2.84]	
**Subtotal (95% CI)**			**318**			**222**	**55.7%**	**0.78 [−0.44, 2.00]**	
Heterogeneity: Chi^2 = 0.02, df = 1 (P = 0.87); I^2 = 0%									
Test for overall effect: Z = 1.25 (P = 0.21)									
**Total (95% CI)**			**600**			**501**	**100.0%**	**1.70 [0.79, 2.61]**	
Heterogeneity: Chi^2 = 6.24, df = 4 (P = 0.18); I^2 = 36%									
Test for overall effect: Z = 3.66 (P = 0.0003)									
Test for subgroup differences: Chi^2 = 4.93, df = 1 (P = 0.03), I^2 = 79.7%									

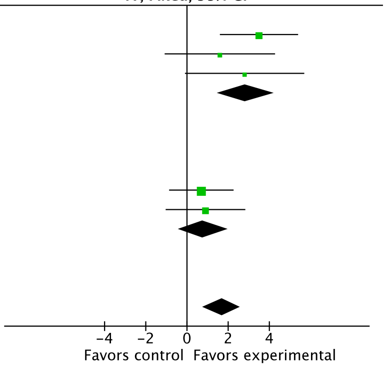

Figure legend: 95%CI, 95% confidence interval; IV, inverse variance; SD, standard deviation.

eFigure 44. Forest plot showing the mean differences in **systolic blood pressure** between control and experimental groups using **amphetamines** subdivided by age.

	Experimental			Control				Mean Difference
**Study or Subgroup**	**Mean**	**SD**	**Total**	**Mean**	**SD**	**Total**	**Weight**	**IV, Fixed, 95% CI**
**15.7.1 Children/Adolescents**								
Brams et al, 2008	3.8	9.15	132	2.1	8.72	131	25.1%	1.70 [−0.46, 3.86]
Cutler et al, 2022B	3.3	11	105	3.1	10.8	105	13.5%	0.20 [−2.75, 3.15]
Mattingly et al, 2020	1.8	6.52	45	−0.8	6.23	43	16.5%	2.60 [−0.06, 5.26]
**Subtotal (95% CI)**			**282**			**279**	**55.0%**	**1.60 [0.14, 3.06]**
Heterogeneity: $Chi^2 = 1.42$, df = 2 (P = 0.49); $I^2 = 0\%$								
Test for overall effect: Z = 2.15 (P = 0.03)								
**15.7.2 Adults/Older adults**								
Spencer et al, 2008	1.3	9.3	136	0.2	9.4	133	23.4%	1.10 [−1.13, 3.33]
Weisler et al, 2017	0.2	7.24	182	−0.8	9.99	89	21.6%	1.00 [−1.33, 3.33]
**Subtotal (95% CI)**			**318**			**222**	**45.0%**	**1.05 [−0.56, 2.66]**
Heterogeneity: $Chi^2 = 0.00$, df = 1 (P = 0.95); $I^2 = 0\%$								
Test for overall effect: Z = 1.28 (P = 0.20)								
**Total (95% CI)**			**600**			**501**	**100.0%**	**1.35 [0.27, 2.44]**
Heterogeneity: $Chi^2 = 1.67$, df = 4 (P = 0.80); $I^2 = 0\%$								
Test for overall effect: Z = 2.46 (P = 0.01)								
Test for subgroup differences: $Chi^2 = 0.25$, df = 1 (P = 0.62), $I^2 = 0\%$								

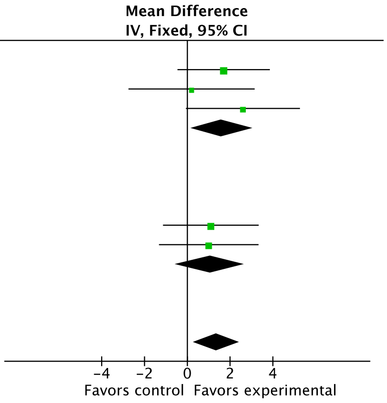

Figure legend: 95%CI, 95% confidence interval; IV, inverse variance; SD, standard deviation.

eFigures 45-99. Forest plots dividing groups according to participant age and stimulant dosage.

eFigure 45. Forest plot showing the risk ratio of **all adverse events** between control and experimental groups in **children** using **methylphenidate** subdivided by stimulant dosage.

Study or Subgroup	Experimental Events	Experimental Total	Control Events	Control Total	Weight	Risk Ratio M-H, Random, 95% CI
**16.10.1 Low**						
Schulz et al, 2010	44	146	38	146	5.9%	1.16 [0.80, 1.67]
**Subtotal (95% CI)**		**146**		**146**	**5.9%**	**1.16 [0.80, 1.67]**
Total events	44		38			
Heterogeneity: Not applicable						
Test for overall effect: Z = 0.78 (P = 0.44)						
**16.10.2 Medium**						
Brams et al, 2008	15	86	19	86	3.7%	0.79 [0.43, 1.45]
Childress et al, 2009	116	182	36	63	7.5%	1.12 [0.88, 1.42]
Childress et al, 2020C	10	39	6	50	2.0%	2.14 [0.85, 5.37]
Coghill et al, 2013 - MPH	72	111	63	110	7.8%	1.13 [0.92, 1.40]
Findling et al, 2008 - MPH	74	98	49	85	7.8%	1.31 [1.06, 1.62]
Huang et al, 2021	79	110	10	101	3.7%	7.25 [3.98, 13.21]
Muniz et al, 2008	40	332	0	83	0.3%	20.43 [1.27, 328.88]
Newcorn et al, 2008	146	219	40	74	7.6%	1.23 [0.98, 1.55]
Silva et al, 2005 - ER	5	53	2	53	0.8%	2.50 [0.51, 12.32]
Silva et al, 2005 - OROS	11	53	2	53	0.9%	5.50 [1.28, 23.63]
Wilens et al, 2006	15	87	14	90	3.3%	1.11 [0.57, 2.16]
Winhusen et al, 2011	111	151	98	152	8.4%	1.14 [0.98, 1.33]
Wolraich et al, 2001 - IR	40	95	31	90	5.9%	1.22 [0.84, 1.77]
Wolraich et al, 2001 - OROS	45	97	31	90	6.1%	1.35 [0.94, 1.92]
Zheng et al, 2024	74	110	55	112	7.6%	1.37 [1.09, 1.72]
**Subtotal (95% CI)**		**1823**		**1292**	**73.3%**	**1.39 [1.16, 1.68]**
Total events	853		456			
Heterogeneity: Tau² = 0.08; Chi² = 56.22, df = 14 (P < 0.00001); I² = 75%						
Test for overall effect: Z = 3.47 (P = 0.0005)						
**16.10.3 High**						
Childress et al, 2020A	24	65	22	54	5.0%	0.91 [0.58, 1.42]
Childress et al, 2020B	18	75	7	73	2.5%	2.50 [1.11, 5.63]
Pliszka et al, 2017	56	81	39	80	7.1%	1.42 [1.09, 1.85]
Weiss et al, 2021	154	293	24	74	6.2%	1.62 [1.15, 2.29]
**Subtotal (95% CI)**		**514**		**281**	**20.8%**	**1.41 [1.05, 1.90]**
Total events	252		92			
Heterogeneity: Tau² = 0.05; Chi² = 6.30, df = 3 (P = 0.10); I² = 52%						
Test for overall effect: Z = 2.26 (P = 0.02)						
**Total (95% CI)**		**2483**		**1719**	**100.0%**	**1.37 [1.18, 1.59]**
Total events	1149		586			
Heterogeneity: Tau² = 0.06; Chi² = 63.04, df = 19 (P < 0.00001); I² = 70%						
Test for overall effect: Z = 4.15 (P < 0.0001)						
Test for subgroup differences: Chi² = 0.86, df = 2 (P = 0.65), I² = 0%						

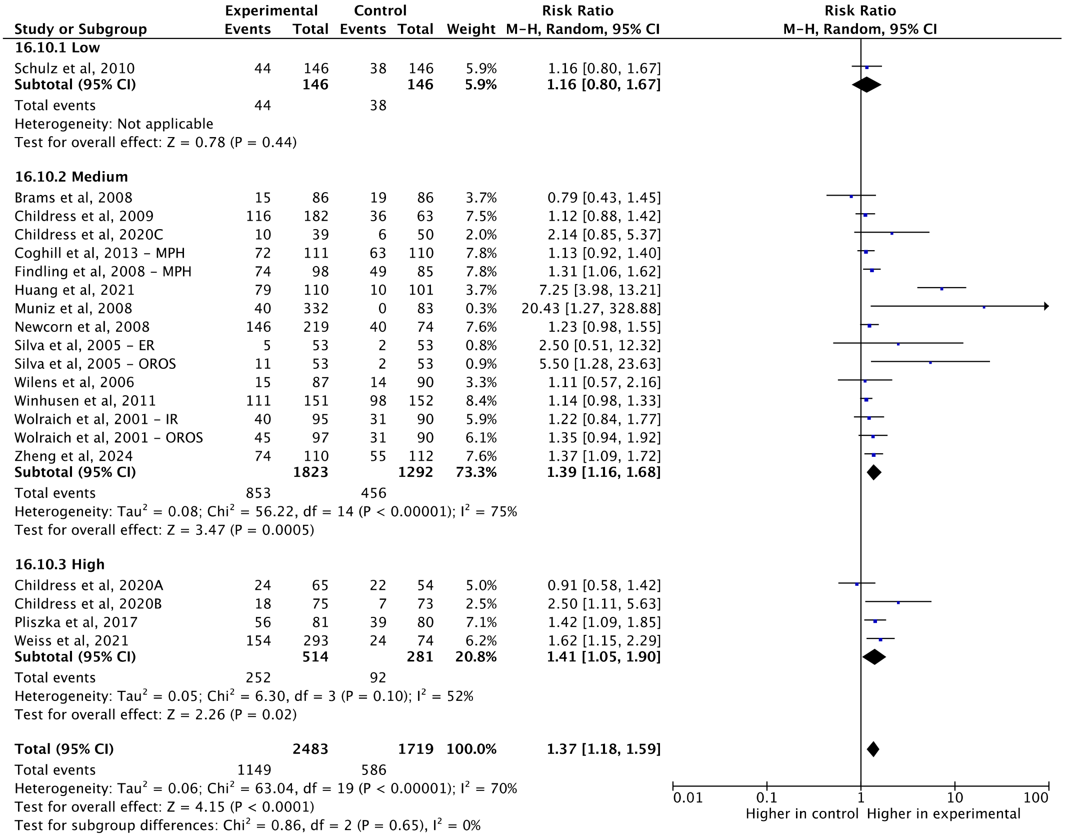

Figure legend: 95%CI, 95% confidence interval; ER, extended release; IR, immediate release; M-H, Mantel–Haenszel; MPH, methylphenidate; OROS, osmotic release oral system.

eFigure 46. Forest plot showing the risk ratio of **anxiety** between control and experimental groups in **children** using **methylphenidate** subdivided by stimulant dosage.

Study or Subgroup	Experimental Events	Experimental Total	Control Events	Control Total	Weight	Risk Ratio M-H, Fixed, 95% CI
**16.6.1 Low**						
**Subtotal (95% CI)**		**0**		**0**		**Not estimable**
Total events	0		0			
Heterogeneity: Not applicable						
Test for overall effect: Not applicable						
**16.6.2 Medium**						
Froehlich et al, 2020	32	171	26	171	44.1%	1.23 [0.77, 1.97]
Ramtvedt et al, 2014 - MPH	10	34	7	34	11.9%	1.43 [0.62, 3.31]
Stein et al, 2003	22	47	24	47	40.7%	0.92 [0.61, 1.39]
Zheng et al, 2024	3	110	2	112	3.4%	1.53 [0.26, 8.96]
**Subtotal (95% CI)**		**362**		**364**	**100.0%**	**1.14 [0.85, 1.53]**
Total events	67		59			
Heterogeneity: $Chi^2 = 1.54$, df = 3 (P = 0.67); $I^2 = 0\%$						
Test for overall effect: Z = 0.85 (P = 0.40)						
**16.6.3 High**						
**Subtotal (95% CI)**		**0**		**0**		**Not estimable**
Total events	0		0			
Heterogeneity: Not applicable						
Test for overall effect: Not applicable						
**Total (95% CI)**		**362**		**364**	**100.0%**	**1.14 [0.85, 1.53]**
Total events	67		59			
Heterogeneity: $Chi^2 = 1.54$, df = 3 (P = 0.67); $I^2 = 0\%$						
Test for overall effect: Z = 0.85 (P = 0.40)						
Test for subgroup differences: Not applicable						

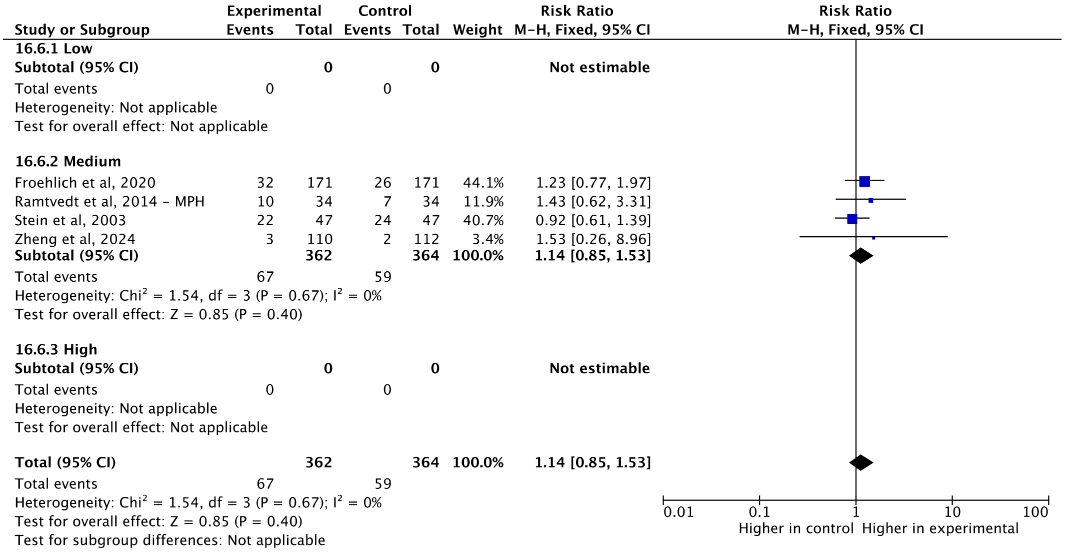

Figure legend: 95%CI, 95% confidence interval; M-H, Mantel–Haenszel; MPH, methylphenidate. No study encompassing low or high dosages was included in the meta-analysis.

eFigure 47. Forest plot showing the risk ratio of **decreased appetite** between control and experimental groups in **children** using **methylphenidate** subdivided by stimulant dosage.

Study or Subgroup	Experimental Events	Experimental Total	Control Events	Control Total	Weight	Risk Ratio M-H, Random, 95% CI
**16.1.1 Low**						
Schulz et al, 2010	3	146	2	146	4.7%	1.50 [0.25, 8.85]
**Subtotal (95% CI)**		**146**		**146**	**4.7%**	**1.50 [0.25, 8.85]**
Total events	3		2			
Heterogeneity: Not applicable						
Test for overall effect: Z = 0.45 (P = 0.65)						
**16.1.2 Medium**						
Childress et al, 2009	22	182	3	63	7.7%	2.54 [0.79, 8.19]
Coghill et al, 2013 - MPH	17	111	3	110	7.5%	5.62 [1.69, 18.62]
Findling et al, 2008 - MPH	25	98	4	85	8.8%	5.42 [1.97, 14.95]
Froehlich et al, 2020	106	171	45	171	14.4%	2.36 [1.79, 3.11]
Huang et al, 2021	53	110	1	101	4.1%	48.66 [6.86, 345.42]
Muniz et al, 2008	3	332	0	83	2.1%	1.77 [0.09, 33.86]
Newcorn et al, 2008	37	219	2	74	6.4%	6.25 [1.54, 25.31]
Ramtvedt et al, 2014 - MPH	25	34	17	34	13.7%	1.47 [0.99, 2.18]
Stein et al, 2003	35	47	16	47	13.4%	2.19 [1.42, 3.37]
Zheng et al, 2024	33	110	2	112	6.4%	16.80 [4.13, 68.32]
**Subtotal (95% CI)**		**1414**		**880**	**84.6%**	**3.74 [2.21, 6.34]**
Total events	356		93			
Heterogeneity: $Tau^2 = 0.42$; $Chi^2 = 41.44$, df = 9 (P < 0.00001); $I^2 = 78\%$						
Test for overall effect: Z = 4.91 (P < 0.00001)						
**16.1.3 High**						
Chronis-Tuscano et al, 2008	2	20	1	23	3.2%	2.30 [0.23, 23.51]
Pliszka et al, 2017	15	81	3	80	7.5%	4.94 [1.49, 16.40]
**Subtotal (95% CI)**		**101**		**103**	**10.7%**	**4.20 [1.45, 12.22]**
Total events	17		4			
Heterogeneity: $Tau^2 = 0.00$; $Chi^2 = 0.33$, df = 1 (P = 0.57); $I^2 = 0\%$						
Test for overall effect: Z = 2.64 (P = 0.008)						
**Total (95% CI)**		**1661**		**1129**	**100.0%**	**3.55 [2.23, 5.64]**
Total events	376		99			
Heterogeneity: $Tau^2 = 0.37$; $Chi^2 = 42.77$, df = 12 (P < 0.0001); $I^2 = 72\%$						
Test for overall effect: Z = 5.35 (P < 0.00001)						
Test for subgroup differences: $Chi^2 = 1.04$, df = 2 (P = 0.60), $I^2 = 0\%$						

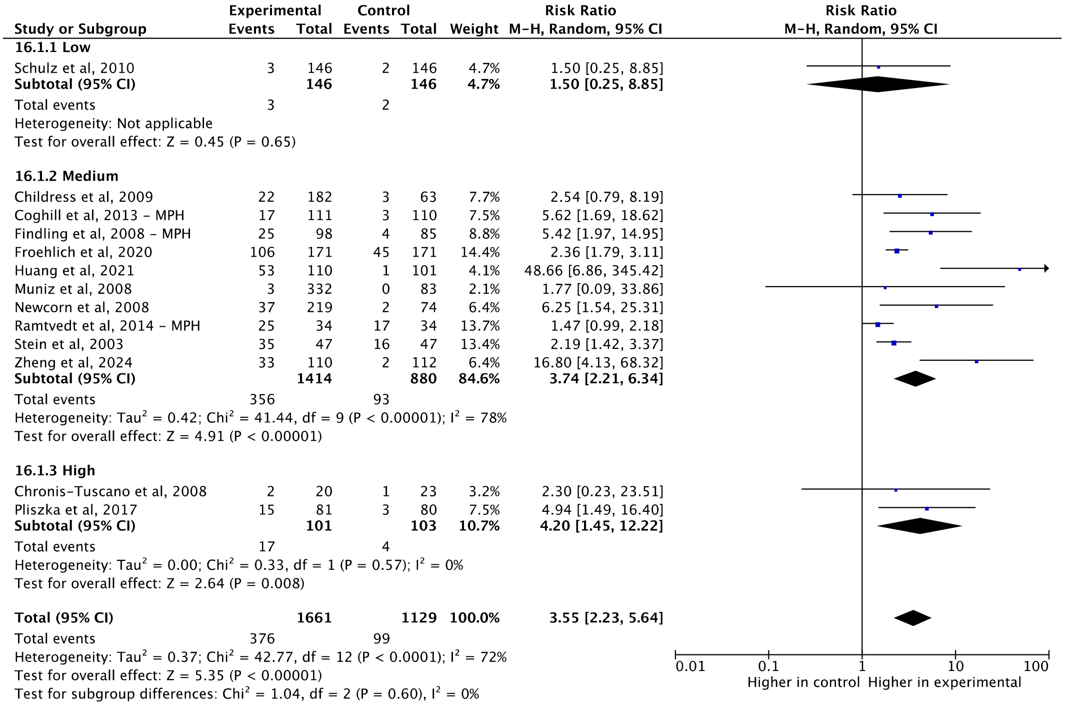

Figure legend: 95%CI, 95% confidence interval; M-H, Mantel–Haenszel; MPH, methylphenidate.

eFigure 48. Forest plot showing the risk ratio of **headache** between control and experimental groups in **children** using **methylphenidate** subdivided by stimulant dosage.

Study or Subgroup	Experimental Events	Experimental Total	Control Events	Control Total	Weight	Risk Ratio M-H, Fixed, 95% CI
**16.2.1 Low**						
Schulz et al, 2010	7	146	5	146	3.9%	1.40 [0.45, 4.31]
**Subtotal (95% CI)**		**146**		**146**	**3.9%**	**1.40 [0.45, 4.31]**
Total events	7		5			
Heterogeneity: Not applicable						
Test for overall effect: Z = 0.59 (P = 0.56)						
**16.2.2 Medium**						
Brams et al, 2018	3	86	2	86	1.6%	1.50 [0.26, 8.75]
Childress et al, 2009	22	182	6	63	7.0%	1.27 [0.54, 2.99]
Childress et al, 2020A	1	65	1	54	0.9%	0.83 [0.05, 12.97]
Coghill et al, 2013 - MPH	22	111	22	110	17.4%	0.99 [0.58, 1.68]
Huang et al, 2021	9	110	1	101	0.8%	8.26 [1.07, 64.08]
Newcorn et al, 2008	25	219	7	74	8.3%	1.21 [0.54, 2.67]
Pelham Jr et al, 2001 - IR	11	36	16	36	12.6%	0.69 [0.37, 1.27]
Pelham Jr et al, 2001 - LA	8	36	16	36	12.6%	0.50 [0.25, 1.02]
Ramtvedt et al, 2014 - MPH	9	34	8	34	6.3%	1.13 [0.49, 2.57]
Stein et al, 2003	17	47	16	47	12.6%	1.06 [0.61, 1.84]
Wilens et al, 2006	3	87	6	90	4.7%	0.52 [0.13, 2.00]
**Subtotal (95% CI)**		**1013**		**731**	**84.9%**	**0.99 [0.78, 1.26]**
Total events	130		101			
Heterogeneity: $Chi^2 = 10.85$, df = 10 (P = 0.37); $I^2 = 8\%$						
Test for overall effect: Z = 0.08 (P = 0.93)						
**16.2.3 High**						
Pliszka et al, 2017	6	81	3	80	2.4%	1.98 [0.51, 7.63]
Weiss et al, 2021	44	293	7	74	8.8%	1.59 [0.75, 3.38]
**Subtotal (95% CI)**		**374**		**154**	**11.2%**	**1.67 [0.86, 3.23]**
Total events	50		10			
Heterogeneity: $Chi^2 = 0.08$, df = 1 (P = 0.78); $I^2 = 0\%$						
Test for overall effect: Z = 1.53 (P = 0.13)						
**Total (95% CI)**		**1533**		**1031**	**100.0%**	**1.08 [0.87, 1.35]**
Total events	187		116			
Heterogeneity: $Chi^2 = 13.99$, df = 13 (P = 0.37); $I^2 = 7\%$						
Test for overall effect: Z = 0.70 (P = 0.48)						
Test for subgroup differences: $Chi^2 = 2.38$, df = 2 (P = 0.30), $I^2 = 15.9\%$						

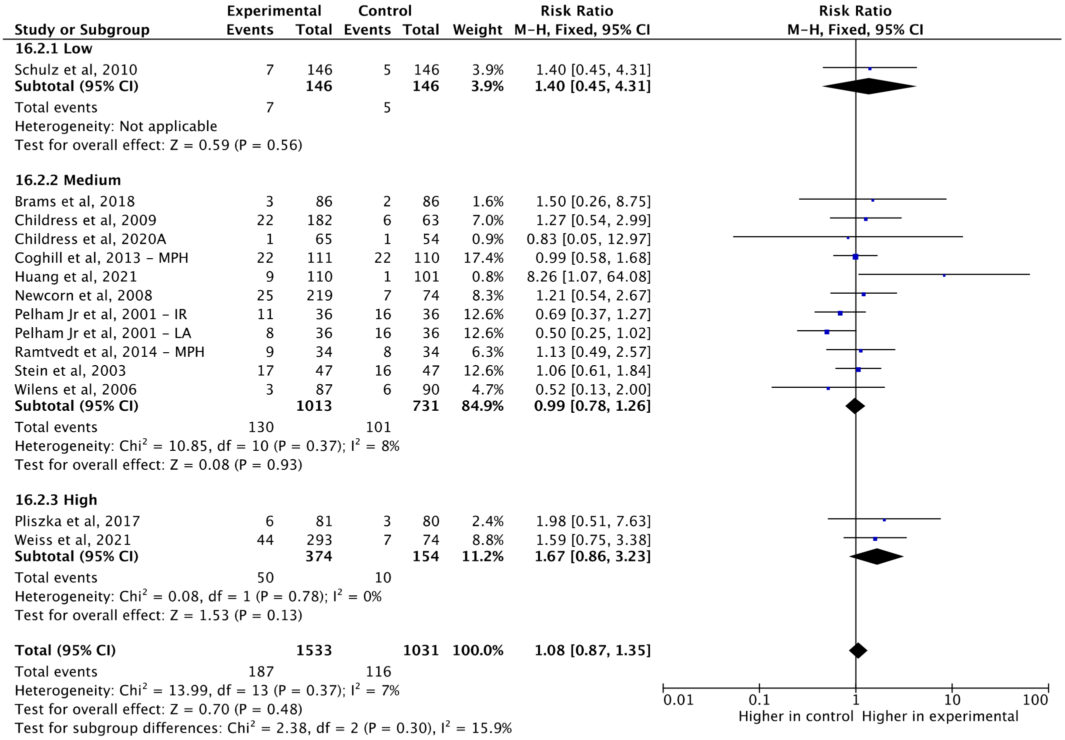

Figure legend: 95%CI, 95% confidence interval; IR, immediate release; LA, long-acting; M-H, Mantel–Haenszel; MPH, methylphenidate.

eFigure 49. Forest plot showing the risk ratio of **insomnia** between control and experimental groups in **children** using **methylphenidate** subdivided by stimulant dosage.

Study or Subgroup	Experimental Events	Experimental Total	Control Events	Control Total	Weight	Risk Ratio M-H, Fixed, 95% CI
**16.3.1 Low**						
**Subtotal (95% CI)**		**0**		**0**		**Not estimable**
Total events	0		0			
Heterogeneity: Not applicable						
Test for overall effect: Not applicable						
**16.3.2 Medium**						
Findling et al, 2008 - MPH	13	98	4	85	4.5%	2.82 [0.96, 8.32]
Froehlich et al, 2020	94	171	37	171	38.6%	2.54 [1.85, 3.48]
Newcorn et al, 2008	17	219	1	74	1.6%	5.74 [0.78, 42.42]
Ramtvedt et al, 2014 - MPH	18	34	14	34	14.6%	1.29 [0.77, 2.14]
Stein et al, 2003	30	47	21	47	21.9%	1.43 [0.97, 2.10]
Zheng et al, 2024	3	110	2	112	2.1%	1.53 [0.26, 8.96]
**Subtotal (95% CI)**		**679**		**523**	**83.2%**	**2.08 [1.67, 2.59]**
Total events	175		79			
Heterogeneity: $Chi^2 = 10.00$, df = 5 (P = 0.08); $I^2 = 50\%$						
Test for overall effect: Z = 6.53 (P < 0.00001)						
**16.3.3 High**						
Childress et al, 2020A	5	65	5	54	5.7%	0.83 [0.25, 2.72]
Pliszka et al, 2017	27	81	9	80	9.4%	2.96 [1.49, 5.90]
Weiss et al, 2021	19	293	1	74	1.7%	4.80 [0.65, 35.27]
**Subtotal (95% CI)**		**439**		**208**	**16.8%**	**2.42 [1.38, 4.25]**
Total events	51		15			
Heterogeneity: $Chi^2 = 3.91$, df = 2 (P = 0.14); $I^2 = 49\%$						
Test for overall effect: Z = 3.09 (P = 0.002)						
**Total (95% CI)**		**1118**		**731**	**100.0%**	**2.14 [1.74, 2.62]**
Total events	226		94			
Heterogeneity: $Chi^2 = 14.41$, df = 8 (P = 0.07); $I^2 = 45\%$						
Test for overall effect: Z = 7.21 (P < 0.00001)						
Test for subgroup differences: $Chi^2 = 0.25$, df = 1 (P = 0.62), $I^2 = 0\%$						

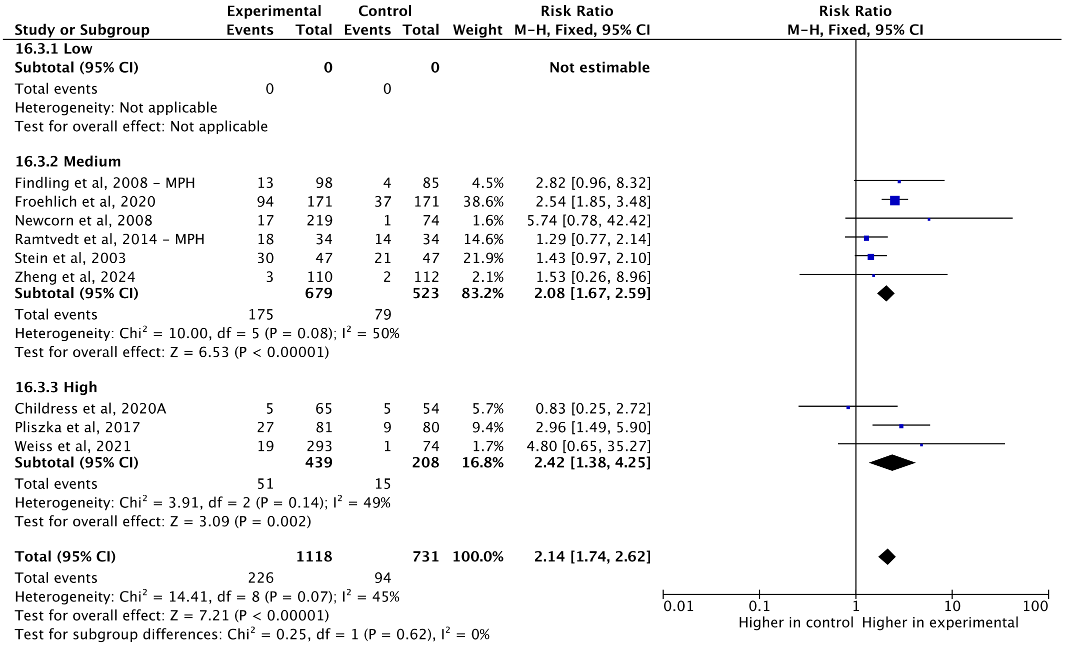

Figure legend: 95%CI, 95% confidence interval; M-H, Mantel–Haenszel; MPH, methylphenidate. No study encompassing low dosage was included in the meta-analysis.

eFigure 50. Forest plot showing the risk ratio of **irritability** between control and experimental groups in **children** using **methylphenidate** subdivided by stimulant dosage.

Study or Subgroup	Experimental Events	Experimental Total	Control Events	Control Total	Weight	Risk Ratio M-H, Fixed, 95% CI
**16.4.1 Low**						
**Subtotal (95% CI)**		**0**		**0**		**Not estimable**
Total events	0		0			
Heterogeneity: Not applicable						
Test for overall effect: Not applicable						
**16.4.2 Medium**						
Froehlich et al, 2020	77	171	67	171	50.4%	1.15 [0.90, 1.48]
Ramtvedt et al, 2014 - MPH	22	34	27	34	20.3%	0.81 [0.60, 1.10]
Stein et al, 2003	33	47	27	47	20.3%	1.22 [0.90, 1.66]
**Subtotal (95% CI)**		**252**		**252**	**91.0%**	**1.09 [0.92, 1.29]**
Total events	132		121			
Heterogeneity: $Chi^2 = 4.29$, df = 2 (P = 0.12); $I^2 = 53\%$						
Test for overall effect: Z = 1.00 (P = 0.32)						
**16.4.3 High**						
Chronis-Tuscano et al, 2008	3	15	1	23	0.6%	4.60 [0.53, 40.19]
Weiss et al, 2021	24	293	7	74	8.4%	0.87 [0.39, 1.93]
**Subtotal (95% CI)**		**308**		**97**	**9.0%**	**1.11 [0.54, 2.29]**
Total events	27		8			
Heterogeneity: $Chi^2 = 2.02$, df = 1 (P = 0.16); $I^2 = 51\%$						
Test for overall effect: Z = 0.29 (P = 0.77)						
**Total (95% CI)**		**560**		**349**	**100.0%**	**1.09 [0.92, 1.29]**
Total events	159		129			
Heterogeneity: $Chi^2 = 6.31$, df = 4 (P = 0.18); $I^2 = 37\%$						
Test for overall effect: Z = 1.04 (P = 0.30)						
Test for subgroup differences: $Chi^2 = 0.00$, df = 1 (P = 0.96), $I^2 = 0\%$						

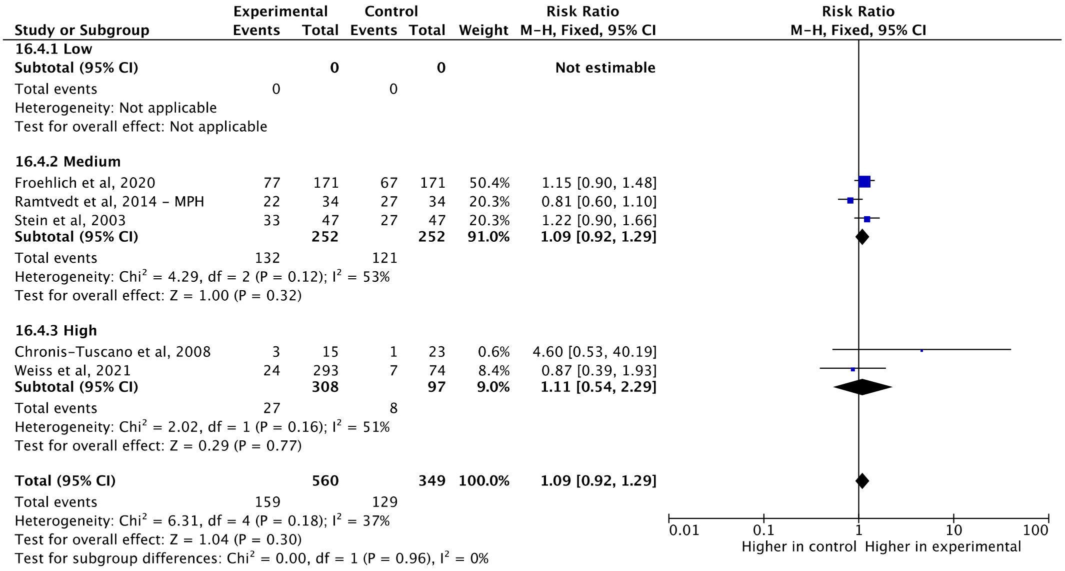

Figure legend: 95%CI, 95% confidence interval; M-H, Mantel–Haenszel; MPH, methylphenidate. No study encompassing low dosage was included in the meta-analysis.

eFigure 51. Forest plot showing the mean differences in **heart rate** between control and experimental groups using **methylphenidate** in **children** using **methylphenidate** subdivided by stimulant dosage.

Study or Subgroup	Experimental Mean	Experimental SD	Experimental Total	Control Mean	Control SD	Control Total	Weight	Mean Difference IV, Random, 95% CI
**16.9.1 Low**								
**Subtotal (95% CI)**			**0**			**0**		**Not estimable**
Heterogeneity: Not applicable								
Test for overall effect: Not applicable								
**16.9.2 Medium**								
Coghill et al, 2013 - MPH	5	12.8	222	−1.1	9.6	110	25.9%	6.10 [3.64, 8.56]
Newcorn et al, 2008	3	11.8	219	0.4	12	74	21.0%	2.60 [−0.55, 5.75]
Zheng et al, 2024	1.1	12.42	110	−1.9	10.59	112	21.7%	3.00 [−0.04, 6.04]
**Subtotal (95% CI)**			**551**			**296**	**68.6%**	**4.07 [1.77, 6.38]**
Heterogeneity: $Tau^2 = 2.01$; $Chi^2 = 3.88$, df = 2 (P = 0.14); $I^2 = 48\%$								
Test for overall effect: Z = 3.47 (P = 0.0005)								
**16.9.3 High**								
Childress et al, 2020A	1.7	11.12	65	−0.2	14.8	54	12.9%	1.90 [−2.88, 6.68]
Pliszka et al, 2017	3.5	11.49	81	3.1	11.58	80	18.5%	0.40 [−3.16, 3.96]
**Subtotal (95% CI)**			**146**			**134**	**31.4%**	**0.94 [−1.92, 3.79]**
Heterogeneity: $Tau^2 = 0.00$; $Chi^2 = 0.24$, df = 1 (P = 0.62); $I^2 = 0\%$								
Test for overall effect: Z = 0.64 (P = 0.52)								
**Total (95% CI)**			**697**			**430**	**100.0%**	**3.10 [1.02, 5.17]**
Heterogeneity: $Tau^2 = 2.76$; $Chi^2 = 8.04$, df = 4 (P = 0.09); $I^2 = 50\%$								
Test for overall effect: Z = 2.92 (P = 0.003)								
Test for subgroup differences: $Chi^2 = 2.81$, df = 1 (P = 0.09), $I^2 = 64.4\%$								

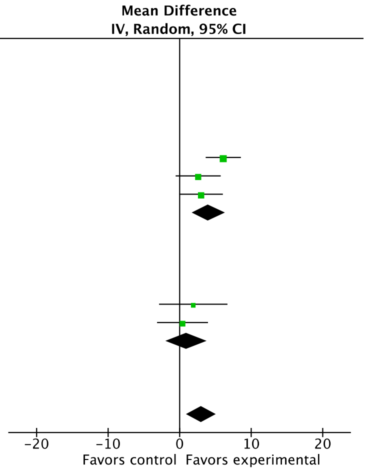

Figure legend: 95%CI, 95% confidence interval; IV, inverse variance; MPH, methylphenidate; SD, standard deviation. No study encompassing low dosage was included in the meta-analysis.

eFigure 52. Forest plot showing the mean differences in **diastolic blood pressure** between control and experimental groups using **methylphenidate** in **children** subdivided by stimulant dosage.

Study or Subgroup	Experimental Mean	Experimental SD	Experimental Total	Control Mean	Control SD	Control Total	Weight	Mean Difference IV, Fixed, 95% CI
**16.8.1 Low**								
**Subtotal (95% CI)**			**0**			**0**		**Not estimable**
Heterogeneity: Not applicable								
Test for overall effect: Not applicable								
**16.8.2 Medium**								
Coghill et al, 2013 - MPH	1.7	9.9	222	1.2	8.7	110	26.3%	0.50 [−1.58, 2.58]
Newcorn et al, 2008	3.1	8.04	219	0.4	7.8	74	26.6%	2.70 [0.63, 4.77]
Zheng et al, 2024	0.3	8.39	110	0.1	9.29	112	21.0%	0.20 [−2.13, 2.53]
**Subtotal (95% CI)**			**551**			**296**	**73.8%**	**1.21 [−0.04, 2.45]**
Heterogeneity: $Chi^2 = 3.16$, df = 2 (P = 0.21); $I^2 = 37\%$								
Test for overall effect: Z = 1.90 (P = 0.06)								
**16.8.3 High**								
Childress et al, 2020A	5.1	8.1	65	5.9	8.27	54	13.0%	−0.80 [−3.76, 2.16]
Pliszka et al, 2017	2.5	9.06	81	−0.4	9.99	80	13.1%	2.90 [−0.05, 5.85]
**Subtotal (95% CI)**			**146**			**134**	**26.2%**	**1.06 [−1.03, 3.14]**
Heterogeneity: $Chi^2 = 3.02$, df = 1 (P = 0.08); $I^2 = 67\%$								
Test for overall effect: Z = 0.99 (P = 0.32)								
**Total (95% CI)**			**697**			**430**	**100.0%**	**1.17 [0.10, 2.23]**
Heterogeneity: $Chi^2 = 6.19$, df = 4 (P = 0.19); $I^2 = 35\%$								
Test for overall effect: Z = 2.14 (P = 0.03)								
Test for subgroup differences: $Chi^2 = 0.01$, df = 1 (P = 0.90), $I^2 = 0\%$								

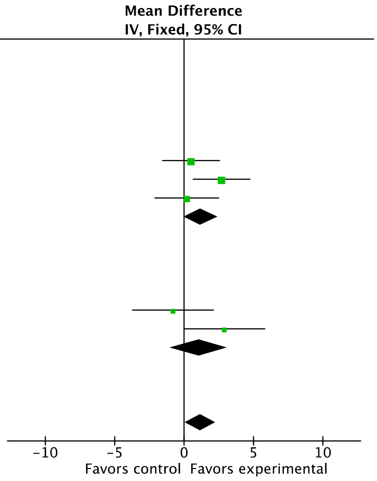

Figure legend: 95%CI, 95% confidence interval; IV, inverse variance; MPH, methylphenidate; SD, standard deviation. No study encompassing low dosage was included in the meta-analysis.

eFigure 53. Forest plot showing the mean differences in **systolic blood pressure** between control and experimental groups using **methylphenidate** in **children** subdivided by stimulant dosage.

	Experimental			Control				Mean Difference
**Study or Subgroup**	**Mean**	**SD**	**Total**	**Mean**	**SD**	**Total**	**Weight**	**IV, Fixed, 95% CI**
**16.7.1 Low**								
**Subtotal (95% CI)**			**0**			**0**		**Not estimable**
Heterogeneity: Not applicable								
Test for overall effect: Not applicable								
**16.7.2 Medium**								
Coghill et al, 2013 – MPH	0.3	11.1	222	1	9.6	110	27.3%	−0.70 [−3.01, 1.61]
Newcorn et al, 2008	2.4	9.7	219	1.3	8	74	29.3%	1.10 [−1.13, 3.33]
Zheng et al, 2024	1.7	9.29	110	1.7	10.83	112	20.7%	0.00 [−2.65, 2.65]
**Subtotal (95% CI)**			**551**			**296**	**77.3%**	**0.17 [−1.20, 1.54]**
Heterogeneity: $Chi^2 = 1.23$, df = 2 (P = 0.54); $I^2 = 0\%$								
Test for overall effect: Z = 0.24 (P = 0.81)								
**16.7.3 High**								
Childress et al, 2020A	6.3	9.32	65	3.6	10.64	54	11.1%	2.70 [−0.93, 6.33]
Pliszka et al, 2017	1.5	12.18	81	1.1	10.72	80	11.6%	0.40 [−3.14, 3.94]
**Subtotal (95% CI)**			**146**			**134**	**22.7%**	**1.52 [−1.01, 4.06]**
Heterogeneity: $Chi^2 = 0.79$, df = 1 (P = 0.37); $I^2 = 0\%$								
Test for overall effect: Z = 1.18 (P = 0.24)								
**Total (95% CI)**			**697**			**430**	**100.0%**	**0.48 [−0.73, 1.68]**
Heterogeneity: $Chi^2 = 2.86$, df = 4 (P = 0.58); $I^2 = 0\%$								
Test for overall effect: Z = 0.77 (P = 0.44)								
Test for subgroup differences: $Chi^2 = 0.84$, df = 1 (P = 0.36), $I^2 = 0\%$								

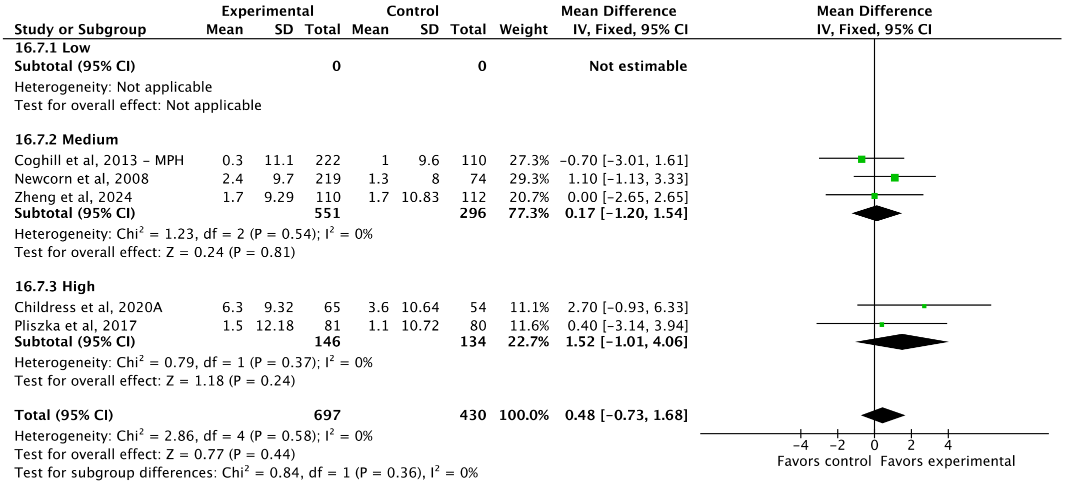

Figure legend: 95%CI, 95% confidence interval; IV, inverse variance; MPH, methylphenidate; SD, standard deviation. No study encompassing low dosage was included in the meta-analysis.

eFigure 54. Forest plot showing the risk ratio of **all adverse events** between control and experimental groups in **adults** using **methylphenidate** subdivided by stimulant dosage.

Study or Subgroup	Experimental Events	Experimental Total	Control Events	Control Total	Weight	Risk Ratio M-H, Random, 95% CI
**13.10.1 Low**						
**Subtotal (95% CI)**		**0**		**0**		**Not estimable**
Total events	0		0			
Heterogeneity: Not applicable						
Test for overall effect: Not applicable						
**13.10.2 Medium**						
Bron et al, 2014	17	22	10	22	2.3%	1.70 [1.02, 2.83]
Casas et al, 2013	161	181	76	97	11.3%	1.14 [1.01, 1.28]
Goodman et al, 2017	126	174	87	175	8.9%	1.46 [1.22, 1.74]
Medori et al, 2008	182	305	41	96	6.4%	1.40 [1.09, 1.79]
Patkar et al, 2006	19	30	17	30	3.2%	1.12 [0.74, 1.69]
Rösler et al, 2009	135	183	37	66	7.0%	1.32 [1.05, 1.66]
Spencer et al, 2007	145	165	36	53	8.2%	1.29 [1.07, 1.57]
Takahashi et al, 2014	117	143	76	141	9.1%	1.52 [1.28, 1.80]
**Subtotal (95% CI)**		**1203**		**680**	**56.5%**	**1.33 [1.20, 1.48]**
Total events	902		380			
Heterogeneity: $Tau^2 = 0.01$; $Chi^2 = 12.76$, df = 7 (P = 0.08); $I^2 = 45\%$						
Test for overall effect: Z = 5.52 (P < 0.00001)						
**13.10.3 High**						
Adler et al, 2009	93	110	74	116	9.6%	1.33 [1.13, 1.55]
Buitelaar et al, 2012	7	23	8	22	1.0%	0.84 [0.37, 1.92]
Childress et al, 2022	25	121	18	118	2.0%	1.35 [0.78, 2.35]
Ginsberg et al, 2014	175	216	65	82	10.8%	1.02 [0.90, 1.16]
Huss et al, 2014	401	542	108	180	10.8%	1.23 [1.08, 1.40]
Retz et al, 2012	55	84	32	78	5.0%	1.60 [1.17, 2.17]
Weiss et al, 2020	158	297	25	78	4.3%	1.66 [1.18, 2.33]
**Subtotal (95% CI)**		**1393**		**674**	**43.5%**	**1.27 [1.10, 1.48]**
Total events	914		330			
Heterogeneity: $Tau^2 = 0.02$; $Chi^2 = 16.89$, df = 6 (P = 0.010); $I^2 = 64\%$						
Test for overall effect: Z = 3.18 (P = 0.001)						
**Total (95% CI)**		**2596**		**1354**	**100.0%**	**1.30 [1.20, 1.42]**
Total events	1816		710			
Heterogeneity: $Tau^2 = 0.01$; $Chi^2 = 31.60$, df = 14 (P = 0.005); $I^2 = 56\%$						
Test for overall effect: Z = 6.12 (P < 0.00001)						
Test for subgroup differences: $Chi^2 = 0.23$, df = 1 (P = 0.63), $I^2 = 0\%$						

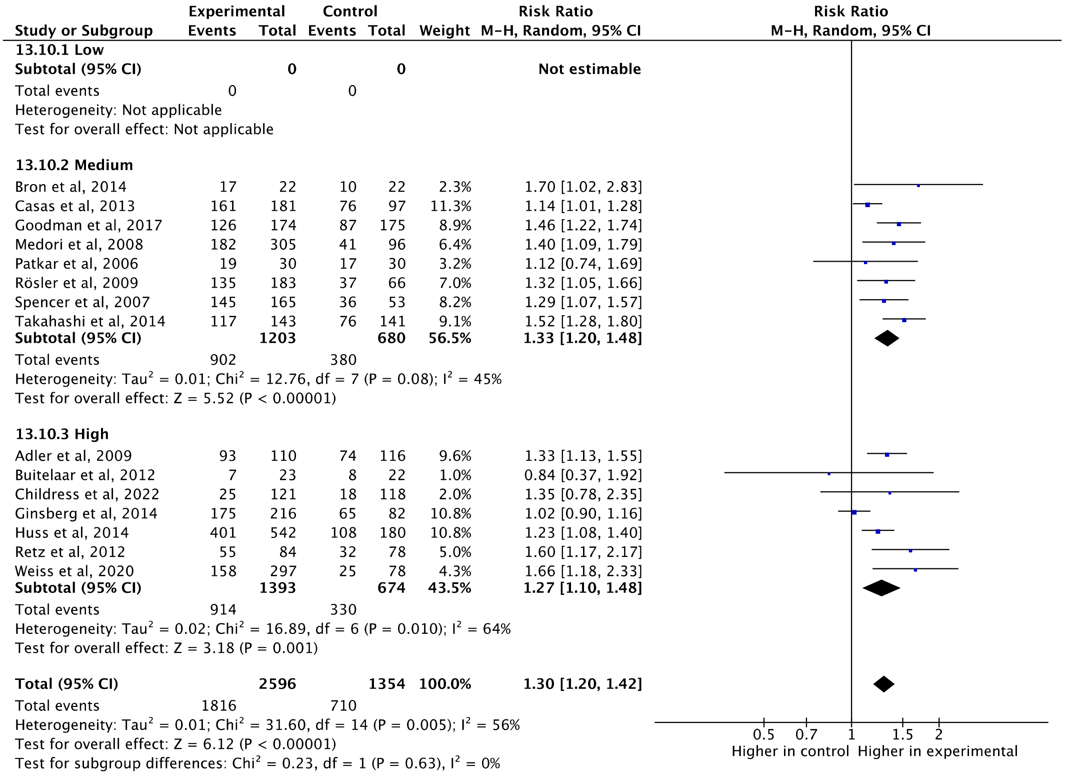

Figure legend: 95%CI, 95% confidence interval; M-H, Mantel–Haenszel. No study encompassing low dosage was included in the meta-analysis.

eFigure 55. Forest plot showing the risk ratio of **anxiety** between control and experimental groups in **adults** using **methylphenidate** subdivided by stimulant dosage.

Study or Subgroup	Experimental Events	Experimental Total	Control Events	Control Total	Weight	Risk Ratio M-H, Random, 95% CI
**13.6.1 Low**						
**Subtotal (95% CI)**		**0**		**0**		**Not estimable**
Total events	0		0			
Heterogeneity: Not applicable						
Test for overall effect: Not applicable						
**13.6.2 Medium**						
Medori et al, 2008	16	305	1	96	22.7%	5.04 [0.68, 37.48]
Patkar et al, 2006	2	30	1	30	19.0%	2.00 [0.19, 20.90]
**Subtotal (95% CI)**		**335**		**126**	**41.7%**	**3.41 [0.74, 15.67]**
Total events	18		2			
Heterogeneity: $Tau^2 = 0.00$; $Chi^2 = 0.37$, df = 1 (P = 0.55); $I^2 = 0\%$						
Test for overall effect: Z = 1.58 (P = 0.12)						
**13.6.3 High**						
Adler et al, 2009	18	110	4	116	36.9%	4.75 [1.66, 13.58]
Konstenius et al, 2014	1	27	4	27	21.3%	0.25 [0.03, 2.09]
**Subtotal (95% CI)**		**137**		**143**	**58.3%**	**1.27 [0.07, 22.45]**
Total events	19		8			
Heterogeneity: $Tau^2 = 3.62$; $Chi^2 = 5.94$, df = 1 (P = 0.01); $I^2 = 83\%$						
Test for overall effect: Z = 0.16 (P = 0.87)						
**Total (95% CI)**		**472**		**269**	**100.0%**	**2.18 [0.59, 8.09]**
Total events	37		10			
Heterogeneity: $Tau^2 = 0.93$; $Chi^2 = 6.34$, df = 3 (P = 0.10); $I^2 = 53\%$						
Test for overall effect: Z = 1.16 (P = 0.25)						
Test for subgroup differences: $Chi^2 = 0.36$, df = 1 (P = 0.55), $I^2 = 0\%$						

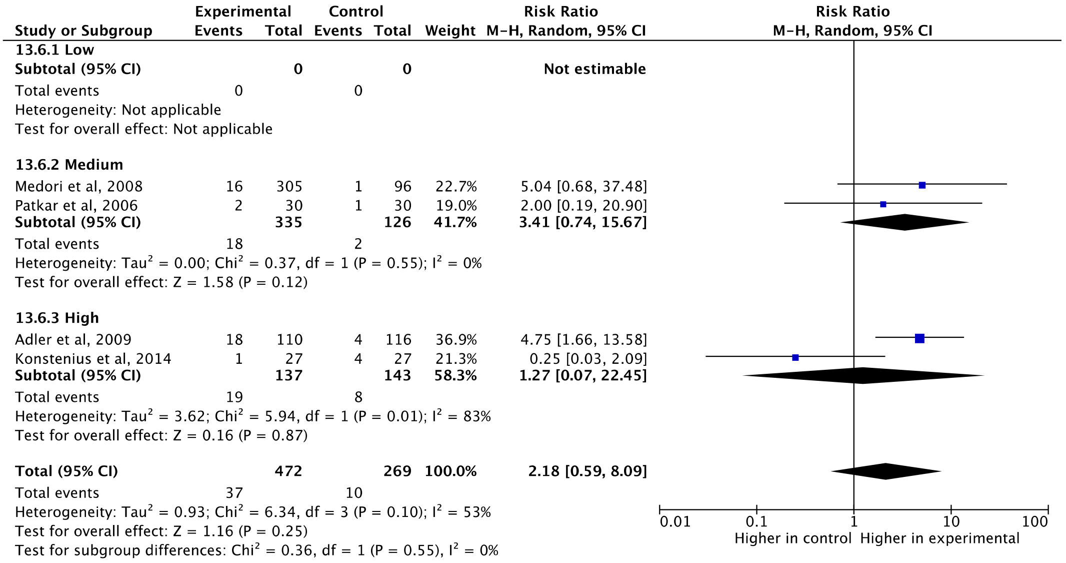

Figure legend: 95%CI, 95% confidence interval; M-H, Mantel–Haenszel. No study encompassing low dosage was included in the meta-analysis.

eFigure 56. Forest plot showing the risk ratio of **decreased appetite** between control and experimental groups in **adults** using **methylphenidate** subdivided by stimulant dosage.

Study or Subgroup	Experimental Events	Experimental Total	Control Events	Control Total	Weight	Risk Ratio M-H, Random, 95% CI
**13.1.1 Low**						
**Subtotal (95% CI)**		**0**		**0**		**Not estimable**
Total events	0		0			
Heterogeneity: Not applicable						
Test for overall effect: Not applicable						
**13.1.2 Medium**						
Biederman et al, 2006	23	67	2	74	5.7%	12.70 [3.11, 51.85]
Casas et al, 2013	43	181	5	97	8.6%	4.61 [1.89, 11.25]
Goodman et al, 2017	25	174	7	175	9.1%	3.59 [1.60, 8.09]
Medori et al, 2008	77	305	7	96	9.6%	3.46 [1.65, 7.25]
Patkar et al, 2006	3	30	1	30	3.2%	3.00 [0.33, 27.23]
Rösler et al, 2009	70	183	9	66	10.3%	2.81 [1.49, 5.29]
Spencer et al, 2007	30	165	6	53	9.1%	1.61 [0.71, 3.65]
**Subtotal (95% CI)**		**1105**		**591**	**55.6%**	**3.30 [2.26, 4.82]**
Total events	271		37			
Heterogeneity: $Tau^2 = 0.05$; $Chi^2 = 7.49$, df = 6 (P = 0.28); $I^2 = 20\%$						
Test for overall effect: Z = 6.19 (P < 0.00001)						
**13.1.3 High**						
Adler et al, 2009	28	110	7	116	9.3%	4.22 [1.92, 9.26]
Ginsberg et al, 2014	15	216	11	82	9.6%	0.52 [0.25, 1.08]
Huss et al, 2014	136	542	8	180	9.9%	5.65 [2.82, 11.29]
Retz et al, 2012	40	84	8	78	9.9%	4.64 [2.32, 9.29]
Weiss et al, 2020	33	297	2	78	5.8%	4.33 [1.06, 17.67]
**Subtotal (95% CI)**		**1249**		**534**	**44.4%**	**2.96 [1.13, 7.76]**
Total events	252		36			
Heterogeneity: $Tau^2 = 1.01$; $Chi^2 = 28.40$, df = 4 (P < 0.0001); $I^2 = 86\%$						
Test for overall effect: Z = 2.21 (P = 0.03)						
**Total (95% CI)**		**2354**		**1125**	**100.0%**	**3.24 [2.06, 5.09]**
Total events	523		73			
Heterogeneity: $Tau^2 = 0.41$; $Chi^2 = 35.95$, df = 11 (P = 0.0002); $I^2 = 69\%$						
Test for overall effect: Z = 5.08 (P < 0.00001)						
Test for subgroup differences: $Chi^2 = 0.04$, df = 1 (P = 0.84), $I^2 = 0\%$						

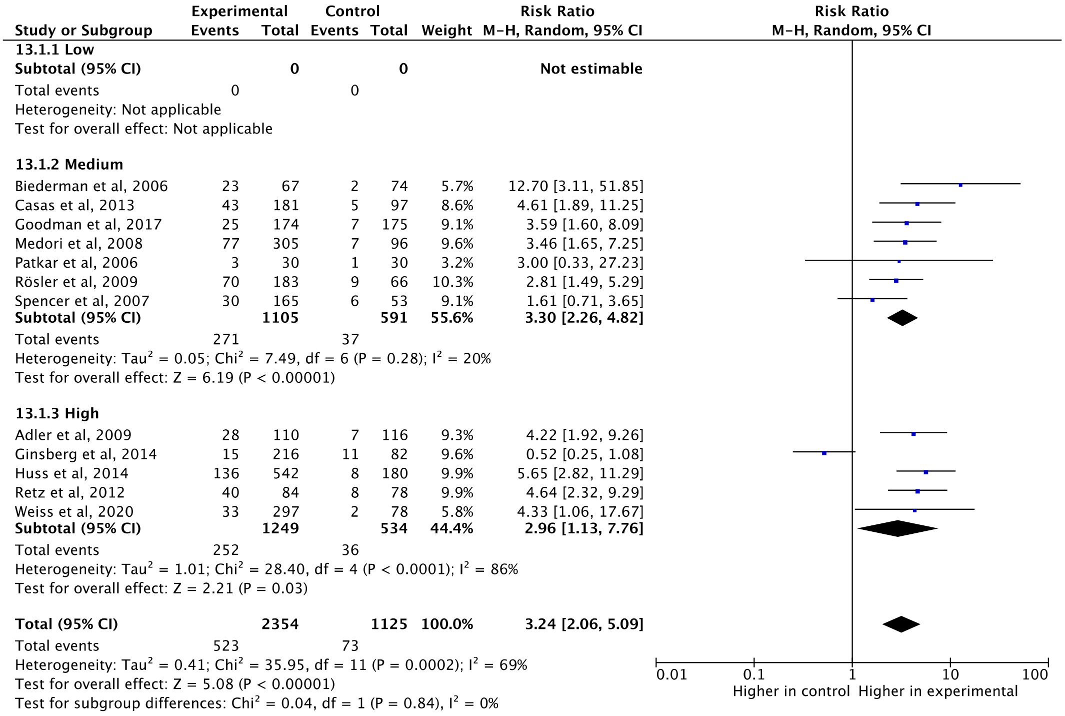

Figure legend: 95%CI, 95% confidence interval; M-H, Mantel–Haenszel. No study encompassing low dosage was included in the meta-analysis.

eFigure 57. Forest plot showing the risk ratio of **dry mouth** between control and experimental groups in **adults** using **methylphenidate** subdivided by stimulant dosage.

Study or Subgroup	Experimental Events	Experimental Total	Control Events	Control Total	Weight	Risk Ratio M-H, Random, 95% CI
**13.5.1 Low**						
**Subtotal (95% CI)**		**0**		**0**		**Not estimable**
Total events	0		0			
Heterogeneity: Not applicable						
Test for overall effect: Not applicable						
**13.5.2 Medium**						
Biederman et al, 2006	23	67	5	74	9.3%	5.08 [2.05, 12.61]
Casas et al, 2013	32	181	3	97	7.7%	5.72 [1.80, 18.19]
Goodman et al, 2017	27	174	3	175	7.6%	9.05 [2.80, 29.29]
Medori et al, 2008	36	305	2	96	6.4%	5.67 [1.39, 23.10]
Rösler et al, 2009	55	183	10	66	11.5%	1.98 [1.08, 3.66]
Spencer et al, 2007	26	165	2	53	6.4%	4.18 [1.03, 17.01]
**Subtotal (95% CI)**		**1075**		**561**	**48.9%**	**4.23 [2.48, 7.21]**
Total events	199		25			
Heterogeneity: Tau² = 0.16; Chi² = 7.87, df = 5 (P = 0.16); I² = 36%						
Test for overall effect: Z = 5.30 (P < 0.00001)						
**13.5.3 High**						
Adler et al, 2009	22	110	6	116	9.7%	3.87 [1.63, 9.18]
Ginsberg et al, 2014	15	216	9	82	10.2%	0.63 [0.29, 1.39]
Huss et al, 2014	110	542	4	180	8.8%	9.13 [3.42, 24.41]
Konstenius et al, 2014	2	27	1	27	3.2%	2.00 [0.19, 20.77]
Retz et al, 2012	32	84	11	78	11.5%	2.70 [1.47, 4.98]
Weiss et al, 2020	27	297	3	78	7.7%	2.36 [0.74, 7.59]
**Subtotal (95% CI)**		**1276**		**561**	**51.1%**	**2.58 [1.15, 5.78]**
Total events	208		34			
Heterogeneity: Tau² = 0.72; Chi² = 21.05, df = 5 (P = 0.0008); I² = 76%						
Test for overall effect: Z = 2.30 (P = 0.02)						
**Total (95% CI)**		**2351**		**1122**	**100.0%**	**3.38 [2.09, 5.45]**
Total events	407		59			
Heterogeneity: Tau² = 0.42; Chi² = 31.02, df = 11 (P = 0.001); I² = 65%						
Test for overall effect: Z = 4.98 (P < 0.00001)						
Test for subgroup differences: Chi² = 1.00, df = 1 (P = 0.32), I² = 0.3%						

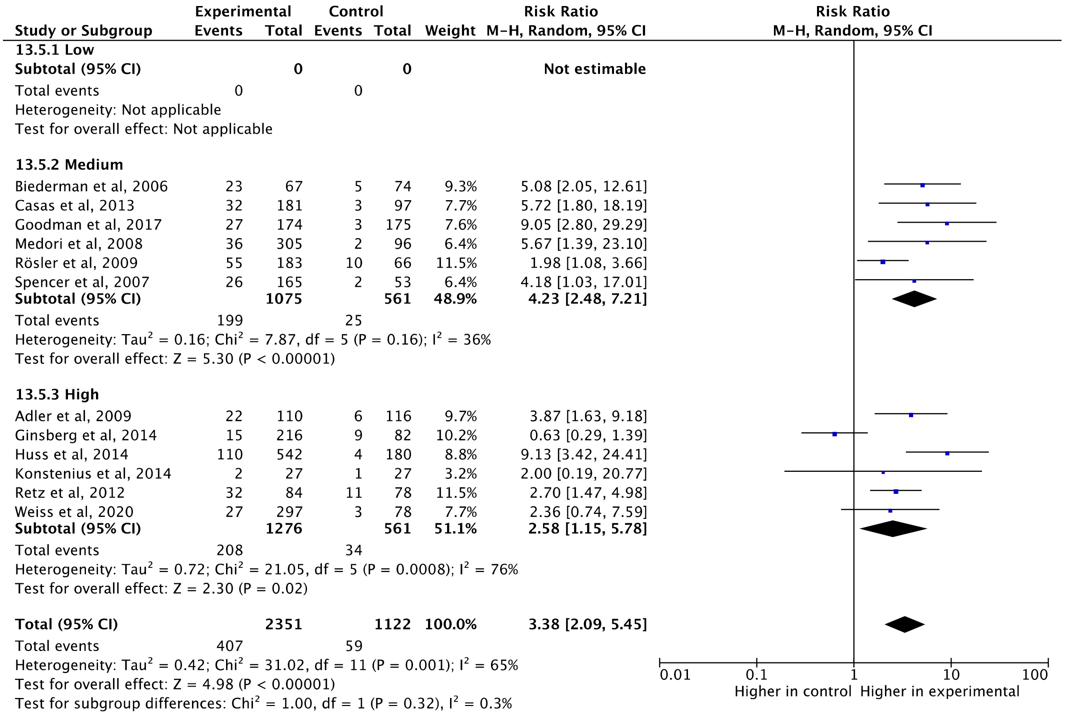

Figure legend: 95%CI, 95% confidence interval; M-H, Mantel–Haenszel. No study encompassing low dosage was included in the meta-analysis.

eFigure 58. Forest plot showing the risk ratio of **headache** between control and experimental groups in **adults** using **methylphenidate** subdivided by stimulant dosage.

Study or Subgroup	Experimental Events	Experimental Total	Control Events	Control Total	Weight	Risk Ratio M-H, Fixed, 95% CI
**13.2.1 Low**						
**Subtotal (95% CI)**		**0**		**0**		**Not estimable**
Total events	0		0			
Heterogeneity: Not applicable						
Test for overall effect: Not applicable						
**13.2.2 Medium**						
Biederman et al, 2006	21	67	22	74	8.6%	1.05 [0.64, 1.74]
Casas et al, 2013	52	181	27	97	14.5%	1.03 [0.70, 1.53]
Goodman et al, 2017	33	174	20	175	8.2%	1.66 [0.99, 2.78]
Medori et al, 2008	64	305	17	96	10.7%	1.18 [0.73, 1.92]
Patkar et al, 2006	3	30	2	30	0.8%	1.50 [0.27, 8.34]
Spencer et al, 2007	52	165	10	53	6.3%	1.67 [0.91, 3.05]
**Subtotal (95% CI)**		**922**		**525**	**49.2%**	**1.26 [1.02, 1.57]**
Total events	225		98			
Heterogeneity: Chi² = 3.53, df = 5 (P = 0.62); I² = 0%						
Test for overall effect: Z = 2.13 (P = 0.03)						
**13.2.3 High**						
Adler et al, 2009	28	110	16	116	6.4%	1.85 [1.06, 3.22]
Ginsberg et al, 2014	41	216	21	82	12.6%	0.74 [0.47, 1.17]
Huss et al, 2014	111	542	30	180	18.6%	1.23 [0.85, 1.77]
Konstenius et al, 2014	6	27	2	27	0.8%	3.00 [0.66, 13.56]
Retz et al, 2012	25	84	13	78	5.6%	1.79 [0.98, 3.24]
Shram et al, 2022	5	48	2	46	0.8%	2.40 [0.49, 11.74]
Weiss et al, 2020	52	297	9	78	5.9%	1.52 [0.78, 2.94]
**Subtotal (95% CI)**		**1324**		**607**	**50.8%**	**1.33 [1.07, 1.65]**
Total events	268		93			
Heterogeneity: Chi² = 10.44, df = 6 (P = 0.11); I² = 43%						
Test for overall effect: Z = 2.60 (P = 0.009)						
**Total (95% CI)**		**2246**		**1132**	**100.0%**	**1.30 [1.11, 1.51]**
Total events	493		191			
Heterogeneity: Chi² = 14.06, df = 12 (P = 0.30); I² = 15%						
Test for overall effect: Z = 3.35 (P = 0.0008)						
Test for subgroup differences: Chi² = 0.11, df = 1 (P = 0.74), I² = 0%						

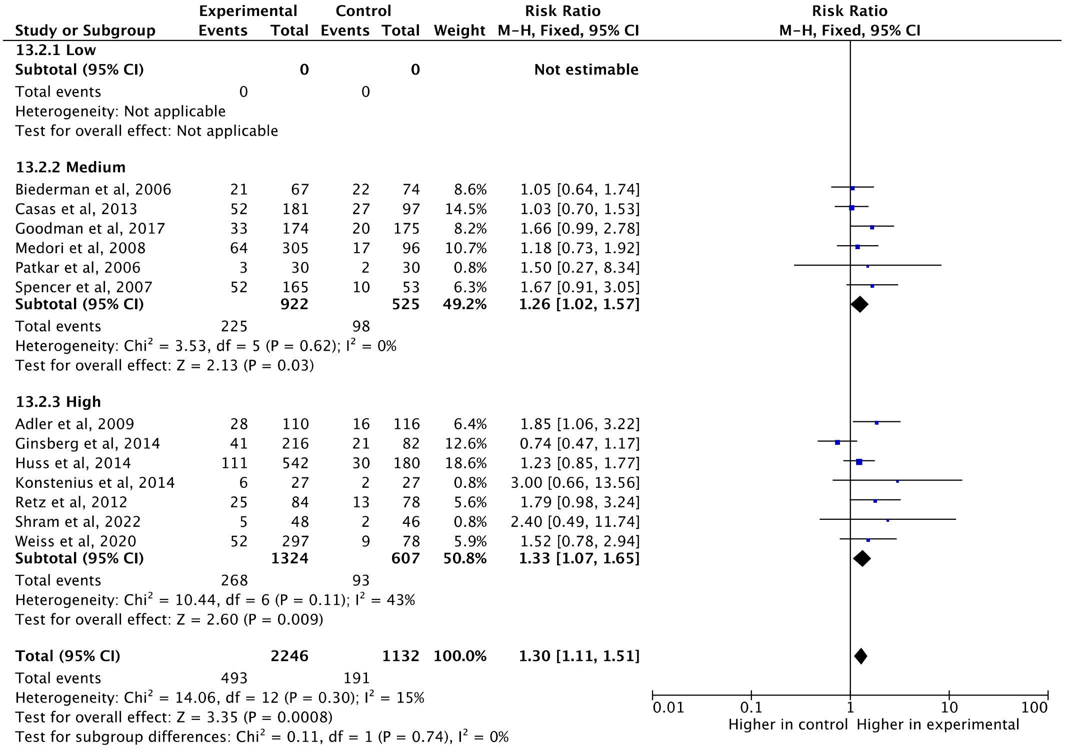

Figure legend: 95%CI, 95% confidence interval; M-H, Mantel–Haenszel. No study encompassing low dosage was included in the meta-analysis.

eFigure 59. Forest plot showing the risk ratio of **insomnia** between control and experimental groups in **adults** using **methylphenidate** subdivided by stimulant dosage.

Study or Subgroup	Experimental Events	Experimental Total	Control Events	Control Total	Weight	Risk Ratio M-H, Fixed, 95% CI
**13.3.1 Low**						
**Subtotal (95% CI)**		**0**		**0**		**Not estimable**
Total events	0		0			
Heterogeneity: Not applicable						
Test for overall effect: Not applicable						
**13.3.2 Medium**						
Biederman et al, 2006	12	67	4	74	5.6%	3.31 [1.12, 9.78]
Casas et al, 2013	28	181	11	97	21.1%	1.36 [0.71, 2.62]
Medori et al, 2008	41	305	7	96	15.7%	1.84 [0.86, 3.97]
Patkar et al, 2006	3	30	3	30	4.4%	1.00 [0.22, 4.56]
Rösler et al, 2009	46	183	12	66	26.0%	1.38 [0.78, 2.44]
Spencer et al, 2007	27	165	6	53	13.4%	1.45 [0.63, 3.31]
**Subtotal (95% CI)**		**931**		**416**	**86.1%**	**1.58 [1.15, 2.16]**
Total events	157		43			
Heterogeneity: $Chi^2 = 2.75$, df = 5 (P = 0.74); $I^2 = 0\%$						
Test for overall effect: Z = 2.82 (P = 0.005)						
**13.3.3 High**						
Adler et al, 2009	10	110	6	116	8.6%	1.76 [0.66, 4.67]
Konstenius et al, 2014	6	27	2	27	2.9%	3.00 [0.66, 13.56]
Weiss et al, 2020	18	297	1	78	2.3%	4.73 [0.64, 34.87]
**Subtotal (95% CI)**		**434**		**221**	**13.9%**	**2.52 [1.17, 5.43]**
Total events	34		9			
Heterogeneity: $Chi^2 = 0.95$, df = 2 (P = 0.62); $I^2 = 0\%$						
Test for overall effect: Z = 2.36 (P = 0.02)						
**Total (95% CI)**		**1365**		**637**	**100.0%**	**1.71 [1.28, 2.29]**
Total events	191		52			
Heterogeneity: $Chi^2 = 4.63$, df = 8 (P = 0.80); $I^2 = 0\%$						
Test for overall effect: Z = 3.59 (P = 0.0003)						
Test for subgroup differences: $Chi^2 = 1.22$, df = 1 (P = 0.27), $I^2 = 18.2\%$						

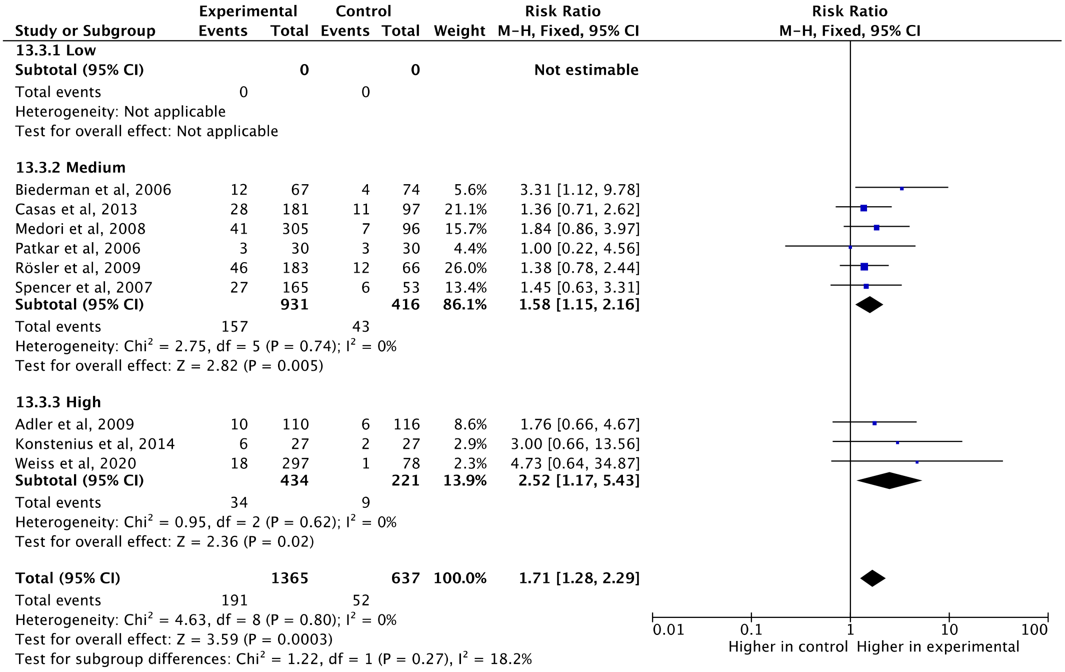

Figure legend: 95%CI, 95% confidence interval; M-H, Mantel–Haenszel. No study encompassing low dosage was included in the meta-analysis.

eFigure 60. Forest plot showing the risk ratio of **irritability** between control and experimental groups in **adults** using **methylphenidate** subdivided by stimulant dosage.

Study or Subgroup	Experimental Events	Experimental Total	Control Events	Control Total	Weight	Risk Ratio M-H, Fixed, 95% CI
**13.4.1 Low**						
**Subtotal (95% CI)**		**0**		**0**		**Not estimable**
Total events	0		0			
Heterogeneity: Not applicable						
Test for overall effect: Not applicable						
**13.4.2 Medium**						
Medori et al, 2008	17	305	1	96	15.5%	5.35 [0.72, 39.68]
**Subtotal (95% CI)**		**305**		**96**	**15.5%**	**5.35 [0.72, 39.68]**
Total events	17		1			
Heterogeneity: Not applicable						
Test for overall effect: Z = 1.64 (P = 0.10)						
**13.4.3 High**						
Adler et al, 2009	7	110	2	116	19.9%	3.69 [0.78, 17.38]
Weiss et al, 2020	16	297	4	78	64.6%	1.05 [0.36, 3.05]
**Subtotal (95% CI)**		**407**		**194**	**84.5%**	**1.67 [0.72, 3.89]**
Total events	23		6			
Heterogeneity: $Chi^2 = 1.73$, df = 1 (P = 0.19); $I^2 = 42\%$						
Test for overall effect: Z = 1.19 (P = 0.23)						
**Total (95% CI)**		**712**		**290**	**100.0%**	**2.24 [1.03, 4.89]**
Total events	40		7			
Heterogeneity: $Chi^2 = 3.06$, df = 2 (P = 0.22); $I^2 = 35\%$						
Test for overall effect: Z = 2.03 (P = 0.04)						
Test for subgroup differences: $Chi^2 = 1.10$, df = 1 (P = 0.29), $I^2 = 9.1\%$						

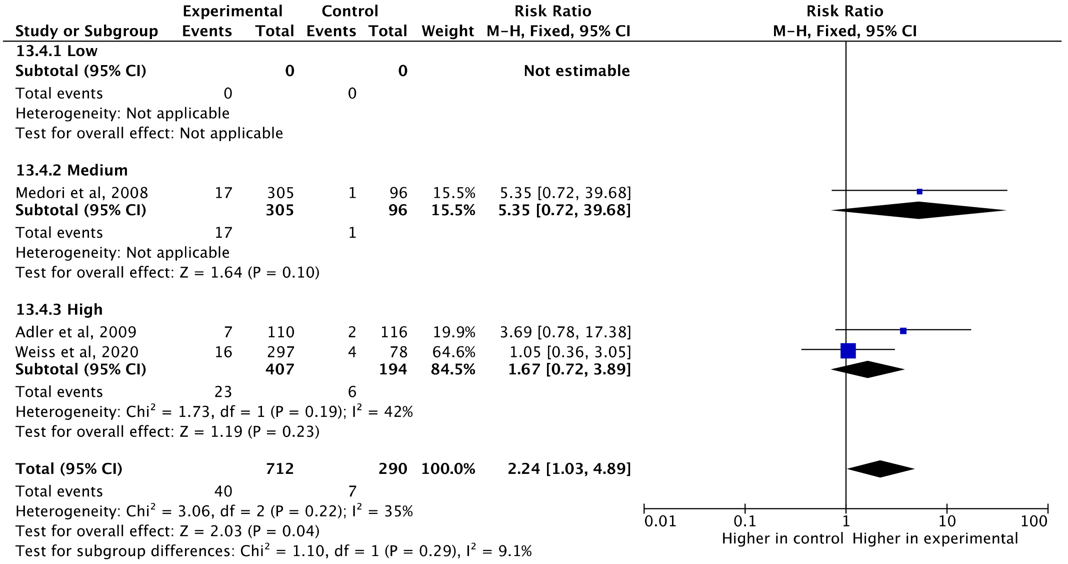

Figure legend: 95%CI, 95% confidence interval; M-H, Mantel–Haenszel. No study encompassing low dosage was included in the meta-analysis.

eFigure 61. Forest plot showing the risk ratio of **nausea** between control and experimental groups in **adults** using **methylphenidate** subdivided by stimulant dosage.

Study or Subgroup	Experimental Events	Experimental Total	Control Events	Control Total	Weight	Risk Ratio M-H, Fixed, 95% CI
**13.11.1 Low**						
**Subtotal (95% CI)**		**0**		**0**		**Not estimable**
Total events	0		0			
Heterogeneity: Not applicable						
Test for overall effect: Not applicable						
**13.11.2 Medium**						
Casas et al, 2013	31	181	8	97	24.1%	2.08 [0.99, 4.34]
Medori et al, 2008	39	305	4	96	14.1%	3.07 [1.13, 8.37]
Patkar et al, 2006	2	30	1	30	2.3%	2.00 [0.19, 20.90]
Rösler et al, 2009	16	183	2	66	6.8%	2.89 [0.68, 12.21]
**Subtotal (95% CI)**		**699**		**289**	**47.4%**	**2.48 [1.45, 4.25]**
Total events	88		15			
Heterogeneity: $Chi^2 = 0.47$, df = 3 (P = 0.93); $I^2 = 0\%$						
Test for overall effect: Z = 3.32 (P = 0.0009)						
**13.11.3 High**						
Adler et al, 2009	14	110	3	116	6.8%	4.92 [1.45, 16.66]
Huss et al, 2014	58	542	9	180	31.3%	2.14 [1.08, 4.23]
Retz et al, 2012	14	84	3	78	7.2%	4.33 [1.29, 14.51]
Weiss et al, 2020	18	297	2	78	7.3%	2.36 [0.56, 9.97]
**Subtotal (95% CI)**		**1033**		**452**	**52.6%**	**2.83 [1.73, 4.63]**
Total events	104		17			
Heterogeneity: $Chi^2 = 1.97$, df = 3 (P = 0.58); $I^2 = 0\%$						
Test for overall effect: Z = 4.14 (P < 0.0001)						
**Total (95% CI)**		**1732**		**741**	**100.0%**	**2.67 [1.85, 3.83]**
Total events	192		32			
Heterogeneity: $Chi^2 = 2.60$, df = 7 (P = 0.92); $I^2 = 0\%$						
Test for overall effect: Z = 5.30 (P < 0.00001)						
Test for subgroup differences: $Chi^2 = 0.12$, df = 1 (P = 0.73), $I^2 = 0\%$						

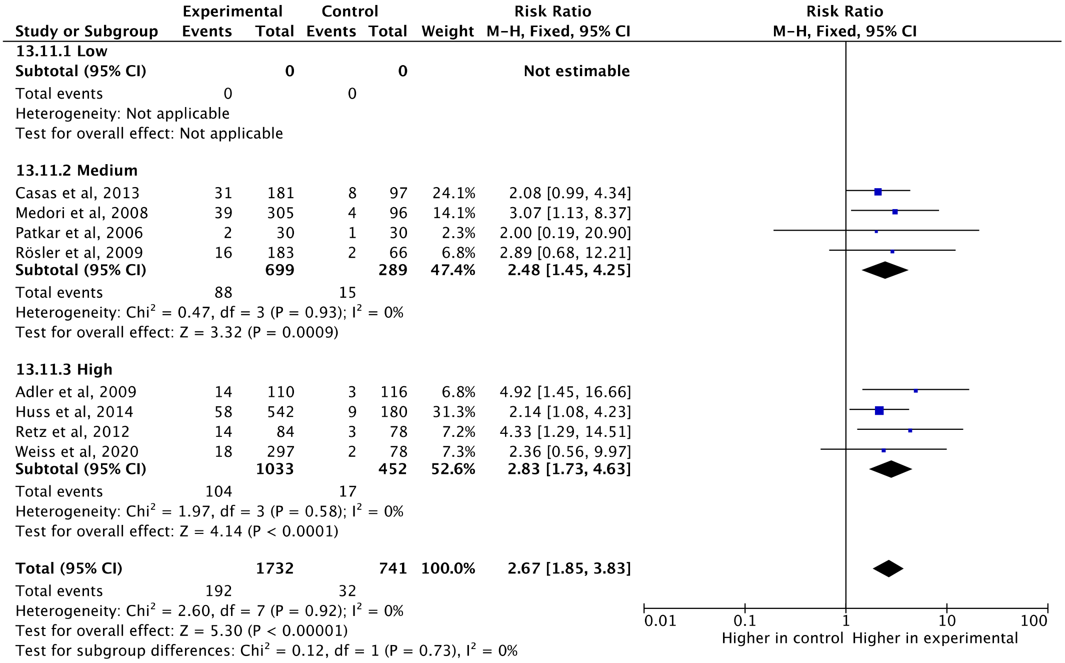

Figure legend: 95%CI, 95% confidence interval; M-H, Mantel–Haenszel. No study encompassing low dosage was included in the meta-analysis.

eFigure 62. Forest plot showing the mean differences in **heart rate** between control and experimental groups using **methylphenidate** in **adults** subdivided by stimulant dosage.

Study or Subgroup	Experimental Mean	Experimental SD	Experimental Total	Control Mean	Control SD	Control Total	Weight	Mean Difference IV, Random, 95% CI
**13.9.1 Low**								
**Subtotal (95% CI)**			**0**			**0**		**Not estimable**
Heterogeneity: Not applicable								
Test for overall effect: Not applicable								
**13.9.2 Medium**								
Biederman et al, 2006	4.5	10.5	67	−2.7	12.4	74	36.0%	7.20 [3.42, 10.98]
Hegerl et al, 2018	−6.24	14.66	22	−0.26	9.64	20	23.2%	−5.98 [−13.42, 1.46]
**Subtotal (95% CI)**			**89**			**94**	**59.3%**	**1.02 [−11.88, 13.91]**
Heterogeneity: $Tau^2 = 77.79$; $Chi^2 = 9.58$, df = 1 (P = 0.002); $I^2 = 90\%$								
Test for overall effect: Z = 0.15 (P = 0.88)								
**13.9.3 High**								
Adler et al, 2009	3.6	9.78	110	−1.6	8.33	116	40.7%	5.20 [2.83, 7.57]
**Subtotal (95% CI)**			**110**			**116**	**40.7%**	**5.20 [2.83, 7.57]**
Heterogeneity: Not applicable								
Test for overall effect: Z = 4.29 (P < 0.0001)								
**Total (95% CI)**			**199**			**210**	**100.0%**	**3.32 [−1.87, 8.51]**
Heterogeneity: $Tau^2 = 15.75$; $Chi^2 = 9.69$, df = 2 (P = 0.008); $I^2 = 79\%$								
Test for overall effect: Z = 1.25 (P = 0.21)								
Test for subgroup differences: $Chi^2 = 0.39$, df = 1 (P = 0.53), $I^2 = 0\%$								

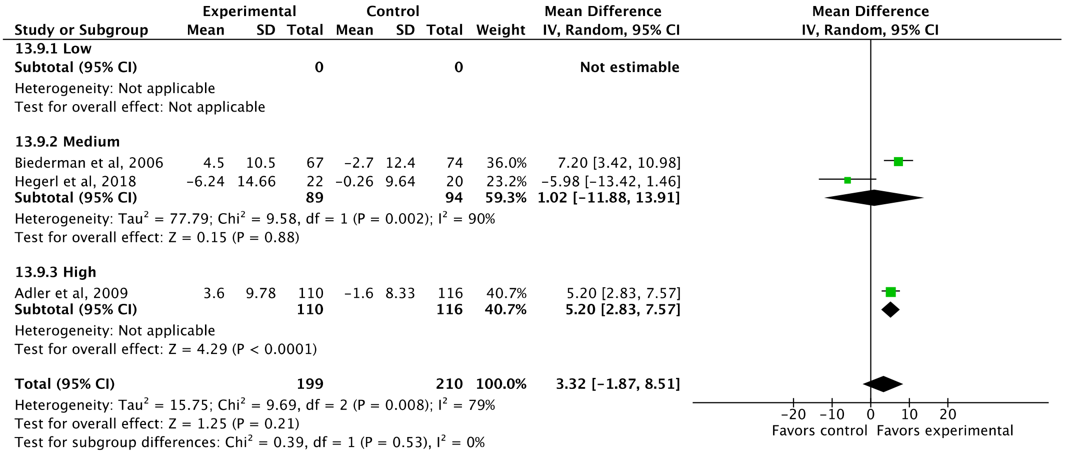

Figure legend: 95%CI, 95% confidence interval; IV, inverse variance; MPH, methylphenidate; SD, standard deviation. No study encompassing low dosage was included in the meta-analysis.

eFigure 63. Forest plot showing the mean differences in **diastolic blood pressure** between control and experimental groups using **methylphenidate** in **adults** subdivided by stimulant dosage.

Study or Subgroup	Experimental Mean	Experimental SD	Experimental Total	Control Mean	Control SD	Control Total	Weight	Mean Difference IV, Random, 95% CI
**13.8.1 Low**								
**Subtotal (95% CI)**			**0**			**0**		**Not estimable**
Heterogeneity: Not applicable								
Test for overall effect: Not applicable								
**13.8.2 Medium**								
Biederman et al, 2006	4	8.5	67	−2.1	8.9	74	38.8%	6.10 [3.23, 8.97]
Hegerl et al, 2018	0.86	18.67	22	9.79	10.79	20	19.8%	−8.93 [−18.05, 0.19]
**Subtotal (95% CI)**			**89**			**94**	**58.6%**	**−0.77 [−15.44, 13.91]**
Heterogeneity: $Tau^2 = 101.04$; $Chi^2 = 9.49$, df = 1 (P = 0.002); $I^2 = 89\%$								
Test for overall effect: Z = 0.10 (P = 0.92)								
**13.8.3 High**								
Adler et al, 2009	1.1	6.72	110	0.4	7.43	116	41.4%	0.70 [−1.15, 2.55]
**Subtotal (95% CI)**			**110**			**116**	**41.4%**	**0.70 [−1.15, 2.55]**
Heterogeneity: Not applicable								
Test for overall effect: Z = 0.74 (P = 0.46)								
**Total (95% CI)**			**199**			**210**	**100.0%**	**0.89 [−4.61, 6.39]**
Heterogeneity: $Tau^2 = 18.14$; $Chi^2 = 15.24$, df = 2 (P = 0.0005); $I^2 = 87\%$								
Test for overall effect: Z = 0.32 (P = 0.75)								
Test for subgroup differences: $Chi^2 = 0.04$, df = 1 (P = 0.85), $I^2 = 0\%$								

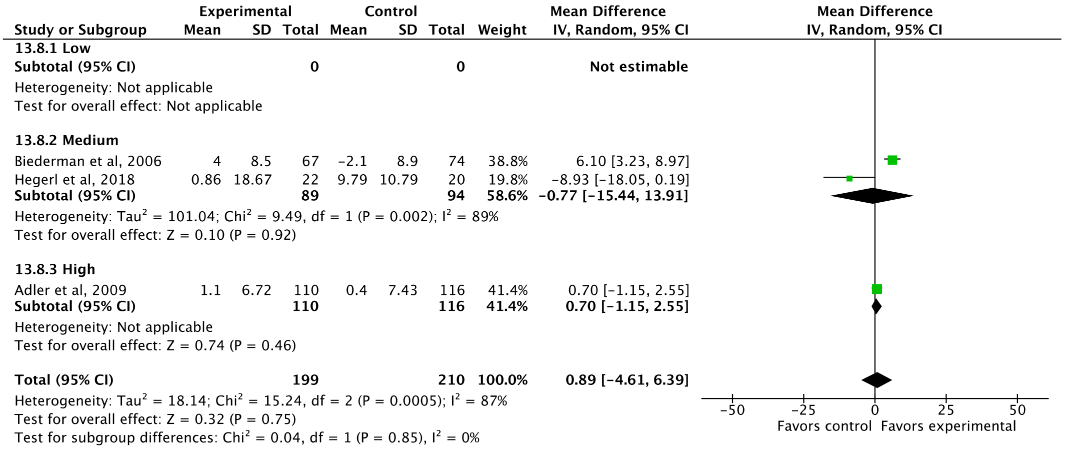

Figure legend: 95%CI, 95% confidence interval; IV, inverse variance; MPH, methylphenidate; SD, standard deviation. No study encompassing low dosage was included in the meta-analysis.

eFigure 64. Forest plot showing the mean differences in **systolic blood pressure** between control and experimental groups using **methylphenidate** in **adults** subdivided by stimulant dosage.

	Experimental			Control				Mean Difference
**Study or Subgroup**	**Mean**	**SD**	**Total**	**Mean**	**SD**	**Total**	**Weight**	**IV, Random, 95% CI**
**13.7.1 Low**								
**Subtotal (95% CI)**			**0**			**0**		**Not estimable**
Heterogeneity: Not applicable								
Test for overall effect: Not applicable								
**13.7.2 Medium**								
Biederman et al, 2006	3.5	11.8	67	−1.1	11.4	74	37.4%	4.60 [0.76, 8.44]
Hegerl et al, 2018	4.62	12.7	22	11.42	15.87	20	19.3%	−6.80 [−15.55, 1.95]
**Subtotal (95% CI)**			**89**			**94**	**56.8%**	**−0.39 [−11.48, 10.69]**
Heterogeneity: Tau² = 53.10; Chi² = 5.47, df = 1 (P = 0.02); I² = 82%								
Test for overall effect: Z = 0.07 (P = 0.94)								
**13.7.3 High**								
Adler et al, 2009	−1.2	8.92	110	−0.5	9.72	116	43.2%	−0.70 [−3.13, 1.73]
**Subtotal (95% CI)**			**110**			**116**	**43.2%**	**−0.70 [−3.13, 1.73]**
Heterogeneity: Not applicable								
Test for overall effect: Z = 0.56 (P = 0.57)								
**Total (95% CI)**			**199**			**210**	**100.0%**	**0.10 [−4.87, 5.08]**
Heterogeneity: Tau² = 13.37; Chi² = 7.99, df = 2 (P = 0.02); I² = 75%								
Test for overall effect: Z = 0.04 (P = 0.97)								
Test for subgroup differences: Chi² = 0.00, df = 1 (P = 0.96), I² = 0%								

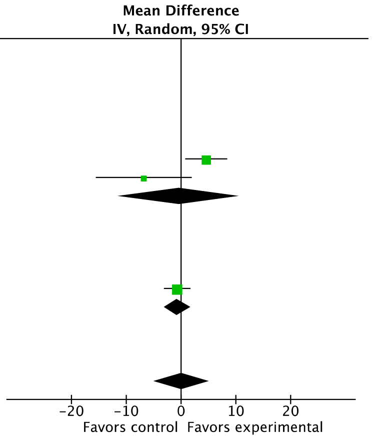

Figure legend: 95%CI, 95% confidence interval; IV, inverse variance; MPH, methylphenidate; SD, standard deviation. No study encompassing low dosage was included in the meta-analysis.

eFigure 65. Forest plot showing the risk ratio of **all adverse events** between control and experimental groups in **children** using **lisdexamfetamine** subdivided by stimulant dosage.

Study or Subgroup	Experimental Events	Experimental Total	Control Events	Control Total	Weight	Risk Ratio M-H, Fixed, 95% CI
**17.10.1 Low**						
Childress et al, 2022	68	146	19	45	16.2%	1.10 [0.75, 1.62]
**Subtotal (95% CI)**		**146**		**45**	**16.2%**	**1.10 [0.75, 1.62]**
Total events	68		19			
Heterogeneity: Not applicable						
Test for overall effect: Z = 0.50 (P = 0.62)						
**17.10.2 Medium**						
Coghill et al, 2013 - LDX	80	111	63	110	35.2%	1.26 [1.03, 1.53]
Coghill et al, 2014	31	78	20	79	11.1%	1.57 [0.98, 2.50]
Findling et al, 2011	160	232	45	77	37.6%	1.18 [0.96, 1.45]
**Subtotal (95% CI)**		**421**		**266**	**83.8%**	**1.26 [1.10, 1.45]**
Total events	271		128			
Heterogeneity: Chi^2 = 1.25, df = 2 (P = 0.53); I^2 = 0%						
Test for overall effect: Z = 3.28 (P = 0.001)						
**17.10.3 High**						
**Subtotal (95% CI)**		**0**		**0**		**Not estimable**
Total events	0		0			
Heterogeneity: Not applicable						
Test for overall effect: Not applicable						
**Total (95% CI)**		**567**		**311**	**100.0%**	**1.24 [1.08, 1.41]**
Total events	339		147			
Heterogeneity: Chi^2 = 1.57, df = 3 (P = 0.67); I^2 = 0%						
Test for overall effect: Z = 3.16 (P = 0.002)						
Test for subgroup differences: Chi^2 = 0.43, df = 1 (P = 0.51), I^2 = 0%						

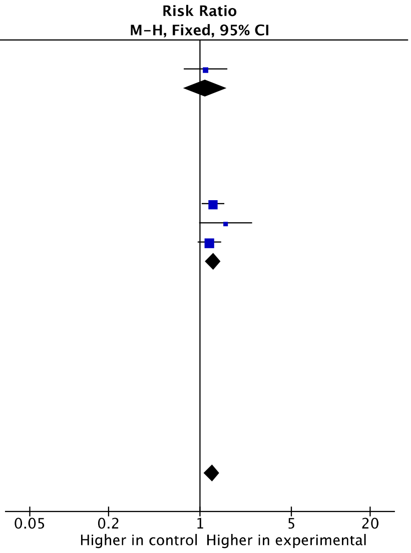

Figure legend: 95%CI, 95% confidence interval; LDX, lisdexamfetamine; M-H, Mantel–Haenszel. No study encompassing high dosage was included in the meta-analysis.

eFigure 66. Forest plot showing the risk ratio of **decreased appetite** between control and experimental groups in **children** using **lisdexamfetamine** subdivided by stimulant dosage.

Study or Subgroup	Experimental Events	Experimental Total	Control Events	Control Total	Weight	Risk Ratio M-H, Random, 95% CI
**17.1.1 Low**						
Childress et al, 2022	20	146	4	45	35.5%	1.54 [0.56, 4.27]
**Subtotal (95% CI)**		**146**		**45**	**35.5%**	**1.54 [0.56, 4.27]**
Total events	20		4			
Heterogeneity: Not applicable						
Test for overall effect: Z = 0.83 (P = 0.41)						
**17.1.2 Medium**						
Coghill et al, 2013 - LDX	28	111	3	110	33.7%	9.25 [2.90, 29.54]
Findling et al, 2011	79	232	2	77	30.9%	13.11 [3.30, 52.08]
**Subtotal (95% CI)**		**343**		**187**	**64.5%**	**10.69 [4.40, 25.98]**
Total events	107		5			
Heterogeneity: $Tau^2 = 0.00$; $Chi^2 = 0.15$, df = 1 (P = 0.70); $I^2 = 0\%$						
Test for overall effect: Z = 5.23 (P < 0.00001)						
**17.1.3 High**						
**Subtotal (95% CI)**		**0**		**0**		**Not estimable**
Total events	0		0			
Heterogeneity: Not applicable						
Test for overall effect: Not applicable						
**Total (95% CI)**		**489**		**232**	**100.0%**	**5.46 [1.30, 22.84]**
Total events	127		9			
Heterogeneity: $Tau^2 = 1.23$; $Chi^2 = 8.84$, df = 2 (P = 0.01); $I^2 = 77\%$						
Test for overall effect: Z = 2.32 (P = 0.02)						
Test for subgroup differences: $Chi^2 = 7.87$, df = 1 (P = 0.005), $I^2 = 87.3\%$						

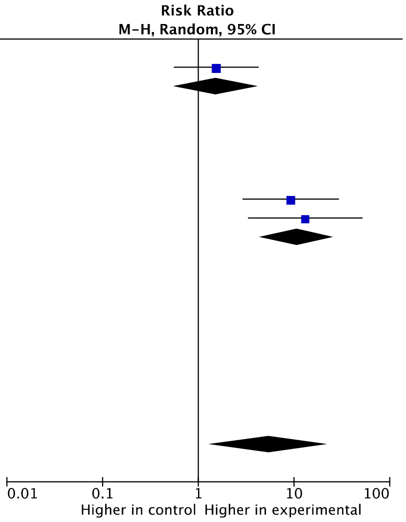

Figure legend: 95%CI, 95% confidence interval; LDX, lisdexamfetamine; M-H, Mantel–Haenszel. No study encompassing high dosage was included in the meta-analysis.

eFigure 67. Forest plot showing the risk ratio of **headache** between control and experimental groups in **children** using **lisdexamfetamine** subdivided by stimulant dosage.

Study or Subgroup	Experimental Events	Experimental Total	Control Events	Control Total	Weight	Risk Ratio M-H, Fixed, 95% CI
**17.2.1 Low**						
**Subtotal (95% CI)**		**0**		**0**		**Not estimable**
Total events	0		0			
Heterogeneity: Not applicable						
Test for overall effect: Not applicable						
**17.2.2 Medium**						
Coghill et al, 2013 - LDX	16	111	22	110	52.5%	0.72 [0.40, 1.30]
Coghill et al, 2014	6	78	5	79	11.8%	1.22 [0.39, 3.82]
Findling et al, 2011	34	232	10	77	35.7%	1.13 [0.59, 2.18]
**Subtotal (95% CI)**		**421**		**266**	**100.0%**	**0.92 [0.62, 1.39]**
Total events	56		37			
Heterogeneity: $Chi^2 = 1.26$, df = 2 (P = 0.53); $I^2 = 0\%$						
Test for overall effect: Z = 0.38 (P = 0.71)						
**17.2.3 High**						
**Subtotal (95% CI)**		**0**		**0**		**Not estimable**
Total events	0		0			
Heterogeneity: Not applicable						
Test for overall effect: Not applicable						
**Total (95% CI)**		**421**		**266**	**100.0%**	**0.92 [0.62, 1.39]**
Total events	56		37			
Heterogeneity: $Chi^2 = 1.26$, df = 2 (P = 0.53); $I^2 = 0\%$						
Test for overall effect: Z = 0.38 (P = 0.71)						
Test for subgroup differences: Not applicable						

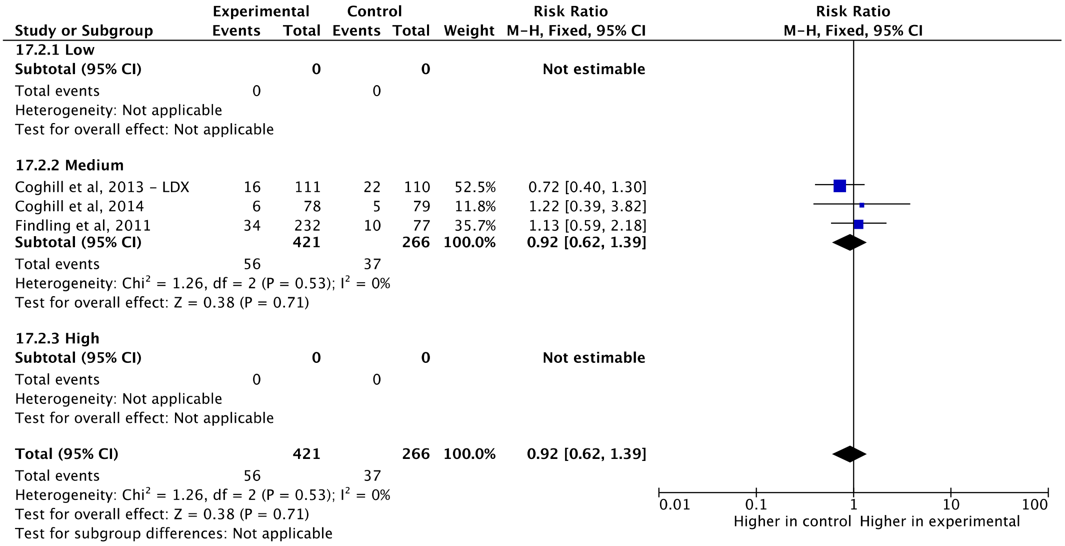

Figure legend: 95%CI, 95% confidence interval; LDX, lisdexamfetamine; M-H, Mantel–Haenszel. No study encompassing high dosage was included in the meta-analysis.

eFigure 68. Forest plot showing the mean differences in **heart rate** between control and experimental groups using **lisdexamfetamine** in **children** subdivided by stimulant dosage.

Study or Subgroup	Experimental Mean	Experimental SD	Experimental Total	Control Mean	Control SD	Control Total	Weight	Mean Difference IV, Random, 95% CI
**17.9.1 Low**								
Childress et al, 2022	2.7	10.79	146	1.9	9.9	45	17.6%	0.80 [−2.58, 4.18]
**Subtotal (95% CI)**			**146**			**45**	**17.6%**	**0.80 [−2.58, 4.18]**
Heterogeneity: Not applicable								
Test for overall effect: Z = 0.46 (P = 0.64)								
**17.9.2 Medium**								
Coghill et al, 2013 - LDX	5.7	15.3	222	−1.1	9.6	110	22.5%	6.80 [4.10, 9.50]
Coghill et al, 2014	6.1	12.59	78	2.3	9.29	79	17.1%	3.80 [0.34, 7.26]
Findling et al, 2011	5.4	1.27	78	0.8	1.36	77	42.7%	4.60 [4.19, 5.01]
**Subtotal (95% CI)**			**378**			**266**	**82.4%**	**4.86 [3.68, 6.04]**
Heterogeneity: $Tau^2 = 0.44$; $Chi^2 = 2.73$, df = 2 (P = 0.26); $I^2 = 27\%$								
Test for overall effect: Z = 8.09 (P < 0.00001)								
**17.9.3 High**								
**Subtotal (95% CI)**			**0**			**0**		**Not estimable**
Heterogeneity: Not applicable								
Test for overall effect: Not applicable								
**Total (95% CI)**			**524**			**311**	**100.0%**	**4.29 [2.45, 6.13]**
Heterogeneity: $Tau^2 = 2.01$; $Chi^2 = 7.61$, df = 3 (P = 0.05); $I^2 = 61\%$								
Test for overall effect: Z = 4.57 (P < 0.00001)								
Test for subgroup differences: $Chi^2 = 4.94$, df = 1 (P = 0.03), $I^2 = 79.8\%$								

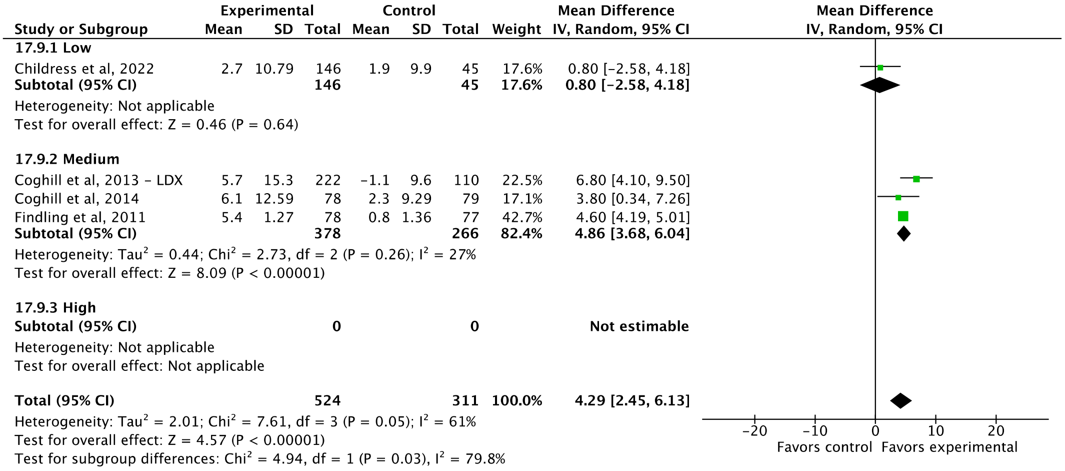

Figure legend: 95%CI, 95% confidence interval; IV, inverse variance; LDX, lisdexamfetamine; SD, standard deviation. No study encompassing high dosage was included in the meta-analysis.

eFigure 69. Forest plot showing the mean differences in **diastolic blood pressure** between control and experimental groups using **lisdexamfetamine** in **children** subdivided by stimulant dosage.

	Experimental			Control				Mean Difference
**Study or Subgroup**	**Mean**	**SD**	**Total**	**Mean**	**SD**	**Total**	**Weight**	**IV, Random, 95% CI**
**17.8.1 Low**								
Childress et al, 2022	1.7	5.9	146	0	6.88	45	23.5%	1.70 [−0.53, 3.93]
**Subtotal (95% CI)**			**146**			**45**	**23.5%**	**1.70 [−0.53, 3.93]**
Heterogeneity: Not applicable								
Test for overall effect: Z = 1.50 (P = 0.13)								
**17.8.2 Medium**								
Coghill et al, 2013 – LDX	0.2	9.6	222	1.2	8.7	110	24.6%	−1.00 [−3.06, 1.06]
Coghill et al, 2014	1.4	11.27	78	−0.1	8.12	79	18.5%	1.50 [−1.58, 4.58]
Findling et al, 2011	3.4	0.8	78	0.5	0.97	77	33.4%	2.90 [2.62, 3.18]
**Subtotal (95% CI)**			**378**			**266**	**76.5%**	**1.24 [−1.48, 3.97]**
Heterogeneity: $Tau^2 = 4.76$; $Chi^2 = 14.25$, df = 2 (P = 0.0008); $I^2 = 86\%$								
Test for overall effect: Z = 0.89 (P = 0.37)								
**17.8.3 High**								
**Subtotal (95% CI)**			**0**			**0**		**Not estimable**
Heterogeneity: Not applicable								
Test for overall effect: Not applicable								
**Total (95% CI)**			**524**			**311**	**100.0%**	**1.40 [−0.57, 3.37]**
Heterogeneity: $Tau^2 = 3.01$; $Chi^2 = 15.20$, df = 3 (P = 0.002); $I^2 = 80\%$								
Test for overall effect: Z = 1.39 (P = 0.16)								
Test for subgroup differences: $Chi^2 = 0.07$, df = 1 (P = 0.80), $I^2 = 0\%$								

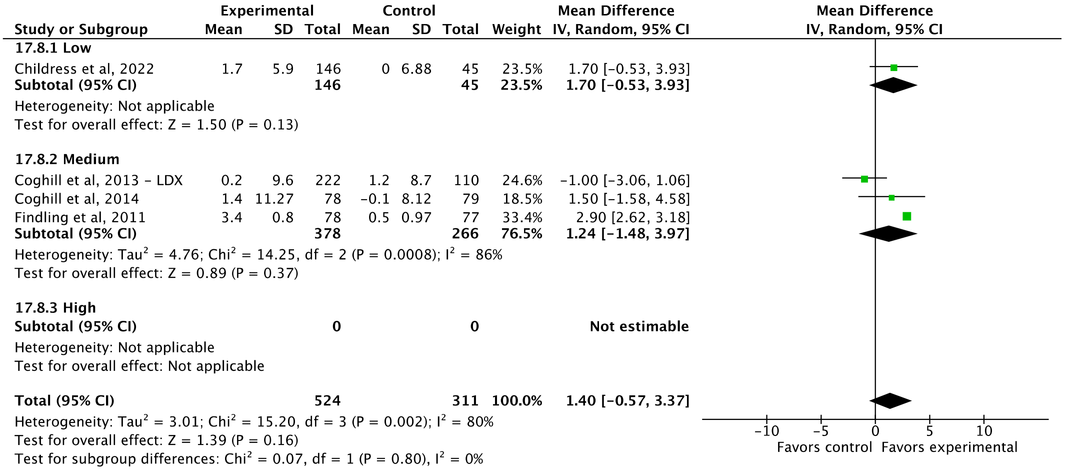

Figure legend: 95%CI, 95% confidence interval; IV, inverse variance; LDX, lisdexamfetamine; SD, standard deviation. No study encompassing high dosage was included in the meta-analysis.

eFigure 70. Forest plot showing the mean differences in **systolic blood pressure** between control and experimental groups using **lisdexamfetamine** in **children** subdivided by stimulant dosage.

Study or Subgroup	Experimental Mean	Experimental SD	Experimental Total	Control Mean	Control SD	Control Total	Weight	Mean Difference IV, Fixed, 95% CI
**17.7.1 Low**								
Childress et al, 2022	1	7.51	146	0.3	6.06	45	2.6%	0.70 [−1.45, 2.85]
**Subtotal (95% CI)**			**146**			**45**	**2.6%**	**0.70 [−1.45, 2.85]**
Heterogeneity: Not applicable								
Test for overall effect: Z = 0.64 (P = 0.52)								
**17.7.2 Medium**								
Coghill et al, 2013 - LDX	1	9.8	222	1	9.6	110	2.4%	0.00 [−2.21, 2.21]
Coghill et al, 2014	1.5	11.79	78	−0.6	9.66	79	1.0%	2.10 [−1.27, 5.47]
Findling et al, 2011	1.7	1.21	78	2.2	1.04	77	94.0%	−0.50 [−0.86, −0.14]
**Subtotal (95% CI)**			**378**			**266**	**97.4%**	**−0.46 [−0.81, −0.11]**
Heterogeneity: Chi^2 = 2.43, df = 2 (P = 0.30); I^2 = 18%								
Test for overall effect: Z = 2.58 (P = 0.010)								
**17.7.3 High**								
**Subtotal (95% CI)**			**0**			**0**		**Not estimable**
Heterogeneity: Not applicable								
Test for overall effect: Not applicable								
**Total (95% CI)**			**524**			**311**	**100.0%**	**−0.43 [−0.77, −0.09]**
Heterogeneity: Chi^2 = 3.52, df = 3 (P = 0.32); I^2 = 15%								
Test for overall effect: Z = 2.45 (P = 0.01)								
Test for subgroup differences: Chi^2 = 1.09, df = 1 (P = 0.30), I^2 = 8.3%								

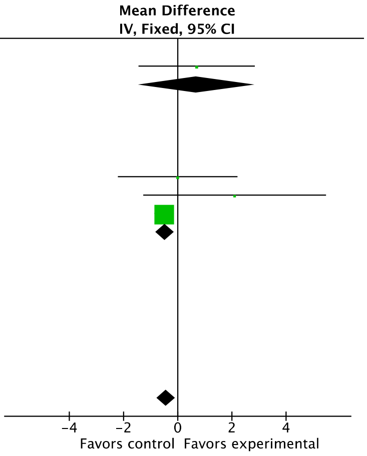

Figure legend: 95%CI, 95% confidence interval; IV, inverse variance; LDX, lisdexamfetamine; SD, standard deviation. No study encompassing high dosage was included in the meta-analysis.

eFigure 71. Forest plot showing the risk ratio of **all adverse events** between control and experimental groups in **adults** using **lisdexamfetamine** subdivided by stimulant dosage.

Study or Subgroup	Experimental Events	Experimental Total	Control Events	Control Total	Weight	Risk Ratio M-H, Fixed, 95% CI
**16.10.1 Short**						
Findling (2011)	160	232	45	77	37.6%	1.18 [0.96, 1.45]
**Subtotal (95% CI)**		**232**		**77**	**37.6%**	**1.18 [0.96, 1.45]**
Total events	160		45			
Heterogeneity: Not applicable						
Test for overall effect: Z = 1.57 (P = 0.12)						
**16.10.2 Medium**						
Childress (2022)	68	146	19	45	16.2%	1.10 [0.75, 1.62]
Coghill (2013) - LDX	80	111	63	110	35.2%	1.26 [1.03, 1.53]
**Subtotal (95% CI)**		**257**		**155**	**51.4%**	**1.21 [1.01, 1.45]**
Total events	148		82			
Heterogeneity: $Chi^2 = 0.37$, df = 1 (P = 0.54); $I^2 = 0\%$						
Test for overall effect: Z = 2.07 (P = 0.04)						
**16.10.3 Long**						
Coghill (2014)	31	78	20	79	11.1%	1.57 [0.98, 2.50]
**Subtotal (95% CI)**		**78**		**79**	**11.1%**	**1.57 [0.98, 2.50]**
Total events	31		20			
Heterogeneity: Not applicable						
Test for overall effect: Z = 1.89 (P = 0.06)						
**Total (95% CI)**		**567**		**311**	**100.0%**	**1.24 [1.08, 1.41]**
Total events	339		147			
Heterogeneity: $Chi^2 = 1.57$, df = 3 (P = 0.67); $I^2 = 0\%$						
Test for overall effect: Z = 3.16 (P = 0.002)						
Test for subgroup differences: $Chi^2 = 1.23$, df = 2 (P = 0.54), $I^2 = 0\%$						

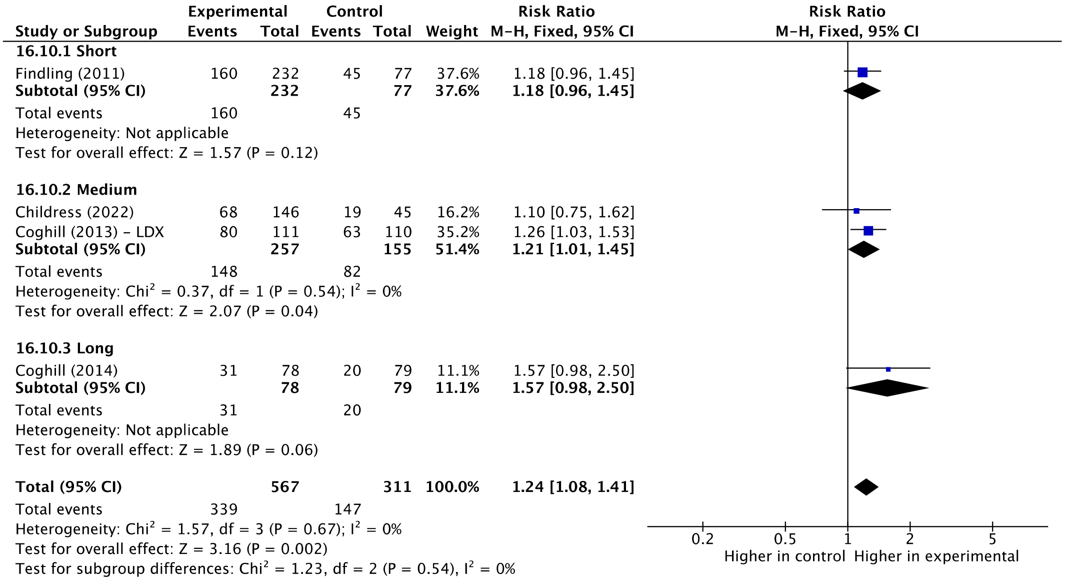

Figure legend: 95%CI, 95% confidence interval; LDX, lisdexamfetamine; M-H, Mantel–Haenszel.

eFigure 72. Forest plot showing the risk ratio of **anxiety** between control and experimental groups in **adults** using **lisdexamfetamine** subdivided by stimulant dosage.

	Experimental		Control			Risk Ratio
Study or Subgroup	Events	Total	Events	Total	Weight	M-H, Random, 95% CI
**14.6.1 Low**						
**Subtotal (95% CI)**		**0**		**0**		**Not estimable**
Total events	0		0			
Heterogeneity: Not applicable						
Test for overall effect: Not applicable						
**14.6.2 Medium**						
McElroy et al, 2015	9	196	5	63	55.9%	0.58 [0.20, 1.66]
Mooney et al, 2015	7	22	1	21	44.1%	6.68 [0.90, 49.78]
**Subtotal (95% CI)**		**218**		**84**	**100.0%**	**1.70 [0.14, 20.21]**
Total events	16		6			
Heterogeneity: $Tau^2 = 2.56$; $Chi^2 = 4.82$, df = 1 (P = 0.03); $I^2 = 79\%$						
Test for overall effect: Z = 0.42 (P = 0.67)						
**14.6.3 High**						
**Subtotal (95% CI)**		**0**		**0**		**Not estimable**
Total events	0		0			
Heterogeneity: Not applicable						
Test for overall effect: Not applicable						
**Total (95% CI)**		**218**		**84**	**100.0%**	**1.70 [0.14, 20.21]**
Total events	16		6			
Heterogeneity: $Tau^2 = 2.56$; $Chi^2 = 4.82$, df = 1 (P = 0.03); $I^2 = 79\%$						
Test for overall effect: Z = 0.42 (P = 0.67)						
Test for subgroup differences: Not applicable						

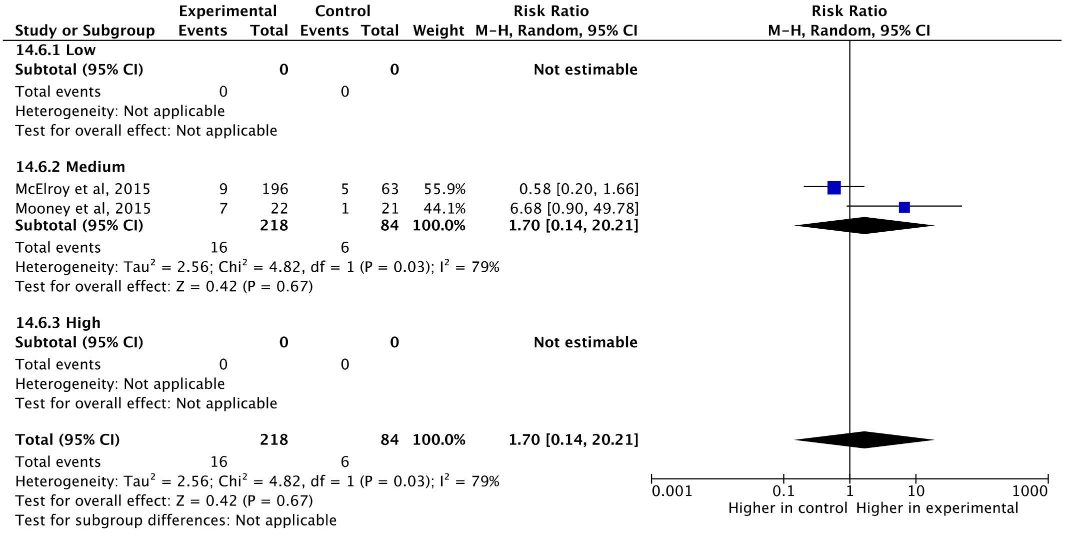

Figure legend: 95%CI, 95% confidence interval; M-H, Mantel–Haenszel. No study encompassing low or high dosages was included in the meta-analysis.

eFigure 73. Forest plot showing the risk ratio of **decreased appetite** between control and experimental groups in **adults** using **lisdexamfetamine** subdivided by stimulant dosage.

	Experimental		Control			Risk Ratio
**Study or Subgroup**	**Events**	**Total**	**Events**	**Total**	**Weight**	**M-H, Fixed, 95% CI**
**14.1.1 Low**						
**Subtotal (95% CI)**		**0**		**0**		**Not estimable**
Total events	0		0			
Heterogeneity: Not applicable						
Test for overall effect: Not applicable						
**14.1.2 Medium**						
Martin et al, 2014B - LDX	3	18	1	17	6.3%	2.83 [0.33, 24.66]
McElroy et al, 2015	42	196	4	63	37.3%	3.38 [1.26, 9.04]
Mooney et al, 2015	12	22	7	21	44.1%	1.64 [0.80, 3.35]
Wigal et al, 2010 - LDX	4	115	2	117	12.2%	2.03 [0.38, 10.89]
**Subtotal (95% CI)**		**351**		**218**	**100.0%**	**2.41 [1.38, 4.22]**
Total events	61		14			
Heterogeneity: $Chi^2 = 1.63$, df = 3 (P = 0.65); $I^2 = 0\%$						
Test for overall effect: Z = 3.07 (P = 0.002)						
**14.1.3 High**						
**Subtotal (95% CI)**		**0**		**0**		**Not estimable**
Total events	0		0			
Heterogeneity: Not applicable						
Test for overall effect: Not applicable						
**Total (95% CI)**		**351**		**218**	**100.0%**	**2.41 [1.38, 4.22]**
Total events	61		14			
Heterogeneity: $Chi^2 = 1.63$, df = 3 (P = 0.65); $I^2 = 0\%$						
Test for overall effect: Z = 3.07 (P = 0.002)						
Test for subgroup differences: Not applicable						

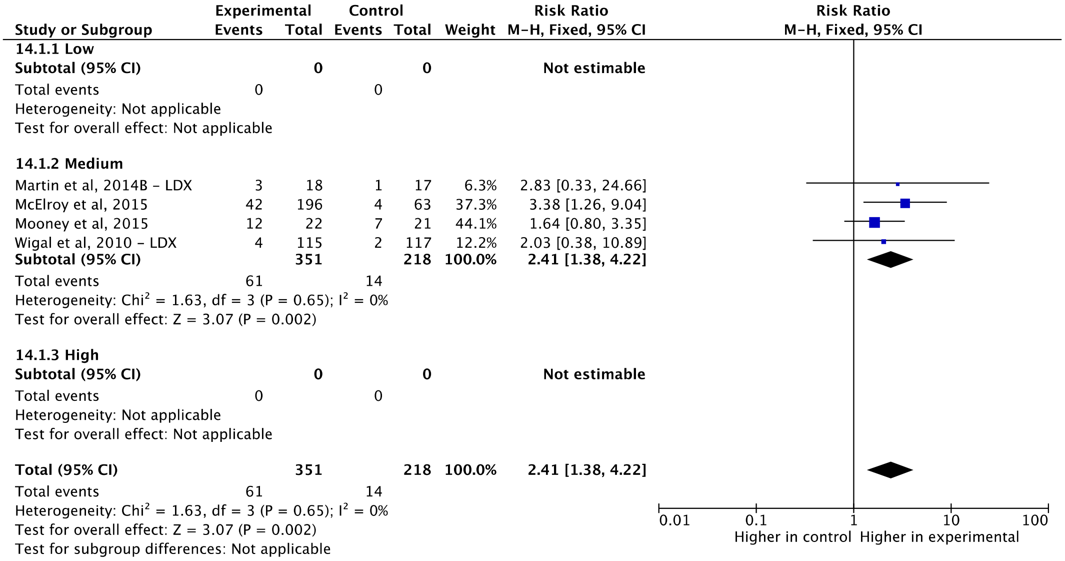

Figure legend: 95%CI, 95% confidence interval; LDX, lisdexamfetamine; M-H, Mantel–Haenszel. No study encompassing low or high dosages was included in the meta-analysis.

eFigure 74. Forest plot showing the risk ratio of **dry mouth** between control and experimental groups in **adults** using **lisdexamfetamine** subdivided by stimulant dosage.

Study or Subgroup	Experimental Events	Experimental Total	Control Events	Control Total	Weight	Risk Ratio M-H, Fixed, 95% CI
**14.5.1 Low**						
**Subtotal (95% CI)**		**0**		**0**		**Not estimable**
Total events	0		0			
Heterogeneity: Not applicable						
Test for overall effect: Not applicable						
**14.5.2 Medium**						
Martin et al, 2014B - LDX	6	18	3	17	26.5%	1.89 [0.56, 6.38]
McElroy et al, 2015	71	196	5	63	65.0%	4.56 [1.93, 10.80]
Wigal et al, 2010 - LDX	4	115	1	117	8.5%	4.07 [0.46, 35.86]
**Subtotal (95% CI)**		**329**		**197**	**100.0%**	**3.81 [1.93, 7.53]**
Total events	81		9			
Heterogeneity: $Chi^2 = 1.45$, df = 2 (P = 0.48); $I^2 = 0\%$						
Test for overall effect: Z = 3.85 (P = 0.0001)						
**14.5.3 High**						
**Subtotal (95% CI)**		**0**		**0**		**Not estimable**
Total events	0		0			
Heterogeneity: Not applicable						
Test for overall effect: Not applicable						
**Total (95% CI)**		**329**		**197**	**100.0%**	**3.81 [1.93, 7.53]**
Total events	81		9			
Heterogeneity: $Chi^2 = 1.45$, df = 2 (P = 0.48); $I^2 = 0\%$						
Test for overall effect: Z = 3.85 (P = 0.0001)						
Test for subgroup differences: Not applicable						

Risk Ratio M-H, Fixed, 95% CI

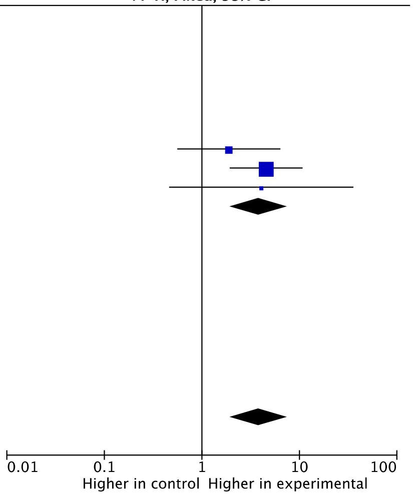

Figure legend: 95%CI, 95% confidence interval; LDX, lisdexamfetamine; M-H, Mantel–Haenszel. No study encompassing low or high dosages was included in the meta-analysis.

eFigure 75. Forest plot showing the risk ratio of **headache** between control and experimental groups in **adults** using **lisdexamfetamine** subdivided by stimulant dosage.

Study or Subgroup	Experimental Events	Experimental Total	Control Events	Control Total	Weight	Risk Ratio M-H, Fixed, 95% CI
**14.2.1 Low**						
**Subtotal (95% CI)**		**0**		**0**		**Not estimable**
Total events	0		0			
Heterogeneity: Not applicable						
Test for overall effect: Not applicable						
**14.2.2 Medium**						
Martin et al, 2014B - LDX	1	18	3	17	18.0%	0.31 [0.04, 2.74]
McElroy et al, 2015	23	196	6	63	52.8%	1.23 [0.53, 2.89]
Mooney et al, 2015	10	22	2	21	11.9%	4.77 [1.18, 19.27]
Wigal et al, 2010 - LDX	2	115	3	117	17.3%	0.68 [0.12, 3.98]
**Subtotal (95% CI)**		**351**		**218**	**100.0%**	**1.39 [0.77, 2.53]**
Total events	36		14			
Heterogeneity: $Chi^2 = 5.52$, df = 3 (P = 0.14); $I^2 = 46\%$						
Test for overall effect: Z = 1.09 (P = 0.27)						
**14.2.3 High**						
**Subtotal (95% CI)**		**0**		**0**		**Not estimable**
Total events	0		0			
Heterogeneity: Not applicable						
Test for overall effect: Not applicable						
**Total (95% CI)**		**351**		**218**	**100.0%**	**1.39 [0.77, 2.53]**
Total events	36		14			
Heterogeneity: $Chi^2 = 5.52$, df = 3 (P = 0.14); $I^2 = 46\%$						
Test for overall effect: Z = 1.09 (P = 0.27)						
Test for subgroup differences: Not applicable						

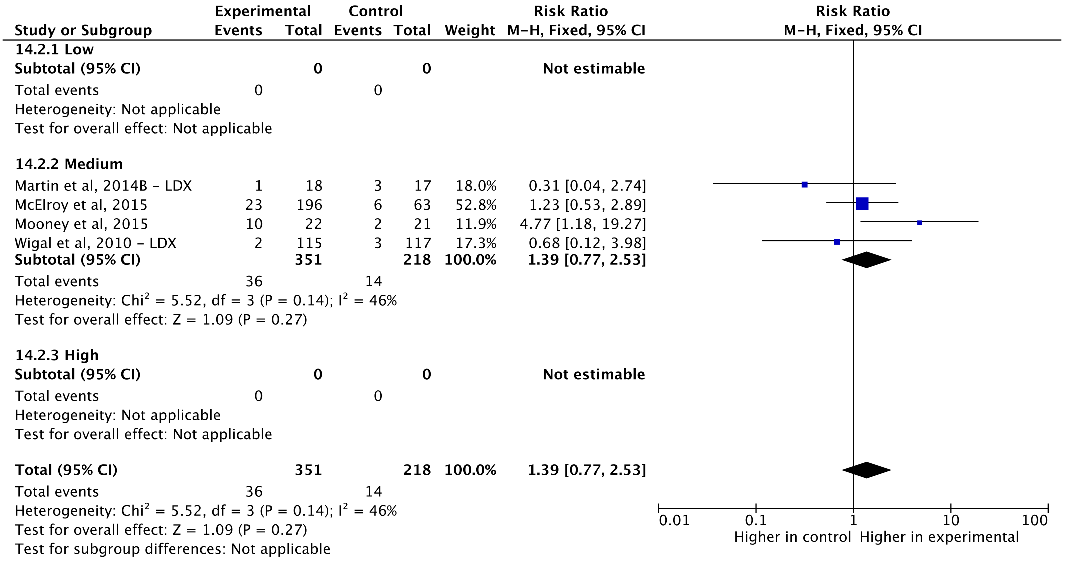

Figure legend: 95%CI, 95% confidence interval; LDX, lisdexamfetamine; M-H, Mantel–Haenszel. No study encompassing low or high dosages was included in the meta-analysis.

eFigure 76. Forest plot showing the risk ratio of **insomnia** between control and experimental groups in **adults** using **lisdexamfetamine** subdivided by stimulant dosage.

Study or Subgroup	Experimental Events	Experimental Total	Control Events	Control Total	Weight	Risk Ratio M-H, Fixed, 95% CI
**14.3.1 Low**						
**Subtotal (95% CI)**		**0**		**0**		**Not estimable**
Total events	0		0			
Heterogeneity: Not applicable						
Test for overall effect: Not applicable						
**14.3.2 Medium**						
McElroy et al, 2015	26	196	5	63	65.3%	1.67 [0.67, 4.17]
Mooney et al, 2015	7	22	2	21	17.6%	3.34 [0.78, 14.29]
Wigal et al, 2010 - LDX	3	115	2	117	17.1%	1.53 [0.26, 8.96]
**Subtotal (95% CI)**		**333**		**201**	**100.0%**	**1.94 [0.96, 3.91]**
Total events	36		9			
Heterogeneity: $Chi^2 = 0.71$, df = 2 (P = 0.70); $I^2 = 0\%$						
Test for overall effect: Z = 1.85 (P = 0.06)						
**14.3.3 High**						
**Subtotal (95% CI)**		**0**		**0**		**Not estimable**
Total events	0		0			
Heterogeneity: Not applicable						
Test for overall effect: Not applicable						
**Total (95% CI)**		**333**		**201**	**100.0%**	**1.94 [0.96, 3.91]**
Total events	36		9			
Heterogeneity: $Chi^2 = 0.71$, df = 2 (P = 0.70); $I^2 = 0\%$						
Test for overall effect: Z = 1.85 (P = 0.06)						
Test for subgroup differences: Not applicable						

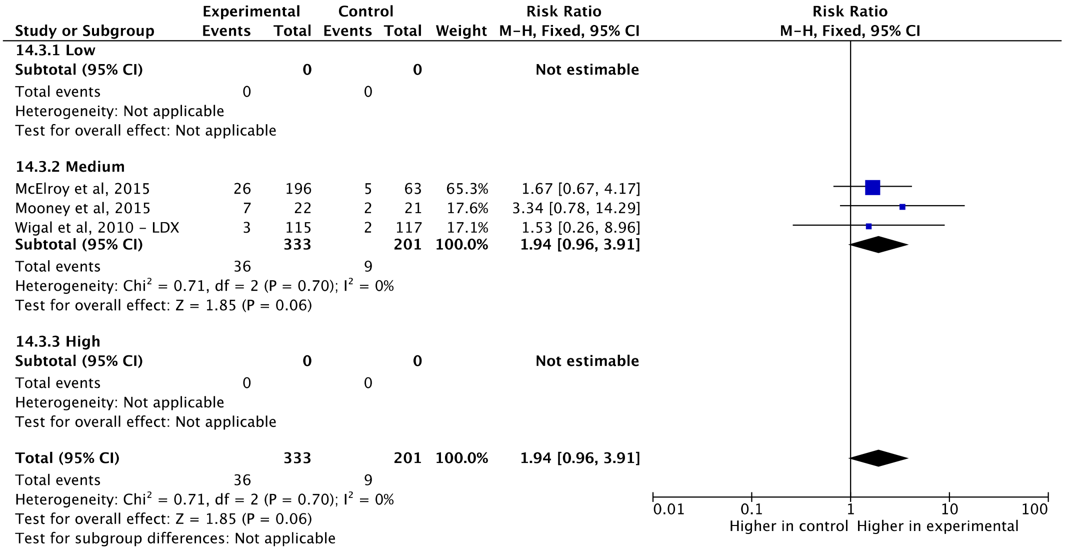

Figure legend: 95%CI, 95% confidence interval; LDX, lisdexamfetamine; M-H, Mantel–Haenszel. No study encompassing low or high dosages was included in the meta-analysis.

eFigure 77. Forest plot showing the mean differences in **heart rate** between control and experimental groups using **lisdexamfetamine** in **adults** subdivided by stimulant dosage.

	Experimental			Control				Mean Difference
Study or Subgroup	Mean	SD	Total	Mean	SD	Total	Weight	IV, Random, 95% CI
**16.9.1 Short**								
Findling (2011)	5.4	1.27	78	0.8	1.36	77	42.7%	4.60 [4.19, 5.01]
**Subtotal (95% CI)**			**78**			**77**	**42.7%**	**4.60 [4.19, 5.01]**
Heterogeneity: Not applicable								
Test for overall effect: Z = 21.76 (P < 0.00001)								
**16.9.2 Medium**								
Childress (2022)	2.7	10.79	146	1.9	9.9	45	17.6%	0.80 [−2.58, 4.18]
Coghill (2013) – LDX	5.7	15.3	222	−1.1	9.6	110	22.5%	6.80 [4.10, 9.50]
**Subtotal (95% CI)**			**368**			**155**	**40.2%**	**3.89 [−1.99, 9.77]**
Heterogeneity: $Tau^2 = 15.57$; $Chi^2 = 7.40$, df = 1 (P = 0.007); $I^2 = 86\%$								
Test for overall effect: Z = 1.30 (P = 0.19)								
**16.9.3 Long**								
Coghill (2014)	6.1	12.59	78	2.3	9.29	79	17.1%	3.80 [0.34, 7.26]
**Subtotal (95% CI)**			**78**			**79**	**17.1%**	**3.80 [0.34, 7.26]**
Heterogeneity: Not applicable								
Test for overall effect: Z = 2.15 (P = 0.03)								
**Total (95% CI)**			**524**			**311**	**100.0%**	**4.29 [2.45, 6.13]**
Heterogeneity: $Tau^2 = 2.01$; $Chi^2 = 7.61$, df = 3 (P = 0.05); $I^2 = 61\%$								
Test for overall effect: Z = 4.57 (P < 0.00001)								
Test for subgroup differences: $Chi^2 = 0.26$, df = 2 (P = 0.88), $I^2 = 0\%$								

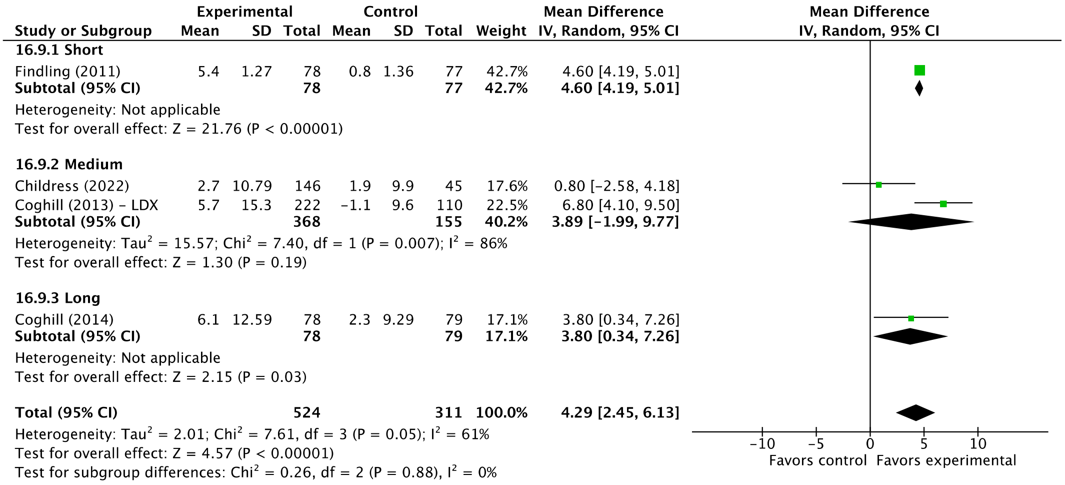

Figure legend: 95%CI, 95% confidence interval; IV, inverse variance; LDX, lisdexamfetamine; SD, standard deviation.

eFigure 78. Forest plot showing the mean differences in **diastolic blood pressure** between control and experimental groups using **lisdexamfetamine** in **adults** subdivided by stimulant dosage.

	Experimental			Control				Mean Difference
**Study or Subgroup**	**Mean**	**SD**	**Total**	**Mean**	**SD**	**Total**	**Weight**	**IV, Random, 95% CI**
**16.8.1 Short**								
Findling (2011)	3.4	0.8	78	0.5	0.97	77	33.4%	2.90 [2.62, 3.18]
**Subtotal (95% CI)**			**78**			**77**	**33.4%**	**2.90 [2.62, 3.18]**
Heterogeneity: Not applicable								
Test for overall effect: Z = 20.29 (P < 0.00001)								
**16.8.2 Medium**								
Childress (2022)	1.7	5.9	146	0	6.88	45	23.5%	1.70 [−0.53, 3.93]
Coghill (2013) - LDX	0.2	9.6	222	1.2	8.7	110	24.6%	−1.00 [−3.06, 1.06]
**Subtotal (95% CI)**			**368**			**155**	**48.1%**	**0.32 [−2.33, 2.96]**
Heterogeneity: Tau^2 = 2.45; Chi^2 = 3.05, df = 1 (P = 0.08); I^2 = 67%								
Test for overall effect: Z = 0.23 (P = 0.82)								
**16.8.3 Long**								
Coghill (2014)	1.4	11.27	78	−0.1	8.12	79	18.5%	1.50 [−1.58, 4.58]
**Subtotal (95% CI)**			**78**			**79**	**18.5%**	**1.50 [−1.58, 4.58]**
Heterogeneity: Not applicable								
Test for overall effect: Z = 0.96 (P = 0.34)								
**Total (95% CI)**			**524**			**311**	**100.0%**	**1.40 [−0.57, 3.37]**
Heterogeneity: Tau^2 = 3.01; Chi^2 = 15.20, df = 3 (P = 0.002); I^2 = 80%								
Test for overall effect: Z = 1.39 (P = 0.16)								
Test for subgroup differences: Chi^2 = 4.38, df = 2 (P = 0.11), I^2 = 54.4%								

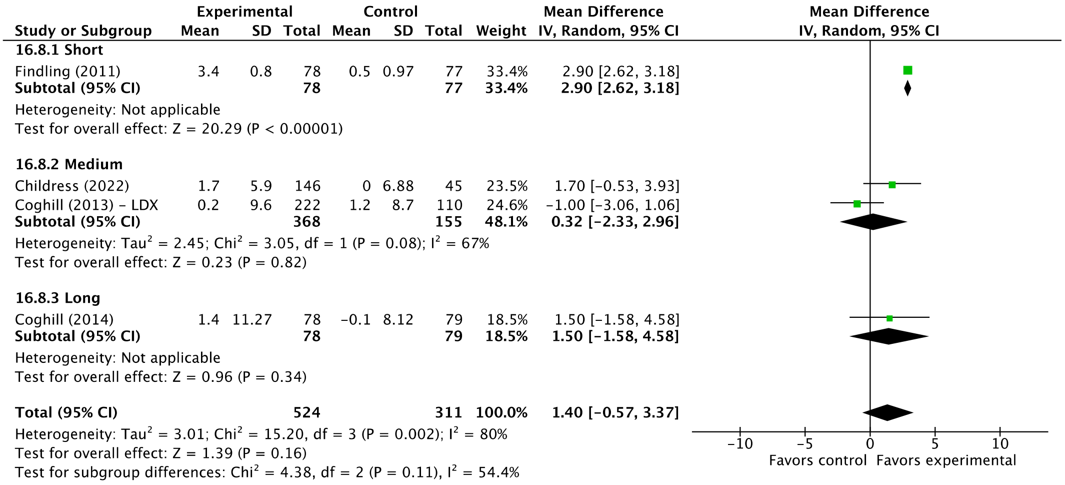

Figure legend: 95%CI, 95% confidence interval; IV, inverse variance; LDX, lisdexamfetamine; SD, standard deviation.

eFigure 79. Forest plot showing the mean differences in **systolic blood pressure** between control and experimental groups using **lisdexamfetamine** in **adults** subdivided by stimulant dosage.

Study or Subgroup	Experimental Mean	Experimental SD	Experimental Total	Control Mean	Control SD	Control Total	Weight	Mean Difference IV, Fixed, 95% CI
**16.7.1 Short**								
Findling (2011)	1.7	1.21	78	2.2	1.04	77	94.0%	−0.50 [−0.86, −0.14]
**Subtotal (95% CI)**			**78**			**77**	**94.0%**	**−0.50 [−0.86, −0.14]**
Heterogeneity: Not applicable								
Test for overall effect: Z = 2.76 (P = 0.006)								
**16.7.2 Medium**								
Childress (2022)	1	7.51	146	0.3	6.06	45	2.6%	0.70 [−1.45, 2.85]
Coghill (2013) - LDX	1	9.8	222	1	9.6	110	2.4%	0.00 [−2.21, 2.21]
**Subtotal (95% CI)**			**368**			**155**	**5.0%**	**0.36 [−1.18, 1.90]**
Heterogeneity: $Chi^2 = 0.20$, df = 1 (P = 0.66); $I^2 = 0\%$								
Test for overall effect: Z = 0.46 (P = 0.65)								
**16.7.3 Long**								
Coghill (2014)	1.5	11.79	78	−0.6	9.66	79	1.0%	2.10 [−1.27, 5.47]
**Subtotal (95% CI)**			**78**			**79**	**1.0%**	**2.10 [−1.27, 5.47]**
Heterogeneity: Not applicable								
Test for overall effect: Z = 1.22 (P = 0.22)								
**Total (95% CI)**			**524**			**311**	**100.0%**	**−0.43 [−0.77, −0.09]**
Heterogeneity: $Chi^2 = 3.52$, df = 3 (P = 0.32); $I^2 = 15\%$								
Test for overall effect: Z = 2.45 (P = 0.01)								
Test for subgroup differences: $Chi^2 = 3.32$, df = 2 (P = 0.19), $I^2 = 39.7\%$								

Mean Difference IV, Fixed, 95% CI

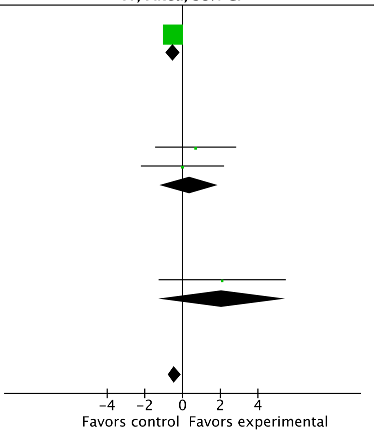

Figure legend: 95%CI, 95% confidence interval; IV, inverse variance; LDX, lisdexamfetamine; SD, standard deviation.

eFigure 80. Forest plot showing the risk ratio of **all adverse events** between control and experimental groups in **children** using **amphetamines** subdivided by stimulant dosage.

Study or Subgroup	Experimental Events	Experimental Total	Control Events	Control Total	Weight	Risk Ratio M-H, Random, 95% CI
**18.10.1 Low**						
Mattingly et al, 2020	11	45	7	43	13.5%	1.50 [0.64, 3.51]
**Subtotal (95% CI)**		**45**		**43**	**13.5%**	**1.50 [0.64, 3.51]**
Total events	11		7			
Heterogeneity: Not applicable						
Test for overall effect: Z = 0.94 (P = 0.35)						
**18.10.2 Medium**						
Brams et al, 2018	70	131	34	131	31.7%	2.06 [1.48, 2.87]
Childress et al, 2015	10	97	6	97	11.2%	1.67 [0.63, 4.41]
Childress et al, 2017	9	51	6	48	11.5%	1.41 [0.54, 3.67]
Cutler et al, 2022B	44	105	43	105	32.1%	1.02 [0.74, 1.41]
**Subtotal (95% CI)**		**384**		**381**	**86.5%**	**1.47 [0.93, 2.32]**
Total events	133		89			
Heterogeneity: $Tau^2 = 0.13$; $Chi^2 = 8.98$, df = 3 (P = 0.03); $I^2 = 67\%$						
Test for overall effect: Z = 1.67 (P = 0.10)						
**18.10.3 High**						
**Subtotal (95% CI)**		**0**		**0**		**Not estimable**
Total events	0		0			
Heterogeneity: Not applicable						
Test for overall effect: Not applicable						
**Total (95% CI)**		**429**		**424**	**100.0%**	**1.47 [1.01, 2.16]**
Total events	144		96			
Heterogeneity: $Tau^2 = 0.09$; $Chi^2 = 8.98$, df = 4 (P = 0.06); $I^2 = 55\%$						
Test for overall effect: Z = 2.00 (P = 0.05)						
Test for subgroup differences: $Chi^2 = 0.00$, df = 1 (P = 0.97), $I^2 = 0\%$						

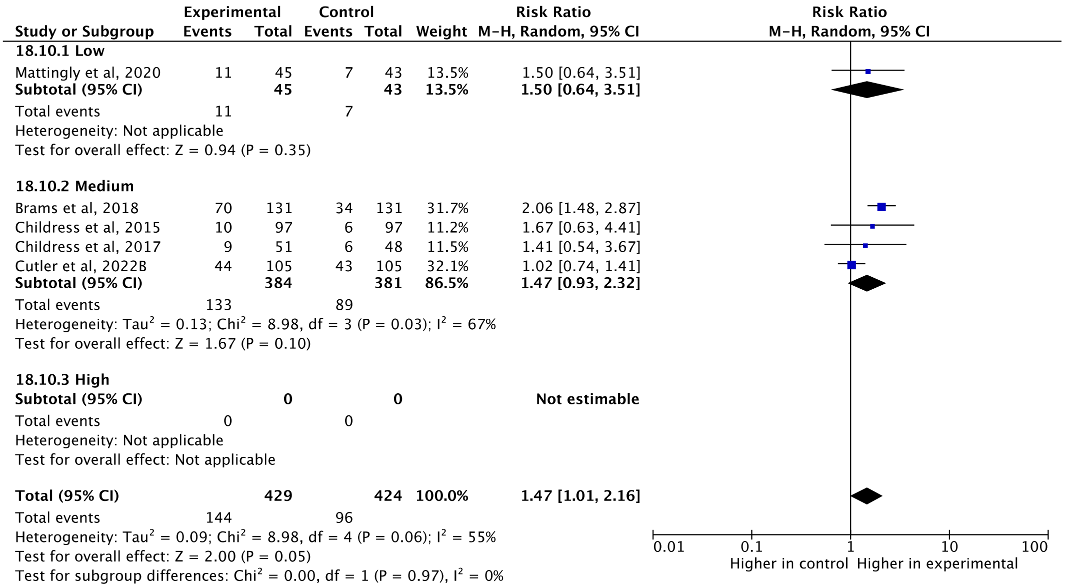

Figure legend: 95%CI, 95% confidence interval; M-H, Mantel–Haenszel.

eFigure 81. Forest plot showing the risk ratio of **anxiety** between control and experimental groups in **children** using **amphetamines** subdivided by stimulant dosage.

Study or Subgroup	Experimental Events	Experimental Total	Control Events	Control Total	Weight	Risk Ratio M-H, Fixed, 95% CI
**18.6.1 Low**						
**Subtotal (95% CI)**		**0**		**0**		**Not estimable**
Total events	0		0			
Heterogeneity: Not applicable						
Test for overall effect: Not applicable						
**18.6.2 Medium**						
McCracken et al, 2003	11	48	10	49	36.8%	1.12 [0.53, 2.40]
McCracken et al, 2003 - XR	9	49	10	49	37.2%	0.90 [0.40, 2.02]
Ramtvedt et al, 2014 - AMPH	8	34	7	34	26.0%	1.14 [0.47, 2.80]
**Subtotal (95% CI)**		**131**		**132**	**100.0%**	**1.05 [0.65, 1.67]**
Total events	28		27			
Heterogeneity: $Chi^2 = 0.20$, df = 2 (P = 0.90); $I^2 = 0\%$						
Test for overall effect: Z = 0.18 (P = 0.85)						
**18.6.3 High**						
**Subtotal (95% CI)**		**0**		**0**		**Not estimable**
Total events	0		0			
Heterogeneity: Not applicable						
Test for overall effect: Not applicable						
**Total (95% CI)**		**131**		**132**	**100.0%**	**1.05 [0.65, 1.67]**
Total events	28		27			
Heterogeneity: $Chi^2 = 0.20$, df = 2 (P = 0.90); $I^2 = 0\%$						
Test for overall effect: Z = 0.18 (P = 0.85)						
Test for subgroup differences: Not applicable						

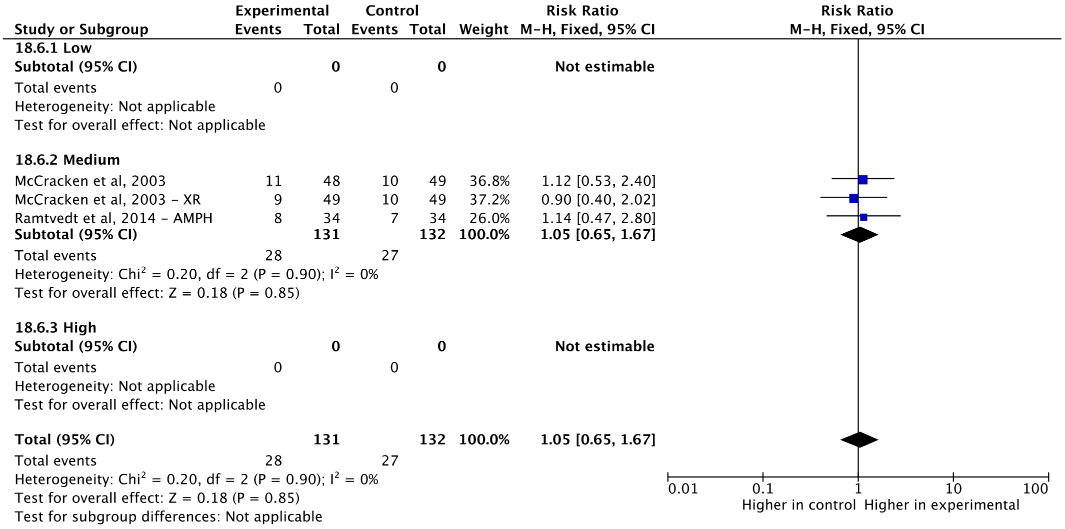

Figure legend: 95%CI, 95% confidence interval; AMPH, amphetamines; M-H, Mantel–Haenszel; XR, extended release. No study encompassing low or high dosages was included in the meta-analysis.

eFigure 82. Forest plot showing the risk ratio of **decreased appetite** between control and experimental groups in **children** using **amphetamines** subdivided by stimulant dosage.

Study or Subgroup	Experimental Events	Experimental Total	Control Events	Control Total	Weight	Risk Ratio M-H, Random, 95% CI
**18.1.1 Low**						
Mattingly et al, 2020	1	45	2	43	5.1%	0.48 [0.04, 5.08]
**Subtotal (95% CI)**		**45**		**43**	**5.1%**	**0.48 [0.04, 5.08]**
Total events	1		2			
Heterogeneity: Not applicable						
Test for overall effect: Z = 0.61 (P = 0.54)						
**18.1.2 Medium**						
Brams et al, 2018	40	132	9	131	18.2%	4.41 [2.23, 8.72]
Cutler et al, 2022B	13	105	2	105	9.9%	6.50 [1.50, 28.10]
McCracken et al, 2003	22	48	11	49	19.1%	2.04 [1.12, 3.74]
McCracken et al, 2003 - XR	27	49	11	49	19.4%	2.45 [1.38, 4.38]
Ramtvedt et al, 2014 - AMPH	24	34	17	34	21.4%	1.41 [0.95, 2.11]
Spencer et al, 2006	83	226	1	52	6.9%	19.10 [2.72, 134.04]
**Subtotal (95% CI)**		**594**		**420**	**94.9%**	**3.00 [1.61, 5.61]**
Total events	209		51			
Heterogeneity: $Tau^2 = 0.41$; $Chi^2 = 22.01$, df = 5 (P = 0.0005); $I^2 = 77\%$						
Test for overall effect: Z = 3.45 (P = 0.0006)						
**18.1.3 High**						
**Subtotal (95% CI)**		**0**		**0**		**Not estimable**
Total events	0		0			
Heterogeneity: Not applicable						
Test for overall effect: Not applicable						
**Total (95% CI)**		**639**		**463**	**100.0%**	**2.73 [1.49, 5.00]**
Total events	210		53			
Heterogeneity: $Tau^2 = 0.40$; $Chi^2 = 23.11$, df = 6 (P = 0.0008); $I^2 = 74\%$						
Test for overall effect: Z = 3.25 (P = 0.001)						
Test for subgroup differences: $Chi^2 = 2.17$, df = 1 (P = 0.14), $I^2 = 54.0\%$						

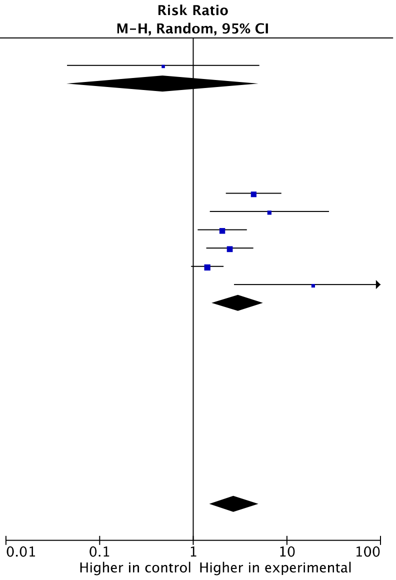

Figure legend: 95%CI, 95% confidence interval; AMPH, amphetamines; M-H, Mantel–Haenszel; XR, extended release. No study encompassing high dosage was included in the meta-analysis.

eFigure 83. Forest plot showing the risk ratio of **headache** between control and experimental groups in **children** using **amphetamines** subdivided by stimulant dosage.

Study or Subgroup	Experimental Events	Experimental Total	Control Events	Control Total	Weight	Risk Ratio M-H, Fixed, 95% CI
**18.2.1 Low**						
Mattingly et al, 2020	2	45	3	43	4.2%	0.64 [0.11, 3.63]
**Subtotal (95% CI)**		**45**		**43**	**4.2%**	**0.64 [0.11, 3.63]**
Total events	2		3			
Heterogeneity: Not applicable						
Test for overall effect: Z = 0.51 (P = 0.61)						
**18.2.2 Medium**						
Brams et al, 2018	16	132	14	131	19.1%	1.13 [0.58, 2.23]
Childress et al, 2017	1	51	1	48	1.4%	0.94 [0.06, 14.63]
Cutler et al, 2022B	6	105	4	105	5.4%	1.50 [0.44, 5.16]
McCracken et al, 2003	12	48	12	49	16.2%	1.02 [0.51, 2.04]
McCracken et al, 2003 - XR	12	49	12	49	16.3%	1.00 [0.50, 2.00]
Ramtvedt et al, 2014 - AMPH	8	34	8	34	10.9%	1.00 [0.42, 2.36]
Spencer et al, 2006	38	226	12	54	26.4%	0.76 [0.42, 1.35]
**Subtotal (95% CI)**		**645**		**470**	**95.8%**	**0.99 [0.74, 1.33]**
Total events	93		63			
Heterogeneity: Chi² = 1.43, df = 6 (P = 0.96); I² = 0%						
Test for overall effect: Z = 0.06 (P = 0.95)						
**18.2.3 High**						
**Subtotal (95% CI)**		**0**		**0**		**Not estimable**
Total events	0		0			
Heterogeneity: Not applicable						
Test for overall effect: Not applicable						
**Total (95% CI)**		**690**		**513**	**100.0%**	**0.98 [0.73, 1.31]**
Total events	95		66			
Heterogeneity: Chi² = 1.66, df = 7 (P = 0.98); I² = 0%						
Test for overall effect: Z = 0.16 (P = 0.87)						
Test for subgroup differences: Chi² = 0.24, df = 1 (P = 0.62), I² = 0%						

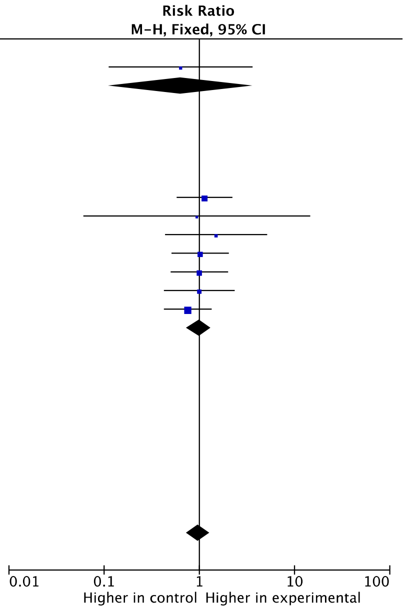

Figure legend: 95%CI, 95% confidence interval; AMPH, amphetamines; M-H, Mantel–Haenszel; XR, extended release. No study encompassing high dosage was included in the meta-analysis.

eFigure 84. Forest plot showing the risk ratio of **insomnia** between control and experimental groups in **children** using **amphetamines** subdivided by stimulant dosage.

	Experimental		Control			Risk Ratio
**Study or Subgroup**	**Events**	**Total**	**Events**	**Total**	**Weight**	**M-H, Fixed, 95% CI**
**18.3.1 Low**						
**Subtotal (95% CI)**		**0**		**0**		**Not estimable**
Total events	0		0			
Heterogeneity: Not applicable						
Test for overall effect: Not applicable						
**18.3.2 Medium**						
Brams et al, 2018	22	132	3	131	6.5%	7.28 [2.23, 23.73]
Childress et al, 2017	1	51	1	48	2.2%	0.94 [0.06, 14.63]
Cutler et al, 2022B	6	105	5	105	10.8%	1.20 [0.38, 3.81]
McCracken et al, 2003	17	48	10	49	21.4%	1.74 [0.89, 3.40]
McCracken et al, 2003 - XR	14	49	10	49	21.6%	1.40 [0.69, 2.84]
Ramtvedt et al, 2014 - AMPH	30	34	14	34	30.3%	2.14 [1.41, 3.26]
Spencer et al, 2006	28	226	2	52	7.0%	3.22 [0.79, 13.10]
**Subtotal (95% CI)**		**645**		**468**	**100.0%**	**2.18 [1.61, 2.95]**
Total events	118		45			
Heterogeneity: $Chi^2 = 7.62$, df = 6 (P = 0.27); $I^2 = 21\%$						
Test for overall effect: Z = 5.02 (P < 0.00001)						
**18.3.3 High**						
**Subtotal (95% CI)**		**0**		**0**		**Not estimable**
Total events	0		0			
Heterogeneity: Not applicable						
Test for overall effect: Not applicable						
**Total (95% CI)**		**645**		**468**	**100.0%**	**2.18 [1.61, 2.95]**
Total events	118		45			
Heterogeneity: $Chi^2 = 7.62$, df = 6 (P = 0.27); $I^2 = 21\%$						
Test for overall effect: Z = 5.02 (P < 0.00001)						
Test for subgroup differences: Not applicable						

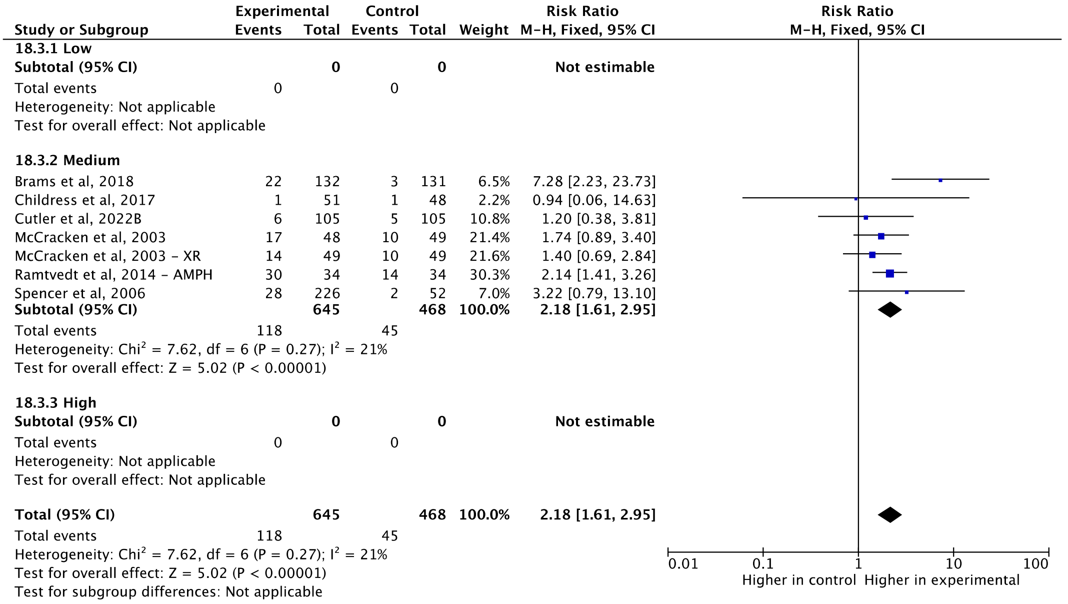

Figure legend: 95%CI, 95% confidence interval; AMPH, amphetamines; M-H, Mantel–Haenszel; XR, extended release. No study encompassing low or high dosages was included in the meta-analysis.

eFigure 85. Forest plot showing the risk ratio of **irritability** between control and experimental groups in **children** using **amphetamines** subdivided by stimulant dosage.

Study or Subgroup	Experimental Events	Experimental Total	Control Events	Control Total	Weight	Risk Ratio M-H, Fixed, 95% CI
**18.4.1 Low**						
**Subtotal (95% CI)**		**0**		**0**		**Not estimable**
Total events	0		0			
Heterogeneity: Not applicable						
Test for overall effect: Not applicable						
**18.4.2 Medium**						
Brams et al, 2018	9	132	2	131	2.3%	4.47 [0.98, 20.28]
Cutler et al, 2022B	2	105	1	105	1.1%	2.00 [0.18, 21.72]
McCracken et al, 2003	22	48	29	49	32.7%	0.77 [0.53, 1.14]
McCracken et al, 2003 - XR	21	49	29	49	33.1%	0.72 [0.49, 1.08]
Ramtvedt et al, 2014 - AMPH	23	34	27	34	30.8%	0.85 [0.64, 1.14]
**Subtotal (95% CI)**		**368**		**368**	**100.0%**	**0.88 [0.71, 1.08]**
Total events	77		88			
Heterogeneity: $Chi^2 = 6.28$, df = 4 (P = 0.18); $I^2 = 36\%$						
Test for overall effect: Z = 1.20 (P = 0.23)						
**18.4.3 High**						
**Subtotal (95% CI)**		**0**		**0**		**Not estimable**
Total events	0		0			
Heterogeneity: Not applicable						
Test for overall effect: Not applicable						
**Total (95% CI)**		**368**		**368**	**100.0%**	**0.88 [0.71, 1.08]**
Total events	77		88			
Heterogeneity: $Chi^2 = 6.28$, df = 4 (P = 0.18); $I^2 = 36\%$						
Test for overall effect: Z = 1.20 (P = 0.23)						
Test for subgroup differences: Not applicable						

Risk Ratio M-H, Fixed, 95% CI

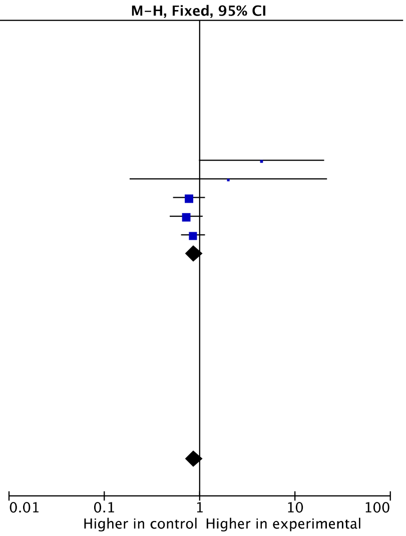

Figure legend: 95%CI, 95% confidence interval; AMPH, amphetamines; M-H, Mantel–Haenszel; XR, extended release. No study encompassing low or high dosages was included in the meta-analysis.

eFigure 86. Forest plot showing the risk ratio of **nausea** between control and experimental groups in **children** using **amphetamines** subdivided by stimulant dosage.

Study or Subgroup	Experimental Events	Experimental Total	Control Events	Control Total	Weight	Risk Ratio M-H, Fixed, 95% CI
**18.11.1 Low**						
**Subtotal (95% CI)**		**0**		**0**		**Not estimable**
Total events	0		0			
Heterogeneity: Not applicable						
Test for overall effect: Not applicable						
**18.11.2 Medium**						
Brams et al, 2018	9	132	4	131	36.5%	2.23 [0.71, 7.07]
Cutler et al, 2022B	3	105	1	105	9.1%	3.00 [0.32, 28.38]
Ramtvedt et al, 2014 - AMPH	10	34	6	34	54.5%	1.67 [0.68, 4.07]
**Subtotal (95% CI)**		**271**		**270**	**100.0%**	**1.99 [1.01, 3.92]**
Total events	22		11			
Heterogeneity: Chi^2 = 0.32, df = 2 (P = 0.85); I^2 = 0%						
Test for overall effect: Z = 2.00 (P = 0.05)						
**18.11.3 High**						
**Subtotal (95% CI)**		**0**		**0**		**Not estimable**
Total events	0		0			
Heterogeneity: Not applicable						
Test for overall effect: Not applicable						
**Total (95% CI)**		**271**		**270**	**100.0%**	**1.99 [1.01, 3.92]**
Total events	22		11			
Heterogeneity: Chi^2 = 0.32, df = 2 (P = 0.85); I^2 = 0%						
Test for overall effect: Z = 2.00 (P = 0.05)						
Test for subgroup differences: Not applicable						

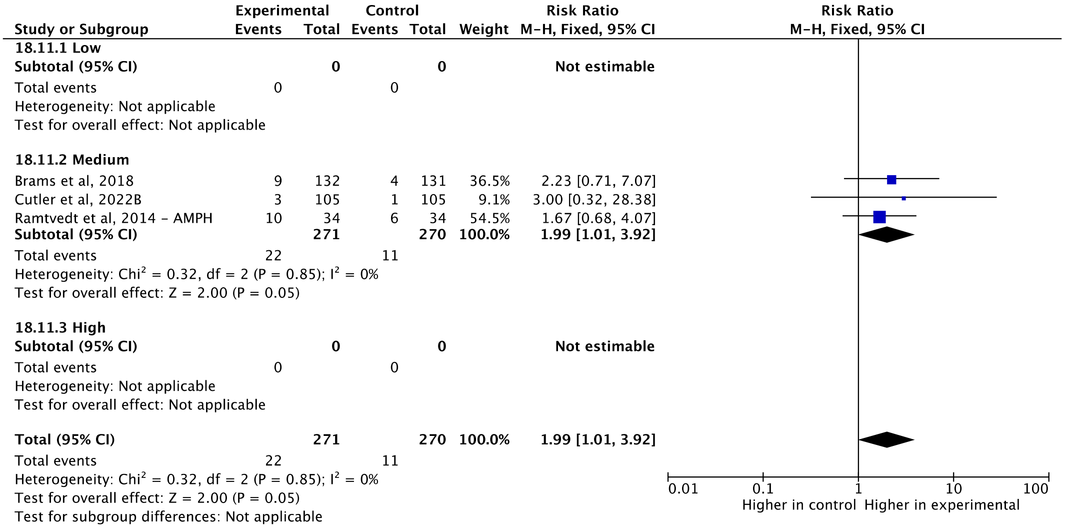

Figure legend: 95%CI, 95% confidence interval; AMPH, amphetamines; M-H, Mantel–Haenszel. No study encompassing low or high dosages was included in the meta-analysis.

eFigure 87. Forest plot showing the mean differences in **heart rate** between control and experimental groups using **amphetamines** in **children** subdivided by stimulant dosage.

	Experimental			Control				Mean Difference
**Study or Subgroup**	**Mean**	**SD**	**Total**	**Mean**	**SD**	**Total**	**Weight**	**IV, Fixed, 95% CI**
**18.9.1 Low**								
Mattingly et al, 2020	−0.5	9.87	45	−1.8	10.02	43	21.5%	1.30 [−2.86, 5.46]
**Subtotal (95% CI)**			**45**			**43**	**21.5%**	**1.30 [−2.86, 5.46]**
Heterogeneity: Not applicable								
Test for overall effect: Z = 0.61 (P = 0.54)								
**18.9.2 Medium**								
Brams et al, 2008	5.7	11.78	132	0.7	10.79	131	49.8%	5.00 [2.27, 7.73]
Cutler et al, 2022B	13.4	13.7	105	12.8	12.9	105	28.7%	0.60 [−3.00, 4.20]
**Subtotal (95% CI)**			**237**			**236**	**78.5%**	**3.39 [1.22, 5.57]**
Heterogeneity: $Chi^2 = 3.64$, df = 1 (P = 0.06); $I^2 = 73\%$								
Test for overall effect: Z = 3.06 (P = 0.002)								
**18.9.3 High**								
**Subtotal (95% CI)**			**0**			**0**		**Not estimable**
Heterogeneity: Not applicable								
Test for overall effect: Not applicable								
**Total (95% CI)**			**282**			**279**	**100.0%**	**2.94 [1.02, 4.87]**
Heterogeneity: $Chi^2 = 4.41$, df = 2 (P = 0.11); $I^2 = 55\%$								
Test for overall effect: Z = 2.99 (P = 0.003)								
Test for subgroup differences: $Chi^2 = 0.76$, df = 1 (P = 0.38), $I^2 = 0\%$								

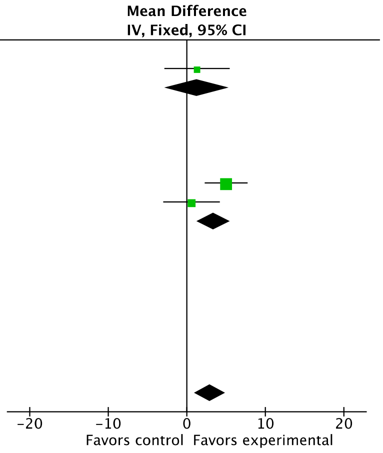

Figure legend: 95%CI, 95% confidence interval; IV, inverse variance; SD, standard deviation. No study encompassing high dosage was included in the meta-analysis.

eFigure 88. Forest plot showing the mean differences in **diastolic blood pressure** between control and experimental groups using **amphetamines** in **children** subdivided by stimulant dosage.

Study or Subgroup	Experimental Mean	Experimental SD	Experimental Total	Control Mean	Control SD	Control Total	Weight	Mean Difference IV, Fixed, 95% CI
**18.8.1 Low**								
Mattingly et al, 2020	3.1	7.24	45	0.3	6.61	43	22.2%	2.80 [−0.09, 5.69]
**Subtotal (95% CI)**			**45**			**43**	**22.2%**	**2.80 [−0.09, 5.69]**
Heterogeneity: Not applicable								
Test for overall effect: Z = 1.90 (P = 0.06)								
**18.8.2 Medium**								
Brams et al, 2008	4	8.23	132	0.5	7.45	131	51.8%	3.50 [1.60, 5.40]
Cutler et al, 2022B	3.4	9.9	105	1.8	9.9	105	26.0%	1.60 [−1.08, 4.28]
**Subtotal (95% CI)**			**237**			**236**	**77.8%**	**2.87 [1.32, 4.41]**
Heterogeneity: Chi^2 = 1.29, df = 1 (P = 0.26); I^2 = 22%								
Test for overall effect: Z = 3.63 (P = 0.0003)								
**18.8.3 High**								
**Subtotal (95% CI)**			**0**			**0**		**Not estimable**
Heterogeneity: Not applicable								
Test for overall effect: Not applicable								
**Total (95% CI)**			**282**			**279**	**100.0%**	**2.85 [1.49, 4.22]**
Heterogeneity: Chi^2 = 1.29, df = 2 (P = 0.52); I^2 = 0%								
Test for overall effect: Z = 4.09 (P < 0.0001)								
Test for subgroup differences: Chi^2 = 0.00, df = 1 (P = 0.97), I^2 = 0%								

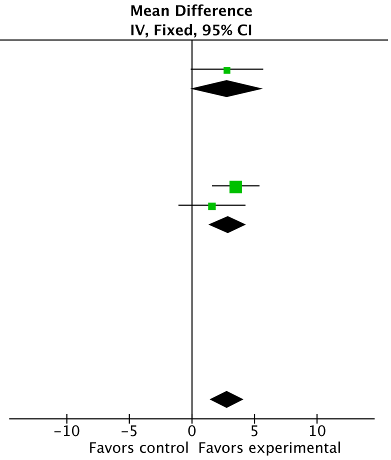

Figure legend: 95%CI, 95% confidence interval; IV, inverse variance; SD, standard deviation. No study encompassing high dosage was included in the meta-analysis.

eFigure 89. Forest plot showing the mean differences in **systolic blood pressure** between control and experimental groups using **amphetamines** in **children** subdivided by stimulant dosage.

	Experimental			Control				Mean Difference
**Study or Subgroup**	**Mean**	**SD**	**Total**	**Mean**	**SD**	**Total**	**Weight**	**IV, Fixed, 95% CI**
**18.7.1 Low**								
Mattingly et al, 2020	1.8	6.52	45	−0.8	6.23	43	30.0%	2.60 [−0.06, 5.26]
**Subtotal (95% CI)**			**45**			**43**	**30.0%**	**2.60 [−0.06, 5.26]**
Heterogeneity: Not applicable								
Test for overall effect: Z = 1.91 (P = 0.06)								
**18.7.2 Medium**								
Brams et al, 2008	3.8	9.15	132	2.1	8.72	131	45.6%	1.70 [−0.46, 3.86]
Cutler et al, 2022B	3.3	11	105	3.1	10.8	105	24.5%	0.20 [−2.75, 3.15]
**Subtotal (95% CI)**			**237**			**236**	**70.0%**	**1.18 [−0.57, 2.92]**
Heterogeneity: Chi^2 = 0.65, df = 1 (P = 0.42); I^2 = 0%								
Test for overall effect: Z = 1.32 (P = 0.19)								
**18.7.3 High**								
**Subtotal (95% CI)**			**0**			**0**		**Not estimable**
Heterogeneity: Not applicable								
Test for overall effect: Not applicable								
**Total (95% CI)**			**282**			**279**	**100.0%**	**1.60 [0.14, 3.06]**
Heterogeneity: Chi^2 = 1.42, df = 2 (P = 0.49); I^2 = 0%								
Test for overall effect: Z = 2.15 (P = 0.03)								
Test for subgroup differences: Chi^2 = 0.77, df = 1 (P = 0.38), I^2 = 0%								

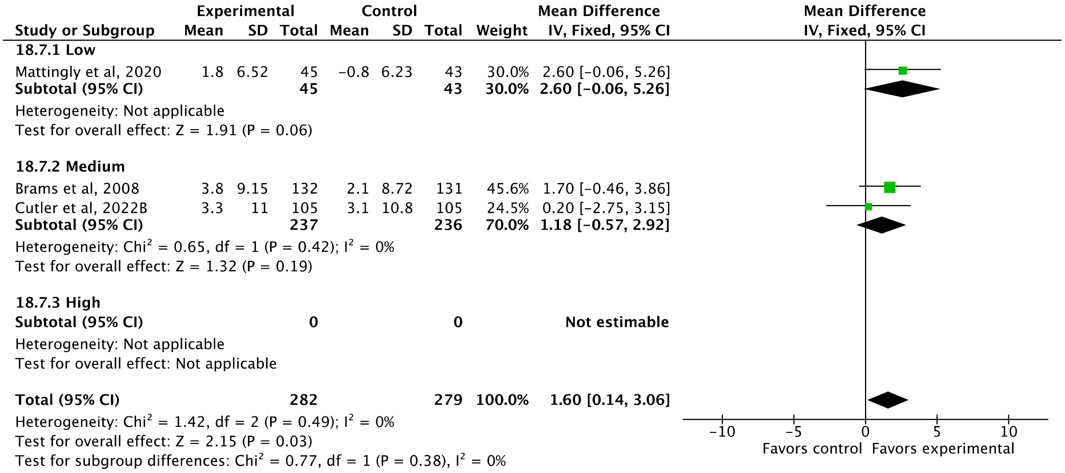

Figure legend: 95%CI, 95% confidence interval; IV, inverse variance; SD, standard deviation. No study encompassing high dosage was included in the meta-analysis.

eFigure 90. Forest plot showing the risk ratio of **all adverse events** between control and experimental groups in **adults** using **amphetamines** subdivided by stimulant dosage.

	Experimental		Control			Risk Ratio
**Study or Subgroup**	**Events**	**Total**	**Events**	**Total**	**Weight**	**M-H, Random, 95% CI**
**15.10.1 Low**						
**Subtotal (95% CI)**		**0**		**0**		**Not estimable**
Total events	0		0			
Heterogeneity: Not applicable						
Test for overall effect: Not applicable						
**15.10.2 Medium**						
Cutler et al, 2022A	54	62	35	65	25.5%	1.62 [1.27, 2.07]
Martin et al, 2014B - AMPH	9	17	8	17	9.3%	1.13 [0.57, 2.21]
Nujiten et al, 2016	28	38	16	35	17.3%	1.61 [1.07, 2.42]
Spencer et al, 2008	122	137	86	135	31.1%	1.40 [1.22, 1.61]
Weisler et al, 2017	104	182	19	89	16.9%	2.68 [1.76, 4.07]
**Subtotal (95% CI)**		**436**		**341**	**100.0%**	**1.63 [1.28, 2.07]**
Total events	317		164			
Heterogeneity: $Tau^2 = 0.04$; $Chi^2 = 11.50$, df = 4 (P = 0.02); $I^2 = 65\%$						
Test for overall effect: Z = 3.98 (P < 0.0001)						
**15.10.3 High**						
**Subtotal (95% CI)**		**0**		**0**		**Not estimable**
Total events	0		0			
Heterogeneity: Not applicable						
Test for overall effect: Not applicable						
**Total (95% CI)**		**436**		**341**	**100.0%**	**1.63 [1.28, 2.07]**
Total events	317		164			
Heterogeneity: $Tau^2 = 0.04$; $Chi^2 = 11.50$, df = 4 (P = 0.02); $I^2 = 65\%$						
Test for overall effect: Z = 3.98 (P < 0.0001)						
Test for subgroup differences: Not applicable						

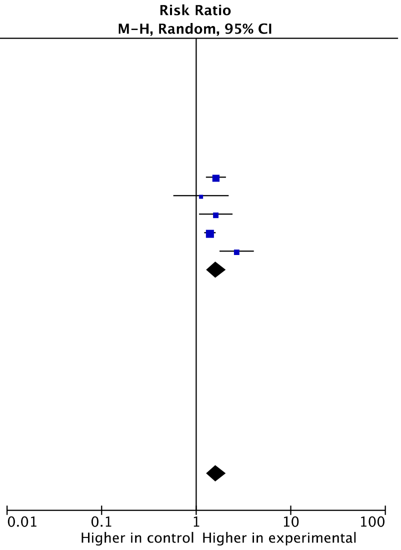

Figure legend: 95%CI, 95% confidence interval; AMPH, amphetamines; M-H, Mantel–Haenszel. No study encompassing low or high dosages was included in the meta-analysis.

eFigure 91. Forest plot showing the risk ratio of **anxiety** between control and experimental groups in **adults** using **amphetamines** subdivided by stimulant dosage.

Study or Subgroup	Experimental Events	Experimental Total	Control Events	Control Total	Weight	Risk Ratio M-H, Fixed, 95% CI
**15.6.1 Low**						
**Subtotal (95% CI)**		**0**		**0**		**Not estimable**
Total events	0		0			
Heterogeneity: Not applicable						
Test for overall effect: Not applicable						
**15.6.2 Medium**						
Spencer et al, 2008	9	137	4	135	75.0%	2.22 [0.70, 7.03]
Weisler et al, 2017	10	182	1	89	25.0%	4.89 [0.64, 37.61]
**Subtotal (95% CI)**		**319**		**224**	**100.0%**	**2.89 [1.05, 7.91]**
Total events	19		5			
Heterogeneity: $Chi^2 = 0.46$, df = 1 (P = 0.50); $I^2 = 0\%$						
Test for overall effect: Z = 2.06 (P = 0.04)						
**15.6.3 High**						
**Subtotal (95% CI)**		**0**		**0**		**Not estimable**
Total events	0		0			
Heterogeneity: Not applicable						
Test for overall effect: Not applicable						
**Total (95% CI)**		**319**		**224**	**100.0%**	**2.89 [1.05, 7.91]**
Total events	19		5			
Heterogeneity: $Chi^2 = 0.46$, df = 1 (P = 0.50); $I^2 = 0\%$						
Test for overall effect: Z = 2.06 (P = 0.04)						
Test for subgroup differences: Not applicable						

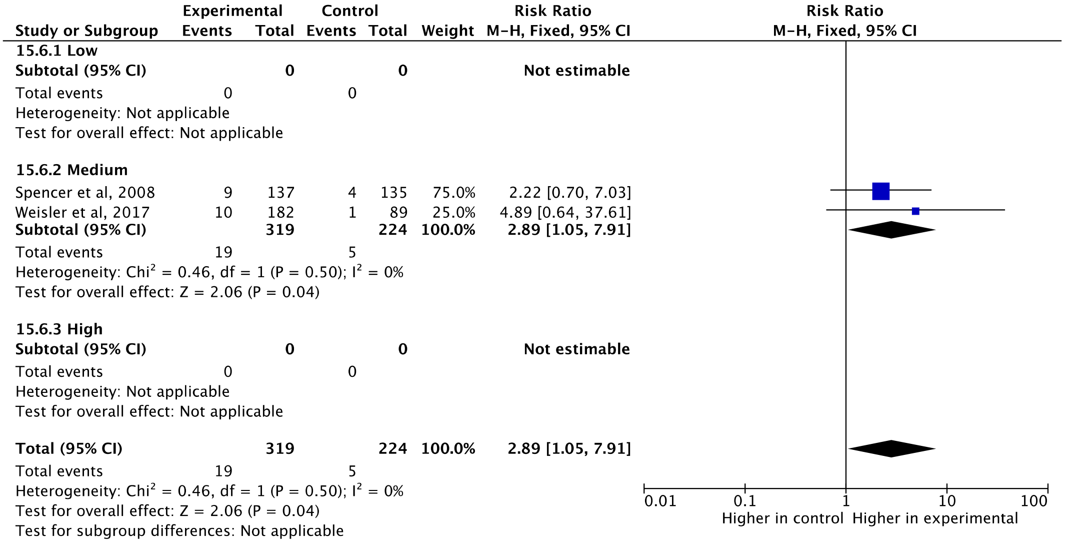

Figure legend: 95%CI, 95% confidence interval; M-H, Mantel–Haenszel. No study encompassing low or high dosages was included in the meta-analysis.

eFigure 92. Forest plot showing the risk ratio of **decreased appetite** between control and experimental groups in **adults** using **amphetamines** subdivided by stimulant dosage.

Study or Subgroup	Experimental Events	Experimental Total	Control Events	Control Total	Weight	Risk Ratio M-H, Fixed, 95% CI
**15.1.1 Low**						
**Subtotal (95% CI)**		**0**		**0**		**Not estimable**
Total events	0		0			
Heterogeneity: Not applicable						
Test for overall effect: Not applicable						
**15.1.2 Medium**						
Cutler et al, 2022A	30	62	14	65	44.4%	2.25 [1.32, 3.82]
Faraone et al, 2021	25	214	5	107	21.6%	2.50 [0.98, 6.35]
Martin et al, 2014B - AMPH	4	17	1	17	3.2%	4.00 [0.50, 32.20]
Nujiten et al, 2016	6	38	2	35	6.8%	2.76 [0.60, 12.80]
Spencer et al, 2008	27	137	2	135	6.5%	13.30 [3.23, 54.84]
Weisler et al, 2017	45	182	4	89	17.4%	5.50 [2.04, 14.82]
**Subtotal (95% CI)**		**650**		**448**	**100.0%**	**3.68 [2.50, 5.43]**
Total events	137		28			
Heterogeneity: Chi² = 7.93, df = 5 (P = 0.16); I² = 37%						
Test for overall effect: Z = 6.60 (P < 0.00001)						
**15.1.3 High**						
**Subtotal (95% CI)**		**0**		**0**		**Not estimable**
Total events	0		0			
Heterogeneity: Not applicable						
Test for overall effect: Not applicable						
**Total (95% CI)**		**650**		**448**	**100.0%**	**3.68 [2.50, 5.43]**
Total events	137		28			
Heterogeneity: Chi² = 7.93, df = 5 (P = 0.16); I² = 37%						
Test for overall effect: Z = 6.60 (P < 0.00001)						
Test for subgroup differences: Not applicable						

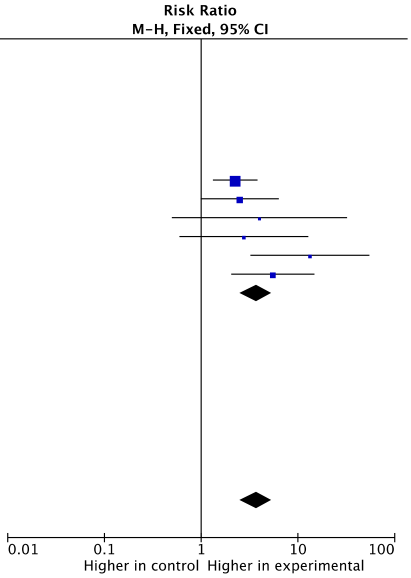

Figure legend: 95%CI, 95% confidence interval; AMPH, amphetamines; M-H, Mantel–Haenszel. No study encompassing low or high dosages was included in the meta-analysis.

eFigure 93. Forest plot showing the risk ratio of **dry mouth** between control and experimental groups in **adults** using **amphetamines** subdivided by stimulant dosage.

Study or Subgroup	Experimental Events	Experimental Total	Control Events	Control Total	Weight	Risk Ratio M-H, Fixed, 95% CI
**15.5.1 Low**						
**Subtotal (95% CI)**		**0**		**0**		**Not estimable**
Total events	0		0			
Heterogeneity: Not applicable						
Test for overall effect: Not applicable						
**15.5.2 Medium**						
Cutler et al, 2022A	12	62	1	65	6.5%	12.58 [1.69, 93.90]
Martin et al, 2014B - AMPH	4	17	3	17	19.9%	1.33 [0.35, 5.08]
Spencer et al, 2008	31	137	7	135	46.8%	4.36 [1.99, 9.57]
Weisler et al, 2017	33	182	3	89	26.8%	5.38 [1.70, 17.06]
**Subtotal (95% CI)**		**398**		**306**	**100.0%**	**4.56 [2.62, 7.96]**
Total events	80		14			
Heterogeneity: $Chi^2 = 4.32$, df = 3 (P = 0.23); $I^2 = 31\%$						
Test for overall effect: Z = 5.35 (P < 0.00001)						
**15.5.3 High**						
**Subtotal (95% CI)**		**0**		**0**		**Not estimable**
Total events	0		0			
Heterogeneity: Not applicable						
Test for overall effect: Not applicable						
**Total (95% CI)**		**398**		**306**	**100.0%**	**4.56 [2.62, 7.96]**
Total events	80		14			
Heterogeneity: $Chi^2 = 4.32$, df = 3 (P = 0.23); $I^2 = 31\%$						
Test for overall effect: Z = 5.35 (P < 0.00001)						
Test for subgroup differences: Not applicable						

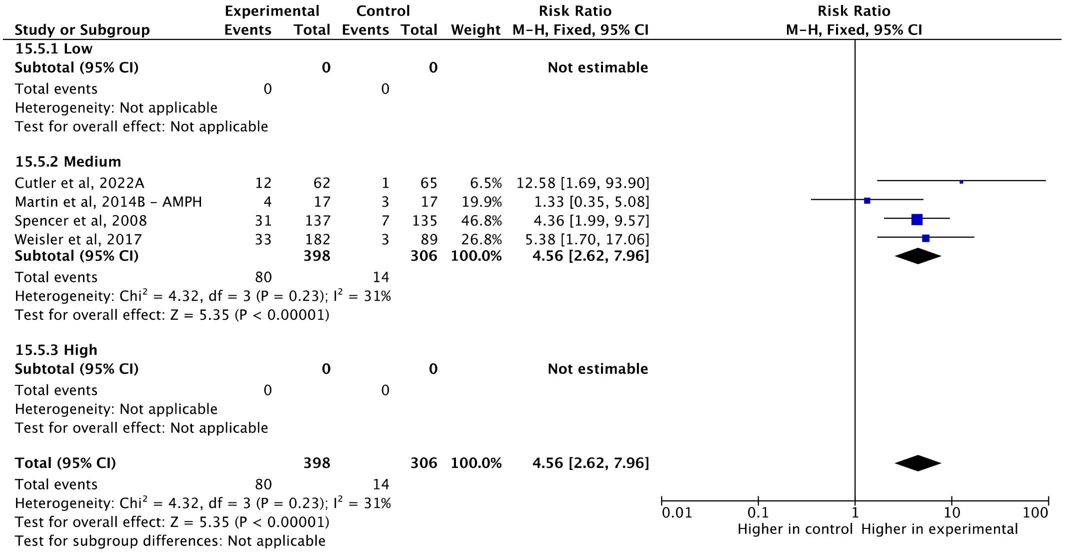

Figure legend: 95%CI, 95% confidence interval; AMPH, amphetamines; M-H, Mantel–Haenszel. No study encompassing low or high dosages was included in the meta-analysis.

eFigure 94. Forest plot showing the risk ratio of **headache** between control and experimental groups in **adults** using **amphetamines** subdivided by stimulant dosage.

Study or Subgroup	Experimental Events	Experimental Total	Control Events	Control Total	Weight	Risk Ratio M-H, Fixed, 95% CI
**15.2.1 Low**						
**Subtotal (95% CI)**		**0**		**0**		**Not estimable**
Total events	0		0			
Heterogeneity: Not applicable						
Test for overall effect: Not applicable						
**15.2.2 Medium**						
Martin et al, 2014B - AMPH	1	17	3	17	10.9%	0.33 [0.04, 2.89]
Spencer et al, 2008	25	137	19	135	69.6%	1.30 [0.75, 2.24]
Weisler et al, 2017	17	182	4	89	19.5%	2.08 [0.72, 5.99]
**Subtotal (95% CI)**		**336**		**241**	**100.0%**	**1.34 [0.84, 2.15]**
Total events	43		26			
Heterogeneity: $Chi^2 = 2.27$, df = 2 (P = 0.32); $I^2 = 12\%$						
Test for overall effect: Z = 1.24 (P = 0.22)						
**15.2.3 High**						
**Subtotal (95% CI)**		**0**		**0**		**Not estimable**
Total events	0		0			
Heterogeneity: Not applicable						
Test for overall effect: Not applicable						
**Total (95% CI)**		**336**		**241**	**100.0%**	**1.34 [0.84, 2.15]**
Total events	43		26			
Heterogeneity: $Chi^2 = 2.27$, df = 2 (P = 0.32); $I^2 = 12\%$						
Test for overall effect: Z = 1.24 (P = 0.22)						
Test for subgroup differences: Not applicable						

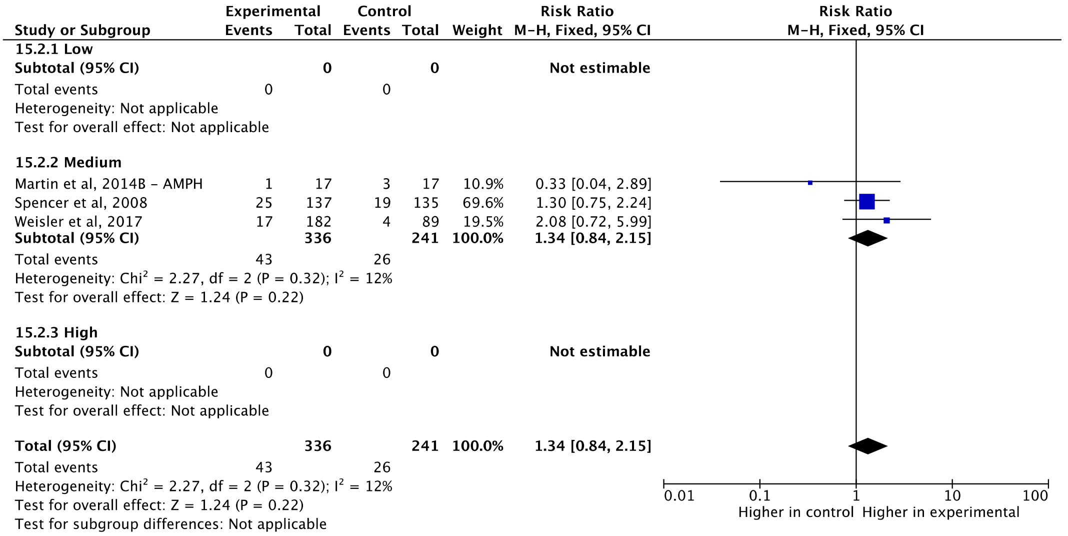

Figure legend: 95%CI, 95% confidence interval; AMPH, amphetamines; M-H, Mantel–Haenszel. No study encompassing low or high dosages was included in the meta-analysis.

eFigure 95. Forest plot showing the risk ratio of **insomnia** between control and experimental groups in **adults** using **amphetamines** subdivided by stimulant dosage.

Study or Subgroup	Experimental Events	Experimental Total	Control Events	Control Total	Weight	Risk Ratio M-H, Fixed, 95% CI
**15.3.1 Low**						
**Subtotal (95% CI)**		**0**		**0**		**Not estimable**
Total events	0		0			
Heterogeneity: Not applicable						
Test for overall effect: Not applicable						
**15.3.2 Medium**						
Cutler et al, 2022A	14	62	8	65	36.8%	1.83 [0.83, 4.07]
Spencer et al, 2008	40	137	12	135	56.9%	3.28 [1.80, 5.98]
Weisler et al, 2017	22	182	1	89	6.3%	10.76 [1.47, 78.54]
**Subtotal (95% CI)**		**381**		**289**	**100.0%**	**3.22 [2.02, 5.15]**
Total events	76		21			
Heterogeneity: $Chi^2 = 3.34$, df = 2 (P = 0.19); $I^2 = 40\%$						
Test for overall effect: Z = 4.89 (P < 0.00001)						
**15.3.3 High**						
**Subtotal (95% CI)**		**0**		**0**		**Not estimable**
Total events	0		0			
Heterogeneity: Not applicable						
Test for overall effect: Not applicable						
**Total (95% CI)**		**381**		**289**	**100.0%**	**3.22 [2.02, 5.15]**
Total events	76		21			
Heterogeneity: $Chi^2 = 3.34$, df = 2 (P = 0.19); $I^2 = 40\%$						
Test for overall effect: Z = 4.89 (P < 0.00001)						
Test for subgroup differences: Not applicable						

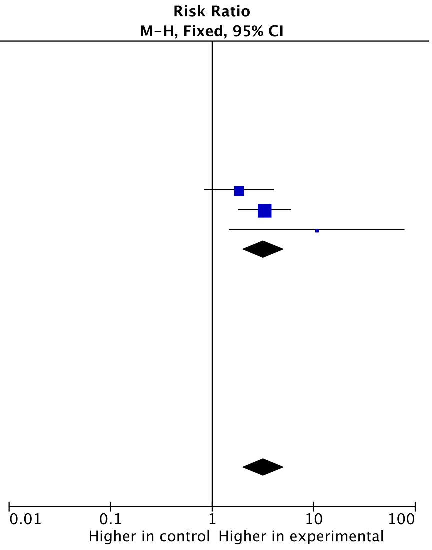

Figure legend: 95%CI, 95% confidence interval; M-H, Mantel–Haenszel. No study encompassing low or high dosages was included in the meta-analysis.

eFigure 96. Forest plot showing the risk ratio of **irritability** between control and experimental groups in **adults** using **amphetamines** subdivided by stimulant dosage.

Study or Subgroup	Experimental Events	Experimental Total	Control Events	Control Total	Weight	Risk Ratio M-H, Fixed, 95% CI
**15.4.1 Low**						
**Subtotal (95% CI)**		**0**		**0**		**Not estimable**
Total events	0		0			
Heterogeneity: Not applicable						
Test for overall effect: Not applicable						
**15.4.2 Medium**						
Cutler et al, 2022A	11	62	5	65	49.2%	2.31 [0.85, 6.26]
Spencer et al, 2008	13	137	5	135	50.8%	2.56 [0.94, 6.99]
**Subtotal (95% CI)**		**199**		**200**	**100.0%**	**2.44 [1.20, 4.95]**
Total events	24		10			
Heterogeneity: $Chi^2 = 0.02$, df = 1 (P = 0.88); $I^2 = 0\%$						
Test for overall effect: Z = 2.46 (P = 0.01)						
**15.4.3 High**						
**Subtotal (95% CI)**		**0**		**0**		**Not estimable**
Total events	0		0			
Heterogeneity: Not applicable						
Test for overall effect: Not applicable						
**Total (95% CI)**		**199**		**200**	**100.0%**	**2.44 [1.20, 4.95]**
Total events	24		10			
Heterogeneity: $Chi^2 = 0.02$, df = 1 (P = 0.88); $I^2 = 0\%$						
Test for overall effect: Z = 2.46 (P = 0.01)						
Test for subgroup differences: Not applicable						

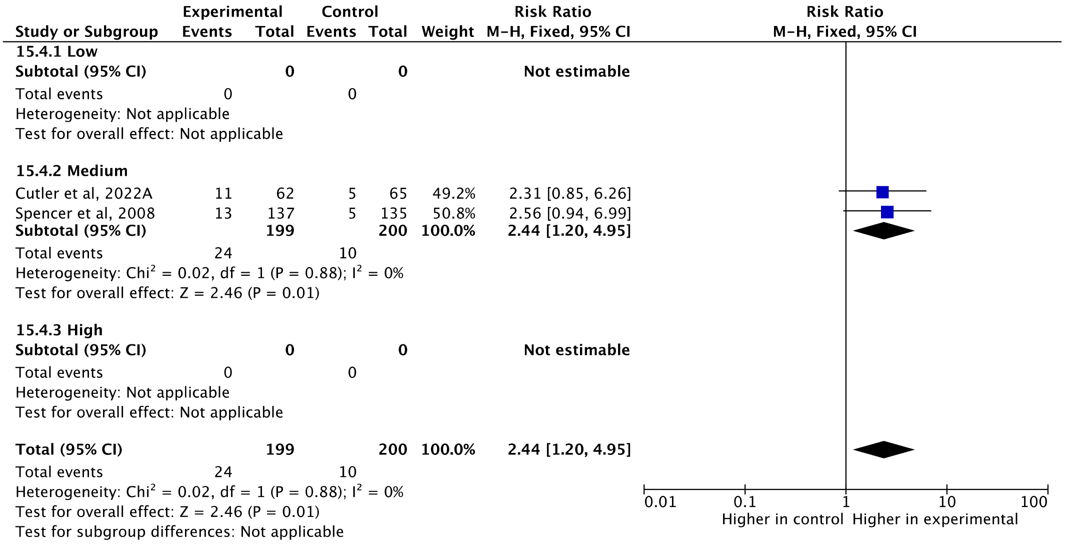

Figure legend: 95%CI, 95% confidence interval; M-H, Mantel–Haenszel. No study encompassing low or high dosages was included in the meta-analysis.

eFigure 97. Forest plot showing the mean differences in **heart rate** between control and experimental groups using **amphetamines** in **adults** subdivided by stimulant dosage.

	Experimental			Control				Mean Difference
**Study or Subgroup**	**Mean**	**SD**	**Total**	**Mean**	**SD**	**Total**	**Weight**	**IV, Fixed, 95% CI**
**15.9.1 Low**								
**Subtotal (95% CI)**			**0**			**0**		**Not estimable**
Heterogeneity: Not applicable								
Test for overall effect: Not applicable								
**15.9.2 Medium**								
Spencer et al, 2008	4.7	11.1	136	0.4	10.5	133	44.5%	4.30 [1.72, 6.88]
Weisler et al, 2017	3.3	10.52	182	0.1	8.35	89	55.5%	3.20 [0.89, 5.51]
**Subtotal (95% CI)**			**318**			**222**	**100.0%**	**3.69 [1.97, 5.41]**
Heterogeneity: Chi² = 0.39, df = 1 (P = 0.53); I² = 0%								
Test for overall effect: Z = 4.20 (P < 0.0001)								
**15.9.3 High**								
**Subtotal (95% CI)**			**0**			**0**		**Not estimable**
Heterogeneity: Not applicable								
Test for overall effect: Not applicable								
**Total (95% CI)**			**318**			**222**	**100.0%**	**3.69 [1.97, 5.41]**
Heterogeneity: Chi² = 0.39, df = 1 (P = 0.53); I² = 0%								
Test for overall effect: Z = 4.20 (P < 0.0001)								
Test for subgroup differences: Not applicable								

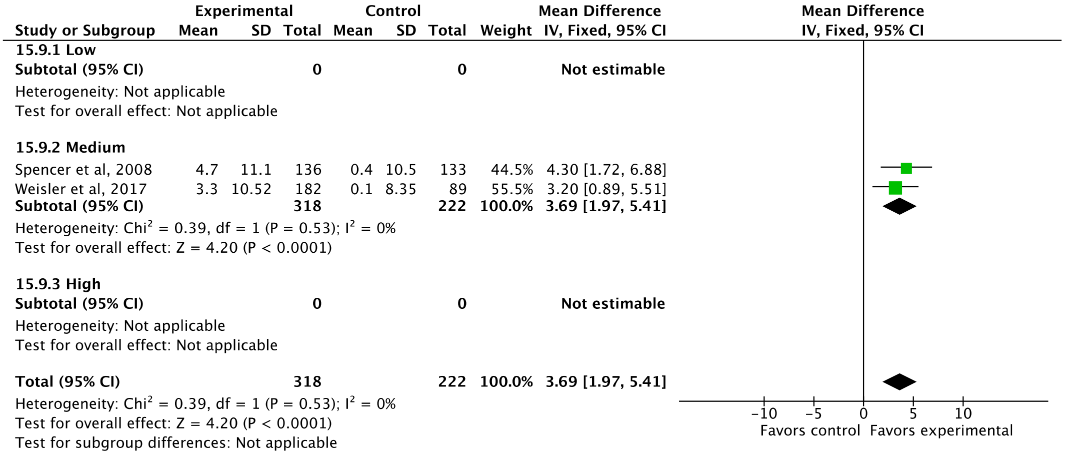

Figure legend: 95%CI, 95% confidence interval; IV, inverse variance; SD, standard deviation. No study encompassing low or high dosages was included in the meta-analysis.

eFigure 98. Forest plot showing the mean differences in **diastolic blood pressure** between control and experimental groups using **amphetamines** in **adults** subdivided by stimulant dosage.

	Experimental			Control				Mean Difference
**Study or Subgroup**	**Mean**	**SD**	**Total**	**Mean**	**SD**	**Total**	**Weight**	**IV, Fixed, 95% CI**
**15.8.1 Low**								
**Subtotal (95% CI)**			**0**			**0**		**Not estimable**
Heterogeneity: Not applicable								
Test for overall effect: Not applicable								
**15.8.2 Medium**								
Spencer et al, 2008	1.8	6.7	136	1.1	6.4	133	60.5%	0.70 [−0.87, 2.27]
Weisler et al, 2017	1	7.46	182	0.1	7.72	89	39.5%	0.90 [−1.04, 2.84]
**Subtotal (95% CI)**			**318**			**222**	**100.0%**	**0.78 [−0.44, 2.00]**
Heterogeneity: $Chi^2 = 0.02$, df = 1 (P = 0.87); $I^2 = 0\%$								
Test for overall effect: Z = 1.25 (P = 0.21)								
**15.8.3 High**								
**Subtotal (95% CI)**			**0**			**0**		**Not estimable**
Heterogeneity: Not applicable								
Test for overall effect: Not applicable								
**Total (95% CI)**			**318**			**222**	**100.0%**	**0.78 [−0.44, 2.00]**
Heterogeneity: $Chi^2 = 0.02$, df = 1 (P = 0.87); $I^2 = 0\%$								
Test for overall effect: Z = 1.25 (P = 0.21)								
Test for subgroup differences: Not applicable								

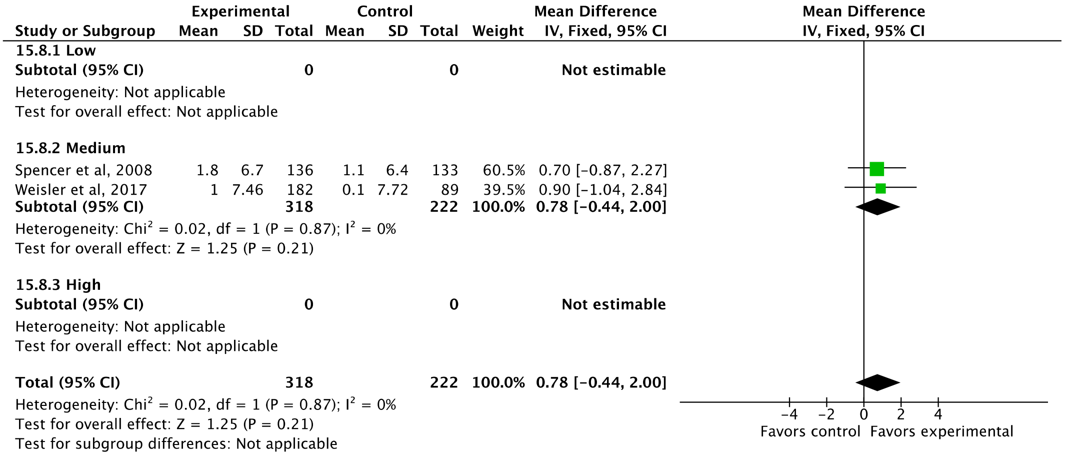

Figure legend: 95%CI, 95% confidence interval; IV, inverse variance; SD, standard deviation. No study encompassing low or high dosages was included in the meta-analysis.

eFigure 99. Forest plot showing the mean differences in **systolic blood pressure** between control and experimental groups using **amphetamines** in **adults** subdivided by stimulant dosage.

Study or Subgroup	Experimental Mean	Experimental SD	Experimental Total	Control Mean	Control SD	Control Total	Weight	Mean Difference IV, Fixed, 95% CI
**15.7.1 Low**								
**Subtotal (95% CI)**			**0**			**0**		**Not estimable**
Heterogeneity: Not applicable								
Test for overall effect: Not applicable								
**15.7.2 Medium**								
Spencer et al, 2008	1.3	9.3	136	0.2	9.4	133	52.0%	1.10 [−1.13, 3.33]
Weisler et al, 2017	0.2	7.24	182	−0.8	9.99	89	48.0%	1.00 [−1.33, 3.33]
**Subtotal (95% CI)**			**318**			**222**	**100.0%**	**1.05 [−0.56, 2.66]**
Heterogeneity: $Chi^2 = 0.00$, df = 1 (P = 0.95); $I^2 = 0\%$								
Test for overall effect: Z = 1.28 (P = 0.20)								
**15.7.3 High**								
**Subtotal (95% CI)**			**0**			**0**		**Not estimable**
Heterogeneity: Not applicable								
Test for overall effect: Not applicable								
**Total (95% CI)**			**318**			**222**	**100.0%**	**1.05 [−0.56, 2.66]**
Heterogeneity: $Chi^2 = 0.00$, df = 1 (P = 0.95); $I^2 = 0\%$								
Test for overall effect: Z = 1.28 (P = 0.20)								
Test for subgroup differences: Not applicable								

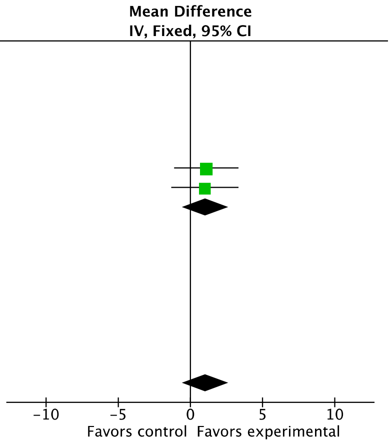

Figure legend: 95%CI, 95% confidence interval; IV, inverse variance; SD, standard deviation. No study encompassing low or high dosages was included in the meta-analysis.

eFigures 100-144. Forest plots dividing groups according to stimulant dosage and duration of use.

eFigure 100. Forest plot showing the risk ratio of **all adverse events** between control and experimental groups in participants using **medium** dose **methylphenidate** subdivided by duration of use.

Study or Subgroup	Experimental Events	Experimental Total	Control Events	Control Total	Weight	Risk Ratio M-H, Random, 95% CI
**15.8.1 Short duration**						
Brams et al, 2008	15	86	19	86	2.2%	0.79 [0.43, 1.45]
Childress et al, 2020C	10	39	6	50	1.1%	2.14 [0.85, 5.37]
Huang et al, 2021	79	110	10	101	2.3%	7.25 [3.98, 13.21]
Muniz et al, 2008	40	332	0	83	0.1%	20.43 [1.27, 328.88]
Patkar et al, 2006	19	30	17	30	3.6%	1.12 [0.74, 1.69]
Wilens et al, 2006	15	87	14	90	1.9%	1.11 [0.57, 2.16]
Wolraich et al, 2001 - IR	40	95	31	90	4.1%	1.22 [0.84, 1.77]
Wolraich et al, 2001 - OROS	45	97	31	90	4.3%	1.35 [0.94, 1.92]
**Subtotal (95% CI)**		**876**		**620**	**19.7%**	**1.67 [1.00, 2.78]**
Total events	263		128			
Heterogeneity: Tau² = 0.40; Chi² = 44.79, df = 7 (P < 0.00001); I² = 84%						
Test for overall effect: Z = 1.97 (P = 0.05)						
**15.8.2 Medium duration**						
Bron et al, 2014	17	22	10	22	2.8%	1.70 [1.02, 2.83]
Childress et al, 2009	116	182	36	63	5.8%	1.12 [0.88, 1.42]
Coghill et al, 2013 - MPH	72	111	63	110	6.2%	1.13 [0.92, 1.40]
Findling et al, 2008 - MPH	74	98	49	85	6.2%	1.31 [1.06, 1.62]
Goodman et al, 2017	126	174	87	175	6.8%	1.46 [1.22, 1.74]
Medori et al, 2008	182	305	41	96	5.7%	1.40 [1.09, 1.79]
Newcorn et al, 2008	146	219	40	74	6.0%	1.23 [0.98, 1.55]
Silva et al, 2005 - ER	5	53	2	53	0.4%	2.50 [0.51, 12.32]
Silva et al, 2005 - OROS	11	53	2	53	0.5%	5.50 [1.28, 23.63]
Spencer et al, 2007	145	165	36	53	6.5%	1.29 [1.07, 1.57]
Takahashi et al, 2014	117	143	76	141	6.8%	1.52 [1.28, 1.80]
Zheng et al, 2024	74	110	55	112	6.0%	1.37 [1.09, 1.72]
**Subtotal (95% CI)**		**1635**		**1037**	**59.7%**	**1.34 [1.24, 1.45]**
Total events	1085		497			
Heterogeneity: Tau² = 0.00; Chi² = 13.50, df = 11 (P = 0.26); I² = 19%						
Test for overall effect: Z = 7.26 (P < 0.00001)						
**15.8.3 Long duration**						
Casas et al, 2013	161	181	76	97	7.6%	1.14 [1.01, 1.28]
Rösler et al, 2009	135	183	37	66	6.0%	1.32 [1.05, 1.66]
Winhusen et al, 2011	111	151	98	152	7.1%	1.14 [0.98, 1.33]
**Subtotal (95% CI)**		**515**		**315**	**20.6%**	**1.16 [1.06, 1.26]**
Total events	407		211			
Heterogeneity: Tau² = 0.00; Chi² = 1.43, df = 2 (P = 0.49); I² = 0%						
Test for overall effect: Z = 3.40 (P = 0.0007)						
**Total (95% CI)**		**3026**		**1972**	**100.0%**	**1.34 [1.21, 1.49]**
Total events	1755		836			
Heterogeneity: Tau² = 0.03; Chi² = 68.25, df = 22 (P < 0.00001); I² = 68%						
Test for overall effect: Z = 5.45 (P < 0.00001)						
Test for subgroup differences: Chi² = 6.93, df = 2 (P = 0.03), I² = 71.1%						

Risk Ratio
M-H, Random, 95% CI

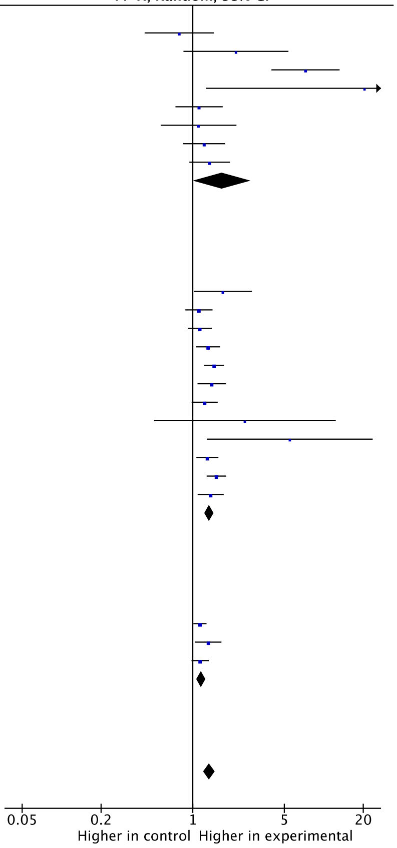

Figure legend: 95%CI, 95% confidence interval; ER, extended release; IR, immediate release; M-H, Mantel–Haenszel; MPH, methylphenidate; OROS, osmotic release oral system.

eFigure 101. Forest plot showing the risk ratio of **anxiety** between control and experimental groups in participants using **medium** dose **methylphenidate** subdivided by duration of use.

Study or Subgroup	Experimental Events	Experimental Total	Control Events	Control Total	Weight	Risk Ratio M-H, Fixed, 95% CI
**15.4.1 Short duration**						
Froehlich et al, 2020	32	171	26	171	43.7%	1.23 [0.77, 1.97]
Patkar et al, 2006	2	30	1	30	1.7%	2.00 [0.19, 20.90]
Stein et al, 2003	22	47	24	47	40.3%	0.92 [0.61, 1.39]
**Subtotal (95% CI)**		**248**		**248**	**85.7%**	**1.10 [0.80, 1.51]**
Total events	56		51			
Heterogeneity: $Chi^2 = 1.21$, df = 2 (P = 0.55); $I^2 = 0\%$						
Test for overall effect: Z = 0.58 (P = 0.56)						
**15.4.2 Medium duration**						
Medori et al, 2008	16	305	1	96	2.6%	5.04 [0.68, 37.48]
Ramtvedt et al, 2014 - MPH	10	34	7	34	11.8%	1.43 [0.62, 3.31]
**Subtotal (95% CI)**		**339**		**130**	**14.3%**	**2.07 [0.93, 4.61]**
Total events	26		8			
Heterogeneity: $Chi^2 = 1.50$, df = 1 (P = 0.22); $I^2 = 34\%$						
Test for overall effect: Z = 1.78 (P = 0.07)						
**15.4.3 Long duration**						
**Subtotal (95% CI)**		**0**		**0**		**Not estimable**
Total events	0		0			
Heterogeneity: Not applicable						
Test for overall effect: Not applicable						
**Total (95% CI)**		**587**		**378**	**100.0%**	**1.24 [0.92, 1.66]**
Total events	82		59			
Heterogeneity: $Chi^2 = 4.17$, df = 4 (P = 0.38); $I^2 = 4\%$						
Test for overall effect: Z = 1.41 (P = 0.16)						
Test for subgroup differences: $Chi^2 = 2.09$, df = 1 (P = 0.15), $I^2 = 52.2\%$						

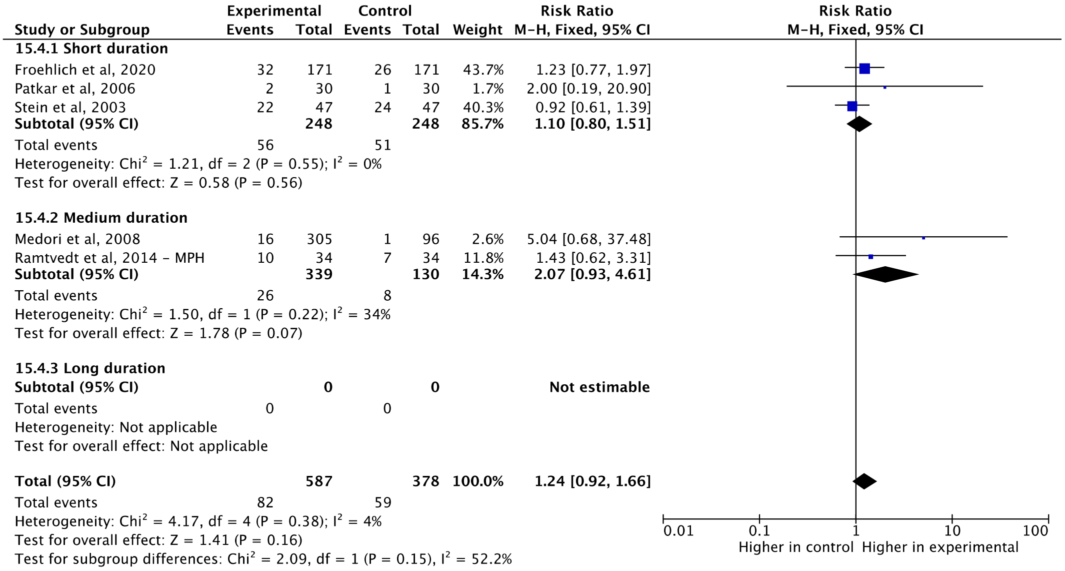

Figure legend: 95%CI, 95% confidence interval; M-H, Mantel–Haenszel; MPH, methylphenidate. No study encompassing long duration was included in the meta-analysis.

eFigure 102. Forest plot showing the risk ratio of **decreased appetite** between control and experimental groups in participants using **medium** dose **methylphenidate** subdivided by duration of use.

Study or Subgroup	Experimental Events	Experimental Total	Control Events	Control Total	Weight	Risk Ratio M-H, Random, 95% CI
**15.1.1 Short duration**						
Froehlich et al, 2020	106	171	45	171	10.6%	2.36 [1.79, 3.11]
Huang et al, 2021	53	110	1	101	2.5%	48.66 [6.86, 345.42]
Muniz et al, 2008	3	332	0	83	1.3%	1.77 [0.09, 33.86]
Patkar et al, 2006	3	30	1	30	2.1%	3.00 [0.33, 27.23]
Stein et al, 2003	35	47	16	47	9.7%	2.19 [1.42, 3.37]
**Subtotal (95% CI)**		**690**		**432**	**26.0%**	**3.10 [1.54, 6.24]**
Total events	200		63			
Heterogeneity: Tau² = 0.30; Chi² = 13.68, df = 4 (P = 0.008); I² = 71%						
Test for overall effect: Z = 3.17 (P = 0.002)						
**15.1.2 Medium duration**						
Biederman et al, 2006	23	67	2	74	4.0%	12.70 [3.11, 51.85]
Childress et al, 2009	22	182	3	63	5.0%	2.54 [0.79, 8.19]
Coghill et al, 2013 - MPH	17	111	3	110	4.9%	5.62 [1.69, 18.62]
Findling et al, 2008 - MPH	25	98	4	85	5.8%	5.42 [1.97, 14.95]
Goodman et al, 2017	25	174	7	175	7.0%	3.59 [1.60, 8.09]
Medori et al, 2008	77	305	7	96	7.5%	3.46 [1.65, 7.25]
Newcorn et al, 2008	37	219	2	74	4.0%	6.25 [1.54, 25.31]
Ramtvedt et al, 2014 - MPH	25	34	17	34	9.9%	1.47 [0.99, 2.18]
Spencer et al, 2007	30	165	6	53	7.0%	1.61 [0.71, 3.65]
Zheng et al, 2024	33	110	2	112	4.0%	16.80 [4.13, 68.32]
**Subtotal (95% CI)**		**1465**		**876**	**59.2%**	**3.94 [2.23, 6.93]**
Total events	314		53			
Heterogeneity: Tau² = 0.56; Chi² = 34.39, df = 9 (P < 0.0001); I² = 74%						
Test for overall effect: Z = 4.74 (P < 0.00001)						
**15.1.3 Long duration**						
Casas et al, 2013	43	181	5	97	6.5%	4.61 [1.89, 11.25]
Rösler et al, 2009	70	183	9	66	8.3%	2.81 [1.49, 5.29]
**Subtotal (95% CI)**		**364**		**163**	**14.8%**	**3.31 [1.98, 5.56]**
Total events	113		14			
Heterogeneity: Tau² = 0.00; Chi² = 0.80, df = 1 (P = 0.37); I² = 0%						
Test for overall effect: Z = 4.54 (P < 0.00001)						
**Total (95% CI)**		**2519**		**1471**	**100.0%**	**3.49 [2.46, 4.96]**
Total events	627		130			
Heterogeneity: Tau² = 0.28; Chi² = 49.82, df = 16 (P < 0.0001); I² = 68%						
Test for overall effect: Z = 6.99 (P < 0.00001)						
Test for subgroup differences: Chi² = 0.32, df = 2 (P = 0.85), I² = 0%						

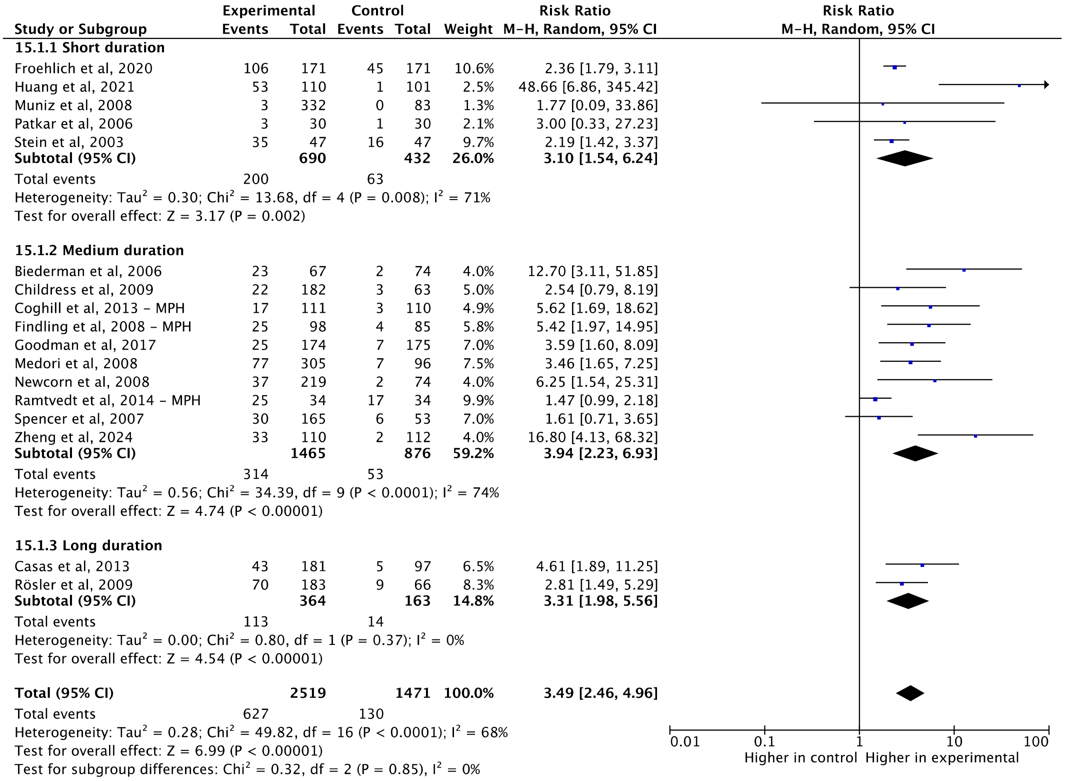

Figure legend: 95%CI, 95% confidence interval; M-H, Mantel–Haenszel; MPH, methylphenidate.

eFigure 103. Forest plot showing the risk ratio of **dry mouth** between control and experimental groups in participants using **medium** dose **methylphenidate** subdivided by duration of use.

Study or Subgroup	Experimental Events	Experimental Total	Control Events	Control Total	Weight	Risk Ratio M-H, Fixed, 95% CI
**15.6.1 Short duration**						
**Subtotal (95% CI)**		**0**		**0**		**Not estimable**
Total events	0		0			
Heterogeneity: Not applicable						
Test for overall effect: Not applicable						
**15.6.2 Medium duration**						
Biederman et al, 2006	23	67	5	74	14.7%	5.08 [2.05, 12.61]
Goodman et al, 2017	27	174	3	175	9.2%	9.05 [2.80, 29.29]
Medori et al, 2008	36	305	2	96	9.4%	5.67 [1.39, 23.10]
Spencer et al, 2007	26	165	2	53	9.3%	4.18 [1.03, 17.01]
**Subtotal (95% CI)**		**711**		**398**	**42.6%**	**5.87 [3.27, 10.56]**
Total events	112		12			
Heterogeneity: $Chi^2 = 0.85$, df = 3 (P = 0.84); $I^2 = 0\%$						
Test for overall effect: Z = 5.91 (P < 0.00001)						
**15.6.3 Long duration**						
Casas et al, 2013	32	181	3	97	12.1%	5.72 [1.80, 18.19]
Rösler et al, 2009	55	183	10	66	45.3%	1.98 [1.08, 3.66]
**Subtotal (95% CI)**		**364**		**163**	**57.4%**	**2.77 [1.62, 4.74]**
Total events	87		13			
Heterogeneity: $Chi^2 = 2.65$, df = 1 (P = 0.10); $I^2 = 62\%$						
Test for overall effect: Z = 3.71 (P = 0.0002)						
**Total (95% CI)**		**1075**		**561**	**100.0%**	**4.09 [2.76, 6.06]**
Total events	199		25			
Heterogeneity: $Chi^2 = 7.87$, df = 5 (P = 0.16); $I^2 = 36\%$						
Test for overall effect: Z = 7.03 (P < 0.00001)						
Test for subgroup differences: $Chi^2 = 3.43$, df = 1 (P = 0.06), $I^2 = 70.8\%$						

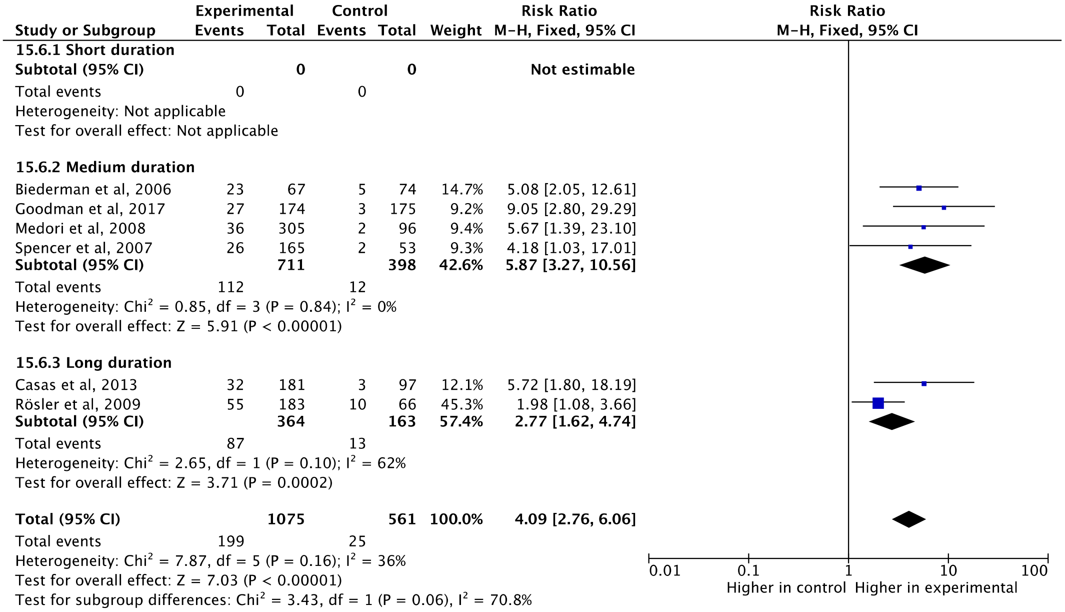

Figure legend: 95%CI, 95% confidence interval; M-H, Mantel–Haenszel. No study encompassing short duration was included in the meta-analysis.

eFigure 104. Forest plot showing the risk ratio of **headache** between control and experimental groups in participants using **medium** dose **methylphenidate** subdivided by duration of use.

Study or Subgroup	Experimental Events	Experimental Total	Control Events	Control Total	Weight	Risk Ratio M-H, Fixed, 95% CI
**15.2.1 Short duration**						
Brams et al, 2008	3	86	2	86	0.9%	1.50 [0.26, 8.75]
Huang et al, 2021	9	110	1	101	0.5%	8.26 [1.07, 64.08]
Patkar et al, 2006	3	30	2	30	0.9%	1.50 [0.27, 8.34]
Pelham Jr et al, 2001 - IR	11	36	16	36	7.1%	0.69 [0.37, 1.27]
Pelham Jr et al, 2001 - LA	8	36	16	36	7.1%	0.50 [0.25, 1.02]
Stein et al, 2003	17	47	16	47	7.1%	1.06 [0.61, 1.84]
Wilens et al, 2006	3	87	6	90	2.6%	0.52 [0.13, 2.00]
**Subtotal (95% CI)**		**432**		**426**	**26.1%**	**0.91 [0.66, 1.25]**
Total events	54		59			
Heterogeneity: Chi² = 9.59, df = 6 (P = 0.14); I² = 37%						
Test for overall effect: Z = 0.58 (P = 0.56)						
**15.2.2 Medium duration**						
Biederman et al, 2006	21	67	22	74	9.3%	1.05 [0.64, 1.74]
Childress et al, 2009	22	182	6	63	4.0%	1.27 [0.54, 2.99]
Coghill et al, 2013 - MPH	22	111	22	110	9.8%	0.99 [0.58, 1.68]
Goodman et al, 2017	33	174	20	175	8.8%	1.66 [0.99, 2.78]
Medori et al, 2008	64	305	17	96	11.5%	1.18 [0.73, 1.92]
Newcorn et al, 2008	25	219	7	74	4.6%	1.21 [0.54, 2.67]
Ramtvedt et al, 2014 - MPH	9	34	8	34	3.5%	1.13 [0.49, 2.57]
Spencer et al, 2007	52	165	10	53	6.7%	1.67 [0.91, 3.05]
**Subtotal (95% CI)**		**1257**		**679**	**58.3%**	**1.26 [1.02, 1.56]**
Total events	248		112			
Heterogeneity: Chi² = 3.38, df = 7 (P = 0.85); I² = 0%						
Test for overall effect: Z = 2.19 (P = 0.03)						
**15.2.3 Long duration**						
Casas et al, 2013	52	181	27	97	15.6%	1.03 [0.70, 1.53]
**Subtotal (95% CI)**		**181**		**97**	**15.6%**	**1.03 [0.70, 1.53]**
Total events	52		27			
Heterogeneity: Not applicable						
Test for overall effect: Z = 0.16 (P = 0.88)						
**Total (95% CI)**		**1870**		**1202**	**100.0%**	**1.14 [0.97, 1.33]**
Total events	354		198			
Heterogeneity: Chi² = 17.18, df = 15 (P = 0.31); I² = 13%						
Test for overall effect: Z = 1.55 (P = 0.12)						
Test for subgroup differences: Chi² = 3.06, df = 2 (P = 0.22), I² = 34.6%						

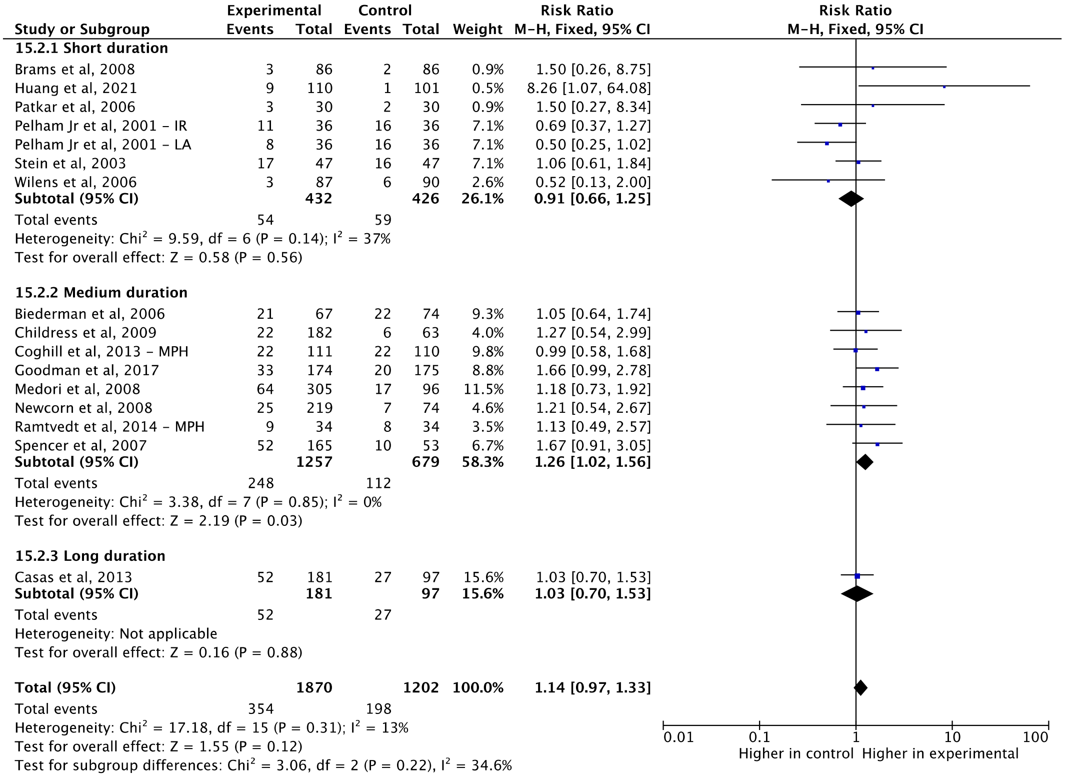

Figure legend: 95%CI, 95% confidence interval; IR, immediate release; LA, long acting; M-H, Mantel–Haenszel; MPH, methylphenidate.

eFigure 105. Forest plot showing the risk ratio of **insomnia** between control and experimental groups in participants using **medium** dose **methylphenidate** subdivided by duration of use.

Study or Subgroup	Experimental Events	Experimental Total	Control Events	Control Total	Weight	Risk Ratio M-H, Fixed, 95% CI
**15.3.1 Short duration**						
Froehlich et al, 2020	94	171	37	171	26.8%	2.54 [1.85, 3.48]
Patkar et al, 2006	3	30	3	30	2.2%	1.00 [0.22, 4.56]
Stein et al, 2003	30	47	21	47	15.2%	1.43 [0.97, 2.10]
**Subtotal (95% CI)**		**248**		**248**	**44.1%**	**2.08 [1.63, 2.66]**
Total events	127		61			
Heterogeneity: Chi² = 6.12, df = 2 (P = 0.05); I² = 67%						
Test for overall effect: Z = 5.89 (P < 0.00001)						
**15.3.2 Medium duration**						
Biederman et al, 2006	12	67	4	74	2.7%	3.31 [1.12, 9.78]
Findling et al, 2008 - MPH	13	98	4	85	3.1%	2.82 [0.96, 8.32]
Medori et al, 2008	41	305	7	96	7.7%	1.84 [0.86, 3.97]
Newcorn et al, 2008	17	219	1	74	1.1%	5.74 [0.78, 42.42]
Ramtvedt et al, 2014 - MPH	18	34	14	34	10.1%	1.29 [0.77, 2.14]
Spencer et al, 2007	27	165	6	53	6.6%	1.45 [0.63, 3.31]
Zheng et al, 2024	3	110	2	112	1.4%	1.53 [0.26, 8.96]
**Subtotal (95% CI)**		**998**		**528**	**32.8%**	**1.92 [1.37, 2.70]**
Total events	131		38			
Heterogeneity: Chi² = 5.51, df = 6 (P = 0.48); I² = 0%						
Test for overall effect: Z = 3.76 (P = 0.0002)						
**15.3.3 Long duration**						
Casas et al, 2013	28	181	11	97	10.4%	1.36 [0.71, 2.62]
Rösler et al, 2009	46	183	12	66	12.8%	1.38 [0.78, 2.44]
**Subtotal (95% CI)**		**364**		**163**	**23.1%**	**1.37 [0.89, 2.11]**
Total events	74		23			
Heterogeneity: Chi² = 0.00, df = 1 (P = 0.98); I² = 0%						
Test for overall effect: Z = 1.45 (P = 0.15)						
**Total (95% CI)**		**1610**		**939**	**100.0%**	**1.87 [1.56, 2.24]**
Total events	332		122			
Heterogeneity: Chi² = 13.43, df = 11 (P = 0.27); I² = 18%						
Test for overall effect: Z = 6.73 (P < 0.00001)						
Test for subgroup differences: Chi² = 2.73, df = 2 (P = 0.26), I² = 26.8%						

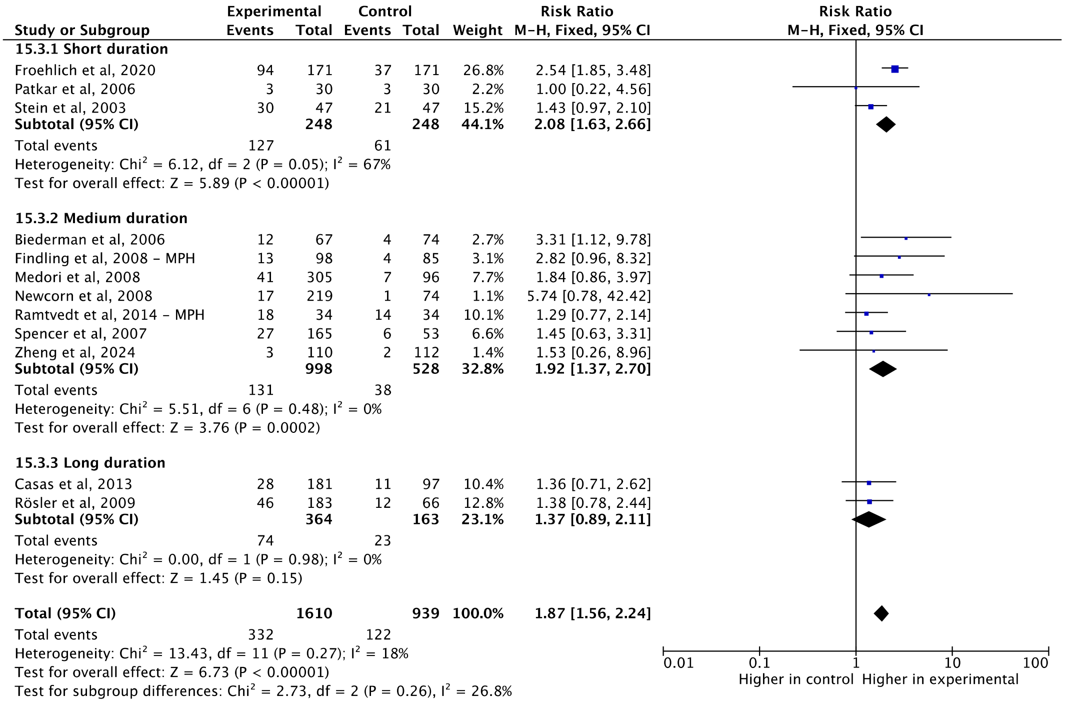

Figure legend: 95%CI, 95% confidence interval; M-H, Mantel–Haenszel; MPH, methylphenidate.

eFigure 106. Forest plot showing the risk ratio of **irritability** between control and experimental groups in participants using **medium** dose **methylphenidate** subdivided by duration of use.

Study or Subgroup	Experimental Events	Experimental Total	Control Events	Control Total	Weight	Risk Ratio M-H, Fixed, 95% CI
**15.7.1 Short duration**						
Froehlich et al, 2020	77	171	67	171	54.7%	1.15 [0.90, 1.48]
Stein et al, 2003	33	47	27	47	22.0%	1.22 [0.90, 1.66]
**Subtotal (95% CI)**		**218**		**218**	**76.7%**	**1.17 [0.96, 1.43]**
Total events	110		94			
Heterogeneity: $Chi^2 = 0.10$, df = 1 (P = 0.76); $I^2 = 0\%$						
Test for overall effect: Z = 1.55 (P = 0.12)						
**15.7.2 Medium duration**						
Medori et al, 2008	17	305	1	96	1.2%	5.35 [0.72, 39.68]
Ramtvedt et al, 2014 - MPH	22	34	27	34	22.0%	0.81 [0.60, 1.10]
**Subtotal (95% CI)**		**339**		**130**	**23.3%**	**1.06 [0.75, 1.50]**
Total events	39		28			
Heterogeneity: $Chi^2 = 5.37$, df = 1 (P = 0.02); $I^2 = 81\%$						
Test for overall effect: Z = 0.31 (P = 0.76)						
**15.7.3 Long duration**						
**Subtotal (95% CI)**		**0**		**0**		**Not estimable**
Total events	0		0			
Heterogeneity: Not applicable						
Test for overall effect: Not applicable						
**Total (95% CI)**		**557**		**348**	**100.0%**	**1.14 [0.96, 1.36]**
Total events	149		122			
Heterogeneity: $Chi^2 = 7.32$, df = 3 (P = 0.06); $I^2 = 59\%$						
Test for overall effect: Z = 1.53 (P = 0.13)						
Test for subgroup differences: $Chi^2 = 0.25$, df = 1 (P = 0.62), $I^2 = 0\%$						

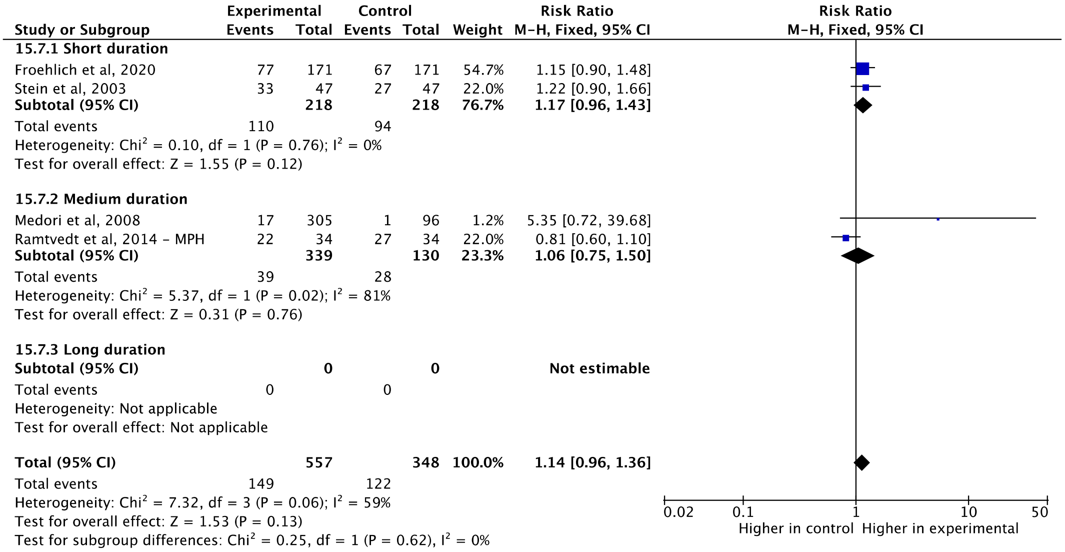

Figure legend: 95%CI, 95% confidence interval; ER, extended release; IR, immediate release; M-H, Mantel–Haenszel; MPH, methylphenidate; OROS, osmotic release oral system. No study encompassing long duration was included in the meta-analysis.

eFigure 107. Forest plot showing the risk ratio of **nausea** between control and experimental groups in participants using **medium** dose **methylphenidate** subdivided by duration of use.

Study or Subgroup	Experimental Events	Experimental Total	Control Events	Control Total	Weight	Risk Ratio M–H, Fixed, 95% CI
**15.5.1 Short duration**						
Patkar et al, 2006	2	30	1	30	2.1%	2.00 [0.19, 20.90]
Wilens et al, 2006	1	87	2	90	4.2%	0.52 [0.05, 5.60]
**Subtotal (95% CI)**		**117**		**120**	**6.3%**	**1.02 [0.21, 4.94]**
Total events	3		3			
Heterogeneity: Chi² = 0.63, df = 1 (P = 0.43); I² = 0%						
Test for overall effect: Z = 0.02 (P = 0.98)						
**15.5.2 Medium duration**						
Coghill et al, 2013 - MPH	8	111	3	110	6.4%	2.64 [0.72, 9.70]
Findling et al, 2008 - MPH	12	198	4	85	11.9%	1.29 [0.43, 3.88]
Medori et al, 2008	39	305	4	96	13.0%	3.07 [1.13, 8.37]
Newcorn et al, 2008	13	219	6	74	19.1%	0.73 [0.29, 1.86]
Ramtvedt et al, 2014 - MPH	10	34	6	34	12.8%	1.67 [0.68, 4.07]
Zheng et al, 2024	12	110	1	112	2.1%	12.22 [1.62, 92.38]
**Subtotal (95% CI)**		**977**		**511**	**65.3%**	**2.04 [1.33, 3.13]**
Total events	94		24			
Heterogeneity: Chi² = 9.32, df = 5 (P = 0.10); I² = 46%						
Test for overall effect: Z = 3.26 (P = 0.001)						
**15.5.3 Long duration**						
Casas et al, 2013	31	181	8	97	22.2%	2.08 [0.99, 4.34]
Rösler et al, 2009	16	183	2	66	6.3%	2.89 [0.68, 12.21]
**Subtotal (95% CI)**		**364**		**163**	**28.4%**	**2.25 [1.17, 4.35]**
Total events	47		10			
Heterogeneity: Chi² = 0.16, df = 1 (P = 0.69); I² = 0%						
Test for overall effect: Z = 2.42 (P = 0.02)						
**Total (95% CI)**		**1458**		**794**	**100.0%**	**2.04 [1.44, 2.89]**
Total events	144		37			
Heterogeneity: Chi² = 10.80, df = 9 (P = 0.29); I² = 17%						
Test for overall effect: Z = 3.99 (P < 0.0001)						
Test for subgroup differences: Chi² = 0.83, df = 2 (P = 0.66), I² = 0%						

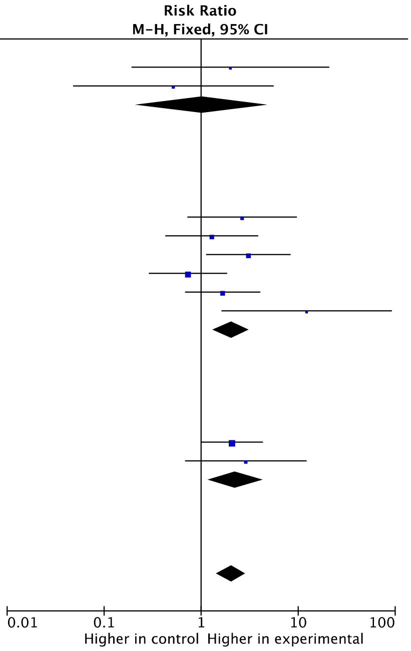

Figure legend: 95%CI, 95% confidence interval; M-H, Mantel–Haenszel; MPH, methylphenidate.

eFigure 108. Forest plot showing the mean differences in **heart rate** between control and experimental groups in participants using **medium** dose **methylphenidate** subdivided by duration of use.

Study or Subgroup	Experimental Mean	Experimental SD	Experimental Total	Control Mean	Control SD	Control Total	Weight	Mean Difference IV, Fixed, 95% CI
**15.11.1 Short duration**								
**Subtotal (95% CI)**			**0**			**0**		**Not estimable**
Heterogeneity: Not applicable								
Test for overall effect: Not applicable								
**15.11.2 Medium duration**								
Biederman et al, 2006	4.5	10.5	67	−2.7	12.4	74	15.7%	7.20 [3.42, 10.98]
Coghill et al, 2013 - MPH	5	12.8	222	−1.1	9.6	110	37.2%	6.10 [3.64, 8.56]
Newcorn et al, 2008	3	11.8	219	0.4	12	74	22.7%	2.60 [−0.55, 5.75]
Zheng et al, 2024	1.1	12.42	110	−1.9	10.59	112	24.4%	3.00 [−0.04, 6.04]
**Subtotal (95% CI)**			**618**			**370**	**100.0%**	**4.72 [3.22, 6.22]**
Heterogeneity: Chi² = 5.83, df = 3 (P = 0.12); I² = 49%								
Test for overall effect: Z = 6.17 (P < 0.00001)								
**15.11.3 Long duration**								
**Subtotal (95% CI)**			**0**			**0**		**Not estimable**
Heterogeneity: Not applicable								
Test for overall effect: Not applicable								
**Total (95% CI)**			**618**			**370**	**100.0%**	**4.72 [3.22, 6.22]**
Heterogeneity: Chi² = 5.83, df = 3 (P = 0.12); I² = 49%								
Test for overall effect: Z = 6.17 (P < 0.00001)								
Test for subgroup differences: Not applicable								

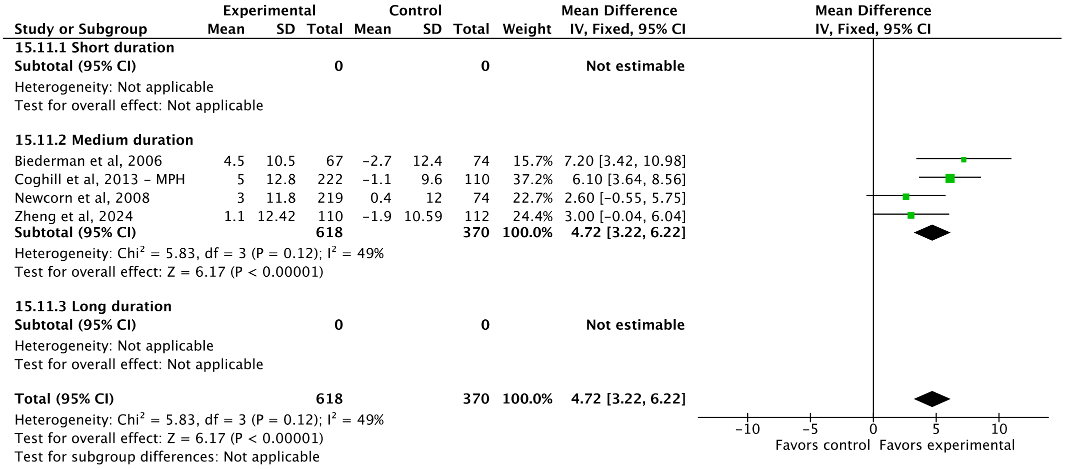

Figure legend: 95%CI, 95% confidence interval; IV, inverse variance; MPH, methylphenidate; SD, standard deviation. No study encompassing short or long duration was included in the meta-analysis.

eFigure 109. Forest plot showing the mean differences in **diastolic blood pressure** between control and experimental groups in participants using **medium** dose **methylphenidate** subdivided by duration of use.

Study or Subgroup	Experimental Mean	Experimental SD	Experimental Total	Control Mean	Control SD	Control Total	Weight	Mean Difference IV, Random, 95% CI
**15.10.1 Short duration**								
**Subtotal (95% CI)**			**0**			**0**		**Not estimable**
Heterogeneity: Not applicable								
Test for overall effect: Not applicable								
**15.10.2 Medium duration**								
Biederman et al, 2006	4	8.5	67	−2.1	8.9	74	22.2%	6.10 [3.23, 8.97]
Coghill et al, 2013 - MPH	1.7	9.9	222	1.2	8.7	110	26.3%	0.50 [−1.58, 2.58]
Newcorn et al, 2008	3.1	8.04	219	0.4	7.8	74	26.4%	2.70 [0.63, 4.77]
Zheng et al, 2024	0.3	8.39	110	0.1	9.29	112	25.1%	0.20 [−2.13, 2.53]
**Subtotal (95% CI)**			**618**			**370**	**100.0%**	**2.25 [−0.11, 4.61]**
Heterogeneity: $Tau^2 = 4.39$; $Chi^2 = 12.55$, df = 3 (P = 0.006); $I^2 = 76\%$								
Test for overall effect: Z = 1.87 (P = 0.06)								
**15.10.3 Long duration**								
**Subtotal (95% CI)**			**0**			**0**		**Not estimable**
Heterogeneity: Not applicable								
Test for overall effect: Not applicable								
**Total (95% CI)**			**618**			**370**	**100.0%**	**2.25 [−0.11, 4.61]**
Heterogeneity: $Tau^2 = 4.39$; $Chi^2 = 12.55$, df = 3 (P = 0.006); $I^2 = 76\%$								
Test for overall effect: Z = 1.87 (P = 0.06)								
Test for subgroup differences: Not applicable								

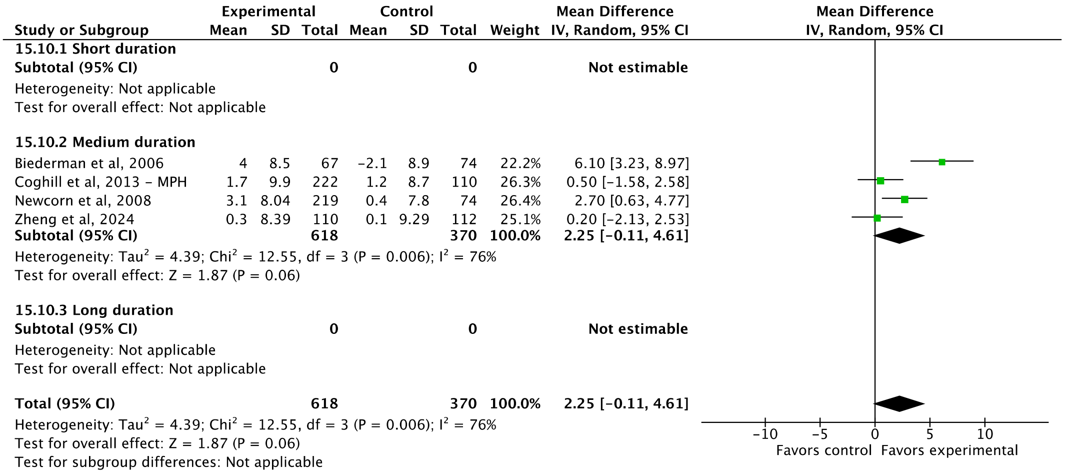

Figure legend: 95%CI, 95% confidence interval; IV, inverse variance; MPH, methylphenidate; SD, standard deviation. No study encompassing short or long duration was included in the meta-analysis.

eFigure 110. Forest plot showing the mean differences in **systolic blood pressure** between control and experimental groups in participants using **medium** dose **methylphenidate** subdivided by duration of use.

	Experimental			Control				Mean Difference
**Study or Subgroup**	**Mean**	**SD**	**Total**	**Mean**	**SD**	**Total**	**Weight**	**IV, Fixed, 95% CI**
**15.9.1 Short duration**								
**Subtotal (95% CI)**			**0**			**0**		**Not estimable**
Heterogeneity: Not applicable								
Test for overall effect: Not applicable								
**15.9.2 Medium duration**								
Biederman et al, 2006	3.5	11.8	67	−1.1	11.4	74	11.4%	4.60 [0.76, 8.44]
Coghill et al, 2013 - MPH	0.3	11.1	222	1	9.6	110	31.3%	−0.70 [−3.01, 1.61]
Newcorn et al, 2008	2.4	9.7	219	1.3	8	74	33.6%	1.10 [−1.13, 3.33]
Zheng et al, 2024	1.7	9.29	110	1.7	10.83	112	23.8%	0.00 [−2.65, 2.65]
**Subtotal (95% CI)**			**618**			**370**	**100.0%**	**0.67 [−0.62, 1.97]**
Heterogeneity: $Chi^2 = 5.76$, df = 3 (P = 0.12); $I^2 = 48\%$								
Test for overall effect: Z = 1.02 (P = 0.31)								
**15.9.3 Long duration**								
**Subtotal (95% CI)**			**0**			**0**		**Not estimable**
Heterogeneity: Not applicable								
Test for overall effect: Not applicable								
**Total (95% CI)**			**618**			**370**	**100.0%**	**0.67 [−0.62, 1.97]**
Heterogeneity: $Chi^2 = 5.76$, df = 3 (P = 0.12); $I^2 = 48\%$								
Test for overall effect: Z = 1.02 (P = 0.31)								
Test for subgroup differences: Not applicable								

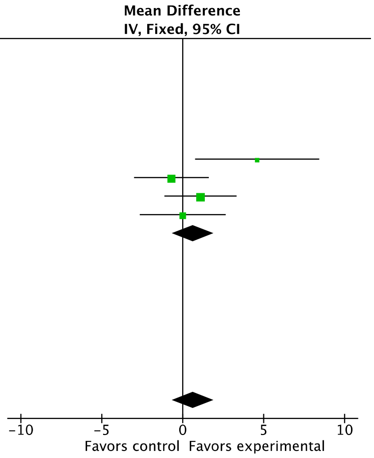

Figure legend: 95%CI, 95% confidence interval; IV, inverse variance; MPH, methylphenidate; SD, standard deviation. No study encompassing short or long duration was included in the meta-analysis.

eFigure 111. Forest plot showing the risk ratio of **all adverse events** between control and experimental groups in participants using **high** dose **methylphenidate** subdivided by duration of use.

	Experimental		Control			Risk Ratio
**Study or Subgroup**	**Events**	**Total**	**Events**	**Total**	**Weight**	**M-H, Random, 95% CI**
**18.8.1 Short duration**						
Childress et al, 2020A	24	65	22	54	6.0%	0.91 [0.58, 1.42]
Childress et al, 2020B	18	75	7	73	2.4%	2.50 [1.11, 5.63]
Childress et al, 2022	25	121	18	118	4.6%	1.35 [0.78, 2.35]
Pliszka et al, 2017	56	81	39	80	10.8%	1.42 [1.09, 1.85]
Weiss et al, 2020	158	297	25	78	8.6%	1.66 [1.18, 2.33]
Weiss et al, 2021	154	293	24	74	8.4%	1.62 [1.15, 2.29]
**Subtotal (95% CI)**		**932**		**477**	**40.8%**	**1.45 [1.19, 1.77]**
Total events	435		135			
Heterogeneity: Tau² = 0.02; Chi² = 7.06, df = 5 (P = 0.22); I² = 29%						
Test for overall effect: Z = 3.72 (P = 0.0002)						
**18.8.2 Medium duration**						
Adler et al, 2009	93	110	74	116	15.0%	1.33 [1.13, 1.55]
Buitelaar et al, 2012	7	23	8	22	2.3%	0.84 [0.37, 1.92]
Huss et al, 2014	401	542	108	180	16.1%	1.23 [1.08, 1.40]
Retz et al, 2012	55	84	32	78	9.5%	1.60 [1.17, 2.17]
**Subtotal (95% CI)**		**759**		**396**	**43.0%**	**1.30 [1.16, 1.45]**
Total events	556		222			
Heterogeneity: Tau² = 0.00; Chi² = 3.46, df = 3 (P = 0.33); I² = 13%						
Test for overall effect: Z = 4.66 (P < 0.00001)						
**18.8.3 Long duration**						
Ginsberg et al, 2014	175	216	65	82	16.2%	1.02 [0.90, 1.16]
**Subtotal (95% CI)**		**216**		**82**	**16.2%**	**1.02 [0.90, 1.16]**
Total events	175		65			
Heterogeneity: Not applicable						
Test for overall effect: Z = 0.33 (P = 0.74)						
**Total (95% CI)**		**1907**		**955**	**100.0%**	**1.31 [1.15, 1.50]**
Total events	1166		422			
Heterogeneity: Tau² = 0.02; Chi² = 26.19, df = 10 (P = 0.003); I² = 62%						
Test for overall effect: Z = 3.96 (P < 0.0001)						
Test for subgroup differences: Chi² = 11.48, df = 2 (P = 0.003), I² = 82.6%						

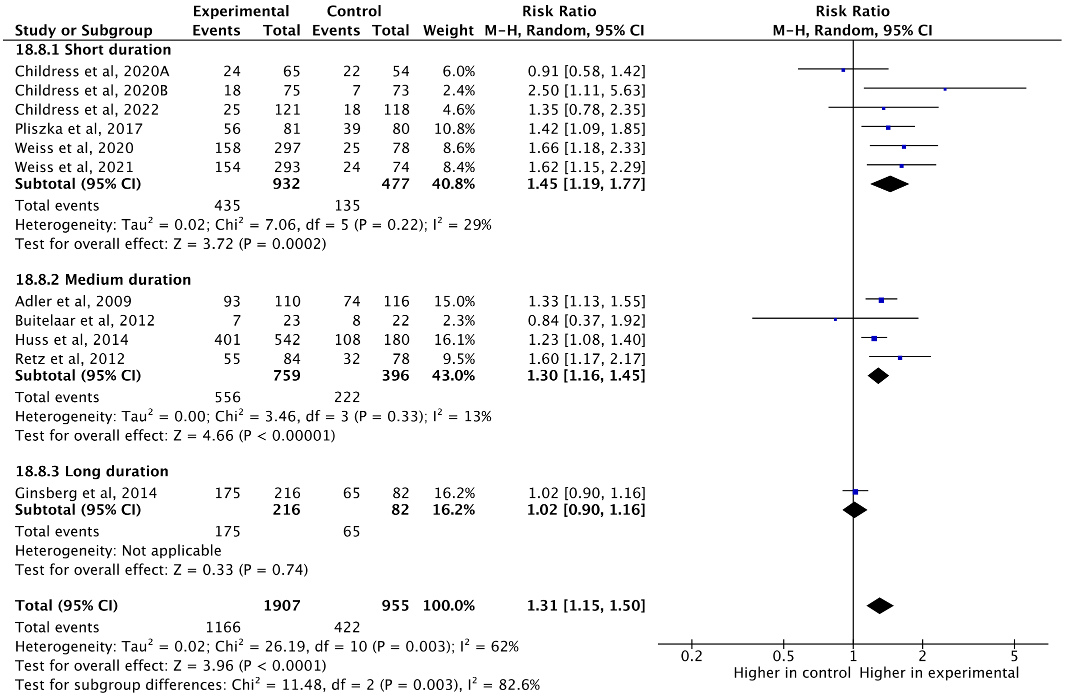

Figure legend: 95%CI, 95% confidence interval; M-H, Mantel–Haenszel.

eFigure 112. Forest plot showing the risk ratio of **anxiety** between control and experimental groups in participants using **high** dose **methylphenidate** subdivided by duration of use.

Study or Subgroup	Experimental Events	Experimental Total	Control Events	Control Total	Weight	Risk Ratio M-H, Random, 95% CI
**18.4.1 Short duration**						
**Subtotal (95% CI)**		**0**		**0**		**Not estimable**
Total events	0		0			
Heterogeneity: Not applicable						
Test for overall effect: Not applicable						
**18.4.2 Medium duration**						
Adler et al, 2009	18	110	4	116	55.1%	4.75 [1.66, 13.58]
**Subtotal (95% CI)**		**110**		**116**	**55.1%**	**4.75 [1.66, 13.58]**
Total events	18		4			
Heterogeneity: Not applicable						
Test for overall effect: Z = 2.90 (P = 0.004)						
**18.4.3 Long duration**						
Konstenius et al, 2014	1	27	4	27	44.9%	0.25 [0.03, 2.09]
**Subtotal (95% CI)**		**27**		**27**	**44.9%**	**0.25 [0.03, 2.09]**
Total events	1		4			
Heterogeneity: Not applicable						
Test for overall effect: Z = 1.28 (P = 0.20)						
**Total (95% CI)**		**137**		**143**	**100.0%**	**1.27 [0.07, 22.45]**
Total events	19		8			
Heterogeneity: Tau² = 3.62; Chi² = 5.94, df = 1 (P = 0.01); I² = 83%						
Test for overall effect: Z = 0.16 (P = 0.87)						
Test for subgroup differences: Chi² = 5.92, df = 1 (P = 0.01), I² = 83.1%						

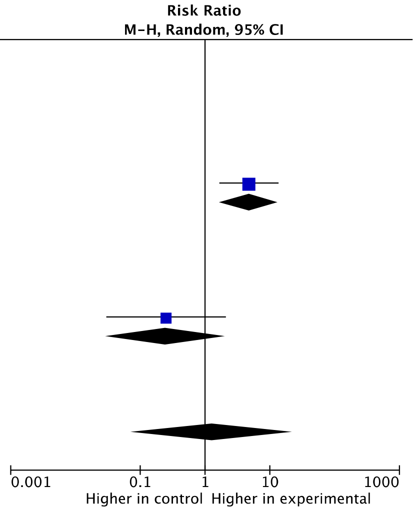

Figure legend: 95%CI, 95% confidence interval; M-H, Mantel–Haenszel. No study encompassing short duration was included in the meta-analysis.

eFigure 113. Forest plot showing the risk ratio of **decreased appetite** between control and experimental groups in participants using **high** dose **methylphenidate** subdivided by duration of use.

Study or Subgroup	Experimental Events	Experimental Total	Control Events	Control Total	Weight	Risk Ratio M-H, Random, 95% CI
**18.1.1 Short duration**						
Pliszka et al, 2017	15	81	3	80	13.5%	4.94 [1.49, 16.40]
Weiss et al, 2020	33	297	2	78	12.1%	4.33 [1.06, 17.67]
**Subtotal (95% CI)**		**378**		**158**	**25.5%**	**4.67 [1.88, 11.64]**
Total events	48		5			
Heterogeneity: Tau^2 = 0.00; Chi^2 = 0.02, df = 1 (P = 0.89); I^2 = 0%						
Test for overall effect: Z = 3.31 (P = 0.0009)						
**18.1.2 Medium duration**						
Adler et al, 2009	28	110	7	116	16.4%	4.22 [1.92, 9.26]
Chronis-Tuscano et al, 2008	2	20	1	23	7.2%	2.30 [0.23, 23.51]
Huss et al, 2014	136	542	8	180	17.0%	5.65 [2.82, 11.29]
Retz et al, 2012	40	84	8	78	17.0%	4.64 [2.32, 9.29]
**Subtotal (95% CI)**		**756**		**397**	**57.7%**	**4.74 [3.15, 7.14]**
Total events	206		24			
Heterogeneity: Tau^2 = 0.00; Chi^2 = 0.73, df = 3 (P = 0.87); I^2 = 0%						
Test for overall effect: Z = 7.45 (P < 0.00001)						
**18.1.3 Long duration**						
Ginsberg et al, 2014	15	216	11	82	16.8%	0.52 [0.25, 1.08]
**Subtotal (95% CI)**		**216**		**82**	**16.8%**	**0.52 [0.25, 1.08]**
Total events	15		11			
Heterogeneity: Not applicable						
Test for overall effect: Z = 1.75 (P = 0.08)						
**Total (95% CI)**		**1350**		**637**	**100.0%**	**3.11 [1.41, 6.83]**
Total events	269		40			
Heterogeneity: Tau^2 = 0.82; Chi^2 = 29.07, df = 6 (P < 0.0001); I^2 = 79%						
Test for overall effect: Z = 2.82 (P = 0.005)						
Test for subgroup differences: Chi^2 = 27.63, df = 2 (P < 0.00001), I^2 = 92.8%						

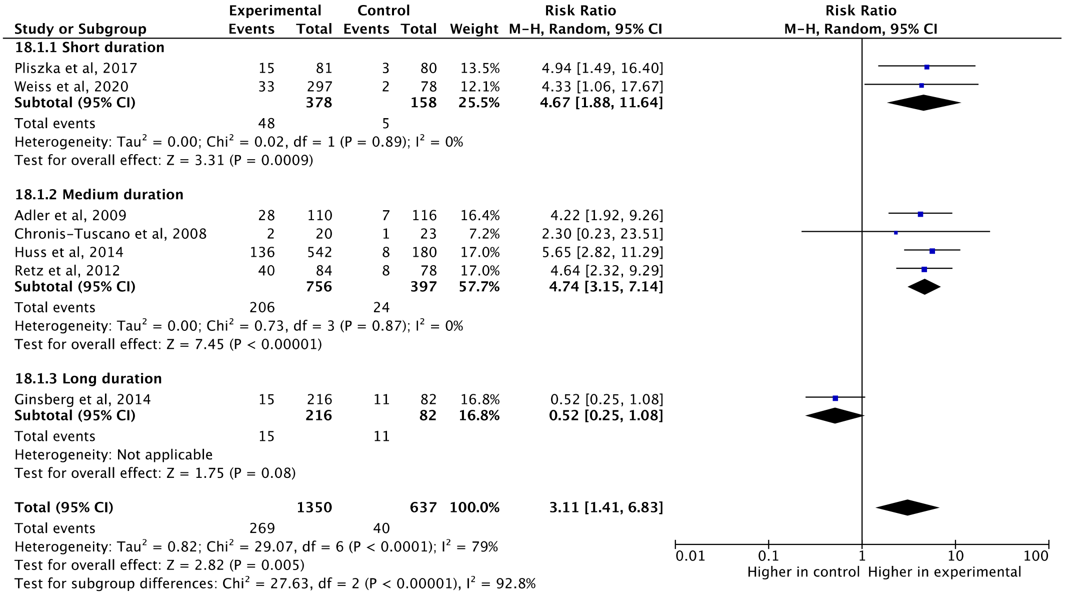

Figure legend: 95%CI, 95% confidence interval; M-H, Mantel–Haenszel.

eFigure 114. Forest plot showing the risk ratio of **dry mouth** between control and experimental groups in participants using **high** dose **methylphenidate** subdivided by duration of use.

Study or Subgroup	Experimental Events	Experimental Total	Control Events	Control Total	Weight	Risk Ratio M-H, Random, 95% CI
**18.6.1 Short duration**						
Weiss et al, 2020	27	297	3	78	15.8%	2.36 [0.74, 7.59]
**Subtotal (95% CI)**		**297**		**78**	**15.8%**	**2.36 [0.74, 7.59]**
Total events	27		3			
Heterogeneity: Not applicable						
Test for overall effect: Z = 1.45 (P = 0.15)						
**18.6.2 Medium duration**						
Adler et al, 2009	22	110	6	116	18.6%	3.87 [1.63, 9.18]
Huss et al, 2014	110	542	4	180	17.5%	9.13 [3.42, 24.41]
Retz et al, 2012	32	84	11	78	20.8%	2.70 [1.47, 4.98]
**Subtotal (95% CI)**		**736**		**374**	**56.9%**	**4.26 [2.03, 8.93]**
Total events	164		21			
Heterogeneity: $Tau^2 = 0.26$; $Chi^2 = 5.02$, df = 2 (P = 0.08); $I^2 = 60\%$						
Test for overall effect: Z = 3.84 (P = 0.0001)						
**18.6.3 Long duration**						
Ginsberg et al, 2014	15	216	9	82	19.3%	0.63 [0.29, 1.39]
Konstenius et al, 2014	2	27	1	27	7.9%	2.00 [0.19, 20.77]
**Subtotal (95% CI)**		**243**		**109**	**27.2%**	**0.71 [0.34, 1.50]**
Total events	17		10			
Heterogeneity: $Tau^2 = 0.00$; $Chi^2 = 0.84$, df = 1 (P = 0.36); $I^2 = 0\%$						
Test for overall effect: Z = 0.90 (P = 0.37)						
**Total (95% CI)**		**1276**		**561**	**100.0%**	**2.58 [1.15, 5.78]**
Total events	208		34			
Heterogeneity: $Tau^2 = 0.72$; $Chi^2 = 21.05$, df = 5 (P = 0.0008); $I^2 = 76\%$						
Test for overall effect: Z = 2.30 (P = 0.02)						
Test for subgroup differences: $Chi^2 = 11.36$, df = 2 (P = 0.003), $I^2 = 82.4\%$						

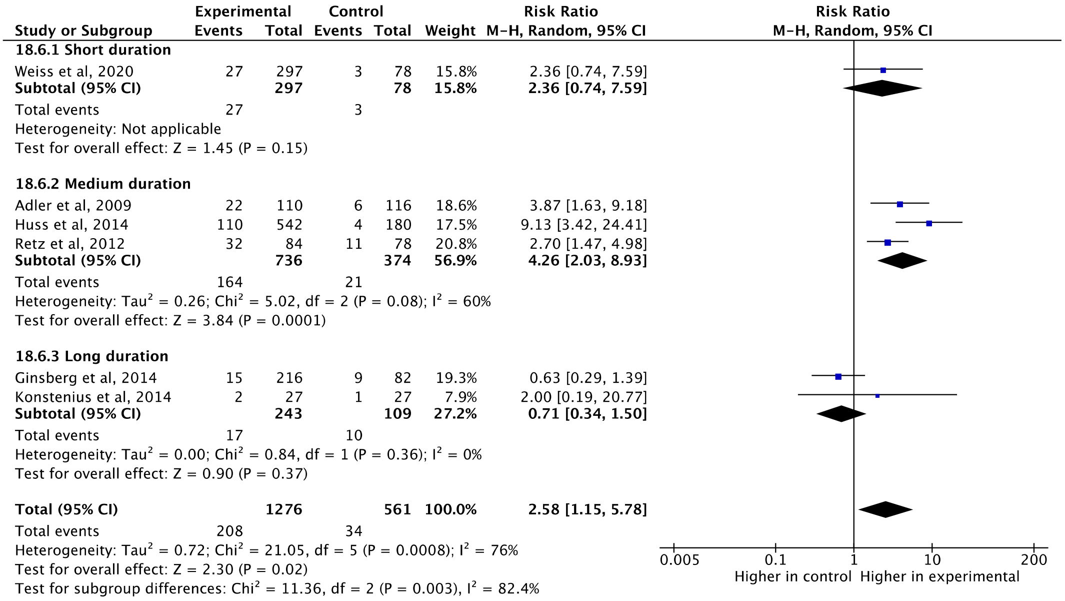

Figure legend: 95%CI, 95% confidence interval; M-H, Mantel–Haenszel.

eFigure 115. Forest plot showing the risk ratio of **headache** between control and experimental groups in participants using **high** dose **methylphenidate** subdivided by duration of use.

Study or Subgroup	Experimental Events	Experimental Total	Control Events	Control Total	Weight	Risk Ratio M-H, Fixed, 95% CI
**18.2.1 Short duration**						
Childress et al, 2020A	1	65	1	54	0.8%	0.83 [0.05, 12.97]
Pliszka et al, 2017	6	81	3	80	2.2%	1.98 [0.51, 7.63]
Shram et al, 2022	5	48	2	46	1.5%	2.40 [0.49, 11.74]
Weiss et al, 2020	52	297	9	78	10.3%	1.52 [0.78, 2.94]
Weiss et al, 2021	44	293	7	74	8.1%	1.59 [0.75, 3.38]
**Subtotal (95% CI)**		**784**		**332**	**22.9%**	**1.62 [1.04, 2.52]**
Total events	108		22			
Heterogeneity: $Chi^2 = 0.58$, df = 4 (P = 0.96); $I^2 = 0\%$						
Test for overall effect: Z = 2.14 (P = 0.03)						
**18.2.2 Medium duration**						
Adler et al, 2009	28	110	16	116	11.3%	1.85 [1.06, 3.22]
Huss et al, 2014	111	542	30	180	32.6%	1.23 [0.85, 1.77]
Retz et al, 2012	25	84	13	78	9.8%	1.79 [0.98, 3.24]
**Subtotal (95% CI)**		**736**		**374**	**53.6%**	**1.46 [1.11, 1.91]**
Total events	164		59			
Heterogeneity: $Chi^2 = 1.97$, df = 2 (P = 0.37); $I^2 = 0\%$						
Test for overall effect: Z = 2.74 (P = 0.006)						
**18.2.3 Long duration**						
Ginsberg et al, 2014	41	216	21	82	22.0%	0.74 [0.47, 1.17]
Konstenius et al, 2014	6	27	2	27	1.4%	3.00 [0.66, 13.56]
**Subtotal (95% CI)**		**243**		**109**	**23.5%**	**0.88 [0.57, 1.36]**
Total events	47		23			
Heterogeneity: $Chi^2 = 3.07$, df = 1 (P = 0.08); $I^2 = 67\%$						
Test for overall effect: Z = 0.58 (P = 0.56)						
**Total (95% CI)**		**1763**		**815**	**100.0%**	**1.36 [1.11, 1.67]**
Total events	319		104			
Heterogeneity: $Chi^2 = 11.16$, df = 9 (P = 0.26); $I^2 = 19\%$						
Test for overall effect: Z = 2.96 (P = 0.003)						
Test for subgroup differences: $Chi^2 = 4.72$, df = 2 (P = 0.09), $I^2 = 57.6\%$						

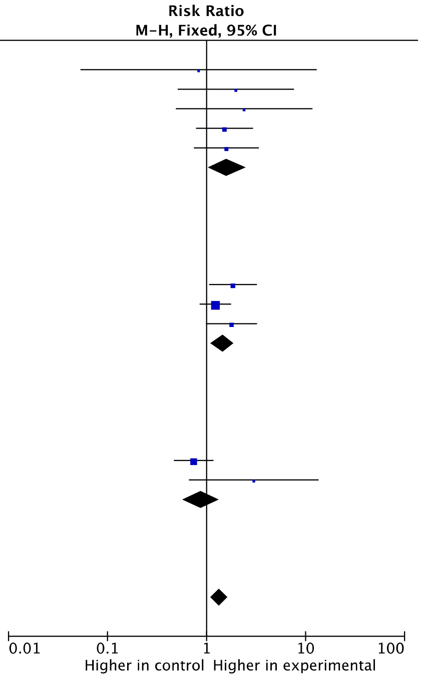

Figure legend: 95%CI, 95% confidence interval; M-H, Mantel–Haenszel.

eFigure 116. Forest plot showing the risk ratio of **insomnia** between control and experimental groups in participants using **high** dose **methylphenidate** subdivided by duration of use.

Study or Subgroup	Experimental Events	Experimental Total	Control Events	Control Total	Weight	Risk Ratio M-H, Random, 95% CI
**18.3.1 Short duration**						
Childress et al, 2020A	5	65	5	54	14.8%	0.83 [0.25, 2.72]
Pliszka et al, 2017	27	81	9	80	43.9%	2.96 [1.49, 5.90]
Weiss et al, 2020	18	297	1	78	5.2%	4.73 [0.64, 34.87]
Weiss et al, 2021	19	293	1	74	5.2%	4.80 [0.65, 35.27]
**Subtotal (95% CI)**		**736**		**286**	**69.1%**	**2.36 [1.09, 5.12]**
Total events	69		16			
Heterogeneity: Tau² = 0.21; Chi² = 4.42, df = 3 (P = 0.22); I² = 32%						
Test for overall effect: Z = 2.17 (P = 0.03)						
**18.3.2 Medium duration**						
Adler et al, 2009	10	110	6	116	21.7%	1.76 [0.66, 4.67]
**Subtotal (95% CI)**		**110**		**116**	**21.7%**	**1.76 [0.66, 4.67]**
Total events	10		6			
Heterogeneity: Not applicable						
Test for overall effect: Z = 1.13 (P = 0.26)						
**18.3.3 Long duration**						
Konstenius et al, 2014	6	27	2	27	9.1%	3.00 [0.66, 13.56]
**Subtotal (95% CI)**		**27**		**27**	**9.1%**	**3.00 [0.66, 13.56]**
Total events	6		2			
Heterogeneity: Not applicable						
Test for overall effect: Z = 1.43 (P = 0.15)						
**Total (95% CI)**		**873**		**429**	**100.0%**	**2.31 [1.46, 3.64]**
Total events	85		24			
Heterogeneity: Tau² = 0.00; Chi² = 4.86, df = 5 (P = 0.43); I² = 0%						
Test for overall effect: Z = 3.59 (P = 0.0003)						
Test for subgroup differences: Chi² = 0.40, df = 2 (P = 0.82), I² = 0%						

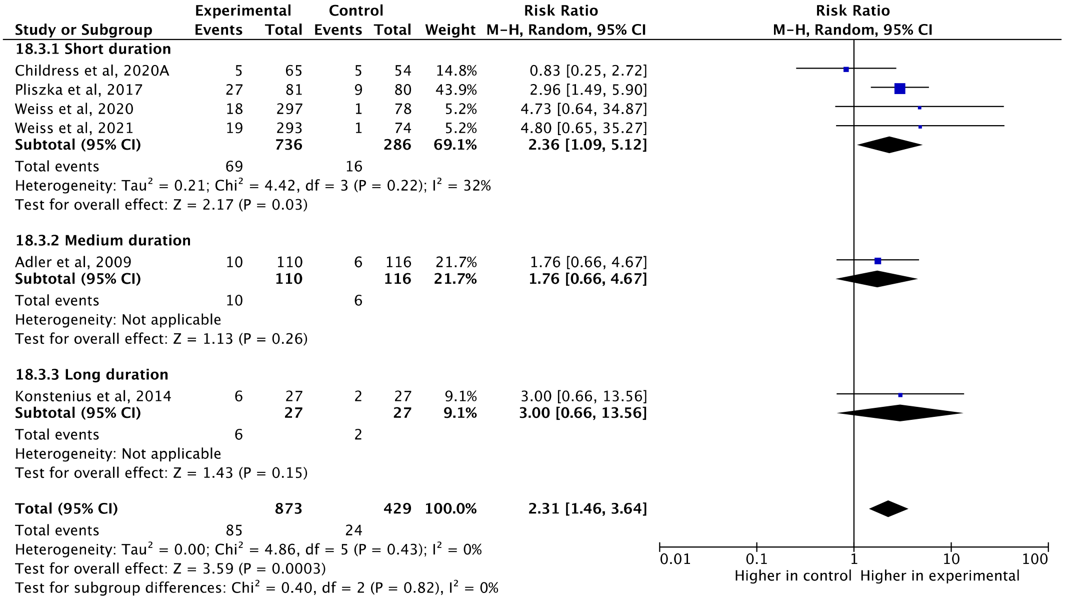

Figure legend: 95%CI, 95% confidence interval; M-H, Mantel–Haenszel.

eFigure 117. Forest plot showing the risk ratio of **irritability** between control and experimental groups in participants using **high** dose **methylphenidate** subdivided by duration of use.

Study or Subgroup	Experimental Events	Experimental Total	Control Events	Control Total	Weight	Risk Ratio M-H, Fixed, 95% CI
**18.7.1 Short duration**						
Weiss et al, 2020	16	297	4	78	31.3%	1.05 [0.36, 3.05]
Weiss et al, 2021	24	293	7	74	55.2%	0.87 [0.39, 1.93]
**Subtotal (95% CI)**		**590**		**152**	**86.5%**	**0.93 [0.49, 1.77]**
Total events	40		11			
Heterogeneity: Chi^2 = 0.08, df = 1 (P = 0.78); I^2 = 0%						
Test for overall effect: Z = 0.21 (P = 0.83)						
**18.7.2 Medium duration**						
Adler et al, 2009	7	110	2	116	9.6%	3.69 [0.78, 17.38]
Chronis-Tuscano et al, 2008	3	15	1	23	3.9%	4.60 [0.53, 40.19]
**Subtotal (95% CI)**		**125**		**139**	**13.5%**	**3.95 [1.12, 13.95]**
Total events	10		3			
Heterogeneity: Chi^2 = 0.03, df = 1 (P = 0.87); I^2 = 0%						
Test for overall effect: Z = 2.14 (P = 0.03)						
**18.7.3 Long duration**						
**Subtotal (95% CI)**		**0**		**0**		**Not estimable**
Total events	0		0			
Heterogeneity: Not applicable						
Test for overall effect: Not applicable						
**Total (95% CI)**		**715**		**291**	**100.0%**	**1.34 [0.78, 2.32]**
Total events	50		14			
Heterogeneity: Chi^2 = 4.22, df = 3 (P = 0.24); I^2 = 29%						
Test for overall effect: Z = 1.05 (P = 0.29)						
Test for subgroup differences: Chi^2 = 4.00, df = 1 (P = 0.05), I^2 = 75.0%						

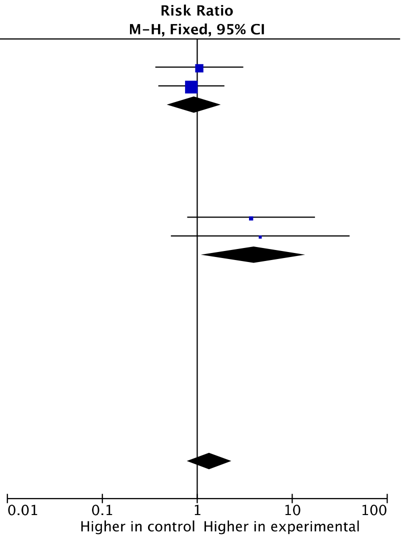

Figure legend: 95%CI, 95% confidence interval; M-H, Mantel–Haenszel. No study encompassing long duration was included in the meta-analysis.

eFigure 118. Forest plot showing the risk ratio of **nausea** between control and experimental groups in participants using **high** dose **methylphenidate** subdivided by duration of use.

Study or Subgroup	Experimental Events	Experimental Total	Control Events	Control Total	Weight	Risk Ratio M-H, Fixed, 95% CI
**18.5.1 Short duration**						
Weiss et al, 2020	18	297	2	78	11.5%	2.36 [0.56, 9.97]
Weiss et al, 2021	17	293	3	74	17.4%	1.43 [0.43, 4.75]
**Subtotal (95% CI)**		**590**		**152**	**28.9%**	**1.80 [0.72, 4.52]**
Total events	35		5			
Heterogeneity: Chi^2 = 0.28, df = 1 (P = 0.60); I^2 = 0%						
Test for overall effect: Z = 1.26 (P = 0.21)						
**18.5.2 Medium duration**						
Adler et al, 2009	14	110	3	116	10.6%	4.92 [1.45, 16.66]
Huss et al, 2014	58	542	9	180	49.1%	2.14 [1.08, 4.23]
Retz et al, 2012	14	84	3	78	11.3%	4.33 [1.29, 14.51]
**Subtotal (95% CI)**		**736**		**374**	**71.1%**	**2.90 [1.72, 4.91]**
Total events	86		15			
Heterogeneity: Chi^2 = 1.91, df = 2 (P = 0.38); I^2 = 0%						
Test for overall effect: Z = 3.99 (P < 0.0001)						
**18.5.3 Long duration**						
**Subtotal (95% CI)**		**0**		**0**		**Not estimable**
Total events	0		0			
Heterogeneity: Not applicable						
Test for overall effect: Not applicable						
**Total (95% CI)**		**1326**		**526**	**100.0%**	**2.59 [1.64, 4.07]**
Total events	121		20			
Heterogeneity: Chi^2 = 3.01, df = 4 (P = 0.56); I^2 = 0%						
Test for overall effect: Z = 4.11 (P < 0.0001)						
Test for subgroup differences: Chi^2 = 0.78, df = 1 (P = 0.38), I^2 = 0%						

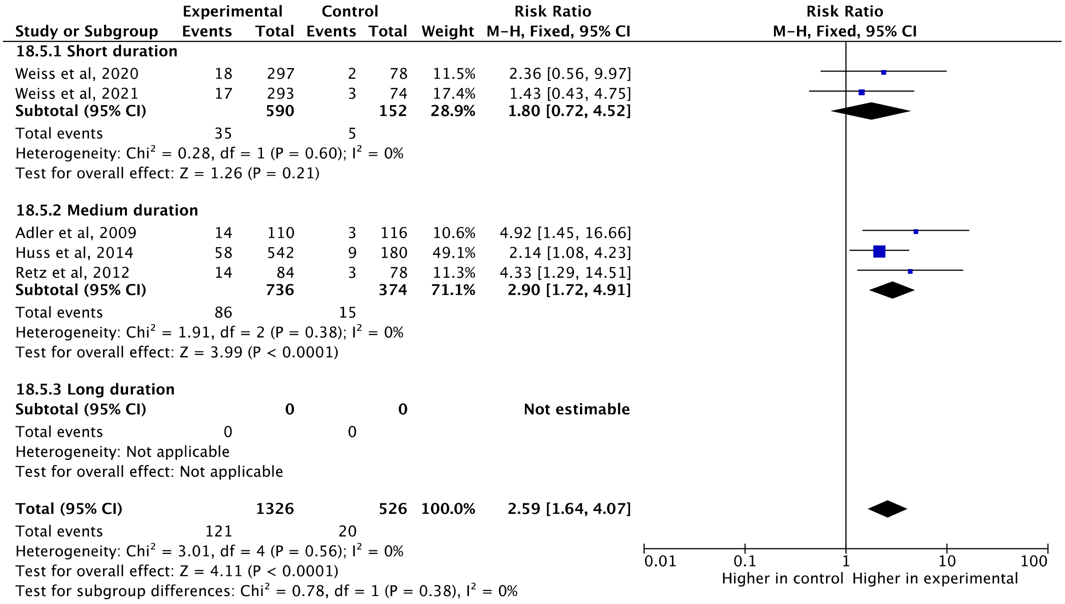

Figure legend: 95%CI, 95% confidence interval; M-H, Mantel–Haenszel. No study encompassing long duration was included in the meta-analysis.

eFigure 119. Forest plot showing the mean differences in **heart rate** between control and experimental groups in participants using **high** dose **methylphenidate** subdivided by duration of use.

Study or Subgroup	Experimental Mean	Experimental SD	Experimental Total	Control Mean	Control SD	Control Total	Weight	Mean Difference IV, Random, 95% CI
**18.11.1 Short duration**								
Childress et al, 2020A	1.7	11.12	65	−0.2	14.8	54	25.0%	1.90 [−2.88, 6.68]
Pliszka et al, 2017	3.5	11.49	81	3.1	11.58	80	32.9%	0.40 [−3.16, 3.96]
**Subtotal (95% CI)**			**146**			**134**	**57.9%**	**0.94 [−1.92, 3.79]**
Heterogeneity: $Tau^2 = 0.00$; $Chi^2 = 0.24$, df = 1 (P = 0.62); $I^2 = 0\%$								
Test for overall effect: Z = 0.64 (P = 0.52)								
**18.11.2 Medium duration**								
Adler et al, 2009	3.6	9.78	110	−1.6	8.33	116	42.1%	5.20 [2.83, 7.57]
**Subtotal (95% CI)**			**110**			**116**	**42.1%**	**5.20 [2.83, 7.57]**
Heterogeneity: Not applicable								
Test for overall effect: Z = 4.29 (P < 0.0001)								
**18.11.3 Long duration**								
**Subtotal (95% CI)**			**0**			**0**		**Not estimable**
Heterogeneity: Not applicable								
Test for overall effect: Not applicable								
**Total (95% CI)**			**256**			**250**	**100.0%**	**2.79 [−0.47, 6.06]**
Heterogeneity: $Tau^2 = 5.12$; $Chi^2 = 5.30$, df = 2 (P = 0.07); $I^2 = 62\%$								
Test for overall effect: Z = 1.68 (P = 0.09)								
Test for subgroup differences: $Chi^2 = 5.06$, df = 1 (P = 0.02), $I^2 = 80.2\%$								

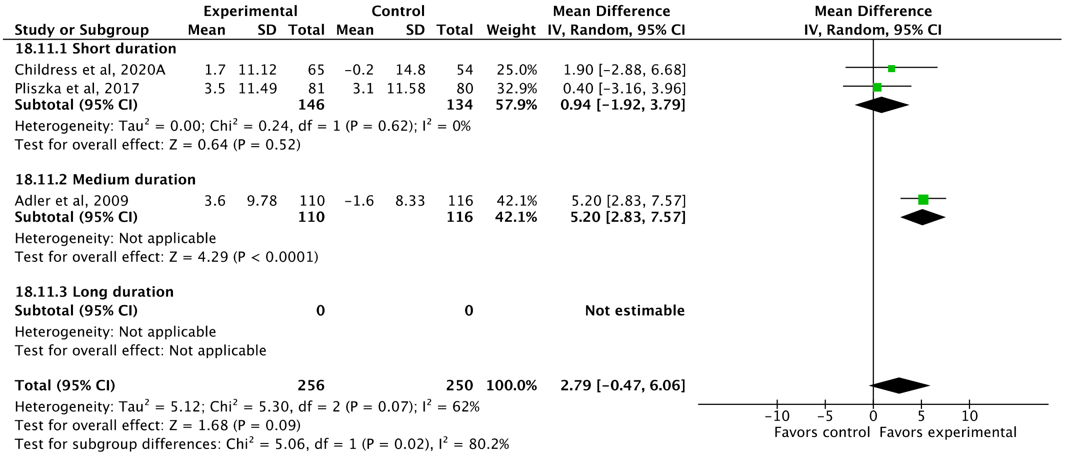

Figure legend: 95%CI, 95% confidence interval; IV, inverse variance; SD, standard deviation. No study encompassing long duration was included in the meta-analysis.

eFigure 120. Forest plot showing the mean differences in **diastolic blood pressure** between control and experimental groups in participants using **high** dose **methylphenidate** subdivided by duration of use.

Study or Subgroup	Experimental Mean	Experimental SD	Experimental Total	Control Mean	Control SD	Control Total	Weight	Mean Difference IV, Fixed, 95% CI
**18.10.1 Short duration**								
Childress et al, 2020A	5.1	8.1	65	5.9	8.27	54	21.9%	−0.80 [−3.76, 2.16]
Pliszka et al, 2017	2.5	9.06	81	−0.4	9.99	80	22.0%	2.90 [−0.05, 5.85]
**Subtotal (95% CI)**			**146**			**134**	**43.9%**	**1.06 [−1.03, 3.14]**
Heterogeneity: Chi² = 3.02, df = 1 (P = 0.08); I² = 67%								
Test for overall effect: Z = 0.99 (P = 0.32)								
**18.10.2 Medium duration**								
Adler et al, 2009	1.1	6.72	110	0.4	7.43	116	56.1%	0.70 [−1.15, 2.55]
**Subtotal (95% CI)**			**110**			**116**	**56.1%**	**0.70 [−1.15, 2.55]**
Heterogeneity: Not applicable								
Test for overall effect: Z = 0.74 (P = 0.46)								
**18.10.3 Long duration**								
**Subtotal (95% CI)**			**0**			**0**		**Not estimable**
Heterogeneity: Not applicable								
Test for overall effect: Not applicable								
**Total (95% CI)**			**256**			**250**	**100.0%**	**0.86 [−0.53, 2.24]**
Heterogeneity: Chi² = 3.08, df = 2 (P = 0.21); I² = 35%								
Test for overall effect: Z = 1.21 (P = 0.22)								
Test for subgroup differences: Chi² = 0.06, df = 1 (P = 0.80), I² = 0%								

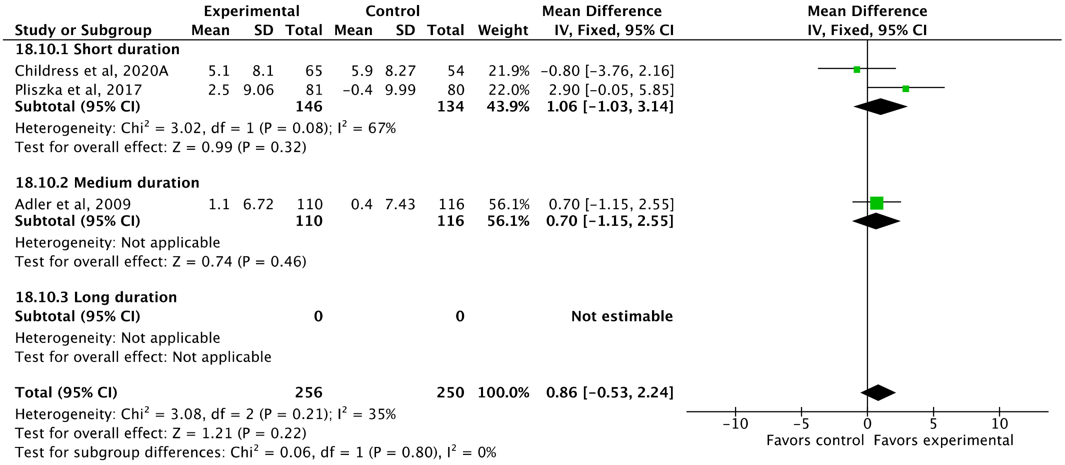

Figure legend: 95%CI, 95% confidence interval; IV, inverse variance; SD, standard deviation. No study encompassing long duration was included in the meta-analysis.

eFigure 121. Forest plot showing the mean differences in **systolic blood pressure** between control and experimental groups in participants using **high** dose **methylphenidate** subdivided by duration of use.

Study or Subgroup	Experimental Mean	Experimental SD	Experimental Total	Control Mean	Control SD	Control Total	Weight	Mean Difference IV, Fixed, 95% CI
**18.9.1 Short duration**								
Childress et al, 2020A	6.3	9.32	65	3.6	10.64	54	23.3%	2.70 [−0.93, 6.33]
Pliszka et al, 2017	1.5	12.18	81	1.1	10.72	80	24.5%	0.40 [−3.14, 3.94]
**Subtotal (95% CI)**			**146**			**134**	**47.9%**	**1.52 [−1.01, 4.06]**
Heterogeneity: $Chi^2 = 0.79$, df = 1 (P = 0.37); $I^2 = 0\%$								
Test for overall effect: Z = 1.18 (P = 0.24)								
**18.9.2 Medium duration**								
Adler et al, 2009	−1.2	8.92	110	−0.5	9.72	116	52.1%	−0.70 [−3.13, 1.73]
**Subtotal (95% CI)**			**110**			**116**	**52.1%**	**−0.70 [−3.13, 1.73]**
Heterogeneity: Not applicable								
Test for overall effect: Z = 0.56 (P = 0.57)								
**18.9.3 Long duration**								
**Subtotal (95% CI)**			**0**			**0**		**Not estimable**
Heterogeneity: Not applicable								
Test for overall effect: Not applicable								
**Total (95% CI)**			**256**			**250**	**100.0%**	**0.36 [−1.39, 2.12]**
Heterogeneity: $Chi^2 = 2.33$, df = 2 (P = 0.31); $I^2 = 14\%$								
Test for overall effect: Z = 0.41 (P = 0.68)								
Test for subgroup differences: $Chi^2 = 1.54$, df = 1 (P = 0.22), $I^2 = 34.9\%$								

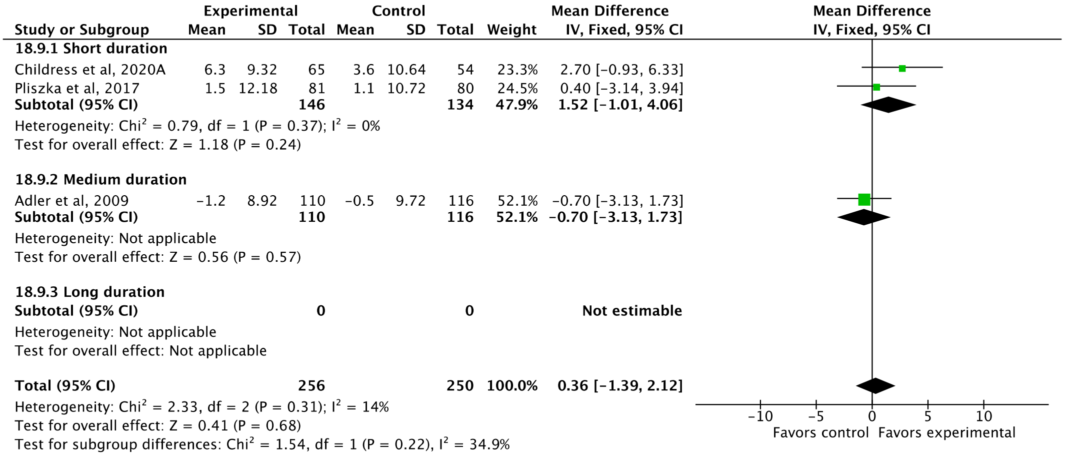

Figure legend: 95%CI, 95% confidence interval; IV, inverse variance; SD, standard deviation. No study encompassing long duration was included in the meta-analysis.

eFigure 122. Forest plot showing the risk ratio of **all adverse events** between control and experimental groups in participants using **medium** dose **lisdexamfetamine** subdivided by duration of use.

Study or Subgroup	Experimental Events	Experimental Total	Control Events	Control Total	Weight	Risk Ratio M-H, Fixed, 95% CI
**16.8.1 Short duration**						
Ermer et al, 2019	20	26	1	6	0.6%	4.62 [0.76, 27.96]
Findling et al, 2011	160	232	45	77	25.7%	1.18 [0.96, 1.45]
Martin et al, 2014B - LDX	12	18	8	17	3.1%	1.42 [0.78, 2.58]
**Subtotal (95% CI)**		**276**		**100**	**29.4%**	**1.28 [1.05, 1.56]**
Total events	192		54			
Heterogeneity: Chi² = 2.63, df = 2 (P = 0.27); I² = 24%						
Test for overall effect: Z = 2.40 (P = 0.02)						
**16.8.2 Medium duration**						
Adler et al, 2013	62	79	47	80	17.7%	1.34 [1.08, 1.66]
Coghill et al, 2013 - LDX	80	111	63	110	24.0%	1.26 [1.03, 1.53]
McElroy et al, 2015	166	196	37	63	21.3%	1.44 [1.16, 1.79]
**Subtotal (95% CI)**		**386**		**253**	**63.0%**	**1.34 [1.19, 1.52]**
Total events	308		147			
Heterogeneity: Chi² = 0.83, df = 2 (P = 0.66); I² = 0%						
Test for overall effect: Z = 4.75 (P < 0.00001)						
**16.8.3 Long duration**						
Coghill et al, 2014	31	78	20	79	7.5%	1.57 [0.98, 2.50]
**Subtotal (95% CI)**		**78**		**79**	**7.5%**	**1.57 [0.98, 2.50]**
Total events	31		20			
Heterogeneity: Not applicable						
Test for overall effect: Z = 1.89 (P = 0.06)						
**Total (95% CI)**		**740**		**432**	**100.0%**	**1.34 [1.21, 1.49]**
Total events	531		221			
Heterogeneity: Chi² = 4.56, df = 6 (P = 0.60); I² = 0%						
Test for overall effect: Z = 5.57 (P < 0.00001)						
Test for subgroup differences: Chi² = 0.66, df = 2 (P = 0.72), I² = 0%						

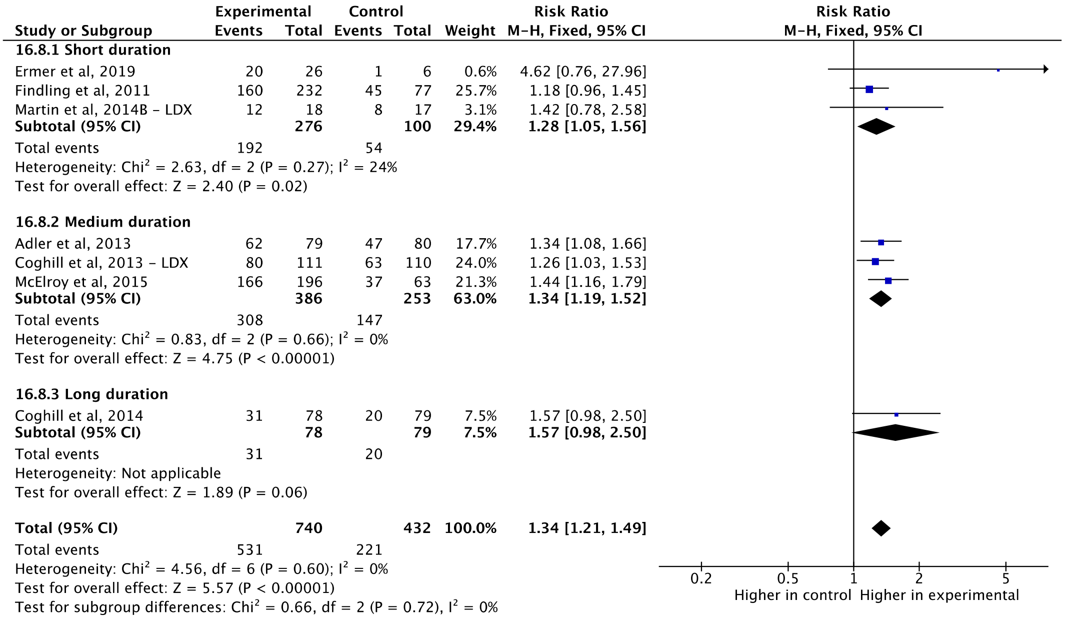

Figure legend: 95%CI, 95% confidence interval; LDX, lisdexamfetamine; M-H, Mantel–Haenszel.

eFigure 123. Forest plot showing the risk ratio of **anxiety** between control and experimental groups in participants using **medium** dose **lisdexamfetamine** subdivided by duration of use.

Study or Subgroup	Experimental Events	Experimental Total	Control Events	Control Total	Weight	Risk Ratio M-H, Random, 95% CI
**16.4.1 Short duration**						
**Subtotal (95% CI)**		**0**		**0**		**Not estimable**
Total events	0		0			
Heterogeneity: Not applicable						
Test for overall effect: Not applicable						
**16.4.2 Medium duration**						
McElroy et al, 2015	9	196	5	63	55.9%	0.58 [0.20, 1.66]
**Subtotal (95% CI)**		**196**		**63**	**55.9%**	**0.58 [0.20, 1.66]**
Total events	9		5			
Heterogeneity: Not applicable						
Test for overall effect: Z = 1.02 (P = 0.31)						
**16.4.3 Long duration**						
Mooney et al, 2015	7	22	1	21	44.1%	6.68 [0.90, 49.78]
**Subtotal (95% CI)**		**22**		**21**	**44.1%**	**6.68 [0.90, 49.78]**
Total events	7		1			
Heterogeneity: Not applicable						
Test for overall effect: Z = 1.85 (P = 0.06)						
**Total (95% CI)**		**218**		**84**	**100.0%**	**1.70 [0.14, 20.21]**
Total events	16		6			
Heterogeneity: $Tau^2 = 2.56$; $Chi^2 = 4.82$, df = 1 (P = 0.03); $I^2 = 79\%$						
Test for overall effect: Z = 0.42 (P = 0.67)						
Test for subgroup differences: $Chi^2 = 4.47$, df = 1 (P = 0.03), $I^2 = 77.6\%$						

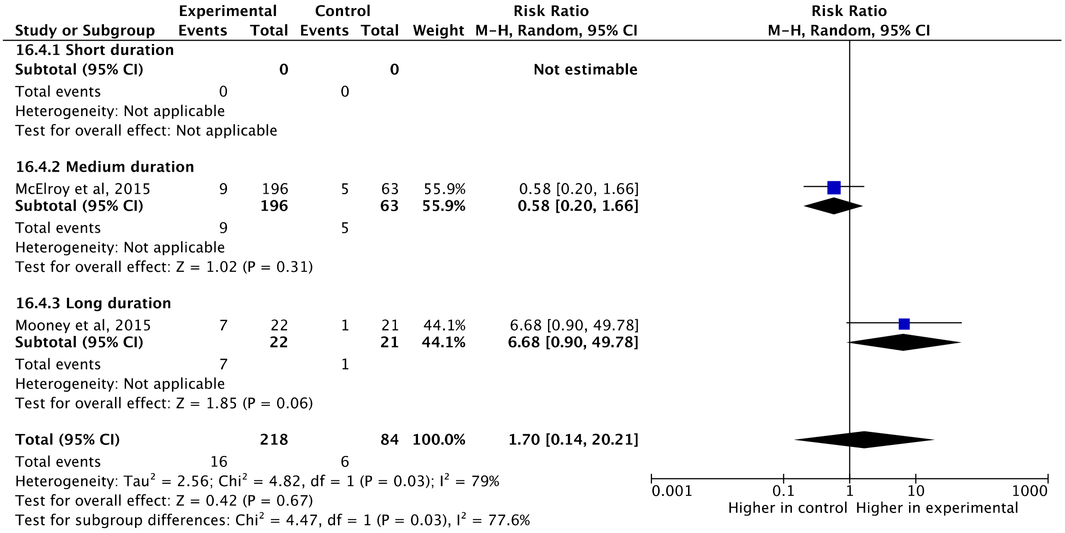

Figure legend: 95%CI, 95% confidence interval; LDX, lisdexamfetamine; M-H, Mantel–Haenszel. No study encompassing short duration was included in the meta-analysis.

eFigure 124. Forest plot showing the risk ratio of **decreased appetite** between control and experimental groups in participants using **medium** dose **lisdexamfetamine** subdivided by duration of use.

Study or Subgroup	Experimental Events	Experimental Total	Control Events	Control Total	Weight	Risk Ratio M-H, Random, 95% CI
**16.1.1 Short duration**						
Findling et al, 2011	79	232	2	77	18.5%	13.11 [3.30, 52.08]
Martin et al, 2014B - LDX	3	18	1	17	11.8%	2.83 [0.33, 24.66]
**Subtotal (95% CI)**		**250**		**94**	**30.2%**	**7.55 [1.67, 34.24]**
Total events	82		3			
Heterogeneity: $Tau^2 = 0.43$; $Chi^2 = 1.51$, df = 1 (P = 0.22); $I^2 = 34\%$						
Test for overall effect: Z = 2.62 (P = 0.009)						
**16.1.2 Medium duration**						
Coghill et al, 2013 - LDX	28	111	3	110	20.9%	9.25 [2.90, 29.54]
McElroy et al, 2015	42	196	4	63	22.9%	3.38 [1.26, 9.04]
**Subtotal (95% CI)**		**307**		**173**	**43.8%**	**5.32 [1.99, 14.26]**
Total events	70		7			
Heterogeneity: $Tau^2 = 0.21$; $Chi^2 = 1.69$, df = 1 (P = 0.19); $I^2 = 41\%$						
Test for overall effect: Z = 3.32 (P = 0.0009)						
**16.1.3 Long duration**						
Mooney et al, 2015	12	22	7	21	26.0%	1.64 [0.80, 3.35]
**Subtotal (95% CI)**		**22**		**21**	**26.0%**	**1.64 [0.80, 3.35]**
Total events	12		7			
Heterogeneity: Not applicable						
Test for overall effect: Z = 1.35 (P = 0.18)						
**Total (95% CI)**		**579**		**288**	**100.0%**	**4.35 [1.69, 11.20]**
Total events	164		17			
Heterogeneity: $Tau^2 = 0.76$; $Chi^2 = 13.34$, df = 4 (P = 0.010); $I^2 = 70\%$						
Test for overall effect: Z = 3.04 (P = 0.002)						
Test for subgroup differences: $Chi^2 = 5.45$, df = 2 (P = 0.07), $I^2 = 63.3\%$						

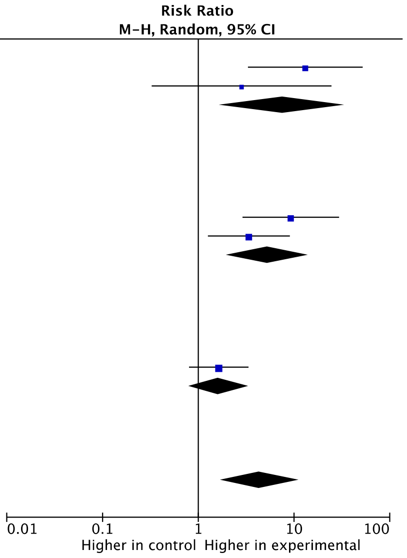

Figure legend: 95%CI, 95% confidence interval; LDX, lisdexamfetamine; M-H, Mantel–Haenszel.

eFigure 125. Forest plot showing the risk ratio of **dry mouth** between control and experimental groups in participants using **medium** dose **lisdexamfetamine** subdivided by duration of use.

Study or Subgroup	Experimental Events	Experimental Total	Control Events	Control Total	Weight	Risk Ratio M-H, Random, 95% CI
**16.6.1 Short duration**						
Findling et al, 2011	10	232	1	77	9.7%	3.32 [0.43, 25.51]
Martin et al, 2014B - LDX	6	18	3	17	27.3%	1.89 [0.56, 6.38]
**Subtotal (95% CI)**		**250**		**94**	**37.0%**	**2.19 [0.77, 6.23]**
Total events	16		4			
Heterogeneity: Tau² = 0.00; Chi² = 0.24, df = 1 (P = 0.63); I² = 0%						
Test for overall effect: Z = 1.47 (P = 0.14)						
**16.6.2 Medium duration**						
McElroy et al, 2015	71	196	5	63	54.5%	4.56 [1.93, 10.80]
Wigal et al, 2010 - LDX	4	115	1	117	8.5%	4.07 [0.46, 35.86]
**Subtotal (95% CI)**		**311**		**180**	**63.0%**	**4.49 [2.02, 10.01]**
Total events	75		6			
Heterogeneity: Tau² = 0.00; Chi² = 0.01, df = 1 (P = 0.92); I² = 0%						
Test for overall effect: Z = 3.68 (P = 0.0002)						
**16.6.3 Long duration**						
**Subtotal (95% CI)**		**0**		**0**		**Not estimable**
Total events	0		0			
Heterogeneity: Not applicable						
Test for overall effect: Not applicable						
**Total (95% CI)**		**561**		**274**	**100.0%**	**3.44 [1.82, 6.50]**
Total events	91		10			
Heterogeneity: Tau² = 0.00; Chi² = 1.44, df = 3 (P = 0.70); I² = 0%						
Test for overall effect: Z = 3.81 (P = 0.0001)						
Test for subgroup differences: Chi² = 1.14, df = 1 (P = 0.28), I² = 12.6%						

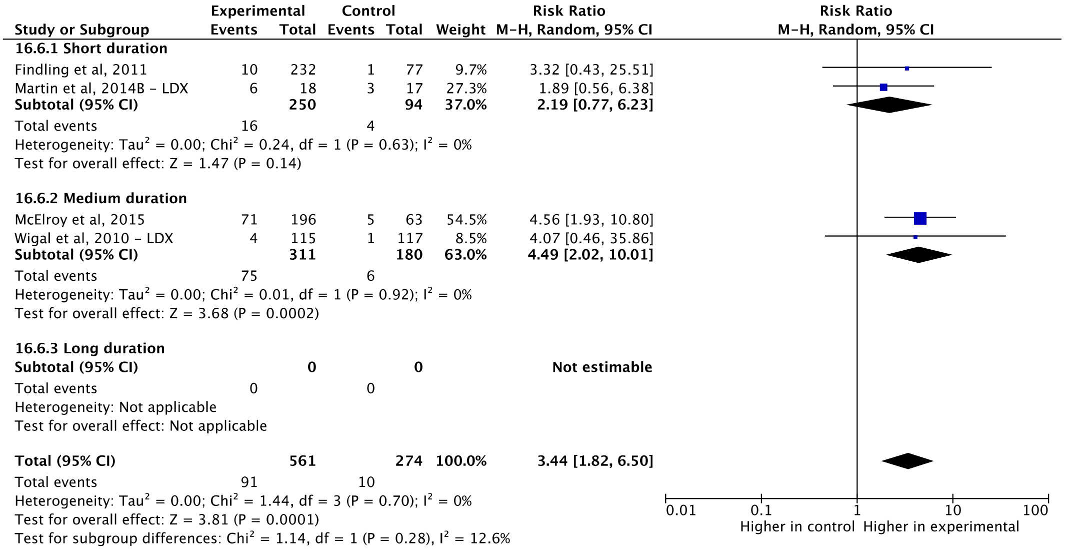

Figure legend: 95%CI, 95% confidence interval; LDX, lisdexamfetamine; M-H, Mantel–Haenszel. No study encompassing long duration was included in the meta-analysis.

eFigure 126. Forest plot showing the risk ratio of **headache** between control and experimental groups in participants using **medium** dose **lisdexamfetamine** subdivided by duration of use.

Study or Subgroup	Experimental Events	Experimental Total	Control Events	Control Total	Weight	Risk Ratio M-H, Fixed, 95% CI
**16.2.1 Short duration**						
Findling et al, 2011	34	232	10	77	25.3%	1.13 [0.59, 2.18]
Martin et al, 2014B - LDX	1	18	3	17	5.2%	0.31 [0.04, 2.74]
**Subtotal (95% CI)**		**250**		**94**	**30.5%**	**0.99 [0.53, 1.84]**
Total events	35		13			
Heterogeneity: Chi² = 1.23, df = 1 (P = 0.27); I² = 19%						
Test for overall effect: Z = 0.03 (P = 0.97)						
**16.2.2 Medium duration**						
Coghill et al, 2013 - LDX	16	111	22	110	37.3%	0.72 [0.40, 1.30]
McElroy et al, 2015	23	196	6	63	15.3%	1.23 [0.53, 2.89]
Wigal et al, 2010 - LDX	2	115	3	117	5.0%	0.68 [0.12, 3.98]
**Subtotal (95% CI)**		**422**		**290**	**57.6%**	**0.85 [0.54, 1.36]**
Total events	41		31			
Heterogeneity: Chi² = 1.10, df = 2 (P = 0.58); I² = 0%						
Test for overall effect: Z = 0.67 (P = 0.50)						
**16.2.3 Long duration**						
Coghill et al, 2014	6	78	5	79	8.4%	1.22 [0.39, 3.82]
Mooney et al, 2015	10	22	2	21	3.5%	4.77 [1.18, 19.27]
**Subtotal (95% CI)**		**100**		**100**	**11.8%**	**2.25 [0.97, 5.22]**
Total events	16		7			
Heterogeneity: Chi² = 2.23, df = 1 (P = 0.14); I² = 55%						
Test for overall effect: Z = 1.90 (P = 0.06)						
**Total (95% CI)**		**772**		**484**	**100.0%**	**1.06 [0.76, 1.48]**
Total events	92		51			
Heterogeneity: Chi² = 7.79, df = 6 (P = 0.25); I² = 23%						
Test for overall effect: Z = 0.34 (P = 0.73)						
Test for subgroup differences: Chi² = 3.98, df = 2 (P = 0.14), I² = 49.7%						

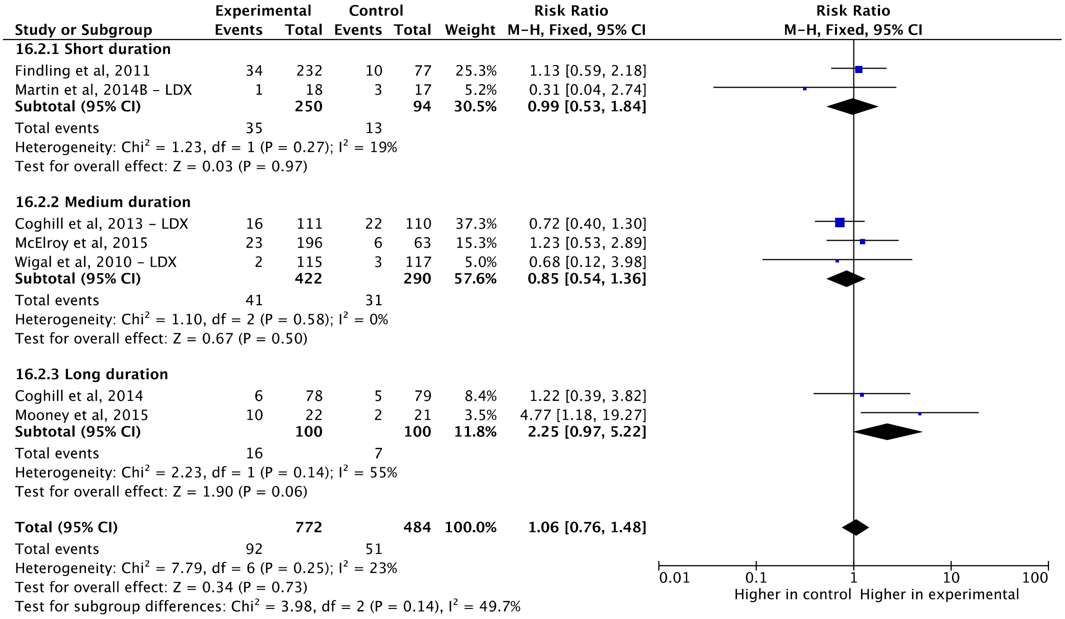

Figure legend: 95%CI, 95% confidence interval; LDX, lisdexamfetamine; M-H, Mantel–Haenszel.

eFigure 127. Forest plot showing the risk ratio of **insomnia** between control and experimental groups in participants using **medium** dose **lisdexamfetamine** subdivided by duration of use.

	Experimental		Control			Risk Ratio
**Study or Subgroup**	**Events**	**Total**	**Events**	**Total**	**Weight**	**M-H, Fixed, 95% CI**
**16.3.1 Short duration**						
Findling et al, 2011	26	232	3	77	28.0%	2.88 [0.90, 9.24]
**Subtotal (95% CI)**		**232**		**77**	**28.0%**	**2.88 [0.90, 9.24]**
Total events	26		3			
Heterogeneity: Not applicable						
Test for overall effect: Z = 1.77 (P = 0.08)						
**16.3.2 Medium duration**						
McElroy et al, 2015	26	196	5	63	47.0%	1.67 [0.67, 4.17]
Wigal et al, 2010 - LDX	3	115	2	117	12.3%	1.53 [0.26, 8.96]
**Subtotal (95% CI)**		**311**		**180**	**59.3%**	**1.64 [0.73, 3.70]**
Total events	29		7			
Heterogeneity: $Chi^2 = 0.01$, df = 1 (P = 0.93); $I^2 = 0\%$						
Test for overall effect: Z = 1.20 (P = 0.23)						
**16.3.3 Long duration**						
Mooney et al, 2015	7	22	2	21	12.7%	3.34 [0.78, 14.29]
**Subtotal (95% CI)**		**22**		**21**	**12.7%**	**3.34 [0.78, 14.29]**
Total events	7		2			
Heterogeneity: Not applicable						
Test for overall effect: Z = 1.63 (P = 0.10)						
**Total (95% CI)**		**565**		**278**	**100.0%**	**2.20 [1.21, 4.03]**
Total events	62		12			
Heterogeneity: $Chi^2 = 1.03$, df = 3 (P = 0.79); $I^2 = 0\%$						
Test for overall effect: Z = 2.57 (P = 0.01)						
Test for subgroup differences: $Chi^2 = 1.02$, df = 2 (P = 0.60), $I^2 = 0\%$						

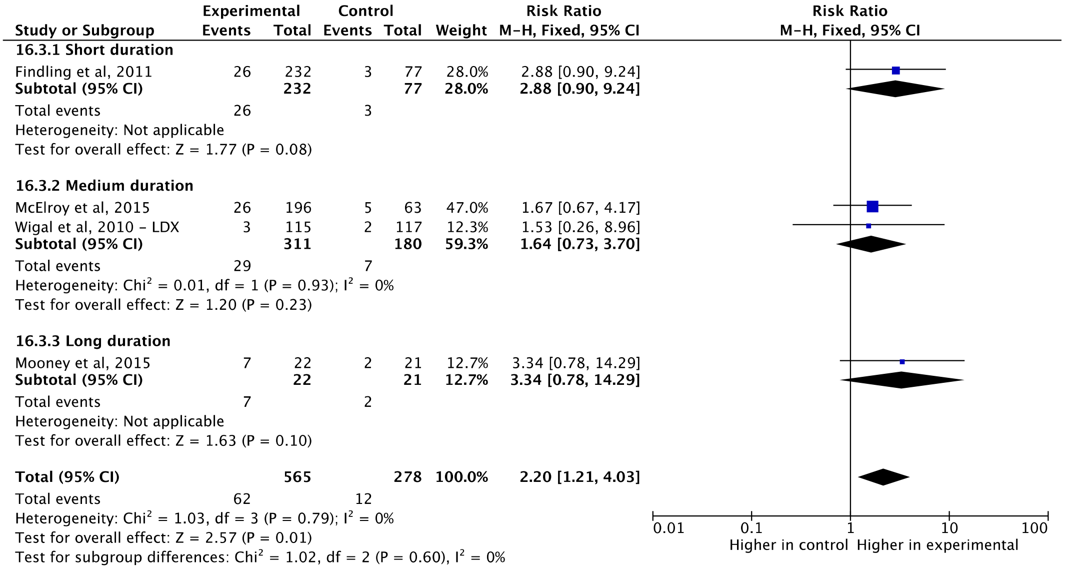

Figure legend: 95%CI, 95% confidence interval; LDX, lisdexamfetamine; M-H, Mantel–Haenszel.

eFigure 128. Forest plot showing the risk ratio of **irritability** between control and experimental groups in participants using **medium** dose **lisdexamfetamine** subdivided by duration of use.

Study or Subgroup	Experimental Events	Experimental Total	Control Events	Control Total	Weight	Risk Ratio M-H, Fixed, 95% CI
**16.7.1 Short duration**						
Findling et al, 2011	16	232	3	77	42.7%	1.77 [0.53, 5.91]
**Subtotal (95% CI)**		**232**		**77**	**42.7%**	**1.77 [0.53, 5.91]**
Total events	16		3			
Heterogeneity: Not applicable						
Test for overall effect: Z = 0.93 (P = 0.35)						
**16.7.2 Medium duration**						
McElroy et al, 2015	11	196	4	63	57.3%	0.88 [0.29, 2.68]
**Subtotal (95% CI)**		**196**		**63**	**57.3%**	**0.88 [0.29, 2.68]**
Total events	11		4			
Heterogeneity: Not applicable						
Test for overall effect: Z = 0.22 (P = 0.83)						
**16.7.3 Long duration**						
**Subtotal (95% CI)**		**0**		**0**		**Not estimable**
Total events	0		0			
Heterogeneity: Not applicable						
Test for overall effect: Not applicable						
**Total (95% CI)**		**428**		**140**	**100.0%**	**1.26 [0.56, 2.83]**
Total events	27		7			
Heterogeneity: $Chi^2 = 0.70$, df = 1 (P = 0.40); $I^2 = 0\%$						
Test for overall effect: Z = 0.56 (P = 0.57)						
Test for subgroup differences: $Chi^2 = 0.69$, df = 1 (P = 0.41), $I^2 = 0\%$						

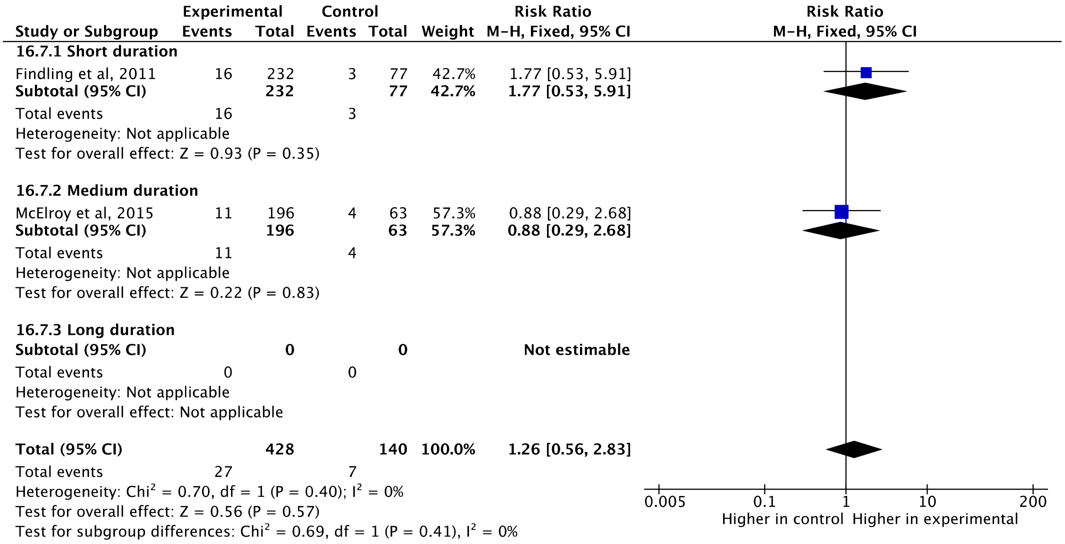

Figure legend: 95%CI, 95% confidence interval; LDX, lisdexamfetamine; M-H, Mantel–Haenszel. No study encompassing long duration was included in the meta-analysis.

eFigure 129. Forest plot showing the risk ratio of **nausea** between control and experimental groups in participants using **medium** dose **lisdexamfetamine** subdivided by duration of use.

Study or Subgroup	Experimental Events	Experimental Total	Control Events	Control Total	Weight	Risk Ratio M-H, Fixed, 95% CI
**16.5.1 Short duration**						
Findling et al, 2011	9	232	2	77	38.5%	1.49 [0.33, 6.76]
**Subtotal (95% CI)**		**232**		**77**	**38.5%**	**1.49 [0.33, 6.76]**
Total events	9		2			
Heterogeneity: Not applicable						
Test for overall effect: Z = 0.52 (P = 0.60)						
**16.5.2 Medium duration**						
Coghill et al, 2013 - LDX	12	111	3	110	38.7%	3.96 [1.15, 13.66]
McElroy et al, 2015	15	196	0	63	9.7%	10.07 [0.61, 165.95]
**Subtotal (95% CI)**		**307**		**173**	**48.3%**	**5.19 [1.62, 16.65]**
Total events	27		3			
Heterogeneity: $Chi^2 = 0.40$, df = 1 (P = 0.53); $I^2 = 0\%$						
Test for overall effect: Z = 2.77 (P = 0.006)						
**16.5.3 Long duration**						
Mooney et al, 2015	5	22	1	21	13.1%	4.77 [0.61, 37.52]
**Subtotal (95% CI)**		**22**		**21**	**13.1%**	**4.77 [0.61, 37.52]**
Total events	5		1			
Heterogeneity: Not applicable						
Test for overall effect: Z = 1.49 (P = 0.14)						
**Total (95% CI)**		**561**		**271**	**100.0%**	**3.71 [1.63, 8.44]**
Total events	41		6			
Heterogeneity: $Chi^2 = 1.95$, df = 3 (P = 0.58); $I^2 = 0\%$						
Test for overall effect: Z = 3.13 (P = 0.002)						
Test for subgroup differences: $Chi^2 = 1.75$, df = 2 (P = 0.42), $I^2 = 0\%$						

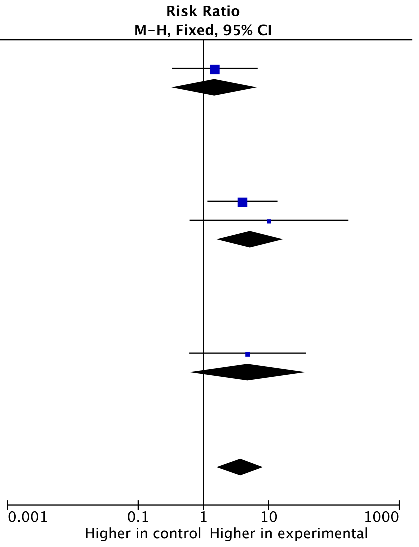

Figure legend: 95%CI, 95% confidence interval; LDX, lisdexamfetamine; M-H, Mantel–Haenszel..

eFigure 130. Forest plot showing the mean differences in **heart rate** between control and experimental groups in participants using **medium** dose **lisdexamfetamine** subdivided by duration of use.

Study or Subgroup	Experimental Mean	Experimental SD	Experimental Total	Control Mean	Control SD	Control Total	Weight	Mean Difference IV, Fixed, 95% CI
**16.11.1 Short duration**								
Findling et al, 2011	5.4	1.27	78	0.8	1.36	77	94.0%	4.60 [4.19, 5.01]
**Subtotal (95% CI)**			**78**			**77**	**94.0%**	**4.60 [4.19, 5.01]**
Heterogeneity: Not applicable								
Test for overall effect: Z = 21.76 (P < 0.00001)								
**16.11.2 Medium duration**								
Coghill et al, 2013 - LDX	5.7	15.3	222	−1.1	9.6	110	2.2%	6.80 [4.10, 9.50]
Coghill et al, 2014	6.1	12.59	78	2.3	9.29	79	1.3%	3.80 [0.34, 7.26]
McElroy et al, 2015	3.8	11.57	196	1	8.1	63	2.4%	2.80 [0.23, 5.37]
**Subtotal (95% CI)**			**496**			**252**	**6.0%**	**4.50 [2.86, 6.14]**
Heterogeneity: $Chi^2 = 4.63$, df = 2 (P = 0.10); $I^2 = 57\%$								
Test for overall effect: Z = 5.38 (P < 0.00001)								
**16.11.3 Long duration**								
**Subtotal (95% CI)**			**0**			**0**		**Not estimable**
Heterogeneity: Not applicable								
Test for overall effect: Not applicable								
**Total (95% CI)**			**574**			**329**	**100.0%**	**4.59 [4.19, 5.00]**
Heterogeneity: $Chi^2 = 4.64$, df = 3 (P = 0.20); $I^2 = 35\%$								
Test for overall effect: Z = 22.41 (P < 0.00001)								
Test for subgroup differences: $Chi^2 = 0.01$, df = 1 (P = 0.91), $I^2 = 0\%$								

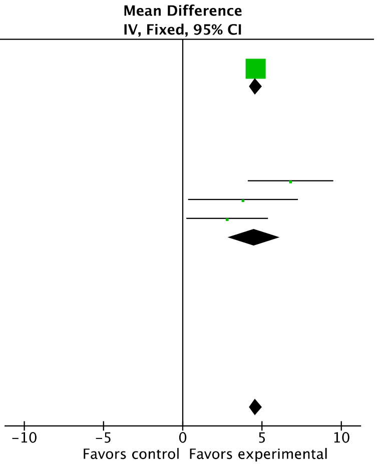

Figure legend: 95%CI, 95% confidence interval; IV, inverse variance; SD, standard deviation. No study encompassing long duration was included in the meta-analysis.

eFigure 131. Forest plot showing the mean differences in **diastolic blood pressure** between control and experimental groups in participants using **medium** dose **lisdexamfetamine** subdivided by duration of use.

Study or Subgroup	Experimental Mean	Experimental SD	Experimental Total	Control Mean	Control SD	Control Total	Weight	Mean Difference IV, Random, 95% CI
**16.10.1 Short duration**								
Findling et al, 2011	3.4	0.8	78	0.5	0.97	77	30.5%	2.90 [2.62, 3.18]
**Subtotal (95% CI)**			**78**			**77**	**30.5%**	**2.90 [2.62, 3.18]**
Heterogeneity: Not applicable								
Test for overall effect: Z = 20.29 (P < 0.00001)								
**16.10.2 Medium duration**								
Coghill et al, 2013 - LDX	0.2	9.6	222	1.2	8.7	110	24.6%	−1.00 [−3.06, 1.06]
Coghill et al, 2014	1.4	11.27	78	−0.1	8.12	79	19.8%	1.50 [−1.58, 4.58]
McElroy et al, 2015	−0.7	7.32	196	−0.5	6.8	63	25.0%	−0.20 [−2.17, 1.77]
**Subtotal (95% CI)**			**496**			**252**	**69.5%**	**−0.22 [−1.51, 1.08]**
Heterogeneity: $Tau^2 = 0.00$; $Chi^2 = 1.75$, df = 2 (P = 0.42); $I^2 = 0\%$								
Test for overall effect: Z = 0.33 (P = 0.74)								
**16.10.3 Long duration**								
**Subtotal (95% CI)**			**0**			**0**		**Not estimable**
Heterogeneity: Not applicable								
Test for overall effect: Not applicable								
**Total (95% CI)**			**574**			**329**	**100.0%**	**0.89 [−1.42, 3.19]**
Heterogeneity: $Tau^2 = 4.50$; $Chi^2 = 23.12$, df = 3 (P < 0.0001); $I^2 = 87\%$								
Test for overall effect: Z = 0.75 (P = 0.45)								
Test for subgroup differences: $Chi^2 = 21.36$, df = 1 (P < 0.00001), $I^2 = 95.3\%$								

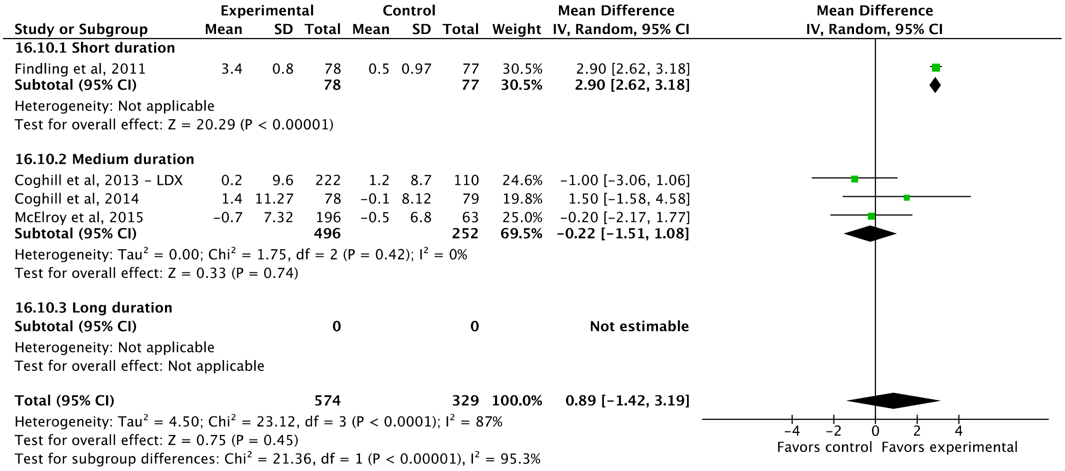

Figure legend: 95%CI, 95% confidence interval; IV, inverse variance; SD, standard deviation. No study encompassing long duration was included in the meta-analysis.

eFigure 132. Forest plot showing the mean differences in **systolic blood pressure** between control and experimental groups in participants using **medium** dose **lisdexamfetamine** subdivided by duration of use.

	Experimental			Control				Mean Difference
**Study or Subgroup**	**Mean**	**SD**	**Total**	**Mean**	**SD**	**Total**	**Weight**	**IV, Fixed, 95% CI**
**16.9.1 Short duration**								
Findling et al, 2011	1.7	1.21	78	2.2	1.04	77	94.6%	−0.50 [−0.86, −0.14]
**Subtotal (95% CI)**			**78**			**77**	**94.6%**	**−0.50 [−0.86, −0.14]**
Heterogeneity: Not applicable								
Test for overall effect: Z = 2.76 (P = 0.006)								
**16.9.2 Medium duration**								
Coghill et al, 2013 - LDX	1	9.8	222	1	9.6	110	2.4%	0.00 [−2.21, 2.21]
Coghill et al, 2014	1.5	11.79	78	−0.6	9.66	79	1.0%	2.10 [−1.27, 5.47]
McElroy et al, 2015	0.1	9.85	196	−1.5	8.41	63	1.9%	1.60 [−0.89, 4.09]
**Subtotal (95% CI)**			**496**			**252**	**5.4%**	**0.97 [−0.51, 2.46]**
Heterogeneity: $Chi^2 = 1.42$, df = 2 (P = 0.49); $I^2 = 0\%$								
Test for overall effect: Z = 1.29 (P = 0.20)								
**16.9.3 Long duration**								
**Subtotal (95% CI)**			**0**			**0**		**Not estimable**
Heterogeneity: Not applicable								
Test for overall effect: Not applicable								
**Total (95% CI)**			**574**			**329**	**100.0%**	**−0.42 [−0.77, −0.07]**
Heterogeneity: $Chi^2 = 5.00$, df = 3 (P = 0.17); $I^2 = 40\%$								
Test for overall effect: Z = 2.39 (P = 0.02)								
Test for subgroup differences: $Chi^2 = 3.58$, df = 1 (P = 0.06), $I^2 = 72.1\%$								

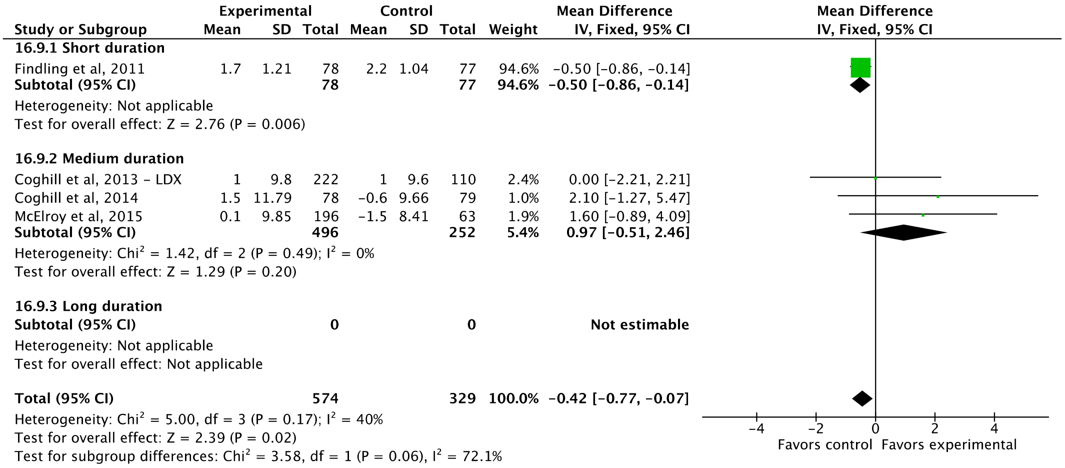

Figure legend: 95%CI, 95% confidence interval; IV, inverse variance; SD, standard deviation. No study encompassing long duration was included in the meta-analysis.

eFigure 133. Forest plot showing the risk ratio of **all adverse events** between control and experimental groups in participants using **high** dose **lisdexamfetamine** subdivided by duration of use.

Study or Subgroup	Experimental Events	Experimental Total	Control Events	Control Total	Weight	Risk Difference M-H, Random, 95% CI
**19.8.1 Short duration**						
Jasinski et al, 2009	15	37	6	36	35.4%	0.24 [0.04, 0.44]
Martin et al, 2014A	18	24	2	7	25.5%	0.46 [0.09, 0.84]
**Subtotal (95% CI)**		**61**		**43**	**60.8%**	**0.29 [0.10, 0.48]**
Total events	33		8			
Heterogeneity: $Tau^2 = 0.00$; $Chi^2 = 1.08$, df = 1 (P = 0.30); $I^2 = 7\%$						
Test for overall effect: Z = 3.04 (P = 0.002)						
**19.8.2 Medium duration**						
Wigal et al, 2010 - LDX	32	115	42	117	39.2%	−0.08 [−0.20, 0.04]
**Subtotal (95% CI)**		**115**		**117**	**39.2%**	**−0.08 [−0.20, 0.04]**
Total events	32		42			
Heterogeneity: Not applicable						
Test for overall effect: Z = 1.32 (P = 0.19)						
**19.8.3 Long duration**						
**Subtotal (95% CI)**		**0**		**0**		**Not estimable**
Total events	0		0			
Heterogeneity: Not applicable						
Test for overall effect: Not applicable						
**Total (95% CI)**		**176**		**160**	**100.0%**	**0.17 [−0.13, 0.48]**
Total events	65		50			
Heterogeneity: $Tau^2 = 0.06$; $Chi^2 = 12.62$, df = 2 (P = 0.002); $I^2 = 84\%$						
Test for overall effect: Z = 1.10 (P = 0.27)						
Test for subgroup differences: $Chi^2 = 10.75$, df = 1 (P = 0.001), $I^2 = 90.7\%$						

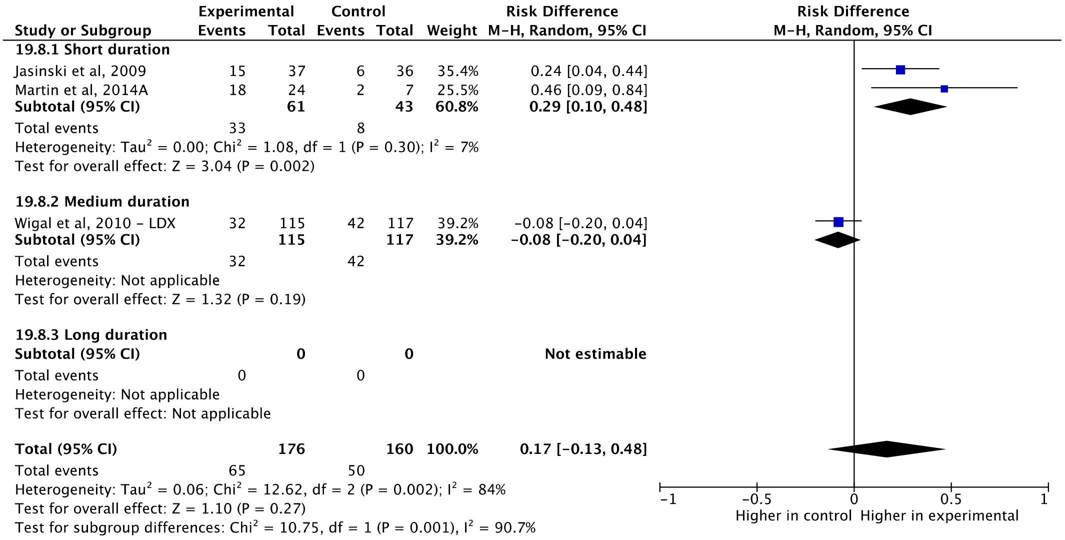

Figure legend: 95%CI, 95% confidence interval; LDX, lisdexamfetamine; M-H, Mantel–Haenszel. No study encompassing long duration was included in the meta-analysis.

eFigure 134. Forest plot showing the risk ratio of **all adverse events** between control and experimental groups in participants using **medium** dose **amphetamines** subdivided by duration of use.

Study or Subgroup	Experimental Events	Experimental Total	Control Events	Control Total	Weight	Risk Ratio M-H, Random, 95% CI
**17.8.1 Short duration**						
Brams et al, 2018	70	131	34	131	15.5%	2.06 [1.48, 2.87]
Childress et al, 2015	10	97	6	97	4.2%	1.67 [0.63, 4.41]
Childress et al, 2017	9	51	6	48	4.3%	1.41 [0.54, 3.67]
Cutler et al, 2022B	44	105	43	105	15.8%	1.02 [0.74, 1.41]
Martin et al, 2014B - AMPH	9	17	8	17	7.3%	1.13 [0.57, 2.21]
Weisler et al, 2017	104	182	19	89	12.8%	2.68 [1.76, 4.07]
**Subtotal (95% CI)**		**583**		**487**	**59.7%**	**1.60 [1.09, 2.36]**
Total events	246		116			
Heterogeneity: $Tau^2 = 0.15$; $Chi^2 = 17.07$, df = 5 (P = 0.004); $I^2 = 71\%$						
Test for overall effect: Z = 2.37 (P = 0.02)						
**17.8.2 Medium duration**						
Cutler et al, 2022A	54	62	35	65	18.4%	1.62 [1.27, 2.07]
Spencer et al, 2008	122	137	86	135	21.8%	1.40 [1.22, 1.61]
**Subtotal (95% CI)**		**199**		**200**	**40.3%**	**1.45 [1.28, 1.65]**
Total events	176		121			
Heterogeneity: $Tau^2 = 0.00$; $Chi^2 = 1.04$, df = 1 (P = 0.31); $I^2 = 4\%$						
Test for overall effect: Z = 5.80 (P < 0.00001)						
**17.8.3 Long duration**						
**Subtotal (95% CI)**		**0**		**0**		**Not estimable**
Total events	0		0			
Heterogeneity: Not applicable						
Test for overall effect: Not applicable						
**Total (95% CI)**		**782**		**687**	**100.0%**	**1.56 [1.26, 1.95]**
Total events	422		237			
Heterogeneity: $Tau^2 = 0.05$; $Chi^2 = 19.77$, df = 7 (P = 0.006); $I^2 = 65\%$						
Test for overall effect: Z = 4.02 (P < 0.0001)						
Test for subgroup differences: $Chi^2 = 0.22$, df = 1 (P = 0.64), $I^2 = 0\%$						

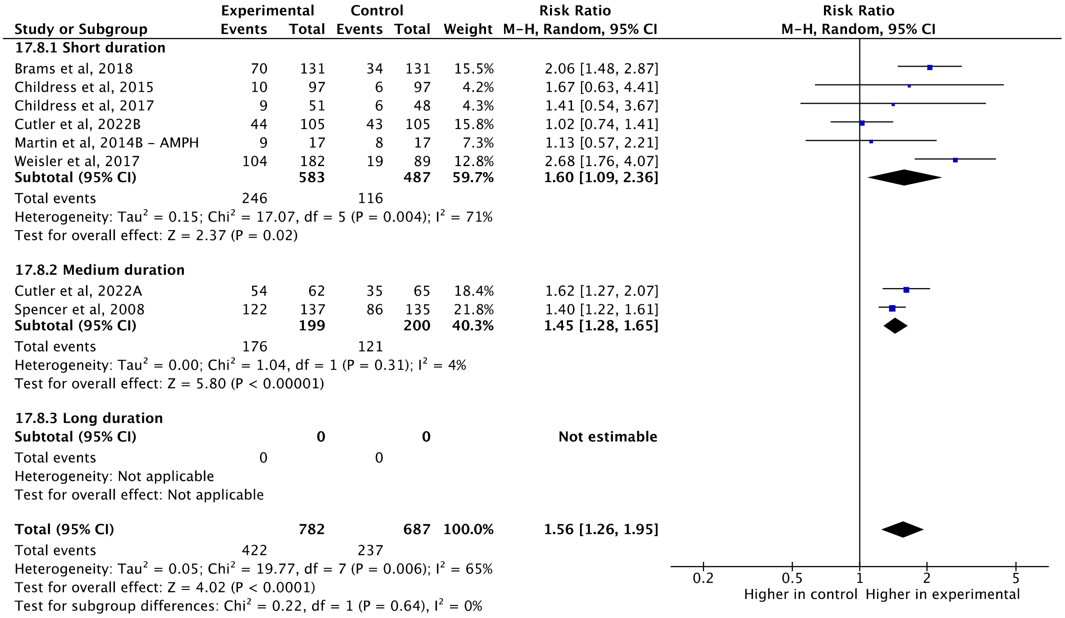

Figure legend: 95%CI, 95% confidence interval; AMPH, amphetamines; M-H, Mantel–Haenszel. No study encompassing long duration was included in the meta-analysis.

eFigure 135. Forest plot showing the risk ratio of **anxiety** between control and experimental groups in participants using **medium** dose **amphetamines** subdivided by duration of use.

	Experimental		Control			Risk Ratio
**Study or Subgroup**	**Events**	**Total**	**Events**	**Total**	**Weight**	**M-H, Fixed, 95% CI**
**17.4.1 Short duration**						
Weisler et al, 2017	10	182	1	89	4.2%	4.89 [0.64, 37.61]
**Subtotal (95% CI)**		**182**		**89**	**4.2%**	**4.89 [0.64, 37.61]**
Total events	10		1			
Heterogeneity: Not applicable						
Test for overall effect: Z = 1.52 (P = 0.13)						
**17.4.2 Medium duration**						
McCracken et al, 2003	11	48	10	49	30.7%	1.12 [0.53, 2.40]
McCracken et al, 2003 - XR	9	49	10	49	31.0%	0.90 [0.40, 2.02]
Ramtvedt et al, 2014 - AMPH	8	34	7	34	21.7%	1.14 [0.47, 2.80]
Spencer et al, 2008	9	137	4	135	12.5%	2.22 [0.70, 7.03]
**Subtotal (95% CI)**		**268**		**267**	**95.8%**	**1.20 [0.78, 1.85]**
Total events	37		31			
Heterogeneity: Chi² = 1.61, df = 3 (P = 0.66); I² = 0%						
Test for overall effect: Z = 0.82 (P = 0.41)						
**17.4.3 Long duration**						
**Subtotal (95% CI)**		**0**		**0**		**Not estimable**
Total events	0		0			
Heterogeneity: Not applicable						
Test for overall effect: Not applicable						
**Total (95% CI)**		**450**		**356**	**100.0%**	**1.35 [0.89, 2.06]**
Total events	47		32			
Heterogeneity: Chi² = 3.57, df = 4 (P = 0.47); I² = 0%						
Test for overall effect: Z = 1.40 (P = 0.16)						
Test for subgroup differences: Chi² = 1.75, df = 1 (P = 0.19), I² = 42.8%						

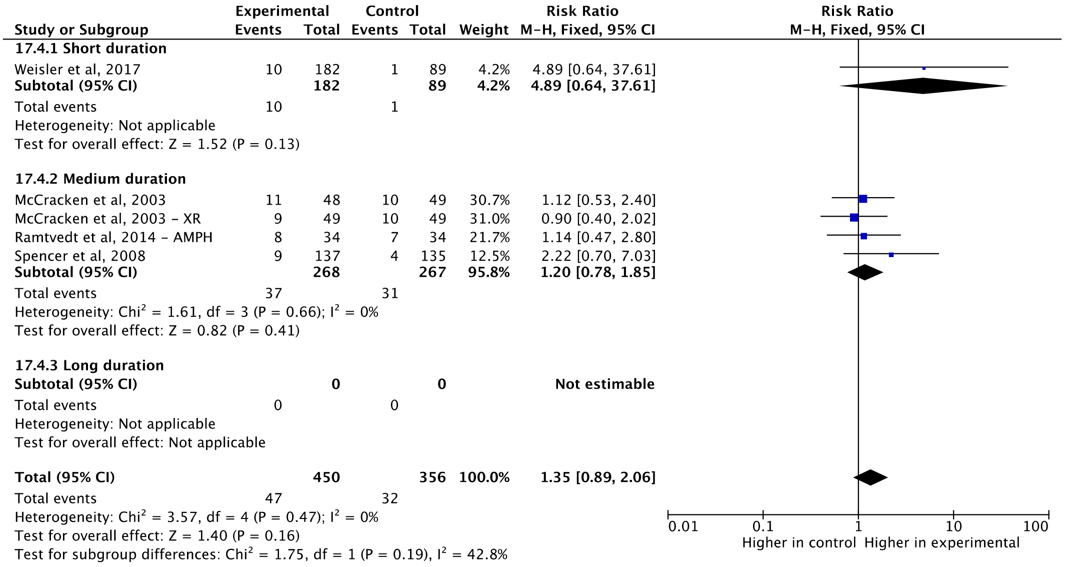

Figure legend: 95%CI, 95% confidence interval; AMPH, amphetamines; M-H, Mantel–Haenszel; XR, extended release. No study encompassing long duration was included in the meta-analysis.

eFigure 136. Forest plot showing the risk ratio of **decreased appetite** between control and experimental groups in participants using **medium** dose **amphetamines** subdivided by duration of use.

Study or Subgroup	Experimental Events	Experimental Total	Control Events	Control Total	Weight	Risk Ratio M-H, Random, 95% CI
**17.1.1 Short duration**						
Brams et al, 2018	40	132	9	131	12.5%	4.41 [2.23, 8.72]
Cutler et al, 2022B	13	105	2	105	6.7%	6.50 [1.50, 28.10]
Martin et al, 2014B - AMPH	4	17	1	17	4.2%	4.00 [0.50, 32.20]
Spencer et al, 2006	83	226	1	52	4.6%	19.10 [2.72, 134.04]
Weisler et al, 2017	45	182	4	89	9.9%	5.50 [2.04, 14.82]
**Subtotal (95% CI)**		**662**		**394**	**37.8%**	**5.31 [3.25, 8.69]**
Total events	185		17			
Heterogeneity: Tau² = 0.00; Chi² = 2.45, df = 4 (P = 0.65); I² = 0%						
Test for overall effect: Z = 6.65 (P < 0.00001)						
**17.1.2 Medium duration**						
Cutler et al, 2022A	30	62	14	65	13.8%	2.25 [1.32, 3.82]
McCracken et al, 2003	22	48	11	49	13.2%	2.04 [1.12, 3.74]
McCracken et al, 2003 - XR	27	49	11	49	13.4%	2.45 [1.38, 4.38]
Ramtvedt et al, 2014 - AMPH	24	34	17	34	14.8%	1.41 [0.95, 2.11]
Spencer et al, 2008	27	137	2	135	7.0%	13.30 [3.23, 54.84]
**Subtotal (95% CI)**		**330**		**332**	**62.2%**	**2.31 [1.43, 3.75]**
Total events	130		55			
Heterogeneity: Tau² = 0.19; Chi² = 12.67, df = 4 (P = 0.01); I² = 68%						
Test for overall effect: Z = 3.41 (P = 0.0007)						
**17.1.3 Long duration**						
**Subtotal (95% CI)**		**0**		**0**		**Not estimable**
Total events	0		0			
Heterogeneity: Not applicable						
Test for overall effect: Not applicable						
**Total (95% CI)**		**992**		**726**	**100.0%**	**3.42 [2.09, 5.59]**
Total events	315		72			
Heterogeneity: Tau² = 0.38; Chi² = 33.23, df = 9 (P = 0.0001); I² = 73%						
Test for overall effect: Z = 4.90 (P < 0.00001)						
Test for subgroup differences: Chi² = 5.60, df = 1 (P = 0.02), I² = 82.1%						

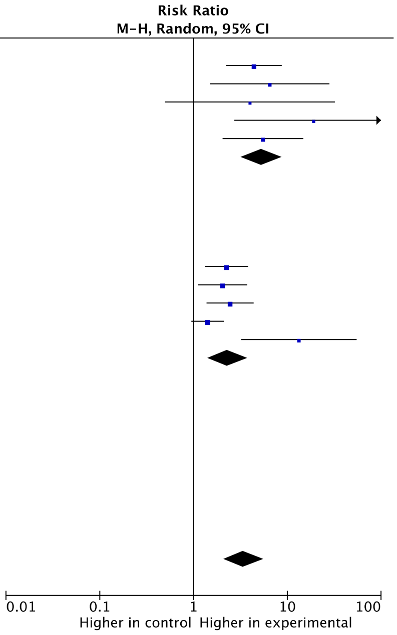

Figure legend: 95%CI, 95% confidence interval; AMPH, amphetamines; M-H, Mantel–Haenszel; XR, extended release. No study encompassing long duration was included in the meta-analysis.

eFigure 137. Forest plot showing the risk ratio of **dry mouth** between control and experimental groups in participants using **medium** dose **amphetamines** subdivided by duration of use.

Study or Subgroup	Experimental Events	Experimental Total	Control Events	Control Total	Weight	Risk Ratio M-H, Fixed, 95% CI
**17.6.1 Short duration**						
Martin et al, 2014B - AMPH	4	17	3	17	19.9%	1.33 [0.35, 5.08]
Weisler et al, 2017	33	182	3	89	26.8%	5.38 [1.70, 17.06]
**Subtotal (95% CI)**		**199**		**106**	**46.7%**	**3.65 [1.52, 8.75]**
Total events	37		6			
Heterogeneity: $Chi^2 = 2.61$, df = 1 (P = 0.11); $I^2 = 62\%$						
Test for overall effect: Z = 2.91 (P = 0.004)						
**17.6.2 Medium duration**						
Cutler et al, 2022A	12	62	1	65	6.5%	12.58 [1.69, 93.90]
Spencer et al, 2008	31	137	7	135	46.8%	4.36 [1.99, 9.57]
**Subtotal (95% CI)**		**199**		**200**	**53.3%**	**5.36 [2.60, 11.08]**
Total events	43		8			
Heterogeneity: $Chi^2 = 0.96$, df = 1 (P = 0.33); $I^2 = 0\%$						
Test for overall effect: Z = 4.53 (P < 0.00001)						
**17.6.3 Long duration**						
**Subtotal (95% CI)**		**0**		**0**		**Not estimable**
Total events	0		0			
Heterogeneity: Not applicable						
Test for overall effect: Not applicable						
**Total (95% CI)**		**398**		**306**	**100.0%**	**4.56 [2.62, 7.96]**
Total events	80		14			
Heterogeneity: $Chi^2 = 4.32$, df = 3 (P = 0.23); $I^2 = 31\%$						
Test for overall effect: Z = 5.35 (P < 0.00001)						
Test for subgroup differences: $Chi^2 = 0.44$, df = 1 (P = 0.51), $I^2 = 0\%$						

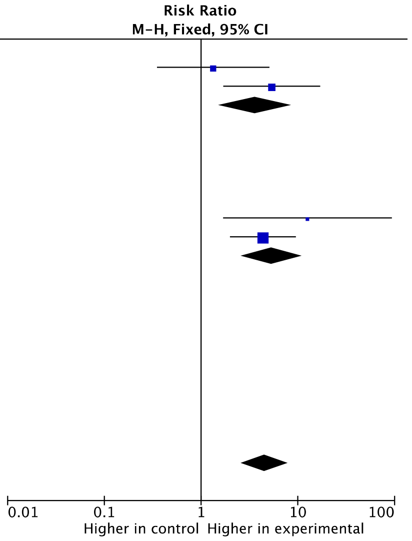

Figure legend: 95%CI, 95% confidence interval; AMPH, amphetamines; M-H, Mantel–Haenszel. No study encompassing long duration was included in the meta-analysis.

eFigure 138. Forest plot showing the risk ratio of **headache** between control and experimental groups in participants using **medium** dose **amphetamines** subdivided by duration of use.

Study or Subgroup	Experimental Events	Experimental Total	Control Events	Control Total	Weight	Risk Ratio M-H, Fixed, 95% CI
**17.2.1 Short duration**						
Brams et al, 2018	16	132	14	131	14.4%	1.13 [0.58, 2.23]
Childress et al, 2017	1	51	1	48	1.1%	0.94 [0.06, 14.63]
Cutler et al, 2022B	6	105	4	105	4.1%	1.50 [0.44, 5.16]
Martin et al, 2014B - AMPH	1	17	3	17	3.1%	0.33 [0.04, 2.89]
Spencer et al, 2006	38	226	12	54	19.8%	0.76 [0.42, 1.35]
Weisler et al, 2017	17	182	4	89	5.5%	2.08 [0.72, 5.99]
**Subtotal (95% CI)**		**713**		**444**	**47.9%**	**1.06 [0.73, 1.54]**
Total events	79		38			
Heterogeneity: $Chi^2 = 4.32$, df = 5 (P = 0.50); $I^2 = 0\%$						
Test for overall effect: Z = 0.32 (P = 0.75)						
**17.2.2 Medium duration**						
McCracken et al, 2003	12	48	12	49	12.1%	1.02 [0.51, 2.04]
McCracken et al, 2003 - XR	12	49	12	49	12.3%	1.00 [0.50, 2.00]
Ramtvedt et al, 2014 - AMPH	8	34	8	34	8.2%	1.00 [0.42, 2.36]
Spencer et al, 2008	25	137	19	135	19.6%	1.30 [0.75, 2.24]
**Subtotal (95% CI)**		**268**		**267**	**52.1%**	**1.12 [0.80, 1.56]**
Total events	57		51			
Heterogeneity: $Chi^2 = 0.51$, df = 3 (P = 0.92); $I^2 = 0\%$						
Test for overall effect: Z = 0.64 (P = 0.52)						
**17.2.3 Long duration**						
**Subtotal (95% CI)**		**0**		**0**		**Not estimable**
Total events	0		0			
Heterogeneity: Not applicable						
Test for overall effect: Not applicable						
**Total (95% CI)**		**981**		**711**	**100.0%**	**1.09 [0.85, 1.40]**
Total events	136		89			
Heterogeneity: $Chi^2 = 4.92$, df = 9 (P = 0.84); $I^2 = 0\%$						
Test for overall effect: Z = 0.68 (P = 0.50)						
Test for subgroup differences: $Chi^2 = 0.04$, df = 1 (P = 0.85), $I^2 = 0\%$						

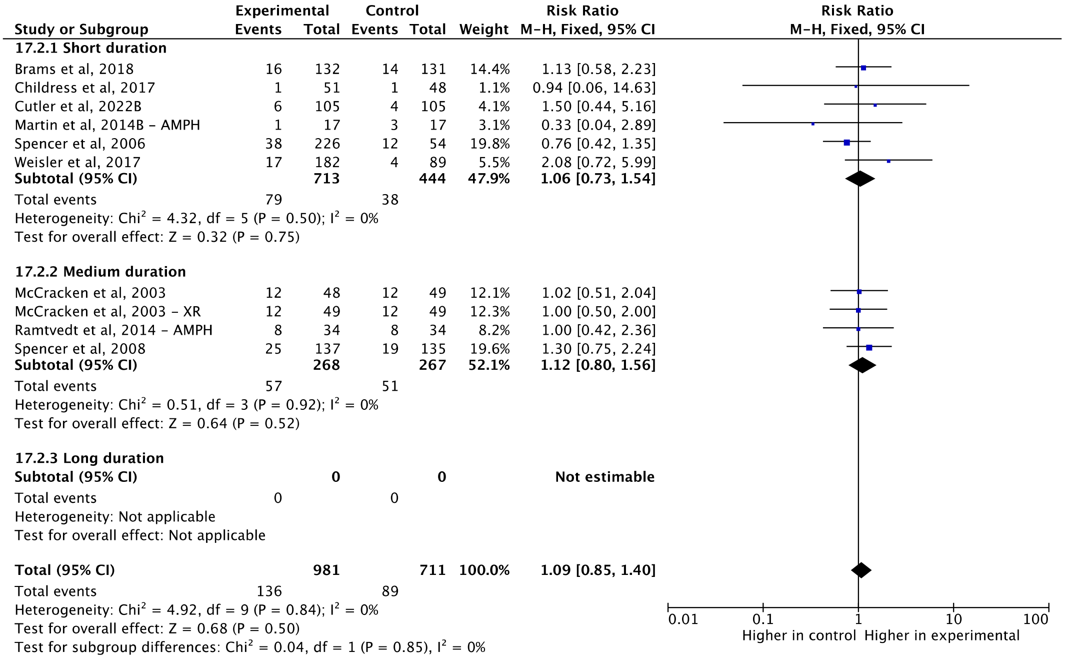

Figure legend: 95%CI, 95% confidence interval; AMPH, amphetamines; M-H, Mantel–Haenszel; XR, extended release. No study encompassing long duration was included in the meta-analysis.

eFigure 139. Forest plot showing the risk ratio of **insomnia** between control and experimental groups in participants using **medium** dose **amphetamines** subdivided by duration of use.

Study or Subgroup	Experimental Events	Experimental Total	Control Events	Control Total	Weight	Risk Ratio M-H, Fixed, 95% CI
**17.3.1 Short duration**						
Brams et al, 2018	22	132	3	131	4.5%	7.28 [2.23, 23.73]
Childress et al, 2017	1	51	1	48	1.5%	0.94 [0.06, 14.63]
Cutler et al, 2022B	6	105	5	105	7.4%	1.20 [0.38, 3.81]
Spencer et al, 2006	28	226	2	52	4.8%	3.22 [0.79, 13.10]
Weisler et al, 2017	22	182	1	89	2.0%	10.76 [1.47, 78.54]
**Subtotal (95% CI)**		**696**		**425**	**20.2%**	**3.95 [2.12, 7.35]**
Total events	79		12			
Heterogeneity: $Chi^2 = 7.21, df = 4 (P = 0.13); I^2 = 45\%$						
Test for overall effect: Z = 4.33 (P < 0.0001)						
**17.3.2 Medium duration**						
Cutler et al, 2022A	14	62	8	65	11.6%	1.83 [0.83, 4.07]
McCracken et al, 2003	17	48	10	49	14.7%	1.74 [0.89, 3.40]
McCracken et al, 2003 - XR	14	49	10	49	14.8%	1.40 [0.69, 2.84]
Ramtvedt et al, 2014 - AMPH	30	34	14	34	20.8%	2.14 [1.41, 3.26]
Spencer et al, 2008	40	137	12	135	17.9%	3.28 [1.80, 5.98]
**Subtotal (95% CI)**		**330**		**332**	**79.8%**	**2.14 [1.63, 2.82]**
Total events	115		54			
Heterogeneity: $Chi^2 = 3.86, df = 4 (P = 0.43); I^2 = 0\%$						
Test for overall effect: Z = 5.45 (P < 0.00001)						
**17.3.3 Long duration**						
**Subtotal (95% CI)**		**0**		**0**		**Not estimable**
Total events	0		0			
Heterogeneity: Not applicable						
Test for overall effect: Not applicable						
**Total (95% CI)**		**1026**		**757**	**100.0%**	**2.51 [1.94, 3.24]**
Total events	194		66			
Heterogeneity: $Chi^2 = 13.01, df = 9 (P = 0.16); I^2 = 31\%$						
Test for overall effect: Z = 7.03 (P < 0.00001)						
Test for subgroup differences: $Chi^2 = 3.11, df = 1 (P = 0.08), I^2 = 67.9\%$						

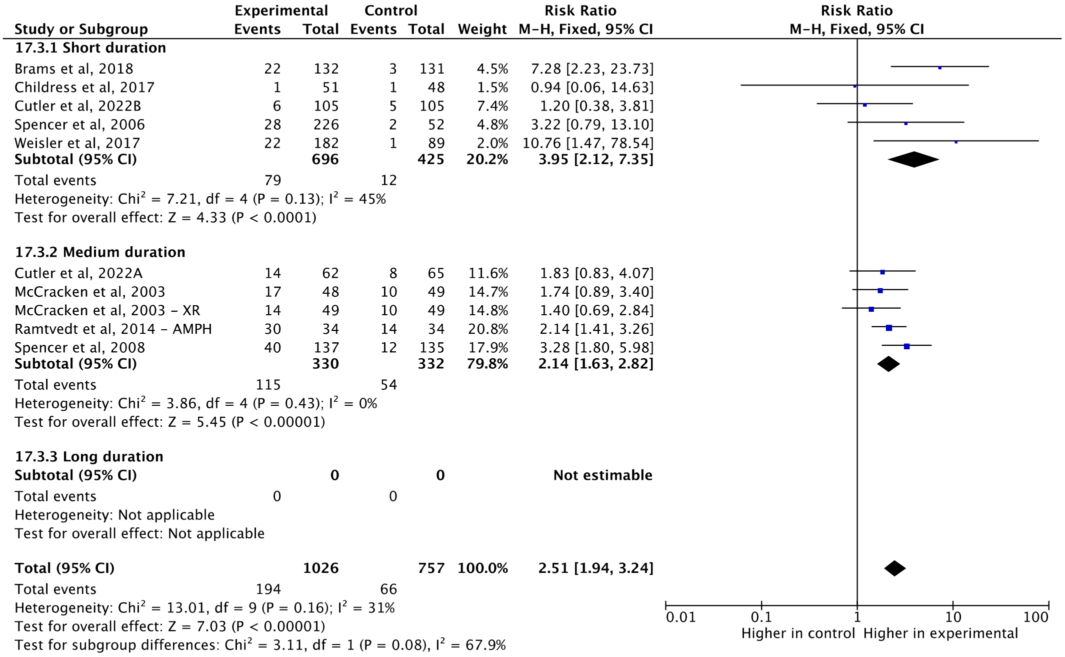

Figure legend: 95%CI, 95% confidence interval; AMPH, amphetamines; M-H, Mantel–Haenszel; XR, extended release. No study encompassing long duration was included in the meta-analysis.

eFigure 140. Forest plot showing the risk ratio of **irritability** between control and experimental groups in participants using **medium** dose **amphetamines** subdivided by duration of use.

Study or Subgroup	Experimental Events	Experimental Total	Control Events	Control Total	Weight	Risk Ratio M-H, Random, 95% CI
**17.7.1 Short duration**						
Brams et al, 2018	9	132	2	131	5.7%	4.47 [0.98, 20.28]
Cutler et al, 2022B	2	105	1	105	2.6%	2.00 [0.18, 21.72]
**Subtotal (95% CI)**		**237**		**236**	**8.2%**	**3.55 [0.99, 12.73]**
Total events	11		3			
Heterogeneity: Tau² = 0.00; Chi² = 0.31, df = 1 (P = 0.58); I² = 0%						
Test for overall effect: Z = 1.94 (P = 0.05)						
**17.7.2 Medium duration**						
Cutler et al, 2022A	11	62	5	65	10.4%	2.31 [0.85, 6.26]
McCracken et al, 2003	22	48	29	49	23.0%	0.77 [0.53, 1.14]
McCracken et al, 2003 - XR	21	49	29	49	22.7%	0.72 [0.49, 1.08]
Ramtvedt et al, 2014 - AMPH	23	34	27	34	25.4%	0.85 [0.64, 1.14]
Spencer et al, 2008	13	137	5	135	10.3%	2.56 [0.94, 6.99]
**Subtotal (95% CI)**		**330**		**332**	**91.8%**	**0.97 [0.68, 1.39]**
Total events	90		95			
Heterogeneity: Tau² = 0.09; Chi² = 10.31, df = 4 (P = 0.04); I² = 61%						
Test for overall effect: Z = 0.16 (P = 0.88)						
**17.7.3 Long duration**						
**Subtotal (95% CI)**		**0**		**0**		**Not estimable**
Total events	0		0			
Heterogeneity: Not applicable						
Test for overall effect: Not applicable						
**Total (95% CI)**		**567**		**568**	**100.0%**	**1.12 [0.75, 1.67]**
Total events	101		98			
Heterogeneity: Tau² = 0.14; Chi² = 16.60, df = 6 (P = 0.01); I² = 64%						
Test for overall effect: Z = 0.56 (P = 0.58)						
Test for subgroup differences: Chi² = 3.66, df = 1 (P = 0.06), I² = 72.7%						

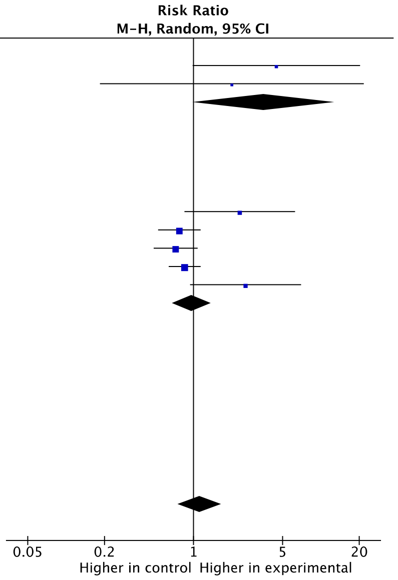

Figure legend: 95%CI, 95% confidence interval; AMPH, amphetamines; M-H, Mantel–Haenszel; XR, extended release. No study encompassing long duration was included in the meta-analysis.

eFigure 141. Forest plot showing the risk ratio of **nausea** between control and experimental groups in participants using **medium** dose **amphetamines** subdivided by duration of use.

Study or Subgroup	Experimental Events	Experimental Total	Control Events	Control Total	Weight	Risk Ratio M-H, Fixed, 95% CI
**17.4.1 Short duration**						
Weisler et al, 2017	10	182	1	89	4.2%	4.89 [0.64, 37.61]
**Subtotal (95% CI)**		**182**		**89**	**4.2%**	**4.89 [0.64, 37.61]**
Total events	10		1			
Heterogeneity: Not applicable						
Test for overall effect: Z = 1.52 (P = 0.13)						
**17.4.2 Medium duration**						
McCracken et al, 2003	11	48	10	49	30.7%	1.12 [0.53, 2.40]
McCracken et al, 2003 - XR	9	49	10	49	31.0%	0.90 [0.40, 2.02]
Ramtvedt et al, 2014 - AMPH	8	34	7	34	21.7%	1.14 [0.47, 2.80]
Spencer et al, 2008	9	137	4	135	12.5%	2.22 [0.70, 7.03]
**Subtotal (95% CI)**		**268**		**267**	**95.8%**	**1.20 [0.78, 1.85]**
Total events	37		31			
Heterogeneity: $Chi^2 = 1.61$, df = 3 (P = 0.66); $I^2 = 0\%$						
Test for overall effect: Z = 0.82 (P = 0.41)						
**17.4.3 Long duration**						
**Subtotal (95% CI)**		**0**		**0**		**Not estimable**
Total events	0		0			
Heterogeneity: Not applicable						
Test for overall effect: Not applicable						
**Total (95% CI)**		**450**		**356**	**100.0%**	**1.35 [0.89, 2.06]**
Total events	47		32			
Heterogeneity: $Chi^2 = 3.57$, df = 4 (P = 0.47); $I^2 = 0\%$						
Test for overall effect: Z = 1.40 (P = 0.16)						
Test for subgroup differences: $Chi^2 = 1.75$, df = 1 (P = 0.19), $I^2 = 42.8\%$						

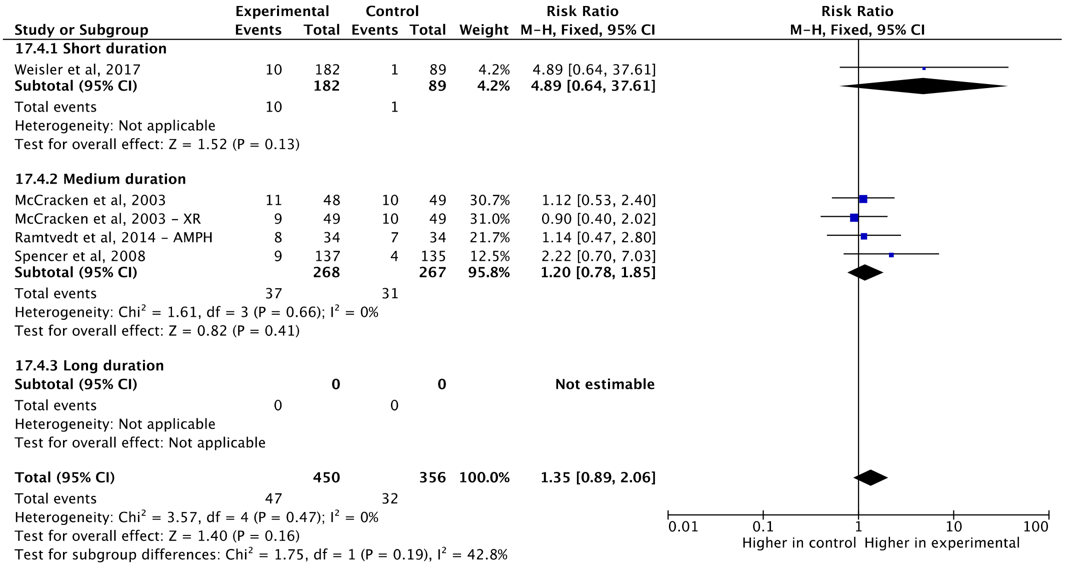

Figure legend: 95%CI, 95% confidence interval; AMPH, amphetamines; M-H, Mantel–Haenszel; XR, extended release. No study encompassing long duration was included in the meta-analysis.

eFigure 142. Forest plot showing the mean differences in **heart rate** between control and experimental groups in participants using **medium** dose **amphetamines** subdivided by duration of use.

	Experimental			Control				Mean Difference
**Study or Subgroup**	**Mean**	**SD**	**Total**	**Mean**	**SD**	**Total**	**Weight**	**IV, Fixed, 95% CI**
**17.11.1 Short duration**								
Brams et al, 2008	5.7	11.78	132	0.7	10.79	131	24.5%	5.00 [2.27, 7.73]
Cutler et al, 2022B	13.4	13.7	105	12.8	12.9	105	14.1%	0.60 [−3.00, 4.20]
Weisler et al, 2017	3.3	10.52	182	0.1	8.35	89	34.1%	3.20 [0.89, 5.51]
**Subtotal (95% CI)**			**419**			**325**	**72.6%**	**3.30 [1.72, 4.89]**
Heterogeneity: $Chi^2 = 3.66$, df = 2 (P = 0.16); $I^2 = 45\%$								
Test for overall effect: Z = 4.09 (P < 0.0001)								
**17.11.2 Medium duration**								
Spencer et al, 2008	4.7	11.1	136	0.4	10.5	133	27.4%	4.30 [1.72, 6.88]
**Subtotal (95% CI)**			**136**			**133**	**27.4%**	**4.30 [1.72, 6.88]**
Heterogeneity: Not applicable								
Test for overall effect: Z = 3.26 (P = 0.001)								
**17.11.3 Long duration**								
**Subtotal (95% CI)**			**0**			**0**		**Not estimable**
Heterogeneity: Not applicable								
Test for overall effect: Not applicable								
**Total (95% CI)**			**555**			**458**	**100.0%**	**3.58 [2.23, 4.93]**
Heterogeneity: $Chi^2 = 4.08$, df = 3 (P = 0.25); $I^2 = 26\%$								
Test for overall effect: Z = 5.19 (P < 0.00001)								
Test for subgroup differences: $Chi^2 = 0.42$, df = 1 (P = 0.52), $I^2 = 0\%$								

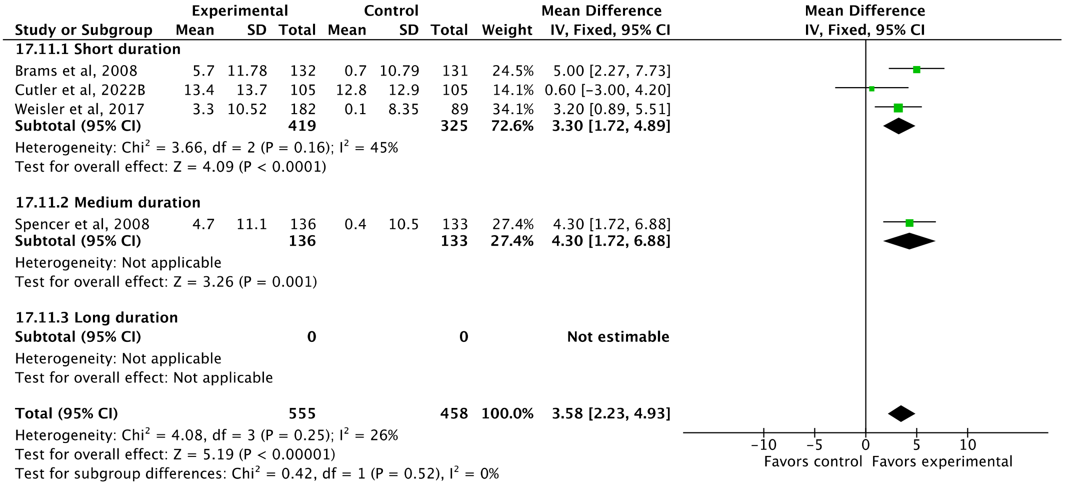

Figure legend: 95%CI, 95% confidence interval; IV, inverse variance; SD, standard deviation. No study encompassing long duration was included in the meta-analysis.

eFigure 143. Forest plot showing the mean differences in **diastolic blood pressure** between control and experimental groups in participants using **medium** dose **amphetamines** subdivided by duration of use.

	Experimental			Control				Mean Difference
**Study or Subgroup**	**Mean**	**SD**	**Total**	**Mean**	**SD**	**Total**	**Weight**	**IV, Fixed, 95% CI**
**17.10.1 Short duration**								
Brams et al, 2008	4	8.23	132	0.5	7.45	131	25.4%	3.50 [1.60, 5.40]
Cutler et al, 2022B	3.4	9.9	105	1.8	9.9	105	12.8%	1.60 [−1.08, 4.28]
Weisler et al, 2017	1	7.46	182	0.1	7.72	89	24.4%	0.90 [−1.04, 2.84]
**Subtotal (95% CI)**			**419**			**325**	**62.6%**	**2.10 [0.89, 3.31]**
Heterogeneity: $Chi^2 = 3.70$, df = 2 (P = 0.16); $I^2 = 46\%$								
Test for overall effect: Z = 3.40 (P = 0.0007)								
**17.10.2 Medium duration**								
Spencer et al, 2008	1.8	6.7	136	1.1	6.4	133	37.4%	0.70 [−0.87, 2.27]
**Subtotal (95% CI)**			**136**			**133**	**37.4%**	**0.70 [−0.87, 2.27]**
Heterogeneity: Not applicable								
Test for overall effect: Z = 0.88 (P = 0.38)								
**17.10.3 Long duration**								
**Subtotal (95% CI)**			**0**			**0**		**Not estimable**
Heterogeneity: Not applicable								
Test for overall effect: Not applicable								
**Total (95% CI)**			**555**			**458**	**100.0%**	**1.58 [0.62, 2.53]**
Heterogeneity: $Chi^2 = 5.62$, df = 3 (P = 0.13); $I^2 = 47\%$								
Test for overall effect: Z = 3.23 (P = 0.001)								
Test for subgroup differences: $Chi^2 = 1.92$, df = 1 (P = 0.17), $I^2 = 47.9\%$								

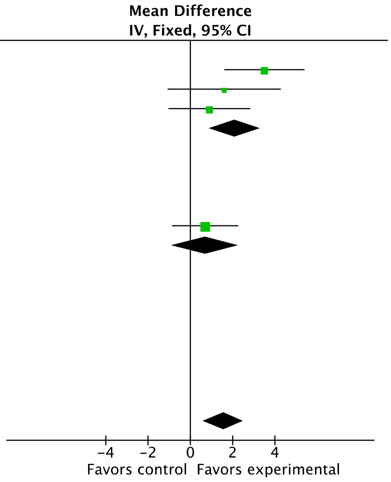

Figure legend: 95%CI, 95% confidence interval; IV, inverse variance; SD, standard deviation. No study encompassing long duration was included in the meta-analysis.

eFigure 144. Forest plot showing the mean differences in **systolic blood pressure** between control and experimental groups in participants using **medium** dose **amphetamines** subdivided by duration of use.

Study or Subgroup	Experimental Mean	Experimental SD	Experimental Total	Control Mean	Control SD	Control Total	Weight	Mean Difference IV, Fixed, 95% CI
**17.9.1 Short duration**								
Brams et al, 2008	3.8	9.15	132	2.1	8.72	131	30.0%	1.70 [−0.46, 3.86]
Cutler et al, 2022B	3.3	11	105	3.1	10.8	105	16.1%	0.20 [−2.75, 3.15]
Weisler et al, 2017	0.2	7.24	182	−0.8	9.99	89	25.9%	1.00 [−1.33, 3.33]
**Subtotal (95% CI)**			**419**			**325**	**72.0%**	**1.11 [−0.28, 2.51]**
Heterogeneity: $Chi^2 = 0.66$, df = 2 (P = 0.72); $I^2 = 0\%$								
Test for overall effect: Z = 1.56 (P = 0.12)								
**17.9.2 Medium duration**								
Spencer et al, 2008	1.3	9.3	136	0.2	9.4	133	28.0%	1.10 [−1.13, 3.33]
**Subtotal (95% CI)**			**136**			**133**	**28.0%**	**1.10 [−1.13, 3.33]**
Heterogeneity: Not applicable								
Test for overall effect: Z = 0.96 (P = 0.33)								
**17.9.3 Long duration**								
**Subtotal (95% CI)**			**0**			**0**		**Not estimable**
Heterogeneity: Not applicable								
Test for overall effect: Not applicable								
**Total (95% CI)**			**555**			**458**	**100.0%**	**1.11 [−0.07, 2.29]**
Heterogeneity: $Chi^2 = 0.66$, df = 3 (P = 0.88); $I^2 = 0\%$								
Test for overall effect: Z = 1.84 (P = 0.07)								
Test for subgroup differences: $Chi^2 = 0.00$, df = 1 (P = 0.99), $I^2 = 0\%$								

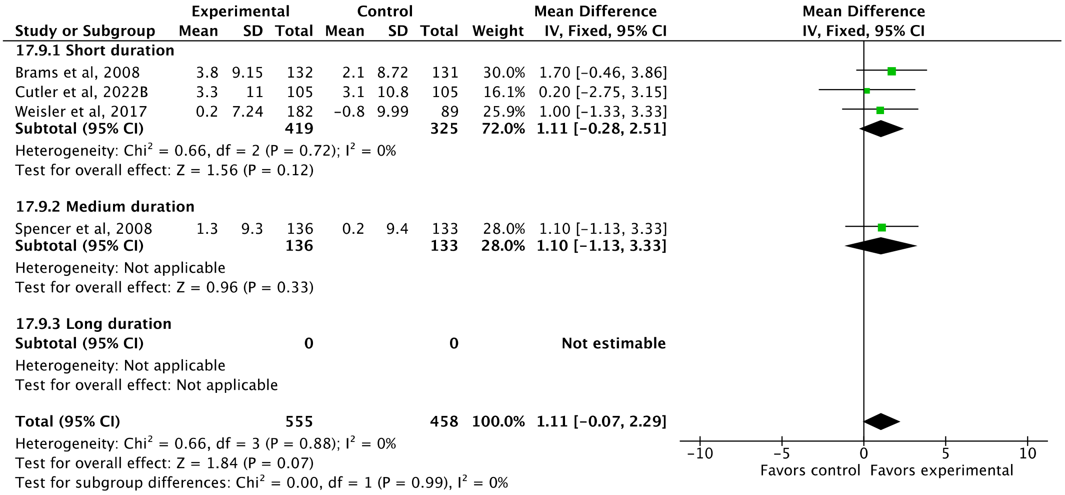

Figure legend: 95%CI, 95% confidence interval; IV, inverse variance; SD, standard deviation. No study encompassing long duration was included in the meta-analysis.

eFigure 145. Word cloud

Word cloud containing some of the most common reported adverse events after the use of stimulants.

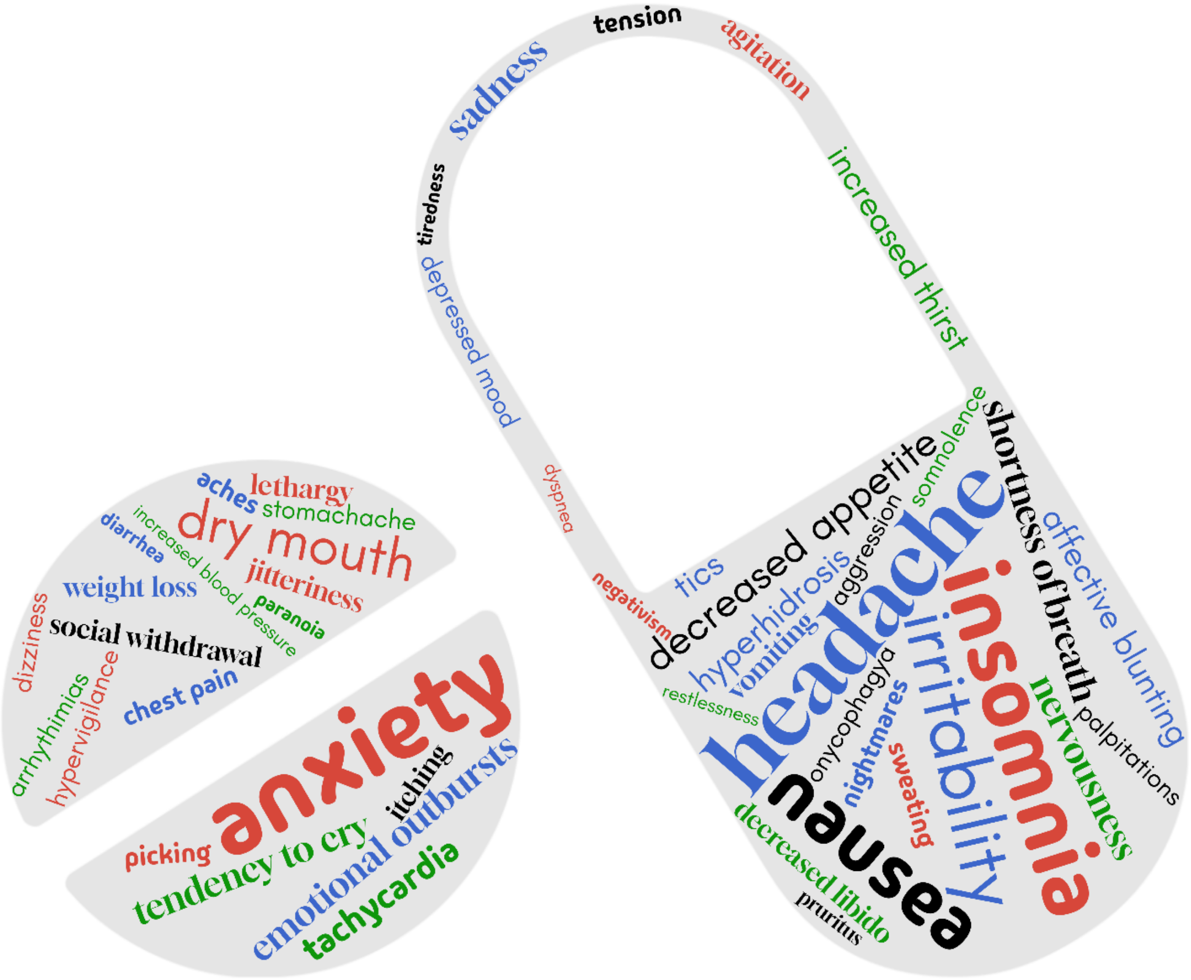

eReferences

1. Adler LA, Goodman DW, Kollins SH, et al. Double-blind, placebo-controlled study of the efficacy and safety of lisdexamfetamine dimesylate in adults with attention-deficit/hyperactivity disorder. *J Clin Psychiatry*. Sep 2008;69(9):1364-73. doi:10.4088/jcp.v69n0903
2. Adler LA, Weisler RH, Goodman DW, Hamdani M, Niebler GE. Short-term effects of lisdexamfetamine dimesylate on cardiovascular parameters in a 4-week clinical trial in adults with attention-deficit/hyperactivity disorder. *J Clin Psychiatry*. Dec 2009;70(12):1652-61. doi:10.4088/JCP.09m05335pur
3. Adler LA, Zimmerman B, Starr HL, et al. Efficacy and safety of OROS methylphenidate in adults with attention-deficit/hyperactivity disorder: a randomized, placebo-controlled, double-blind, parallel group, dose-escalation study. *J Clin Psychopharmacol*. Jun 2009;29(3):239-47. doi:10.1097/JCP.0b013e3181a390ce
4. Adler LA, Dirks B, Deas P, et al. Self-Reported quality of life in adults with attention-deficit/hyperactivity disorder and executive function impairment treated with lisdexamfetamine dimesylate: a randomized, double-blind, multicenter, placebo-controlled, parallel-group study. *BMC Psychiatry*. Oct 9 2013;13:253. doi:10.1186/1471-244x-13-253
5. Ahmann PA, Theye FW, Berg R, Linquist AJ, Van Erem AJ, Campbell LR. Placebo-controlled evaluation of amphetamine mixture-dextroamphetamine salts and amphetamine salts (Adderall): efficacy rate and side effects. *Pediatrics*. Jan 2001;107(1):E10. doi:10.1542/peds.107.1.e10
6. Armstrong RB, Damaraju CV, Ascher S, Schwarzman L, O'Neill J, Starr HL. Time course of treatment effect of OROS® methylphenidate in children with ADHD. *J Atten Disord*. Nov 2012;16(8):697-705. doi:10.1177/1087054711425772
7. Biederman J, Mick E, Surman C, et al. A randomized, placebo-controlled trial of OROS methylphenidate in adults with attention-deficit/hyperactivity disorder. *Biol Psychiatry*. May 1 2006;59(9):829-35. doi:10.1016/j.biopsych.2005.09.011
8. Biederman J, Boellner SW, Childress A, Lopez FA, Krishnan S, Zhang Y. Lisdexamfetamine dimesylate and mixed amphetamine salts extended-release in children with ADHD: a double-blind, placebo-controlled, crossover analog classroom study. *Biol Psychiatry*. Nov 1 2007;62(9):970-6. doi:10.1016/j.biopsych.2007.04.015
9. Biederman J, Krishnan S, Zhang Y, McGough JJ, Findling RL. Efficacy and tolerability of lisdexamfetamine dimesylate (NRP-104) in children with attention-deficit/hyperactivity disorder: a phase III, multicenter, randomized, double-blind, forced-dose, parallel-group study. *Clin Ther*. Mar 2007;29(3):450-63. doi:10.1016/s0149-2918(07)80083-x
10. Biederman J, Quinn D, Weiss M, et al. Efficacy and safety of Ritalin LA, a new, once daily, extended-release dosage form of methylphenidate, in children with attention deficit hyperactivity disorder. *Paediatr Drugs*. 2003;5(12):833-41. doi:10.2165/00148581-200305120-00006
11. Bouffard R, Hechtman L, Minde K, Iaboni-Kassab F. The efficacy of 2 different dosages of methylphenidate in treating adults with attention-deficit hyperactivity disorder. *Can J Psychiatry*. Sep 2003;48(8):546-54. doi:10.1177/070674370304800806

12. Brams M, Muniz R, Childress A, et al. A randomized, double-blind, crossover study of once-daily dexmethylphenidate in children with attention-deficit hyperactivity disorder: rapid onset of effect. *CNS Drugs*. 2008;22(8):693-704. doi:10.2165/00023210-200822080-00006
13. Brams M, Giblin J, Gasior M, Gao J, Wigal T. Effects of open-label lisdexamfetamine dimesylate on self-reported quality of life in adults with ADHD. *Postgrad Med*. May 2011;123(3):99-108. doi:10.3810/pgm.2011.05.2288
14. Brams M, Childress AC, Greenbaum M, et al. SHP465 Mixed Amphetamine Salts in the Treatment of Attention-Deficit/Hyperactivity Disorder in Children and Adolescents: Results of a Randomized, Double-Blind Placebo-Controlled Study. *J Child Adolesc Psychopharmacol*. Feb 2018;28(1):19-28. doi:10.1089/cap.2017.0053
15. Bron TI, Bijlenga D, Boonstra AM, et al. OROS-methylphenidate efficacy on specific executive functioning deficits in adults with ADHD: a randomized, placebo-controlled cross-over study. *Eur Neuropsychopharmacol*. Apr 2014;24(4):519-28. doi:10.1016/j.euroneuro.2014.01.007
16. Brown TE, Brams M, Gao J, Gasior M, Childress A. Open-label administration of lisdexamfetamine dimesylate improves executive function impairments and symptoms of attention-deficit/hyperactivity disorder in adults. *Postgrad Med*. Sep 2010;122(5):7-17. doi:10.3810/pgm.2010.09.2196
17. Buitelaar JK, Trott GE, Hofecker M, et al. Long-term efficacy and safety outcomes with OROS-MPH in adults with ADHD. *Int J Neuropsychopharmacol*. Feb 2012;15(1):1-13. doi:10.1017/s1461145711001131
18. Casas M, Rösler M, Sandra Kooij JJ, et al. Efficacy and safety of prolonged-release OROS methylphenidate in adults with attention deficit/hyperactivity disorder: a 13-week, randomized, double-blind, placebo-controlled, fixed-dose study. *World J Biol Psychiatry*. May 2013;14(4):268-81. doi:10.3109/15622975.2011.600333
19. Childress AC, Spencer T, Lopez F, et al. Efficacy and safety of dexmethylphenidate extended-release capsules administered once daily to children with attention-deficit/hyperactivity disorder. *J Child Adolesc Psychopharmacol*. Aug 2009;19(4):351-61. doi:10.1089/cap.2009.0007
20. Childress AC, Brams M, Cutler AJ, et al. The Efficacy and Safety of Evekeo, Racemic Amphetamine Sulfate, for Treatment of Attention-Deficit/Hyperactivity Disorder Symptoms: A Multicenter, Dose-Optimized, Double-Blind, Randomized, Placebo-Controlled Crossover Laboratory Classroom Study. *J Child Adolesc Psychopharmacol*. Jun 2015;25(5):402-14. doi:10.1089/cap.2014.0176
21. Childress AC, Wigal SB, Brams MN, et al. Efficacy and Safety of Amphetamine Extended-Release Oral Suspension in Children with Attention-Deficit/Hyperactivity Disorder. *J Child Adolesc Psychopharmacol*. Jun 2018;28(5):306-313. doi:10.1089/cap.2017.0095
22. Childress AC, Cutler AJ, Marraffino A, et al. A Randomized, Double-Blind, Placebo-Controlled Study of HLD200, a Delayed-Release and Extended-Release Methylphenidate, in Children with Attention-Deficit/Hyperactivity Disorder: An Evaluation of Safety and Efficacy Throughout the Day and Across Settings. *J Child Adolesc Psychopharmacol*. Feb 2020;30(1):2-14. doi:10.1089/cap.2019.0070
23. Childress AC, Brams MN, Cutler AJ, Donnelly GAE, Bhaskar S. Efficacy and Safety of Multilayer, Extended-Release Methylphenidate (PRC-063) in

Children 6-12 Years of Age with Attention-Deficit/Hyperactivity Disorder: A Laboratory Classroom Study. *J Child Adolesc Psychopharmacol*. Dec 2020;30(10):580-589. doi:10.1089/cap.2020.0109
24. Childress AC, Kollins SH, Foehl HC, et al. Randomized, Double-Blind, Placebo-Controlled, Flexible-Dose Titration Study of Methylphenidate Hydrochloride Extended-Release Capsules (Aptensio XR) in Preschool Children with Attention-Deficit/Hyperactivity Disorder. *J Child Adolesc Psychopharmacol*. Mar 2020;30(2):58-68. doi:10.1089/cap.2019.0085
25. Childress A, Cutler AJ, Marraffino AH, Bhaskar S, Donnelly G. Randomized, Double-Blind, Placebo-Controlled, Parallel-Group, Adult Laboratory Classroom Study of the Efficacy and Safety of PRC-063 (Extended-Release Methylphenidate) for the Treatment of ADHD. *J Atten Disord*. Apr 2022;26(6):857-869. doi:10.1177/10870547211025610
26. Childress AC, Lloyd E, Jacobsen L, Gunawardhana L, Johnson SA, Jr., Findling RL. Efficacy and Safety of Lisdexamfetamine in Preschool Children With Attention-Deficit/Hyperactivity Disorder. *J Am Acad Child Adolesc Psychiatry*. Dec 2022;61(12):1423-1434. doi:10.1016/j.jaac.2022.03.034
27. Chronis-Tuscano A, Seymour KE, Stein MA, et al. Efficacy of osmotic-release oral system (OROS) methylphenidate for mothers with attention-deficit/hyperactivity disorder (ADHD): preliminary report of effects on ADHD symptoms and parenting. *J Clin Psychiatry*. Dec 2008;69(12):1938-47. doi:10.4088/jcp.v69n1213
28. Coghill D, Banaschewski T, Lecendreux M, et al. European, randomized, phase 3 study of lisdexamfetamine dimesylate in children and adolescents with attention-deficit/hyperactivity disorder. *Eur Neuropsychopharmacol*. Oct 2013;23(10):1208-18. doi:10.1016/j.euroneuro.2012.11.012
29. Coghill DR, Banaschewski T, Lecendreux M, et al. Maintenance of efficacy of lisdexamfetamine dimesylate in children and adolescents with attention-deficit/hyperactivity disorder: randomized-withdrawal study design. *J Am Acad Child Adolesc Psychiatry*. Jun 2014;53(6):647-657.e1. doi:10.1016/j.jaac.2014.01.017
30. Cutler AJ, Childress AC, Pardo A, et al. Randomized, Double-Blind, Placebo-Controlled, Fixed-Dose Study to Evaluate the Efficacy and Safety of Amphetamine Extended-Release Tablets in Adults With Attention-Deficit/Hyperactivity Disorder. *J Clin Psychiatry*. Jul 20 2022;83(5)doi:10.4088/JCP.22m14438
31. Cutler AJ, Suzuki K, Starling B, et al. Efficacy and Safety of Dextroamphetamine Transdermal System for the Treatment of Attention-Deficit/Hyperactivity Disorder in Children and Adolescents: Results from a Pivotal Phase 2 Study. *J Child Adolesc Psychopharmacol*. Mar 2022;32(2):89-97. doi:10.1089/cap.2021.0107
32. Dupaul GJ, Weyandt LL, Rossi JS, et al. Double-blind, placebo-controlled, crossover study of the efficacy and safety of lisdexamfetamine dimesylate in college students with ADHD. *J Atten Disord*. Apr 2012;16(3):202-20. doi:10.1177/1087054711427299
33. Ermer J, Martin P, Corcoran M, Matsuo Y. A phase 1, randomized, double-blind, placebo-controlled study to evaluate the safety, tolerability, and pharmacokinetics of single and multiple doses of lisdexamfetamine dimesylate in Japanese and Caucasian healthy adult subjects. *Neuropsychopharmacol Rep*. Mar 2020;40(1):16-29. doi:10.1002/npr2.12082

34. Faraone SV, Childress A, Caras S, et al. A Randomized, Double-Blind, Placebo-Controlled Trial to Evaluate the Efficacy and Safety of AR19, a Manipulation-Resistant Formulation of Amphetamine Sulfate, in Adults With Attention-Deficit/Hyperactivity Disorder. *J Clin Psychiatry*. Aug 24 2021;82(5)doi:10.4088/JCP.21m13927
35. Findling RL, Bukstein OG, Melmed RD, et al. A randomized, double-blind, placebo-controlled, parallel-group study of methylphenidate transdermal system in pediatric patients with attention-deficit/hyperactivity disorder. *J Clin Psychiatry*. Jan 2008;69(1):149-59. doi:10.4088/jcp.v69n0120
36. Findling RL, Childress AC, Cutler AJ, et al. Efficacy and safety of lisdexamfetamine dimesylate in adolescents with attention-deficit/hyperactivity disorder. *J Am Acad Child Adolesc Psychiatry*. Apr 2011;50(4):395-405. doi:10.1016/j.jaac.2011.01.007
37. Froehlich TE, Brinkman WB, Peugh JL, Piedra AN, Vitucci DJ, Epstein JN. Pre-Existing Comorbid Emotional Symptoms Moderate Short-Term Methylphenidate Adverse Effects in a Randomized Trial of Children with Attention-Deficit/Hyperactivity Disorder. *J Child Adolesc Psychopharmacol*. Apr 2020;30(3):137-147. doi:10.1089/cap.2019.0125
38. Galloway GP, Buscemi R, Coyle JR, et al. A randomized, placebo-controlled trial of sustained-release dextroamphetamine for treatment of methamphetamine addiction. *Clin Pharmacol Ther*. Feb 2011;89(2):276-82. doi:10.1038/clpt.2010.307
39. Ginsberg Y, Lindefors N. Methylphenidate treatment of adult male prison inmates with attention-deficit hyperactivity disorder: randomised double-blind placebo-controlled trial with open-label extension. *Br J Psychiatry*. Jan 2012;200(1):68-73. doi:10.1192/bjp.bp.111.092940
40. Ginsberg Y, Arngrim T, Philipsen A, et al. Long-term (1 year) safety and efficacy of methylphenidate modified-release long-acting formulation (MPH-LA) in adults with attention-deficit hyperactivity disorder: a 26-week, flexible-dose, open-label extension to a 40-week, double-blind, randomised, placebo-controlled core study. *CNS Drugs*. Oct 2014;28(10):951-62. doi:10.1007/s40263-014-0180-4
41. Goodman DW, Starr HL, Ma YW, Rostain AL, Ascher S, Armstrong RB. Randomized, 6-Week, Placebo-Controlled Study of Treatment for Adult Attention-Deficit/Hyperactivity Disorder: Individualized Dosing of Osmotic-Release Oral System (OROS) Methylphenidate With a Goal of Symptom Remission. *J Clin Psychiatry*. Jan 2017;78(1):105-114. doi:10.4088/JCP.15m10348
42. Greenhill LL, Findling RL, Swanson JM. A double-blind, placebo-controlled study of modified-release methylphenidate in children with attention-deficit/hyperactivity disorder. *Pediatrics*. Mar 2002;109(3):E39. doi:10.1542/peds.109.3.e39
43. Hegerl U, Mergl R, Sander C, et al. A multi-centre, randomised, double-blind, placebo-controlled clinical trial of methylphenidate in the initial treatment of acute mania (MEMAP study). *Eur Neuropsychopharmacol*. Jan 2018;28(1):185-194. doi:10.1016/j.euroneuro.2017.11.003
44. Huang YS, Yeh CB, Chen CH, Shang CY, Gau SS. A Randomized, Double-Blind, Placebo-Controlled, Two-Way Crossover Clinical Trial of ORADUR-Methylphenidate for Treating Children and Adolescents with

Attention-Deficit/Hyperactivity Disorder. *J Child Adolesc Psychopharmacol*. Apr 2021;31(3):164-178. doi:10.1089/cap.2020.0104
45. Huss M, Ginsberg Y, Tvedten T, et al. Methylphenidate hydrochloride modified-release in adults with attention deficit hyperactivity disorder: a randomized double-blind placebo-controlled trial. *Adv Ther*. Jan 2014;31(1):44-65. doi:10.1007/s12325-013-0085-5
46. Jain U, Hechtman L, Weiss M, et al. Efficacy of a novel biphasic controlled-release methylphenidate formula in adults with attention-deficit/hyperactivity disorder: results of a double-blind, placebo-controlled crossover study. *J Clin Psychiatry*. Feb 2007;68(2):268-77. doi:10.4088/jcp.v68n0213
47. Jasinski D, Krishnan S. Abuse liability and safety of oral lisdexamfetamine dimesylate in individuals with a history of stimulant abuse. *Journal of Psychopharmacology*. 2009;23(4):419-427. doi:10.1177/0269881109103113
48. Kis B, Lucke C, Abdel-Hamid M, et al. Safety Profile of Methylphenidate Under Long-Term Treatment in Adult ADHD Patients - Results of the COMPAS Study. *Pharmacopsychiatry*. Nov 2020;53(6):263-271. doi:10.1055/a-1207-9851
49. Konstenius M, Jayaram-Lindström N, Guterstam J, Beck O, Philips B, Franck J. Methylphenidate for attention deficit hyperactivity disorder and drug relapse in criminal offenders with substance dependence: a 24-week randomized placebo-controlled trial. *Addiction*. Mar 2014;109(3):440-9. doi:10.1111/add.12369
50. Kooij JJ, Burger H, Boonstra AM, Van der Linden PD, Kalma LE, Buitelaar JK. Efficacy and safety of methylphenidate in 45 adults with attention-deficit/hyperactivity disorder. A randomized placebo-controlled double-blind cross-over trial. *Psychol Med*. Aug 2004;34(6):973-82. doi:10.1017/s0033291703001776
51. Lee J, Grizenko N, Bhat V, Sengupta S, Polotskaia A, Joober R. Relation between therapeutic response and side effects induced by methylphenidate as observed by parents and teachers of children with ADHD. *BMC Psychiatry*. Apr 21 2011;11:70. doi:10.1186/1471-244x-11-70
52. Ling W, Chang L, Hillhouse M, et al. Sustained-release methylphenidate in a randomized trial of treatment of methamphetamine use disorder. *Addiction*. Sep 2014;109(9):1489-500. doi:10.1111/add.12608
53. Lopez FA, Ginsberg LD, Arnold V. Effect of lisdexamfetamine dimesylate on parent-rated measures in children aged 6 to 12 years with attention-deficit/hyperactivity disorder: a secondary analysis. *Postgrad Med*. Sep 2008;120(3):89-102. doi:10.3810/pgm.2008.09.1910
54. Martin P, Dirks B, Gertsik L, et al. Safety and pharmacokinetics of lisdexamfetamine dimesylate in adults with clinically stable schizophrenia: a randomized, double-blind, placebo-controlled trial of ascending multiple doses. *J Clin Psychopharmacol*. Dec 2014;34(6):682-9. doi:10.1097/jcp.0000000000000205
55. Martin PT, Corcoran M, Zhang P, Katic A. Randomized, double-blind, placebo-controlled, crossover study of the effects of lisdexamfetamine dimesylate and mixed amphetamine salts on cognition throughout the day in adults with attention-deficit/hyperactivity disorder. *Clin Drug Investig*. Feb 2014;34(2):147-57. doi:10.1007/s40261-013-0156-z

56. Mattingly G, Arnold V, Yan B, Yu M, Robertson B. A Phase 3, Randomized Double-Blind Study of the Efficacy and Safety of Low-Dose SHP465 Mixed Amphetamine Salts Extended-Release in Children with Attention-Deficit/Hyperactivity Disorder. *J Child Adolesc Psychopharmacol*. Nov 2020;30(9):549-557. doi:10.1089/cap.2020.0005
57. McElroy SL, Hudson JI, Mitchell JE, et al. Efficacy and safety of lisdexamfetamine for treatment of adults with moderate to severe binge-eating disorder: a randomized clinical trial. *JAMA Psychiatry*. Mar 2015;72(3):235-46. doi:10.1001/jamapsychiatry.2014.2162
58. Medori R, Ramos-Quiroga JA, Casas M, et al. A randomized, placebo-controlled trial of three fixed dosages of prolonged-release OROS methylphenidate in adults with attention-deficit/hyperactivity disorder. *Biol Psychiatry*. May 15 2008;63(10):981-9. doi:10.1016/j.biopsych.2007.11.008
59. McCracken JT, Biederman J, Greenhill LL, et al. Analog classroom assessment of a once-daily mixed amphetamine formulation, SLI381 (Adderall XR), in children with ADHD. *J Am Acad Child Adolesc Psychiatry*. Jun 2003;42(6):673-83. doi:10.1097/01.Chi.0000046863.56865.Fe
60. Mooney ME, Herin DV, Specker S, Babb D, Levin FR, Grabowski J. Pilot study of the effects of lisdexamfetamine on cocaine use: A randomized, double-blind, placebo-controlled trial. *Drug Alcohol Depend*. Aug 1 2015;153:94-103. doi:10.1016/j.drugalcdep.2015.05.042
61. Muniz R, Brams M, Mao A, McCague K, Pestreich L, Silva R. Efficacy and safety of extended-release dexmethylphenidate compared with d,l-methylphenidate and placebo in the treatment of children with attention-deficit/hyperactivity disorder: a 12-hour laboratory classroom study. *J Child Adolesc Psychopharmacol*. Jun 2008;18(3):248-56. doi:10.1089/cap.2007.0015
62. Newcorn JH, Kratochvil CJ, Allen AJ, et al. Atomoxetine and osmotically released methylphenidate for the treatment of attention deficit hyperactivity disorder: acute comparison and differential response. *Am J Psychiatry*. Jun 2008;165(6):721-30. doi:10.1176/appi.ajp.2007.05091676
63. Nuijten M, Blanken P, van de Wetering B, Nuijen B, van den Brink W, Hendriks VM. Sustained-release dexamfetamine in the treatment of chronic cocaine-dependent patients on heroin-assisted treatment: a randomised, double-blind, placebo-controlled trial. *Lancet*. May 28 2016;387(10034):2226-34. doi:10.1016/s0140-6736(16)00205-1
64. Patkar AA, Masand PS, Pae CU, et al. A randomized, double-blind, placebo-controlled trial of augmentation with an extended release formulation of methylphenidate in outpatients with treatment-resistant depression. *J Clin Psychopharmacol*. Dec 2006;26(6):653-6. doi:10.1097/01.jcp.0000246212.03530.fd
65. Pearson DA, Santos CW, Aman MG, et al. Effects of extended release methylphenidate treatment on ratings of attention-deficit/hyperactivity disorder (ADHD) and associated behavior in children with autism spectrum disorders and ADHD symptoms. *J Child Adolesc Psychopharmacol*. Jun 2013;23(5):337-51. doi:10.1089/cap.2012.0096
66. Pelham WE, Gnagy EM, Burrows-Maclean L, et al. Once-a-day Concerta methylphenidate versus three-times-daily methylphenidate in laboratory and natural settings. *Pediatrics*. Jun 2001;107(6):E105. doi:10.1542/peds.107.6.e105

67. Pelham WE, Jr., Manos MJ, Ezzell CE, et al. A dose-ranging study of a methylphenidate transdermal system in children with ADHD. *J Am Acad Child Adolesc Psychiatry*. Jun 2005;44(6):522-9. doi:10.1097/01.chi.0000157548.48960.95
68. Pliszka SR, Wilens TE, Bostrom S, et al. Efficacy and Safety of HLD200, Delayed-Release and Extended-Release Methylphenidate, in Children with Attention-Deficit/Hyperactivity Disorder. *J Child Adolesc Psychopharmacol*. Aug 2017;27(6):474-482. doi:10.1089/cap.2017.0084
69. Quinn D, Wigal S, Swanson J, et al. Comparative pharmacodynamics and plasma concentrations of d-threo-methylphenidate hydrochloride after single doses of d-threo-methylphenidate hydrochloride and d,l-threo-methylphenidate hydrochloride in a double-blind, placebo-controlled, crossover laboratory school study in children with attention-deficit/hyperactivity disorder. *J Am Acad Child Adolesc Psychiatry*. Nov 2004;43(11):1422-9. doi:10.1097/01.chi.0000140455.96946.2b
70. Ramtvedt BE, Aabech HS, Sundet K. Minimizing adverse events while maintaining clinical improvement in a pediatric attention-deficit/hyperactivity disorder crossover trial with dextroamphetamine and methylphenidate. *J Child Adolesc Psychopharmacol*. Apr 2014;24(3):130-9. doi:10.1089/cap.2013.0114
71. Retz W, Rösler M, Ose C, et al. Multiscale assessment of treatment efficacy in adults with ADHD: a randomized placebo-controlled, multi-centre study with extended-release methylphenidate. *World J Biol Psychiatry*. Jan 2012;13(1):48-59. doi:10.3109/15622975.2010.540257
72. Rosenberg PB, Lanctot KL, Drye LT, et al. Safety and efficacy of methylphenidate for apathy in Alzheimer's disease: a randomized, placebo-controlled trial. *J Clin Psychiatry*. Aug 2013;74(8):810-6. doi:10.4088/JCP.12m08099
73. Rösler M, Fischer R, Ammer R, Ose C, Retz W. A randomised, placebo-controlled, 24-week, study of low-dose extended-release methylphenidate in adults with attention-deficit/hyperactivity disorder. *Eur Arch Psychiatry Clin Neurosci*. Mar 2009;259(2):120-9. doi:10.1007/s00406-008-0845-4
74. Schulz E, Fleischhaker C, Hennighausen K, et al. A double-blind, randomized, placebo/active controlled crossover evaluation of the efficacy and safety of Ritalin ® LA in children with attention-deficit/hyperactivity disorder in a laboratory classroom setting. *J Child Adolesc Psychopharmacol*. Oct 2010;20(5):377-85. doi:10.1089/cap.2009.0106
75. Shram MJ, Setnik B, Webster L, et al. Oral, intranasal, and intravenous abuse potential of serdexmethylphenidate, a novel prodrug of d-methylphenidate. *Curr Med Res Opin*. Jul 2022;38(7):1237-1250. doi:10.1080/03007995.2022.2076474
76. Silva R, Muniz R, Pestreich LK, Brams M, Childress A, Lopez FA. Efficacy of two long-acting methylphenidate formulations in children with attention- deficit/hyperactivity disorder in a laboratory classroom setting. *J Child Adolesc Psychopharmacol*. Aug 2005;15(4):637-54. doi:10.1089/cap.2005.15.637
77. Spencer TJ, Wilens TE, Biederman J, Weisler RH, Read SC, Pratt R. Efficacy and safety of mixed amphetamine salts extended release (Adderall XR) in the management of attention-deficit/hyperactivity disorder in adolescent patients: a 4-week, randomized, double-blind, placebo-controlled, parallel-group study. *Clin Ther*. Feb 2006;28(2):266-79. doi:10.1016/j.clinthera.2006.02.011

78. Spencer TJ, Adler LA, McGough JJ, Muniz R, Jiang H, Pestreich L. Efficacy and safety of dexmethylphenidate extended-release capsules in adults with attention-deficit/hyperactivity disorder. *Biol Psychiatry*. Jun 15 2007;61(12):1380-7. doi:10.1016/j.biopsych.2006.07.032
79. Spencer TJ, Adler LA, Weisler RH, Youcha SH. Triple-bead mixed amphetamine salts (SPD465), a novel, enhanced extended-release amphetamine formulation for the treatment of adults with ADHD: a randomized, double-blind, multicenter, placebo-controlled study. *J Clin Psychiatry*. Sep 2008;69(9):1437-48. doi:10.4088/jcp.v69n0911
80. Stein MA, Sarampote CS, Waldman ID, et al. A dose-response study of OROS methylphenidate in children with attention-deficit/hyperactivity disorder. *Pediatrics*. Nov 2003;112(5):e404. doi:10.1542/peds.112.5.e404
81. Sugaya LS, Salum GA, de Sousa Gurgel W, et al. Efficacy and safety of methylphenidate and behavioural parent training for children aged 3-5 years with attention-deficit hyperactivity disorder: a randomised, double-blind, placebo-controlled, and sham behavioural parent training-controlled trial. *Lancet Child Adolesc Health*. Dec 2022;6(12):845-856. doi:10.1016/S2352-4642(22)00279-6
82. Takahashi N, Koh T, Tominaga Y, Saito Y, Kashimoto Y, Matsumura T. A randomized, double-blind, placebo-controlled, parallel-group study to evaluate the efficacy and safety of osmotic-controlled release oral delivery system methylphenidate HCl in adults with attention-deficit/hyperactivity disorder in Japan. *World J Biol Psychiatry*. Aug 2014;15(6):488-98. doi:10.3109/15622975.2013.868925
83. Weisler RH, Greenbaum M, Arnold V, et al. Efficacy and Safety of SHP465 Mixed Amphetamine Salts in the Treatment of Attention-Deficit/Hyperactivity Disorder in Adults: Results of a Randomized, Double-Blind, Placebo-Controlled, Forced-Dose Clinical Study. *Cns Drugs*. Aug 2017;31(8):685-697. doi:10.1007/s40263-017-0455-7
84. Weiss MD, Childress AC, Donnelly GAE. Efficacy and Safety of PRC-063, Extended-Release Multilayer Methylphenidate in Adults with ADHD Including 6-Month Open-Label Extension. *J Atten Disord*. Aug 2021;25(10):1417-1428. doi:10.1177/1087054719896853
85. Weiss MD, Cutler AJ, Kollins SH, Donnelly GAE. Efficacy and Safety of a Long-Acting Multilayer-Release Methylphenidate Formulation (PRC-063) in the Treatment of Adolescent Attention-Deficit/Hyperactivity Disorder: A Randomized, Double-Blind Clinical Trial with a 6-Month Open-Label Extension. *J Child Adolesc Psychopharmacol*. Nov 2021;31(9):610-622. doi:10.1089/cap.2021.0034
86. Wigal S, Swanson JM, Feifel D, et al. A double-blind, placebo-controlled trial of dexmethylphenidate hydrochloride and d,l-threo-methylphenidate hydrochloride in children with attention-deficit/hyperactivity disorder. *J Am Acad Child Adolesc Psychiatry*. Nov 2004;43(11):1406-14. doi:10.1097/01.chi.0000138351.98604.92
87. Wigal T, Greenhill L, Chuang S, et al. Safety and tolerability of methylphenidate in preschool children with ADHD. *J Am Acad Child Adolesc Psychiatry*. Nov 2006;45(11):1294-1303. doi:10.1097/01.chi.0000235082.63156.27
88. Wigal T, Brams M, Gasior M, Gao J, Squires L, Giblin J. Randomized, double-blind, placebo-controlled, crossover study of the efficacy and safety of

lisdexamfetamine dimesylate in adults with attention-deficit/hyperactivity disorder: novel findings using a simulated adult workplace environment design. *Behav Brain Funct*. Jun 24 2010;6:34. doi:10.1186/1744-9081-6-34
89. Wilens TE, Spencer TJ, Biederman J. Short- and Long-Term Cardiovascular Effects of Mixed Amphetamine Salts Extended-Release in Adolescents With ADHD. *CNS Spectrums*. 2005;10(S15):22-30. doi:10.1017/S1092852900014115
90. Wilens TE, McBurnett K, Bukstein O, et al. Multisite controlled study of OROS methylphenidate in the treatment of adolescents with attention-deficit/hyperactivity disorder. *Arch Pediatr Adolesc Med*. Jan 2006;160(1):82-90. doi:10.1001/archpedi.160.1.82
91. Winhusen TM, Lewis DF, Riggs PD, et al. Subjective effects, misuse, and adverse effects of osmotic-release methylphenidate treatment in adolescent substance abusers with attention-deficit/hyperactivity disorder. *J Child Adolesc Psychopharmacol*. Oct 2011;21(5):455-63. doi:10.1089/cap.2011.0014
92. Wolraich ML, Greenhill LL, Pelham W, et al. Randomized, controlled trial of oros methylphenidate once a day in children with attention-deficit/hyperactivity disorder. *Pediatrics*. Oct 2001;108(4):883-92. doi:10.1542/peds.108.4.883
93. Zheng Y, Liu H, Wang X, et al. Randomized, Double-Blind, Placebo-Controlled Trial on the Efficacy, Safety and Tolerability of Modified-Release Methylphenidate (MPH-MR) in Chinese Children and Adolescents with Attention-Deficit/Hyperactivity Disorder (ADHD). *CNS Drugs*. Dec 13 2024;doi:10.1007/s40263-024-01136-6
94. Kruschke JK. *Doing Bayesian Data Analysis: A Tutorial with R, JAGS, and Stan*. Academic Press; 2015.
95. van de Schoot R, Depaoli S, King R, et al. Bayesian statistics and modelling. *Nature Reviews Methods Primers*. 2021/01/14 2021;1(1):1. doi:10.1038/s43586-020-00001-2
96. Bürkner P-C. brms: An R Package for Bayesian Multilevel Models Using Stan. *Journal of Statistical Software*. 08/29 2017;80(1):1 - 28. doi:10.18637/jss.v080.i01
97. Carpenter B, Gelman A, Hoffman MD, et al. Stan: A Probabilistic Programming Language. *J Stat Softw*. 2017;76doi:10.18637/jss.v076.i01
98. Macri EM, Callaghan M, van Middelkoop M, Hattle M, Bierma-Zeinstra SMA. Effects of mechanical interventions in the management of knee osteoarthritis: protocol for an OA Trial Bank systematic review and individual participant data meta-analysis. *BMJ Open*. Feb 5 2021;11(2):e043026. doi:10.1136/bmjopen-2020-043026
