# Supplementary material for: Safety of Stimulants Across Patient Populations: A Meta-Analysis
Source: JAMA Netw Open. 2025 May 9;8(5):e259492. doi: 10.1001/jamanetworkopen.2025.9492 (PMC12065045; doi:10.1001/jamanetworkopen.2025.9492)
Supplement: Supplement 2. — Data Sharing Statement [file jamanetwopen-e259492-s002.pdf]

Data Sharing Statement

Oliva. Safety of Stimulants Across Patient Populations. *JAMA Netw Open*. Published May 09, 2025. doi:10.1001/jamanetworkopen.2025.9492

Data
Data available: Yes
Data types: Data (not involving human participants)
How to access data: Corresponding author, and supplementary materials.
When available: With publication

Supporting Documents
Document types: None

Additional Information
Who can access the data: anyone requesting the data
Types of analyses: for any purpose
Mechanisms of data availability: with investigator support
